# RNA Microarray Analysis of Macroscopically Normal Articular Cartilage from Knees Undergoing Partial Medial Meniscectomy: Potential Prediction of the Risk for Developing Osteoarthritis

**DOI:** 10.1371/journal.pone.0155373

**Published:** 2016-05-12

**Authors:** Muhammad Farooq Rai, Linda J. Sandell, Bo Zhang, Rick W. Wright, Robert H. Brophy

**Affiliations:** 1 Department of Orthopaedic Surgery, Musculoskeletal Research Center, Washington University School of Medicine at Barnes Jewish Hospital, St. Louis, Missouri, United States of America; 2 Department of Cell Biology and Physiology, Washington University School of Medicine at Barnes Jewish Hospital, St. Louis, Missouri, United States of America; 3 Department of Biomedical Engineering, Washington University in St. Louis, St. Louis, Missouri, United States of America; 4 Department of Developmental Biology, Center of Regenerative Medicine, Washington University School of Medicine at Barnes Jewish Hospital, St. Louis, Missouri, United States of America; University of Pécs Medical School, HUNGARY

## Abstract

**Objectives:**

(i) To provide baseline knowledge of gene expression in macroscopically normal articular cartilage, (ii) to test the hypothesis that age, body-mass-index (BMI), and sex are associated with cartilage RNA transcriptome, and (iii) to predict individuals at potential risk for developing “pre-osteoarthritis” (OA) based on screening of genetic risk-alleles associated with OA and gene transcripts differentially expressed between normal and OA cartilage.

**Design:**

Healthy-appearing cartilage was obtained from the medial femoral notch of 12 knees with a meniscus tear undergoing arthroscopic partial meniscectomy. Cartilage had no radiographic, magnetic-resonance-imaging or arthroscopic evidence for degeneration. RNA was subjected to Affymetrix microarrays followed by validation of selected transcripts by microfluidic digital polymerase-chain-reaction. The underlying biological processes were explored computationally. Transcriptome-wide gene expression was probed for association with known OA genetic risk-alleles assembled from published literature and for comparison with gene transcripts differentially expressed between healthy and OA cartilage from other studies.

**Results:**

We generated a list of 27,641 gene transcripts in healthy cartilage. Several gene transcripts representing numerous biological processes were correlated with age and BMI and differentially expressed by sex. Based on disease-specific Ingenuity Pathways Analysis, gene transcripts associated with aging were enriched for bone/cartilage disease while the gene expression profile associated with BMI was enriched for growth-plate calcification and OA. When segregated by genetic risk-alleles, two clusters of study patients emerged, one cluster containing transcripts predicted by risk studies. When segregated by OA-associated gene transcripts, three clusters of study patients emerged, one of which is remarkably similar to gene expression pattern in OA.

**Conclusions:**

Our study provides a list of gene transcripts in healthy-appearing cartilage. Preliminary analysis into groupings based on OA risk-alleles and OA-associated gene transcripts reveals a subset of patients expressing OA transcripts. Prospective studies in larger cohorts are needed to assess whether these patterns are predictive for OA.

## Introduction

Articular cartilage is a specialized connective tissue of diarthrodial joints. Several lines of evidence suggest that age [1], body-mass-index (BMI) [2, 3], genetics [4, 5], and sex [3, 6] affect the biology of cartilage leading to its degeneration and loss. Degeneration of cartilage is the hallmark end-stage finding in osteoarthritis (OA), causing joint failure and often resulting in total joint replacement. There is a higher prevalence of OA in older and obese individuals, as well as in females [7].

Studying healthy cartilage from humans is challenging but not impossible. Cadaver knees are a significant source of tissue but often lack adequate information regarding the presence or absence of concomitant joint injuries and OA. Cartilage from patients undergoing knee amputation due to chondrosarcoma is another source but generally comes from a younger population and may have been subjected to chemotherapy drugs or radiation [8]. Non-fibrillated cartilage from total knee replacement has been used, but this cartilage is exposed to an OA environment [9, 10]. Joint replacement for symptomatic osteonecrosis may be another source [11] but the cartilage is affected by the diseased state of the underlying bone. Several other studies have attempted to compare the gene expression differences between healthy and degenerated cartilage isolated from knees with OA [12–14] or have used cartilage obtained from both hip and knee joints [15, 16]. In the current study, we obtained healthy and seemingly normal cartilage from knees with a meniscus tear but with no evidence for OA, chondrosis, or inflammation (as assessed by radiographs, magnetic-resonance-imaging i.e. MRI and arthroscopy), and examined the RNA expression profile.

Aging elevates the risk of cartilage degeneration by suppression of proteoglycan synthesis, augmented collagen cross-linking and loss of tensile strength [17], and increase in inflammation often resulting in OA [18]. In addition, age-related loss of chondrocyte function may result from progressive cell senescence, beta galactosidase overexpression, and erosion of telomere length [19, 20]. The synthetic activity of chondrocytes declines with age through modulation of insulin like growth factor 1 [21, 22]. In murine joint tissues, age affects the basal pattern of gene expression as determined by a decline in extracellular matrix genes and an elevation of immune response genes [23]. Along these lines, a recent equine study provided important insights into the transcriptional networks of aging cartilage showing that age dysregulates matrix, anabolic and catabolic factors [24].

Obesity is an important risk factor for OA development and progression and is associated with alterations in joint biomechanics and inflammatory environment [25, 26]. Adipocytokines contribute to the low-grade inflammatory state of obese patients and may promote cartilage degeneration [27, 28]. Furthermore, stimulation of chondrocytes with leptin, adiponectin, or resistin, alone or in combination with other (pro)inflammatory cytokines, fuels the expression of cytokines, matrix metalloproteinases, and nitric oxide synthase [29–31]. Studies have shown that females have less knee cartilage than males [32, 33] thus potentially explaining why females have 4 to 10 times higher risk of OA [34]. This higher susceptibility may also be associated with other factors such as sex-based hormonal differences [32, 33].

While aging, obesity and female sex are associated with changes in cartilage metabolism and OA [7, 34, 35], there are little, if any, details that define their association with gene expression in healthy cartilage. Human cartilage specimens from patients undergoing partial meniscectomy provide macroscopically intact tissue that may reflect early effects of meniscus injury or even a propensity to injury in the otherwise healthy tissue. We hypothesize that the association of patient age, BMI, and sex with the cartilage gene expression profile may identify early evidence for cartilage degeneration and inflammation.

Lastly, it has been reported that approximately 50% of patients with meniscus injury will go on to develop OA within 10–15 years [36]. Therefore, we attempted to determine if we could detect a molecular indication for “pre-OA” phenotype in our study patients. To achieve this, we screened a set of genetic risk-alleles from genome-wide association scans (GWAS) and a set of gene transcripts differentially expressed between healthy and OA cartilage from other studies.

## Materials and Methods

### Study design

All procedures were approved by the Institutional Review Board (Human Research Protection Office) of Washington University School of Medicine (St. Louis). Patients diagnosed with a symptomatic tear in the posterior horn of the medial meniscus and undergoing arthroscopic surgery furnished a written informed consent as approved by the Human Research Protection Office of study institution. All patients in the study had radiographs and an MRI of the knee prior to surgery. To be considered for the study, the patients could not have any evidence for OA, chondrosis, bone marrow lesions/edema, and ligamentous injury greater than Grade I medial collateral ligament strain. Patients of any age, BMI and from both sexes were recruited.

### Characteristics of study patients

The patient cohort was relatively homogeneous (Table 1). The median age of study patients was 51.5 years (range = 31–65 years; mean age = 49.2 years) and mean BMI was 26.9 kg/m^2^ ± 3.9 (mean ± standard deviation), with 5 females (mean age = 50.6 years; mean BMI = 24.3 kg/m^2^) and 7 males (mean age = 48.1 years; mean BMI = 28.7 kg/m^2^). None of the patients were smokers or had diabetes.

**Table 1:** Characteristics of study participants and quality of RNA samples.

Patient #	Age (years)	BMI (kg/m^2^)	Sex	Time from injury (months)	Smoking	Diabetes	RIN
P4-001	37	21.9	Female	26	No	No	7.4
P4-002	45	28.8	Male	20	No	No	6.7
P4-003	62	27.8	Female	9	No	No	7.1
P4-004	58	20.5	Female	5	No	No	6.8
P4-005	31	24.3	Female	8	No	No	8.1
P4-007	53	26.6	Male	2	No	No	7.0
P4-008	50	28.5	Male	21	No	No	6.9
P4-009	65	27.1	Female	12	No	No	7.0
P4-010	53	32.0	Male	2	No	No	6.7
P4-011	43	34.4	Male	2.5	No	No	6.5
P4-012	40	24.5	Male	18	No	No	6.8
P4-013	53	26.3	Male	2	No	No	6.7
***Mean***	***49*.*2***	***26*.*9***	***5 females***	***10*.*6***	-	-	***-***
			***7 males***				

BMI = Body mass index; RIN = RNA integrity number

### Sample collection

A fragment (160–235 mg) of healthy cartilage was taken from the non-weight bearing site of the medial intercondylar notch with the use of a sharp curette, in a technique similar to a Carticel^™^ biopsy [37]. The tissue fragments were immersed in RNAlater stabilization solution (Applied Biosystems, Foster City, CA) and delivered to the laboratory for processing and analysis.

### RNA isolation

Tissues were refrigerated for 24 hours followed by storage at 80°C until RNA isolation [38]. The tissues were pulverized using a Mikro-dismembrator (B. Braun, Biotech International, Melsungen, Germany) at 2000 rpm for 2 minutes under cryogenic conditions. The pulverized cartilage was dissolved in 1 mL of TRIzol reagent (Invitrogen, Carlsbad, CA). After addition of 200 μL of chloroform, the sample was mixed vigorously and the contents were transferred to phase lock gel tube and centrifuged for 15 minutes at 4°C. The clear aqueous layer was transferred by decanting of pipetting and 1 volume of 70% RNase-free ethanol was added to precipitate RNA. RNA was collected using RNeasy spin columns (Qiagen Inc. Valencia, CA). The quality of the RNA was assessed by Agilent 2100 Bioanalyzer (Agilent Technologies, Santa Clara CA). Average RNA yield was 7–8 ng/mg of tissue.

### Affymetrix microarrays

Five microgram of biotinylated cDNA (from 50 ng total RNA) was prepared through NuGEN Ovation Pico WTA Amplification System. This system provides a fast and simple method for preparing amplified cDNA for gene expression analysis. Amplification is initiated at the 3’ end as well as randomly throughout the transcriptome in the sample, making the Ovation Pico WTA system ideal for amplification of partially degraded and compromised RNA samples from tissue. The amplified product of the Ovation Pico WTA system is optimized for gene expression analysis applications on microarrays. Following fragmentation with NuGEN Encore Biotin Module, cDNA was hybridized into the GeneChip Hybridization Oven 640 for 18 hours at 45°C on Affymetrix Gene 2.0 ST Array (Affymetrix Inc. Santa Clara, CA). The microarrays were washed and stained in the Affymetrix Fluidics Station 450 and scanned using the Affymetrix GeneChip 7G 3000 Scanner. The microarray data were analyzed with the Affymetrix GeneChip Command Console and processed with the use of Command Console (Affymetrix, Inc. Santa Clara, CA) and the raw (.CEL) files generated were analyzed using Expression Console software with Affymetrix default RMA Gene analysis settings (Affymetrix, Inc. Santa Clara, CA). Probe summarization (Robust Multichip Analysis, RMA), quality control analysis, and probe annotation were performed per recommended guidelines (Expression Console Software, Affymetrix, Inc. Santa Clara, CA). Data were quantile normalized and log_2_ transformed before downstream analysis by Partek Genomic Suite v6.4 (Partek Inc. Chesterfield, MO). Statistical significant raw P values were adjusted for multiple comparisons. The effect of all factors (age, BMI, and sex) was assessed at a significance level set at unadjusted P value of <0.05. Pearson correlation coefficients (r values) were calculated using ANCOVA for age and BMI while fold change differences were calculated between males and females. Age and BMI were used a continuous variables while sex was treated as a categorical variable. The distribution of males and females across age was similar. Mean age for female participants was 50.6 years (range = 31–65 years) while that of male participants was 48.1 years (range = 40–53 years) and there was no significant difference between the mean age of females and males (P = 0.702). However, BMI of female participants (24.3 kg/m^2^) was significantly (P = 0.048) lower than that of male participants (28.7 kg/m^2^). Therefore, to circumvent the influence of any one variable on the differentially correlated or differentially expressed gene transcripts, we added all three variables (age, BMI, sex) in the model.

### Baseline gene expression values

We created a list of all detectable gene transcripts in order of their relative intensity values to provide baseline gene expression abundance in healthy articular cartilage.

### Gene ontology analysis

To facilitate the interpretation of differentially expressed transcripts, we used gene ontology analysis. A list of differentially expressed gene transcripts was uploaded on the GeneGo MetaCore web portal v6.22 (https://portal.genego.com). Then “one click” gene ontology was applied to obtain highly enriched and statistically significant biological processes and molecular functions. For the sake of simplicity we focused only on the biological processes, information on molecular functions can be found by plugging the gene list into GeneGo MetaCore web portal and by clicking “gene ontology molecular functions” tab.

### Ingenuity pathway analysis (IPA)

IPA (Ingenuity Systems, Redwood City, CA) software program was used to determine the functional pathways and human disease-associated pathways represented by the identified genes, which were associated with age (S1 Table), BMI (S2 Table), and sex (S3 Table). The IPA software contains a knowledge database of biological interactions among genes and proteins. IPA software was used to calculate the probability of a relationship between each canonical pathway, upstream pathway and the identified genes. The genes associated with age, BMI and sex were input into IPA to perform Core Analysis. Only those gene transcripts were used as input that exhibited a significant correlation with age and BMI (P < 0.05) and a significant differential expression between males and females (fold change ≥ 1.5, P < 0.05). The connective tissue related disease terms and biological functions were selected based on a P value cutoff at 0.05.

### Comparison with OA genes identified by GWAS and by gene transcripts differentially expressed between healthy and OA cartilage

A list of susceptibility alleles was assembled from the literature [39] and expression levels were probed in our dataset. We collected the genes around OA-associated genetic risk-alleles that were reported in published GWAS studies [39] and then computed the z-scores (representing the expression difference of each gene to averaged expression of all samples) of these genes to investigate the expression pattern of OA associated genes in healthy cartilage after surgery. We also used a previously published list of gene transcripts generated by microarray analysis of healthy and OA cartilage [40] to evaluate our patient population. The differentially expressed gene transcripts (>15-fold) between 8 healthy controls and 5 OA cartilage tissues were taken as reported [40]. Cluster analysis of our cohort with the above gene transcripts was performed with the use of Limma in R environment to identify patients with a molecular profile suggestive of “pre-OA”.

### Microfluidic digital polymerase-chain-reaction (PCR) to confirm microarray data

To confirm microarray data, we measured the expression pattern of 9 genes via microfluidic digital PCR 96.96 Dynamic Arrays (Fluidigm Corp. San Francisco, CA) using RNA from all 12 study samples that were used for microarrays. The selection of these genes was mainly based on their differential expression by age, BMI and sex in this study. The probe sequences can be found by locating the gene ID provided for each gene below by visiting http://www.lifetechnologies.com and then by selecting “best coverage” and “human” under “narrow your results” tab: *SNAI2* (Hs00950344_m1), *PSMD5 AS1* (Hs00404560_m1), *CYP7A1* (Hs00167982_m1), *GDF11* (Hs00195156_m1), *S100A8* (Hs00374264_g1), *SFRP2* (Hs00293258_m1), *ACAN* (Hs00153936_m1), *CHAD* (Hs00154382_m1), and *COL2A1* (Hs00264051_m1). These genes were chosen for three reasons: (1) those that were positively correlated with age (*SNA12*) and BMI (*CYP7A1*) and expressed at higher levels in females (*S100A8*,); (2) those that were negatively correlated with age (*PSMD5AS1*) and BMI (*GDF11*) and expressed at lower levels in females (*SFRP2*) and (3) those that were neither correlated with age or BMI nor were differentially expressed by sex (*ACAN*, *CHAD*, *COL2A1*).

The cDNAs were subjected to specific target amplification using TaqMan PreAmp Mastermix (Applied Biosystems, Foster City, CA) and High Capacity cDNA Reverse Transcription Kit (Life Technologies, Carlsbad, CA). The sample and assay mixtures were prepared using manufacturers’ guidelines and were loaded on the NanoFlex 4 Integrated Fluidic Circuit Controller for equal distribution followed by insertion into the BioMark^™^ Reverse Transcription PCR System. The cycling program consisted of 10 minutes at 95°C followed by 14 cycles of 95°C for 15 seconds and then 60°C for 60 seconds. Data were analyzed using Fluidigm Reverse Transcription PCR Analysis software. Analysis was performed using 2^−ΔCt^ approach with TATA box binding protein (*TBP;* Hs00427620_m1) as internal reference gene. The data were analyzed by SPSS software (SPSS Inc. Chicago, IL). Pearson correlation coefficients were calculated for age and BMI. The differences between the means were measured for males and females using student’s t-test and reported as mean ± standard error of the mean.

## Results

### Quantitative transcriptome differences by age, BMI, and sex

**Fig 1:**
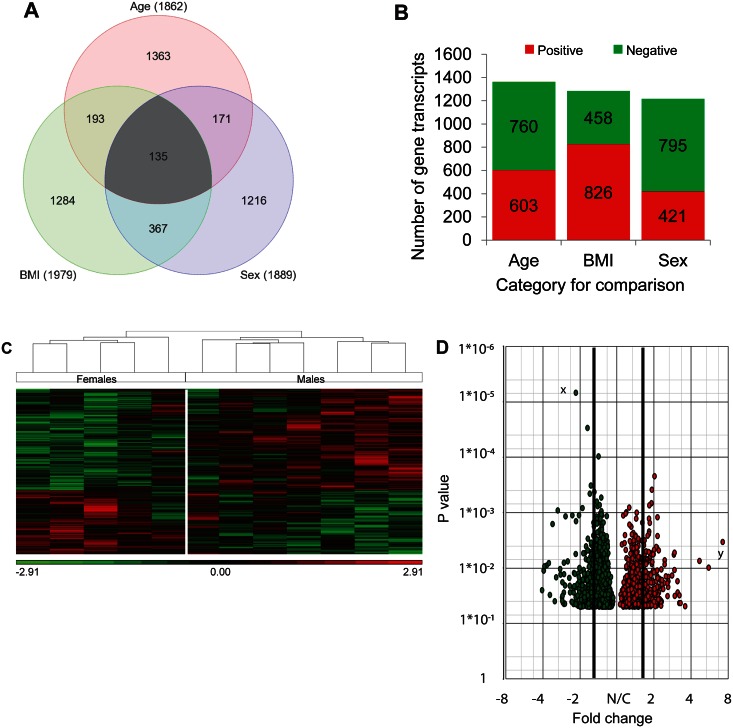
Pictorial representation of microarray data. Microarray analysis of human healthy articular cartilage was conducted to assess the effect of age, BMI, and sex. **A.** Venn diagrams represent the number of differentially expressed genes in each comparison. Values in parentheses are the numbers of differentially expressed genes. Values in overlapping areas are the numbers of genes common to any two comparisons. **B.** Numbers of differentially expressed gene transcripts across all three factors are shown. Number of gene transcripts shown for age and BMI indicate positively (red) and negatively (green) correlated gene transcripts while for sex they represent up-regulated (red) and down-regulated (green) gene transcripts in females versus males. **C.** Hierarchical clustering map, representing the transcripts that were significantly (P<0.05) differentially expressed between the two sexes is shown. Each vertical row depicts a single sample, and each horizontal line stands for a single gene transcript. Green color indicates down-regulated gene transcripts while red color indicates up-regulated gene transcripts. **D.** The differentially expressed gene transcripts at all fold changes are shown in the form of volcano plots to show the trend of expression by P value (y axes) and fold change (x axes). Gene transcripts outside the thick black lines are those that have ≥1.5 fold expression x = gene transcripts with lowest P value (statistically highly significant) y = gene transcripts with highest fold change (biologically highly significant), green circles = gene transcripts down-regulated in females, red circles = gene transcripts up-regulated in females, N/C = no change.

Out of 53,617 transcripts available on the microarray chip, the number of significantly differentially expressed transcripts was 1979 (3.69%) for BMI, 1889 (3.52%) for sex and 1862 (3.47%) for age. Interestingly, the number of transcripts for each category was not substantially different from each other. Among these, 1363 transcripts were exclusively associated with age, 1284 with BMI and 1216 with sex. BMI and sex had overall more common transcripts (367 transcripts) than BMI and age (193 transcripts) or age and sex (171 transcripts). 135 transcripts were common to age, BMI and sex (Fig 1A). The number of transcripts positively or negatively correlated with age and BMI and number of gene transcripts higher or lower in expression by sex are shown in Fig 1B. Hierarchical clustering by sex showed a clear clustering of our specimens into two groups, females, and males, thus confirming that real differences in transcripts exist between females and males (Fig 1C). We also created volcano plots to depict overview of individual genes differentially expressed by sex (Fig 1D) and to show their distribution by fold change and P values simultaneously.

### Effect of age on transcriptomic signatures

**Fig 2:**
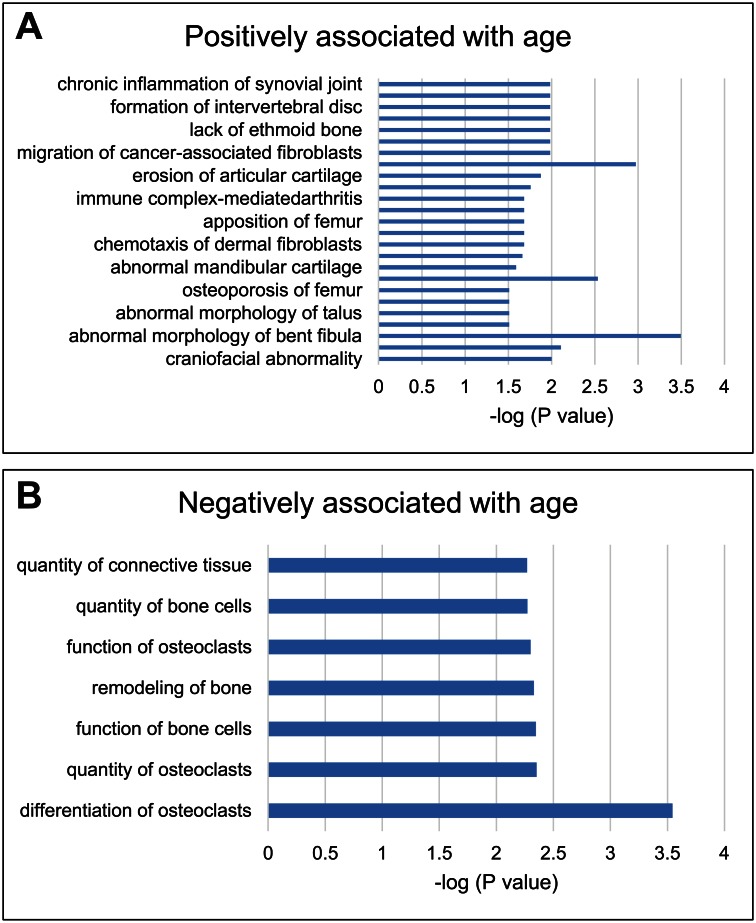
Biological processes associated with age. Based on statistical significance i.e. -log (P value) as determined by IPA analysis, the biological processes positively (A) and negatively (B) associated with age are shown.

After removal of duplicate, non-annotated, and uncharacterized transcripts, 592 gene transcripts were differentially expressed exclusively by age. Among these, 267 gene transcripts were positively correlated with age and 325 gene transcripts were negatively correlated with age (S1 Table). A list of top 20 gene transcripts correlated (positively or negatively) with age is shown in Table 2. These biological processes were correlated positively with age: regulation of intracellular signal transduction, fat cell differentiation, cell-cell communication, and necroptotic process and these were negatively correlated with age: immune response and leukocyte activation (Table 3). IPA analysis further showed that the genes positively associated with age were correlated to distinct cartilage and bone abnormalities (Fig 2A), and genes negatively associated with age were correlated to normal bone functions, including quantity of connective tissue and differentiation of osteoclasts (Fig 2B).

**Table 2:** Selected list of gene transcripts correlated with age and BMI*.

*Symbol*	*Gene name*		*P value*	*R*	*Symbol*	*Gene name*	*P value*	*R*
*Gene transcripts positively correlated with age*					*Gene transcripts negatively correlated with age*			
*TSC2*	tuberous sclerosis 2		0.004	0.81	*PFKFB2*	6 phosphofructo 2 kinase/fructose 2,6 biphosphatase 2	0.001	0.87
*SNAI2*	snail family zinc finger 2		0.004	0.81	*PSMD5 AS1*	**proteasome (prosome, macropain) 26S subunit, non- ATPase, 5 –antisense RNA 1**	0.001	0.87
*SLC35E1*	solute carrier family 35, member E1		0.003	0.81	*CD14*	CD14 molecule	0.001	0.84
*DCUN1D2 AS2*	DCN1, Defective In Cullin Neddylation 1 –antisense RNA 2		0.004	0.80	*FCGR2B*	Fc fragment of IgG, low affinity IIb, receptor	0.002	0.82
*PREX2*	phosphatidylinositol 3,4,5 trisphosphate dependent Rac exchange factor 2		0.005	0.80	*TAGAP*	T cell activation RhoGTPase activating protein	0.003	0.82
*IFITM2*	interferon induced transmembrane protein 2		0.004	0.79	*OR2M3*	olfactory receptor, family 2, subfamily M, member 3	0.002	0.82
*DOT1L*	DOT1 like histone H3K79 methyltransferase		0.004	0.79	*SLC8A1 AS1*	solute carrier 8 family A1 –antisense RNA 1	0.003	0.82
*STXBP4*	syntaxin binding protein 4		0.002	0.79	*DIRC3*	disrupted in renal carcinoma 3	0.003	0.81
*MOV10*	Moloney leukemia virus 10, homolog		0.005	0.79	*ATP8B4*	ATPase, class I, type 8B, member 4	0.003	0.81
*CNNM3*	cyclin M3		0.006	0.78	*RASSF2*	Ras association (RalGDS/AF 6) domain family member 2	0.004	0.80
*CDK2*	cyclin dependent kinase 2		0.005	0.78	*MMP24 AS1*	matrix metallopeptidase 24 –antisense RNA 1	0.000	0.80
*RAD51 AS1*	RAD51 antisense RNA 1		0.007	0.78	*FGD2*	FYVE, RhoGEF and PH domain containing 2	0.005	0.79
*ZNF546*	zinc finger protein 546		0.005	0.77	*HNF1A AS1*	**Hepatocyte nuclear factor 1 alpha—antisense RNA 1**	0.006	0.78
*TRIB2*	tribbles pseudokinase 2		0.005	0.77	*TCEAL3*	transcription elongation factor A (SII) like 3	0.007	
*SLC25A37*	solute carrier family 25 (mitochondrial iron transporter), member 37		0.007	0.77	*CYTIP*	cytohesin 1 interacting protein	0.007	0.77
*SLC39A1*	solute carrier family 39 (zinc transporter), member 1		0.005	0.77	*NCF1B*	neutrophil cytosolic factor 1B	0.007	0.77
*XAB2*	XPA binding protein 2		0.007	0.76	*SLC5A8*	solute carrier family 5 (sodium/monocarboxylate cotransporter), member 8	0.007	0.77
*TAF6L*	TAF6 like RNA polymerase II, p300/CBP associated factor (PCAF) associated factor		0.008	0.76	*RAB11FIP1*	RAB11 family interacting protein 1	0.007	0.77
*GMCL1*	germ cell less, spermatogenesis associated 1		0.006	0.75	POTEE	POTE ankyrin domain family, member E	0.007	0.76
*RING1*	ring finger protein 1		0.009	0.75	*PPEF1 AS1*	protein phosphatase, EF hand calcium binding domain 1 antisense RNA 1	0.011	0.76
*Gene transcripts positively correlated with BMI*					*Gene transcripts negatively correlated with BMI*			
*CYP7A1*		cytochrome P450, family 7, subfamily A, polypeptide 1	0.001	0.85	*ERCC2*	excision repair cross complementation group 2	0.002	0.80
*TMEM235*		transmembrane protein 235	0.001	0.82	*TRIM49*	tripartite motif containing 49	0.004	0.80
*CCDC79*		coiled coil domain containing 79	0.005	0.79	*GDF11*	growth differentiation factor 11	0.005	0.79
*SLC26A5*		solute carrier family 26 (anion exchanger), member 5	0.005	0.78	*ZFYVE28*	zinc finger, FYVE domain containing 28	0.006	0.78
*ZNF852*		zinc finger protein 852	0.006	0.78	*AANAT*	aralkylamine N acetyltransferase	0.006	0.78
*DDR1*		discoidin domain receptor tyrosine kinase 1	0.004	0.77	*LAMTOR2*	late endosomal/lysosomal adaptor, MAPK and MTOR activator 2	0.004	0.78
*LRRC2*		leucine rich repeat containing 2	0.007	0.77	*BAAT*	bile acid CoA: amino acid N acyltransferase	0.007	0.78
*LYPD6*		LY6/PLAUR domain containing 6	0.008	0.77	*DIEXF*	digestive organ expansion factor homolog	0.003	0.77
*FFAR1*		free fatty acid receptor 1	0.008	0.76	*FGD1*	FYVE, RhoGEF and PH domain containing 1	0.008	0.77
*IGLV4 69*		immunoglobulin lambda variable 4 *69*	0.008	0.76	*SLC35B1*	solute carrier family 35, member B1	0.010	0.76
*CDHR3*		cadherin-related family member 3	0.010	0.76	*TMEM243*	transmembrane protein 243	0.002	0.76
*RGS21*		regulator of G protein signaling 21	0.011	0.75	*HEXIM1*	hexamethylene bis acetamide inducible 1	0.010	0.76
*ATXN3L*		ataxin 3 like	0.008	0.75	*MICB*	MHC class I polypeptide-related sequence B	0.010	0.75
*RASSF10*		Ras association (RalGDS/AF 6) domain family (N terminal) member 10	0.012	0.75	*HIST1H2AJ*	histone cluster 1, H2aj	0.010	0.75
*SORBS2*		sorbin and SH3 domain containing 2	0.013	0.74	*CCDC28B*	coiled coil domain containing 28B	0.015	0.73
*HAO2*		hydroxyacid oxidase 2	0.007	0.74	*ZNF846*	zinc finger protein 846	0.008	0.73
*RIC8A*		RIC8 guanine nucleotide exchange factor A	0.014	0.73	*PGP*	phosphoglycolate phosphatase	0.003	0.73
*SLC6A15*		solute carrier family 6 (neutral amino acid transporter), member 15	0.008	0.73	*RHOA IT1*	*RHOA* intronic transcript 1	0.004	0.73
*AADACL4*		arylacetamide deacetylase like 4	0.011	0.73	*FAM174B*	family with sequence similarity 174	0.016	0.72
*ANK1*		ankyrin 1	0.008	0.73	*NXPH4*	neurexophilin 4	0.014	0.72

*Top 20 gene transcripts highly correlated with age and BMI are shown.

BMI = body mass index, r = correlation coefficient

**Table 3:** Biological processes represented by differentially expressed gene transcripts by age, BMI, and sex.

*Process*	*P value*	*FDR*	*Process*	*P value*	*FDR*
***Biological processes positively correlated with age***			***Biological processes negatively correlated with age***		
regulation of intracellular signal transduction	9.504E-10	3.476E-06	immune response	2.558E-15	1.051E-11
regulation of signal transduction	1.095E-08	2.002E-05	innate immune response	9.620E-13	1.977E-09
regulation of fat cell differentiation	5.523E-08	6.733E-05	defense response	4.465E-12	6.117E-09
regulation of cell communication	9.753E-08	8.243E-05	myeloid leukocyte activation	3.507E-11	3.603E-08
necroptotic process	1.127E-07	8.243E-05	immune system process	6.500E-11	5.343E-08
regulation of signaling	2.080E-07	1.268E-04	leukocyte activation	9.098E-11	6.232E-08
regulation of cellular protein metabolic process	2.838E-07	1.458E-04	positive regulation of cell activation	1.980E-10	1.163E-07
positive regulation of neurotransmitter transport	3.190E-07	1.458E-04	regulation of immune system process	2.873E-10	1.476E-07
regulation of establishment of protein localization	5.196E-07	2.111E-04	regulation of immune response	8.050E-10	3.676E-07
positive regulation of muscle contraction	6.796E-07	2.307E-04	response to cytokine	1.499E-09	6.160E-07
***Biological processes positively correlated with BMI***			***Biological processes negatively correlated with BMI***		
positive regulation of memory T cell activation	1.952E-11	1.950E-08	response to mineralocorticoid	1.252E-06	3.061E-03
regulation of multicellular organismal process	6.924E-10	2.767E-07	response to toxic substance	2.288E-06	3.061E-03
positive regulation of multicellular organismal process	1.451E-09	5.271E-07	adenylate cyclase modulating G protein receptor signaling	3.273E-06	3.061E-03
tolerance induction	3.325E-09	1.022E-06	adenylate cyclase inhibiting serotonin receptor signaling	4.652E-06	3.061E-03
regulation of system process	6.153E-09	1.639E-06	cellular response to estrogen stimulus	5.894E-06	3.061E-03
regulation of response to external stimulus	7.850E-09	1.960E-06	cellular response to abiotic stimulus	7.710E-06	3.061E-03
positive regulation of interferon gamma production	1.448E-08	3.095E-06	negative regulation of synaptic transmission, GABAergic	7.947E-06	3.061E-03
G protein coupled receptor signaling pathway, coupled to cyclic nucleotide second messenger	1.674E-08	3.344E-06	rhodopsin mediated signaling pathway	9.845E-06	3.061E-03
cell-cell signaling	2.144E-08	4.079E-06	regulation of endocrine process	9.845E-06	3.061E-03
regulation of blood pressure	2.266E-08	4.116E-06	G protein coupled receptor internalization	1.016E-05	3.061E-03
***Biological processes elevated in females***			***Biological processes repressed in females***		
detection of chemical stimulus involved in sensory perception of taste	4.045E-05	2.702E-02	neuron differentiation	6.716E—11	1.608E-07
L-ascorbic acid transport	5.015E-05	2.702E-02	tissue development	1.064E-10	1.608E-07
peptidyl cysteine oxidation	5.015E-05	2.702E-02	cell development	1.096E-10	1.608E-07
aminoglycan metabolic process	8.458E-05	2.702E-02	cell differentiation	1.534E-10	1.688E-07
chaperone mediated protein folding	9.212E-05	2.702E-02	cellular developmental process	5.230E-10	4.605E-07
negative regulation of cysteine type endopeptidase activity involved in apoptotic process	1.050E-04	2.702E-02	heart development	1.716E-09	1.098E-06
regulation of cysteine type endopeptidase activity involved in apoptotic process	1.235E-04	2.702E-02	system development	1.769E-09	1.098E-06
glycosaminoglycan biosynthetic process	1.426E-04	2.702E-02	heart morphogenesis	2.193E-09	1.098E-06
mitotic cell cycle checkpoint	1.452E-04	2.702E-02	anatomical structure morphogenesis	2.244E-09	1.098E-06
detection of chemical stimulus involved in sensory perception of bitter taste	2.664E-04	3.950E-02	circulatory system development	3.306E-09	1.323E-06

BMI = body mass index, FDR = false discovery rate

### Effect of BMI on transcriptomic signatures

**Fig 3:**
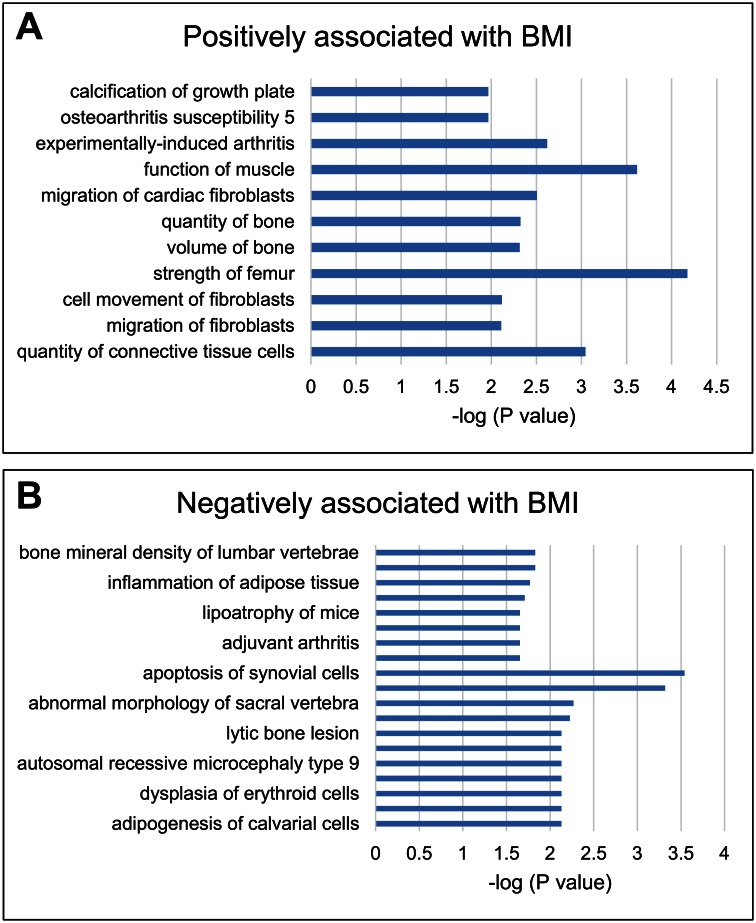
Biological processes associated with BMI. Based on statistical significance i.e. -log (P value) as determined by IPA analysis, the biological processes positively (A) and negatively (B) associated with BMI are shown.

After removal of duplicate, non-annotated, and uncharacterized transcripts, 509 gene transcripts were differentially expressed exclusively by BMI. Among these, 317 gene transcripts were positively correlated with BMI and 192 gene transcripts were negatively correlated with BMI (S2 Table). A list of top 20 gene transcripts correlated (positively or negatively) with BMI is shown in Table 2. The gene transcripts positively correlated with BMI were enriched for these biological processes: regulation of memory T-cell activation and regulation of tolerance induction to nonself antigen. The gene transcripts negatively correlated with BMI were enriched for these biological processes: response to mineralocorticoid (steroid hormones) and response to toxic substances (Table 3). IPA analysis showed that the genes positively associated with BMI were correlated to bone strength and volume, and enriched for OA (Fig 3A) while those negatively associated with BMI were correlated with adipogenesis, apoptosis and bone mineral density (Fig 3B). These associations are evidence that elevated BMI is a risk factor for OA.

### Effect of sex on transcriptomic signatures

**Fig 4:**
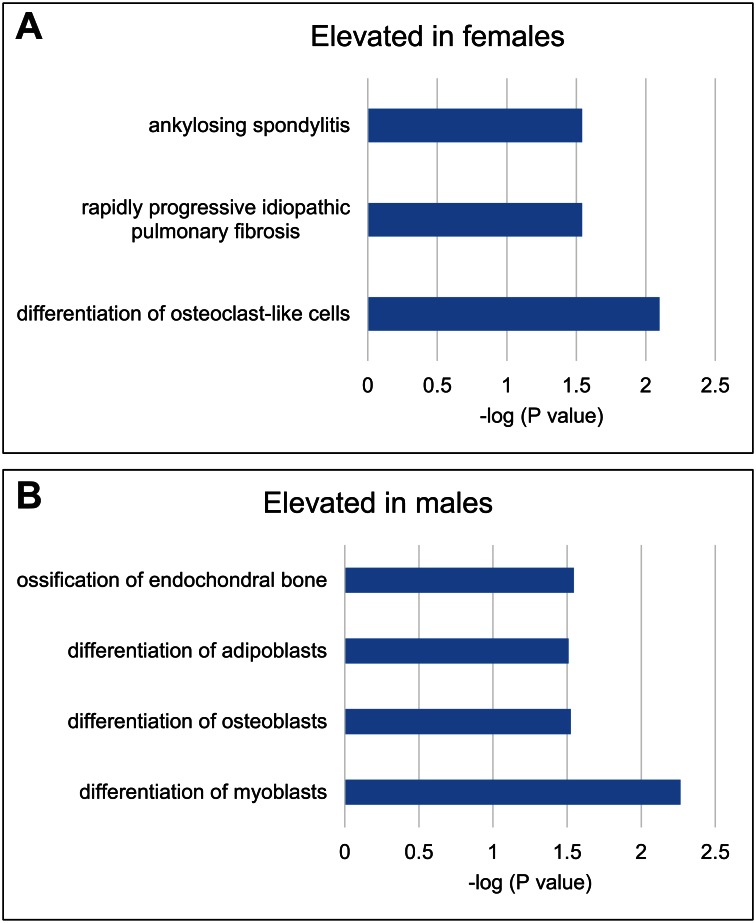
Biological processes associated with sex. Based on statistical significance i.e. -log (P value) as determined by IPA analysis, the biological processes elevated in females (A) and males (B) are shown.

After removal of duplicate, non-annotated, and uncharacterized transcripts, 484 transcripts were differentially expressed by sex. To identify transcripts with the highest significant difference, the analysis was restricted to transcripts showing fold change of ≥1.5 and P<0.05. By these criteria, the number of differentially regulated transcripts was reduced to 13% (63 of 484 transcripts). More transcripts were lower in expression in females (317 transcripts) than those that were higher in expression (167 transcripts). S3 Table lists all gene transcripts differentially regulated by sex at ≥1.5 fold while Table 4 shows top 10 gene transcripts only. Gene transcripts elevated in females were enriched for these biological processes: detection of chemical stimulus involved sensory perception of taste, L-ascorbic acid transport, peptidyl cysteine oxidation, aminoglycan metabolic process, and chaperone-mediated protein folding (Table 3). These biological processes were enriched by gene transcripts repressed in females: cell and tissue development and cell differentiation (Table 3). IPA analysis showed that the genes highly expressed in females were correlated with differentiation of osteoclast-like cells and ankylosing spondylitis (Fig 4A). Likewise, genes highly expressed in males were correlated with differentiation of myoblasts, osteoblasts, and adenoblasts and endochondral bone ossification (Fig 4B).

**Table 4:** Selected list of gene transcripts differentially expressed by sex*.

*Gene symbol*	*Gene name*	*P value*	*LS Mean (Females)*	*LS Mean (Males)*	*Ratio*	*Fold change*
***Gene transcripts up-regulated in females***						
*S100A8*	S100 calcium binding protein A8	0.032	157.62	53.40	2.95	2.95
*CCDC144A*	coiled coil domain containing 144A	0.024	75.20	31.99	2.35	2.35
*CSN1S1*	casein alpha s1	0.048	21.14	9.26	2.28	2.28
*MEDAG*	mesenteric estrogen dependent adipogenesis	0.039	160.68	73.58	2.18	2.18
*TREML5P*	triggering receptor expressed on myeloid cells like 5	0.005	20.45	10.22	2.00	2.00
*ITGBL1*	integrin, beta like 1	0.049	562.50	298.70	1.88	1.88
*CD68*	*CD68* molecule	0.038	899.71	478.62	1.88	1.88
*CSNK2A3*	casein kinase 2, alpha 3 polypeptide	0.025	41.67	22.30	1.87	1.87
*SPDYE5*	speedy/RINGO cell cycle regulator family member E5	0.045	69.83	37.60	1.86	1.86
*UST*	Uronyl 2 sulfotransferase	0.040	6.24	5.47	1.71	1.71
***Gene transcripts down-regulated in females***						
*SLPI*	secretory leukocyte peptidase inhibitor	0.013	39.84	114.70	0.35	2.88
*SFRP2*	secreted frizzled-related protein 2	0.041	29.77	69.61	0.43	2.34
*CEACAM5*	carcinoembryonic antigen-related cell adhesion molecule 5	0.028	7.34	13.46	0.55	1.83
*HCG22*	HLA complex group 22	0.007	14.89	25.59	0.58	1.72
*MTRNR2L4*	MT RNR2 like 4	0.050	6.42	10.70	0.60	1.67
*PODXL*	podocalyxin like	0.032	43.40	70.96	0.61	1.64
*THRB AS1*	THRB antisense RNA 1	0.039	11.11	18.17	0.61	1.63
*CYP2C8*	cytochrome P450, family 2, subfamily C, polypeptide 8	0.037	7.83	12.75	0.61	1.63
*DPPA2*	developmental pluripotency associated 2	0.003	4.67	7.59	0.61	1.63
*OR5D14*	olfactory receptor, family 5, subfamily D, member 14	0.024	16.31	26.23	0.62	1.61

*top 10 gene transcripts differentially expressed between males and females are shown.

LS = least squares

### Gene transcripts common to age, BMI, and sex

Several gene transcripts were common between any two comparisons of patient factors. For instance, there were 193 transcripts common to age and BMI, 367 to BMI and sex and 171 to age and sex. There were 135 gene transcripts common to all three factors (age, BMI, and sex). After removal of duplicate, non-annotated, and uncharacterized gene transcripts, 55 gene transcripts were common to all three categories (Table 5).

**Table 5:** Gene transcripts common to age, BMI and sex*.

*Gene Symbol*	*P value (age)*	*r (age)*	*P value (BMI)*	*r (BMI)*	*P value (sex)*	*Fold change (sex)*	*Description (sex)*
*LINC00173*	0.000	0.86	0.008	-0.48	0.007	-1.39	Down-regulated in females
*LY6E*	0.001	0.85	0.036	-0.39	0.017	-1.23	Down-regulated in females
*MYLK*	0.002	0.80	0.032	-0.44	0.014	-1.21	Down-regulated in females
*ETS1*	0.005	0.74	0.045	-0.46	0.025	-1.44	Down-regulated in females
*MRPL23*	0.005	0.68	0.005	-0.67	0.030	-1.21	Down-regulated in females
*KIRREL-IT1*	0.014	0.66	0.023	-0.59	0.049	-1.34	Down-regulated in females
*CACNA1C*	0.015	0.62	0.038	-0.50	0.012	-1.29	Down-regulated in females
*FAM229A*	0.007	0.62	0.042	-0.41	0.003	-1.30	Down-regulated in females
*PCED1A*	0.028	0.60	0.039	-0.56	0.042	-1.26	Down-regulated in females
*OR6C4*	0.019	0.59	0.029	-0.53	0.009	-1.33	Down-regulated in females
*MED19*	0.030	0.58	0.025	-0.60	0.031	-1.23	Down-regulated in females
*RFPL1*	0.038	0.57	0.031	-0.60	0.049	-1.31	Down-regulated in females
*SNORA75*	0.040	0.57	0.036	-0.58	0.049	-1.77	Down-regulated in females
*FBLN1*	0.030	0.56	0.042	-0.52	0.014	-1.51	Down-regulated in females
*ADAM19*	0.028	0.56	0.014	-0.66	0.020	-1.30	Down-regulated in females
*SLC2A11*	0.043	0.53	0.017	-0.66	0.039	-1.26	Down-regulated in females
*G6PD*	0.023	0.52	0.006	-0.68	0.004	-1.19	Down-regulated in females
*NIT1*	0.029	0.50	0.004	-0.75	0.007	-1.25	Down-regulated in females
*MAP3K13*	0.015	0.49	0.001	-0.80	0.045	-1.15	Down-regulated in females
*MIR519C*	0.044	0.47	0.013	-0.63	0.005	-1.28	Down-regulated in females
*SCGB2A2*	0.035	0.46	0.003	-0.77	0.005	-1.43	Down-regulated in females
*CXCR2P1*	0.031	0.44	0.021	-0.48	0.001	-1.37	Down-regulated in females
*IGBP1P1*	0.041	0.41	0.009	0.58	0.008	1.50	Up-regulated in females
*MIR548J*	0.007	0.39	0.001	-0.60	0.000	-1.74	Down-regulated in females
*CCDC117*	0.045	0.39	0.001	-0.86	0.012	-1.23	Down-regulated in females
*CLPB*	0.034	-0.42	0.003	0.66	0.001	1.26	Up-regulated in females
*LINC00330*	0.007	-0.45	0.000	0.86	0.020	1.32	Up-regulated in females
*PKD1L3*	0.033	-0.48	0.003	0.79	0.016	1.33	Up-regulated in females
*OSR1*	0.048	-0.49	0.009	0.73	0.040	1.28	Up-regulated in females
*SUSD4*	0.011	-0.50	0.001	0.84	0.005	1.41	Up-regulated in females
*HPGD*	0.006	-0.53	0.000	0.83	0.013	1.27	Up-regulated in females
*FLJ32154*	0.034	-0.53	0.028	0.55	0.008	1.42	Up-regulated in females
*KRT4*	0.024	-0.54	0.007	0.69	0.010	1.32	Up-regulated in females
*CACNA1I*	0.030	-0.54	0.024	0.58	0.010	1.36	Up-regulated in females
*SCARB1*	0.036	-0.55	0.019	0.65	0.038	1.28	Up-regulated in females
*DQX1*	0.029	-0.56	0.016	0.64	0.017	1.24	Up-regulated in females
*OR8U1*	0.016	-0.59	0.006	0.70	0.020	1.52	Up-regulated in females
*DOK2*	0.008	-0.59	0.002	0.77	0.013	1.26	Up-regulated in females
*ENTPD2*	0.027	-0.59	0.037	0.55	0.023	1.13	Up-regulated in females
*CLEC10A*	0.026	-0.59	0.020	0.63	0.041	1.31	Up-regulated in females
*CDH4*	0.030	-0.60	0.044	0.54	0.045	1.20	Up-regulated in females
*CLIP4*	0.006	-0.60	0.002	0.74	0.003	1.49	Up-regulated in females
*CCHCR1*	0.016	-0.62	0.023	0.57	0.016	1.41	Up-regulated in females
*C14orf79*	0.008	-0.63	0.007	0.65	0.006	1.33	Up-regulated in females
*PRKCD*	0.007	-0.63	0.030	0.46	0.004	1.31	Up-regulated in females
*ZNF490*	0.018	-0.64	0.036	0.55	0.036	1.15	Up-regulated in females
*TMEM51*	0.009	-0.66	0.022	0.54	0.010	1.50	Up-regulated in females
*CSTA*	0.010	-0.67	0.020	0.57	0.018	1.48	Up-regulated in females
*PLCB2-AS1*	0.011	-0.67	0.022	0.57	0.022	1.25	Up-regulated in females
*GMEB2*	0.003	-0.67	0.002	0.70	0.046	1.09	Up-regulated in females
*RNPEP*	0.001	-0.67	0.017	-0.43	0.040	-1.08	Down-regulated in females
*FGR*	0.002	-0.68	0.002	0.65	0.002	1.31	Up-regulated in females
*HLA-DPB2*	0.007	-0.70	0.038	0.47	0.012	1.51	Up-regulated in females
*STIL*	0.005	-0.70	0.032	0.46	0.007	1.20	Up-regulated in females
*TIAM1*	0.001	-0.77	0.003	0.63	0.015	1.22	Up-regulated in females

*Duplicate, uncharacterized and non-annotated gene transcripts were removed.

BMI = body mass index

The biological processes common to all three patient factors (age, BMI and sex) were cellular hypertonic response, smooth muscle contraction, positive regulation of wound healing and production of interleukin-10 and interleukin-12 (Table 6).

**Table 6:** Biological processes common to BMI age and sex*.

*Process*	*P value*	*FDR*
cellular hypotonic response	3.376E-09	7.141E-06
hypotonic response	8.812E-09	9.319E-06
smooth muscle contraction	4.369E-08	3.080E-05
tonic smooth muscle contraction	1.021E-07	5.398E-05
positive regulation of wound healing	1.917E-07	7.358E-05
interleukin-12 production	2.435E-07	7.358E-05
interleukin-10 production	2.435E-07	7.358E-05
aorta smooth muscle tissue morphogenesis	4.254E-07	1.125E-04
calcium ion transport into cytosol	8.911E-07	2.094E-04
cytosolic calcium ion transport	1.017E-06	2.150E-04
smooth muscle tissue development	1.506E-06	2.896E-04
regulation of metal ion transport	2.426E-06	4.276E-04
bleb assembly	2.652E-06	4.315E-04
regulation of calcium ion transport	3.071E-06	4.639E-04
cellular response to osmotic stress	4.102E-06	4.899E-04
circulatory system development	5.410E-06	4.899E-04
cardiovascular system development	5.410E-06	4.899E-04
regulation of sphingomyelin catabolic process	5.422E-06	4.899E-04
regulation of phospholipid scramblase activity	5.422E-06	4.899E-04
positive regulation of sphingomyelin catabolic process	5.422E-06	4.899E-04
positive regulation of phospholipid scramblase activity	5.422E-06	4.899E-04
positive regulation of glucosylceramide catabolic process	5.422E-06	4.899E-04
regulation of glucosylceramide catabolic process	5.422E-06	4.899E-04
membrane depolarization during action potential	5.749E-06	4.899E-04
positive regulation of cell migration	5.791E-06	4.899E-04
calcium ion import	6.227E-06	5.066E-04
regulation of ion transport	6.896E-06	5.280E-04
positive regulation of cell motility	6.990E-06	5.280E-04
positive regulation of cellular component movement	8.671E-06	6.324E-04
positive regulation of locomotion	9.711E-06	6.645E-04
regulation of phospholipid catabolic process	9.740E-06	6.645E-04
peptidyl-threonine phosphorylation	1.548E-05	1.023E-03
peptidyl-threonine modification	1.971E-05	1.263E-03
positive regulation of endothelial cell migration	2.756E-05	1.714E-03
regulation of wound healing	3.231E-05	1.952E-03
membrane depolarization	3.647E-05	2.143E-03
artery morphogenesis	3.749E-05	2.143E-03
aorta morphogenesis	5.251E-05	2.892E-03
calcium-mediated signaling using extracellular calcium source	5.398E-05	2.892E-03
artery development	5.698E-05	2.892E-03
muscle contraction	5.740E-05	2.892E-03
calcium ion transmembrane transport	5.744E-05	2.892E-03
calcium ion transport	6.194E-05	3.046E-03
aorta development	6.956E-05	3.344E-03
response to hydrogen peroxide	7.850E-05	3.638E-03
positive regulation of ceramide biosynthetic process	8.084E-05	3.638E-03
positive regulation of sphingolipid biosynthetic process	8.084E-05	3.638E-03
blood vessel development	9.356E-05	4.123E-03
divalent metal ion transport	9.724E-05	4.197E-03
positive regulation of epithelial cell migration	1.085E-04	4.434E-03

*as determined by GeneGo MetaCore.

BMI = body mass index, FDR = false discovery rate

As mentioned above, gene transcripts exclusively associated with age, BMI and sex were respectively 592, 509, and 484 which is about 8–10 fold higher than the gene transcripts common to all three factors (55). These results indicate that age, BMI and sex have differential impact on the expression profile of gene transcripts in macroscopically normal articular cartilage.

### Baseline gene expression values in healthy cartilage

A list of 27,641 gene transcripts with their log_2_ transformed relative signal intensity values is presented in S4 Table. These gene transcripts and their signal intensity values can be used to compare with the gene transcripts and signal intensity values obtained from OA cartilage.

### Expression of the OA genes identified by genetic associations and GWAS and OA gene expression

It is known that approximately 50% of patients with meniscus tears go on to develop OA [36]. We questioned whether there were phenotype differences that could be detected by the genes expressed in cartilage at the time of meniscus injury. Therefore, to explore potential phenotype differences in the seemingly healthy cartilage samples, we queried our dataset in two ways: for expression of gene alleles associated with the risk of developing OA [39] and for expression of gene transcripts shown to be up-regulated or down-regulated in OA cartilage [40].

**Fig 5:**
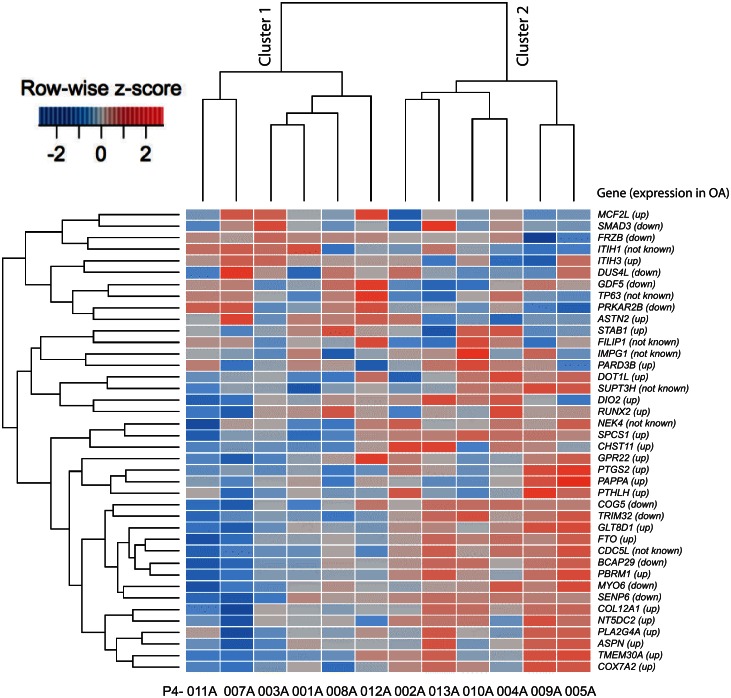
Heat map view of z-scored expression profiles of genes associated with OA risk-alleles. Hierarchical clustering identified two clusters that have distinct expression pattern. When known, the expression pattern of OA-associated genes is provided in parentheses.

Recent GWAS studies along with several adequately powered candidate gene studies have yielded a number of risk-alleles for OA. This allele number is now sufficiently large to allow conclusions regarding the nature of genetic susceptibility. Functional studies have revealed that effects on gene expression are likely to be one of the main mechanisms by which susceptibility to OA manifests in patients [39]. We generated a list of OA-associated candidate genes from published GWAS and adequately powered candidate gene studies and then computed their z-scores (which represent the expression difference of each gene to the averaged expression of all samples). The risk-alleles associated with OA may affect the expression level of their tagged genes. We performed hierarchical clustering based on z-scores of gene expression and two clear clusters of study patients emerged from this analysis. The genes associated with OA risk-alleles were differentially expressed in these clusters as indicated by an arrow next to the gene symbol (Fig 5). Cluster 2 of GWAS genes was shown to have a better match with the OA risk-alleles compared to Cluster 1 of GWAS genes. Out of the 33 genes with known expression pattern, 21 (64%) showed a similar direction of expression (18 up-regulated, 3 down-regulated).

**Fig 6:**
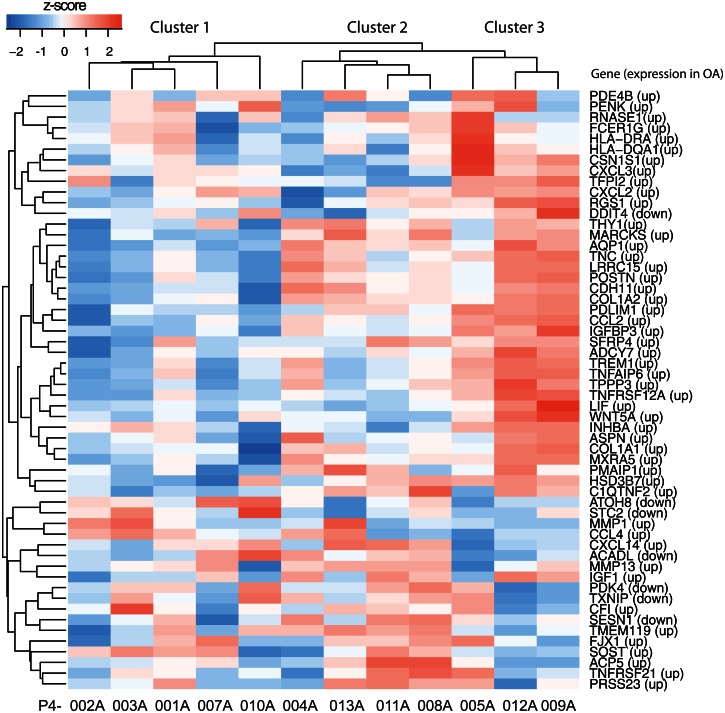
Heat map view of z-scored expression profiles of gene transcripts differentially expressed between healthy and OA cartilage. Hierarchical clustering separated the study patients into three distinct clusters. The list of gene transcripts used for cluster analysis was generated in a prior study by Karlsson et al. [40] that looked at the transcriptome-wide RNA expression of healthy and OA cartilage. This list represented all the expressed gene transcripts differentially expressed between healthy and OA cartilage at >15-fold difference.

Using 62 highly differentially expressed gene transcripts between healthy and OA cartilage (53 up-regulated, 9 down-regulated) from the published dataset [40], we found that our study patients clustered into three clusters encompassing 56 genes (Fig 6). These results suggest a varying response of patients to meniscus injury. Although preliminary due to the limited number of samples in our study, three of twelve patients were shown to segregate into Cluster 3 of OA-associated gene transcripts and demonstrated a remarkable similarity to the OA gene expression shown by Karlsson et. al., [40]. When compared to 53 gene transcripts up-regulated in the published paper, in our Cluster 3, 37/38 gene transcripts were up-regulated with only one transcript (*DDIT4*) showed down-regulation instead of up-regulation. Of the published 9 down-regulated gene transcripts, 5 were down-regulated in Cluster 3. These clusters are likely to have varying susceptibility to developing OA, with the greatest predilection for OA in the patients from Cluster 3.

### PCR findings to confirm microarray data

**Fig 7:**
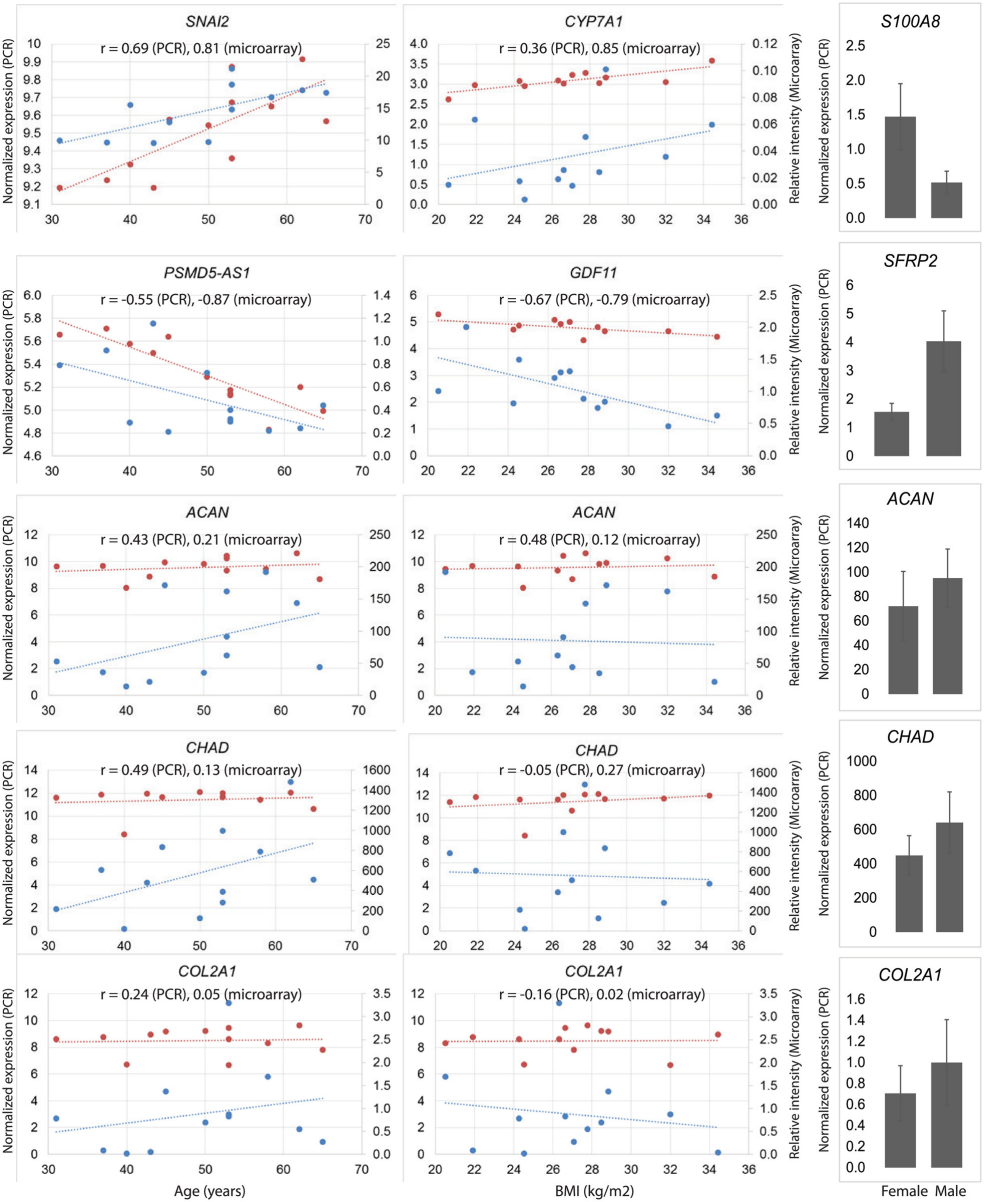
Findings from microfluidic digital PCR and comparison with microarray for selected gene transcripts. The expression of six differentially expressed gene transcripts was validated by microfluidic-based digital PCR. The expression profile of the entire set of gene transcripts obtained from TaqMan digital PCR assay was concordant with the microarray data. Pearson correlation coefficient was calculated for age (left panel; red microarray, blue digital PCR), and BMI (center panel; red microarray, blue digital PCR) and differential expression was presented for sex (right panel; these data are presented as mean with standard error of the mean). r = correlation coefficient

PCR was undertaken on selected gene transcripts from microarray data confirmed the findings from the microarray assay as shown in Fig 7. *SNAI2*, which was positively correlated with age by microarray (r = 0.81, P = 0.004), was also found to exhibit similar pattern by digital PCR (r = 0.69, P = 0.013). Similarly, *PSMD5AS1* was negatively correlated with age by microarray (r = −0.87, P = 0.001) and was also negatively correlated when determined by digital PCR (r = −0.55, P = 0.063). For BMI, *CYP7A1*, and *GDF11* were respectively positively and negatively correlated by microarray (r = 0.85, P = 0.001; r = −0.79, P = 0.005) and showed same trend by digital PCR (r = 0.36, P = 0.250; r = −0.67, P = 0.017). *S100A8* was highly expressed in females (2.95 fold, P = 0.032) while *SFRP2* was highly expressed in males (−2.34 fold, P = 0.041) and this pattern was consistent when measured by digital PCR as their respective fold change differences were respectively 2.85 fold (P = 0.074) and −2.60 fold (P = 0.040). In addition, we tested three cartilage genes (*ACAN*, *CHAD*, *COL2A1)* none of which were significantly different by age, BMI, and sex when analyzed by microarray or by digital PCR. Their correlation coefficients are provided on respective graphs (Fig 7). Overall, we found a high concordance between PCR and microarray results.

## Discussion

This study reports the transcriptome profile of macroscopically normal (intact) cartilage (based on imaging and arthroscopic evaluation) procured from knees with meniscus tears undergoing partial meniscectomy. While some segregation for age, BMI and sex differences is seen, one of the primary roles of this dataset will be to provide near healthy cartilage transcriptome data for comparison with other cohorts on the spectrum of healthy to OA cartilage. We also noted that age, BMI and sex were differentially associated with the gene expression profile in this cartilage. Only a handful of gene transcripts and biological processes were common to all three factors (age, BMI, sex).

Approximately 50% of patients with meniscus tears go on to develop OA [36]. We questioned whether there were phenotype differences that could be detected by the genes expressed in cartilage at the time of meniscus injury. We used two approaches: comparison with the expression of genes associated with OA by genetic studies [39] and comparison with gene transcripts expressed in OA cartilage [40]. Most GWAS-positive single nucleotide polymorphisms i.e. SNPs, are not in the coding sequence, but in regulatory regions [41], thus GWAS-associated genes may be more likely to show expression differences, particularly in a tissue that is involved in the disease [42]. When we explored differences in the cartilage for expression of candidate gene alleles associated with the risk of developing OA [39], two clear subsets of cartilage samples emerged potentially suggesting that patients with gene expression associated with genetic risk-alleles may be more likely to develop OA. When we explored differences in study patients based on gene transcripts differentially expressed between healthy and diseased cartilage [40], we identified three clusters of patients. One of these clusters, i.e. Cluster 3 (of OA-associated gene transcripts) made up of three of twelve patients, exhibited remarkable similarity to the OA gene expression pattern with 37 of 38 up-regulated genes demonstrating similar expression. These three patients were not alike in age (65, 40, 31 years), BMI (27.1, 24.5, 24.3 kg/m^2^) or sex (2 females, 1 male). Interestingly, two of these patients (P4-005A and P4-009A) were also in Cluster 2 of the GWAS genes showing more similarity with expression of the OA risk-alleles. One gene in particular was associated with both, *ASPN*, encoding the Asporin protein which binds to TGF-β [43]. In GWAS and candidate gene studies, allelic variants with D-repeat polymorphism of *ASPN* were associated with OA, with increased expression from some variants. In our Cluster 3, all three patients demonstrated high levels of *ASPN* transcripts. As specific variants have been shown to result in higher gene expression, it would be interesting to determine the allelic composition of our twelve patients.

Although association between these distinct expression patterns of OA risk-alleles or OA-associated gene transcripts and OA initiation warrants further investigation, our preliminary analysis suggests that a small subset of patients expresses an OA gene expression profile. While the clustering from both analyses (OA risk-alleles and OA-associated gene transcripts) is informative in providing a clue for variation in individual’s response to injury, confirmation can only come from a follow-up study on patients to identify those who develop OA.

Age appears to be an important factor relating to molecular changes in cartilage. The primary biological processes affected with aging included intracellular signal transduction, fat cell differentiation, cell-cell communication, and necroptotic process. The intracellular signal transduction pathway, involved in production of cytokines as well as other inflammatory compounds and enzymes, is commonly affected by several mechanisms in chondrocytes. For instance, it has been shown that nitric oxide can induce this pathway along with inflammatory gene activation. In addition, both receptors of TNFα are involved in signal transduction after being activated by TNFα during processes occurring in cartilage loss and OA [44, 45]. Similarly, IL-1β stimulated IL-1β receptor initiates an intracellular signaling pathway [46], which in concert with reactive oxygen species further stimulates apoptosis [47]. Thus, aging appears to initiate an intracellular signal transduction detrimental to joint health. Intervention of intracellular signal transduction pathways in cartilage could result in suppression of its destruction and amelioration of inflammation in OA. Appropriate cell matrix and cell-cell communication is critical to retain the structural and functional integrity of a tissue. However, the role of necroptotic process and regulation of fat cell differentiation in aging cartilage remains unknown.

The biological processes negatively correlated with age were primarily immune response and leukocyte activation. It is known that the aging immune system becomes less capable of resolving inflammation. Therefore, a negative correlation of immune response with aging cartilage hints towards decreased capability to suppress inflammation in the joint. This is compounded by a decrease in leukocyte activation in cartilage, which is regulated by IL-8 through p38 mitogen activated protein kinase signaling [48] and triggers neutrophil accumulation and destruction of cartilage [49]. IL-8, which is over expressed in OA chondrocytes, is involved in chondrocyte hypertrophic differentiation causing altered matrix synthesis and pathologic calcification in OA [50]. These findings are consistent with a mouse study that showed that functional annotations related to immune system development, immune regulation, and leukocyte activation were repressed with age after destabilization of medial meniscus [23]. An RNA-seq study on equine cartilage, however, showed that biological processes related to immune response were elevated in older horses [24] similar to what we have shown for human injured meniscus tissues [38].

The main biological processes positively correlated with BMI were related to regulation of memory T cell activation. It has been reported that after synovial inflammation is initiated, memory T cell activation leads to progression of inflammation in rheumatoid synovium [51]. This suggests that the elevated memory T cell activation with elevated BMI signals a higher inflammatory environment in the joint. The biological processes negatively correlated with BMI included response to mineralocorticoid and response to toxic substances but their significance in cartilage pathobiology has not been fully elucidated in the literature. Especially intriguing, growth and differentiation factor *GDF11*, is negatively associated with BMI. Since this factor is a negative regulator of chondrogenesis [52], our findings indicate that chondrogenesis is suppressed in patients with higher BMI. *GDF11 was* recently identified as the circulating factor that reverses age-related cardiac hypertrophy [53].

Our study also showed some influence of patients’ sex on transcriptome changes in cartilage. The gene transcripts representing processes such as detection of chemical stimulus involved in sensory perception of taste, L-ascorbic acid transport, peptidyl cysteine oxidation, aminoglycan metabolic process, and chaperone mediated protein folding were elevated in females. Their biological significance both in the context of cartilage and OA is not completely understood. However, the functional classifications repressed in females were represented by cell and tissue development and cell differentiation. Interestingly, chondrogenic differentiation of stem cells is influenced by sex as studies have demonstrated that muscle derived stem cells from females display less chondrogenic differentiation and lower quality cartilage healing than males [54]. Therefore, this finding suggests that processes repressed in females may contribute to poor chondrogenic potential and less healing ability, which is consistent with our findings of IPA analysis.

Our study has certain limitations. A potential limitation of the study is the use of cartilage from the notch. While the sample is not from the direct weight-bearing portion of the knee, it does come from a portion of the notch that can develop degenerative changes and OA. In addition, these findings would benefit from confirmation by immunohistochemistry as well as from an independent cohort.

Taken together, these findings may identify pathways that put older and heavier patients, as well as females, at greater risk for knee OA after partial meniscectomy. While it is known that patients with previous knee surgery undergo total knee arthroplasty at a younger age than patients without previous knee surgery [55], the exact biologic underpinnings of this difference are not established. Establishing a genetic risk for OA is complicated by the various phenotypes of OA and by the large populations necessary for GWAS studies. This list of risk-alleles associated genes will continue to grow over the next few years and can be used to probe our dataset. Importantly, we will be able to test the hypothesis by following up on the joint health of these individuals in our study. These findings may be the first step towards better understanding of these pathways, which could lead to novel and improved therapies to delay or prevent the development of OA in these patients. Our study demonstrates that gene expression of cartilage is associated with age, BMI, and sex in patients undergoing partial meniscectomy. The pattern of activation and suppression of genes associated with aging differs distinctly from the pattern associated with sex or BMI. Nevertheless, our current findings provide a novel baseline dataset relating patient factors to the transcriptome of disease free cartilage.

While transcriptome profiling in conjunction with OA risk-alleles and OA-related gene transcripts suggests a molecular “pre-OA” phenotype in a subset of patients, the ability to predict a population at risk for can only be assessed by future radiographic and clinical outcomes. Furthermore, validation and generalization of the findings from the current study necessitate confirmation in a distinct, larger cohort of patients.

## Supporting Information

S1 Table Gene transcripts correlated with age. (PDF)

S2 Table Gene transcripts correlated with BMI. (PDF)

S3 Table Gene transcripts differentially expressed by sex. (PDF)

S4 Table Gene transcripts relative intensity values. (PDF)
