# Supplementary material for: RNA Microarray Analysis of Macroscopically Normal Articular Cartilage from Knees Undergoing Partial Medial Meniscectomy: Potential Prediction of the Risk for Developing Osteoarthritis
Source: PLoS One. 2016 May 12;11(5):e0155373. doi: 10.1371/journal.pone.0155373 (PMC4865200; doi:10.1371/journal.pone.0155373)
Supplement: S1 Table — (PDF) [file pone.0155373.s001.pdf]

Supplementary Table 1: Gene transcripts correlated with age*

Gene symbol	*P value*	*r*	*Gene symbol*	*P value*	*r*
Gene transcripts positively correlated			*Gene transcripts negatively correlated*		
TSC2	0.004	0.81	*PFKFB2*	0.001	-0.87
SNAI2	0.004	0.81	*PSMD5-AS1*	0.001	-0.87
SLC35E1	0.003	0.81	*CD14*	0.001	-0.84
TBC1D3P1-DHX40P1	0.004	0.81	*FCGR2B*	0.002	-0.82
DCUN1D2-AS2	0.004	0.80	*TAGAP*	0.003	-0.82
PREX2	0.005	0.80	*OR2M3*	0.002	-0.82
IFITM2	0.004	0.79	*SLC8A1-AS1*	0.003	-0.82
DOT1L	0.004	0.79	*DIRC3*	0.003	-0.81
STXBP4	0.002	0.79	*ATP8B4*	0.003	-0.81
MOV10	0.005	0.79	*RASSF2*	0.004	-0.80
CNNM3	0.006	0.78	*MMP24-AS1*	0.000	-0.80
CDK2	0.005	0.78	*FGD2*	0.005	-0.79
RAD51-AS1	0.007	0.78	*LINC00489*	0.005	-0.79
ZNF546	0.005	0.77	*HNF1A-AS1*	0.006	-0.78
TRIB2	0.005	0.77	*TCEAL3*	0.007	-0.77
SLC25A37	0.007	0.77	*CYTIP*	0.007	-0.77
MIR4465	0.006	0.77	*NCF1B*	0.007	-0.77
SLC39A1	0.005	0.77	*SLC5A8*	0.007	-0.77
XAB2	0.007	0.76	*RAB11FIP1*	0.007	-0.77
TAF6L	0.008	0.76	*RNU7-25P*	0.009	-0.77
GMCL1	0.006	0.75	*POTEE*	0.007	-0.76
RING1	0.009	0.75	*LINC00891*	0.010	-0.76
DTWD1	0.007	0.75	*PPEF1-AS1*	0.011	-0.76
TACSTD2	0.007	0.75	*EFHC2*	0.010	-0.76
C6orf165	0.007	0.74	*GPBAR1*	0.009	-0.76
DCAKD	0.011	0.74	*HAVCR2*	0.010	-0.75
IRF2BP1	0.010	0.74	*GAGE2A*	0.006	-0.75
FAM215B	0.009	0.74	*FCER1A*	0.009	-0.75
RPL36	0.008	0.73	*THEG*	0.010	-0.74
TMUB2	0.011	0.73	*SERPINB13*	0.010	-0.74
ANGPTL7	0.009	0.73	*C19orf57*	0.006	-0.74
SYNGAP1	0.009	0.73	*BTNL3*	0.012	-0.74
C17orf100	0.010	0.73	*PBOV1*	0.011	-0.74
ADARB1	0.016	0.73	*CEACAM18*	0.013	-0.74
ANKZF1	0.004	0.73	*BTC*	0.007	-0.74
MIR4671	0.015	0.73	*HBG2*	0.012	-0.74
FXYD6	0.014	0.73	*MS4A4E*	0.012	-0.74
CLIP3	0.011	0.73	*IL2RG*	0.014	-0.74
USP20	0.010	0.73	*TRBV30*	0.014	-0.74
GATSL3	0.016	0.73	*HPGDS*	0.008	-0.74
KIAA0355	0.012	0.73	*NRG1*	0.009	-0.74
PVRIG	0.005	0.73	*LCE1C*	0.008	-0.74
FOXO3	0.016	0.72	*IGKV1-37*	0.015	-0.74

SSBP3	0.018	0.72	*RNU1-15P*	0.012	-0.73
POLE2	0.006	0.72	*GLT6D1*	0.011	-0.73
RNU5A-2P	0.006	0.72	*XIRP2-AS1*	0.008	-0.73
DRD4	0.016	0.72	*CD226*	0.013	-0.73
SLC7A2	0.015	0.72	*SNORD46*	0.012	-0.73
MIR485	0.012	0.72	*PRKXP1*	0.014	-0.73
ZNF532	0.015	0.72	*DAB1*	0.016	-0.73
SCARNA8	0.017	0.72	*RFX4*	0.014	-0.73
PTP4A3	0.012	0.72	*TMCO5B*	0.015	-0.73
TMEM99	0.018	0.71	*SLC22A1*	0.016	-0.73
SGSM3	0.017	0.71	*FAM133A*	0.016	-0.73
GPR21	0.012	0.71	*CD83*	0.012	-0.72
HOXC-AS1	0.022	0.71	*PER4*	0.015	-0.72
RGMA	0.022	0.71	*MT1B*	0.006	-0.72
FBXL13	0.007	0.70	*IL1RN*	0.016	-0.72
KPNA5	0.019	0.70	*RNA5SP44*	0.016	-0.72
C1GALT1C1	0.019	0.70	*SPINT1*	0.006	-0.72
TRMT2A	0.023	0.70	*C2CD4A*	0.018	-0.72
LIN37	0.014	0.70	*KCNJ9*	0.004	-0.72
NUDT4	0.009	0.70	*LTA4H*	0.011	-0.71
ZBTB11-AS1	0.021	0.69	*CLEC12A*	0.018	-0.71
MAK	0.024	0.69	*ARMCX3-AS1*	0.018	-0.71
CHRNE	0.020	0.69	*C11orf86*	0.020	-0.71
SEC31A	0.023	0.69	*GUCD1*	0.017	-0.71
CACHD1	0.027	0.69	*GAGE12H*	0.011	-0.71
NKAIN4	0.022	0.69	*ARHGEF5*	0.017	-0.71
TDH	0.026	0.69	*CD3EAP*	0.012	-0.71
FAM194A	0.024	0.69	*FABP2*	0.019	-0.71
MIR1224	0.011	0.69	*CD200R1*	0.016	-0.71
DDIT4L	0.028	0.69	*NTN3*	0.010	-0.71
LIPC	0.025	0.68	*RPS6KA5*	0.016	-0.71
RCAN2	0.015	0.68	*SPIN2A*	0.016	-0.71
IL6ST	0.024	0.68	*P2RX7*	0.014	-0.71
PCDHB9	0.027	0.68	*HLA-DQA2*	0.010	-0.71
TRAF7	0.028	0.68	*SNX10*	0.015	-0.71
IFITM4P	0.026	0.68	*SNN*	0.018	-0.71
HIC2	0.001	0.68	*LRRC8D*	0.013	-0.71
MIR543	0.024	0.68	*ANKRD18B*	0.018	-0.70
FAM195A	0.027	0.68	*EPB41L3*	0.012	-0.70
FOXN4	0.011	0.68	*TRAJ56*	0.018	-0.70
RANGRF	0.032	0.67	*TEKT2*	0.023	-0.70
TTC38	0.033	0.67	*GOLGA6L4*	0.008	-0.70
B3GNT8	0.028	0.67	*FAM90A12P*	0.009	-0.70
GNG8	0.019	0.67	*VCX3A*	0.008	-0.70
BIRC3	0.022	0.67	*HLA-DPB1*	0.020	-0.70
SLC25A38	0.021	0.67	*NCEH1*	0.015	-0.70
CTGF	0.019	0.67	*MS4A6A*	0.014	-0.70

GPR56	0.031	0.67	*CERS4*	0.024	-0.70
GATM-AS1	0.028	0.67	*KIAA1656*	0.021	-0.70
PER1	0.020	0.67	*LRP5*	0.020	-0.70
OR2A1	0.032	0.67	*ALDH1A1*	0.015	-0.69
PRDM15	0.033	0.67	*MAP7D2*	0.007	-0.69
RNU12	0.025	0.67	*APOBEC4*	0.014	-0.69
ISG20	0.014	0.67	*ADAMTS3*	0.025	-0.69
TMEM240	0.027	0.67	*RNA5-8SP3*	0.019	-0.69
ACOT4	0.026	0.67	*LGALS2*	0.019	-0.69
LINC00568	0.035	0.66	*ACP2*	0.026	-0.69
CNOT3	0.033	0.66	*PARP15*	0.020	-0.69
C4BPA	0.024	0.66	*NOD2*	0.020	-0.69
HIST1H4B	0.032	0.66	*ALOX5AP*	0.020	-0.69
GUSBP9	0.030	0.66	*SNCA*	0.016	-0.69
OR52H1	0.029	0.66	*CES1*	0.020	-0.69
LDLRAP1	0.035	0.66	*LINC00656*	0.025	-0.69
YPEL3	0.032	0.66	*LILRB3*	0.017	-0.68
SYMPK	0.021	0.66	*RNASE2*	0.026	-0.68
ABCD4	0.038	0.66	*SNORA22*	0.026	-0.68
MIR938	0.031	0.66	*IGHV3-43*	0.027	-0.68
ARFGAP1	0.038	0.66	*FAM90A20P*	0.024	-0.68
RAB6C	0.040	0.65	*SH2D5*	0.026	-0.68
IGBP1	0.034	0.65	*MROH6*	0.017	-0.68
MIR384	0.028	0.65	*WDFY4*	0.029	-0.68
IGHD	0.029	0.65	*CSAG2*	0.016	-0.68
RAB1B	0.031	0.65	*FCGR2A*	0.017	-0.68
PYGL	0.036	0.65	*FAM90A1*	0.008	-0.67
SMIM18	0.036	0.65	*ZFP69*	0.017	-0.67
ZNF292	0.008	0.65	*SAMHD1*	0.029	-0.67
TCEA3	0.039	0.65	*CXorf21*	0.017	-0.67
JMY	0.041	0.65	*VRK1*	0.021	-0.67
CNOT10	0.013	0.65	*GPR183*	0.010	-0.67
IPO11	0.026	0.65	*RNU7-6P*	0.023	-0.67
MON1A	0.022	0.65	*FLJ42102*	0.023	-0.67
SOS1-IT1	0.029	0.65	*APOC1*	0.019	-0.67
GTF2IRD2	0.031	0.65	*C1QTNF9B-AS1*	0.024	-0.67
FAM19A5	0.034	0.65	*RNA5SP188*	0.019	-0.67
STXBP5L	0.027	0.65	*TAF7L*	0.017	-0.67
CLDN20	0.042	0.65	*SEPT14*	0.033	-0.67
MARK4	0.024	0.65	*CD180*	0.022	-0.67
MIR1185-2	0.032	0.65	*MAPKAPK3*	0.018	-0.67
PLEKHM1	0.025	0.65	*MIR591*	0.026	-0.67
GNL1	0.032	0.65	*TUBB4B*	0.017	-0.67
TMEM255B	0.028	0.65	*LINC00700*	0.020	-0.66
PPT2-EGFL8	0.029	0.65	*TRIM71*	0.030	-0.66
GGCT	0.029	0.65	*RPL36P20*	0.017	-0.66
ATG10	0.035	0.65	*CECR1*	0.026	-0.66

FBXW5	0.028	0.65	*SYK*	0.030	-0.66
RPS29	0.043	0.64	*SNORA79*	0.018	-0.66
ANK3	0.031	0.64	*TRDJ4*	0.026	-0.66
FAM211A	0.041	0.64	*FAM83G*	0.029	-0.66
FGFRL1	0.040	0.64	*AIF1*	0.019	-0.66
SLC25A2	0.030	0.64	*LINC00548*	0.019	-0.66
OIP5	0.033	0.64	*CXCL16*	0.027	-0.66
HSFY2	0.027	0.64	*PLEK*	0.031	-0.66
CCNB1IP1	0.041	0.64	*SERTAD1*	0.039	-0.66
TRAJ21	0.026	0.64	*SECTM1*	0.020	-0.66
TTC25	0.033	0.64	*NPAP1*	0.021	-0.66
XRCC2	0.008	0.64	*SCGB3A2*	0.034	-0.65
C19orf67	0.041	0.64	*HCLS1*	0.037	-0.65
AKT3	0.036	0.64	*FCAR*	0.030	-0.65
PROP1	0.024	0.64	*LPAR5*	0.039	-0.65
PLEKHG2	0.038	0.64	*TMEM86B*	0.039	-0.65
OR7E24	0.038	0.64	*MIR3973*	0.039	-0.65
ADAMTS14	0.042	0.64	*AMY1A*	0.039	-0.65
CCDC57	0.048	0.64	*NAP1L2*	0.035	-0.65
CLK2P	0.021	0.64	*APOBEC1*	0.038	-0.65
FLJ35934	0.015	0.64	*GAGE12C*	0.027	-0.65
RUSC2	0.016	0.64	*THSD4*	0.041	-0.65
GARNL3	0.041	0.64	*MIR3922*	0.035	-0.65
KIAA1147	0.041	0.63	*VAMP8*	0.027	-0.65
ZNF8	0.047	0.63	*ARSE*	0.038	-0.65
MIR451A	0.049	0.63	*HLA-DQB1*	0.039	-0.65
CAMTA2	0.044	0.63	*SCUBE1*	0.033	-0.65
MIRLET7A3	0.026	0.63	*NEUROD4*	0.025	-0.65
FAM160B2	0.044	0.63	*SSX2*	0.025	-0.65
SCARNA17	0.047	0.63	*AGRP*	0.041	-0.65
OR8B8	0.011	0.63	*PRM3*	0.042	-0.64
LNX1-AS2	0.047	0.63	*PPP1R3F*	0.044	-0.64
BOLA1	0.049	0.63	*HCST*	0.021	-0.64
ZNF891	0.038	0.63	*RALGDS*	0.027	-0.64
FEZF1-AS1	0.048	0.63	*ZNF555*	0.023	-0.64
B3GALT4	0.043	0.62	*OR1L4*	0.032	-0.64
MIR1297	0.040	0.62	*KRT23*	0.038	-0.64
BRWD1-IT2	0.038	0.62	*OR1I1*	0.028	-0.64
KLHL4	0.048	0.62	*MSMP*	0.040	-0.64
TMEM91	0.044	0.62	*SNORD116-11*	0.045	-0.64
C1RL-AS1	0.044	0.62	*SIGLEC16*	0.019	-0.64
AK9	0.049	0.62	*AOX2P*	0.039	-0.64
GRIK1-AS2	0.037	0.62	*SIGLEC1*	0.033	-0.64
DENND3	0.035	0.62	*ERAS*	0.041	-0.64
LINC00484	0.043	0.62	*PAQR8*	0.045	-0.64
OR8J3	0.045	0.62	*HLA-DRA*	0.028	-0.64
RNF167	0.031	0.62	*LINC00284*	0.044	-0.64

PURA	0.042	0.62	*NKX3-1*	0.046	-0.64
GPER	0.045	0.62	*RNA5SP64*	0.046	-0.64
BRE	0.030	0.62	*SIGLEC9*	0.046	-0.64
ADIRF	0.049	0.62	*BTK*	0.030	-0.64
RNA5SP271	0.034	0.62	*POP1*	0.029	-0.64
NUMB	0.044	0.62	*KLRC4-KLRK1*	0.033	-0.64
KCNK18	0.020	0.62	*HVCN1*	0.045	-0.64
FNDC1-IT1	0.047	0.61	*TMCO4*	0.033	-0.63
TMEM104	0.033	0.61	*FAM78B*	0.048	-0.63
RPL37	0.038	0.61	*PARK2*	0.033	-0.63
ZNF296	0.029	0.61	*CABP7*	0.022	-0.63
INPP5B	0.030	0.61	*FAM71A*	0.034	-0.63
PHLDA3	0.032	0.61	*ANKRD20A3*	0.017	-0.63
AKAP8L	0.043	0.61	*RGS8*	0.032	-0.63
STK25	0.040	0.61	*NKAPP1*	0.048	-0.63
ADRA1B	0.046	0.61	*MTPAP*	0.030	-0.63
MUC1	0.037	0.61	*RNF113B*	0.041	-0.63
C1S	0.046	0.61	*ASAH1*	0.031	-0.63
RAB24	0.033	0.61	*THAP2*	0.035	-0.63
IL21	0.049	0.61	*C16orf89*	0.015	-0.63
NEBL-AS1	0.036	0.61	*CSF2RA*	0.028	-0.63
SMAD3	0.047	0.61	*THOC3*	0.046	-0.63
TXNRD2	0.035	0.61	*SPRR2B*	0.045	-0.63
GOLGA8A	0.016	0.61	*GAPT*	0.043	-0.63
MCTP2	0.021	0.61	*LIPT2*	0.045	-0.63
INSIG1	0.030	0.60	*ADAM23*	0.047	-0.63
HDAC5	0.049	0.60	*ITGAX*	0.046	-0.63
NNMT	0.028	0.60	*KLRC2*	0.050	-0.63
EGFL8	0.045	0.60	*ECHDC3*	0.030	-0.63
WDR70	0.036	0.60	*SNORD88B*	0.042	-0.63
WASF3	0.039	0.60	*BLNK*	0.035	-0.63
MAN1B1	0.037	0.60	*TEX13A*	0.012	-0.63
ISG20L2	0.042	0.60	*COBL*	0.036	-0.63
EGFL6	0.037	0.60	*APOM*	0.043	-0.63
PAN3-AS1	0.027	0.60	*HLA-DMB*	0.034	-0.62
SPDYA	0.049	0.60	*ANKRD54*	0.028	-0.62
USP27X-AS1	0.044	0.59	*MAST1*	0.033	-0.62
DCUN1D2-AS1	0.048	0.59	*PRSS55*	0.032	-0.62
MPDZ	0.049	0.59	*LPA*	0.034	-0.62
HSFY1P1	0.042	0.59	*LINC00964*	0.050	-0.62
MYO1D	0.041	0.59	*DYRK1B*	0.032	-0.62
AUTS2	0.014	0.59	*USP26*	0.033	-0.62
CUBN	0.036	0.59	*GAGE12J*	0.043	-0.62
SSR4	0.039	0.59	*C9orf135-AS1*	0.037	-0.62
KIAA1549L	0.049	0.59	*PRR22*	0.031	-0.62
GPR124	0.041	0.59	*LIPN*	0.046	-0.62
CCDC54	0.040	0.59	*CYSLTR2*	0.035	-0.62

GOLGA8B	0.024	0.59	*FAM90A10P*	0.043	-0.62
MIR3146	0.033	0.59	*CSH1*	0.027	-0.62
YPEL1	0.040	0.59	*OR9A4*	0.034	-0.62
DOCK9	0.037	0.59	*LINC00365*	0.031	-0.62
MIR2116	0.025	0.59	*NOXA1*	0.030	-0.62
DLEU2L	0.036	0.58	*INPP5J*	0.015	-0.62
ZIC1	0.046	0.58	*RNASE1*	0.032	-0.62
SNORD116-20	0.042	0.58	*SCARA5*	0.047	-0.62
TXLNB	0.047	0.58	*SLC9A4*	0.042	-0.62
C2orf15	0.046	0.58	*SNAR-F*	0.045	-0.62
SPRY4	0.044	0.58	*MAGEA3*	0.045	-0.62
SNX19	0.049	0.58	*WAS*	0.032	-0.62
C1orf101	0.025	0.58	*C10orf85*	0.038	-0.62
PFKFB4	0.034	0.58	*LINC00578*	0.042	-0.61
STYXL1	0.049	0.58	*GCHFR*	0.034	-0.61
OLFM1	0.043	0.57	*DBIL5P2*	0.041	-0.61
SARDH	0.048	0.57	*PCSK2*	0.023	-0.61
RIPK3	0.043	0.57	*CXCR4*	0.030	-0.61
YBX3	0.045	0.57	*MIR338*	0.048	-0.61
OR2B6	0.042	0.57	*KIR3DL2*	0.043	-0.61
SNORD123	0.047	0.57	*GRM7-AS2*	0.028	-0.61
RPS2	0.048	0.57	*LANCL2*	0.040	-0.61
TGM3	0.046	0.57	*TLR1*	0.038	-0.61
KRTAP4-12	0.032	0.57	*IGHV3-33*	0.049	-0.61
PRDX2	0.040	0.57	*IRF8*	0.049	-0.61
NRN1	0.032	0.55	*LINC00901*	0.035	-0.61
BSN	0.036	0.55	*PILRA*	0.030	-0.61
RNF214	0.037	0.54	*FOXG1*	0.034	-0.61
AFF1	0.047	0.54	*IGBP1-AS1*	0.010	-0.61
PEBP1	0.048	0.54	*HFE2*	0.044	-0.60
TMEM209	0.049	0.53	*CTSL1P2*	0.049	-0.60
MIR770	0.047	0.52	*WDR38*	0.043	-0.60
TRADD	0.032	0.52	*IQCF3*	0.046	-0.60
PPFIA3	0.042	0.51	*MIR517B*	0.045	-0.60
ZNF616	0.037	0.49	*SNORA5A*	0.028	-0.60
INPP5K	0.045	0.46	*IL10RA*	0.046	-0.60
			SCARNA27	0.043	-0.60
			IFI30	0.033	-0.60
			ENDOU	0.049	-0.60
			PTPRJ	0.043	-0.60
			GREM2	0.011	-0.59
			ADAMTS16	0.036	-0.59
			TYROBP	0.046	-0.59
			ZNF90	0.039	-0.59
			UBQLNL	0.045	-0.59
			TLR2	0.043	-0.59
			RBP2	0.050	-0.59

			PI15	0.034	-0.58
			SSX4	0.045	-0.58
			OR1L6	0.037	-0.58
			PPAN-P2RY11	0.028	-0.58
			KRTAP13-1	0.034	-0.58
			PLEKHB2	0.045	-0.58
			SLC36A2	0.041	-0.58
			ARMCX4	0.045	-0.58
			LACTBL1	0.010	-0.58
			GS52	0.026	-0.57
			FLJ25917	0.016	-0.57
			BMP10	0.041	-0.57
			CYBB	0.046	-0.57
			MC3R	0.040	-0.57
			C8A	0.021	-0.57
			SLC10A4	0.047	-0.57
			HMGA1P7	0.050	-0.57
			OVOL1	0.035	-0.57
			FSCN3	0.048	-0.57
			TARM1	0.046	-0.56
			TCAP	0.046	-0.56
			LINC00388	0.039	-0.56
			CCDC19	0.020	-0.56
			MIR3157	0.029	-0.56
			GOLGA6L6	0.048	-0.56
			PPT1	0.023	-0.56
			FAM107B	0.050	-0.56
			IGHD2-21	0.029	-0.56
			CT64	0.047	-0.55
			SMTNL2	0.025	-0.55
			MIR3666	0.015	-0.55
			CCDC142	0.024	-0.54
			LINC00698	0.034	-0.54
			OR52K2	0.020	-0.54
			ACR	0.027	-0.54
			TDO2	0.042	-0.54
			MIR3937	0.042	-0.54
			PANX3	0.045	-0.54
			PEG3-AS1	0.039	-0.53
			RAD54L	0.035	-0.53
			SLC26A7	0.033	-0.53
			THEG5	0.045	-0.53
			SLC24A2	0.029	-0.52
			LINC00239	0.041	-0.51
			GH1	0.045	-0.51
			WDR87	0.031	-0.49

			SYCE1	0.044	-0.48

**Duplicate, uncharacterized and non-annotated gene transcripts were removed*
