# Supplementary material for: RNA Microarray Analysis of Macroscopically Normal Articular Cartilage from Knees Undergoing Partial Medial Meniscectomy: Potential Prediction of the Risk for Developing Osteoarthritis
Source: PLoS One. 2016 May 12;11(5):e0155373. doi: 10.1371/journal.pone.0155373 (PMC4865200; doi:10.1371/journal.pone.0155373)
Supplement: S2 Table — (PDF) [file pone.0155373.s002.pdf]

Supplementary Table 2: Gene transcripts correlated with BMI*

Gene symbol	*P value*	*r*	*Gene symbol*	*P value*	*r*
Gene transcripts positively correlated			*Gene transcripts negatively correlated*		
CYP7A1	0.001	0.85	*ERCC2*	0.002	-0.80
LINC00616	0.000	0.84	*TRIM49*	0.004	-0.80
MGC39372	0.001	0.83	*GDF11*	0.005	-0.79
TMEM235	0.001	0.82	*ZFYVE28*	0.006	-0.78
CCDC79	0.005	0.79	*AANAT*	0.006	-0.78
SLC26A5	0.005	0.78	*LAMTOR2*	0.004	-0.78
ZNF852	0.006	0.78	*BAAT*	0.007	-0.78
DDR1	0.004	0.77	*DIEXF*	0.003	-0.77
LRRC2	0.007	0.77	*FGD1*	0.008	-0.77
LYPD6	0.008	0.77	*SLC35B1*	0.010	-0.76
FFAR1	0.008	0.76	*TMEM243*	0.002	-0.76
IGLV4-69	0.008	0.76	*HEXIM1*	0.010	-0.76
CDHR3	0.010	0.76	*MICB*	0.010	-0.75
RGS21	0.011	0.75	*PSIMCT-1*	0.003	-0.75
ATXN3L	0.008	0.75	*SGOL1-AS1*	0.011	-0.75
RASSF10	0.012	0.75	*HIST1H2AJ*	0.010	-0.75
LINC00235	0.009	0.74	*KIAA0247*	0.002	-0.74
RBMS3-AS2	0.008	0.74	*RNASEH2B-AS1*	0.005	-0.74
SORBS2	0.013	0.74	*CCDC28B*	0.015	-0.73
HAO2	0.007	0.74	*ZNF846*	0.008	-0.73
HNRNPKP3	0.014	0.74	*MIR4703*	0.012	-0.73
RIC8A	0.014	0.73	*PGP*	0.003	-0.73
SLC6A15	0.008	0.73	*RHOA-IT1*	0.004	-0.73
AADACL4	0.011	0.73	*FAM174B*	0.016	-0.72
ANK1	0.008	0.73	*NXPH4*	0.014	-0.72
MGC21881	0.005	0.73	*ARFIP2*	0.007	-0.72
HES3	0.015	0.73	*AGBL5*	0.013	-0.72
GPR26	0.015	0.73	*OXNAD1*	0.014	-0.72
FLJ42842	0.010	0.73	*FERD3L*	0.020	-0.71
ACSS2	0.017	0.72	*GPR141*	0.006	-0.71
ISM2	0.011	0.72	*CPOX*	0.021	-0.71
LINC00467	0.013	0.72	*SLFNL1-AS1*	0.019	-0.70
RPL29	0.014	0.72	*MICA*	0.013	-0.70
HLA-A	0.017	0.72	*CEL*	0.023	-0.70
CASS4	0.008	0.72	*FAS*	0.023	-0.70
MEGF8	0.017	0.72	*ARHGAP22*	0.020	-0.70
ITGAL	0.010	0.72	*TRIM11*	0.024	-0.70
IL23A	0.009	0.71	*CHSY3*	0.019	-0.70
LRRC71	0.003	0.71	*FASN*	0.018	-0.69
TTPA	0.015	0.71	*TOP3A*	0.022	-0.69
GABRG1	0.010	0.71	*CRIP2*	0.018	-0.69
PTH2	0.020	0.71	*MIR4282*	0.016	-0.69
FAM47C	0.016	0.71	*HTR1B*	0.027	-0.69

TERT	0.016	0.71	*EMC8*	0.011	-0.69
KCNH8	0.020	0.71	*CDC14B*	0.015	-0.68
HPYR1	0.012	0.71	*FAM118A*	0.028	-0.68
MIR4765	0.020	0.70	*MIR451A*	0.016	-0.68
RIPPLY3	0.023	0.70	*EPS8L2*	0.023	-0.68
CARD17	0.022	0.70	*ZNF77*	0.023	-0.68
PPP2R2B-IT1	0.021	0.70	*EXOC8*	0.029	-0.68
UPF3A	0.015	0.70	*PYCR2*	0.013	-0.68
AKAP14	0.013	0.70	*PPAT*	0.011	-0.68
CLCN7	0.015	0.70	*TRBV21OR9-2*	0.025	-0.68
LINC00879	0.019	0.70	*IL1RAP*	0.022	-0.68
XCR1	0.021	0.70	*SPAG1*	0.027	-0.68
TAS2R14	0.018	0.70	*DGCR2*	0.030	-0.68
COL4A3	0.024	0.70	*DIAPH3-AS1*	0.030	-0.68
NRCAM	0.023	0.70	*FAM120C*	0.027	-0.68
AP4S1	0.016	0.69	*RASL12*	0.019	-0.68
ZNF676	0.012	0.69	*USP42*	0.031	-0.67
ELF3	0.026	0.69	*EIF2S3L*	0.031	-0.67
KCNQ1DN	0.025	0.69	*LYRM7*	0.012	-0.67
RASGRF1	0.017	0.69	*CLIC6*	0.020	-0.67
IGF2BP3	0.019	0.68	*TMEM5*	0.027	-0.67
MRGPRD	0.023	0.68	*KHSRP*	0.033	-0.67
NIPA1	0.010	0.68	*CACNA1C-AS4*	0.029	-0.67
PSG11	0.025	0.68	*IL36RN*	0.024	-0.67
ZNF345	0.015	0.68	*MIR450A2*	0.029	-0.67
DMD-AS3	0.028	0.68	*ADAT1*	0.016	-0.67
EMX2OS	0.022	0.68	*PRRT3*	0.032	-0.67
FLJ27255	0.019	0.68	*ZMAT2*	0.036	-0.66
GDF5	0.027	0.68	*KDM4A-AS1*	0.031	-0.66
MROH2A	0.024	0.68	*CHD6*	0.033	-0.66
LINC00502	0.015	0.68	*ASB8*	0.036	-0.66
MIR129-1	0.028	0.68	*GDI1*	0.031	-0.66
RMDN3	0.020	0.68	*BTD*	0.032	-0.66
FGF12-AS2	0.016	0.68	*ETV6*	0.032	-0.66
LINC00951	0.029	0.68	*RNA5SP268*	0.014	-0.66
GYPA	0.030	0.68	*NPRL2*	0.021	-0.66
LINC00933	0.026	0.68	*F8A3*	0.022	-0.66
ANKDD1A	0.023	0.68	*MIR1184-1*	0.022	-0.66
OR3A4P	0.005	0.67	*POLD4*	0.028	-0.65
PATE3	0.028	0.67	*MIMT1*	0.038	-0.65
MIR4268	0.025	0.67	*KIAA1958*	0.035	-0.65
POU2F3	0.030	0.67	*SLC35F1*	0.030	-0.65
PTRH1	0.022	0.67	*TIGD4*	0.040	-0.65
EXPH5	0.023	0.67	*CBLN3*	0.030	-0.65
NPFFR1	0.031	0.67	*MYO10*	0.024	-0.65
ANKRD2	0.033	0.67	*MRPL52*	0.025	-0.65
LINC00494	0.025	0.67	*PRSS48*	0.033	-0.65

TMCO5A	0.034	0.67	*CYP7B1*	0.028	-0.64
ENTHD2	0.001	0.67	*CCT6P3*	0.034	-0.64
GINS1	0.032	0.67	*SNORD13P2*	0.043	-0.64
ANKRD53	0.008	0.67	*TMEM127*	0.030	-0.64
HBQ1	0.010	0.67	*ARL14*	0.044	-0.64
TTC29	0.022	0.67	*SEC22C*	0.027	-0.64
PRSS12	0.023	0.67	*LAIR2*	0.041	-0.64
FLJ45513	0.009	0.67	*CRYM-AS1*	0.046	-0.64
RAMP2-AS1	0.035	0.66	*OR1Q1*	0.044	-0.64
LINC00910	0.022	0.66	*SNORA58*	0.045	-0.64
SOX1	0.013	0.66	*LRRC37A2*	0.019	-0.64
CNTNAP3	0.013	0.66	*CEP152*	0.028	-0.64
LINC00427	0.030	0.66	*DEDD2*	0.045	-0.64
ARMC2	0.032	0.66	*RBM42*	0.036	-0.64
TSRM	0.017	0.66	*MIR376C*	0.041	-0.64
GPHB5	0.010	0.66	*PPIL2*	0.047	-0.63
PNPLA2	0.028	0.66	*XBP1*	0.018	-0.63
ALX3	0.036	0.66	*MROH1*	0.048	-0.63
CHRNB2	0.030	0.66	*ATAT1*	0.049	-0.63
OCA2	0.032	0.66	*CCRL1*	0.024	-0.63
GPR25	0.029	0.66	*LOXL4*	0.039	-0.63
KCNK17	0.024	0.66	*HOXD9*	0.047	-0.63
SLC22A14	0.032	0.66	*MIR3167*	0.048	-0.63
MIR4791	0.029	0.66	*FAM3C*	0.019	-0.63
FAM156A	0.024	0.66	*CACNA1D*	0.030	-0.63
HOXA-AS3	0.025	0.66	*SKAP2*	0.044	-0.62
AIPL1	0.035	0.66	*PLGRKT*	0.042	-0.62
PDIK1L	0.035	0.66	*CRMP1*	0.047	-0.62
HELZ2	0.036	0.66	*SF3A2*	0.044	-0.62
VMAC	0.030	0.66	*FRMD6-AS1*	0.032	-0.62
AGFG2	0.036	0.65	*HAVCR1P1*	0.017	-0.62
MIR4540	0.020	0.65	*RNF40*	0.036	-0.62
CORIN	0.035	0.65	*MIR7-3*	0.047	-0.62
RNA5SP66	0.024	0.65	*ZSWIM3*	0.048	-0.62
TBX22	0.038	0.65	*SNORA70G*	0.017	-0.62
F2RL1	0.032	0.65	*AP4B1-AS1*	0.037	-0.62
GOLGA2P2Y	0.019	0.65	*SNAI1*	0.046	-0.62
FUT2	0.030	0.65	*NIPSNAP1*	0.043	-0.61
FLJ44385	0.038	0.65	*RNA5SP63*	0.041	-0.61
HTR2A-AS1	0.038	0.65	*PARD3B*	0.039	-0.61
KIAA1875	0.039	0.65	*LIMS1*	0.042	-0.61
PLIN4	0.038	0.65	*MRRF*	0.028	-0.61
SLC6A20	0.032	0.65	*DHRSX*	0.037	-0.61
OR14I1	0.034	0.65	*ANKRD13C*	0.035	-0.61
SP2	0.034	0.65	*MIR3173*	0.045	-0.61
STK11	0.039	0.65	*WWTR1*	0.022	-0.61
FAM138A	0.026	0.65	*BAX*	0.047	-0.60

PRR15L	0.041	0.64	*TRIM33*	0.025	-0.60
RCOR2	0.040	0.64	*AMZ2*	0.021	-0.60
SPP1	0.033	0.64	*HELLPAR*	0.049	-0.60
DGAT2	0.042	0.64	*MED13L*	0.020	-0.60
GAL3ST3	0.034	0.64	*COX6A1*	0.049	-0.60
MIR4502	0.035	0.64	*KATNBL1*	0.038	-0.60
FAM87A	0.031	0.64	*SH3D21*	0.044	-0.60
SLC25A1	0.026	0.64	*CHURC1-FNTB*	0.048	-0.60
SDIM1	0.043	0.64	*LETMD1*	0.015	-0.60
PYGM	0.034	0.64	*KCNQ5-IT1*	0.020	-0.59
NOS1	0.041	0.64	*SNRPN*	0.026	-0.59
RASA2-IT1	0.040	0.64	*BTBD6*	0.049	-0.59
MARS2	0.041	0.64	*PPP1R11*	0.050	-0.59
ATP6V0E2-AS1	0.042	0.64	*TLE4*	0.031	-0.59
EHHADH	0.020	0.64	*ITGB3BP*	0.026	-0.59
TRPM2	0.013	0.64	*COX7A2*	0.029	-0.59
IGLV3-1	0.027	0.64	*ENTPD3-AS1*	0.032	-0.58
HS3ST3B1	0.016	0.64	*IRF9*	0.037	-0.58
TAPBPL	0.035	0.64	*TMEM214*	0.044	-0.58
AMY2B	0.044	0.64	*GPATCH8*	0.029	-0.58
OR1A2	0.034	0.64	*HECTD3*	0.029	-0.58
RNA5SP361	0.020	0.64	*SNORA11C*	0.041	-0.58
SHROOM3	0.036	0.64	*ITCH-IT1*	0.048	-0.58
RFPL4B	0.037	0.64	*MIR340*	0.041	-0.58
PRDM14	0.027	0.64	*TMEM183B*	0.025	-0.57
NLGN4X	0.038	0.64	*TMEM30B*	0.043	-0.57
CDC42EP2	0.020	0.64	*UGCG*	0.045	-0.57
BEND3	0.039	0.64	*BAG4*	0.033	-0.57
FLJ16341	0.044	0.64	*FAM57A*	0.027	-0.56
ALOX15B	0.012	0.63	*FGF18*	0.047	-0.56
PUS1	0.039	0.63	*NMT1*	0.020	-0.56
OR1N2	0.028	0.63	*ELOF1*	0.050	-0.56
WT1	0.037	0.63	*GRK4*	0.038	-0.56
MIR3178	0.013	0.63	*EXOC4*	0.044	-0.56
SOSTDC1	0.047	0.63	*STAT3*	0.027	-0.56
STAT4	0.034	0.63	*ZDHHC16*	0.049	-0.55
LGALS3BP	0.034	0.63	*STON1-GTF2A1L*	0.045	-0.55
PPP3R1	0.042	0.63	*POC1B-GALNT4*	0.047	-0.55
AVPR2	0.016	0.63	*CRIPT*	0.046	-0.55
IQSEC3	0.034	0.63	*GNAT2*	0.041	-0.55
RNA5SP486	0.039	0.63	*ZBED6*	0.036	-0.54
BPIFB2	0.030	0.63	*ARFGEF1*	0.035	-0.54
CLEC1A	0.030	0.63	*OSBP*	0.027	-0.53
MOV10L1	0.039	0.63	*SERPINB9*	0.044	-0.52
HOXB9	0.050	0.63	*SYNPO*	0.045	-0.52
DEFB132	0.049	0.63	*AHCY*	0.042	-0.52
PTPRT	0.032	0.63	*TGFBRAP1*	0.032	-0.51

SLFN14	0.048	0.63	*TMEM161B*	0.031	-0.50
LIM2	0.040	0.63	*HSPA5*	0.018	-0.46
TMEM88B	0.037	0.63	*INF2*	0.027	-0.46
ACTRT1	0.047	0.63	*SETD5*	0.048	-0.46
NECAB2	0.016	0.63	*SDHC*	0.036	-0.46
ERVMER34-1	0.047	0.63	*TPSD1*	0.045	-0.45
CLIC5	0.035	0.63	*EMC1*	0.046	-0.43
FCGRT	0.037	0.63	*GNAT3*	0.047	-0.42
FLJ20444	0.036	0.63			
ASTN2	0.043	0.63			
FAM83C-AS1	0.028	0.63			
LEP	0.045	0.63			
MLK7-AS1	0.009	0.63			
FAM25G	0.033	0.63			
PSKH2	0.045	0.62			
NT5DC4	0.028	0.62			
MIR519B	0.014	0.62			
SRL	0.014	0.62			
KRTAP10-4	0.019	0.62			
GNAT1	0.044	0.62			
GUCY2C	0.047	0.62			
SYT15	0.025	0.62			
CAPS2	0.045	0.62			
KRTAP2-1	0.037	0.62			
SLC13A1	0.035	0.62			
VSX1	0.046	0.62			
DPP3	0.043	0.62			
GYS1	0.040	0.62			
RNA5-8SP4	0.026	0.62			
ERVV-1	0.034	0.62			
OR4D9	0.047	0.62			
LMO2	0.042	0.62			
SSTR5-AS1	0.037	0.62			
IL17B	0.043	0.62			
TRAV4	0.034	0.62			
MIR3914-1	0.043	0.62			
SLC22A3	0.049	0.62			
PDGFD	0.033	0.62			
NEIL2	0.034	0.61			
MKRN7P	0.032	0.61			
FCAMR	0.016	0.61			
TTLL11-IT1	0.043	0.61			
TMEM233	0.022	0.61			
CCK	0.037	0.61			
SPACA5	0.044	0.61			
PZP	0.049	0.61			
LGALS7B	0.033	0.61			

HBD	0.017	0.61			
TEX13B	0.042	0.61			
MIR3180-4	0.045	0.61			
MIR551B	0.037	0.61			
GRIN3A	0.049	0.61			
MOS	0.019	0.61			
IGHG1	0.032	0.61			
PON1	0.047	0.61			
OR56A4	0.047	0.61			
HSP90AB2P	0.050	0.60			
MIR3161	0.033	0.60			
GLUD1P2	0.045	0.60			
OR2A4	0.038	0.60			
SPRY1	0.042	0.60			
TRAJ14	0.039	0.60			
VASP	0.047	0.60			
LINC00052	0.025	0.60			
CASQ1	0.030	0.60			
SEMA3G	0.021	0.60			
RBM22P1	0.046	0.60			
MIR4708	0.044	0.60			
ISL1	0.041	0.60			
AQP7	0.048	0.59			
IFNG-AS1	0.035	0.59			
SLC9A7	0.042	0.59			
FGF5	0.022	0.59			
AGAP9	0.039	0.59			
MIR128-2	0.040	0.59			
RNU6-54	0.047	0.59			
ZNF485	0.023	0.59			
PIGL	0.047	0.59			
MIR134	0.044	0.59			
SLC4A1	0.027	0.59			
SLC51B	0.038	0.59			
TMEM229A	0.045	0.59			
GBP6	0.028	0.58			
OPTC	0.037	0.58			
GOLGA8UP	0.023	0.58			
OLFM3	0.047	0.58			
KLK1	0.043	0.58			
RNA5SP338	0.033	0.58			
FIGN	0.050	0.58			
SOX13	0.035	0.58			
NAALADL2-AS2	0.042	0.58			
FFAR4	0.050	0.58			
OSBPL7	0.014	0.57			
CCL13	0.028	0.57			

TSSK1B	0.049	0.57			
LINC00869	0.031	0.57			
ATP7B	0.037	0.57			
FAM19A3	0.048	0.57			
REG3G	0.048	0.56			
LINC00279	0.039	0.56			
GLP1R	0.011	0.56			
PROKR1	0.047	0.56			
TMEM92	0.042	0.55			
MMRN1	0.049	0.55			
FAM92B	0.044	0.55			
CNTD2	0.025	0.55			
FLJ46134	0.040	0.54			
CXCL13	0.044	0.54			
DCXR	0.046	0.54			
LY6G6F	0.022	0.54			
A2MP1	0.040	0.54			
SPANXN5	0.022	0.54			
EFNA3	0.022	0.53			
MIR1295A	0.046	0.53			
KCTD17	0.025	0.53			
MIR96	0.042	0.53			
MIR3119-1	0.029	0.53			
P2RX1	0.048	0.53			
UBE2U	0.045	0.52			
CXorf67	0.039	0.51			
SLC35G3	0.027	0.51			
FLJ42351	0.024	0.51			
ZAN	0.047	0.51			
MIR302E	0.040	0.50			
TACR3	0.025	0.49			
WBSCR16	0.037	0.49			
SORCS3-AS1	0.043	0.49			
CASR	0.033	0.49			
MT1DP	0.046	0.48			
HPCAL4	0.045	0.46			
ARC	0.047	0.45			
MIR4486	0.049	0.44			
SATB2-AS1	0.032	0.43			

*Duplicate, uncharacterized and non-annotated gene transcripts were removed
BMI = body mass index
