# Supplementary material for: RNA Microarray Analysis of Macroscopically Normal Articular Cartilage from Knees Undergoing Partial Medial Meniscectomy: Potential Prediction of the Risk for Developing Osteoarthritis
Source: PLoS One. 2016 May 12;11(5):e0155373. doi: 10.1371/journal.pone.0155373 (PMC4865200; doi:10.1371/journal.pone.0155373)
Supplement: S3 Table — (PDF) [file pone.0155373.s003.pdf]

Supplementary Table 3: Gene transcripts differentially expressed by sex*

Gene symbol	***P value***	***LS mean (females)***	***LS mean (males)***	***Ratio***	***Fold change***
Gene transcripts up-regulated in females					
S100A8	0.032	7.30	5.74	2.95	2.95
CCDC144A	0.024	6.23	5.00	2.35	2.35
CSN1S1	0.048	4.40	3.21	2.28	2.28
MEDAG	0.039	7.33	6.20	2.18	2.18
TREML5P	0.005	4.35	3.35	2.00	2.00
ITGBL1	0.049	9.14	8.22	1.88	1.88
CD68	0.038	9.81	8.90	1.88	1.88
CSNK2A3	0.025	5.38	4.48	1.87	1.87
SPDYE5	0.045	6.13	5.23	1.86	1.86
MIR329-1	0.022	5.48	4.65	1.78	1.78
UST	0.040	6.24	5.47	1.71	1.71
ZDBF2	0.028	6.01	5.25	1.68	1.68
MIR374B	0.047	2.80	2.05	1.67	1.67
USP32P1	0.043	5.16	4.45	1.63	1.63
DSC2	0.023	5.92	5.23	1.62	1.62
RNU7-22P	0.007	4.15	3.47	1.61	1.61
CRYZ	0.049	6.79	6.11	1.59	1.59
MIR1180	0.009	5.08	4.42	1.58	1.58
SNORA36B	0.018	5.43	4.78	1.56	1.56
SNORD121A	0.029	2.43	1.81	1.54	1.54
LINC00709	0.027	3.10	2.49	1.53	1.53
NSUN6	0.041	5.72	5.12	1.52	1.52
YEATS2-AS1	0.026	3.32	2.72	1.52	1.52
DRAM2	0.035	6.22	5.63	1.51	1.51
RASGEF1B	0.043	5.63	5.04	1.50	1.50

**Duplicate, uncharacterized and non-annotated gene transcripts were removed*

Gene symbol	*P value*	*LS mean (females)*	*LS mean (males)*	*Ratio*	*Fold change*
Gene transcripts down-regulated in females					
SLPI	0.013	5.32	6.84	0.35	-2.88
SFRP2	0.041	4.90	6.12	0.43	-2.34
MIR4655	0.010	3.77	4.78	0.49	-2.02
MIR548S	0.005	1.36	2.27	0.53	-1.88
RNU7-49P	0.034	4.68	5.56	0.54	-1.84
CEACAM5	0.028	2.88	3.75	0.55	-1.83
HCG22	0.007	3.90	4.68	0.58	-1.72
MIR4436A	0.050	3.64	4.40	0.59	-1.69
MTRNR2L4	0.050	2.68	3.42	0.60	-1.67
MIR4729	0.042	2.76	3.48	0.61	-1.64
PODXL	0.032	5.44	6.15	0.61	-1.64
THRB-AS1	0.039	3.47	4.18	0.61	-1.63
CYP2C8	0.037	2.97	3.67	0.61	-1.63
DPPA2	0.003	2.22	2.92	0.61	-1.63
RNU6-78P	0.028	2.43	3.13	0.62	-1.62
RNY3P2	0.042	2.48	3.18	0.62	-1.62
LINC00086	0.031	4.17	4.86	0.62	-1.61
OR5D14	0.024	4.03	4.71	0.62	-1.61
MROH9	0.023	2.16	2.85	0.62	-1.61
LINC00280	0.045	3.87	4.55	0.62	-1.60
MIR609	0.044	4.54	5.22	0.63	-1.60
RNA5SP496	0.032	2.43	3.11	0.63	-1.60
FAM27E3	0.020	3.49	4.15	0.63	-1.58
MIR4640	0.012	3.50	4.16	0.63	-1.58
RNA5SP81	0.032	2.07	2.72	0.64	-1.57
XXYLT1-AS2	0.014	3.31	3.96	0.64	-1.57
YME1L1	0.035	3.02	3.66	0.64	-1.56
CYP4Z2P	0.026	1.89	2.53	0.64	-1.56
POM121L2	0.003	3.61	4.24	0.64	-1.56
TRBV7-8	0.011	1.85	2.48	0.64	-1.55
SERPINF2	0.050	3.83	4.45	0.65	-1.53
OR7E2P	0.024	3.99	4.60	0.65	-1.53
RNU6ATAC5P	0.028	5.07	5.67	0.66	-1.52
TXNDC8	0.040	3.35	3.95	0.66	-1.52
GBP7	0.012	2.79	3.40	0.66	-1.52
MIR133B	0.013	4.05	4.65	0.66	-1.52
LINC00410	0.043	3.42	4.00	0.67	-1.50
OR9A2	0.003	2.74	3.32	0.67	-1.50
