# Supplementary material for: RNA Microarray Analysis of Macroscopically Normal Articular Cartilage from Knees Undergoing Partial Medial Meniscectomy: Potential Prediction of the Risk for Developing Osteoarthritis
Source: PLoS One. 2016 May 12;11(5):e0155373. doi: 10.1371/journal.pone.0155373 (PMC4865200; doi:10.1371/journal.pone.0155373)
Supplement: S4 Table — (PDF) [file pone.0155373.s004.pdf]

Supplementary Table 4: Gene transcripts relative intensity values

Gene Symbol	*Mean*	*Maximum*	*Minimum*
RN7SL1	13.06	13.24	12.73
FTSJD2	13.00	13.16	12.55
CYTB	12.77	12.90	12.32
ND2	12.71	12.91	12.26
LOC100652902	12.69	12.84	12.43
SH3KBP1	12.68	12.94	12.20
COX1	12.64	12.76	12.20
ATP13A5	12.63	12.74	12.29
CCDC104	12.62	12.74	12.24
RPL41	12.58	12.66	12.36
TPT1	12.51	12.70	11.96
PTPRO	12.49	12.70	12.06
FN1	12.33	12.68	12.00
TLE1	12.26	12.43	11.93
EEF1A1	12.15	12.30	11.98
RPS11	12.07	12.19	11.81
RPS27	11.96	12.10	11.72
NPIPB3	11.94	12.21	11.65
FKSG49	11.94	12.23	11.58
CDR1	11.90	12.27	11.11
DNAPTP3	11.80	12.07	11.54
TUBA1B	11.77	12.16	11.22
LUM	11.77	12.10	11.48
OTTHUMG00000158412	11.75	11.94	11.54
ND6	11.70	12.15	11.24
PRG4	11.66	12.20	9.43
MALAT1	11.65	11.76	11.35
FTL	11.62	12.17	11.21
RPS2	11.58	11.74	11.47
RPL13AP5	11.48	11.57	11.18
CHAD	11.42	12.10	8.41
FMOD	11.41	11.90	10.87
SNORA48	11.34	11.61	10.92
RPL21	11.26	11.37	11.08
HNRNPA1P10	11.25	11.38	11.16
UBC	11.24	11.53	10.81
RPL27	11.19	11.39	10.99
RPL12	11.17	11.24	10.98
MIR4461	11.14	11.47	10.66
BGN	11.11	11.66	10.34
HTRA1	11.01	11.87	9.65
NEAT1	10.93	11.38	10.23
PRELP	10.86	11.41	10.12
RPS28	10.82	10.97	10.58
HSP90AB1	10.81	10.90	10.70
ASPN	10.77	11.89	9.26
PLA2G2A	10.76	11.47	9.56

H3F3A	10.69	10.86	10.41
EEF1G	10.69	10.94	10.11
C6orf48	10.64	11.02	10.35
UBA52	10.58	10.75	10.08
CTGF	10.57	11.33	9.66
MGP	10.57	11.22	10.09
YBX1	10.56	10.79	10.01
MT1X	10.56	11.89	9.88
NPC2	10.55	10.80	10.12
LAPTM4A	10.51	10.78	10.18
ITM2B	10.51	10.68	10.34
IBSP	10.50	11.50	7.17
NPIPB5	10.50	10.73	10.26
TUBA1A	10.48	11.17	9.41
CYTL1	10.40	11.90	5.91
LINC00657	10.40	10.83	10.10
MIR4442	10.39	11.13	9.67
TMSB10	10.38	10.93	9.42
RPS5	10.37	10.66	9.74
NPIPA1	10.27	10.45	10.04
HBA2	10.27	11.39	8.47
SNORD13	10.26	11.00	8.91
RPL19	10.26	10.48	10.08
RHOA	10.25	10.53	9.63
RPS19	10.24	10.47	9.82
SNORD18C	10.20	10.38	9.72
CD99	10.15	10.69	9.37
VIM-AS1	10.13	10.83	8.93
RPL21P28	10.11	10.34	9.89
HIST2H2AC	10.10	10.40	9.75
HSP90B1	10.06	10.38	9.76
CALR	10.00	10.43	9.33
OGN	10.00	10.74	7.63
OTTHUMG00000171045	9.94	10.29	9.10
RPS15	9.94	10.11	9.59
HNRNPA2B1	9.93	10.14	9.75
HLA-DRA	9.89	11.33	8.53
CFH	9.89	10.85	8.04
COL3A1	9.87	10.79	8.22
NDUFA1	9.86	9.96	9.73
DDX5	9.86	10.09	9.46
PSAP	9.86	10.50	9.40
ANXA2P2	9.84	10.32	9.35
CHI3L1	9.80	11.22	7.47
NBPF9	9.79	10.03	9.41
NPIPA5	9.77	9.93	9.46
SH3BGRL3	9.75	10.09	9.25
RPL26	9.73	9.80	9.59
SCARNA9	9.71	10.02	9.35

COL6A3	9.71	10.75	8.16
EDF1	9.69	9.92	9.45
SOD1	9.68	9.88	9.45
MIR1282	9.67	9.95	9.23
ANGPTL2	9.67	10.49	8.17
NPIPB11	9.66	9.85	9.07
POSTN	9.66	11.80	7.21
CHI3L2	9.66	11.06	5.89
SBDS	9.65	9.90	9.40
PTMS	9.63	9.92	9.39
PCOLCE2	9.62	9.96	9.12
PLOD2	9.61	10.21	8.51
SMG1P1	9.61	9.91	9.05
COL1A2	9.61	10.77	7.59
COMP	9.61	10.46	8.87
CCDC80	9.60	10.28	8.55
RPL11	9.60	9.73	9.34
EDC4	9.59	10.39	8.79
ACAN	9.57	10.63	8.05
COX6A1	9.57	9.87	9.21
FRZB	9.55	10.90	5.84
PCMTD1	9.55	9.81	9.28
ABI3BP	9.53	9.92	8.61
CAPNS1	9.52	9.88	9.20
SNAI2	9.51	9.91	9.19
COX7A1	9.50	9.83	9.13
RPL5	9.49	9.69	9.34
DAD1	9.48	9.96	8.84
RPS13	9.46	9.60	9.22
TNC	9.45	10.84	7.97
IGFBP7	9.43	10.06	8.66
LOC100190986	9.42	9.72	8.89
MIR1244-1	9.42	9.63	9.06
PTMA	9.42	9.63	9.06
PPP1R3C	9.41	9.95	8.40
RPL18A	9.40	9.77	8.68
ENPP1	9.40	9.74	8.50
PLXDC2	9.39	9.79	8.93
COX4I1	9.39	9.72	8.94
CILP	9.38	10.54	7.52
SRSF5	9.36	9.59	9.02
LOC441081	9.36	9.64	8.97
ACTG1	9.35	9.89	8.92
HSPA5	9.34	9.92	8.82
IGLC1	9.32	9.56	9.11
CSTB	9.32	9.74	8.79
MMP3	9.31	11.26	6.22
SPP1	9.30	10.49	8.13
SNRPD2	9.29	9.63	8.81

OST4	9.29	9.67	8.83
C2orf40	9.28	10.10	6.78
CD68	9.28	10.36	7.85
SPARC	9.27	9.54	8.47
DDX17	9.27	9.59	8.64
RPS8	9.26	9.39	9.08
POMP	9.26	9.61	8.76
ATP5B	9.24	9.68	8.51
OTTHUMG00000163389	9.24	9.46	8.85
CRIP1	9.23	10.02	8.11
HNRNPDL	9.22	9.44	8.58
SUMO2	9.22	9.52	8.96
TIMP1	9.21	9.83	8.71
HNRNPA1	9.21	9.37	8.90
A2M	9.20	9.76	7.98
NOTCH2NL	9.20	9.63	8.85
TPM4	9.17	10.06	7.94
IVNS1ABP	9.15	9.62	8.34
RPL36	9.13	9.26	8.90
HLA-A	9.12	9.41	8.71
SERPINA3	9.12	10.55	7.65
SEPT15	9.10	9.53	8.50
NBPF16	9.09	9.37	8.78
LOC100509635	9.09	9.62	8.57
COL12A1	9.09	9.96	7.06
MT1CP	9.09	10.46	8.66
MT1F	9.08	9.73	8.40
SNORD89	9.07	9.59	8.14
EIF1	9.04	9.44	8.64
RMRP	9.04	9.54	8.05
ANKH	9.03	9.51	8.11
SDC2	9.02	9.46	8.41
PRDX5	9.02	9.49	8.46
SERINC3	9.01	9.49	8.38
SPARCL1	9.01	9.88	7.51
TMED2	9.00	9.34	8.56
HSPA8	8.99	9.57	8.36
EEF2	8.98	9.38	8.07
B2M	8.97	9.26	8.70
S100A11	8.97	9.27	8.62
MIR3689A	8.96	10.27	8.32
TUBB2A	8.95	9.81	7.78
SNHG8	8.95	10.03	8.34
ANXA1	8.94	9.47	7.83
LOC100507369	8.93	9.31	8.48
BRK1	8.93	9.35	8.32
ATP5I	8.92	9.28	8.55
DCN	8.91	9.37	8.61
ATP6V0E1	8.90	9.51	8.11

MIR3907	8.90	9.45	8.42
CRISPLD1	8.90	10.35	6.30
CLU	8.89	9.52	8.42
HIST1H2AC	8.89	9.17	8.44
ATP5H	8.89	9.26	8.29
RAB1A	8.85	9.30	8.07
IGHG1	8.85	9.66	8.25
LOC728734	8.84	9.06	8.33
SNORD46	8.84	9.59	8.07
PMP22	8.84	9.30	8.01
TXN	8.84	9.36	8.32
FGFBP2	8.83	9.29	7.87
SLC38A2	8.83	9.16	8.31
RPS3A	8.82	8.92	8.70
CLEC3A	8.81	10.78	5.67
SEC61G	8.80	9.26	8.42
MT2A	8.80	9.25	7.44
KDELR2	8.80	9.39	8.20
FLJ45340	8.80	9.31	8.31
DCDC5	8.80	9.24	8.53
MBNL1	8.79	9.16	8.44
EDIL3	8.78	9.32	8.10
CSDE1	8.78	9.18	7.88
ZFAS1	8.77	9.28	8.23
CRTAC1	8.77	9.83	7.35
VIM	8.76	9.35	7.69
HSBP1	8.74	9.17	8.03
NBPF24	8.74	9.14	8.40
SCARNA7	8.73	9.34	7.65
PRKAR1A	8.73	9.13	8.18
DPT	8.73	10.24	6.96
MIF	8.72	9.52	8.20
SEC31A	8.72	9.22	8.08
TUBB	8.70	9.57	7.71
TOMM7	8.68	8.91	8.50
APP	8.67	8.87	8.47
NBPF11	8.67	9.05	8.37
SRP9	8.67	8.97	8.17
DST	8.66	9.35	7.77
RNA5SP195	8.66	9.06	8.16
TCEB2	8.66	9.04	8.17
ZRANB2	8.66	8.90	8.30
TM9SF2	8.65	9.08	7.99
RAB7A	8.64	8.99	7.99
UGDH	8.63	9.47	7.69
MMP2	8.63	9.93	7.05
EIF3K	8.63	8.91	8.11
GABARAP	8.63	9.01	8.12
OAZ1	8.62	9.11	7.72

WASF2	8.62	9.06	7.65
CST3	8.61	9.13	7.71
EEF1B2	8.61	8.81	8.31
APLP2	8.61	9.12	7.87
IGFBP6	8.61	9.04	7.75
P4HA1	8.61	9.17	8.04
COPB2	8.61	9.14	7.95
WBP5	8.61	9.32	7.70
MXRA5	8.60	10.38	5.85
ITGBL1	8.60	9.65	7.07
TMEM59	8.60	8.90	8.20
TXNIP	8.60	9.55	7.55
PPIB	8.60	9.09	7.78
GTF2IP1	8.59	8.83	8.06
CTA-313A17.5	8.59	9.56	8.15
LOC100272216	8.59	9.18	7.82
PKM	8.58	8.99	7.96
MYL9	8.58	9.13	7.55
FLJ14186	8.58	9.06	8.19
CTSK	8.57	9.57	6.91
SF3B1	8.57	8.78	8.16
SLC25A6	8.57	8.93	7.98
LOC101060684	8.56	9.01	8.01
SRSF11	8.56	8.82	8.06
ERRFI1	8.56	9.23	6.87
FAM106CP	8.56	9.61	7.82
RPL13AP20	8.55	8.78	8.16
WSB1	8.55	8.96	8.25
SPDYE7P	8.54	9.03	8.24
LGALS1	8.54	9.37	7.51
HIF1A	8.54	9.04	7.51
ARF1	8.53	9.06	8.08
RPS21	8.53	8.77	8.41
CD46	8.52	8.89	7.73
ARL6IP5	8.52	8.94	7.85
LOC100996522	8.51	8.91	8.08
RABAC1	8.51	9.36	7.84
ACTR2	8.51	8.92	7.93
CDO1	8.51	9.35	7.43
ELL2	8.50	9.09	7.93
PLP2	8.50	9.16	7.61
MIR2909	8.50	9.58	7.89
COL2A1	8.49	9.64	6.67
APOD	8.49	11.27	4.97
GJA1	8.49	10.11	6.40
TIMP3	8.49	8.95	7.82
LUC7L3	8.49	8.74	8.04
HNRNPH3	8.48	8.62	8.19
NFIX	8.48	8.91	7.91

CALU	8.47	9.71	7.27
IFITM2	8.47	8.84	7.86
EIF4B	8.46	8.69	8.05
EIF4G2	8.46	9.11	7.58
TMEM219	8.46	8.88	8.04
TRAM1	8.46	8.83	8.15
ARPC5	8.45	9.04	7.60
INPP5B	8.45	8.75	8.12
ANXA5	8.45	9.06	7.65
LOC731275	8.44	8.94	7.89
CD55	8.44	9.28	7.63
MSN	8.44	8.97	7.62
EMP3	8.43	9.33	7.38
MIR3689B	8.43	9.83	7.64
NSA2	8.43	8.74	8.13
MRPS21	8.43	8.86	7.86
LRP1	8.41	8.81	7.88
MIR548I1	8.41	9.33	7.72
BNIP3L	8.41	8.81	7.73
SNORD3B-1	8.40	8.99	7.96
CCDC47	8.40	8.83	7.94
SNORD3A	8.40	9.00	7.94
SNAR-E	8.39	9.22	7.76
PRDX1	8.39	8.88	7.53
SPDYE2	8.38	8.87	8.04
GUSBP9	8.38	8.71	7.94
HNRNPU	8.38	8.55	7.75
NCKAP1	8.37	8.79	7.74
LOC100288069	8.37	8.77	7.86
COPZ2	8.36	8.86	7.32
UACA	8.35	8.83	7.81
FSTL1	8.35	9.21	7.63
NOP10	8.34	9.01	7.12
F8A1	8.34	8.85	7.49
EFEMP1	8.34	9.53	7.49
ECM2	8.34	9.15	7.09
LOC100288102	8.34	8.84	7.86
RSU1	8.33	8.63	7.76
NOMO2	8.33	8.76	7.96
IQGAP1	8.32	8.81	7.50
KIF5B	8.32	9.14	7.58
SERPING1	8.31	9.06	7.64
SRSF3	8.31	8.67	7.71
NOMO3	8.31	8.73	7.93
IL13RA1	8.31	8.62	7.86
C17orf89	8.31	8.63	7.90
GOLGA8A	8.31	8.92	7.60
SPDYE2B	8.31	8.80	7.97
TPTE	8.30	8.66	7.75

HP1BP3	8.30	8.68	7.50
PAPSS2	8.30	9.69	7.29
OTTHUMG00000177010	8.29	8.86	7.69
CLIC4	8.29	8.69	7.64
SSR1	8.29	8.71	7.87
RPS16	8.29	8.41	8.10
HADHA	8.29	8.72	7.50
PLS3	8.28	9.13	6.94
CBWD1	8.28	8.83	7.90
SULF2	8.27	8.94	7.13
STT3A	8.26	8.80	7.75
XRCC5	8.26	8.71	7.55
SAP18	8.26	8.54	7.71
ESYT2	8.26	8.91	7.55
FGF2	8.25	8.82	7.78
PJA2	8.25	8.69	7.43
RNA5SP428	8.25	8.48	8.00
CD164	8.25	8.57	7.79
IFITM1	8.25	8.98	7.48
TRPS1	8.24	9.01	7.22
UAP1	8.24	8.84	7.47
HNRNPK	8.24	8.52	7.72
CANX	8.24	8.60	7.81
IL6ST	8.24	8.46	7.88
LUST	8.24	8.55	7.88
TGFBR2	8.23	8.66	7.62
ARL3	8.23	8.66	7.76
RNA5SP312	8.22	8.56	7.69
RNA5SP311	8.21	8.59	7.71
DMTF1	8.21	8.74	7.82
TAX1BP1	8.21	8.43	7.49
COL6A1	8.20	9.15	6.72
TUG1	8.20	8.52	7.77
BOLA2	8.20	8.65	7.89
RBM39	8.20	8.50	7.64
RPL10A	8.20	8.31	8.06
MIR1238	8.20	8.87	7.85
SMA4	8.20	8.62	7.82
VCAN-AS1	8.19	9.13	6.93
LOC100216479	8.19	9.12	7.52
ANXA7	8.19	8.44	7.85
PRRC2C	8.19	8.56	7.55
BHLHE40	8.19	9.18	7.34
UTRN	8.19	8.69	7.47
RN7SK	8.18	8.86	7.74
LUZP6	8.18	8.64	7.51
TUBB4B	8.18	8.35	7.97
LOC729737	8.18	8.55	7.69
OTTHUMG00000165313	8.18	9.13	7.39

RNA5SP310	8.18	8.57	7.83
PSMA7	8.18	8.59	7.69
OGT	8.17	8.50	7.70
ARCN1	8.17	8.76	7.44
UQCRHL	8.17	8.56	7.42
MIR3689F	8.17	8.73	7.54
IGLJ7	8.17	8.90	7.69
CIRBP	8.16	8.62	7.45
CHMP4B	8.16	8.56	7.59
PIGT	8.16	8.69	7.40
SERINC1	8.16	8.67	7.36
CDON	8.16	8.98	6.58
SPDYE8P	8.15	8.85	7.77
ATP5G3	8.15	8.57	7.69
ABI1	8.15	8.69	7.34
BCAT1	8.15	8.88	7.48
ARL6IP1	8.15	8.69	7.47
MIR4732	8.14	8.52	7.81
USO1	8.14	8.50	7.74
CDC42	8.14	8.47	7.63
RGPD8	8.14	8.34	7.88
H3F3B	8.14	8.57	7.52
AGAP9	8.14	8.37	7.81
PNISR	8.13	8.44	7.82
YIPF5	8.13	8.73	7.38
COX8A	8.13	8.59	7.31
GABARAPL2	8.13	8.50	7.61
COL1A1	8.13	9.32	6.02
UBE2K	8.13	8.61	7.52
ECH1	8.12	8.58	7.65
ZC3H11A	8.12	8.61	7.51
NDUFS5	8.12	8.62	7.54
TYROBP	8.11	9.48	6.89
CHCHD2	8.11	8.32	7.60
AKT3	8.11	8.57	7.69
PBX1	8.11	8.62	7.65
NBPF8	8.11	8.46	7.75
WWP2	8.11	8.71	7.24
HNRNPM	8.10	8.34	7.77
RPN2	8.10	8.38	7.54
CAP1	8.10	8.84	7.32
SFT2D2	8.10	8.52	7.42
LOC100499405	8.10	8.30	7.66
METRN	8.09	8.63	7.42
TAF1D	8.09	8.56	7.57
C1orf43	8.09	8.71	7.30
FBXO9	8.08	8.65	7.48
GOLGA8B	8.08	8.67	7.39
NRN1	8.07	8.76	7.55

LMAN1	8.07	9.06	7.09
EMC7	8.07	8.73	7.46
CMPK1	8.07	8.32	7.83
VCAN	8.07	9.07	6.69
PICALM	8.07	8.43	7.51
TOMM6	8.06	8.32	7.71
SLC14A1	8.06	9.16	5.46
OTTHUMG00000169696	8.06	8.49	7.58
IGFBP5	8.06	8.76	6.64
OSMR	8.06	8.61	6.78
RGPD6	8.06	8.28	7.84
SRRM1	8.05	8.30	7.72
GLG1	8.05	8.47	7.38
MIR3689D2	8.05	9.23	7.43
SNORD17	8.05	9.09	6.86
SLAIN2	8.04	8.58	7.59
NPTN	8.04	8.49	7.36
OTTHUMG00000169357	8.04	10.36	5.51
PNRC1	8.03	8.41	7.70
ATF4	8.03	8.34	7.66
IFI30	8.03	10.20	6.14
SFPQ	8.03	8.26	7.61
SPTBN1	8.02	8.50	7.03
DPCR1	8.02	8.67	7.29
PSMA3	8.02	8.33	7.61
GNG5P2	8.01	8.59	7.21
NUCKS1	8.01	8.21	7.72
IFI16	8.01	8.50	7.59
MT1H	8.00	10.58	6.36
LSM14A	8.00	8.37	7.52
LOC101060147	8.00	8.28	7.64
LOC101060211	8.00	8.44	7.79
FXYD5	7.99	8.47	7.45
MIR21	7.99	9.22	6.78
MTPN	7.99	8.66	6.82
COLEC12	7.99	8.81	7.10
LTBP1	7.99	9.31	6.57
MTDH	7.99	8.47	7.50
SNORA24	7.99	8.56	7.59
EID1	7.98	8.15	7.75
BASP1	7.98	9.45	5.97
KHDC1	7.98	8.49	7.46
C19orf53	7.98	8.57	7.37
TAF15	7.98	8.29	7.59
DLX5	7.98	8.63	6.86
PTPN11	7.97	8.61	7.35
MIR520F	7.97	8.78	7.18
PCOLCE	7.97	8.29	7.38
EFCAB14	7.97	8.18	7.57

SNAR-B1	7.97	8.61	7.35
EIF3A	7.97	8.63	7.22
CASC4	7.96	8.28	7.52
ATP5A1	7.96	8.23	7.65
SUPT4H1	7.96	8.38	7.64
DDR2	7.96	8.63	7.42
PKD1P1	7.96	8.25	7.59
S100B	7.96	9.00	5.23
OMD	7.95	8.66	6.38
SRRM2	7.95	8.15	7.53
FNDC3B	7.95	8.53	7.27
CLTC	7.95	8.47	7.38
SGCB	7.95	8.75	6.80
EIF4EBP2	7.95	8.36	7.04
F8A3	7.95	8.28	7.53
MIR1184-1	7.95	8.28	7.53
PDGFRA	7.94	8.68	6.80
PSMB4	7.94	8.24	7.30
PTPRM	7.94	8.37	7.22
SCARNA10	7.94	8.42	7.26
ATP2B4	7.94	8.46	7.23
KRTAP5-7	7.93	8.20	7.69
ROMO1	7.93	8.18	7.47
MGEA5	7.93	8.30	7.28
HNRNPC	7.93	8.15	7.26
BMS1P5	7.93	8.24	7.40
CAMSAP2	7.93	8.31	7.63
HIGD1A	7.93	8.49	7.22
GINM1	7.93	8.41	7.11
LGMN	7.93	9.29	7.04
EPB41L2	7.92	8.51	6.95
EIF3C	7.92	8.36	7.30
AFF4	7.92	8.45	7.28
SMARCA5	7.92	8.23	7.60
USP17L15	7.92	8.83	7.27
CYBB	7.92	9.56	6.15
USP53	7.92	8.57	7.24
YME1L1	7.92	8.22	7.43
FBL	7.91	8.30	7.43
UNC50	7.91	8.22	7.39
CYBRD1	7.91	8.20	7.25
MIR4737	7.91	8.23	7.23
DEFB109P1	7.90	8.36	7.63
GOLGA8O	7.89	8.31	7.40
LOC100129148	7.89	8.06	7.67
BNIP3	7.89	8.52	7.31
MIR1976	7.89	8.42	7.35
TMF1	7.89	8.27	7.34
CHD9	7.89	8.25	7.30

SRSF4	7.89	8.27	7.31
ARL6IP4	7.88	8.19	7.73
GPNMB	7.88	8.68	6.69
ALG12	7.88	8.47	7.37
MIR3180-1	7.88	8.58	7.37
MST4	7.88	9.23	6.16
PCNP	7.88	8.25	7.02
OTTHUMG00000019295	7.87	8.70	6.97
CCDC14	7.87	8.15	7.46
AHNAK	7.87	8.31	7.12
MAGED1	7.87	8.25	6.87
ITGAV	7.87	8.53	7.09
H1F0	7.86	8.21	7.24
LAPTM5	7.86	9.38	6.48
SCARB2	7.86	8.60	6.71
C6orf62	7.85	8.52	7.00
SESTD1	7.85	8.48	7.28
RAPH1	7.85	8.16	7.40
BMS1P1	7.85	8.13	7.41
YWHAQ	7.85	8.25	7.30
KMT2E	7.85	8.12	7.32
NDUFAB1	7.85	8.31	7.45
MIR3619	7.84	8.38	7.53
PCBP2	7.84	8.12	7.31
FBN1	7.84	8.87	6.73
AGAP4	7.84	8.02	7.59
MAR6	7.84	8.17	7.33
LOC101060615	7.84	9.30	7.22
SON	7.84	8.03	7.44
HIST1H1C	7.84	8.73	6.48
COX20	7.83	8.10	7.51
FUBP1	7.83	8.02	7.49
PAR5	7.83	8.27	7.28
ROCK1	7.83	8.48	6.70
REEP3	7.83	8.24	7.21
UQCR10	7.82	8.09	7.47
SERPINF1	7.82	8.48	7.10
AGAP7	7.82	8.15	7.46
DUX4L3	7.82	8.58	7.18
MGC72080	7.81	8.23	7.51
EIF2S2	7.81	8.23	7.28
ARGLU1	7.81	8.06	7.49
GLIPR1	7.81	8.50	6.96
CAPRIN1	7.81	8.18	7.13
SLC39A6	7.80	8.47	6.72
THBS1	7.80	8.77	7.03
TTC3P1	7.80	8.25	7.33
SEC24B	7.80	8.16	7.29
CREBBP	7.80	8.25	7.15

DUX2	7.80	8.47	7.24
SNORD113-3	7.80	9.23	6.57
KCNRG	7.80	8.51	6.78
CNBP	7.80	8.12	7.25
RPL23AP32	7.79	8.49	7.39
PRPF8	7.79	8.15	7.18
GLI3	7.79	8.48	7.01
EIF4A2	7.78	8.07	7.38
GOLIM4	7.78	8.06	7.09
MIR1910	7.78	8.21	7.56
LAMC1	7.78	8.52	6.64
AGAP8	7.78	8.02	7.59
RNU4-5P	7.78	8.38	6.77
LOC100996554	7.77	8.17	7.23
SLC29A1	7.77	8.25	7.16
NAIP	7.77	8.12	7.41
SMOC2	7.77	9.52	6.41
MIR647	7.77	8.71	7.19
LOC100129060	7.77	8.03	7.47
STAT3	7.77	8.27	7.27
COPA	7.77	8.24	7.06
SRP14	7.77	8.01	7.44
ARL2BP	7.77	8.47	6.68
COL11A1	7.76	8.60	7.07
MIR4669	7.76	8.46	7.11
EMP2	7.76	8.13	7.37
STX7	7.76	8.15	7.25
PFN1	7.76	8.14	7.33
GOLGA6L5	7.75	8.17	7.38
ZNF532	7.75	8.43	7.24
USP17L9P	7.75	8.69	7.15
HIF1A-AS2	7.75	8.54	6.83
LOC648987	7.75	7.96	7.45
SERBP1	7.75	7.89	7.54
CTSA	7.75	8.33	7.07
RARS	7.75	8.25	7.22
PTN	7.75	9.69	5.86
ARHGEF12	7.74	8.08	7.12
EIF3L	7.74	7.99	7.44
OTTHUMG00000168327	7.74	8.24	6.94
LIN7C	7.74	8.23	7.15
CTNNB1	7.73	8.30	7.05
ACBD3	7.73	8.20	7.34
PPP3CA	7.73	8.19	7.29
SNORD116-17	7.73	8.15	7.23
FNBP4	7.73	8.09	7.02
GPC6	7.72	8.66	6.79
USP17L6P	7.72	8.73	7.11
PCM1	7.72	8.03	7.24

RHOB	7.72	7.99	7.22
SEC61B	7.71	8.13	7.03
RNF168	7.71	8.04	7.14
FAM114A1	7.71	8.41	6.84
RAD23B	7.71	8.18	7.21
SYNCRIP	7.70	8.17	7.08
SLC40A1	7.70	8.05	7.22
RPN1	7.70	8.20	7.06
MIR548I2	7.70	8.80	7.08
TMEM14A	7.70	8.24	6.87
BHLHE41	7.69	8.14	6.84
CCNI	7.69	8.07	7.16
PTGES3	7.69	8.08	7.03
SDHAP2	7.69	8.24	7.18
HLA-E	7.69	8.08	7.08
PSMB1	7.69	8.12	7.09
C19orf43	7.68	8.29	6.90
SH3GLB1	7.68	8.28	7.18
PDCD4	7.68	8.07	7.09
HSPD1	7.68	7.94	7.32
LRRC37A2	7.67	8.23	7.16
MIR185	7.67	8.25	7.22
RRAGA	7.67	8.03	7.02
PEBP1	7.67	8.10	7.23
HDLBP	7.67	8.03	7.10
PLAGL1	7.67	8.27	7.20
ATP5J2	7.67	8.10	7.23
MT1G	7.67	9.66	6.40
MYOF	7.66	8.46	6.27
WDFY3-AS1	7.66	7.91	7.09
ZCRB1	7.66	7.93	7.02
HEXA	7.66	8.10	7.13
TM9SF3	7.65	8.06	7.09
CD109	7.65	8.75	6.35
DCTN6	7.65	7.99	7.14
LOC100996920	7.65	8.22	6.94
MIR574	7.65	8.06	7.17
BMPR2	7.64	8.10	7.05
NTN4	7.64	8.10	6.71
RAB6A	7.64	8.20	6.95
SMA5	7.64	8.11	7.26
VMP1	7.64	8.18	6.89
CD74	7.64	8.49	7.02
NDUFB1	7.64	7.87	7.33
TMEM47	7.64	8.32	6.42
SNAR-C1	7.63	8.43	6.99
CLINT1	7.63	8.12	6.89
POLR2L	7.63	8.04	6.94
COL6A2	7.63	8.38	6.71

OTTHUMG00000160932	7.63	8.41	6.65
HBA1	7.63	9.54	6.03
GDI2	7.63	8.13	6.89
MGC2752	7.62	8.16	7.20
IQSEC3	7.62	8.06	7.14
LENG9	7.62	7.88	7.26
USP17L5	7.62	8.92	6.88
NKTR	7.61	7.89	6.98
RASA4	7.61	8.02	7.31
UQCRC2	7.61	7.87	7.12
SHC1	7.61	8.15	6.86
JAK1	7.61	7.85	7.14
UBXN4	7.61	7.92	7.27
SEC63	7.60	7.91	7.03
OTTHUMG00000151089	7.60	7.93	6.89
MAP3K8	7.60	8.10	7.18
RGCC	7.60	8.23	6.92
GOLPH3	7.59	7.92	7.31
REEP5	7.59	7.87	7.07
MSMP	7.59	9.95	5.79
NOTCH2	7.59	8.17	7.00
TRAM2	7.59	8.30	6.50
HSPA9	7.59	7.92	6.90
VCAM1	7.58	8.29	6.42
MYADM	7.58	8.28	6.47
MYO1B	7.58	8.18	6.63
SAT1	7.58	7.92	7.27
RPS6	7.58	7.69	7.38
COL14A1	7.58	8.54	6.31
EIF3E	7.58	7.94	7.19
DDX1	7.58	8.01	6.62
RGPD2	7.57	7.90	7.29
HERC2P3	7.57	7.78	7.20
TCF4	7.57	8.18	6.98
PIK3R1	7.57	8.12	6.85
U2SURP	7.57	7.84	6.95
RBP4	7.56	8.61	6.09
CD248	7.56	8.25	6.93
ZNF207	7.56	7.81	7.17
ARF4	7.56	8.41	6.90
MIR1254-1	7.56	8.02	7.10
SAR1A	7.55	8.20	6.97
PIK3C3	7.55	7.96	7.11
ZNF358	7.55	7.91	6.93
ELF1	7.55	8.14	6.48
CSNK1E	7.55	7.82	7.15
PSD3	7.55	8.16	6.96
HIPK3	7.54	7.92	6.95
MRPL33	7.54	7.72	6.86

CHRDL2	7.54	9.55	5.71
ACTR3BP5	7.54	8.57	6.98
MIR1538	7.54	8.05	7.02
CD9	7.54	7.84	7.15
ZNF841	7.53	7.97	7.04
PLAC9	7.53	7.99	6.86
TTC14	7.53	7.75	7.21
FOXC1	7.53	7.97	7.06
C5orf15	7.53	7.88	6.69
NFE2L1	7.53	7.97	6.95
FAM21A	7.53	7.71	7.25
ZFP36	7.52	8.13	6.87
SELT	7.52	7.85	7.10
ILF2	7.52	8.18	6.96
OTTHUMG00000157333	7.52	7.98	7.11
TSPAN2	7.51	8.93	5.31
RAB22A	7.51	8.39	6.46
PSMB6	7.51	7.98	7.14
TNFRSF11B	7.51	8.48	6.42
SRSF6	7.51	7.99	7.06
ARL17A	7.51	8.19	6.85
PGK1	7.51	8.35	6.75
SMARCA1	7.51	7.85	6.90
DDX6	7.51	7.96	6.95
MXRA8	7.51	8.23	6.46
GNL3	7.51	7.98	7.01
NENF	7.50	7.86	6.68
SRP72	7.50	8.19	6.76
CHMP5	7.50	7.79	6.84
GRN	7.50	8.25	6.77
CLK1	7.50	7.75	7.07
CD63	7.50	7.72	7.31
PALMD	7.50	7.94	6.90
VAMP3	7.50	7.95	6.96
TPR	7.50	7.78	6.99
PDCD5	7.50	7.83	7.12
NDFIP1	7.50	7.84	6.92
NARS	7.50	8.00	6.62
HIST2H2AA4	7.49	7.81	6.65
SLC20A1	7.49	8.09	6.13
RNA5SP65	7.49	8.24	6.98
N4BP2L2	7.49	7.72	7.25
SNRNP70	7.49	7.74	6.90
NUDT21	7.49	7.75	7.00
ITIH6	7.49	8.75	5.50
SNHG5	7.49	8.02	7.00
SOX5	7.49	7.96	7.11
GREM1	7.49	9.22	5.26
GTF2A1	7.49	7.97	6.77

CRTAP	7.48	7.90	7.09
CTSD	7.48	8.08	6.88
COL5A2	7.48	8.34	6.06
SPRED1	7.48	7.90	6.59
MDM4	7.48	7.69	7.05
C19orf60	7.48	8.01	6.99
GNG12	7.48	7.91	6.55
DIS3	7.48	7.78	6.77
HIST1H2BK	7.48	7.93	6.92
NDEL1	7.48	8.00	6.74
MIR570	7.47	8.10	6.71
DEFB109P1B	7.47	8.00	7.13
OTTHUMG00000090442	7.47	7.88	7.07
CCDC6	7.47	8.04	6.88
CNOT1	7.47	7.82	6.89
SULF1	7.46	8.15	6.35
TRAPPC2L	7.46	8.06	6.61
ACTR3	7.46	8.30	6.14
HLA-DQA1	7.46	9.09	6.16
TIMP4	7.46	8.77	6.96
SMC5	7.46	8.00	6.69
NNMT	7.46	8.09	6.61
LYZ	7.46	9.94	5.19
PMS2L2	7.46	7.69	7.16
PMS2P5	7.46	7.69	7.16
POM121	7.45	7.84	6.95
SAFB2	7.45	7.66	7.08
AP2B1	7.45	8.10	6.86
DHX15	7.45	7.82	7.02
TMEM50A	7.45	7.93	6.58
CDH11	7.45	8.40	5.98
KRTAP10-6	7.45	8.33	6.88
DAB2	7.45	8.00	6.63
PSMB3	7.45	8.03	6.71
GOLGA6L4	7.44	7.71	7.20
SNORD116-2	7.44	8.04	7.04
SEC24D	7.44	7.95	6.73
ARRDC3	7.44	8.66	6.29
RBMS1	7.44	7.83	6.88
SNORD13P2	7.44	8.01	6.90
PLOD1	7.44	8.28	6.81
PPDPF	7.44	7.93	6.86
MLEC	7.44	7.76	7.05
LOC400682	7.44	7.69	7.22
C1orf54	7.44	8.55	6.75
MAP7D3	7.43	7.73	6.87
DNAJC3	7.43	8.02	7.07
LOC399753	7.43	7.62	7.11
YWHAZ	7.43	7.96	6.53

ZNF638	7.43	7.70	7.20
PCBP1	7.43	7.74	7.08
COX5A	7.43	7.95	6.93
HNRNPUL1	7.43	7.89	6.69
MIR644A	7.43	7.76	6.20
NCL	7.43	7.54	7.13
MEA1	7.42	7.84	6.76
GPR64	7.42	8.14	6.59
CTBS	7.42	7.84	7.02
CDC37	7.42	7.65	6.83
PDCL3P4	7.41	8.03	6.97
OXA1L	7.41	7.65	6.93
DUSP23	7.41	7.75	7.06
VAPA	7.41	8.14	6.86
PAFAH1B1	7.41	7.92	6.60
EIF3D	7.41	7.67	6.75
NFIB	7.41	7.87	6.67
HUWE1	7.41	7.74	6.87
PTBP3	7.41	7.84	6.86
WNK1	7.41	7.82	7.06
FBXO11	7.41	7.76	6.91
CYB5R3	7.40	7.82	6.83
SNORD69	7.40	7.74	7.00
TACC1	7.40	7.79	6.76
CLEC3B	7.40	7.96	5.90
ZC3H13	7.40	7.70	6.89
RARS2	7.40	7.90	6.60
ILK	7.40	7.80	6.49
SCARNA17	7.40	8.03	6.74
ITGB8	7.40	8.13	5.32
CCDC88A	7.40	7.73	6.67
NRD1	7.40	7.65	6.91
CAV1	7.40	7.88	6.65
SNORD116-6	7.39	7.94	6.72
LOC100996709	7.39	8.06	6.96
CBWD5	7.39	7.73	7.11
DEK	7.39	7.80	6.82
MFAP1	7.39	7.79	6.96
LENG8	7.39	7.75	6.98
RAC1	7.39	7.64	7.02
OTTHUMG00000156089	7.39	8.24	6.64
LRLE1	7.38	7.77	6.98
SLC44A2	7.38	8.03	6.23
RASA4B	7.38	8.45	6.73
MIR4533	7.38	8.14	7.10
SCAMP1	7.38	8.16	6.64
IGHJ1	7.38	7.94	7.08
GMFG	7.38	8.08	6.58
GFPT1	7.38	7.97	6.90

ASS1	7.38	7.96	6.96
MBNL2	7.38	7.89	6.56
CXCR7	7.37	8.00	6.38
CASC3	7.37	7.80	6.31
AFF1	7.37	7.82	6.86
HCG8	7.37	8.24	6.61
AK2	7.37	7.62	7.19
KRTAP9-2	7.37	7.90	6.88
GLTSCR2	7.37	7.78	6.83
C1orf123	7.37	7.67	6.86
EP300	7.37	7.82	6.65
HDDC2	7.37	7.65	6.83
PYURF	7.37	7.61	6.96
USP34	7.37	7.72	6.76
ITM2A	7.37	8.44	5.20
ISM1	7.37	8.60	6.52
C14orf1	7.36	7.83	6.80
HIST1H1E	7.36	7.90	6.32
IFI6	7.36	8.09	6.72
HTRA4	7.36	8.49	6.09
GOLGA6L10	7.36	7.47	7.25
KDELR1	7.36	7.93	6.67
MEOX2	7.36	8.45	6.02
MIR1280	7.36	7.87	6.73
NRP1	7.36	8.04	6.25
MACF1	7.36	7.76	6.73
LSMD1	7.36	7.72	7.00
EGR1	7.36	8.07	6.61
RPS18	7.36	7.51	7.09
CBR4	7.35	7.73	6.87
SLTM	7.35	7.65	6.56
NFKBIZ	7.35	8.05	6.65
RAMP2	7.35	8.50	6.18
SYF2	7.35	7.75	6.75
ULK4P2	7.35	7.68	6.48
ANXA4	7.35	7.83	6.54
ANKRD10	7.35	7.70	6.83
KCTD12	7.35	7.92	6.75
CTTN	7.35	7.76	6.71
FAM21B	7.35	7.57	7.02
LOC400927	7.34	8.05	6.57
ANTXR1	7.34	8.66	5.53
HLA-C	7.34	7.67	7.11
TISP43	7.34	8.00	6.75
LOC100506745	7.34	7.60	7.01
PRRC1	7.34	8.00	6.60
MAN1A1	7.34	7.94	6.52
LOC100128364	7.34	8.09	6.66
GADD45GIP1	7.34	7.84	7.05

DDX3X	7.34	8.13	6.40
PHF1	7.34	7.66	7.03
SMC3	7.34	7.75	6.80
HCG25	7.34	7.58	7.19
LOC100508046	7.34	8.14	6.80
AK1	7.34	7.89	6.53
BZW1	7.33	7.85	6.80
FNDC3A	7.33	7.76	6.65
SETD5	7.33	7.60	7.03
MAPRE2	7.33	7.85	6.45
SLK	7.33	7.75	6.54
SPPL2A	7.33	7.78	6.49
MON2	7.33	7.81	7.01
TYW1	7.33	7.67	6.91
TWSG1	7.33	7.87	6.54
CAPN2	7.33	7.97	6.43
GHITM	7.33	7.77	7.01
TFG	7.33	7.80	6.68
ATF2	7.32	7.75	6.73
PHF3	7.32	7.69	6.89
NET1	7.32	7.93	6.55
RRBP1	7.32	7.99	6.46
CRIPAK	7.32	7.66	6.85
PFDN2	7.32	8.12	6.50
USP7	7.32	7.83	6.83
LOC285074	7.32	7.61	6.62
PPIC	7.32	7.63	6.13
DNAJC15	7.32	7.85	6.51
NUPR1	7.32	7.60	7.05
KIFAP3	7.32	7.85	6.54
SRI	7.31	7.54	6.86
TERF2IP	7.31	7.65	6.97
SCXA	7.31	9.42	5.78
EIF3M	7.31	7.69	6.98
PSMC2	7.31	7.85	6.65
TXNDC15	7.31	7.59	6.82
MARCKS	7.31	7.71	6.91
KTN1	7.31	7.56	6.73
TRA2B	7.30	7.77	6.55
ANTXR2	7.30	7.79	6.73
SNORD116-15	7.30	7.63	6.88
GNB2L1	7.30	7.54	7.06
MBOAT1	7.30	7.87	6.48
TMOD3	7.30	7.72	6.86
PSMD7	7.30	7.99	6.48
WASH7P	7.30	7.70	7.14
ATP5O	7.30	7.53	7.06
UQCR11	7.30	7.67	6.75
PAM	7.29	7.68	6.53

SKIL	7.29	7.77	6.55
LOC220729	7.29	7.69	6.47
DRG1	7.29	7.62	6.63
BRD2	7.29	7.52	6.76
PLTP	7.29	7.86	6.20
RAN	7.29	7.73	6.93
NFIC	7.29	7.86	6.64
CSE1L	7.29	7.74	6.65
KMT2C	7.29	7.57	6.70
C1S	7.28	7.61	7.00
CTSL1	7.28	7.97	6.54
SNHG16	7.28	7.98	6.66
BCL6	7.28	7.99	6.77
GOLGB1	7.28	7.50	6.93
ADAM10	7.28	7.74	6.71
PDK1	7.28	8.03	6.16
ACADM	7.28	7.70	6.52
ARHGAP1	7.28	8.29	6.65
TAF7	7.28	7.73	6.71
REREP3	7.28	7.69	6.84
IGKC	7.28	7.67	7.03
HSD17B4	7.27	7.78	6.93
RANBP9	7.27	7.89	6.48
GUSBP3	7.27	7.69	7.01
CCAR1	7.27	7.57	6.77
IQSEC1	7.27	7.58	6.93
LRRC15	7.27	9.22	5.06
LAMA4	7.27	7.89	6.23
KCNMA1	7.27	7.65	6.87
MTRNR2L8	7.27	7.59	6.91
CD59	7.27	7.56	6.76
RBM5	7.27	7.61	6.65
FUS	7.27	7.53	6.89
MYCBP2	7.27	7.59	6.74
MIR593	7.27	7.58	6.88
AHR	7.26	7.96	6.68
CSNK2B	7.26	7.39	7.13
IGIP	7.26	7.71	6.66
MEG3	7.26	8.39	6.48
PRRX1	7.26	8.14	6.28
IK	7.26	7.58	6.83
MIR92B	7.26	7.84	6.91
NIPBL	7.26	7.57	6.77
EXOC1	7.26	7.65	6.64
SPCS1	7.26	7.42	6.91
TBX15	7.26	7.50	6.62
LMBRD1	7.26	7.68	6.45
MDM2	7.26	7.80	6.52
IGFBP4	7.26	7.64	6.94

MIR3689D1	7.26	8.01	6.53
FCER1G	7.25	8.82	5.60
TPM2	7.25	7.68	6.82
ANKRD12	7.25	7.52	6.92
GPBP1	7.25	7.60	6.86
GGNBP2	7.25	7.56	6.78
LOC100506562	7.25	7.89	6.64
ANKRD10-IT1	7.25	8.25	6.47
DNAJC7	7.25	7.60	6.57
ANP32E	7.25	7.85	6.80
ZCCHC6	7.25	7.70	6.42
SOX9	7.25	8.07	6.71
COX6B1	7.25	7.58	6.49
TMEM60	7.25	7.69	6.90
SETX	7.25	7.58	6.61
PNN	7.25	7.39	6.98
SHOC2	7.25	7.76	6.66
ANO6	7.25	8.11	6.29
ZMYM2	7.25	7.62	6.78
PSMB7	7.25	7.71	6.50
ARPC2	7.25	7.76	6.43
ERLEC1	7.24	7.59	6.89
FBXO7	7.24	7.65	6.60
PSMD8	7.24	7.84	6.53
ANPEP	7.24	8.35	6.07
TLK2	7.24	7.56	6.83
RCN2	7.24	7.49	6.66
TAF9B	7.24	7.61	6.63
SEPT2	7.24	7.67	6.75
KDM1A	7.24	7.96	6.49
SYPL1	7.24	7.68	6.54
CNPY2	7.23	7.53	6.86
ANKRD50	7.23	8.12	6.41
TAF13	7.23	8.32	6.29
BTAF1	7.23	7.61	6.55
CBX3	7.23	7.68	6.36
IMPACT	7.23	7.82	6.71
MAN1A2	7.23	7.55	6.66
LOC100127910	7.23	8.19	6.45
COPB1	7.23	7.86	6.55
WIPI1	7.23	8.06	6.47
WISP2	7.23	7.96	6.35
ZBED6	7.23	7.90	6.72
BPTF	7.23	7.58	6.65
TRAPPC1	7.22	7.85	6.30
NDUFB7	7.22	7.66	6.65
MARK3	7.22	7.57	6.55
IL1R1	7.22	7.81	6.54
SCP2	7.22	7.56	6.53

JMJD1C	7.22	7.60	6.71
SNHG9	7.22	7.69	6.49
THOC7	7.22	7.55	6.74
COPS2	7.22	7.67	6.71
AGAP5	7.22	7.55	6.89
TNKS2	7.21	7.54	6.24
FAM21C	7.21	7.61	6.58
ZNF106	7.21	7.60	6.78
CD47	7.21	7.86	6.25
AFTPH	7.21	7.47	6.93
ITCH	7.21	7.80	6.47
RBM6	7.21	7.39	6.62
HNRNPR	7.21	7.55	6.82
ARNT	7.20	7.56	6.80
MYO1D	7.20	8.10	6.53
POLR2J4	7.20	7.79	6.72
HHIPL2	7.20	7.83	6.55
TSPAN6	7.20	8.13	6.49
POU5F1P4	7.20	7.78	6.72
GOLGA6L9	7.20	7.39	7.09
KMT2A	7.20	7.67	6.80
ITFG1	7.20	7.57	6.41
HMGN3	7.20	7.60	6.66
SNAP23	7.20	7.53	6.80
TOP2B	7.20	7.67	6.37
RHOBTB3	7.20	7.88	6.49
POLR2A	7.20	7.40	6.49
STAM2	7.20	7.86	6.65
SUZ12P1	7.20	7.88	6.69
BSG	7.19	7.49	6.66
TIAL1	7.19	7.55	6.67
LOC100133091	7.19	7.64	6.77
CDC42BPA	7.19	7.63	6.65
ESD	7.19	7.70	6.35
GTF2H2B	7.19	7.53	6.27
COPS6	7.19	7.44	6.88
MAFB	7.19	8.46	6.12
GATAD2B	7.19	7.48	6.47
SPAG9	7.19	7.59	6.44
MIR3661	7.19	7.87	6.70
LOC727896	7.19	7.71	6.40
HNRNPAB	7.19	7.67	6.65
HEXB	7.19	7.55	6.26
RNA5SP488	7.19	7.62	6.80
LOC644450	7.18	7.53	6.65
GPS2	7.18	7.53	6.73
OTTHUMG00000133664	7.18	7.45	6.69
NCBP1	7.18	7.76	6.63
PKD2	7.18	7.57	6.69

TGFBI	7.18	8.98	5.30
DDX42	7.18	7.50	6.65
MIR346	7.18	7.68	6.80
MIR3935	7.18	7.60	6.70
RAD50	7.18	7.53	6.61
RSL1D1	7.17	7.48	6.72
SRGN	7.17	8.27	6.49
SCARA3	7.17	7.96	6.04
CXCL12	7.17	8.00	6.59
HILPDA	7.17	8.11	5.81
HTATSF1	7.17	7.46	6.92
STEAP4	7.17	8.45	5.76
SNRPN	7.17	7.49	6.60
USP22	7.17	7.48	6.62
UBL5	7.17	7.72	6.66
RPL17	7.16	7.53	6.77
PARVA	7.16	7.75	6.12
LOC100652999	7.16	7.67	6.54
FAP	7.16	8.70	5.01
SEPW1	7.16	7.74	6.37
HEG1	7.16	7.59	6.23
SS18	7.16	7.55	6.51
OR2L1P	7.16	7.96	6.79
YIPF2	7.16	7.62	6.43
LARS	7.16	7.68	6.68
COX7C	7.16	7.36	6.77
KLC1	7.16	7.66	6.27
HAPLN1	7.16	8.30	4.94
KLF7-IT1	7.16	8.07	6.57
PATL1	7.15	7.57	6.69
RFTN2	7.15	7.64	6.63
GOLGA7	7.15	7.62	6.48
GSTP1	7.15	7.50	6.66
STOM	7.15	7.79	6.59
SMCHD1	7.15	7.44	6.37
RNASEK-C17orf49	7.15	7.40	6.70
SNORD114-30	7.15	8.25	6.47
HIGD2A	7.15	7.87	6.25
ROBO1	7.15	7.48	6.39
AP2S1	7.15	7.91	6.27
ATP2A2	7.15	7.74	6.41
CHD4	7.15	7.41	6.61
SRPX2	7.15	8.24	6.30
KRT18P15	7.15	7.78	6.75
GPI	7.15	8.00	6.39
SOX4	7.14	7.83	6.16
DNAJC10	7.14	7.50	6.45
CTDNEP1	7.14	7.68	6.51
KPNB1	7.14	7.54	6.68

GPER	7.14	7.38	6.53
INTS3	7.14	7.45	6.89
STK39	7.14	7.66	6.48
TRA2A	7.14	7.60	6.59
EIF1AX	7.14	7.84	6.56
OTTHUMG00000014335	7.14	8.10	6.44
CYR61	7.14	7.57	6.46
GALNT1	7.14	7.96	5.83
SNORA22	7.14	7.81	6.52
RCOR3	7.14	7.51	6.80
TCEAL8	7.14	7.63	6.51
SNORA11	7.13	7.69	6.43
CTSS	7.13	8.92	5.71
GLS	7.13	7.56	6.31
MIR29C	7.13	7.58	6.46
ZDHHC9	7.13	7.84	6.69
MTCH2	7.13	7.99	6.30
PPIP5K1	7.13	8.13	6.58
ACO1	7.13	7.55	6.61
GATC	7.13	7.49	6.78
LAMB2	7.13	7.40	6.45
OTTHUMG00000008492	7.13	7.53	6.46
PDE4DIP	7.13	7.33	6.84
SF3B14	7.13	7.61	6.63
ENOSF1	7.13	7.64	6.78
DNM3OS	7.13	7.92	6.09
MKLN1	7.12	7.55	6.41
BDP1	7.12	7.44	6.82
KLF3	7.12	7.61	6.51
SCD	7.12	8.05	6.43
CDC27	7.12	7.48	6.90
GOLGA4	7.12	7.45	6.64
NDUFS6	7.12	7.44	6.56
LCE3B	7.12	7.64	6.60
COL15A1	7.12	7.72	6.11
LY6G5B	7.12	7.27	6.93
PITPNA-AS1	7.12	7.28	6.98
ATP6V1G1	7.12	7.37	6.52
MIR1229	7.12	7.78	6.14
FIP1L1	7.12	7.36	6.73
LAMP1	7.12	7.42	6.59
LOC100287934	7.12	7.49	6.67
OTTHUMG00000169101	7.11	7.52	6.70
MBD2	7.11	7.41	6.58
CCDC144CP	7.11	8.33	6.17
CBWD3	7.11	7.44	6.79
CAMLG	7.11	7.56	6.66
HERC4	7.11	7.53	6.61
KDM2A	7.11	7.46	6.51

BAZ1B	7.11	7.45	6.44
ERP44	7.11	7.49	6.42
CDC73	7.11	7.44	6.42
TMEM131	7.11	7.61	6.60
FKBP10	7.11	7.59	6.60
TIMP2	7.11	7.76	6.23
DDRGK1	7.10	7.59	6.65
ADNP	7.10	7.57	6.36
BUB3	7.10	7.79	6.36
ADAM9	7.10	7.81	6.29
EPS8	7.10	7.76	6.48
TSIX	7.10	10.07	4.71
HSPB1	7.10	7.59	6.52
ROCK2	7.09	7.51	6.29
DICER1	7.09	7.45	6.45
TCP1	7.09	7.67	6.39
MIR4739	7.09	8.34	6.31
NICN1	7.09	7.36	6.82
CSGALNACT2	7.09	7.79	5.91
IPO7	7.09	7.93	6.30
PPP1CC	7.09	7.51	6.32
STARD13-AS	7.09	7.61	6.60
PTGR1	7.09	7.51	6.71
PPFIBP1	7.09	8.10	6.21
ITGA10	7.09	7.71	6.11
STK38L	7.09	7.75	6.55
YWHAG	7.09	7.66	6.70
FNDC1	7.09	8.59	4.82
GOLM1	7.08	7.99	6.44
YAP1	7.08	7.61	6.59
ERC1	7.08	7.32	6.54
DPM1	7.08	7.46	6.60
PRDX6	7.08	7.33	6.84
HERPUD2	7.08	7.43	6.49
KDELC2	7.08	7.48	6.26
CHD2	7.08	7.38	6.69
RPS3	7.08	7.23	6.90
PSMC5	7.08	7.59	6.39
LGALSL	7.08	7.33	6.54
SPECC1	7.08	7.62	6.61
LOC100133130	7.08	7.53	6.61
ZMYM4	7.08	7.39	6.48
DLGAP4	7.08	7.59	6.87
C12orf23	7.08	7.49	6.64
PAPD4	7.08	7.39	6.52
SPIN1	7.08	7.49	6.41
RSL24D1	7.08	7.40	6.68
LOC728323	7.07	7.74	6.36
MORF4L2	7.07	7.64	6.21

THRAP3	7.07	7.40	6.15
BROX	7.07	7.54	6.41
KIAA0040	7.07	7.81	6.57
SRSF7	7.07	7.58	6.09
ATP5C1	7.07	7.43	6.19
PARK7	7.07	7.26	6.80
FGL2	7.07	8.94	5.28
ATRX	7.07	7.45	6.47
CSNK2A2	7.07	7.45	6.28
POLR2B	7.06	7.44	6.48
MEF2A	7.06	7.42	6.49
SLFN5	7.06	7.63	6.65
COMMD4	7.06	7.72	6.62
ESF1	7.06	7.55	6.16
RBMX	7.06	7.40	6.63
RPL29P2	7.06	7.31	6.72
EPRS	7.06	7.50	6.55
ILF3	7.06	7.49	6.34
KHDRBS1	7.06	7.53	6.37
TNPO1	7.06	7.55	6.48
KRIT1	7.05	7.61	6.47
LINC00566	7.05	7.72	6.52
MAPK1IP1L	7.05	7.62	6.61
MATN3	7.05	9.80	5.03
TIPARP	7.05	7.63	6.52
REXO1L1	7.05	8.25	6.45
MIR210	7.05	7.70	6.56
OTTHUMG00000155081	7.05	8.00	6.48
PHIP	7.05	7.36	6.76
LOC100128508	7.05	7.61	6.53
PSME1	7.05	7.66	6.39
SMAD4	7.05	7.57	6.17
FIBIN	7.05	7.81	5.90
LOC100507217	7.05	7.48	6.02
HERPUD1	7.05	7.53	6.28
LIFR	7.05	7.53	6.29
RNPC3	7.05	7.52	6.32
HINT1	7.05	7.20	6.72
SUV420H1	7.05	7.49	6.49
MAMDC2	7.05	8.16	5.36
LASP1	7.05	7.46	6.54
LOC100130285	7.05	7.45	6.79
OTTHUMG00000161861	7.05	7.79	6.34
ZBTB20	7.05	7.53	6.77
KIAA1033	7.04	7.52	6.12
RGL1	7.04	7.56	6.57
OLFML2B	7.04	7.88	5.97
BCLAF1	7.04	7.38	6.33
DHRS7	7.04	7.40	6.41

PMPCB	7.04	7.55	6.24
MIR3155A	7.04	7.36	6.52
TIA1	7.04	7.56	6.30
MIA-RAB4B	7.04	7.41	6.29
REV3L	7.04	7.39	6.57
VSIG4	7.04	8.68	5.08
XPOT	7.04	7.65	6.00
KIAA1377	7.03	7.53	6.50
AUP1	7.03	7.54	6.48
COPG1	7.03	7.47	6.40
KRT16P2	7.03	7.25	6.42
EEA1	7.03	7.92	6.12
SEMA3C	7.03	8.19	5.14
ITPR2	7.03	7.61	6.56
EIF3G	7.03	7.53	6.28
NDUFA2	7.03	7.42	6.60
IER3IP1	7.03	7.78	6.15
HNRNPL	7.03	7.35	6.62
LOC100506990	7.03	7.38	6.63
XRN1	7.03	7.44	6.15
WASL	7.03	7.73	6.22
COG5	7.03	7.35	6.44
IFT20	7.03	7.41	6.44
ACADVL	7.03	7.49	6.17
TRIP12	7.03	7.40	6.40
MIR1250	7.03	7.32	6.59
AP2M1	7.03	7.50	6.69
CCT5	7.03	7.52	6.54
C1orf63	7.02	7.33	6.60
CCT2	7.02	7.75	6.35
TCF7L2	7.02	7.44	6.58
ZNF521	7.02	7.51	6.22
CLMP	7.02	8.03	5.86
LUC7L	7.02	7.32	6.50
FAM222B	7.02	7.31	6.76
RNU1-18P	7.02	7.35	6.57
NDRG1	7.02	7.85	6.47
MIR184	7.02	8.44	6.43
UBL3	7.02	7.93	5.95
GOLGA8R	7.02	7.35	6.58
RASA1	7.02	7.52	6.46
ARL17B	7.02	7.84	6.25
PPIG	7.02	7.22	6.47
FAM86B2	7.02	7.86	6.40
SNRPB2	7.02	7.37	6.67
DDX18	7.01	7.35	6.61
EMP1	7.01	7.58	5.88
ULK4P3	7.01	7.33	6.15
TMED10	7.01	7.62	6.15

EPAS1	7.01	7.64	6.37
MAP4	7.01	7.53	6.30
HERC2P7	7.01	7.53	6.17
TMEM214	7.01	7.37	6.46
DCXR	7.01	7.47	6.53
GOLGA2	7.01	7.31	6.50
TMTC3	7.01	7.74	6.10
NCOR1	7.01	7.32	6.30
PRG2	7.01	7.80	5.59
JUND	7.01	7.40	6.63
VPS4B	7.01	7.47	6.19
UBAP2L	7.01	7.30	6.62
MGA	7.01	7.23	6.68
STAT1	7.01	7.33	6.60
TBL1XR1	7.01	7.60	6.22
UBE3A	7.00	7.46	6.37
MNF1	7.00	7.82	6.07
SMIM19	7.00	7.53	6.21
UBAP2	7.00	7.41	6.69
TMEM30A	7.00	7.70	6.34
LINC00938	7.00	7.16	6.70
PHAX	7.00	7.47	6.25
LOC651337	7.00	7.43	6.63
MRPL41	7.00	7.64	6.34
PPIAL4A	7.00	7.64	5.97
MPC2	7.00	7.50	6.03
CRIM1	7.00	7.77	5.59
SCUBE1	7.00	8.19	4.82
FCHSD2	7.00	7.27	6.65
RCN1	7.00	7.26	6.38
GPBP1L1	6.99	7.29	6.49
TMED4	6.99	7.62	6.29
DERL1	6.99	7.51	6.03
SCARNA2	6.99	7.60	6.21
ITGB1	6.99	7.96	6.05
VPS53	6.99	7.28	6.45
C17orf85	6.99	7.31	6.48
POM121C	6.99	7.33	6.44
OTTHUMG00000030769	6.99	7.72	5.89
TMX1	6.99	7.49	6.41
DARS	6.99	7.44	6.64
ZMYM6NB	6.99	7.42	6.57
PDS5B	6.99	7.44	6.63
BLOC1S6	6.99	7.59	6.33
ACTN4	6.99	7.54	6.32
FAT1	6.99	7.70	6.11
PHC3	6.99	7.34	6.68
MIR663B	6.99	7.53	6.78
NUDC	6.99	7.40	6.65

MIR5047	6.99	7.78	5.95
TMEM89	6.98	7.19	6.64
TXNDC17	6.98	7.77	6.10
SEPN1	6.98	7.17	6.72
MYSM1	6.98	7.31	6.43
NCOA3	6.98	7.44	6.19
MIR421	6.98	7.56	6.33
CYP4V2	6.98	7.37	6.40
NAP1L1	6.98	7.16	6.71
DARC	6.98	7.91	6.00
CDK4	6.98	7.48	6.63
GNG11	6.98	7.99	6.06
KRTAP4-16P	6.98	7.17	6.69
SEC62	6.98	7.28	6.51
MMP9	6.98	8.98	5.21
SP1	6.98	7.39	6.14
TARDBP	6.98	7.36	6.28
CCNL1	6.98	7.25	6.48
LINC00854	6.98	7.15	6.65
ALG11	6.97	7.31	6.49
CAST	6.97	7.31	6.24
SNX9	6.97	7.67	5.76
TCF25	6.97	7.28	6.29
UBE2D3	6.97	7.17	6.67
GSTA4	6.97	7.32	6.54
ZMIZ1	6.97	7.41	6.46
CDH13	6.97	7.98	5.27
PRB1	6.97	7.74	6.40
ZMPSTE24	6.97	7.84	5.91
MIR3622B	6.97	7.42	6.57
ZC3H7A	6.97	7.31	6.23
NDUFV2	6.97	7.37	6.42
PSMB2	6.96	7.37	6.46
ATL3	6.96	7.44	6.33
HGSNAT	6.96	7.45	6.10
SNORA50	6.96	7.41	6.26
DEFB1	6.96	7.89	5.74
SMS	6.96	7.51	6.48
GPR137B	6.96	7.68	6.14
PIK3C2A	6.95	7.30	6.59
ANAPC5	6.95	7.25	6.28
LOC646513	6.95	7.46	6.17
IGKV1-39	6.95	7.96	6.31
LINC00265	6.95	7.24	6.62
LOC100996568	6.95	7.34	6.62
SMARCA2	6.95	7.44	6.17
PDE7B	6.95	8.05	6.10
ADIRF	6.95	7.46	6.48
CDC5L	6.95	7.39	6.30

BRD7	6.95	7.34	6.34
TAPBP	6.95	7.17	6.68
SNORD13P3	6.95	7.64	6.38
C1orf21	6.95	7.55	6.15
EBPL	6.95	7.34	6.41
DYNC1H1	6.95	7.48	6.09
AHSA2	6.95	7.27	6.39
SPTAN1	6.95	7.36	6.13
TRIP11	6.95	7.42	6.17
ATXN2	6.95	7.12	6.67
RNA5SP413	6.95	7.82	6.56
ARFGEF1	6.94	7.26	6.57
TRBV23-1	6.94	7.06	6.52
LARP1	6.94	7.25	6.24
AGFG1	6.94	7.55	6.01
ERBB2IP	6.94	7.52	6.26
B4GALT1	6.94	7.80	6.42
PCGF2	6.94	7.49	6.25
LYVE1	6.94	8.10	4.97
SENP3-EIF4A1	6.94	7.45	6.59
MKI67IP	6.94	7.34	6.42
PI4KAP2	6.94	7.46	6.30
SEC22B	6.94	7.41	6.51
SMARCC1	6.93	7.30	6.36
OR2T3	6.93	7.62	6.45
ANKRD17	6.93	7.29	6.35
OTTHUMG00000169461	6.93	7.50	6.50
MIR449B	6.93	7.30	6.49
GSK3B	6.93	7.58	6.16
BUD31	6.93	7.49	6.09
PPAP2A	6.93	7.62	5.87
ITGA11	6.93	8.36	5.76
SCPEP1	6.93	7.34	6.43
EWSR1	6.93	7.18	6.50
RTN3	6.93	7.22	6.37
DNAJA2	6.93	7.39	6.28
USP48	6.93	7.34	6.31
SMAD2	6.93	7.27	6.36
RNF38	6.93	7.19	6.64
MIR606	6.93	7.40	6.16
SERPINB6	6.93	7.24	6.48
FUCA2	6.93	7.42	6.30
MAP4K4	6.92	7.53	6.03
CNPY3	6.92	7.26	6.63
FBXL20	6.92	7.38	6.19
GOLGA8I	6.92	7.19	6.51
XPO1	6.92	7.36	6.36
SEC24A	6.92	7.65	6.38
ARF6	6.92	7.55	6.18

OTTHUMG00000161858	6.92	7.95	6.34
PPP6R3	6.92	7.29	6.31
SH3D19	6.92	7.55	5.84
PARN	6.92	7.34	6.30
OR2A20P	6.92	7.46	6.54
HELZ	6.92	7.25	6.48
MAF	6.92	7.47	6.14
B4GALT2	6.92	7.45	6.32
DDX46	6.92	7.30	6.57
SNORD116-3	6.92	7.49	5.98
UBR4	6.92	7.34	6.32
ARID4B	6.92	7.11	6.44
ENAH	6.92	7.58	6.25
PSMD1	6.92	7.77	5.92
CAMK1D	6.92	7.42	6.10
BAZ2B	6.92	7.26	6.30
MRPL35	6.91	7.52	6.41
SERTAD4	6.91	7.86	5.47
MAGI2-AS3	6.91	7.12	6.60
RBM26	6.91	7.17	6.49
PDGFRL	6.91	7.80	5.85
PCNXL4	6.91	7.53	6.23
TTC17	6.91	7.19	6.66
SND1	6.91	7.33	6.55
HNRNPD	6.91	7.10	6.50
ATF6	6.91	7.31	6.44
KLHDC10	6.91	7.34	6.30
DNAJC5	6.91	7.10	6.69
ARPP19	6.91	7.58	5.72
SF1	6.91	7.30	6.58
HIVEP2	6.91	8.02	5.76
PLEKHA1	6.91	7.53	6.02
NEMF	6.91	7.26	6.41
STX8	6.91	7.34	6.21
LPAR1	6.91	7.15	6.56
ASAP1	6.91	7.44	6.05
FGFR2	6.91	7.83	6.18
GNB1	6.91	7.28	6.48
PHF10	6.91	7.40	5.98
NUFIP2	6.91	7.53	5.86
SMIM14	6.91	7.37	6.00
LRCH3	6.91	7.23	6.28
AIMP1	6.91	7.34	6.44
SNORA70G	6.91	7.84	5.06
DOCK11	6.91	7.45	5.86
APOOL	6.91	7.19	6.56
TPRG1	6.91	7.51	6.21
TMEM45A	6.91	7.65	6.47
MUC22	6.91	7.63	6.46

NAA50	6.90	7.46	6.08
TPM1	6.90	7.83	6.06
PARP4	6.90	7.61	5.75
XYLT1	6.90	7.58	6.45
SMYD3-IT1	6.90	8.28	5.91
RIOK3	6.90	7.28	6.27
SF3B5	6.90	7.35	6.62
TGFBR1	6.90	7.83	5.97
ASAP2	6.90	7.63	5.95
SF3A3	6.90	7.19	6.33
CSTF3	6.90	7.26	6.15
SNX27	6.90	7.25	6.18
NID2	6.90	7.76	5.87
CLK4	6.90	7.19	6.51
SRSF1	6.90	7.19	6.62
SDCCAG8	6.90	7.23	6.61
SPINT2	6.90	7.85	5.42
RPS23	6.90	7.12	6.42
CYP2A7P1	6.90	7.93	6.28
MAGED2	6.90	7.42	6.17
TULP3	6.90	7.33	5.95
XPO6	6.90	7.52	6.13
MMP14	6.90	8.47	5.43
STARD7	6.90	7.57	6.33
MMP13	6.90	8.70	3.31
DYM	6.89	7.36	6.31
TRIQK	6.89	7.21	6.33
TK2	6.89	7.19	6.45
SNORD116-8	6.89	7.50	5.83
ANKRD28	6.89	7.69	5.90
SNRNP200	6.89	7.20	6.42
GTF3A	6.89	7.40	6.42
DERL2	6.89	7.55	6.24
FRY	6.89	7.86	5.72
TNFAIP6	6.89	9.08	4.60
CEP350	6.89	7.34	6.45
PBRM1	6.89	7.37	6.28
EIF2A	6.89	7.35	6.36
CBFB	6.89	7.27	6.10
WDR83OS	6.89	7.32	5.77
SNORA33	6.89	7.36	6.53
LOC100129502	6.89	7.30	5.97
GATAD1	6.89	7.14	6.60
KRTAP5-1	6.89	7.34	6.51
XIST	6.89	10.68	3.34
AMOTL1	6.89	7.15	6.42
TRPM7	6.88	7.39	6.27
ADIPOR1	6.88	7.27	6.39
RNA5SP370	6.88	7.34	6.39

TRPC1	6.88	7.51	6.29
EPS15	6.88	7.40	6.09
GOPC	6.88	7.22	6.26
SDC4	6.88	7.48	6.41
ATP6AP2	6.88	7.76	5.90
RAB11A	6.88	7.33	6.20
ZC3H15	6.88	7.11	6.39
TSG101	6.88	7.28	6.39
LOC100287704	6.88	7.08	6.57
RBP5	6.88	7.36	6.63
GPX4	6.88	7.11	6.47
SCAF11	6.88	7.19	6.47
NMD3	6.88	7.33	6.00
FTX	6.88	7.21	6.17
OTTHUMG00000156440	6.88	7.79	6.13
RNA5SP161	6.88	7.72	6.36
POGZ	6.88	7.23	6.41
TCEA3	6.88	7.24	6.49
MIA3	6.88	7.30	6.06
NCOA1	6.88	7.10	6.49
SNX14	6.88	7.35	5.95
TRAPPC6B	6.87	7.29	6.13
MED13L	6.87	7.47	6.32
MIR3650	6.87	7.43	6.31
LTBP2	6.87	7.83	6.26
SNORA8	6.87	7.43	6.36
NAMPT	6.87	7.73	5.57
EIF3J	6.87	7.14	6.45
OTTHUMG00000170985	6.87	7.43	5.86
SAMHD1	6.87	8.27	6.00
MED13	6.87	7.53	6.05
MIR433	6.87	7.17	6.68
SNX6	6.87	7.20	6.32
TMEM43	6.87	7.75	5.67
CBX5	6.87	7.26	6.30
THBS4	6.87	7.38	5.55
OTTHUMG00000161852	6.87	7.96	6.29
IMMT	6.87	7.37	6.16
PRDX2	6.87	7.41	6.35
MAPRE1	6.87	7.46	5.94
ETS2	6.86	7.53	6.30
ARFGAP3	6.86	7.62	6.17
HIST1H2BH	6.86	7.40	6.37
TRIM33	6.86	7.26	6.29
ATP6V1F	6.86	7.16	6.26
LONP2	6.86	7.08	6.44
HADHB	6.86	7.36	5.88
RANBP2	6.86	7.26	6.39
VDAC1	6.86	7.36	6.33

PPWD1	6.86	7.17	6.53
THOC2	6.86	7.28	5.89
WDFY3	6.86	7.31	6.36
STRN3	6.86	7.34	6.23
BMP2K	6.86	7.76	5.85
KLHDC1	6.86	7.46	6.48
PSMB5	6.86	7.17	6.48
ELK3	6.86	7.31	6.15
LITAF	6.86	7.15	6.57
CBLB	6.86	7.21	6.33
SLC16A4	6.86	7.43	6.44
PSIP1	6.86	7.09	6.37
ALDOA	6.86	7.38	6.37
ZNF280D	6.86	7.14	6.44
SLC39A7	6.86	7.31	6.53
ATP2B1	6.86	7.71	6.39
ODC1	6.85	7.17	6.39
TMEM165	6.85	7.58	6.16
CHIC2	6.85	7.50	6.15
PSMD12	6.85	7.36	6.03
REXO1L2P	6.85	7.65	6.26
PRSS23	6.85	7.55	6.30
MPP6	6.85	7.31	6.16
MSL1	6.85	7.08	6.53
DCTN5	6.85	7.34	6.26
HOOK3	6.85	7.39	5.93
MIS12	6.85	7.11	6.39
SNTB2	6.85	7.22	6.21
GBF1	6.85	7.35	6.46
RCC2	6.85	7.19	6.57
ALDOC	6.85	7.26	6.58
NOP58	6.85	7.11	6.46
LOC100132705	6.85	8.29	5.61
VPS39	6.85	7.23	5.90
IRF2BP2	6.85	7.40	6.12
LEPRE1	6.85	7.56	6.04
NRAS	6.84	7.93	5.43
HSPA1B	6.84	7.33	6.51
RPL36AL	6.84	7.13	6.36
WASH1	6.84	7.12	6.67
EVI5	6.84	7.33	6.21
SLC25A37	6.84	7.42	6.31
WHSC1L1	6.84	7.16	6.43
ABI2	6.84	7.26	5.92
C1QC	6.84	7.99	5.81
ASPH	6.84	7.21	6.56
MIR339	6.84	7.24	6.19
LMAN2	6.84	7.23	6.22
PARG	6.84	7.40	6.52

OTTHUMG00000021966	6.84	7.36	6.55
TMEM14E	6.84	7.57	6.38
ATP11B	6.84	7.50	6.22
ANGPTL7	6.84	9.14	4.71
CLCC1	6.84	7.21	6.27
MAP1B	6.84	7.39	6.23
VCL	6.84	7.67	6.11
ERCC1	6.83	7.24	6.45
SNRPA	6.83	7.19	6.30
GLT8D2	6.83	7.47	5.81
NT5E	6.83	8.13	5.16
OTTHUMG00000170174	6.83	7.52	6.38
MIR342	6.83	7.36	6.31
C7orf55-LUC7L2	6.83	7.22	6.57
SEPT9	6.83	7.16	6.59
TIPRL	6.83	7.34	5.99
FCGR1B	6.83	7.28	6.40
PDCD6IP	6.83	7.29	6.13
CD163	6.83	8.07	5.22
CLSTN1	6.83	7.49	6.21
SLC25A3	6.83	7.23	6.21
FAM46A	6.83	7.13	6.46
CMAHP	6.83	7.29	6.06
EXOSC10	6.83	7.11	6.41
FERMT2	6.83	7.61	6.31
TMEM116	6.83	7.10	6.60
SMG1	6.83	7.13	6.19
CYSTM1	6.83	7.30	6.37
NDUFA11	6.83	7.11	6.52
LECT1	6.82	8.00	5.05
NFKBIA	6.82	7.41	6.42
PCBD2	6.82	7.09	6.68
LOC100506748	6.82	7.61	5.69
LOC100289637	6.82	7.62	6.55
WLS	6.82	7.31	5.94
PSMA2	6.82	7.46	6.20
NDUFA7	6.82	7.06	6.45
ABR	6.82	7.19	6.08
HERC2P9	6.82	7.13	6.06
VPS36	6.82	7.13	6.27
KCTD3	6.82	7.24	6.03
PTPLAD1	6.82	7.42	5.61
ATRAID	6.82	7.07	6.57
OTTHUMG00000013474	6.82	7.29	6.37
PCYOX1	6.82	7.12	6.50
PTTG1IP	6.82	7.55	5.79
ABCA5	6.82	7.37	5.59
DLG1	6.81	7.86	5.85
BIVM-ERCC5	6.81	7.12	6.27

NR3C1	6.81	7.25	6.29
DEGS1	6.81	7.32	6.18
RAB14	6.81	7.27	6.27
STYX	6.81	7.32	6.07
GRSF1	6.81	7.22	6.22
RAB1B	6.81	7.22	6.25
MN1	6.81	7.39	6.35
OSBPL8	6.81	7.40	5.96
ORMDL2	6.81	7.51	6.34
FTO	6.81	7.12	6.33
STAG3L3	6.81	7.16	6.62
PGAM4	6.81	7.20	6.32
CSNK1G3	6.81	7.25	6.08
SNAR-G1	6.81	7.42	6.50
OTTHUMG00000177424	6.81	7.47	6.23
VAMP8	6.81	7.93	6.08
CD44	6.81	7.60	5.43
ATM	6.81	7.21	6.33
PITPNA	6.81	7.25	6.17
CAPN7	6.81	7.26	6.41
PHACTR4	6.81	7.01	6.56
WHAMMP1	6.80	7.20	6.29
SOD3	6.80	7.79	6.12
SLC38A6	6.80	7.40	5.81
CCDC117	6.80	7.13	6.45
UBQLN1	6.80	7.25	6.27
SHFM1	6.80	6.90	6.66
OPTN	6.80	7.33	6.21
ANXA6	6.80	7.14	6.28
BIRC6	6.80	7.18	6.25
GTF2IRD2B	6.80	7.39	6.41
MFGE8	6.80	7.90	5.86
AHCYL1	6.80	7.17	6.36
UBA6	6.80	7.42	6.21
KRR1	6.80	7.15	6.44
LEO1	6.80	7.38	5.86
TRPV4	6.80	7.89	6.00
SNX29	6.80	7.25	6.25
PHPT1	6.80	7.11	6.05
ZC3H6	6.80	7.19	6.52
HLA-L	6.80	7.15	6.60
CYB5B	6.80	7.16	6.05
UBE2B	6.80	7.49	6.12
LRRFIP1	6.80	7.70	6.14
DPYSL2	6.80	7.38	5.68
PDLIM5	6.80	7.18	6.09
ANG	6.80	7.39	6.40
PGM5-AS1	6.80	7.74	6.17
NBPF15	6.80	7.14	6.40

CHD3	6.79	7.17	6.29
RC3H1	6.79	7.24	6.24
TTC37	6.79	7.41	5.86
OAT	6.79	7.23	6.05
RBM14	6.79	7.02	6.44
IGHV4-31	6.79	7.13	6.42
CEMP1	6.79	7.19	6.44
CRLS1	6.79	7.26	6.16
MIR4441	6.79	7.57	6.12
EMC3	6.79	7.16	6.21
RIN2	6.79	7.67	5.67
DAZAP2	6.79	7.19	6.19
PIN1	6.79	7.15	6.53
CRIPT	6.79	7.20	6.40
RB1CC1	6.79	7.20	5.94
ITGA6	6.79	7.34	5.44
PKN2	6.79	7.13	6.18
SERPINB1	6.79	7.15	6.17
RPL37	6.79	7.13	6.42
SAFB	6.79	6.96	6.43
SENP6	6.79	7.05	6.32
VEGFA	6.79	8.17	5.83
MIR4516	6.78	7.32	6.49
FER	6.78	7.56	6.08
URI1	6.78	7.24	6.11
IBTK	6.78	7.20	6.47
ATG3	6.78	7.21	6.28
FAM192A	6.78	7.20	6.09
LTV1	6.78	7.31	6.06
CEP95	6.78	7.20	6.31
OXT	6.78	7.18	6.51
GAGE12G	6.78	7.26	6.08
PPP3CB	6.78	7.16	6.25
NDUFB6	6.78	7.47	5.89
NPHP3-ACAD11	6.78	7.35	6.29
TOR1AIP1	6.78	7.06	6.39
MT4	6.78	7.20	6.51
SIK2	6.78	7.30	6.11
AIF1	6.78	8.02	5.80
VDAC3	6.78	7.32	6.14
CDC123	6.78	7.20	6.20
TBC1D3H	6.78	7.02	6.55
SBSPON	6.78	8.13	5.76
LOC541473	6.78	7.39	6.08
CUL1	6.78	7.17	6.01
MIR941-1	6.78	7.12	6.24
TNXB	6.78	7.45	5.92
ARID1B	6.78	6.93	6.51
PDLIM1	6.78	7.12	6.21

WTAP	6.78	7.09	6.22
CRISPLD2	6.78	8.53	5.44
FLOT1	6.77	7.09	6.36
EIF5B	6.77	7.30	6.00
MIR127	6.77	7.21	6.37
HLA-DPA1	6.77	7.77	5.94
OS9	6.77	7.09	6.49
ZCCHC7	6.77	7.03	6.39
CHMP2A	6.77	7.00	6.28
SNRNP27	6.77	7.19	6.32
RYK	6.77	7.18	6.30
DNAJA1	6.77	7.22	6.07
DDOST	6.77	7.16	6.09
RNF24	6.77	7.22	6.14
MIR1302-11	6.77	7.71	6.26
VIT	6.77	8.19	4.80
NAV1	6.77	7.50	6.09
LINC00686	6.77	7.03	6.46
CREB3L2	6.77	7.37	6.43
DPP4	6.77	8.11	4.94
AIMP2	6.76	7.16	6.21
RPL8	6.76	6.91	6.59
OTTHUMG00000016713	6.76	7.29	6.32
NDUFB11	6.76	7.30	6.36
SNAR-D	6.76	7.54	6.19
ARHGDIG	6.76	7.08	6.36
ZMYM6	6.76	7.08	6.19
LINC00493	6.76	7.34	6.26
MIR4519	6.76	7.40	6.46
TIMMDC1	6.76	7.04	6.23
NOMO1	6.76	7.13	5.98
NFAT5	6.76	7.06	6.41
SPTSSA	6.76	7.34	6.13
FAM138C	6.76	7.40	6.42
LRIG3	6.76	7.20	6.01
NSRP1	6.76	7.09	6.33
ARIH1	6.76	7.09	6.25
UHRF1BP1L	6.76	7.31	6.14
FAM103A1	6.76	7.29	6.06
SUPT16H	6.76	7.19	6.13
CNN3	6.76	7.17	6.33
PFDN5	6.76	7.02	6.46
PIGK	6.76	7.37	6.26
EIF4G3	6.76	7.03	6.14
ZDHHC6	6.76	7.07	6.23
S100A4	6.76	7.39	6.22
AMFR	6.76	7.32	5.81
TGFB2	6.76	7.64	5.41
JUNB	6.75	7.58	5.94

ATXN2L	6.75	7.06	6.41
LOX	6.75	7.96	5.67
TUFM	6.75	7.08	6.26
SEC61A1	6.75	7.52	6.17
AKAP9	6.75	7.16	6.17
MIR103B2	6.75	7.18	5.99
VCP	6.75	7.14	6.28
CORO1C	6.75	7.49	5.79
MAPK1	6.75	7.20	5.95
DHX36	6.75	7.10	6.13
PHB2	6.75	7.21	6.35
ZNF250	6.75	7.24	6.43
AEBP1	6.75	7.54	6.10
PYCARD	6.75	7.25	6.07
AKAP13	6.75	7.26	5.94
UBR2	6.75	7.10	6.10
LRP6	6.75	7.16	6.20
SPDYE1	6.75	7.14	6.20
ZFR	6.75	7.22	6.16
LIMCH1	6.75	7.19	6.08
CHMP1B	6.75	7.26	5.76
DCAF16	6.74	6.99	6.28
PHACTR2	6.74	7.07	6.03
MYL4	6.74	7.57	5.95
AMZ2	6.74	7.09	6.12
CENPC1	6.74	7.38	6.24
MIR1202	6.74	7.26	6.44
TMEM126B	6.74	7.12	5.82
KCNA6	6.74	7.49	6.22
SNORA42	6.74	7.32	6.11
SUMO3	6.74	7.06	6.50
CCSER2	6.74	7.06	6.32
LOC647264	6.74	7.52	6.03
RAB31	6.74	7.89	5.35
KDM3A	6.74	7.32	6.33
LNPEP	6.74	7.27	6.13
TXNDC11	6.74	7.05	6.19
DNAJB11	6.73	7.38	6.17
ABHD2	6.73	7.94	5.63
NAA35	6.73	6.98	6.09
SEPT11	6.73	7.38	5.31
KLK11	6.73	7.19	6.17
UFL1	6.73	7.02	6.22
ARPC1A	6.73	7.08	6.21
TMEM230	6.73	7.07	6.23
GBP2	6.73	7.67	6.38
SWAP70	6.73	7.07	6.30
HECTD2	6.73	7.27	6.24
PAFAH1B2	6.73	7.01	6.40

IST1	6.73	7.08	5.90
CTF1	6.73	7.09	6.47
LOC654342	6.73	7.85	5.72
LOC100506735	6.73	7.18	6.35
TNXA	6.73	7.38	5.36
KLF10	6.73	7.48	5.84
TENC1	6.73	7.20	6.23
LOC440300	6.73	7.34	6.07
ZNF25	6.73	7.33	6.18
COL11A2	6.72	7.19	5.82
MRPS16	6.72	7.08	6.35
ARPC4	6.72	6.95	6.02
DDAH2	6.72	7.13	6.40
MIB1	6.72	7.29	5.94
TMEM147	6.72	6.93	6.36
SIPA1L1	6.72	7.21	6.04
SLC17A5	6.72	7.51	5.69
KIF13B	6.72	7.33	5.50
FGD5-AS1	6.72	7.06	6.21
XAF1	6.72	7.65	6.31
RBM12	6.72	7.11	5.97
ADAM17	6.72	7.31	5.86
RBM25	6.72	7.00	6.11
TOP1	6.72	7.05	5.87
PTPN12	6.72	7.12	5.86
C1QBP	6.72	7.11	6.14
FXR1	6.72	7.13	6.23
SNORD114-19	6.72	8.24	4.60
KDM5B	6.72	7.34	6.20
NIF3L1	6.72	7.24	6.19
HERC1	6.72	6.99	6.21
EHD2	6.72	7.41	5.99
EBF1	6.72	7.49	5.74
TTC1	6.72	7.23	5.83
SSR3	6.72	7.25	6.08
WAC	6.72	7.09	6.36
AP1G1	6.72	7.16	5.94
RBM4B	6.72	6.94	6.10
SRSF2	6.72	7.10	5.98
ALOX5AP	6.72	7.69	5.62
JAK2	6.71	7.07	6.22
ANKHD1-EIF4EBP3	6.71	7.03	6.23
FZD8	6.71	7.31	6.12
KLF6	6.71	7.37	6.00
MIR3670-1	6.71	7.18	6.20
DCAF6	6.71	7.06	6.36
PDS5A	6.71	7.16	6.12
PRDX3	6.71	7.13	6.11
QKI	6.71	7.04	5.89

MAP4K5	6.71	7.29	6.24
PARP6	6.71	7.25	5.89
DSTN	6.71	6.91	6.28
RABGAP1	6.71	7.15	6.00
TMLHE	6.71	7.07	5.88
AQP1	6.71	7.55	5.67
MIR661	6.71	7.51	6.37
PLXDC1	6.71	7.47	6.02
PXDN	6.71	7.74	5.62
GOLGA8G	6.71	7.02	6.39
IGHV3-13	6.71	7.75	6.24
ISM1-AS1	6.71	7.92	5.48
MIR3676	6.70	7.03	6.23
SLCO2A1	6.70	7.89	5.12
FOXO1	6.70	7.05	6.25
SERPINE1	6.70	8.43	5.31
SDHAP1	6.70	7.10	6.15
INE2	6.70	7.74	5.16
GAGE12J	6.70	7.38	6.04
KIAA0430	6.70	7.14	6.19
MRPS28	6.70	7.03	6.07
POLR2J2	6.70	7.23	6.05
RPS15A	6.70	7.20	6.00
ZEB1	6.70	7.47	5.85
ADAMTS2	6.70	7.80	5.30
LZIC	6.70	7.17	6.13
TMEM57	6.70	7.22	5.91
ATP6AP1	6.70	7.00	6.44
TRIM44	6.70	7.04	6.25
RABEP1	6.70	7.09	6.28
S100A10	6.70	7.57	5.99
SCAMP2	6.70	7.20	6.23
REXO2	6.70	7.27	6.32
HCFC1R1	6.70	7.17	6.26
NEK1	6.70	7.03	6.45
KIAA0100	6.70	7.09	6.18
FLJ39739	6.70	6.91	6.40
WASH5P	6.70	7.06	6.16
NMT1	6.70	7.07	6.21
TMEM14C	6.70	7.01	6.36
ASF1A	6.70	7.18	6.11
FAM99B	6.69	7.07	6.40
ATP5D	6.69	7.02	6.20
C14orf166	6.69	7.33	6.00
ELP3	6.69	6.90	6.45
ENG	6.69	7.15	6.25
MIR548AL	6.69	7.37	6.08
NEO1	6.69	7.17	6.09
MIR3143	6.69	6.96	6.44

ENO1	6.69	7.89	5.88
ERG	6.69	7.11	5.98
RBBP7	6.69	7.11	6.15
OTTHUMG00000168788	6.69	7.39	5.57
RNF13	6.69	7.03	6.17
COA3	6.69	7.12	5.93
COLGALT2	6.69	7.74	5.63
TIMM23	6.69	7.28	6.18
GNS	6.69	7.13	6.02
MYL6	6.69	6.89	6.29
PACSIN2	6.69	7.06	6.28
ENPP2	6.69	7.31	6.12
PITRM1	6.69	7.14	6.12
OTTHUMG00000161847	6.69	7.74	6.20
MPC1	6.68	6.95	6.44
HSPA4	6.68	7.10	5.98
SRGAP2	6.68	7.19	6.08
LOC100134868	6.68	7.23	6.14
UPF3A	6.68	7.12	6.33
RNA5SP199	6.68	7.08	6.32
JKAMP	6.68	7.03	6.18
MIR4518	6.68	7.45	5.99
SNAR-I	6.68	7.22	6.18
EZR	6.68	7.55	5.41
GABRA4	6.68	8.27	5.86
NBPF12	6.68	7.01	6.06
KRTAP10-9	6.68	7.01	6.32
NF1	6.68	7.07	6.11
FNBP1	6.68	7.08	5.79
CDK5RAP3	6.68	7.01	6.29
SIRPA	6.68	7.15	6.25
MPDZ	6.68	7.07	6.32
ACLY	6.68	7.44	5.78
UBXN6	6.67	7.03	6.05
MIR3150A	6.67	7.23	6.25
UBA1	6.67	7.15	6.20
HNMT	6.67	7.11	6.14
NPIPB6	6.67	7.10	6.11
CTNND1	6.67	6.91	6.08
ORMDL1	6.67	7.12	6.00
NDUFA13	6.67	7.25	6.31
TSPO	6.67	6.99	6.13
MYO1C	6.67	7.11	5.72
ASCC3	6.67	7.06	6.06
DDX24	6.67	7.01	6.24
EFR3A	6.67	7.20	5.86
SNRPD1	6.67	7.42	5.81
ATP6V1G2-DDX39B	6.67	6.94	6.35
MEDAG	6.67	7.69	5.21

LOC100132352	6.67	7.23	5.96
SHMT2	6.67	7.06	6.16
CPNE2	6.67	7.08	6.24
ATP6V0A1	6.67	7.01	5.97
SRPR	6.67	7.00	6.24
CDR2	6.67	7.24	5.94
DUX4L9	6.67	7.20	6.01
PTCHD3P1	6.67	6.96	6.29
PLAUR	6.67	8.18	5.64
MTR	6.67	6.95	6.02
MTMR11	6.67	6.88	6.44
DPY30	6.67	7.16	6.12
MALL	6.67	7.31	6.22
PSMD13	6.67	7.06	6.31
USP47	6.67	7.21	5.96
ZBED5	6.67	6.91	6.34
MRPS18A	6.66	6.96	6.17
ZNF767	6.66	7.09	6.11
GOLGA8CP	6.66	6.96	6.35
CFB	6.66	7.14	6.08
KIAA1598	6.66	6.97	6.07
RPL23AP82	6.66	6.97	6.26
NEBL	6.66	7.40	5.93
POLI	6.66	7.08	6.18
DAP	6.66	7.09	6.31
ZKSCAN1	6.66	7.22	6.30
PIAS1	6.66	7.05	6.30
ZNF652	6.66	6.96	5.93
DCUN1D1	6.66	7.03	6.01
SLC35G5	6.66	7.78	6.07
MRFAP1	6.66	7.02	6.06
PANK3	6.66	7.36	5.82
PPA1	6.66	6.93	6.13
RUFY3	6.66	7.08	5.89
NUB1	6.66	6.88	6.29
MIR3689E	6.66	7.91	5.73
C2orf15	6.65	7.12	6.32
WDR33	6.65	7.11	6.28
FAM168A	6.65	7.11	6.38
MED10	6.65	7.34	5.95
NDUFA8	6.65	7.09	6.11
MUC1	6.65	7.39	5.44
MIR571	6.65	7.13	6.32
NDRG2	6.65	7.49	5.71
KIAA1109	6.65	7.02	6.12
INHBA	6.65	7.73	5.19
GOLGA8F	6.65	7.00	6.37
NFATC2	6.65	7.18	5.60
SASH1	6.65	6.99	6.25

CEP112	6.65	6.98	6.10
OTTHUMG00000001097	6.65	6.80	6.28
VKORC1L1	6.65	7.40	6.04
ZNF148	6.65	7.10	5.90
SYNJ2BP-COX16	6.64	7.17	6.12
ATP10D	6.64	6.97	6.34
HOXA5	6.64	7.14	6.02
GGTLC2	6.64	7.41	6.30
TAOK1	6.64	7.16	5.70
UBE2J1	6.64	7.39	6.02
NBR1	6.64	7.03	6.16
EIF2B1	6.64	6.96	5.89
SCIN	6.64	7.41	5.53
POLR3K	6.64	7.11	6.27
MIR646	6.64	7.00	6.07
PDXDC2P	6.64	6.99	5.83
MOCS2	6.64	6.93	6.20
MAPKAP1	6.64	6.94	6.22
MKRN2	6.64	6.94	6.35
USP36	6.64	6.84	6.21
CUL4A	6.64	7.02	5.73
SFXN3	6.64	7.00	6.20
TBC1D15	6.64	6.97	6.10
PDIA4	6.64	7.12	6.11
CHST6	6.64	7.53	5.93
CLPTM1	6.64	7.20	6.22
OTTHUMG00000032220	6.64	7.12	6.22
UBE2V1	6.64	6.92	6.30
PTPRD	6.64	7.52	5.10
KAT2B	6.63	7.24	6.08
RUFY2	6.63	6.96	6.06
COX7A2	6.63	6.98	6.22
SLC35F5	6.63	7.31	5.67
KLF2	6.63	7.09	6.38
HSPG2	6.63	6.85	6.13
EZH1	6.63	7.05	5.95
NARR	6.63	6.99	6.29
GOLGA3	6.63	7.03	6.36
SDHD	6.63	6.88	6.23
LOC100287098	6.63	6.97	6.30
GANAB	6.63	7.04	6.10
NHLRC3	6.63	7.08	6.08
C19orf70	6.63	6.95	6.16
KRTAP10-10	6.63	7.17	6.20
ITGB5	6.63	7.11	5.66
NRBP1	6.63	7.03	5.91
PHKB	6.63	7.06	6.00
SEMA3E	6.63	7.61	5.22
DDX50	6.63	7.10	6.22

HLF	6.63	7.58	5.61
BAG6	6.63	6.84	6.19
REST	6.63	6.94	6.03
LDB1	6.62	6.94	6.07
ZC3H14	6.62	6.86	6.24
DYNLT3	6.62	7.15	5.95
DOCK1	6.62	7.05	6.01
UBE4A	6.62	7.11	6.24
GAGE12C	6.62	7.22	5.82
ANKRD36B	6.62	7.10	5.95
PAIP2	6.62	6.94	6.29
RBM8A	6.62	6.86	6.29
UGGT1	6.62	7.00	6.26
SLC9A3R1	6.62	7.00	5.98
NR2C2	6.62	6.86	6.02
MDP1	6.62	6.98	6.29
CGB8	6.62	7.41	6.10
TOB1	6.62	7.09	5.82
KNOP1	6.62	7.02	6.13
CUTC	6.62	7.08	5.94
TNNT3	6.62	7.39	5.92
PGM3	6.62	7.09	5.99
SUPT7L	6.62	6.84	6.14
TGOLN2	6.62	7.00	6.22
ARF3	6.62	6.85	6.29
LGALS3	6.62	6.90	6.15
ERGIC3	6.62	6.87	6.40
SIN3A	6.62	7.05	6.21
OTTHUMG00000183874	6.62	7.23	5.96
MARS	6.62	6.93	6.03
ANGPTL5	6.62	7.25	6.03
SNORA5A	6.62	7.06	5.89
OR4N4	6.62	7.64	6.00
SNIP1	6.62	6.90	6.39
AARS	6.62	7.27	6.13
OTTHUMG00000169612	6.62	7.05	6.11
PROS1	6.62	7.03	5.95
RALB	6.62	7.41	5.80
OTTHUMG00000059509	6.62	7.16	5.99
RGS16	6.62	7.41	6.10
LINC00173	6.62	7.73	5.29
SERF1A	6.61	6.85	6.34
ISOC2	6.61	6.91	6.21
MIR557	6.61	7.50	6.06
TMEM208	6.61	7.52	5.57
LOC100507672	6.61	7.39	6.20
C6orf70	6.61	7.12	5.94
DNAJC19	6.61	6.88	6.04
PREPL	6.61	7.17	6.00

SRGAP2C	6.61	7.00	6.07
TAB3-AS2	6.61	7.11	6.10
TPP1	6.61	7.09	6.08
SAP130	6.61	7.01	5.98
GPATCH8	6.61	7.10	6.18
CNOT3	6.61	6.85	6.31
YES1	6.61	7.12	5.70
CDV3	6.61	7.14	6.02
SCAF4	6.61	7.01	6.24
SBNO1	6.61	7.18	5.89
XPR1	6.61	7.09	5.90
ATF1	6.61	6.95	5.96
ENTPD4	6.61	7.13	6.07
NPEPPS	6.61	7.02	6.14
FOXJ2	6.61	7.00	6.04
SMU1	6.61	7.19	5.84
GLUL	6.61	7.02	5.92
HSPH1	6.61	7.85	5.53
STAU1	6.61	7.00	5.79
RHOJ	6.61	7.19	6.18
KIAA1468	6.61	6.81	6.32
TGFB1	6.61	7.06	5.92
LPCAT3	6.61	6.97	6.16
CCT3	6.61	7.10	5.69
MYO9A	6.61	6.90	6.17
SERPINA1	6.61	7.11	5.94
RPL35A	6.61	6.90	6.40
ICT1	6.61	7.01	5.82
DENND4A	6.61	7.00	6.12
FAM49A	6.60	7.20	5.55
NAA38	6.60	7.05	6.18
FBLN1	6.60	7.30	6.03
MAP3K7	6.60	6.95	5.95
USP3	6.60	7.06	6.24
CYP2A7	6.60	7.35	6.08
CD97	6.60	7.60	5.36
TIMM13	6.60	7.29	6.03
NCSTN	6.60	6.89	6.25
TAB1	6.60	6.92	6.20
CTNNA1	6.60	7.06	5.75
CALD1	6.60	7.15	6.02
YPEL3	6.60	7.11	5.99
SSB	6.60	6.94	6.26
METTL7A	6.60	7.20	5.15
ETF1	6.60	7.08	6.09
HAS2	6.60	7.10	6.01
SOS2	6.60	7.09	5.96
FBXL4	6.60	6.96	5.99
CLIP1	6.60	6.90	6.04

RALGAPB	6.60	6.85	5.81
SPG11	6.60	6.90	6.00
APOA1BP	6.60	7.01	5.93
TDRD3	6.60	6.81	6.07
EXT1	6.60	7.14	5.82
XBP1	6.60	7.05	6.06
GPX3	6.60	7.65	5.77
CEBPZ	6.60	6.92	5.85
SMIM15	6.60	7.20	6.06
KIAA2026	6.60	6.94	6.24
ZNF667-AS1	6.60	7.03	6.15
SMARCAD1	6.60	7.05	6.00
TRAK2	6.59	7.40	5.68
MIR604	6.59	6.96	6.07
PDHB	6.59	6.97	5.91
PPIP5K2	6.59	7.17	6.17
WBSCR16	6.59	6.79	6.23
PPIL4	6.59	6.92	6.00
RNA5SP486	6.59	6.95	6.11
TRIM25	6.59	6.95	6.05
EVC	6.59	7.13	6.17
LINC00957	6.59	7.18	6.03
SEC24C	6.59	6.80	6.41
OSTF1	6.59	7.08	6.15
RABGEF1	6.59	7.14	6.04
IFITM3	6.59	6.96	5.92
COMMD7	6.59	7.01	6.01
PRKRIP1	6.59	6.93	6.17
CDC16	6.59	6.83	6.05
FRS2	6.59	7.18	5.70
RNPS1	6.59	7.00	5.68
TMOD1	6.59	7.61	5.81
MORN2	6.59	7.15	6.04
MYO1E	6.59	7.32	5.92
HLA-G	6.59	6.98	6.23
YIPF3	6.59	7.04	5.69
SUPT6H	6.59	6.93	6.01
TMEM88B	6.59	6.87	6.34
GPX1	6.59	7.19	6.08
GTF2IRD1P1	6.59	7.14	6.13
HLA-F	6.59	6.84	6.32
SLX1A-SULT1A3	6.59	6.97	6.41
H19	6.59	7.51	5.76
MPHOSPH8	6.58	7.00	6.04
MRPL21	6.58	6.85	6.10
COPS7A	6.58	6.80	6.33
SUN1	6.58	7.17	5.87
FTH1P3	6.58	6.85	6.30
FLJ32255	6.58	6.96	6.08

SECISBP2	6.58	6.80	6.25
TNPO2	6.58	6.99	5.97
MIR98	6.58	7.15	5.84
GAGE2A	6.58	7.24	5.80
C16orf62	6.58	6.83	6.10
TAF10	6.58	6.94	5.98
MIR324	6.58	7.43	5.90
STARD9	6.58	6.87	6.23
MIR103A2	6.58	7.02	6.13
NUP153	6.58	7.19	5.57
SNORD114-14	6.58	7.70	5.60
TIMM10	6.58	6.84	6.02
HS2ST1	6.58	7.29	6.07
SPECC1L	6.58	7.07	6.07
CLCF1	6.58	6.77	6.42
COX15	6.58	6.94	6.24
HECTD1	6.58	6.94	6.12
WHAMMP3	6.58	6.86	6.27
HAGH	6.58	6.93	6.03
HMOX1	6.57	7.19	5.49
TECR	6.57	6.90	6.16
STT3B	6.57	7.01	6.08
CD93	6.57	6.96	5.96
ZNF326	6.57	6.90	6.17
CALCOCO2	6.57	6.78	6.40
ACP1	6.57	7.12	5.89
USP4	6.57	7.01	5.75
PEA15	6.57	7.23	5.88
TPGS2	6.57	7.23	5.72
UBE2Z	6.57	7.10	6.00
PRPF3	6.57	6.92	6.13
POLDIP3	6.57	7.04	5.97
RNF10	6.57	6.84	6.26
NOL7	6.57	6.92	6.04
WSB2	6.57	7.20	5.98
PSMA4	6.57	6.97	5.82
WAPAL	6.57	7.05	5.96
PTBP1	6.57	7.17	5.64
MANBA	6.57	6.87	6.12
DDA1	6.56	6.97	5.78
DIAPH2	6.56	7.12	5.81
ALDH18A1	6.56	7.11	5.90
DENND5A	6.56	7.11	5.70
GAGE13	6.56	7.14	6.01
FAR1	6.56	7.19	5.94
GTF2IRD2	6.56	6.82	6.27
MRPL37	6.56	7.06	5.96
SPIRE1	6.56	7.08	5.96
ANAPC11	6.56	7.25	5.92

KIAA1586	6.56	7.12	5.91
C16orf13	6.56	6.98	6.15
SEL1L	6.56	7.14	5.75
SHPRH	6.56	6.85	6.16
HCG11	6.56	6.79	6.13
IGK	6.56	7.76	5.44
DNM1L	6.56	7.05	5.96
C12orf57	6.56	6.91	6.17
ATP6V0CP3	6.56	7.41	6.07
MICB	6.56	6.83	6.29
LIMA1	6.56	7.07	5.82
GBE1	6.56	6.93	5.89
MICA	6.56	7.00	6.13
SCAPER	6.56	6.88	6.02
UFC1	6.56	7.06	6.17
PSME4	6.56	6.92	6.10
COX7B	6.56	6.96	5.91
ATF7IP	6.56	6.82	5.93
METAP2	6.56	6.92	6.01
IFNAR1	6.56	7.26	5.68
SLC30A7	6.56	7.01	6.03
BMS1	6.56	6.99	6.21
RNF213	6.55	6.90	5.96
TRPC4AP	6.55	6.99	6.10
OTTHUMG00000002261	6.55	7.21	6.07
SKIV2L2	6.55	6.99	6.02
EED	6.55	7.04	6.24
CCL2	6.55	7.86	4.89
CDC42SE1	6.55	6.98	6.04
WDR75	6.55	7.21	6.00
GADD45A	6.55	7.32	5.82
LOC388152	6.55	6.88	6.27
NACA	6.55	6.90	6.07
TSPAN3	6.55	6.86	6.17
STAG3L1	6.55	7.14	6.11
ZNF385B	6.55	7.36	5.10
PTPN14	6.55	7.15	5.69
ICK	6.55	6.77	6.30
TUBG1	6.55	6.78	5.85
MIR3916	6.55	6.82	6.22
PRPF4B	6.55	6.99	5.94
U2AF1	6.55	6.79	6.26
SNORA57	6.55	6.70	6.36
SMDT1	6.55	6.83	6.00
VPS35	6.55	7.12	5.75
NASP	6.55	6.83	6.24
STRAP	6.55	7.27	6.05
CDC42BPB	6.55	6.75	6.02
C21ORF116	6.55	7.07	6.16

SOS1	6.54	7.03	5.88
SECISBP2L	6.54	7.11	6.16
MFN2	6.54	6.87	6.07
PRSS8	6.54	7.07	6.21
BEX4	6.54	7.41	6.09
ETS1	6.54	7.09	5.77
ZNF292	6.54	6.99	6.24
HNRNPU-AS1	6.54	6.89	5.91
LINC00965	6.54	6.86	6.22
ACIN1	6.54	6.88	6.05
RNF11	6.54	7.07	5.82
RNY3P9	6.54	7.29	5.88
LOC100506710	6.54	7.11	5.59
TJP1	6.54	7.08	5.78
KLHDC2	6.54	6.85	5.90
SETD3	6.54	6.85	6.04
VAMP5	6.54	6.93	6.03
PIAS2	6.54	6.90	6.06
MIR3911	6.54	7.03	5.65
TAF11	6.54	7.02	6.09
MYH9	6.54	7.00	5.93
HIST1H3I	6.54	8.23	5.12
RWDD1	6.54	6.96	6.04
SLC44A1	6.54	7.00	5.96
AHI1	6.53	7.15	6.05
HERC2P2	6.53	6.98	5.93
PROCR	6.53	7.18	6.00
TNFAIP1	6.53	7.03	5.85
OTTHUMG00000170029	6.53	7.17	5.76
TEAD2	6.53	7.03	5.96
PTPRG	6.53	7.03	5.63
KRCC1	6.53	6.96	5.82
TNRC6A	6.53	7.00	5.66
KIAA1328	6.53	6.80	6.01
TMEM87A	6.53	7.43	5.77
FKBP7	6.53	6.85	6.14
LHFP	6.53	6.90	5.87
STARD4-AS1	6.53	6.89	5.39
MLLT10	6.53	6.87	6.14
MIR99B	6.53	7.00	6.13
SEH1L	6.53	6.81	5.96
RAB3GAP1	6.53	6.97	6.05
DYNC1LI2	6.53	6.98	5.81
C10orf10	6.53	7.40	5.82
ADK	6.53	6.84	5.86
SUPT20H	6.53	6.87	6.06
CMC2	6.53	7.31	5.95
CDK17	6.53	6.99	5.85
LOC80154	6.53	6.76	6.33

TREML3P	6.53	7.13	5.66
MAGEH1	6.53	7.08	5.72
SENP7	6.53	7.04	6.01
NAPG	6.53	7.18	5.57
FLJ38717	6.53	7.04	6.00
TMEM141	6.53	6.81	6.30
EPC2	6.53	6.88	6.00
TPP2	6.53	6.82	6.22
OTTHUMG00000162779	6.53	7.24	5.93
SLC39A14	6.53	7.21	5.91
C16orf72	6.53	6.90	5.88
ATP6V0C	6.53	7.25	5.63
MAT2A	6.53	7.03	5.80
NUS1	6.52	7.04	6.02
ZFYVE16	6.52	6.96	5.99
DCP2	6.52	6.94	5.82
GTF2I	6.52	6.86	6.15
OTTHUMG00000172523	6.52	6.87	6.31
VPS13C	6.52	7.08	5.62
LOC100289574	6.52	7.05	6.22
ZNF254	6.52	7.10	6.02
C1R	6.52	7.11	6.01
CAT	6.52	6.95	5.97
CCDC50	6.52	6.72	6.15
PPP1R18	6.52	6.96	5.80
PIP4K2B	6.52	6.97	5.94
TXNL1	6.52	7.02	5.93
FAXDC2	6.52	7.07	5.95
AXL	6.52	7.18	5.33
PRPF38A	6.52	6.76	5.86
WBP11	6.52	7.08	6.10
TSC22D2	6.52	6.98	5.76
NTAN1	6.52	6.94	5.80
ZFAND3	6.52	6.95	5.68
OTTHUMG00000163312	6.52	7.00	6.16
BBS2	6.52	7.04	5.87
LOC286109	6.52	7.19	5.63
GAGE12H	6.52	7.15	5.76
MAP1LC3B2	6.52	6.91	6.02
CYB5A	6.52	7.06	5.66
BTG2	6.52	6.97	6.22
UBR5	6.52	6.99	5.92
FLII	6.52	6.91	6.00
PDGFRB	6.52	7.29	5.41
XG	6.52	7.27	5.17
MAPKAPK2	6.52	7.11	5.93
UHMK1	6.52	6.88	6.11
SPEN	6.52	6.92	5.97
RAB12	6.52	6.93	5.90

NT5DC2	6.52	7.06	5.88
RPRD2	6.52	6.82	6.02
RBM34	6.52	6.98	5.80
RNA5SP113	6.52	6.96	6.06
MEX3C	6.52	7.23	5.62
SMOC1	6.52	7.48	5.71
NIN	6.52	7.03	5.79
USP24	6.52	7.06	5.82
ZEB2	6.51	7.35	5.73
LOC101060414	6.51	7.25	6.07
FLJ44342	6.51	6.97	5.67
CMAS	6.51	6.88	5.99
GPR89B	6.51	6.83	5.98
APPL1	6.51	6.81	6.19
RIT1	6.51	6.79	6.01
LOC100129129	6.51	7.06	6.25
MIR4256	6.51	7.07	5.64
JDP2	6.51	6.74	6.12
GPR126	6.51	8.10	4.06
CLASP1	6.51	7.11	6.01
PRPF40A	6.51	6.88	5.50
ADAP2	6.51	7.73	5.92
RNASEH2C	6.51	6.77	6.04
ARFGEF2	6.51	6.87	5.58
UBE2L3	6.51	6.83	6.11
UBE2G1	6.51	7.02	5.82
VPS28	6.51	6.79	6.21
SRM	6.51	6.86	5.85
OTTHUMG00000151714	6.51	7.32	5.77
RECQL	6.51	7.02	5.99
WBP1L	6.51	6.97	6.05
OTTHUMG00000176466	6.51	6.88	6.04
TRAPPC5	6.51	6.93	5.89
PLEKHH2	6.51	7.09	5.90
FAM32A	6.51	6.89	5.89
MTF1	6.50	6.87	5.99
MRPS7	6.50	6.88	6.14
CTCF	6.50	7.00	5.84
SLX1B-SULT1A4	6.50	6.65	6.34
CPSF6	6.50	6.94	6.13
DZIP1	6.50	6.78	6.00
DENND4C	6.50	6.95	5.94
MLLT4	6.50	6.97	5.86
ATP5F1	6.50	6.83	5.98
GOLGA5	6.50	7.13	5.84
SF3B3	6.50	6.88	5.84
YTHDF3	6.50	6.90	5.96
TSC1	6.50	6.85	6.15
KCTD9	6.50	6.87	5.99

MT1L	6.50	7.38	5.72
RBFOX2	6.50	6.94	5.97
COL10A1	6.50	7.64	4.48
CD151	6.50	7.06	5.74
NPIPL2	6.50	6.90	6.15
TMCO3	6.50	7.17	5.90
TMEM176B	6.50	6.96	5.98
KARS	6.50	6.84	6.05
FOXN3	6.50	6.84	6.06
HMGN2P15	6.50	6.75	6.04
SMARCC2	6.50	6.82	5.91
KRTCAP2	6.50	6.88	6.20
MIR3975	6.50	7.31	5.65
VPS26A	6.50	7.07	5.83
TRIB2	6.50	7.00	5.83
EDEM2	6.50	7.09	6.10
GNAI3	6.50	7.29	5.72
SMARCB1	6.50	6.99	6.09
GOLGA8J	6.50	7.21	5.97
SDAD1	6.50	6.92	5.84
CSF1R	6.50	7.34	5.87
DYNC2H1	6.50	6.77	6.27
ADAM12	6.50	8.83	4.91
HLA-DQA2	6.50	7.90	5.52
BOC	6.50	7.10	5.90
OTTHUMG00000160538	6.50	6.78	6.02
TMEM248	6.50	6.97	6.05
MFN1	6.50	6.89	5.86
NXF1	6.50	6.82	6.10
PIK3CA	6.50	6.87	6.10
CNOT4	6.49	6.72	6.20
RBM3	6.49	6.83	5.87
HBS1L	6.49	6.90	5.91
GDF10	6.49	7.51	5.46
THBS3	6.49	7.31	5.63
HBG1	6.49	7.63	5.97
P4HA2	6.49	7.41	5.82
MMP24-AS1	6.49	7.30	5.83
MAR7	6.49	6.97	5.74
MS4A6A	6.49	7.91	5.76
GAS5	6.49	6.83	6.09
SNAPC1	6.49	7.08	5.84
VTI1B	6.49	7.07	5.82
HDGFRP3	6.49	7.00	5.15
PRR14	6.49	6.68	5.98
LINC00294	6.49	6.80	6.16
RWDD4	6.49	6.85	5.97
MARVELD1	6.49	7.12	5.63
FAM156B	6.49	6.79	6.14

PTP4A3	6.49	6.91	5.85
ZNF638-IT1	6.49	6.84	6.00
C1GALT1	6.49	7.06	5.92
CASK	6.49	6.90	5.71
PHF21A	6.49	6.86	6.19
YAE1D1	6.49	6.68	5.89
LRRC16A	6.49	6.93	6.07
CNN2	6.49	7.10	6.03
TRBV11-1	6.49	6.89	5.96
ZMYND11	6.49	6.67	6.00
SLC25A36	6.48	6.80	6.01
RTF1	6.48	6.83	5.79
TXLNA	6.48	7.10	5.79
TAC4	6.48	7.15	5.91
ELTD1	6.48	8.25	4.14
C2CD2	6.48	7.14	5.94
CTDSP2	6.48	6.87	5.82
C1orf85	6.48	7.00	5.99
LRRC41	6.48	6.77	6.16
OTTHUMG00000172175	6.48	7.31	5.70
IGLV1-36	6.48	7.11	6.07
MIR3620	6.48	6.94	5.94
MIR27B	6.48	7.31	5.42
OTUD5	6.48	6.78	6.14
ZFP91-CNTF	6.48	6.82	5.97
PVRL3	6.48	6.84	6.17
RSRC2	6.48	6.80	5.91
CBR3	6.48	7.19	5.41
WWP1	6.48	6.87	5.83
ANXA11	6.48	6.67	6.29
ECD	6.48	6.74	6.01
RAB18	6.48	6.89	5.89
IKBKAP	6.48	6.94	5.86
HIST2H2AB	6.48	6.93	5.57
CCND1	6.48	7.17	5.72
EPN1	6.48	6.99	6.07
GOLT1B	6.48	7.26	5.62
FYTTD1	6.48	6.94	6.02
JAG1	6.48	6.99	5.83
ITPA	6.47	6.93	5.99
API5	6.47	7.15	5.94
RNA5SP190	6.47	6.97	6.00
ATP8B1	6.47	7.04	6.02
LAMB1	6.47	7.15	5.76
SARNP	6.47	7.22	5.88
PIH1D1	6.47	7.00	5.92
DNAJC13	6.47	7.10	5.72
CRNKL1	6.47	6.84	5.71
BICC1	6.47	7.25	5.46

GABBR1	6.47	6.70	6.14
DR1	6.47	6.94	5.90
TROVE2	6.47	6.88	5.77
ARHGAP35	6.47	7.05	5.87
PSMC1	6.47	6.89	5.82
METTL10	6.47	6.87	5.84
NDUFS2	6.47	6.81	5.96
UBE4B	6.47	6.81	6.07
CALM3	6.47	6.72	6.14
ANKRD36BP1	6.47	7.17	5.24
ZNF592	6.47	6.87	6.03
ZC3HAV1	6.47	6.87	5.95
GABPB2	6.47	7.01	6.00
DPYD	6.47	7.10	5.97
PRKACA	6.47	6.84	6.04
ITPRIPL2	6.47	6.83	6.10
NDUFB3	6.47	6.90	6.11
DCTN3	6.47	6.75	6.15
SNORD67	6.47	7.15	5.77
MIR4692	6.46	7.09	5.88
IPO9-AS1	6.46	7.33	5.81
PAICS	6.46	7.01	5.93
LOC100506053	6.46	7.63	5.50
LOXL1	6.46	7.90	5.53
SEC13	6.46	6.93	5.83
COG1	6.46	6.69	6.05
MAGT1	6.46	6.95	5.93
JHDM1D	6.46	7.26	5.43
FOXS1	6.46	6.87	6.03
ITSN2	6.46	6.82	5.96
KIF13A	6.46	6.86	5.74
ATP6V1C1	6.46	7.00	5.71
RNF216	6.46	6.65	6.17
SRP68	6.46	6.77	5.88
GLRX3	6.46	7.03	5.89
TMEM106A	6.46	6.86	5.88
FBXW2	6.46	7.02	5.94
GPR89C	6.46	6.72	6.07
NUTM2D	6.46	6.85	6.15
ALDH3A2	6.46	6.99	5.90
SNX3	6.46	6.84	5.87
TNFRSF1A	6.46	6.79	6.08
LMCD1	6.46	6.86	6.01
BACE1	6.46	6.98	6.00
RAB3GAP2	6.46	6.92	5.90
MIR1269A	6.46	7.24	5.91
RB1	6.46	6.83	5.85
IL17RA	6.46	6.77	6.15
SDHC	6.46	6.97	6.03

CCT7	6.46	6.85	6.02
FZD4	6.46	6.96	5.83
RAB10	6.46	7.13	5.72
MED14	6.46	6.95	5.91
STAM	6.46	7.33	5.75
DOCK7	6.46	7.08	5.70
MAPKAPK5	6.45	6.81	5.94
FLJ43681	6.45	6.85	6.23
FAM160B2	6.45	6.75	6.22
RIMKLB	6.45	6.75	6.22
ETFB	6.45	6.77	6.20
MOB1A	6.45	6.96	5.54
PLEKHM1	6.45	6.80	6.03
ZFAND5	6.45	6.78	6.00
TGFBR3	6.45	7.11	5.65
FBXO22-AS1	6.45	6.94	5.91
LOC389607	6.45	6.85	5.99
PTTG3P	6.45	6.96	5.64
ZRANB1	6.45	6.85	5.67
TMEM39A	6.45	6.93	5.87
CLOCK	6.45	7.06	5.90
IARS	6.45	7.31	5.50
BOD1L1	6.45	6.74	5.93
PSG6	6.45	6.93	5.92
RNF114	6.45	6.91	5.97
NRIP1	6.45	6.95	5.73
ARPC3	6.45	6.93	6.00
MSH6	6.45	6.75	6.12
RNA5SP506	6.45	6.79	5.66
BAZ1A	6.45	6.80	5.71
ATP1B3	6.45	6.76	6.06
RPS6KB1	6.45	6.90	5.95
SLC6A6	6.45	6.88	5.65
RAF1	6.45	6.72	5.98
SH3BGRL	6.45	6.93	5.95
ARMCX1	6.45	6.89	6.07
MTCH1	6.45	6.89	6.03
IGHD2-21	6.45	6.81	5.99
NUP214	6.45	6.72	6.00
OTTHUMG00000013881	6.45	6.95	6.05
CCDC25	6.45	6.80	5.92
SOCS6	6.45	6.78	6.09
TM9SF1	6.45	6.81	6.15
RREB1	6.45	6.88	5.99
SNORD88A	6.45	6.61	6.23
PDIA6	6.45	6.80	5.77
OCIAD1	6.44	6.73	6.17
GDE1	6.44	7.23	5.31
MKL2	6.44	6.99	5.69

CYC1	6.44	6.66	6.05
HMBOX1	6.44	6.73	6.07
DTD1	6.44	7.28	5.70
TGFBRAP1	6.44	6.70	6.15
BRAF	6.44	6.86	5.94
UBE2D1	6.44	6.97	5.91
DDX19B	6.44	6.71	5.69
CLTA	6.44	7.09	5.77
MSANTD2	6.44	6.81	6.24
FOXP4	6.44	6.84	6.22
IL16	6.44	6.85	6.03
AP3M1	6.44	6.93	5.71
MLLT1	6.44	6.73	6.29
TBCB	6.44	7.00	5.88
TPPP3	6.44	7.63	5.30
OR1D2	6.44	7.70	5.74
WFDC13	6.44	6.89	6.09
GTF2B	6.44	6.87	6.00
ERGIC2	6.44	6.95	5.72
CREB3	6.44	6.97	5.68
AQR	6.44	6.87	5.90
DUSP1	6.44	7.14	5.69
IFITM10	6.44	7.42	5.90
TREM1	6.44	8.73	4.24
PPT1	6.44	7.20	6.06
FAM8A1	6.44	6.97	5.93
ZNF780B	6.44	6.85	5.99
CD99P1	6.44	6.94	5.71
VPS25	6.44	6.92	5.78
TMEM259	6.44	6.91	5.79
RFC1	6.43	6.93	5.90
G3BP1	6.43	6.96	5.74
MIR4689	6.43	6.89	6.03
LOC100505876	6.43	6.66	5.99
ZSWIM8-AS1	6.43	6.93	5.92
SEC11C	6.43	6.73	6.12
CTAGE15	6.43	6.75	6.10
CCNT1	6.43	6.76	6.16
HPS3	6.43	6.79	6.04
ADAMTS5	6.43	7.24	4.88
LOC644794	6.43	6.79	5.76
OXLD1	6.43	7.04	5.76
RPPH1	6.43	6.75	6.11
INTS4L2	6.43	6.86	5.99
MT1E	6.43	7.42	5.54
MED4-AS1	6.43	6.98	5.42
ZNF790	6.43	6.67	6.17
OSBPL9	6.43	6.77	5.80
FAM13A	6.43	7.39	6.07

ADSS	6.43	7.03	5.61
LAG3	6.43	7.10	5.93
RIPK1	6.43	6.91	5.88
HAX1	6.43	6.89	6.09
YLPM1	6.43	6.74	6.15
TMX3	6.43	7.00	5.61
RNA5SP449	6.43	7.20	6.00
ICAM2	6.43	7.00	5.91
COPS3	6.43	6.77	5.95
LIMS1	6.43	6.96	5.30
PPP3R1	6.43	6.71	5.94
MIR4522	6.43	6.98	6.13
SCRN1	6.43	7.16	5.85
CNTFR	6.43	7.65	5.24
DEXI	6.43	6.64	5.95
MIR4673	6.43	6.84	5.83
ETFA	6.43	6.72	6.02
FOPNL	6.43	6.82	5.58
KANSL1L	6.43	6.82	5.83
ECM1	6.43	7.40	5.62
SNORD63	6.42	7.29	5.86
EGLN1	6.42	6.87	5.81
MTRNR2L2	6.42	7.31	5.35
TBC1D23	6.42	6.99	6.03
FMNL2	6.42	7.03	5.60
SCCPDH	6.42	6.84	5.72
ESCO1	6.42	6.72	6.02
C16orf91	6.42	6.64	5.97
LOC401357	6.42	7.19	5.61
STX2	6.42	6.95	5.47
YEATS2	6.42	6.84	5.98
CRBN	6.42	6.86	5.80
LAMP2	6.42	7.25	5.43
MMP23A	6.42	6.67	6.33
RXRB	6.42	6.67	5.66
RGS10	6.42	7.17	5.53
STXBP3	6.42	6.93	5.87
TTLL5	6.42	6.68	6.07
DYNLT1	6.42	6.97	5.62
CLK3	6.42	6.72	6.17
LAMTOR3	6.42	7.03	5.41
TCTN2	6.42	6.81	6.08
TMEM167B	6.42	7.18	5.85
GNPAT	6.42	6.99	5.65
CRLF3	6.42	6.96	6.03
ANAPC16	6.42	6.76	5.73
AHCTF1	6.42	7.05	5.63
CD34	6.42	7.69	4.94
PQLC3	6.42	6.94	5.88

COTL1	6.42	7.14	5.88
MIR365A	6.42	6.80	5.88
RELL1	6.42	6.93	5.92
SLC30A9	6.42	6.89	5.65
RPL24	6.42	6.58	6.27
VPS13B	6.42	6.85	5.81
RAB11B	6.41	6.93	5.83
TBC1D17	6.41	6.74	5.99
OTTHUMG00000035468	6.41	6.65	5.98
OTTHUMG00000170442	6.41	6.98	5.96
TBC1D22A	6.41	6.80	5.97
KCNQ5-IT1	6.41	7.33	5.57
C2CD5	6.41	6.84	5.74
MIR4756	6.41	6.84	6.11
PIKFYVE	6.41	6.81	5.82
IDH3G	6.41	6.75	5.61
TUBB6	6.41	6.92	5.59
HK2	6.41	6.93	5.91
POLR2I	6.41	6.91	5.47
HIPK2	6.41	6.75	5.99
PRRC2B	6.41	6.67	6.07
PAPSS1	6.41	6.61	6.22
RBBP6	6.41	6.70	5.69
AKAP10	6.41	6.73	6.15
NUDT1	6.41	6.99	4.88
HLA-DPB1	6.41	7.44	5.74
UBR3	6.41	6.83	5.80
CUL7	6.41	6.82	5.87
SNORD3C	6.41	7.80	5.76
LPGAT1	6.41	6.94	6.00
OSTC	6.41	7.00	5.60
MSL2	6.41	6.61	6.02
YBX3	6.41	6.92	6.05
RPL18	6.41	6.56	6.23
SETD7	6.41	6.67	6.00
ITSN1	6.41	6.86	5.88
PLXNB2	6.41	6.52	6.24
HMCN1	6.40	8.04	4.96
HYMAI	6.40	7.04	5.69
MDC1-AS1	6.40	6.65	5.98
VPS45	6.40	6.84	5.87
ANAPC13	6.40	6.64	6.03
GOSR1	6.40	6.98	6.13
MXRA7	6.40	7.61	5.61
FABP3	6.40	7.16	5.40
TAGLN	6.40	6.93	5.60
OTTHUMG00000164836	6.40	6.71	6.18
ATF6B	6.40	6.65	5.97
TASP1	6.40	6.72	6.04

CSNK1A1	6.40	6.91	5.87
LOC440434	6.40	6.94	5.53
SLC2A1	6.40	7.98	5.04
ZBTB20-AS4	6.40	7.14	5.79
SPCS3	6.40	7.05	5.77
C2	6.40	6.81	6.02
MIR4267	6.40	7.00	5.97
ATP5SL	6.40	6.71	5.87
OTTHUMG00000013244	6.40	6.95	6.02
RYBP	6.40	6.75	5.88
SSRP1	6.40	6.81	6.08
ANKIB1	6.40	6.81	5.31
LMLN-AS1	6.40	6.74	6.14
SLBP	6.40	7.24	5.55
NUDT16L1	6.40	6.94	6.11
TRIM78P	6.40	6.80	6.09
IGF1	6.40	6.86	5.86
CCDC109B	6.40	7.06	5.76
PGGT1B	6.40	6.91	5.74
KIAA1199	6.40	7.99	4.70
SQRDL	6.40	6.91	5.15
MIR4663	6.39	6.79	6.07
SACM1L	6.39	6.80	5.82
PAN3	6.39	6.77	5.73
KIAA1430	6.39	6.73	5.80
CCNL2	6.39	6.60	6.00
FAM89B	6.39	6.85	5.68
OGFOD1	6.39	7.00	5.82
PITPNB	6.39	6.82	5.61
MS4A7	6.39	7.73	5.22
FOS	6.39	7.48	5.35
CRYZ	6.39	6.95	5.49
UBL7	6.39	6.88	5.92
OTTHUMG00000168722	6.39	6.97	6.05
LOC399491	6.39	6.84	5.94
NELFCD	6.39	6.85	5.95
ATP1B2	6.39	7.13	5.24
MDK	6.39	6.95	6.09
TBCA	6.39	6.82	5.56
SNORA80	6.39	6.82	5.98
THBS2	6.39	7.87	4.61
LGALS8	6.39	6.74	5.97
NR1D1	6.39	7.13	5.41
OTTHUMG00000184015	6.39	7.42	5.25
S100A8	6.39	8.18	4.25
TRIM38	6.39	6.79	6.05
SMN1	6.39	7.05	5.75
DUSP11	6.39	6.90	5.67
PIAS4	6.39	6.60	6.22

ISCA2	6.39	6.55	6.14
HEATR3	6.39	6.89	5.80
RAP2C	6.39	6.70	5.69
AKAP11	6.39	6.99	5.85
FBXL5	6.39	6.83	5.84
TRMT13	6.39	6.61	6.18
MSH5-SAPCD1	6.39	6.78	5.94
FDX1L	6.39	6.62	6.17
SUN2	6.39	6.80	6.04
GNA13	6.39	6.79	5.95
HLTF	6.39	7.02	5.82
BCKDHB	6.38	6.74	5.91
THUMPD3	6.38	6.79	5.55
UQCRBP1	6.38	6.65	5.90
RBPJ	6.38	6.88	5.80
WDR60	6.38	6.81	5.73
PIBF1	6.38	6.89	5.85
SCARNA3	6.38	6.81	6.07
TMEM65	6.38	7.07	5.56
TRAPPC11	6.38	6.80	5.88
UROS	6.38	6.70	6.10
OTTHUMG00000040559	6.38	6.58	6.10
PPP1R10	6.38	6.75	5.93
LOC389765	6.38	6.76	5.87
PGAM1P5	6.38	6.77	6.11
SETD5-AS1	6.38	6.66	6.15
SNORA60	6.38	7.63	5.04
IMP4	6.38	6.58	6.15
CEP63	6.38	6.60	6.06
HLA-DMB	6.38	7.54	5.85
PSMC3	6.38	6.70	5.96
GPX8	6.38	7.44	5.03
RPLP0	6.38	6.66	6.06
USP15	6.38	6.75	5.53
GAP43	6.38	6.75	5.92
C8orf46	6.38	6.61	5.90
LOC100505570	6.38	7.18	5.46
CALCOCO1	6.38	6.69	5.92
IGKV1-27	6.38	6.81	6.17
AKR1A1	6.38	7.04	5.66
TMEM109	6.38	6.72	5.98
ACAA2	6.38	7.00	5.13
GALNT5	6.38	6.90	5.17
OTTHUMG00000171037	6.38	6.69	5.88
GLO1	6.38	6.81	5.51
POMZP3	6.37	6.67	5.92
SETD2	6.37	6.87	5.77
KIDINS220	6.37	6.84	5.80
IGHG2	6.37	7.32	5.47

CCNK	6.37	6.76	5.99
MTMR2	6.37	6.80	5.69
SAPCD1	6.37	6.81	5.89
NCK2	6.37	6.72	6.02
GOLGA7B	6.37	7.35	5.03
TULP4	6.37	6.66	5.81
RAP2B	6.37	6.77	5.96
OTTHUMG00000016092	6.37	7.04	6.01
MIR587	6.37	6.96	5.79
TMEM170A	6.37	6.75	5.94
SMC5-AS1	6.37	7.08	5.90
ZNF83	6.37	6.96	5.93
PLCG1	6.37	6.52	6.13
SLC39A9	6.37	6.92	5.74
STK38	6.37	7.03	5.64
DDX58	6.37	6.65	5.92
ECSCR	6.37	6.97	5.29
STAT5B	6.37	6.71	6.07
PTBP2	6.37	6.76	5.88
OTTHUMG00000161703	6.37	6.99	5.77
IAH1	6.37	6.83	5.78
IL4R	6.37	6.73	5.71
C5orf42	6.37	6.79	5.78
SNAPC3	6.37	6.68	5.87
NT5C2	6.37	6.60	5.98
PILRB	6.37	6.88	5.61
SAP30L-AS1	6.37	6.67	6.17
SEPT8	6.37	6.82	5.44
ARHGAP12	6.37	6.93	5.58
RIF1	6.37	6.73	5.89
DNM1P41	6.37	7.01	6.01
SNAR-G2	6.37	7.28	5.83
LRRFIP2	6.37	6.62	6.00
RCN3	6.37	7.26	5.41
WASH2P	6.37	6.86	5.85
FAM214B	6.37	6.81	6.02
UXT	6.37	6.63	5.94
CERCAM	6.37	6.66	5.91
NSMCE1	6.37	6.87	5.86
VCX2	6.37	6.67	5.99
SNX5	6.37	6.74	5.54
LOC100129596	6.37	6.71	6.14
IGKV1-37	6.37	6.81	6.01
UFM1	6.36	6.98	5.61
MIR4786	6.36	6.79	5.83
SUSD5	6.36	7.01	5.94
MRPL14	6.36	7.53	5.54
CLPX	6.36	6.70	5.72
FARSB	6.36	6.85	5.95

CHPF2	6.36	6.80	5.88
MYO5A	6.36	6.95	5.57
SIAH1	6.36	6.62	5.83
ARL4C	6.36	8.55	5.10
HSPA1A	6.36	7.07	5.95
MATR3	6.36	6.58	6.03
SRSF8	6.36	6.67	5.98
PNRC2	6.36	6.91	5.99
SDF2	6.36	6.84	5.75
DTX2P1-UPK3BP1-PMS2P11	6.36	6.74	5.88
TMEM126A	6.36	7.00	5.62
RNF146	6.36	6.63	5.84
RNF115	6.36	6.66	6.00
ITGA1	6.36	7.03	5.73
CDK2AP1	6.36	6.51	6.21
PSMD10	6.36	6.87	5.86
SGCD	6.36	7.23	5.08
ZNF524	6.36	6.55	5.96
CCNH	6.36	6.72	5.85
ZNF318	6.36	6.77	6.07
TP53TG3	6.36	6.82	5.92
FAM27E2	6.36	6.88	5.95
SNORA71A	6.36	7.00	5.91
GPCPD1	6.36	7.08	5.80
R3HDM2	6.36	6.78	5.85
CTDSPL2	6.36	6.81	5.75
DIP2C	6.36	6.83	5.88
CST6	6.36	6.80	5.92
MFAP4	6.35	6.73	5.46
CFI	6.35	7.38	5.70
SNORD53	6.35	6.58	5.95
SUGT1	6.35	6.96	5.39
MAP3K4	6.35	6.61	5.87
NDUFV3	6.35	6.64	6.08
TNFRSF25	6.35	6.94	6.05
PGLS	6.35	6.66	5.83
LIMD2	6.35	6.65	5.93
WIPF1	6.35	7.15	5.77
UTP6	6.35	6.67	5.63
CDC26	6.35	6.83	5.80
ALG2	6.35	6.74	5.88
SLC50A1	6.35	6.72	5.95
SNAR-F	6.35	6.89	5.63
IGLV1-44	6.35	9.14	5.78
EPM2AIP1	6.35	6.66	5.74
ZNF131	6.35	6.57	6.04
LOC154761	6.35	7.05	5.80
TYMS	6.35	6.86	5.68
EIF3F	6.35	6.66	5.82

CELF1	6.35	6.67	5.99
COBLL1	6.35	6.68	5.89
TNFRSF12A	6.35	8.18	5.18
LOC100506142	6.35	6.68	5.99
MOB3A	6.35	6.84	6.06
DIXDC1	6.35	7.25	5.48
DHX9	6.35	6.93	5.48
DYNLL1-AS1	6.35	6.61	6.05
SNAP29	6.35	6.65	5.58
CCDC28A	6.35	6.70	6.04
OTTHUMG00000177423	6.35	6.55	6.20
GEM	6.35	7.00	5.28
RAB23	6.35	7.91	4.96
FAM129A	6.35	7.19	5.12
SLMO2	6.35	6.91	5.68
MYO10	6.35	6.75	5.75
DSEL	6.35	7.41	5.09
BLVRA	6.35	7.05	5.72
FAM73A	6.35	6.65	6.00
SAV1	6.35	6.68	5.70
WDR7	6.35	6.65	5.73
TRBV7-6	6.34	6.67	5.96
SEC11A	6.34	6.87	5.59
KIF2A	6.34	6.64	6.03
MCMBP	6.34	6.80	5.81
POLR1D	6.34	6.78	5.64
SNORA71C	6.34	6.81	5.87
ANKRD13C	6.34	6.68	5.82
CYP20A1	6.34	7.09	5.94
THRA	6.34	7.13	5.61
SEC23A	6.34	7.04	5.88
TLN1	6.34	6.79	5.65
SBF2	6.34	6.80	5.84
DCAF7	6.34	6.69	5.90
TWF1	6.34	7.12	5.70
EOGT	6.34	6.73	5.87
GTF2H5	6.34	6.68	5.93
SYNGAP1	6.34	6.58	5.87
DIAPH1	6.34	6.92	5.76
POLDIP2	6.34	6.70	5.64
OSBP	6.34	6.65	5.90
MRPL53	6.34	6.68	6.01
ARIH2	6.34	6.71	5.78
FAM197Y5	6.34	7.68	4.64
ZCCHC11	6.34	6.69	5.89
RNF185-AS1	6.34	7.13	4.88
STAT2	6.34	6.78	5.60
PCDHB12	6.34	6.71	5.61
RAB8A	6.34	7.08	5.04

TEX35	6.34	6.86	6.04
SSBP2	6.34	6.67	6.08
PPP1R3E	6.34	6.88	5.99
HCP5	6.34	7.20	5.18
EIF4H	6.34	6.79	5.84
FNIP1	6.34	7.02	5.69
LOC100127909	6.34	6.70	5.83
MRPS10	6.34	6.92	5.61
TMUB2	6.34	6.58	6.07
SPATA31C1	6.34	6.75	5.62
LRRC59	6.34	7.02	5.52
RNF214	6.34	6.54	6.14
LOC441454	6.34	6.68	5.81
GUSB	6.34	6.70	5.88
PHF12	6.34	6.69	6.11
APRT	6.34	6.71	5.93
FLNA	6.34	7.21	5.18
DMXL2	6.33	7.18	5.68
ABCF1	6.33	6.62	6.09
AES	6.33	6.50	6.11
CHD8	6.33	6.79	5.74
ARHGAP17	6.33	6.69	5.76
KRTAP10-12	6.33	6.97	5.80
REL	6.33	6.72	6.13
DNAJC21	6.33	6.95	5.83
CHSY1	6.33	7.02	5.66
PRKDC	6.33	6.74	5.80
SCMH1	6.33	6.84	5.71
AHSA1	6.33	6.71	5.79
LOC100509457	6.33	7.26	5.24
MAN2A1	6.33	7.13	5.64
NDUFA4L2	6.33	6.76	5.72
AURKAIP1	6.33	6.77	5.83
IGKV1-9	6.33	6.91	5.73
FAM120B	6.33	6.58	5.36
GABRR2	6.33	6.60	6.09
AGGF1	6.33	6.80	5.81
DNM1	6.33	6.71	5.65
PET100	6.33	6.70	5.92
PLSCR4	6.33	6.90	5.87
DHX32	6.33	6.70	5.96
PRRC2A	6.33	6.75	5.95
VWF	6.33	7.47	4.85
TP53BP1	6.33	6.58	6.07
NFYA	6.33	6.52	5.97
FMR1	6.33	6.76	5.85
NDUFA9	6.33	6.85	5.74
MIR3671	6.33	6.85	5.39
NSD1	6.33	6.66	5.89

RNF19B	6.33	6.96	5.53
PCF11	6.33	6.87	5.36
SAT2	6.33	6.94	5.32
GPSM2	6.33	7.05	5.66
FKBP9	6.33	6.61	5.76
ERLIN1	6.33	7.17	5.19
MEF2C	6.33	6.70	5.64
CERS2	6.33	6.76	5.79
HCFC2	6.33	6.91	5.46
APLNR	6.33	7.97	4.46
CYP1B1	6.32	7.01	5.74
MIR4682	6.32	6.90	6.03
ASH1L	6.32	6.78	5.86
RBL2	6.32	6.67	5.73
MRPS18B	6.32	6.75	6.06
GPR4	6.32	6.96	5.51
SNORD71	6.32	7.16	5.52
TIMM8B	6.32	6.89	5.59
PHC1	6.32	6.82	6.02
FKBP3	6.32	6.93	5.23
FNIP2	6.32	7.57	5.23
CTSO	6.32	6.86	5.28
LDHB	6.32	6.51	6.03
MIR2392	6.32	7.02	5.85
SPDYA	6.32	6.76	5.88
EMC4	6.32	6.65	6.04
RAB8B	6.32	7.28	4.92
IGKV1-16	6.32	7.21	5.72
PQBP1	6.32	6.60	5.95
GSTM3	6.32	6.76	5.52
HDHD2	6.32	6.70	5.84
COPZ1	6.32	6.65	5.81
UFSP2	6.32	7.00	5.85
STMN3	6.32	6.62	6.05
SPCS2	6.32	6.61	5.88
SEPHS1	6.32	6.63	6.05
NIT2	6.32	6.61	5.78
LIN37	6.32	6.62	5.90
RPS29	6.32	6.46	6.13
PTPN13	6.32	7.00	5.66
ANKRD13A	6.32	7.00	5.57
NPFF	6.32	6.59	5.65
EFHD2	6.32	6.60	5.98
SH3YL1	6.32	6.54	5.70
RTCA	6.32	6.79	5.64
SUCO	6.32	6.62	5.88
UPK3BL	6.32	7.11	5.91
NSAP11	6.32	7.05	5.53
ISG15	6.32	7.67	5.82

CPNE3	6.32	6.71	5.91
COG7	6.32	6.67	5.89
PHTF2	6.31	6.91	5.25
DPCD	6.31	6.46	5.96
POLR2H	6.31	6.52	5.87
TCEAL2	6.31	6.87	5.71
MIR3180-4	6.31	6.51	6.03
TLR4	6.31	6.78	5.46
CTSC	6.31	6.92	5.98
STEAP2	6.31	7.50	5.72
MIS18BP1	6.31	6.60	5.73
GOLGA6A	6.31	6.75	5.85
RMDN1	6.31	6.69	5.72
GORAB	6.31	6.72	5.66
ACSL3	6.31	6.70	6.02
ZCCHC17	6.31	6.70	5.86
LOC100288162	6.31	6.62	6.05
C1orf27	6.31	6.78	5.54
RBM27	6.31	6.70	5.92
EHD4	6.31	6.80	5.68
NUMA1	6.31	6.66	6.05
STC2	6.31	8.29	4.98
NAT14	6.31	6.60	6.02
CAMK2D	6.31	6.89	5.44
MIR4748	6.31	6.84	5.87
WWTR1	6.31	6.84	5.77
UBE2R2	6.31	6.84	5.65
KIF22	6.31	6.55	6.03
RNF20	6.31	6.72	5.76
RPS25	6.31	6.48	5.86
OTTHUMG00000020320	6.31	6.97	5.90
SLC43A3	6.31	6.60	5.83
RALBP1	6.31	6.65	5.67
MIR486	6.31	6.67	5.73
ARL1	6.31	6.79	5.82
OTTHUMG00000020540	6.31	6.95	5.78
CHID1	6.31	6.82	5.85
RNU7-6P	6.31	6.81	5.97
C4orf32	6.31	7.32	5.19
TMEM87B	6.31	7.01	5.60
NRBF2	6.31	6.92	5.79
ENY2	6.31	6.79	5.84
STX18	6.30	6.68	6.02
SNX7	6.30	7.19	4.85
OTTHUMG00000169787	6.30	6.64	5.82
WISP3	6.30	7.16	5.21
PSMG1	6.30	6.84	5.53
ELP2	6.30	6.78	5.91
ARHGEF10	6.30	6.76	5.50

NCOA2	6.30	6.64	5.84
NARF	6.30	6.78	5.65
RFWD2	6.30	6.80	5.65
SPAG7	6.30	6.69	5.91
C20orf194	6.30	6.64	5.61
IRF9	6.30	6.58	5.98
TCF12	6.30	6.59	5.95
CKAP5	6.30	7.03	5.41
MED23	6.30	6.57	5.86
AGO4	6.30	6.78	5.75
LSM7	6.30	6.69	5.73
ALG8	6.30	6.75	5.86
RNF181	6.30	6.81	5.73
ABLIM1	6.30	7.12	5.80
NDNF	6.30	7.10	4.82
PER3	6.30	7.40	5.41
LMO4	6.30	6.68	5.64
LOC100129027	6.30	6.86	6.05
RNF141	6.30	6.85	5.82
ANGPTL1	6.30	7.16	5.27
GRK5	6.30	6.68	5.43
ARHGDIA	6.30	6.92	5.14
ATP1B1	6.30	7.13	5.29
ZNF302	6.30	6.90	5.61
LAPTM4B	6.30	6.99	5.81
LINC00266-1	6.30	6.83	5.90
AKIRIN2	6.30	6.77	5.67
SH3BP5-AS1	6.30	6.97	5.73
DTX3L	6.30	6.75	5.83
RNU6-83P	6.30	7.10	5.88
HEBP1	6.30	6.75	5.46
IL11RA	6.30	6.59	5.91
OTTHUMG00000020780	6.30	6.72	5.89
LOC100131564	6.30	6.71	5.54
TMEM2	6.30	6.93	5.80
WNT3	6.30	7.10	5.95
DBI	6.30	6.49	6.04
GNAQ	6.30	6.65	5.83
CAND1	6.30	6.81	5.75
IP6K2	6.30	6.54	5.88
RNA5SP70	6.30	7.05	5.82
PRNP	6.30	6.70	5.91
EIF3J-AS1	6.29	6.63	6.01
GAB1	6.29	6.86	5.31
FEM1B	6.29	6.72	5.91
MLH1	6.29	6.72	5.78
ADAMTS6	6.29	7.32	4.93
SMG9	6.29	6.77	5.86
OTTHUMG00000022274	6.29	6.68	5.77

CLDN3	6.29	6.73	5.93
CP	6.29	7.00	5.84
TM9SF4	6.29	6.66	5.86
RBM12B-AS1	6.29	6.68	5.87
RASA4CP	6.29	6.75	5.77
HEBP2	6.29	6.69	6.03
NQO2	6.29	6.72	5.80
PDLIM7	6.29	7.08	5.85
SDHAP3	6.29	6.63	5.99
LYST	6.29	6.64	5.53
FRYL	6.29	6.71	5.75
PDPK1	6.29	6.50	5.96
CGB	6.29	6.70	5.69
LARP7	6.29	6.70	5.78
SUMF2	6.29	6.62	5.99
UGP2	6.29	6.79	5.69
TAB2	6.29	6.71	5.66
MIR4780	6.29	6.69	5.94
MAST2	6.29	6.75	5.58
OR10P1	6.29	6.75	5.72
MTSS1	6.29	6.72	5.78
EEF1D	6.29	6.56	6.10
FAM96B	6.29	6.96	5.81
SLC33A1	6.29	6.79	5.83
ANKRD36	6.29	6.71	5.63
GOLGA6B	6.29	6.66	5.89
PAPOLA	6.29	6.57	5.61
FOXP1	6.29	6.90	5.76
TALDO1	6.29	6.44	6.10
GMPPA	6.29	6.69	5.91
KDM3B	6.29	6.74	5.66
CEP57	6.29	6.59	5.87
PIAS3	6.29	6.68	5.82
DIDO1	6.29	6.80	5.78
FZD9	6.29	6.94	5.44
MRPL42	6.28	6.62	5.73
NBAS	6.28	6.64	5.77
RING1	6.28	6.50	6.03
C14orf144	6.28	6.63	6.01
USP8	6.28	6.65	5.64
OTTHUMG00000155723	6.28	6.62	5.97
ZNF462	6.28	6.57	5.78
SERPINA5	6.28	7.70	5.34
ABCC1	6.28	6.66	5.71
EFEMP2	6.28	7.01	5.90
CREBZF	6.28	6.68	5.84
KRT18P49	6.28	7.00	5.65
UBR1	6.28	6.68	5.83
CTSZ	6.28	6.84	5.78

COL27A1	6.28	6.79	5.82
FAU	6.28	6.50	6.08
MIER1	6.28	6.78	5.68
CS	6.28	6.55	5.71
NFE2L2	6.28	6.76	5.51
CETN2	6.28	6.57	5.50
MTPAP	6.28	6.68	5.54
GADD45B	6.28	7.10	5.50
RAB30-AS1	6.28	6.71	5.89
PURA	6.28	6.47	5.99
RBMS3	6.28	6.77	5.81
ALMS1	6.28	6.52	5.98
CERS6	6.28	6.86	5.53
PPRC1	6.28	6.59	5.80
C2orf74	6.27	6.82	5.86
IGLV3-16	6.27	6.85	5.88
APOBEC3B-AS1	6.27	6.68	5.70
SLC35C2	6.27	6.58	5.67
APPBP2	6.27	6.81	5.57
SUMO1P3	6.27	6.97	5.37
PDXDC1	6.27	6.69	5.78
OTTHUMG00000169784	6.27	6.94	5.70
MUT	6.27	6.98	5.62
PAPD7	6.27	6.77	5.79
GFPT2	6.27	7.09	5.18
SNED1	6.27	6.59	5.99
RBMS2	6.27	6.67	5.76
OXSR1	6.27	6.95	5.80
DYNC1I2	6.27	6.66	5.68
MAX	6.27	6.61	5.86
MED4	6.27	6.74	5.47
PPFIA1	6.27	6.58	5.82
LBH	6.27	6.87	5.68
RCOR1	6.27	6.56	5.63
LOC100133039	6.27	6.43	5.99
DZIP3	6.27	6.61	5.82
LOC286367	6.27	6.80	5.83
SLMAP	6.27	6.59	5.85
MRPL3	6.27	6.73	5.79
B4GALT5	6.26	7.07	5.60
SNX17	6.26	6.58	5.75
FKBP2	6.26	6.54	5.74
ZNF585A	6.26	6.50	6.03
KIAA0586	6.26	6.66	6.05
ATP6V1B2	6.26	7.18	5.16
CALHM2	6.26	6.67	5.84
GOLGA2P2Y	6.26	6.61	6.04
TGS1	6.26	6.66	6.01
TM7SF3	6.26	6.83	5.07

EME2	6.26	6.55	5.82
EFTUD2	6.26	6.66	5.69
CWC27	6.26	6.58	5.66
HEATR5B	6.26	6.67	5.76
IL10RB	6.26	6.79	5.69
MIR99A	6.26	6.85	5.31
SPTLC1	6.26	7.05	5.83
TNS1	6.26	6.52	5.68
OTTHUMG00000175906	6.26	6.63	5.75
MRPL15	6.26	7.10	5.50
SSC5D	6.26	7.32	4.95
MMADHC	6.26	6.85	5.82
CACNB1	6.26	6.69	5.76
TMEM173	6.26	6.64	5.85
SYNE1	6.26	6.71	5.53
WDR70	6.26	6.69	6.05
PSMB9	6.26	6.91	5.72
IPO9	6.26	6.82	5.80
IGHEP1	6.26	6.56	5.88
NFX1	6.26	6.70	5.69
CLDND1	6.26	6.90	5.57
OTTHUMG00000170026	6.26	6.50	5.90
PCNX	6.26	6.68	5.63
GMPS	6.26	6.91	5.43
LINC00888	6.26	7.13	5.75
MDC1	6.26	6.41	6.12
SLC7A5P2	6.26	6.72	5.71
GOLGA6L1	6.26	6.78	5.76
TOPORS	6.26	6.49	5.87
PDIA5	6.26	6.69	5.58
EPHX1	6.26	6.86	5.84
COG6	6.25	6.74	5.62
CD53	6.25	7.52	5.92
OTTHUMG00000013949	6.25	6.68	5.72
RNASE6	6.25	7.29	5.31
COL4A1	6.25	7.36	5.69
TTL	6.25	6.86	5.72
HIVEP1	6.25	6.90	5.06
LCE2A	6.25	6.89	5.87
EPB41L3	6.25	6.75	5.73
ZZEF1	6.25	6.55	5.75
NDUFA3	6.25	6.67	5.95
RNU105B	6.25	6.79	5.42
MIR3191	6.25	7.02	5.51
RETSAT	6.25	6.66	5.84
IDH2	6.25	6.50	5.91
NDRG3	6.25	6.51	5.88
PLEKHA3	6.25	6.76	5.65
PEX11B	6.25	6.45	5.93

C17orf62	6.25	6.50	5.82
LOC100996511	6.25	6.60	5.11
UQCRC1	6.25	6.77	5.96
FAM57A	6.25	6.52	5.83
TEAD1	6.25	6.94	5.75
CSNK1D	6.25	6.64	5.88
HABP4	6.25	6.62	5.34
PIGB	6.25	6.56	5.65
TCEAL3	6.25	6.59	5.97
TPD52L2	6.25	7.00	5.31
CSAD	6.25	6.67	5.84
RASAL2	6.25	6.61	5.61
GRIFIN	6.25	6.58	5.94
ZBTB44	6.25	6.53	5.92
DDI2	6.25	6.69	5.89
LOC100653515	6.25	6.47	6.08
NDUFA12	6.25	6.57	5.82
IDE	6.25	6.76	5.81
SLIT3	6.25	6.91	5.54
LATS1	6.25	6.74	5.70
RPA3	6.25	6.55	5.79
INO80D	6.25	6.51	5.80
C20orf141	6.24	6.67	5.93
TMEM42	6.24	6.54	6.01
SDF4	6.24	6.62	5.32
ZFC3H1	6.24	6.54	5.94
PPP1CB	6.24	6.55	5.71
NAE1	6.24	6.69	5.75
MAP2K6	6.24	7.20	4.61
LIMS3	6.24	6.77	5.69
TUBG2	6.24	6.64	5.43
ECHS1	6.24	6.60	5.73
NFIL3	6.24	7.05	5.22
MIR657	6.24	6.72	5.73
CHCHD10	6.24	6.39	6.04
QRICH1	6.24	6.46	5.52
AQPEP	6.24	7.20	4.02
LMBR1	6.24	6.76	5.89
ANK3	6.24	7.47	4.83
MPP5	6.24	6.67	5.70
RPL27A	6.24	6.56	5.89
EYA3	6.24	6.75	5.73
TMTC1	6.24	6.86	4.95
MBTPS1	6.24	6.68	5.67
RNA5SP20	6.24	6.46	5.59
TAP1	6.24	6.44	5.99
CYBA	6.24	6.69	5.74
MTX3	6.24	7.04	5.68
DUSP22	6.24	6.63	5.53

MIR3151	6.24	7.26	5.59
MRPL16	6.24	6.65	5.76
TOMM20	6.24	6.41	5.80
IER3	6.24	6.92	5.90
FARSA	6.24	6.65	5.89
SOCS5	6.24	6.57	5.93
MAPK3	6.24	6.54	6.00
PRKAB2	6.24	6.68	5.50
LOC100507419	6.24	7.13	5.68
SNORD20	6.24	6.85	5.92
KIAA1279	6.23	6.55	5.87
DYNC2LI1	6.23	6.73	5.59
KIAA0355	6.23	6.55	5.74
STEAP3	6.23	6.79	5.40
LOC400743	6.23	6.74	5.68
NGEF	6.23	6.55	5.89
USE1	6.23	6.56	5.94
IGKV2D-24	6.23	7.10	5.68
KLHL9	6.23	6.71	5.85
PRDM4	6.23	6.76	5.68
LINC00969	6.23	6.80	5.21
SCARNA5	6.23	7.26	5.39
PTPN1	6.23	6.78	5.52
SERP1	6.23	6.65	5.76
RTCB	6.23	6.58	5.84
TSR3	6.23	6.58	5.63
THY1	6.23	6.62	5.50
TMEM50B	6.23	6.60	5.83
S100A1	6.23	6.67	5.47
YTHDC2	6.23	6.67	5.84
HIBADH	6.23	6.64	5.87
SLC25A39	6.23	6.54	5.91
TXNL4A	6.23	7.25	5.18
FAM129B	6.23	6.68	5.59
IGHV2-70	6.23	7.13	5.57
C4orf29	6.23	6.54	5.84
ATN1	6.23	6.45	5.94
MPZL1	6.23	6.55	5.66
SCO2	6.23	6.58	5.83
PDPN	6.23	6.63	5.59
TMEM33	6.22	6.98	5.55
DENND6A	6.22	6.55	5.54
AKAP8L	6.22	6.44	5.97
LOC729739	6.22	6.61	5.71
OTTHUMG00000014139	6.22	6.78	5.67
BRWD1	6.22	6.70	5.55
TAF12	6.22	6.43	6.01
NFYB	6.22	6.65	5.81
DLGAP1-AS1	6.22	6.45	5.49

SYMPK	6.22	6.48	6.08
ARL8A	6.22	6.54	5.79
SNORA79	6.22	7.13	5.24
PRR7	6.22	6.48	5.85
EIF2AK2	6.22	6.72	5.51
OTTHUMG00000018686	6.22	6.59	5.69
IGHV3-16	6.22	7.42	5.62
GNPTAB	6.22	6.58	5.76
C8orf59	6.22	6.49	5.93
HEATR6	6.22	6.71	5.79
DCBLD2	6.22	6.83	5.42
DONSON	6.22	6.75	5.55
FAM197Y2	6.22	7.50	5.07
GSTTP1	6.22	7.26	5.44
PUM2	6.22	6.73	5.56
UBA3	6.22	6.85	5.23
METTL18	6.22	6.66	5.83
SPRR4	6.22	6.68	5.59
OTTHUMG00000019961	6.22	7.05	5.12
RLF	6.22	6.95	5.52
SNORA59A	6.22	6.68	5.78
AKR1B1	6.22	6.52	5.83
SRF	6.22	6.37	5.95
FLJ42351	6.22	6.70	5.70
SNORD62A	6.22	6.60	5.53
IKBIP	6.22	7.14	5.01
SRCAP	6.22	6.54	5.73
NOSIP	6.22	6.77	5.72
DPYSL3	6.22	6.97	5.36
FLJ35934	6.22	6.63	5.84
HCFC1	6.22	6.58	5.88
PTRF	6.22	6.72	5.55
UROD	6.22	6.40	5.99
SUCLG2	6.22	6.60	5.70
RALA	6.22	6.92	5.01
LAMTOR5	6.22	6.59	5.68
ATXN7L3B	6.22	6.67	5.62
NOB1	6.22	6.62	5.69
OTTHUMG00000067148	6.22	6.89	5.68
GTF3C2	6.22	6.57	5.97
GALNT15	6.22	7.04	5.04
TRBV6-5	6.22	6.88	5.77
MIR3978	6.22	6.65	5.71
ZMIZ2	6.21	6.53	5.89
WDR45	6.21	6.67	5.88
USP33	6.21	6.66	5.56
YY1	6.21	6.50	5.60
PRKCI	6.21	6.85	5.48
FAM220A	6.21	6.56	5.96

RNASE1	6.21	6.99	5.27
GOLGA8S	6.21	6.49	5.76
ACTN1	6.21	6.74	5.69
RAB13	6.21	6.56	5.94
KAL1	6.21	7.03	5.56
RNF103	6.21	6.55	5.76
BCL2L2-PABPN1	6.21	6.50	5.86
FLYWCH1	6.21	6.52	5.78
STK4	6.21	6.53	5.78
MIR4440	6.21	7.22	5.65
RAB27A	6.21	6.44	5.71
LRRC3C	6.21	6.66	5.76
ENDOG	6.21	6.54	5.98
GSN-AS1	6.21	6.52	5.63
TTC19	6.21	6.48	5.76
CEPT1	6.21	6.57	5.74
FKBP11	6.21	6.41	5.90
PRPF38B	6.21	6.53	5.85
PHF2	6.21	6.68	5.92
LPCAT4	6.21	6.51	5.80
OTTHUMG00000159638	6.21	7.33	5.87
APPL2	6.21	7.07	5.25
SMEK1	6.21	6.66	5.52
TRIM47	6.21	6.50	5.80
DHRS4L1	6.21	6.70	5.96
GPR107	6.21	6.42	5.86
EIF2D	6.21	6.54	5.59
LOC100128979	6.21	6.74	5.78
NFU1	6.21	6.46	5.93
TNFSF10	6.21	6.75	5.55
CISD3	6.21	6.66	5.68
PSMD14	6.21	6.89	5.42
UBE2D2	6.21	6.76	5.71
SLPI	6.21	7.65	5.15
RPP21	6.21	6.92	5.40
FKBP4	6.21	6.45	5.74
GTF2H1	6.21	6.48	5.69
NREP	6.21	6.72	5.26
TMEM181	6.21	6.73	5.57
PPP4R1	6.21	6.88	5.62
STK25	6.20	6.54	5.80
ZNF747	6.20	6.59	6.03
CRK	6.20	6.67	5.45
DCTD	6.20	6.52	5.86
CNIH	6.20	6.78	5.63
LRP10	6.20	6.71	5.57
DNAJC2	6.20	6.54	5.75
DYNLL1	6.20	6.56	5.88
IARS2	6.20	6.84	5.43

PRKCA	6.20	6.94	5.50
SAMD4A	6.20	6.75	5.53
PDK3	6.20	6.86	5.35
CASP2	6.20	6.51	5.86
BLVRB	6.20	6.67	5.68
KIF4B	6.20	6.87	5.79
KRBOX4	6.20	6.47	5.59
MIR4314	6.20	6.90	5.78
SLC35B3	6.20	6.85	5.61
AATF	6.20	6.61	5.81
B4GALNT3	6.20	6.51	5.93
KIAA0368	6.20	6.54	5.83
LOC100506365	6.20	6.60	5.66
GULP1	6.20	6.60	5.74
MIR1913	6.20	6.94	5.64
MAVS	6.20	6.47	5.73
ZNF395	6.20	6.58	5.85
AACS	6.20	6.61	5.97
IGLV3-9	6.20	6.62	5.84
FUT11	6.20	6.88	5.48
RGS2	6.20	7.69	4.63
GCNT1	6.20	6.83	5.69
KAT2A	6.20	6.50	5.99
MIR214	6.20	7.35	5.31
SIX5	6.20	6.41	5.89
TES	6.20	6.90	5.31
LRPPRC	6.20	6.57	5.62
FIBP	6.20	6.59	5.49
TP53TG3B	6.20	6.81	5.43
TFDP2	6.20	6.58	5.81
UBTD2	6.20	6.82	5.41
BRE	6.20	6.56	5.80
KCTD13	6.20	6.43	5.86
PFKFB3	6.20	6.90	5.12
ATPIF1	6.19	6.52	6.00
TET2	6.19	6.61	5.46
TPT1-AS1	6.19	6.50	5.77
LOC100130876	6.19	6.64	5.75
BRICD5	6.19	6.62	5.83
C4orf48	6.19	6.74	5.71
MAT2B	6.19	6.35	5.80
OTTHUMG00000164175	6.19	6.82	5.83
APBB2	6.19	6.69	5.46
WDR82	6.19	6.68	5.71
PHF17	6.19	6.86	5.93
SPRN	6.19	6.78	5.59
MIR147A	6.19	6.90	5.66
ARID4A	6.19	6.51	5.85
EFNA5	6.19	6.99	5.56

C7orf73	6.19	6.58	5.82
F5	6.19	7.22	4.46
TCIRG1	6.19	6.39	5.68
FYB	6.19	6.97	5.50
NDUFS4	6.19	6.63	5.68
CCDC59	6.19	6.48	5.73
AK3	6.19	6.46	5.77
ADAR	6.19	6.49	5.85
NCOA4	6.19	6.69	5.50
ALG13	6.19	6.57	5.58
MRPS22	6.19	6.41	5.75
ACAP2	6.19	6.66	5.60
SNW1	6.19	6.66	5.50
PSPC1	6.19	6.53	5.71
ZNF37BP	6.19	6.64	5.97
TRAPPC3	6.19	6.65	5.67
S100A6	6.19	6.38	5.86
DDHD1	6.19	6.55	5.82
CRYBB3	6.19	6.62	5.88
OTTHUMG00000182493	6.19	7.01	5.56
PRPF39	6.19	6.72	5.47
C6orf165	6.19	6.65	5.81
ZNF805	6.19	6.62	5.75
UGGT2	6.19	6.80	5.62
WDR36	6.18	6.52	5.50
BFAR	6.18	6.77	5.54
PTCD3	6.18	6.40	5.86
PPP2CA	6.18	6.63	5.56
GMPR2	6.18	6.47	5.83
ZNF654	6.18	6.80	5.42
TRIM37	6.18	6.49	5.63
HUS1	6.18	6.53	5.53
EPS15L1	6.18	6.67	5.77
GOLGA2P1	6.18	6.70	5.79
LOC645202	6.18	6.74	5.71
CLEC18B	6.18	6.85	5.48
SREK1	6.18	6.48	5.72
SLC23A2	6.18	6.41	5.89
FAM168B	6.18	6.84	5.73
MYO6	6.18	6.74	5.35
SH3PXD2B	6.18	7.19	5.57
UBE2Q2P3	6.18	6.45	5.98
PPP6C	6.18	6.52	5.86
MEGF10	6.18	6.59	5.95
IRF2	6.18	6.46	5.87
FAM27A	6.18	6.58	5.86
PLXND1	6.18	6.67	5.69
SURF2	6.18	6.62	5.70
FAM120A	6.18	6.52	5.61

SMC1A	6.18	6.90	5.50
OFD1	6.18	6.41	5.74
ORC3	6.18	6.39	5.93
BLMH	6.18	6.70	5.41
DLG5	6.18	6.62	5.78
TYW1B	6.18	6.57	5.83
RAB2B	6.18	6.58	5.73
RAD1	6.18	6.56	5.58
ARHGAP21	6.18	6.84	5.34
ATAD1	6.18	6.45	5.47
RNA5SP60	6.18	6.86	5.29
SLC30A1	6.18	6.64	5.35
CILP2	6.18	6.75	5.50
RNF139	6.18	6.56	5.91
C12orf75	6.18	7.16	5.30
AMD1	6.18	6.71	5.56
MAML2	6.18	6.94	5.53
ERVK3-1	6.17	6.55	5.53
C6orf120	6.17	6.44	5.82
DDTL	6.17	6.74	5.74
MRPL20	6.17	6.46	5.73
MESP1	6.17	6.44	6.04
OTTHUMG00000150794	6.17	7.33	5.58
INO80B	6.17	6.41	5.74
R3HDM1	6.17	6.74	5.72
SNORD8	6.17	6.54	5.57
KIF1B	6.17	6.70	5.43
TBX5	6.17	6.68	5.78
KCMF1	6.17	6.98	5.42
TSPAN11	6.17	6.75	5.73
LOC554249	6.17	6.62	5.97
TRIM52	6.17	6.49	5.37
GLIS3	6.17	7.86	5.41
CXCL14	6.17	7.03	4.83
LYL1	6.17	6.39	5.99
OTTHUMG00000155834	6.17	6.68	5.59
ZNF384	6.17	6.47	5.79
NXPH3	6.17	6.47	5.81
MED31	6.17	6.73	5.70
ZFHX3	6.17	6.84	5.41
MRPL39	6.17	6.62	5.49
PPP2R1A	6.17	6.59	5.79
MLX	6.17	6.48	5.49
DGUOK	6.17	6.48	5.74
CCL13	6.17	6.97	5.56
PSMA5	6.17	6.79	5.48
ASAH1	6.17	6.97	5.52
SMIM4	6.17	6.52	5.57
C7orf60	6.17	6.69	5.43

TNRC6B	6.17	6.50	5.51
LOC100505942	6.17	6.55	5.62
LST1	6.17	6.58	5.77
MTMR3	6.17	6.39	5.57
PCMTD2	6.17	6.52	5.75
ATG2B	6.17	6.66	5.39
SLC2A3	6.17	7.22	5.48
HECTD4	6.17	6.36	5.94
CAMTA2	6.17	6.38	5.91
TBCK	6.17	6.47	5.65
LRIG1	6.17	6.35	5.94
CFDP1	6.17	6.55	5.71
LOXL3	6.16	6.88	5.64
RNF217	6.16	6.73	5.72
IGF2R	6.16	6.46	5.74
FAT4	6.16	7.06	5.02
LOC400541	6.16	6.95	5.62
MTIF2	6.16	6.52	5.86
PLN	6.16	6.43	5.79
VEZT	6.16	6.58	5.63
KPNA2	6.16	6.80	5.67
INPPL1	6.16	6.52	5.65
TMCO1	6.16	6.64	5.51
MIR130B	6.16	6.95	5.60
TRIM65	6.16	6.45	5.89
AASS	6.16	6.50	5.77
C8orf33	6.16	6.52	5.91
CXXC5	6.16	6.56	5.67
MRPL12	6.16	6.64	5.78
ARHGEF6	6.16	6.52	5.56
PHF13	6.16	6.44	6.02
ME1	6.16	6.60	5.53
SLC4A7	6.16	6.83	5.34
GID8	6.16	6.46	5.52
FAM20B	6.16	6.60	5.10
CCDC130	6.16	6.36	5.76
GLUD1	6.16	6.49	5.57
RERG	6.16	6.96	5.35
F2R	6.16	6.87	5.74
GSTT2B	6.16	6.64	5.51
ZNF644	6.16	6.48	5.76
CDK2	6.16	6.34	6.00
MAZ	6.16	6.48	5.84
GPR125	6.16	6.54	5.38
SLC3A2	6.16	6.70	5.71
IMPDH2	6.16	6.64	5.85
NCKAP1L	6.16	7.41	5.46
SLC38A1	6.16	6.89	5.59
SNORA11D	6.16	6.56	5.80

FAM98A	6.16	6.74	5.48
LINC00623	6.16	6.40	5.77
EXOSC6	6.16	6.28	5.89
GLOD4	6.16	6.59	5.54
SSTR5	6.16	6.43	5.85
MED12	6.15	6.39	5.75
TCEA1	6.15	6.38	5.79
ZYG11B	6.15	6.84	5.56
LOC100505620	6.15	6.68	5.79
IFNA14	6.15	6.82	5.69
SGK3	6.15	6.42	5.88
USP9X	6.15	6.72	5.33
GSK3A	6.15	6.57	5.66
ZFP36L2	6.15	6.92	5.31
FLJ31306	6.15	6.46	5.82
VTA1	6.15	6.80	5.52
PPAPDC1B	6.15	6.45	5.78
FAM162A	6.15	6.80	5.48
FAM27C	6.15	6.53	5.86
ZNF70	6.15	6.71	5.64
TMEM55A	6.15	6.67	5.35
SMURF2	6.15	6.70	5.45
CTAGE4	6.15	6.46	5.95
DGAT1	6.15	6.47	5.67
MBTPS2	6.15	6.58	5.58
RARRES2	6.15	6.58	5.67
MPHOSPH10	6.15	6.53	5.72
SMAD5	6.15	7.01	5.57
PARL	6.15	6.57	5.51
IWS1	6.15	6.44	5.62
SYVN1	6.15	6.57	5.86
WTIP	6.15	6.35	5.92
LOC493754	6.15	6.56	5.69
TMEM106B	6.15	6.43	5.34
HLA-DMA	6.15	7.11	5.74
FKBP15	6.15	6.59	5.54
BDH2	6.15	6.58	5.38
PHYH	6.15	6.51	5.73
FCHO2	6.15	6.42	5.64
MRC1	6.15	7.12	5.19
CHD1L	6.15	6.89	5.55
NBEAL1	6.15	6.80	5.38
ACP5	6.14	7.25	5.48
MTMR10	6.14	6.73	5.57
XPA	6.14	6.47	5.68
ELOVL1	6.14	6.60	5.74
RUNX1T1	6.14	6.75	5.90
PGM2L1	6.14	7.05	5.53
MT1M	6.14	6.63	5.74

CAMK1	6.14	6.68	5.61
INTS10	6.14	6.45	5.56
FARP1	6.14	6.75	5.43
ZNF704	6.14	6.74	5.67
CLUAP1	6.14	6.61	5.49
FBXL3	6.14	6.51	5.47
UBTF	6.14	6.42	5.58
CYLD	6.14	6.74	5.40
RRAS	6.14	6.59	5.29
VEGFC	6.14	6.68	5.55
PTGIS	6.14	6.78	5.50
CNOT6L	6.14	6.42	5.85
SLC9A9	6.14	6.59	5.29
AGAP6	6.14	6.54	5.20
CD4	6.14	6.61	5.72
FAM120AOS	6.14	6.44	5.87
WDR19	6.14	6.46	5.32
CORO1A	6.14	6.40	5.88
MTHFD1	6.14	6.53	5.85
GLYR1	6.14	6.48	5.56
HSPA13	6.14	7.05	5.19
AP3B1	6.14	6.53	5.50
SNX2	6.14	6.73	5.57
CIRH1A	6.14	6.86	5.53
CDK12	6.14	6.47	5.88
TCTA	6.14	6.41	5.96
GM2A	6.14	6.73	5.47
PTGDS	6.14	7.29	5.26
NUP98	6.14	6.45	5.65
SLC35F6	6.14	6.42	5.69
NFIA	6.14	6.65	5.56
ATXN1	6.14	6.40	5.59
MAU2	6.14	6.36	5.85
CDK7	6.14	6.67	5.66
POLE3	6.14	6.36	5.80
MIR483	6.13	6.59	5.58
SLC35E1	6.13	6.52	5.83
TMEM243	6.13	6.37	5.72
RALGAPA1	6.13	6.60	5.72
C9orf142	6.13	6.59	5.79
SURF4	6.13	6.86	5.45
TPM3P9	6.13	6.60	5.77
METTL5	6.13	6.34	5.64
SESN3	6.13	6.82	5.54
HEXIM1	6.13	6.45	5.80
MRPL54	6.13	6.75	5.83
OR11H12	6.13	6.88	5.64
DDX27	6.13	6.46	5.71
RUVBL2	6.13	6.63	5.62

SLC9A8	6.13	6.42	5.60
MROH1	6.13	6.25	5.98
INPP5F	6.13	6.66	5.39
OTTHUMG00000150172	6.13	7.31	5.39
SAMD9L	6.13	6.75	5.51
ADPGK	6.13	6.71	5.15
SRPK2	6.13	6.53	5.44
SLC25A38	6.13	6.64	5.82
JMY	6.13	6.58	5.53
ATF7	6.13	6.45	5.66
TMEM179B	6.13	6.35	5.78
LAMTOR2	6.13	6.55	5.69
ANAPC7	6.13	6.50	5.82
LOC100505909	6.13	6.91	5.65
NAA60	6.13	6.53	5.62
SLIRP	6.13	6.48	5.53
CYGB	6.13	6.58	5.52
IGLV3-25	6.13	7.09	5.48
RBBP4	6.13	6.45	5.75
HIATL1	6.13	6.53	5.44
XRN2	6.13	6.72	5.56
ZXDC	6.13	6.33	5.55
HERC3	6.13	6.59	5.91
SNORD88B	6.13	6.55	5.39
PTPN18	6.13	6.52	5.82
AEBP2	6.13	6.43	5.56
LOC730268	6.13	6.41	5.33
GTPBP1	6.13	6.48	5.76
TIMM21	6.13	6.75	5.71
UFD1L	6.13	6.51	5.66
USP16	6.13	6.62	5.57
VPS29	6.13	6.59	5.49
EPG5	6.13	6.64	5.68
ALDH7A1	6.13	6.34	5.77
LOC100216546	6.13	6.46	5.61
DMXL1	6.13	6.48	5.53
MBD4	6.13	6.50	5.83
LAMTOR4	6.12	6.38	5.86
USP12-AS1	6.12	6.64	5.00
XPNPEP1	6.12	6.47	5.80
PWP1	6.12	6.85	5.47
LOC92249	6.12	6.46	5.84
DHX16	6.12	6.30	5.94
TMEM205	6.12	6.37	5.80
PPARGC1A	6.12	6.79	5.58
GNL2	6.12	6.60	5.71
C9orf89	6.12	6.38	5.51
AMY1A	6.12	6.44	5.62
NSUN5P2	6.12	6.33	5.90

KIAA1919	6.12	6.43	5.22
XGPY2	6.12	7.34	4.98
LIG4	6.12	6.50	5.66
UBA2	6.12	6.54	5.50
PTPMT1	6.12	6.50	5.27
TBC1D5	6.12	6.39	5.58
CHADL	6.12	6.92	5.37
MED28	6.12	6.45	5.62
SNX13	6.12	6.36	5.53
MCL1	6.12	6.69	5.52
CSPG4P8	6.12	6.37	5.94
SOX12	6.12	6.63	5.84
TUBB2B	6.12	6.86	5.40
LOC642361	6.12	6.34	5.84
IFT74	6.12	6.54	5.50
CCDC93	6.12	6.47	5.47
LSM2	6.12	6.61	5.62
IFNGR2	6.12	6.52	5.44
POU4F3	6.12	6.81	5.74
LOC101060495	6.12	6.65	5.56
LOC100653206	6.12	6.56	5.56
PEAK1	6.12	6.90	5.23
ENSA	6.12	6.26	5.77
SOBP	6.12	6.70	5.39
TERF2	6.12	6.40	5.43
RNU7-35P	6.12	6.57	5.56
SNAPIN	6.12	6.53	5.52
LOC202181	6.12	6.49	5.74
MTOR	6.12	6.33	5.51
RAB11FIP2	6.12	6.57	5.08
KANK2	6.12	6.44	5.73
CCT6A	6.12	6.66	5.34
SERPINI1	6.12	6.99	4.02
ADRB2	6.11	6.42	5.82
GALNT18	6.11	6.66	5.59
RAD23A	6.11	6.43	5.62
OR2L3	6.11	6.73	5.55
GTF2F2	6.11	6.46	5.61
DALRD3	6.11	6.51	5.88
MCEE	6.11	6.51	5.67
ZADH2	6.11	6.37	5.75
ANKRD19P	6.11	6.42	5.83
PDGFC	6.11	6.99	5.31
WDR54	6.11	6.35	5.81
YTHDF1	6.11	6.38	5.86
EIF2B3	6.11	6.45	5.67
MED8	6.11	6.55	5.60
ENOPH1	6.11	6.72	5.65
GGCX	6.11	6.34	5.72

HIP1	6.11	6.63	5.65
EPHA3	6.11	7.06	4.72
ARL14EP	6.11	6.46	5.72
LINC00471	6.11	6.68	5.71
PTK2	6.11	6.43	5.64
ZSWIM6	6.11	7.07	4.87
PTPRS	6.11	6.63	5.28
MEF2D	6.11	6.47	5.61
ARMCX6	6.11	6.37	5.77
P2RY8	6.11	6.40	5.63
SNRPD3	6.11	6.48	5.72
MFAP5	6.11	7.24	5.27
GPHA2	6.11	6.48	5.73
PRR20A	6.11	6.86	5.59
CDKN1B	6.11	6.51	5.64
TMEM63A	6.11	6.50	5.81
BAG5	6.11	6.44	5.81
COL5A1	6.11	7.05	5.57
STX4	6.11	6.52	5.56
NCOA7	6.11	6.55	5.82
PNP	6.11	6.41	5.80
SKIV2L	6.11	6.33	5.86
PTEN	6.11	6.60	5.31
SAP30BP	6.10	6.63	5.27
TSR1	6.10	6.56	5.78
CLEC11A	6.10	6.61	5.76
MIR107	6.10	6.57	5.51
LOC645513	6.10	6.58	5.47
MIR4686	6.10	6.53	5.69
OAF	6.10	6.58	5.64
MRPL47	6.10	6.65	5.20
PDCD2L	6.10	6.31	5.73
CDK13	6.10	6.43	5.47
ATP1A1	6.10	6.46	5.65
MAPK14	6.10	6.51	5.76
PYGL	6.10	6.43	5.80
RNU11	6.10	6.59	5.45
SDHAF2	6.10	6.48	5.56
NABP2	6.10	6.43	5.82
ERCC3	6.10	6.43	5.73
MIR500B	6.10	6.66	5.63
VEZF1	6.10	6.63	5.28
SNORA38B	6.10	6.87	5.45
RCL1	6.10	6.48	5.70
TDG	6.10	6.52	5.81
BCAS3	6.10	6.43	5.85
SETD1B	6.10	6.46	5.76
TOMM70A	6.10	6.45	5.32
C7orf41	6.10	6.35	5.89

NSUN3	6.10	6.44	5.24
CTAGE9	6.10	6.49	5.61
ADORA2B	6.10	6.34	5.58
TUBE1	6.10	6.48	5.70
OTTHUMG00000032918	6.10	6.50	5.77
VSTM4	6.10	6.79	5.42
RINT1	6.10	6.63	5.56
RPL9	6.10	6.33	5.75
ANO1-AS1	6.10	6.82	5.46
XPC	6.10	6.46	5.51
CALCRL	6.10	7.27	4.16
POLH	6.10	6.54	5.51
TBC1D20	6.10	6.64	5.38
VAMP7	6.10	6.63	5.53
CAPRIN2	6.10	6.33	5.60
LOC100506636	6.10	6.40	5.46
DNAJB6	6.10	6.31	5.81
CEBPD	6.10	6.50	5.81
SNAR-H	6.10	6.78	5.40
SCAND2P	6.10	6.54	5.50
SGMS1	6.09	6.41	5.42
DDX39A	6.09	6.57	5.22
GLT8D1	6.09	6.57	5.35
LZTS2	6.09	6.43	5.75
JPX	6.09	6.61	5.49
ANXA2	6.09	6.63	5.47
PODNL1	6.09	6.62	5.62
ISLR	6.09	6.98	5.03
CNOT8	6.09	6.78	5.63
ABCD4	6.09	6.27	5.71
MRPS25	6.09	6.61	5.73
AHNAK2	6.09	6.86	5.31
DDX47	6.09	6.30	5.81
ZNF718	6.09	6.33	5.76
TGFB3	6.09	6.69	5.41
SLC10A3	6.09	6.29	5.82
CTTNBP2NL	6.09	6.68	5.20
TYW3	6.09	6.31	5.72
STOML2	6.09	6.33	5.60
SORT1	6.09	6.72	5.37
IGHM	6.09	6.68	5.45
CRYGS	6.09	6.25	5.77
EXT2	6.09	6.40	5.48
PXDC1	6.09	6.41	5.47
SARS	6.09	6.67	5.40
SGCA	6.09	6.46	5.69
SLC31A1	6.09	6.62	5.69
RBCK1	6.09	6.35	5.87
ELOVL5	6.09	6.39	5.55

ITGB3	6.09	7.00	5.53
PPP4R2	6.09	6.56	5.58
PPP1R11	6.09	6.38	5.73
TCERG1	6.09	6.38	5.59
SLC35D1	6.09	6.37	5.72
DLST	6.09	6.60	5.45
STX10	6.09	6.40	5.74
ITGA5	6.09	6.83	5.28
RERG-AS1	6.09	6.69	5.51
SIK3-IT1	6.09	6.84	5.46
MIR4688	6.09	6.42	5.65
MED21	6.09	6.47	5.48
OTTHUMG00000002204	6.09	6.57	5.41
PAF1	6.09	6.34	5.74
SEPHS2	6.09	6.44	5.65
HMOX2	6.09	6.52	5.55
STXBP1	6.09	6.36	5.40
DYRK1A	6.09	6.43	5.53
CIDEB	6.09	6.28	5.85
ARMCX4	6.09	6.49	5.77
KPNA3	6.09	6.70	5.57
PRDM5	6.09	6.43	5.66
FNBP1L	6.09	6.74	5.33
FSTL3	6.09	6.66	5.74
FAM134B	6.09	7.05	5.49
AGO3	6.09	6.43	5.28
MTHFD1L	6.09	6.75	5.31
GSR	6.09	6.99	5.25
FNTA	6.09	6.30	5.76
GIGYF2	6.09	6.34	5.72
NNT	6.09	6.44	5.52
ACSL4	6.09	6.74	5.47
DNAJB4	6.08	6.48	5.27
PPP2R5E	6.08	6.83	5.61
FLJ20021	6.08	6.42	5.76
BCAS2	6.08	6.62	5.33
FOXD4L6	6.08	6.48	5.80
C1orf52	6.08	6.42	5.51
ZNRD1	6.08	6.27	5.58
KIF3B	6.08	6.47	5.63
STUB1	6.08	6.53	5.51
MRPS31	6.08	6.34	5.77
C16orf80	6.08	6.74	5.45
KRTAP5-5	6.08	6.52	5.46
CNTROB	6.08	6.50	5.82
CREBL2	6.08	6.45	5.77
FLYWCH2	6.08	6.33	5.77
CD58	6.08	6.52	5.27
AKIRIN1	6.08	6.32	5.63

CPAMD8	6.08	6.62	4.73
MRPL1	6.08	6.65	5.57
ZBTB7A	6.08	6.29	5.71
C10orf118	6.08	6.45	5.39
EAPP	6.08	6.49	5.62
DDX54	6.08	6.48	5.67
NLGN2	6.08	6.54	5.59
FAM208A	6.08	6.57	5.60
RERE	6.08	6.36	5.74
NUP85	6.08	6.32	5.64
EIF2AK1	6.08	6.54	5.58
SFRP4	6.08	6.88	4.69
FBXL14	6.08	6.46	5.34
WDR11	6.08	6.52	5.23
OTTHUMG00000183288	6.08	6.54	5.63
KIRREL-IT1	6.08	6.58	5.35
CUL3	6.08	6.64	5.44
USP25	6.08	6.52	5.38
CDK9	6.08	6.34	5.42
LMOD1	6.08	6.51	5.71
TMED7	6.08	6.61	5.38
DHX29	6.08	6.56	5.56
MAP2K1	6.08	6.47	5.71
RAC2	6.08	6.45	5.63
MAGEF1	6.07	6.26	5.82
OGDH	6.07	6.42	5.28
OTTHUMG00000154838	6.07	7.91	4.66
MUS81	6.07	6.37	5.61
ARL13B	6.07	6.57	5.51
PGM2	6.07	6.47	5.61
LRIG2	6.07	6.47	5.27
DDX52	6.07	6.45	5.62
PRKAR2A	6.07	6.68	5.51
S100A9	6.07	7.35	4.91
FUBP3	6.07	6.43	5.62
WDR5	6.07	6.37	5.65
SENP5	6.07	6.57	5.48
ACBD6	6.07	6.44	5.48
ARAP1-AS1	6.07	6.46	5.60
DEFB128	6.07	6.72	5.53
RSBN1L	6.07	6.47	5.60
MIR4677	6.07	6.37	5.55
PCED1A	6.07	6.40	5.73
DAP3	6.07	6.36	5.66
MRS2	6.07	6.36	5.61
SMR3A	6.07	6.60	5.56
RPS6KA3	6.07	6.79	5.03
MRC2	6.07	6.65	5.74
COA4	6.07	6.36	5.41

SPRR2A	6.07	6.58	5.68
COMMD3	6.07	6.72	5.20
STX12	6.07	6.41	5.48
LOC100132707	6.07	6.53	5.84
PIGU	6.07	6.46	5.51
FTH1	6.07	6.60	5.63
C11orf54	6.07	6.39	5.47
OTTHUMG00000154695	6.07	6.45	5.71
PLD3	6.07	6.38	5.70
LINC00085	6.07	6.39	5.75
MRPS34	6.07	6.65	5.49
MIR1227	6.07	6.30	5.73
GRAPL	6.07	6.64	5.54
SEMA5A	6.07	6.61	5.35
FAM115A	6.07	6.45	5.60
DDX12P	6.07	6.44	5.52
PSMD6-AS2	6.07	6.49	5.52
MRPS35	6.07	6.70	4.94
HNRNPUL2	6.07	6.41	5.54
MSLNL	6.07	6.63	5.71
CABIN1	6.07	6.36	5.74
DDHD2	6.07	6.46	5.40
HEATR1	6.06	6.43	5.72
LHB	6.06	6.49	5.79
CRLF1	6.06	7.36	5.23
RANBP3	6.06	6.25	5.60
CALM2	6.06	6.38	5.61
RUSC1-AS1	6.06	6.39	5.72
SDCBP	6.06	6.53	5.24
CIB1	6.06	6.71	5.32
ATXN3	6.06	6.46	5.22
SEC23IP	6.06	6.65	5.67
DPH3	6.06	6.34	5.62
CDC37L1	6.06	6.46	5.50
PURB	6.06	6.54	5.51
OTTHUMG00000171842	6.06	6.64	5.78
MIR4294	6.06	6.60	5.52
RAB20	6.06	6.40	5.60
SDF2L1	6.06	6.40	5.71
XRCC6	6.06	6.37	5.58
OPHN1	6.06	6.64	5.25
IFT172	6.06	6.38	5.71
MAN2B2	6.06	6.35	5.72
ZNF622	6.06	6.56	5.75
POLR3D	6.06	6.51	5.60
UBE2Q1	6.06	6.26	5.80
C19orf10	6.06	6.74	5.43
DIRC2	6.06	6.46	5.62
CA9	6.06	7.38	5.25

MRP63	6.06	6.32	5.66
CLYBL	6.06	6.60	5.66
ANKAR	6.06	6.42	5.56
ATRN	6.06	6.54	5.39
SH3BP5	6.06	6.35	5.52
ID1	6.06	6.90	5.54
COLGALT1	6.06	6.48	5.52
AGA	6.06	6.52	5.46
CPXM1	6.06	7.49	4.88
POLR2G	6.06	6.48	5.40
MRPS30	6.06	6.49	5.46
LOC550643	6.06	6.36	5.25
SUPT5H	6.06	6.29	5.59
RRP36	6.06	6.54	5.40
SNX1	6.06	6.30	5.51
GSTM5	6.06	7.05	5.41
PIEZO2	6.06	6.46	5.16
TMEM64	6.06	6.38	5.85
OTTHUMG00000169385	6.06	6.53	5.63
MUC20	6.06	6.74	5.32
FAM78B	6.06	6.37	5.54
LOC401127	6.06	6.56	5.82
DNM1P46	6.06	6.47	5.64
SGK196	6.06	6.30	5.66
CC2D2A	6.06	6.43	5.77
SLC6A8	6.06	6.40	5.59
ABL1	6.05	6.52	5.64
METTL14	6.05	6.34	5.62
CASD1	6.05	6.47	5.67
ASH2L	6.05	6.37	5.33
LINC00891	6.05	6.44	5.43
PERP	6.05	6.44	5.49
CCDC84	6.05	6.16	5.87
NINJ2	6.05	6.28	5.81
CXCR4	6.05	7.97	5.19
USP11	6.05	6.27	5.77
FAM195B	6.05	6.60	5.71
FPR3	6.05	7.96	4.61
PABPC4	6.05	6.44	5.53
FAM208B	6.05	6.31	5.63
RPF2	6.05	6.75	5.42
CEP192	6.05	6.38	5.56
SPICE1	6.05	6.47	5.51
CACHD1	6.05	6.64	5.41
CISD1	6.05	6.61	5.63
ARHGEF1	6.05	6.28	5.72
RASSF8	6.05	6.57	5.69
TAF9	6.05	6.48	5.46
SIAH2-AS1	6.05	6.26	5.89

PPP1R7	6.05	6.56	5.52
USP1	6.05	6.68	5.68
SF3A1	6.05	6.42	5.76
DLK2	6.05	6.42	5.76
GANC	6.05	6.55	5.40
NOL3	6.05	6.38	5.74
VGLL4	6.05	6.56	5.58
ACTR3B	6.05	6.24	5.82
APEX1	6.05	6.40	5.54
SUMO1	6.05	6.41	5.68
ZCCHC14	6.05	7.06	4.81
C16orf70	6.05	6.50	5.36
TRAV12-2	6.05	6.43	5.49
ARRB1	6.05	6.54	5.46
RER1	6.05	6.40	5.30
ZNF28	6.05	6.52	5.62
RPL26L1	6.05	6.65	5.50
SRPRB	6.05	6.57	5.48
HDAC1	6.05	6.39	5.59
SNORA45	6.05	6.44	5.37
LOC100128494	6.05	6.31	5.53
NSMCE4A	6.05	6.30	5.46
COPS7B	6.05	6.29	5.43
PAPD5	6.05	6.53	5.08
DDX49	6.05	6.25	5.70
EXOSC7	6.05	6.54	5.63
ZDHHC16	6.05	6.34	5.55
RPL35	6.05	6.26	5.84
KPNA6	6.05	6.47	5.74
COL16A1	6.04	6.60	5.45
HMGXB4	6.04	6.63	5.77
KLF7	6.04	7.00	5.10
ECE1	6.04	6.44	5.34
LOC145783	6.04	6.52	5.46
C9orf3	6.04	6.45	5.53
LOC439994	6.04	6.60	5.70
RNU1-23P	6.04	6.86	5.48
KLHL42	6.04	6.84	5.37
PUM1	6.04	6.51	5.24
SPRY2	6.04	6.39	5.42
ATP13A3	6.04	6.58	5.43
CKS1B	6.04	6.22	5.78
KCNT2	6.04	6.52	4.80
CHTF8	6.04	6.63	5.29
MIR210HG	6.04	6.64	5.45
LTBP3	6.04	6.46	5.66
DEDD	6.04	6.40	5.77
GCC2	6.04	6.33	5.57
WRN	6.04	6.46	5.56

ZNF286B	6.04	6.40	5.70
NPR2	6.04	6.52	5.75
ERH	6.04	6.34	5.66
ZNF621	6.04	6.32	5.32
CSPP1	6.04	6.43	5.69
FAF1	6.04	6.48	5.74
ZDHHC12	6.04	6.39	5.59
ARMCX3	6.04	6.47	5.36
DDB2	6.04	6.49	5.18
TRIM4	6.04	6.33	5.54
CDYL	6.04	6.27	5.83
WEE1	6.04	6.74	5.51
RNA5SP151	6.04	6.58	5.59
ACTL6A	6.04	6.62	5.53
UBLCP1	6.04	6.60	5.24
SSBP3	6.04	6.25	5.63
OTTHUMG00000172117	6.04	6.54	5.66
USP14	6.04	6.72	5.40
ARMC10	6.04	6.66	5.56
R3HCC1	6.04	6.54	5.58
ZNF526	6.04	6.20	5.76
OTTHUMG00000170338	6.04	6.45	5.56
ADAM19	6.04	6.62	5.75
PTPRJ	6.04	6.88	5.54
FAM127B	6.04	6.48	5.40
TPD52L1	6.04	6.30	5.68
PAK2	6.03	6.60	5.33
SERPINH1	6.03	6.51	5.48
MIR3941	6.03	6.99	5.32
SLC7A5P1	6.03	6.33	5.28
LOC100132832	6.03	6.95	5.69
SMAD9-AS1	6.03	6.75	5.57
NAGK	6.03	6.86	5.12
SNORD114-21	6.03	7.50	5.28
FOXO3	6.03	6.43	5.42
MSH5	6.03	6.42	5.73
NUP188	6.03	6.46	5.67
REV1	6.03	6.36	5.17
CPSF7	6.03	6.51	5.55
METTL3	6.03	6.37	5.38
HYOU1	6.03	6.35	5.55
RRAGD	6.03	6.46	5.48
RPL23AP64	6.03	6.63	5.76
TMEM53	6.03	6.29	5.65
MFF	6.03	6.44	5.46
TOR1A	6.03	6.55	5.55
UXS1	6.03	6.21	5.76
DCTN1	6.03	6.36	5.58
M6PR	6.03	6.39	5.64

PRC1	6.03	6.63	5.44
PDHA1	6.03	6.20	5.74
RC3H2	6.03	6.61	5.57
HYI	6.03	6.54	5.76
PMS2P4	6.03	6.44	5.73
CISD2	6.03	6.53	5.30
MPI	6.03	6.51	5.77
FAM90A7P	6.03	6.42	5.85
ATG7	6.03	6.26	5.70
TMEM237	6.03	6.27	5.65
KANK3	6.03	6.71	5.33
C14orf2	6.03	6.28	5.64
ADD3	6.03	6.63	5.12
TAOK3	6.03	6.44	5.46
MZT1	6.03	6.64	5.46
ETFDH	6.03	6.54	5.73
TAF2	6.03	6.37	5.21
MLLT11	6.03	6.58	5.67
MPPE1	6.03	6.67	5.38
HOXB2	6.03	6.65	5.70
MIR943	6.03	6.24	5.79
PRR12	6.03	6.41	5.68
SLC25A27	6.03	6.46	5.13
ANKRD52	6.03	6.52	5.52
SLC11A2	6.03	6.36	5.58
PROSER1	6.03	6.74	5.40
KLHL20	6.03	6.49	5.57
KIR3DL2	6.03	6.54	5.53
RAB9A	6.03	6.73	5.44
OTTHUMG00000041435	6.03	6.45	5.31
MRPS17	6.03	6.58	5.60
DHRS3	6.02	6.54	5.37
FOLR2	6.02	6.85	4.88
MIR181D	6.02	6.53	5.76
SART3	6.02	6.35	5.62
ACCS	6.02	6.52	5.65
CRYZL1	6.02	6.44	5.61
LOC651959	6.02	6.71	5.51
OVOL2	6.02	6.64	5.50
HMGA1	6.02	6.33	5.31
DNAJC14	6.02	6.45	5.60
EPN2	6.02	6.43	5.35
CHST15	6.02	6.60	5.19
ARV1	6.02	6.27	5.42
LYPLAL1	6.02	6.48	5.42
UBE2Q2	6.02	7.00	5.28
SNRPB	6.02	6.57	5.54
CCDC94	6.02	6.42	5.57
AKAP8	6.02	6.22	5.66

BABAM1	6.02	6.42	5.61
MIR4288	6.02	6.63	5.33
PXMP2	6.02	6.12	5.83
OTTHUMG00000163795	6.02	6.71	5.64
STRN	6.02	6.37	5.30
DCAF5	6.02	6.49	5.26
CCND2	6.02	6.27	5.73
CUL4B	6.02	6.40	5.44
MIR3178	6.02	6.28	5.76
TVP23C-CDRT4	6.02	6.63	5.50
ATP2C1	6.02	6.46	5.14
FCF1	6.02	6.73	5.25
DENR	6.02	6.55	5.52
WASF3	6.02	6.41	5.72
LOC100507580	6.02	6.79	3.54
NHP2	6.02	6.29	5.58
LOC284191	6.02	6.36	5.67
G6PC3	6.02	6.56	5.41
GMEB1	6.02	6.32	5.71
NFXL1	6.02	6.31	5.57
BCL10	6.02	6.51	5.33
WDR77	6.02	6.32	5.53
PSMD2	6.02	6.47	5.74
FAM105B	6.02	6.41	5.43
GSTK1	6.02	6.49	5.47
RNU7-72P	6.02	6.61	5.61
CHST3	6.02	6.46	5.68
ASXL1	6.02	6.31	5.51
ANKRD26	6.02	6.49	5.61
LOC100506302	6.02	6.50	5.66
SLC25A30	6.02	6.38	5.27
KANSL1	6.02	6.50	5.31
VPS13A	6.02	6.46	5.34
TEX10	6.02	6.31	5.55
WBSCR22	6.02	6.17	5.86
CDK14	6.02	6.33	5.48
MRPS11	6.02	6.36	5.68
PLEKHA4	6.02	6.38	5.64
SLU7	6.02	6.44	5.44
APOL6	6.02	6.54	5.25
TBX4	6.02	6.48	5.33
PSMG3	6.02	6.32	5.64
C10orf11	6.02	6.64	5.52
ZYX	6.02	6.40	5.49
LOC100131541	6.02	6.51	5.61
MGC24103	6.02	7.43	4.71
NCK1	6.01	6.41	5.60
CHTOP	6.01	6.32	5.53
MTA2	6.01	6.35	5.68

HSPB6	6.01	6.62	5.63
MFSD10	6.01	6.32	5.80
TNFRSF21	6.01	6.55	5.65
B3GNT2	6.01	6.28	5.40
SULT1A2	6.01	6.52	5.55
PPIL3	6.01	6.35	5.48
MMGT1	6.01	6.73	4.86
SNHG12	6.01	6.49	5.46
CGGBP1	6.01	6.39	5.36
HK1	6.01	6.40	5.82
MSRB2	6.01	6.28	5.62
MIR665	6.01	6.28	5.54
ZNF623	6.01	6.47	5.73
MIR105-1	6.01	6.48	5.49
KRT16P3	6.01	6.97	5.52
KIAA0754	6.01	6.37	5.44
SIK3	6.01	6.42	5.38
HCG9	6.01	6.33	5.64
EXOC5	6.01	6.53	5.41
LOC441426	6.01	6.88	5.65
TMBIM4	6.01	6.23	5.54
PCSK1N	6.01	6.42	5.66
DCAKD	6.01	6.50	5.39
TRIM13	6.01	6.31	5.45
RSPRY1	6.01	6.69	5.39
POLR1E	6.01	6.19	5.58
NCOR2	6.01	6.27	5.35
C10orf76	6.01	6.58	5.48
EI24	6.01	6.38	5.81
CWC22	6.01	6.70	5.42
TOP3A	6.01	6.29	5.44
NAB1	6.01	6.42	5.56
DOCK9	6.01	6.41	5.26
SGTB	6.01	6.63	5.61
COPS4	6.01	6.59	5.39
NINJ1	6.01	6.44	5.37
LOC283335	6.01	6.36	5.67
LOC100293044	6.01	6.51	5.66
SF3A2	6.01	6.45	5.67
LOC100506217	6.01	7.13	4.81
OTTHUMG00000171012	6.01	6.36	5.79
STRC	6.01	6.61	5.53
BTBD2	6.01	6.51	5.54
RSF1	6.01	6.33	5.07
PCDH18	6.01	6.76	4.73
PCID2	6.01	6.46	5.38
SFSWAP	6.01	6.25	5.59
GOSR2	6.01	6.36	5.61
SDHB	6.01	6.38	5.62

MRPS15	6.01	6.22	5.56
NDUFS3	6.01	6.35	5.55
MIR4731	6.01	6.65	5.35
LINC00094	6.01	6.39	5.60
THAP1	6.01	6.57	5.59
ADAMTS9-AS2	6.01	6.32	5.53
NDST2	6.01	6.31	5.47
MITD1	6.01	6.43	5.41
BTN2A1	6.01	6.46	5.46
MIR4638	6.01	6.47	5.78
HCST	6.01	6.45	5.77
NUMB	6.00	6.29	5.72
PAR-SN	6.00	6.38	5.75
PSME2	6.00	6.25	5.71
PIP4K2C	6.00	6.30	5.65
OSBPL3	6.00	6.75	5.30
FOXN2	6.00	6.40	5.45
PRMT2	6.00	6.56	5.28
ZFP36L1	6.00	6.26	5.71
CSPG4	6.00	6.15	5.78
SUZ12	6.00	6.52	5.47
GJC1	6.00	6.66	5.57
EXOC4	6.00	6.31	5.53
SAMD10	6.00	6.48	5.53
ERICH1	6.00	6.39	5.71
SLC9A3R2	6.00	6.32	5.71
TOX4	6.00	6.32	5.79
TMA16	6.00	6.47	5.55
VWA9	6.00	6.51	5.32
PCNT	6.00	6.23	5.46
STAG3L2	6.00	6.35	5.45
PSMC4	6.00	6.36	5.47
MAPK9	6.00	6.28	5.44
MIR4642	6.00	6.30	5.76
TAOK2	6.00	6.20	5.78
VCPIP1	6.00	6.41	5.17
DUSP10	6.00	6.77	4.91
CRYAB	6.00	6.46	5.61
SLC2A4RG	6.00	6.31	5.74
NLRP1	6.00	6.33	5.70
MED16	6.00	6.32	5.62
WBP2	6.00	6.33	5.41
TCF3	6.00	6.18	5.83
RNA5SP256	6.00	6.56	5.19
C4B	6.00	6.43	5.74
DCUN1D4	6.00	6.46	5.36
OTTHUMG00000149885	6.00	6.41	5.64
MIR23B	6.00	6.49	5.24
TBC1D3B	6.00	6.29	5.69

DPY19L4	6.00	6.41	5.60
TEKT4P2	6.00	6.50	5.49
ZSCAN2	6.00	6.21	5.74
LOC100652768	6.00	6.34	5.61
ISM2	6.00	6.67	5.50
AK4	6.00	6.39	5.56
TMEM120A	6.00	6.34	5.70
RSBN1	5.99	6.25	5.74
ANKDD1A	5.99	6.27	5.71
CREB5	5.99	6.39	5.57
ITFG2	5.99	6.39	5.62
XPO5	5.99	6.53	5.59
LILRB4	5.99	6.54	5.29
PRKAG1	5.99	6.31	5.34
GCSHP3	5.99	6.39	5.36
TBC1D4	5.99	6.28	5.31
NDUFB9	5.99	6.26	5.42
AGO2	5.99	6.22	5.65
POU2F1	5.99	6.24	5.64
MCM6	5.99	6.53	5.44
MYL12A	5.99	6.40	5.55
IGKV5-2	5.99	6.63	5.23
DHX8	5.99	6.32	5.56
SLC35A1	5.99	6.33	5.66
RNU7-45P	5.99	6.48	5.48
GXYLT2	5.99	7.51	4.34
ZSWIM8	5.99	6.27	5.25
IKZF5	5.99	6.67	5.14
SCAMP3	5.99	6.51	5.56
ADHFE1	5.99	6.76	5.33
RLIM	5.99	6.60	5.34
ZNF655	5.99	6.34	5.30
PCGF5	5.99	6.41	5.45
EML1	5.99	6.74	5.27
TRAJ48	5.99	6.82	5.66
CHD6	5.99	6.25	5.54
ZNF414	5.99	6.31	5.68
HSPE1-MOB4	5.99	6.29	5.53
TMEM184B	5.99	6.40	5.60
ZFP112	5.99	6.25	5.76
TCP11L2	5.99	6.48	5.13
GALK1	5.99	6.32	5.75
JAM3	5.99	6.29	5.22
SYT11	5.99	7.01	5.24
GRB2	5.99	6.52	5.55
PEX3	5.99	6.29	5.21
LOC100130428	5.99	6.71	5.61
TUT1	5.99	6.29	5.63
GIT2	5.99	6.42	5.36

HEATR5A	5.99	6.40	5.75
PPP2R2D	5.99	6.56	5.39
WDR61	5.99	6.27	5.63
TSR2	5.99	6.61	4.94
SP2	5.99	6.19	5.73
SCARNA22	5.99	6.24	5.68
GUCA2B	5.99	6.58	5.27
TIAM2	5.99	6.51	5.51
ASNSD1	5.99	6.67	5.31
PDCD7	5.98	6.23	5.53
CEP170	5.98	6.60	5.25
OTTHUMG00000008920	5.98	7.08	4.25
TMEM220	5.98	6.27	5.53
DNPH1	5.98	6.25	5.79
ELAVL1	5.98	6.40	5.57
CPED1	5.98	6.47	5.28
ZNF264	5.98	6.51	5.58
KIF26A	5.98	6.44	5.75
NFKB1	5.98	6.48	5.45
SGK110	5.98	6.32	5.71
FAM13B	5.98	6.27	5.55
KIAA2013	5.98	6.23	5.61
CHMP4A	5.98	6.40	5.47
GSTT1	5.98	6.51	5.82
DGCR11	5.98	6.58	5.51
OR2T12	5.98	6.38	5.66
VPS13D	5.98	6.44	5.32
HADH	5.98	6.28	5.51
HDAC8	5.98	6.40	5.26
FAM20C	5.98	6.37	5.43
SAMD4B	5.98	6.22	5.67
RAI2	5.98	6.40	5.46
SIGLEC1	5.98	6.34	5.54
PILRA	5.98	7.04	5.60
NAP1L4	5.98	6.43	5.23
MTERFD2	5.98	6.45	5.57
TM4SF1	5.98	6.51	5.08
POMGNT1	5.98	6.21	5.73
ITGA9	5.98	6.18	5.76
PPM1G	5.98	6.52	5.06
PLEKHM2	5.98	6.31	5.59
KIAA0391	5.98	6.48	5.29
ASMTL-AS1	5.98	6.18	5.74
SSFA2	5.98	6.67	5.44
ZNF606	5.98	6.23	5.40
RPA2	5.98	6.35	5.57
COPS5	5.98	6.26	5.66
OPA1	5.98	6.65	5.45
EP400	5.98	6.24	5.34

PLIN5	5.98	6.25	5.77
NDUFAF2	5.98	6.41	5.47
LOC100129034	5.98	6.57	5.16
SLC7A2	5.98	6.47	5.29
ZSWIM7	5.98	6.37	4.87
SEBOX	5.98	6.49	5.50
KRAS	5.98	6.49	5.42
PSMG2	5.98	6.35	5.09
OCEL1	5.98	6.22	5.78
CECR6	5.98	6.20	5.63
SPPL3	5.98	6.30	5.43
OSBPL11	5.98	6.59	5.41
PGRMC1	5.98	6.57	5.30
BCCIP	5.98	6.56	5.34
LIMD1	5.97	6.32	5.66
OGFR-AS1	5.97	6.38	5.54
CARHSP1	5.97	6.50	5.63
ACOT9	5.97	6.61	5.32
HNRNPH1	5.97	6.15	5.71
RECK	5.97	6.27	5.64
SPI1	5.97	6.32	5.52
IGLV3-1	5.97	6.62	5.56
WDR27	5.97	6.25	5.60
ATG14	5.97	6.24	5.70
TOMM22	5.97	6.56	5.52
HNRNPF	5.97	6.28	5.62
DDX41	5.97	6.28	5.51
HM13	5.97	6.32	5.53
UNK	5.97	6.39	5.35
LHFPL2	5.97	6.48	5.15
ADSL	5.97	6.24	5.55
ATAD5	5.97	6.31	5.52
TARS	5.97	6.83	5.29
SCRG1	5.97	6.41	5.29
FAM175B	5.97	6.40	5.16
OTTHUMG00000156011	5.97	7.42	4.73
SLC2A10	5.97	6.37	5.41
OTTHUMG00000016049	5.97	6.80	5.21
GLTSCR1L	5.97	6.35	5.74
SLC1A4	5.97	6.43	5.30
RNA5SP450	5.97	6.48	5.11
GABPA	5.97	6.52	5.54
GBAP1	5.97	6.42	5.54
CYFIP1	5.97	6.53	5.36
C5orf56	5.97	6.55	5.38
GLRX	5.97	6.35	5.34
FBRS	5.97	6.35	5.62
CMKLR1	5.97	6.27	5.20
NT5C3B	5.97	6.49	5.26

TMEM175	5.97	6.21	5.56
TRMT112	5.97	6.45	5.55
MIR1301	5.97	6.53	5.56
ZNF43	5.97	6.51	5.38
NRP2	5.97	6.99	5.16
KAT6B	5.97	6.54	5.63
FAM149B1	5.97	6.23	5.39
ATG4D	5.97	6.15	5.75
IGLJ3	5.97	7.75	5.25
FAM43A	5.97	6.40	5.49
C20orf197	5.97	6.19	5.75
LRBA	5.97	6.26	5.54
EIF4A3	5.97	6.40	5.40
CIZ1	5.97	6.33	5.41
EXOC3	5.97	6.10	5.75
FBXW8	5.97	6.30	5.49
GPR153	5.97	6.26	5.45
MAPKAPK3	5.97	6.23	5.67
TTLL4	5.97	6.63	5.53
CD33	5.97	6.32	5.42
MAP1A	5.97	6.66	4.87
PPME1	5.97	6.48	5.26
SUCLA2	5.97	6.47	5.27
ATIC	5.97	6.37	5.43
WWC2	5.97	6.41	5.56
SPAST	5.97	6.48	4.86
NKX3-1	5.97	6.24	5.65
HDAC7	5.97	6.24	5.72
MCCC2	5.96	6.49	5.30
ATP8B2	5.96	6.36	5.40
LOXL2	5.96	7.14	5.10
PDGFA	5.96	6.59	5.40
SEPT7	5.96	6.29	5.52
GSTM2P1	5.96	6.60	5.32
PRAP1	5.96	6.47	5.53
N4BP2	5.96	6.27	5.42
GGT5	5.96	6.50	5.45
DEGS2	5.96	6.42	5.35
DHX40	5.96	6.43	5.24
SOX8	5.96	6.58	5.50
LINC00852	5.96	6.26	5.50
PRKRIR	5.96	6.37	5.62
UTP18	5.96	6.50	5.42
HTT	5.96	6.37	5.45
ERAP2	5.96	6.62	5.18
NAPA	5.96	6.23	5.63
ARAF	5.96	6.31	5.70
FLJ43663	5.96	6.41	5.23
SLC25A26	5.96	6.27	5.60

RPAP2	5.96	6.42	5.42
ZNF397	5.96	6.52	5.51
BMPER	5.96	6.49	5.37
ACTB	5.96	6.25	5.60
SESN1	5.96	6.44	5.31
NARG2	5.96	6.37	5.56
ARMC8	5.96	6.38	5.63
ZXDB	5.96	6.80	4.86
MED1	5.96	6.32	5.52
DUSP16	5.96	6.32	5.66
NGFRAP1	5.96	6.21	5.78
WDR31	5.96	6.43	5.34
CAPN10	5.96	6.84	5.56
RNU7-77P	5.96	6.59	5.45
SNN	5.96	6.37	5.56
SEMA3D	5.96	6.65	5.20
MAGED4	5.96	6.50	5.60
XPNPEP3	5.96	6.41	5.63
PTPDC1	5.96	6.30	5.42
NCR2	5.96	6.40	5.38
RNPEP	5.96	6.14	5.74
PTGFRN	5.96	6.88	5.33
OSBPL2	5.96	6.30	5.49
WDR59	5.96	6.43	5.54
RNF135	5.96	6.70	5.35
AKAP11-IT1	5.96	6.33	5.32
BCL9	5.96	6.37	5.66
ARL5B	5.96	6.31	5.50
BBX	5.96	6.21	5.73
TFCP2	5.96	6.48	5.31
ZZZ3	5.96	6.36	5.20
LOC653562	5.96	6.90	5.15
DOCK4	5.96	6.25	5.56
DDAH1	5.96	6.51	5.18
SLC12A4	5.96	6.25	5.55
PROM2	5.96	6.48	5.59
WARS	5.96	6.24	5.69
TSN	5.96	6.40	5.51
TMEM66	5.96	6.30	5.63
TMEM41A	5.96	6.23	5.39
LRG1	5.95	6.12	5.71
LCP1	5.95	6.82	5.54
C19orf66	5.95	6.37	5.61
ADAMTSL3	5.95	6.59	5.20
PTPN4	5.95	6.30	5.66
NDUFA6	5.95	6.49	5.35
MATN2	5.95	6.61	5.27
NHLRC2	5.95	6.26	5.44
CCDC41	5.95	6.56	5.21

BBS7	5.95	6.25	5.64
RALY	5.95	6.29	5.25
TMEM8B	5.95	6.22	5.80
PREX1	5.95	6.52	5.33
ZBTB4	5.95	6.38	5.56
XPO7	5.95	6.51	5.31
LYPLA2	5.95	6.44	5.61
FAM201A	5.95	6.40	5.44
C9orf114	5.95	6.40	5.41
ARID5B	5.95	6.49	5.47
PDLIM3	5.95	7.26	4.85
MYLIP	5.95	6.59	5.31
BCL2L12	5.95	6.31	5.68
PPP1R12A	5.95	6.44	5.44
LOC646329	5.95	6.50	5.42
RNF144A	5.95	6.59	5.46
EDEM3	5.95	6.43	5.41
RAP1GDS1	5.95	6.44	5.58
TBC1D3F	5.95	6.18	5.66
RAB5B	5.95	6.21	5.56
ETV6	5.95	6.46	5.44
ERO1L	5.95	7.24	4.94
FSCN1	5.95	6.67	5.33
PDCD11	5.95	6.37	5.44
CLPP	5.95	6.38	5.49
INSIG2	5.95	6.45	5.28
GIMAP4	5.95	6.78	5.01
KIAA0141	5.95	6.24	5.57
REXO1	5.95	6.33	5.66
KIAA1731	5.95	6.25	5.58
PRAMEF10	5.95	6.61	5.23
ZNF93	5.95	6.30	5.62
PLEKHA8P1	5.95	6.15	5.59
PKI55	5.95	6.29	5.68
MGC34800	5.95	6.86	5.50
MIR631	5.95	6.48	5.41
MROH6	5.95	6.12	5.63
LDHA	5.95	6.72	5.20
NPC1	5.95	7.35	5.05
GNAS	5.95	6.14	5.76
CNOT2	5.95	6.36	5.47
RARRES3	5.95	6.71	5.49
RORA	5.95	6.47	5.49
KDSR	5.95	6.50	5.27
LOC285638	5.95	6.48	4.74
USP40	5.95	6.24	5.77
IDS	5.95	6.65	5.04
TSPAN32	5.95	6.24	5.69
CD70	5.95	6.34	5.39

PHTF1	5.94	6.27	5.15
MRPL13	5.94	6.85	4.85
LTBR	5.94	6.22	5.43
DOK2	5.94	6.30	5.47
PPP2R5C	5.94	6.38	4.76
CLN5	5.94	6.43	5.45
CHSY3	5.94	6.65	5.23
DICER1-AS1	5.94	6.25	5.52
MIR412	5.94	6.52	5.51
OR4N2	5.94	7.13	4.60
MINK1	5.94	6.37	5.56
ZNF574	5.94	6.39	5.54
MAP3K12	5.94	6.29	5.60
STAT6	5.94	6.17	5.73
ZNF827	5.94	6.47	5.08
NELFE	5.94	6.24	5.52
RUNX2	5.94	6.56	5.09
KIAA0196	5.94	6.52	5.13
RNA5SP258	5.94	6.42	5.58
IFI44	5.94	7.16	5.27
DROSHA	5.94	6.32	5.58
MYO15B	5.94	6.19	5.64
PPP1CA	5.94	6.44	5.41
COL4A3BP	5.94	6.56	5.00
ATMIN	5.94	6.14	5.37
SIAH2	5.94	6.31	5.31
DHPS	5.94	6.38	5.51
HIPK1	5.94	6.49	5.11
FAM101B	5.94	6.51	5.42
SYNRG	5.94	6.50	5.28
RNA5SP427	5.94	6.51	5.42
SLC29A3	5.94	6.59	5.20
MIR2467	5.94	6.31	5.60
KIAA0146	5.94	6.22	5.68
KIAA0319L	5.94	6.20	5.61
PANK2	5.94	6.32	5.46
CUTA	5.94	6.31	5.44
COQ4	5.94	6.30	5.43
RNA5SP482	5.94	6.57	5.02
OTTHUMG00000016583	5.94	6.41	5.44
SNORA10	5.94	6.38	5.19
AGPAT4	5.94	6.44	5.40
SMIM20	5.94	6.30	5.62
H2AFJ	5.94	6.16	5.37
HACL1	5.94	6.16	5.73
NEK4	5.94	6.24	5.36
TNKS1BP1	5.94	6.05	5.62
LOC100128644	5.94	6.25	5.56
FAM224B	5.94	6.23	5.48

KIAA0753	5.94	6.25	5.54
BTF3	5.94	6.14	5.76
HAMP	5.93	6.27	5.54
TGFB1I1	5.93	6.50	5.44
DIP2A	5.93	6.23	5.74
CRTC1	5.93	6.33	5.56
DLD	5.93	6.29	5.39
MRE11A	5.93	6.40	5.19
MLST8	5.93	6.14	5.68
CRTC2	5.93	6.24	5.57
CFLAR	5.93	6.23	5.48
SNORD100	5.93	6.57	5.36
CTC1	5.93	6.25	5.38
FLRT2	5.93	6.56	5.39
KSR1	5.93	6.13	5.59
ZNF227	5.93	6.38	5.62
CYP2D6	5.93	6.40	5.38
GTPBP4	5.93	6.38	5.30
FAM160B1	5.93	6.39	5.46
PTS	5.93	6.36	5.31
RPS4X	5.93	6.46	5.23
ZNF880	5.93	6.30	5.56
BAD	5.93	6.19	5.34
LPP-AS2	5.93	6.18	5.69
PF4	5.93	6.52	5.52
LOC441956	5.93	6.72	5.26
RNF145	5.93	6.36	5.47
ZNF141	5.93	6.57	5.58
SIPA1L3	5.93	6.37	5.61
DHRSX	5.93	6.30	5.52
ADD1	5.93	6.21	5.49
PBDC1	5.93	6.48	5.09
MIR637	5.93	6.60	5.51
NIT1	5.93	6.25	5.71
BBS9	5.93	6.45	5.40
CACUL1	5.93	6.32	5.27
MYO18A	5.93	6.09	5.74
FUT10	5.93	6.22	5.32
NOL8	5.93	6.27	5.53
NLK	5.93	6.22	5.53
NONO	5.93	6.54	5.27
PRKCDBP	5.93	6.33	5.33
LINC00539	5.93	6.80	5.13
BAZ2A	5.93	6.38	5.49
CPD	5.93	6.29	5.43
MIR4742	5.93	6.44	4.98
BIN1	5.93	6.26	5.32
MIR4450	5.93	6.26	5.45
PARM1	5.93	7.15	4.77

PPID	5.93	6.62	5.34
GTPBP2	5.93	6.14	5.57
EAF1	5.93	6.20	5.47
METTL17	5.93	6.25	5.60
KLHL28	5.93	6.16	5.60
GRAMD3	5.93	6.27	5.49
C17orf82	5.93	6.61	5.49
MYO9B	5.93	6.24	5.35
CCDC82	5.93	6.54	5.03
SOX18	5.93	6.60	5.24
PAN2	5.93	6.37	5.62
LINC00641	5.92	6.51	5.47
OTTHUMG00000165259	5.92	6.32	5.50
UBQLN2	5.92	6.31	5.44
DOT1L	5.92	6.29	5.57
ZC3HAV1L	5.92	6.45	5.44
SZRD1	5.92	6.36	5.62
USP21	5.92	6.12	5.60
FGFR1	5.92	6.33	5.30
FAF2	5.92	6.39	5.54
FOXH1	5.92	6.25	5.64
GPR34	5.92	6.79	4.52
HIBCH	5.92	6.36	5.28
POLD2	5.92	6.45	5.60
MLKL	5.92	6.37	5.10
SAP25	5.92	6.48	5.31
SNX29P2	5.92	6.32	5.54
MYOZ3	5.92	6.22	5.54
IRX3	5.92	6.24	5.63
LARP1B	5.92	6.26	5.41
NUDT19	5.92	6.38	5.27
PDHX	5.92	6.09	5.18
APMAP	5.92	6.19	5.57
UPF2	5.92	6.25	5.24
OTTHUMG00000171477	5.92	6.80	4.98
GPN1	5.92	6.38	5.47
KIAA2018	5.92	6.16	5.29
OAZ2	5.92	6.24	5.44
SPTLC2	5.92	6.49	5.29
DNAJB14	5.92	6.34	5.53
SPOP	5.92	6.36	5.48
PEX1	5.92	6.43	5.60
ATP6V0D1	5.92	6.40	5.25
ABCE1	5.92	6.52	5.24
BTBD1	5.92	6.45	5.29
EIF5	5.92	6.35	5.21
CEP290	5.92	6.34	5.30
LOC400558	5.92	6.44	5.48
BLOC1S3	5.92	6.23	5.59

BECN1	5.92	6.36	5.28
DIS3L2	5.92	6.20	5.75
OSBPL1A	5.92	6.41	5.17
ASL	5.92	6.22	5.80
FBXO28	5.92	6.50	5.21
BRAP	5.92	6.21	5.50
GPATCH11	5.92	6.32	5.54
LOC100506276	5.92	6.16	5.61
CRAT	5.92	6.25	5.48
GLDN	5.92	6.62	5.08
DLEU2	5.92	6.65	4.99
NAA30	5.92	6.17	5.54
ABHD10	5.92	6.21	5.55
C21orf15	5.92	6.31	5.47
ZBTB25	5.92	6.37	5.55
ABCF3	5.91	6.18	5.42
KIAA1462	5.91	6.57	4.88
OR2T6	5.91	6.41	5.67
AAK1	5.91	6.33	5.33
BTBD6	5.91	6.29	5.58
TIMM10B	5.91	6.20	5.46
CCDC125	5.91	6.17	5.61
METTL2B	5.91	6.51	5.07
C9orf78	5.91	6.26	5.38
OTTHUMG00000171929	5.91	6.35	5.37
RGPD3	5.91	6.57	5.24
CIAO1	5.91	6.24	5.58
FBXO38	5.91	6.28	5.60
USF2	5.91	6.15	5.45
RPF1	5.91	6.35	5.52
RNF111	5.91	6.24	5.47
PHF20L1	5.91	6.36	5.43
NOLC1	5.91	6.37	5.47
PLCXD1	5.91	6.58	5.14
PPAP2B	5.91	6.47	5.27
SNHG10	5.91	6.23	5.30
UBE2G2	5.91	6.32	5.48
LOC642852	5.91	6.48	5.61
YWHAE	5.91	6.33	5.18
EIF3I	5.91	6.30	5.09
GAPVD1	5.91	6.29	5.58
IGH	5.91	7.10	5.43
TBP	5.91	6.38	5.61
RTFDC1	5.91	6.24	5.65
DNAJC1	5.91	6.63	5.21
OR8B2	5.91	7.04	5.18
TUBGCP5	5.91	6.25	5.43
C5orf24	5.91	6.37	5.28
FAM20A	5.91	6.23	5.54

ANGEL2	5.91	6.21	5.45
FAM180B	5.91	6.57	5.41
ATP11C	5.91	6.36	5.36
SLC25A24	5.91	6.41	5.26
CCL3L3	5.91	6.12	5.71
OTTHUMG00000171841	5.91	6.35	5.54
ASB16	5.91	6.18	5.57
SP100	5.91	6.31	5.49
SIX2	5.91	6.22	5.61
HIST2H4B	5.91	6.44	5.43
RQCD1	5.91	6.31	5.38
SLC39A8	5.91	6.32	5.54
RBPMS	5.91	6.68	5.50
PTGER4P2-CDK2AP2P2	5.91	6.42	5.53
PRKY	5.91	7.01	4.26
SLC37A3	5.91	6.17	5.59
TRIM74	5.91	6.50	5.45
HBP1	5.91	6.31	5.23
LPIN3	5.90	6.33	5.64
OTTHUMG00000171546	5.90	6.63	5.31
ZBTB1	5.90	6.16	5.56
MRPL50	5.90	6.30	5.47
ACPL2	5.90	6.32	5.16
GJA4	5.90	6.39	5.42
BACE1-AS	5.90	6.46	5.22
PFKP	5.90	6.61	4.54
MSI2	5.90	6.27	5.33
MERTK	5.90	7.16	5.23
PSMC6	5.90	6.46	5.44
PCYT1A	5.90	6.13	5.48
LAMTOR1	5.90	6.37	5.61
MRPS5	5.90	6.15	5.56
IFT46	5.90	6.20	5.59
ZNF773	5.90	6.13	5.67
ATXN7	5.90	6.18	5.76
GRPEL1	5.90	6.13	5.48
ODF2L	5.90	6.40	5.22
MTHFD2	5.90	6.28	5.49
HSFX1	5.90	6.36	5.63
PELI1	5.90	6.25	5.30
JUP	5.90	6.11	5.53
CCDC90B	5.90	6.17	5.40
ZFP62	5.90	6.19	5.33
STYXL1	5.90	6.28	5.58
SNX4	5.90	6.31	5.20
SENP1	5.90	6.23	5.30
GK-IT1	5.90	6.32	5.48
SH3BGRL2	5.90	6.36	5.29
ZBTB10	5.90	6.51	5.34

RAB2A	5.90	6.35	5.32
ZDHHC24	5.90	6.17	5.72
ANGPTL4	5.90	6.87	5.09
OTTHUMG00000160985	5.90	6.20	5.67
MIR577	5.90	6.54	5.39
MMS19	5.90	6.29	5.56
MIR4476	5.90	6.47	5.44
YIPF6	5.90	6.22	5.47
BCL3	5.90	6.10	5.54
FBXO2	5.90	6.43	5.52
RPS27L	5.90	6.51	5.48
ATL2	5.90	6.10	5.25
OPCML	5.90	6.58	5.53
PRKCSH	5.90	6.13	5.23
ERV3-1	5.90	6.18	5.61
SMARCA4	5.90	6.30	5.60
C3orf17	5.90	6.39	5.23
TMEM127	5.90	6.27	5.34
VPRBP	5.90	6.23	5.56
SCYL3	5.90	6.15	5.75
JTB	5.90	6.45	5.25
ANKRD39	5.90	6.27	5.52
PREB	5.90	6.05	5.62
VAMP2	5.89	6.13	5.67
MIR378H	5.89	6.58	5.06
FADS2	5.89	6.32	5.44
WDR41	5.89	6.71	5.44
LOC100128281	5.89	6.20	5.63
TTC34	5.89	6.22	5.61
C21orf2	5.89	6.06	5.48
GNG2	5.89	6.56	4.92
MIR4451	5.89	6.68	4.78
SEPT5-GP1BB	5.89	6.03	5.59
LOC100505806	5.89	6.17	5.51
BDKRB2	5.89	6.30	5.66
MIR3125	5.89	6.30	5.67
ARHGEF15	5.89	6.37	5.16
NAA40	5.89	6.21	5.57
CUX1	5.89	6.43	5.21
CD81	5.89	6.14	5.65
SLC25A32	5.89	6.37	5.45
LINC00693	5.89	6.48	5.36
GTF3C6	5.89	6.51	5.23
QSOX1	5.89	6.22	5.57
KCNK7	5.89	6.10	5.66
SYNPO	5.89	6.42	5.37
SNX30	5.89	6.27	5.29
DAAM2	5.89	6.29	5.48
ARHGEF7	5.89	6.15	5.74

EDN3	5.89	6.43	5.59
PPM1M	5.89	6.24	5.59
DCAF10	5.89	6.32	5.37
CDC25B	5.89	6.12	5.63
FAM178A	5.89	6.30	5.15
METTL22	5.89	6.14	5.52
ACD	5.89	6.32	5.64
PGA4	5.89	6.23	5.58
RAPGEF2	5.89	6.24	5.51
SPTY2D1-AS1	5.89	6.20	5.61
ZFX	5.89	6.37	5.36
LINC00853	5.89	6.23	5.52
CUL2	5.89	6.29	5.55
OTTHUMG00000171084	5.89	6.56	5.63
RAMP3	5.89	6.58	5.25
RNA5SP69	5.89	6.54	5.41
TCEAL7	5.89	6.53	5.05
PMM2	5.89	6.42	5.31
SLC39A13	5.89	6.16	5.69
ECRP	5.89	6.34	5.29
RHOD	5.89	6.19	5.70
GTF3C4	5.89	6.30	5.31
SFMBT2	5.89	6.20	5.42
UCP2	5.89	6.80	5.26
SMARCD1	5.89	6.26	5.55
OTTHUMG00000024203	5.89	6.14	5.56
TBC1D3	5.89	6.42	5.53
BTRC	5.89	6.18	5.34
RIN1	5.89	6.19	5.59
C2orf68	5.89	6.21	5.67
TTTY15	5.89	7.24	3.31
RPA1	5.89	6.17	5.53
GOLPH3L	5.89	6.40	5.42
TOPBP1	5.89	6.51	5.35
PNPT1	5.89	6.15	5.32
C12orf44	5.89	6.07	5.67
OTTHUMG00000159124	5.89	6.12	5.36
ZFAND2B	5.89	6.18	5.72
GNRHR2	5.88	6.19	5.61
LTBP4	5.88	6.27	5.59
FOCAD	5.88	6.26	5.45
SERTAD2	5.88	6.63	4.96
ZNF160	5.88	6.28	5.40
GFM2	5.88	6.42	4.97
TOR1AIP2	5.88	6.32	5.29
OXER1	5.88	6.37	5.33
ACTR1A	5.88	6.43	5.28
RNF126	5.88	6.22	5.45
MIR26B	5.88	6.20	5.57

SPOPL	5.88	6.33	5.41
CDKL5	5.88	6.53	4.98
SWI5	5.88	6.12	5.57
ANKRA2	5.88	6.15	5.40
CYB5R1	5.88	6.23	5.58
LOC100128340	5.88	6.53	5.27
MYLK-AS1	5.88	6.18	5.68
LARP4	5.88	6.20	5.27
ADH5	5.88	6.16	5.60
MFSD5	5.88	6.25	5.12
REPS1	5.88	6.20	5.35
MIR4292	5.88	6.37	5.58
PSMD11	5.88	6.26	5.16
SLC35A5	5.88	6.82	5.28
PCAT6	5.88	6.37	5.41
ENTPD7	5.88	6.47	5.46
SRP19	5.88	6.35	5.46
MORF4L1	5.88	6.10	5.52
ARMC9	5.88	6.69	5.11
SOCS7	5.88	6.40	5.43
HSPBAP1	5.88	6.24	5.06
SERPINE2	5.88	7.38	4.61
RPS6KC1	5.88	6.29	5.35
ING4	5.88	6.39	5.40
TAF8	5.88	6.22	5.64
TRMT10B	5.88	6.28	5.50
OTTHUMG00000165033	5.88	6.38	5.36
LPCAT1	5.88	6.41	5.46
GMDS	5.88	6.47	5.43
MOB1B	5.88	6.36	5.08
TMEM206	5.88	6.14	5.57
DNTTIP1	5.88	6.22	5.63
MFSD8	5.88	6.35	5.33
SPRYD7	5.88	6.37	5.47
OTTHUMG00000168766	5.88	6.24	5.59
MLLT6	5.88	6.14	5.47
ALAS1	5.88	6.21	5.54
ST6GALNAC6	5.88	6.27	5.52
SGPL1	5.88	6.60	5.05
SPATA6	5.87	6.17	5.24
PTPRA	5.87	6.20	5.47
PITRM1-AS1	5.87	6.17	5.52
DRAM2	5.87	6.49	5.26
MPLKIP	5.87	6.18	5.20
PLBD1	5.87	6.27	5.39
EIF2AK3	5.87	6.10	5.53
PLK1S1	5.87	6.36	5.37
ACTA2	5.87	7.31	5.15
VAX2	5.87	6.15	5.67

UCHL3	5.87	6.50	5.21
RARA	5.87	6.21	5.71
KDM5A	5.87	6.18	5.22
STAG1	5.87	6.44	5.06
CLIP2	5.87	6.24	5.53
OTTHUMG00000166655	5.87	6.18	5.64
NISCH	5.87	6.28	5.48
LOC100996385	5.87	6.61	5.50
AMMECR1L	5.87	6.37	5.35
AP4B1	5.87	6.31	5.43
GRIN2D	5.87	6.47	5.16
RPS9	5.87	6.16	5.53
PTCRA	5.87	6.10	5.58
WDR35	5.87	6.30	5.20
PRKD3	5.87	6.48	4.96
PPP1R21	5.87	6.18	5.62
MGC21881	5.87	6.41	5.14
PPP1R15B	5.87	6.36	5.36
KANSL3	5.87	6.08	5.50
LOC100652869	5.87	6.12	5.63
PTOV1-AS1	5.87	6.26	5.31
TLK1	5.87	6.19	5.45
FBXO30	5.87	6.57	5.04
OIP5-AS1	5.87	6.65	5.04
RTN4	5.87	6.29	5.36
FAM219A	5.87	6.29	5.61
TAGLN2	5.87	7.04	4.54
KANSL2	5.87	6.16	5.69
HIST1H2BB	5.87	6.38	5.57
IGKV3-15	5.87	6.58	5.32
LINC00478	5.87	6.30	5.28
TOLLIP-AS1	5.87	6.22	5.58
OTTHUMG00000149070	5.87	6.40	5.50
PARP14	5.87	6.57	4.89
TRIO	5.87	6.62	5.21
RNU6ATAC	5.87	6.15	5.58
HOXD10	5.87	6.87	5.31
UBAC2	5.87	6.16	5.43
DNAJC4	5.87	6.27	5.53
FAR1-IT1	5.87	7.02	5.09
FGD1	5.87	6.10	5.71
WBP4	5.87	6.49	5.12
GALK2	5.87	6.19	5.47
BOP1	5.87	6.23	5.37
PTPN22	5.87	6.62	5.15
ZFPM1	5.87	6.22	5.69
FBXO22	5.87	6.27	5.23
BICD1	5.87	6.77	5.19
SLC22A17	5.87	6.32	5.47

IREB2	5.87	6.45	5.11
IFI27	5.87	6.61	5.28
CTDSPL	5.87	6.24	5.49
ERCC6L2	5.87	6.20	5.16
FGF7	5.87	7.22	4.67
CAB39	5.87	6.17	5.33
DLX6	5.87	6.26	5.58
SLC30A6	5.87	6.22	5.48
SLC25A13	5.86	6.35	5.39
PLCE1	5.86	6.19	5.52
KIAA1715	5.86	6.50	5.07
ITPKB	5.86	6.35	5.50
ZNF24	5.86	6.20	5.29
ONECUT2	5.86	6.04	5.62
RIMBP3	5.86	6.11	5.58
EML3	5.86	6.10	5.51
LINC00508	5.86	6.55	5.31
PSMD6	5.86	6.28	5.29
BCL7B	5.86	6.20	5.62
RICTOR	5.86	6.38	4.99
MRPL55	5.86	6.09	5.71
NAA10	5.86	6.15	5.66
C9orf9	5.86	6.06	5.48
EGFL8	5.86	6.06	5.73
PXK	5.86	6.46	4.95
KAT6A	5.86	6.26	5.54
TMEM30B	5.86	6.39	5.32
CARF	5.86	6.48	5.28
NUMBL	5.86	6.05	5.63
SLC25A12	5.86	6.17	5.38
OTUD6B	5.86	6.23	5.30
SCFD2	5.86	6.20	5.40
LOC100130093	5.86	6.26	5.30
UBAC2-IT1	5.86	6.41	5.43
CSF1	5.86	6.29	5.45
SOAT1	5.86	6.20	5.44
FAM65A	5.86	6.12	5.54
FAM126A	5.86	6.81	4.86
PTPLAD2	5.86	6.42	5.04
TMEM187	5.86	6.14	5.59
AAAS	5.86	6.20	5.26
FIS1	5.86	6.36	5.34
LDLRAD3	5.86	6.22	5.21
C11orf39	5.86	6.25	5.36
FPR1	5.86	7.04	5.04
OTTHUMG00000156753	5.86	6.25	5.58
RNA5SP383	5.86	6.32	5.31
DNAJC8	5.86	6.12	5.45
CLDN19	5.86	6.03	5.52

LOC100505573	5.86	6.24	5.39
C16orf90	5.86	6.24	5.44
ELF2	5.86	6.33	5.46
TTBK2	5.86	6.31	5.44
WRNIP1	5.86	6.36	5.29
ANKRD11	5.86	6.26	5.43
HSD17B8	5.86	6.14	5.53
DVL1	5.86	6.13	5.52
DYX1C1-CCPG1	5.86	6.32	4.55
QRFP	5.86	6.24	5.62
PAK1	5.86	6.22	5.48
TRNT1	5.86	6.09	5.59
TMEM196	5.86	6.74	4.68
LINC00482	5.86	6.34	5.31
SEPT6	5.86	6.25	5.52
OTTHUMG00000019916	5.86	6.21	5.45
CDKN2D	5.86	6.05	5.51
C21orf33	5.86	6.07	5.41
SIPA1L2	5.86	6.60	5.06
CLEC7A	5.86	7.46	5.13
MTMR14	5.86	6.19	5.58
TMEM198B	5.86	6.16	5.37
HSP90AB3P	5.86	6.38	5.23
RNF8	5.86	6.21	5.54
EGFR	5.86	6.43	5.51
HIAT1	5.85	6.20	5.19
U2AF1L4	5.85	6.23	5.42
SACS	5.85	6.74	4.78
STAU2	5.85	6.21	5.20
MIR1205	5.85	6.21	5.09
COL9A3	5.85	6.23	5.22
PRPF6	5.85	6.04	5.36
EPB41L1	5.85	6.24	5.29
ETHE1	5.85	6.38	5.31
NFATC3	5.85	6.09	5.50
PIEZO1	5.85	6.34	5.39
C4A	5.85	6.25	5.58
NFYC	5.85	6.05	5.59
HIST1H4J	5.85	6.42	5.46
YIPF4	5.85	6.33	5.26
PODXL	5.85	6.74	5.11
LTN1	5.85	6.27	5.33
SYNGR2	5.85	6.75	5.27
SLC4A1AP	5.85	6.29	5.39
ZNF830	5.85	6.20	5.25
UBE3B	5.85	6.10	5.67
C12orf49	5.85	6.21	5.22
C11orf31	5.85	6.38	5.26
FCGRT	5.85	6.12	5.67

ABCD3	5.85	6.47	5.20
OTTHUMG00000151134	5.85	6.46	5.26
NECAP2	5.85	6.23	5.19
OR1E2	5.85	6.37	5.22
HSPA6	5.85	7.17	4.87
NCOA5	5.85	6.46	5.42
PER1	5.85	6.17	5.50
CNFN	5.85	6.43	5.44
MIR4316	5.85	6.16	5.45
CDC42EP5	5.85	6.24	5.48
TACC2	5.85	6.18	5.09
LGALS9B	5.85	6.19	5.52
LLPH	5.85	6.15	5.48
TNFSF12-TNFSF13	5.85	6.10	5.55
CDK5RAP2	5.85	6.09	5.29
PREX2	5.85	6.49	4.76
SPOCK1	5.85	6.53	5.46
SHQ1	5.85	6.29	5.42
OGFOD3	5.85	6.10	5.51
NUP160	5.85	6.30	5.21
CWF19L2	5.85	6.24	5.18
CTTNBP2	5.85	7.07	4.98
MIR3622A	5.85	6.24	5.32
ZNF121	5.85	6.36	5.25
EIF4G1	5.85	6.23	5.43
MFSD7	5.85	6.08	5.42
HIST1H2BD	5.85	6.44	5.17
ZHX3	5.85	6.08	5.46
UBN2	5.85	6.12	5.43
SDC1	5.85	6.55	5.44
H2AFY	5.85	6.42	5.20
GORASP2	5.85	6.30	5.31
HERC2P4	5.85	6.33	5.25
SCFD1	5.85	6.13	5.14
NTF4	5.84	6.56	5.31
CFL2	5.84	6.29	5.42
ANKLE2	5.84	6.40	5.06
MRPS33	5.84	6.17	5.62
RNF219	5.84	6.29	5.23
PKIG	5.84	6.14	5.36
RAB32	5.84	6.52	4.94
PREP	5.84	6.46	4.86
SNORA7B	5.84	6.54	5.20
ADI1	5.84	6.04	5.56
RNFT1	5.84	6.42	5.33
LOC646862	5.84	6.35	5.05
EIF2B5	5.84	6.10	5.15
DUSP12	5.84	6.22	5.38
PSME3	5.84	6.33	5.15

SLC12A2	5.84	6.33	5.62
HRCT1	5.84	6.16	4.93
NUDT5	5.84	6.37	5.10
DDIT4	5.84	6.64	5.04
NAA15	5.84	6.38	5.24
TPST1	5.84	6.35	5.24
CLIP4	5.84	6.46	5.32
CYB5D2	5.84	6.12	5.47
ATAD2B	5.84	6.29	5.33
BMPR1A	5.84	6.71	5.05
LINC00933	5.84	6.17	5.56
ADARB1	5.84	6.25	5.27
SNAPC5	5.84	6.08	5.53
SLC9B2	5.84	6.50	5.36
NAP1L5	5.84	6.15	5.55
ATAT1	5.84	6.01	5.64
GPX7	5.84	6.46	4.81
CDKN2AIP	5.84	6.09	5.33
SLC43A1	5.84	6.11	5.41
WDR91	5.84	6.11	5.28
ADPRHL1	5.84	6.26	5.56
UQCRQ	5.84	6.08	5.59
NOP14	5.84	6.26	5.41
C6orf15	5.84	6.33	5.65
IFT122	5.84	6.14	5.29
CTAG2	5.84	6.11	5.61
TBC1D3G	5.84	6.06	5.48
FAM135A	5.84	6.18	5.50
CA5B	5.84	6.80	4.75
FXYD6	5.84	6.07	5.54
LOC100128075	5.84	6.29	5.40
MORC4	5.84	6.69	4.88
GARS	5.84	6.83	4.96
ZNF286A	5.84	6.34	4.96
BCOR	5.84	6.23	5.52
ZNF738	5.84	6.12	5.56
FYCO1	5.84	6.39	4.96
KLF16	5.84	6.36	5.37
EXOG	5.84	6.09	5.35
FAM3C	5.84	6.40	5.22
LEPREL4	5.84	6.10	5.56
SCAF8	5.84	6.17	5.22
RNF169	5.84	6.24	5.30
TMEM140	5.84	6.09	5.57
MKNK2	5.84	6.21	5.56
EXOSC1	5.84	6.39	5.21
SLCO3A1	5.84	6.15	5.33
MIR4492	5.84	6.24	5.33
VASP	5.84	6.01	5.49

PPP1R1A	5.84	6.54	4.98
TNPO3	5.84	6.18	5.43
HMGN4	5.83	6.32	4.64
RNF5	5.83	6.05	5.66
SYAP1	5.83	6.66	4.93
KIAA1143	5.83	6.21	5.41
ANGPT2	5.83	6.95	5.07
CARM1	5.83	6.29	5.42
EVA1B	5.83	6.54	5.30
ATP1A1OS	5.83	6.08	5.31
OTTHUMG00000150771	5.83	6.23	5.53
CENPT	5.83	6.15	5.62
RNF14	5.83	6.14	5.21
SNORD10	5.83	6.29	5.56
UBXN1	5.83	6.22	5.42
USMG5	5.83	6.13	5.42
PRDX4	5.83	6.50	4.94
C6orf47-AS1	5.83	6.30	5.13
OTTHUMG00000057531	5.83	6.30	5.13
ZSCAN26	5.83	6.22	5.49
DDX11	5.83	6.14	5.50
HPS4	5.83	6.03	5.50
CRYBG3	5.83	6.37	5.45
NDUFS1	5.83	6.39	5.05
TMEM91	5.83	6.18	5.39
EML4	5.83	6.19	5.19
SIVA1	5.83	6.13	5.34
CRKL	5.83	6.22	5.18
NEU2	5.83	6.36	5.35
NUDT3	5.83	5.97	5.47
NOL10	5.83	6.24	5.39
FLT3LG	5.83	6.27	5.45
NEK8	5.83	6.12	5.45
PLRG1	5.83	6.46	5.22
CUL5	5.83	6.31	5.31
LPCAT2	5.83	6.51	5.34
TTC21B	5.83	6.16	5.21
TEX2	5.83	6.21	5.46
SVIL	5.83	6.08	5.23
SRFBP1	5.83	6.46	5.00
LAMA2	5.83	6.77	4.72
ATP6V1D	5.83	6.50	5.28
PAQR6	5.83	6.35	5.55
LOC401589	5.83	6.21	5.34
ARHGAP5	5.83	6.34	5.33
TMEM19	5.83	6.23	5.32
FLNB	5.83	6.32	5.53
CDKL1	5.83	6.03	5.57
MLXIP	5.83	6.37	5.35

FBXO8	5.83	6.48	4.93
PRCD	5.83	6.30	5.58
CA11	5.83	6.21	5.40
CST4	5.83	6.21	5.45
LOC150776	5.83	6.49	5.30
SEPSECS	5.83	6.48	5.29
PRSS22	5.83	6.17	5.54
FH	5.83	6.39	5.11
IFRD1	5.83	6.10	5.33
TNFAIP2	5.83	6.13	5.35
TM2D3	5.83	6.00	5.44
PLAA	5.83	6.58	5.13
DAXX	5.83	6.21	5.35
FEM1C	5.83	6.48	5.20
CCS	5.83	6.06	5.49
AREL1	5.83	6.36	5.38
FAM90A10P	5.83	6.28	5.45
TRBV23OR9-2	5.83	6.14	5.50
OTTHUMG00000013926	5.83	6.30	5.42
COA1	5.82	6.12	5.41
SNORD56B	5.82	6.40	5.18
RNF4	5.82	6.17	5.14
DECR2	5.82	6.07	5.63
ARFIP1	5.82	6.16	5.21
THG1L	5.82	6.22	5.35
RNH1	5.82	6.14	5.49
SIX3	5.82	6.10	5.50
PLBD2	5.82	6.31	5.42
ZGPAT	5.82	6.44	5.46
CBR1	5.82	6.05	5.52
OGFRL1	5.82	6.11	5.25
HERC2	5.82	5.97	5.64
PMEPA1	5.82	6.44	5.30
NUP43	5.82	6.72	5.01
ZNF512	5.82	6.17	5.51
ATF7IP2	5.82	6.15	5.44
ZNF808	5.82	6.27	5.26
PDCD4-AS1	5.82	6.21	5.37
IFITM4P	5.82	6.28	5.40
PPP1R35	5.82	6.01	5.47
HLA-DPB2	5.82	6.81	5.30
CDK8	5.82	6.67	5.06
RPL14	5.82	6.00	5.68
PPIAP30	5.82	6.26	5.54
PCGF3	5.82	6.16	5.33
C11orf48	5.82	6.27	5.38
ADH1C	5.82	6.56	5.13
LBR	5.82	6.12	5.52
ANKRD37	5.82	6.47	5.20

FGD5	5.82	6.16	5.37
TUBGCP2	5.82	6.25	4.84
LRRC57	5.82	6.06	5.31
LINC00341	5.82	6.27	5.52
ZNF548	5.82	6.11	5.44
TRRAP	5.82	5.99	5.56
GLTP	5.82	6.24	5.35
H1FOO	5.82	6.04	5.68
RNF2	5.82	6.17	5.23
FAM180A	5.82	7.08	4.61
ARHGAP31	5.82	6.29	5.55
B4GALT6	5.82	6.51	5.12
SPG7	5.82	6.12	5.25
PLEKHA6	5.82	6.49	5.54
AP1M1	5.82	6.16	5.44
C21orf59	5.82	6.29	5.19
EXOSC4	5.82	6.39	5.45
XRCC3	5.82	6.14	5.45
CLCN6	5.82	6.25	4.98
SNORA80B	5.82	6.45	5.37
VDAC2	5.82	6.14	4.96
MOSPD3	5.82	6.17	5.26
PDE8A	5.82	6.12	5.14
PPIA	5.82	6.34	5.27
FAM106A	5.81	6.34	5.40
FAM132A	5.81	6.35	5.47
MESDC1	5.81	6.00	5.47
RBM28	5.81	6.20	5.37
LRRC47	5.81	6.42	5.39
LOC100506999	5.81	6.19	5.58
TCF19	5.81	6.25	5.42
DDX11L9	5.81	6.38	5.28
ODF2	5.81	6.14	5.37
GNB2	5.81	6.18	5.33
GLUD1P2	5.81	5.97	5.40
KIAA1432	5.81	6.22	5.31
CNOT11	5.81	6.07	5.57
SLC35A2	5.81	6.29	5.53
ZC3H8	5.81	6.38	5.22
OTTHUMG00000149585	5.81	7.31	4.48
SLC25A46	5.81	6.26	5.29
CYP51A1	5.81	6.10	5.17
GTPBP10	5.81	6.09	5.47
JAGN1	5.81	6.23	5.44
OTTHUMG00000179792	5.81	6.28	5.24
DCAF12	5.81	6.05	5.32
RPL38	5.81	6.02	5.66
LTA	5.81	6.14	5.44
CASP16	5.81	6.10	5.42

DNAJB9	5.81	6.15	5.30
BCL9L	5.81	6.21	5.40
TXNDC12	5.81	6.16	5.44
OTTHUMG00000074727	5.81	6.31	4.99
NEURL	5.81	6.25	5.59
LIMS2	5.81	6.39	5.16
CTSL1P2	5.81	6.08	5.45
HLA-DQB2	5.81	6.25	5.44
LOC100288911	5.81	6.69	4.50
FTSJ3	5.81	5.93	5.62
FAM214A	5.81	6.18	5.22
TRIM27	5.81	6.19	5.30
TTYH3	5.81	6.24	5.56
PGAM2	5.81	6.73	5.19
MIR320D1	5.81	6.72	5.23
ANP32B	5.81	6.09	5.21
FUCA1	5.81	7.25	4.91
GUCA2A	5.81	6.30	5.65
TSPYL4	5.81	6.13	5.47
DYNLL2	5.81	5.98	5.46
DCHS1	5.81	6.46	5.14
DDB1	5.81	6.16	5.45
CCDC127	5.81	6.19	5.41
NFATC2IP	5.81	6.09	5.23
B4GALT3	5.81	6.02	5.44
ZNF12	5.81	6.49	5.31
ROBO4	5.81	6.04	5.27
MDH2	5.81	6.13	5.30
GNG7	5.81	6.04	5.44
UBE3C	5.81	6.44	5.21
RHOU	5.81	6.18	5.35
MNAT1	5.81	6.44	5.05
METTL21B	5.81	6.07	5.64
NUCB1	5.81	6.17	5.24
TMED5	5.81	6.34	5.28
KIF1C	5.81	6.15	5.43
TATDN1	5.81	6.12	5.05
RARG	5.81	6.09	5.33
UGCG	5.81	6.35	5.04
RHOQ	5.81	6.28	5.44
CHURC1-FNTB	5.81	5.99	5.37
CELF2	5.81	6.34	5.16
ACACA	5.80	6.12	5.10
RIOK1	5.80	6.26	5.14
SLC25A17	5.80	6.24	5.37
LINC00674	5.80	6.53	5.39
CREB1	5.80	6.16	4.95
AP1G2	5.80	6.23	5.44
PARP2	5.80	6.07	5.05

LOC100499489	5.80	6.19	5.44
A2M-AS1	5.80	6.33	5.37
MORC2	5.80	6.03	5.37
RHOT2	5.80	6.15	5.32
SIRT2	5.80	6.09	5.43
LAS1L	5.80	6.21	5.33
SPATS2	5.80	6.23	5.23
GPR183	5.80	7.04	5.12
ASAP3	5.80	6.03	5.43
AUTS2	5.80	6.47	5.46
DNAJB12	5.80	6.14	5.15
CDS2	5.80	6.23	5.50
ARID2	5.80	6.22	5.26
TESK1	5.80	6.11	5.52
ZNF98	5.80	6.09	5.41
SMC6	5.80	6.16	5.26
OTUD4	5.80	6.20	5.50
AMT	5.80	6.06	5.51
NPAT	5.80	6.25	4.94
OTTHUMG00000140107	5.80	6.82	4.58
MRPS26	5.80	6.11	5.62
WDR48	5.80	6.18	5.11
ATG9A	5.80	6.15	5.43
TNFRSF14	5.80	6.24	5.14
MESDC2	5.80	6.14	5.37
SNRNP48	5.80	6.14	5.15
CRCP	5.80	6.05	5.54
ASXL2	5.80	6.14	5.23
OTTHUMG00000002915	5.80	6.28	5.48
OSTM1	5.80	6.13	5.16
GNPTG	5.80	6.10	5.58
C18orf8	5.80	6.47	5.15
RASA3	5.80	6.55	5.10
EIF3H	5.80	5.95	5.50
ATPAF1	5.80	6.06	5.50
C15orf40	5.80	5.92	5.63
STXBP5	5.80	6.63	5.09
TTLL7	5.80	6.34	5.35
PCK2	5.80	6.14	5.45
RPAP3	5.80	6.38	5.21
BIRC2	5.80	6.28	5.15
LAT	5.80	6.13	5.38
STAB1	5.80	6.13	5.51
ADRM1	5.80	6.01	5.26
MCOLN1	5.80	6.13	5.48
LTA4H	5.80	6.37	5.39
LOC554174	5.80	6.00	5.56
RILPL2	5.80	6.05	5.46
DEDD2	5.80	6.07	5.50

CERK	5.80	6.07	5.55
CBL	5.80	6.34	5.14
TMEM54	5.80	6.19	5.41
EPT1	5.79	6.34	5.26
HLA-DQB1-AS1	5.79	6.81	4.69
RNA5SP279	5.79	6.18	5.31
MRPL19	5.79	6.27	5.29
ZNF562	5.79	6.34	5.24
SLC35F3	5.79	5.96	5.52
C6orf106	5.79	6.29	5.29
MRPL32	5.79	6.12	5.35
C16orf11	5.79	6.19	5.33
WISP1	5.79	6.96	4.75
FMNL3	5.79	6.32	4.98
CEP135	5.79	6.10	5.22
RNF6	5.79	6.46	5.09
TSHZ1	5.79	6.08	5.48
NSMAF	5.79	6.09	5.32
IGLV2-8	5.79	6.28	5.30
CADM3	5.79	6.69	5.22
EMC8	5.79	5.95	5.57
UST	5.79	6.86	5.24
CAPZB	5.79	6.83	4.62
SNX18	5.79	6.29	5.04
NOL9	5.79	6.21	5.39
ABI3	5.79	6.27	5.54
TMEM86A	5.79	6.14	5.35
RRM1	5.79	6.31	5.31
SNORA52	5.79	5.95	5.54
LDLRAP1	5.79	5.94	5.52
PLEKHB2	5.79	6.38	5.39
DYNLRB1	5.79	6.07	5.61
POLR2J	5.79	6.48	5.23
SFTPD	5.79	6.35	5.07
LOC100507474	5.79	6.23	5.33
PHB	5.79	6.11	5.41
RRAS2	5.79	6.19	5.34
SEMA6D	5.79	6.54	5.41
RHBDD2	5.79	6.13	5.59
RAD52	5.79	6.14	5.51
FRMD8P1	5.79	6.21	5.24
TP53I3	5.79	6.54	5.24
GAA	5.79	6.18	5.45
KATNB1	5.79	6.08	5.59
PPCS	5.79	6.11	5.17
PART1	5.79	6.15	5.55
TAMM41	5.79	6.10	5.56
ZNF354A	5.79	6.29	5.21
ZNF506	5.79	6.21	5.13

ARHGEF11	5.79	6.16	5.35
LSMEM2	5.79	6.46	5.33
ARHGAP10	5.79	6.04	5.04
CHRFAM7A	5.79	6.07	5.25
MPEG1	5.79	6.53	5.02
TUBA4B	5.79	6.27	5.37
SPAG16	5.79	6.02	5.46
LOC643072	5.79	6.24	5.28
FAM173A	5.79	6.47	4.95
OTTHUMG00000169178	5.79	6.42	5.13
RAB35	5.79	6.11	5.23
KIAA1549	5.79	6.60	4.95
KIFC1	5.79	6.01	5.37
LRP11	5.79	6.05	5.26
MRPL43	5.78	6.18	5.41
COPRS	5.78	6.26	5.00
PIWIL4	5.78	6.50	5.02
SLC23A1	5.78	6.20	5.30
METRNL	5.78	6.87	4.80
VPS4A	5.78	6.43	4.91
ABCB10	5.78	6.09	5.23
ACYP2	5.78	6.18	5.30
TREX2	5.78	6.29	4.87
APEH	5.78	6.13	5.39
FANCL	5.78	6.60	5.02
WASIR2	5.78	6.21	5.45
ZDHHC5	5.78	6.25	5.30
FOSL2	5.78	6.32	5.23
TMEM106C	5.78	6.00	5.58
SLC25A53	5.78	6.09	5.50
PI4K2B	5.78	6.47	5.18
CLNS1A	5.78	6.04	5.56
SMKR1	5.78	6.31	5.38
VIMP	5.78	6.30	5.33
CSRP1	5.78	6.05	5.45
SULT1C2	5.78	6.60	5.42
CENPB	5.78	5.99	5.47
KDM6A	5.78	6.41	5.03
AGT	5.78	6.47	5.25
CAMK2N1	5.78	7.10	5.09
FOXJ3	5.78	6.06	5.55
C1QTNF6	5.78	6.36	5.30
C6orf203	5.78	6.16	5.21
OTTHUMG00000041230	5.78	6.22	5.36
LOC400464	5.78	6.11	5.41
TXLNG	5.78	6.20	5.19
FBXO4	5.78	6.23	5.19
BCL2	5.78	6.51	4.83
CAPZA2	5.78	6.33	5.00

VWCE	5.78	6.17	5.42
TMC4	5.78	6.15	5.37
AGO1	5.78	5.99	5.52
ELAC2	5.78	6.11	5.38
ULK2	5.78	5.98	5.33
TTC7A	5.78	6.12	5.14
ELP5	5.78	6.09	5.55
ZDHHC7	5.78	6.36	4.98
STK19	5.78	6.10	5.54
HIST1H2BM	5.78	7.48	4.73
OTTHUMG00000160454	5.78	6.40	4.90
DNMT1	5.78	6.10	5.29
MARCO	5.78	7.23	4.73
CLDND2	5.78	6.26	5.44
TSC2	5.78	5.93	5.52
RNU7-25P	5.78	6.32	5.40
C1orf148	5.78	6.11	5.52
SLC37A2	5.78	6.53	4.93
ZBTB21	5.78	6.69	5.07
SETBP1	5.78	6.10	5.32
TRAF3	5.78	6.10	5.37
ZNF407	5.78	6.02	5.26
VIPAS39	5.78	6.10	5.21
GPC1	5.78	6.06	5.60
PTAFR	5.78	6.52	5.03
CLEC2D	5.78	6.20	5.46
KLF12	5.78	6.46	4.69
SOWAHA	5.78	5.99	5.43
AMN1	5.78	6.02	5.62
CEP89	5.78	6.29	5.33
TCEAL5	5.78	6.37	5.30
GPR116	5.77	7.15	4.14
SLN	5.77	6.93	4.90
C6orf136	5.77	6.03	5.45
LINC00961	5.77	6.37	5.13
NHP2L1	5.77	6.08	5.60
ABCA1	5.77	6.36	5.15
YIPF1	5.77	6.22	5.32
SLC27A3	5.77	6.10	5.27
NUP155	5.77	6.21	5.11
TPRG1L	5.77	6.21	5.08
KIR2DL3	5.77	6.25	5.22
ESYT1	5.77	6.04	5.31
TP53	5.77	6.10	5.37
FAM50A	5.77	6.14	5.36
WDR26	5.77	6.20	4.95
BMP1	5.77	6.48	5.18
LOC730102	5.77	6.33	5.48
SLC38A3	5.77	6.22	5.16

ZMYND8	5.77	5.94	5.48
LOC728392	5.77	6.67	4.97
ZDHHC21	5.77	6.43	4.95
AHRR	5.77	6.05	5.59
EIF1AD	5.77	6.01	5.45
MTRF1	5.77	6.19	5.26
NUP93	5.77	6.18	4.92
RBAK	5.77	6.11	5.51
ASGR2	5.77	6.29	5.43
MIR1231	5.77	6.19	5.39
NR1D2	5.77	6.37	4.42
APBA1	5.77	6.02	5.54
SMC2	5.77	6.47	5.18
TMEM18	5.77	5.97	5.53
IFFO1	5.77	6.25	5.40
SCAP	5.77	6.25	5.52
KRTAP5-6	5.77	6.42	5.48
KCNF1	5.77	6.06	5.30
SLC7A6	5.77	6.00	5.22
KCTD11	5.77	6.09	5.48
GOLGA8EP	5.77	6.18	4.80
KDM4A	5.77	6.02	5.33
DMWD	5.77	6.17	5.31
LOC285300	5.77	6.53	5.07
PLAGL2	5.77	6.02	5.53
ATXN10	5.77	6.35	4.99
CSDC2	5.77	6.16	5.54
EIF4E2	5.77	6.39	5.29
FAM86A	5.77	6.22	5.42
TMED3	5.77	6.13	5.47
LY75-CD302	5.77	6.62	5.10
NPAS2	5.77	6.57	4.89
COQ9	5.77	5.97	5.49
ANGPTL6	5.77	5.98	5.54
PCSK7	5.77	5.93	5.57
NRIP2	5.77	5.92	5.67
SLC35B1	5.77	6.06	5.40
LARP4B	5.77	6.08	5.45
JSRP1	5.77	6.02	5.61
EBNA1BP2	5.77	6.01	5.30
INIP	5.77	6.25	5.15
NUTM2A	5.77	5.94	5.41
MIR502	5.77	6.32	4.95
TBXA2R	5.77	6.09	5.31
MRPS9	5.77	6.11	5.09
ANK2	5.77	6.38	5.01
GNAZ	5.77	6.01	5.54
SPIN4	5.77	6.05	5.26
OR2T34	5.77	6.81	5.04

EFHC1	5.77	6.02	5.17
MRPL27	5.77	6.10	5.54
SPATA18	5.77	6.33	5.17
PPP1R12B	5.76	6.04	5.38
NADK2	5.76	6.16	5.34
ANKRD27	5.76	6.13	5.23
FTSJ1	5.76	6.11	5.52
FAM115C	5.76	6.58	5.18
OTTHUMG00000172608	5.76	6.18	5.23
CARKD	5.76	5.89	5.59
UBXN7	5.76	6.23	5.01
SLIT2	5.76	6.66	4.89
SNORD123	5.76	6.53	4.76
ARHGEF9	5.76	6.09	5.12
ZNF259	5.76	5.98	5.40
FXYD1	5.76	6.27	5.01
SLC6A10P	5.76	6.18	5.23
INO80E	5.76	5.98	5.56
MRPL46	5.76	6.11	5.21
CTR9	5.76	6.25	5.30
ALKBH3	5.76	6.18	5.18
EMD	5.76	6.38	5.25
MGC27345	5.76	6.49	4.94
H2AFZ	5.76	6.37	5.27
TMX4	5.76	6.34	5.07
RASAL3	5.76	6.03	5.44
TMCO6	5.76	5.97	5.51
IPO8	5.76	6.29	5.11
B3GALNT2	5.76	5.96	5.28
HSPA12B	5.76	6.11	5.39
EIF2AK4	5.76	6.18	5.44
DECR1	5.76	6.01	5.48
ME2	5.76	6.72	5.11
RANGRF	5.76	6.28	5.27
DUSP18	5.76	6.59	4.90
GSN	5.76	6.24	5.23
HSPBP1	5.76	6.10	5.42
TP53INP1	5.76	6.27	5.02
DPP8	5.76	6.15	5.30
KCTD7	5.76	6.24	5.49
PEX13	5.76	6.13	5.38
NAA16	5.76	6.08	5.50
GSTA5	5.76	6.42	5.30
PLSCR1	5.76	6.23	5.31
LOC100303749	5.76	6.22	5.11
MAP3K1	5.76	6.34	5.30
TM2D1	5.76	5.99	5.41
TBC1D1	5.76	6.11	5.38
PMF1-BGLAP	5.76	6.56	5.39

TTC27	5.76	6.08	5.39
WDR45B	5.76	6.20	5.30
TMEM9B	5.76	6.05	5.29
IFI35	5.76	6.16	5.35
LINC00163	5.76	6.03	5.31
GLIS3-AS1	5.76	6.63	5.17
TAZ	5.76	6.07	5.52
MIR338	5.76	5.90	5.52
KIF16B	5.76	6.03	5.17
KLF8	5.76	6.43	4.67
OTTHUMG00000176522	5.76	6.17	5.28
TBX18	5.76	6.12	5.39
RNF170	5.76	6.20	5.00
SMG7	5.76	6.17	5.05
PRSS57	5.76	5.98	5.41
HCG27	5.76	6.17	5.45
CDIPT	5.76	5.98	5.54
WDR73	5.76	6.08	5.32
ACVR1	5.76	6.40	4.91
C6orf89	5.76	6.31	4.92
OTTHUMG00000172397	5.76	6.30	4.93
OTTHUMG00000013241	5.76	6.15	5.11
SNORD35B	5.76	6.26	4.79
ZNF579	5.76	6.11	5.39
PAXBP1	5.76	6.38	5.20
NFATC4	5.76	6.15	5.24
SERINC2	5.76	5.98	5.43
CIC	5.76	5.97	5.60
MCM3AP	5.76	6.05	5.30
LOC401010	5.76	6.33	5.38
UTP11L	5.76	6.40	5.11
LOC100506282	5.75	6.39	4.92
PRELID1	5.75	6.21	5.39
KIRREL	5.75	6.29	5.18
KMT2D	5.75	6.05	5.33
IDH1	5.75	5.97	5.55
HDAC3	5.75	6.21	5.00
KCTD10	5.75	6.25	5.01
ZFP3	5.75	6.15	5.26
SPG20	5.75	6.20	5.19
LINC00315	5.75	6.32	4.94
PON2	5.75	6.06	5.46
LOC100129033	5.75	6.32	5.26
SLC12A6	5.75	6.03	5.16
UBA7	5.75	6.36	5.38
SERTAD4-AS1	5.75	6.03	5.48
PARP9	5.75	6.22	5.15
BBIP1	5.75	6.28	5.39
PPP1R8	5.75	6.03	5.37

KIAA0020	5.75	6.43	5.17
CELF6	5.75	6.10	5.44
ADAM33	5.75	6.02	5.41
STK36	5.75	5.95	5.51
ZDHHC4	5.75	6.13	5.46
SLC7A6OS	5.75	6.07	5.37
ALG1L2	5.75	6.42	5.18
SIDT2	5.75	6.13	5.31
ITPR1	5.75	6.71	5.27
PFDN1	5.75	6.05	5.10
UHRF2	5.75	6.36	5.08
RBX1	5.75	6.22	5.00
OTTHUMG00000151492	5.75	6.21	5.41
ZNF330	5.75	6.13	5.16
EXOSC8	5.75	6.05	5.19
ANKRD20A12P	5.75	6.78	5.15
H1FX	5.75	6.07	5.06
NOL11	5.75	6.16	5.18
TP53INP2	5.75	5.95	5.58
RAI1	5.75	6.09	5.34
PRICKLE2	5.75	6.36	5.26
IPMK	5.75	6.20	5.28
CCNB1IP1	5.75	6.21	5.48
C11orf24	5.75	6.44	4.93
VPS54	5.75	6.40	5.09
SGCE	5.75	6.29	4.91
FAM86DP	5.75	6.32	5.17
MGAT4B	5.75	5.96	5.51
ZNF236	5.75	6.13	5.29
SLC19A3	5.75	6.01	5.36
FAM46C	5.75	6.42	4.51
TTC9C	5.75	6.02	5.24
LOC100131825	5.75	6.26	5.17
LCE3A	5.75	6.10	5.34
ALDH1L1-AS1	5.75	6.05	5.52
MSH3	5.75	5.90	5.43
SMARCE1	5.75	6.07	5.08
FUT8	5.75	6.46	5.21
B9D1	5.75	6.01	5.46
C5orf22	5.75	6.12	5.22
TRAF3IP2	5.74	6.27	5.18
SIGMAR1	5.74	6.01	5.29
THAP5	5.74	6.02	5.34
NUDT8	5.74	5.99	5.53
AFG3L2	5.74	6.12	5.22
ELK1	5.74	6.42	5.26
TRIM56	5.74	6.03	5.31
KDELR3	5.74	6.49	4.26
OTTHUMG00000171130	5.74	6.07	5.25

KLHL3	5.74	6.16	5.01
FITM2	5.74	6.21	5.13
OTTHUMG00000163061	5.74	6.22	5.40
KDM4C	5.74	6.16	5.46
ZNF260	5.74	6.38	4.80
MIR1911	5.74	6.45	5.18
TMEM8A	5.74	5.93	5.47
LAD1	5.74	6.09	5.32
OTTHUMG00000164320	5.74	6.73	5.08
NGRN	5.74	6.19	5.36
MICAL1	5.74	6.05	5.39
PTGFR	5.74	6.35	5.26
ABCC4	5.74	5.97	5.38
DEAF1	5.74	6.01	5.42
EIF2S1	5.74	6.53	5.06
NMNAT1	5.74	6.00	5.16
DPAGT1	5.74	6.20	5.19
OTTHUMG00000164136	5.74	6.12	5.18
LOC100130015	5.74	6.05	5.37
NDC1	5.74	5.99	5.32
FAM229A	5.74	6.06	5.39
YPEL2	5.74	6.24	4.85
ACOT13	5.74	6.25	5.03
FZR1	5.74	6.19	5.41
POLR3C	5.74	6.18	5.47
JAM2	5.74	6.53	5.12
CCDC107	5.74	6.35	5.32
CTSB	5.74	6.61	4.85
BASP1P1	5.74	6.45	5.27
C1orf50	5.74	6.01	5.56
PNMA1	5.74	6.12	4.98
FKBP1AP1	5.74	6.14	5.03
PTP4A2	5.74	6.13	5.30
CD2AP	5.74	5.94	5.36
FAM138E	5.74	6.10	5.26
HSD17B10	5.74	5.99	5.08
LGR4	5.74	6.30	4.99
SHARPIN	5.74	6.03	5.48
AGPS	5.74	6.71	4.22
CCDC53	5.74	6.38	5.37
SAP30	5.74	6.39	5.01
TRIM8	5.74	6.01	5.07
CD320	5.74	6.01	5.47
KCNC3	5.74	6.16	5.49
CXorf51A	5.74	6.18	5.15
PHLDA1	5.74	6.24	5.37
OCIAD2	5.74	6.49	5.33
OTTHUMG00000162762	5.74	6.55	5.27
CST2	5.74	6.11	5.23

ASPSCR1	5.74	5.93	5.51
ZCCHC9	5.74	6.12	5.47
LCMT1	5.74	6.28	5.01
USPL1	5.74	6.12	5.32
FOSL1	5.74	6.55	5.16
DDR1	5.74	5.98	5.39
CX3CL1	5.73	6.16	5.38
DMRTC2	5.73	6.30	5.35
RPL23AP53	5.73	6.76	4.89
ALG14	5.73	6.24	4.83
GRHPR	5.73	6.01	5.24
GSS	5.73	6.08	5.48
TCTN3	5.73	6.41	5.27
RBM10	5.73	5.96	5.46
POLG	5.73	5.98	5.29
PCOLCE-AS1	5.73	6.24	5.43
KIAA1429	5.73	6.20	5.28
CNTFR-AS1	5.73	6.37	5.13
TIMM17A	5.73	6.16	5.23
ARHGAP24	5.73	6.05	5.46
MRPS23	5.73	6.42	5.18
SBF2-AS1	5.73	6.11	5.31
USP10	5.73	6.11	5.00
MDN1	5.73	5.93	5.29
ADCY4	5.73	6.27	5.01
CLIC2	5.73	6.69	4.96
DCTN2	5.73	6.38	5.22
DDIT4L	5.73	6.63	5.15
MTRF1L	5.73	6.19	5.23
TBC1D19	5.73	6.11	5.16
FAM199X	5.73	6.03	5.44
SCRN2	5.73	6.12	5.34
CASP3	5.73	6.63	5.03
MTRNR2L3	5.73	6.24	5.30
MYO19	5.73	6.05	5.41
SART1	5.73	5.86	5.55
TDP2	5.73	6.18	5.06
KIAA0947	5.73	6.49	4.83
NUP205	5.73	5.98	5.15
HOXA-AS4	5.73	5.92	5.52
ARMC1	5.73	6.17	5.06
MBD5	5.73	6.16	5.37
RBMX2	5.73	6.04	5.05
PAFAH2	5.73	6.05	5.38
NEDD4	5.73	6.06	5.03
AVPI1	5.73	6.08	5.37
FLJ44635	5.73	6.04	5.23
GOLGA6L2	5.73	6.37	5.13
RABL2A	5.73	6.19	5.05

PLCB3	5.73	6.16	5.33
LSP1	5.73	6.08	5.33
BMP6	5.73	6.37	4.86
MIR24-2	5.73	6.37	5.19
DHX33	5.73	6.07	5.06
BBS1	5.73	6.05	5.58
ZFYVE1	5.73	6.15	5.51
OTTHUMG00000018028	5.73	6.10	4.79
LOC646670	5.73	5.96	5.54
TAPT1-AS1	5.73	5.92	5.53
ST7	5.73	6.01	5.29
PNMA5	5.73	6.14	5.25
OTTHUMG00000171410	5.73	6.27	5.32
CLIP3	5.73	6.17	5.17
TMEM168	5.73	6.08	5.25
DBR1	5.73	6.23	5.30
OTTHUMG00000168751	5.73	6.35	4.55
NID1	5.73	6.42	5.09
HSCB	5.73	6.31	5.19
YIF1A	5.72	6.12	5.16
ARHGAP42	5.72	6.25	5.20
NRBP2	5.72	6.14	5.26
C2orf48	5.72	6.16	5.20
BNC2	5.72	7.09	4.71
KIAA1191	5.72	6.28	4.72
EIF4ENIF1	5.72	5.95	4.95
SNORA12	5.72	7.90	4.36
TSSK3	5.72	6.20	5.17
ARHGAP18	5.72	6.50	4.86
TRABD	5.72	6.14	5.54
FLCN	5.72	5.92	5.46
AHDC1	5.72	6.02	5.54
LCMT2	5.72	6.03	5.38
MIR375	5.72	5.91	5.44
CCNDBP1	5.72	6.04	4.93
OR4C12	5.72	6.62	5.14
ZAK	5.72	6.07	5.29
FHL1	5.72	5.99	5.10
USP41	5.72	6.76	5.03
NBR2	5.72	5.99	5.04
DLAT	5.72	6.06	5.07
RBM7	5.72	6.27	5.14
FOXD1	5.72	6.29	5.13
TARSL2	5.72	6.13	5.21
SNORD115-11	5.72	6.80	4.53
SNORD115-12	5.72	6.80	4.53
PDAP1	5.72	6.09	5.08
CA12	5.72	6.67	5.23
MET	5.72	6.26	5.09

RRAGB	5.72	6.11	5.31
NOTCH4	5.72	6.15	5.25
OTTHUMG00000165331	5.72	6.50	5.14
MRPL51	5.72	6.23	5.11
APAF1	5.72	6.21	5.26
JMJD8	5.72	6.02	5.25
IDH3B	5.72	6.33	5.03
PLIN2	5.72	6.47	5.06
SLC7A8	5.72	6.35	5.11
ILKAP	5.72	6.21	4.91
CLK2	5.72	6.11	5.47
DUSP14	5.72	6.13	5.37
IGHG4	5.72	7.15	5.33
VAT1	5.72	6.27	4.96
LY6E	5.72	6.02	5.15
PSMB10	5.72	6.16	5.42
CDK19	5.72	6.27	5.10
DPM3	5.72	6.25	5.16
CBY1	5.72	6.01	5.51
CBLN3	5.72	6.27	5.46
RAB11FIP3	5.72	6.03	5.37
OTTHUMG00000155475	5.72	6.10	5.40
RBM42	5.72	6.00	5.26
KLHL18	5.72	5.87	5.51
ALDH2	5.72	6.30	5.19
NEK6	5.72	6.01	5.38
PRKAA1	5.72	5.99	5.26
KIAA1755	5.72	6.07	5.50
PRPS2	5.72	6.06	5.06
FAM185A	5.72	6.01	5.42
FNDC4	5.72	6.21	5.19
SMAD1	5.72	6.04	5.46
UBE2A	5.72	6.42	4.99
PHF11	5.72	6.11	5.34
LOC100192426	5.72	6.17	5.40
RNF19A	5.72	6.17	5.18
PHKA2-AS1	5.72	6.08	5.20
FAS	5.71	6.12	5.15
OR4C46	5.71	6.44	5.19
PP7080	5.71	5.97	5.19
ELMO2	5.71	6.13	5.32
ZMYM1	5.71	5.98	5.34
TXNL4B	5.71	6.09	5.19
MIR1226	5.71	6.50	4.59
TBCD	5.71	5.91	5.39
CCL5	5.71	6.23	5.34
C2CD3	5.71	5.93	5.35
TMSB4X	5.71	6.11	4.98
EHMT1	5.71	5.90	5.39

RBSG2	5.71	6.29	5.45
PRPS1	5.71	6.38	5.08
ZNF117	5.71	6.25	4.75
LOC100507367	5.71	6.08	5.35
PEX19	5.71	5.97	5.30
CRB2	5.71	6.41	5.29
SQSTM1	5.71	6.04	5.31
ARL5A	5.71	6.01	5.31
ZNF783	5.71	6.33	5.36
OTTHUMG00000155433	5.71	6.50	5.08
LOC643669	5.71	6.09	5.18
ELOVL4	5.71	6.34	4.83
MIR4468	5.71	6.16	5.17
PDIA3P	5.71	6.12	5.28
ACOT8	5.71	6.04	5.14
RNA5SP55	5.71	6.15	5.22
C3orf70	5.71	6.49	4.74
SLC35G3	5.71	6.32	5.11
MED24	5.71	5.85	5.39
SNORA11B	5.71	6.19	5.24
GNPNAT1	5.71	6.75	4.63
MAN2B1	5.71	6.18	5.19
ABL2	5.71	6.39	4.99
TRAF3IP2-AS1	5.71	6.11	5.18
KCNQ5-AS2	5.71	6.78	4.89
IGSF6	5.71	6.72	5.23
SORBS1	5.71	6.54	5.15
EVPL	5.71	6.18	5.47
TAF4	5.71	5.84	5.55
CSGALNACT1	5.71	6.03	5.14
ALG9	5.71	6.28	5.15
ADAMTSL2	5.71	5.99	5.37
GTF2E2	5.71	6.28	5.26
NEK9	5.71	6.14	5.17
RAET1G	5.71	6.36	5.32
DBNDD2	5.71	5.91	5.53
CPQ	5.71	6.21	5.21
VPS37B	5.71	6.11	5.32
GSC	5.71	6.46	4.89
IRF3	5.71	5.99	5.40
MAP1S	5.71	6.28	5.34
PINK1	5.71	6.22	5.16
SNORD51	5.71	6.46	5.21
LOC100506518	5.71	6.43	5.43
OTTHUMG00000032803	5.71	6.19	5.15
CCDC43	5.71	6.25	5.31
TMEM69	5.71	6.19	5.17
SLC25A4	5.71	6.15	5.09
GAREML	5.71	6.24	5.37

ZACN	5.71	5.98	5.53
CCDC71L	5.71	6.11	5.17
ZC3H12A	5.71	5.96	5.55
SNORD7	5.71	6.35	4.92
SLX4IP	5.71	5.89	5.44
MVP	5.71	6.30	5.03
OAS1	5.71	6.52	5.01
STS	5.71	6.24	5.21
ADSSL1	5.71	6.42	5.19
RNA5-8SP6	5.71	6.41	5.17
MIR100HG	5.71	6.14	5.16
POU6F1	5.71	6.09	5.44
PPIL2	5.71	5.93	5.47
CDH5	5.71	6.65	4.41
MYLPF	5.71	5.84	5.40
CCDC167	5.70	6.29	4.99
SPRTN	5.70	6.10	5.22
SLC24A6	5.70	6.17	5.12
COX11	5.70	5.95	5.43
FAM150B	5.70	6.38	5.40
COL13A1	5.70	6.11	5.31
RNA5SP139	5.70	6.26	4.94
LOC645166	5.70	6.22	5.03
HSD17B11	5.70	6.26	4.76
SNORD6	5.70	6.21	4.79
MBD3L5	5.70	6.37	5.34
FOXD4	5.70	6.03	5.17
TCFL5	5.70	5.90	5.34
ATG13	5.70	5.99	5.46
STIP1	5.70	6.45	5.29
EIF4E1B	5.70	6.24	5.35
WDFY2	5.70	5.95	5.38
MUC16	5.70	6.17	5.22
DDX21	5.70	6.55	5.05
PI4KB	5.70	6.14	5.27
CCRN4L	5.70	6.13	5.02
FAM160A2	5.70	5.92	5.37
LACTB	5.70	6.47	4.96
BIRC7	5.70	6.24	5.06
FBXL2	5.70	6.12	5.38
MANF	5.70	6.07	5.35
MAMDC2-AS1	5.70	6.42	4.65
FAM111A	5.70	6.23	5.15
SNORD114-15	5.70	7.40	4.39
ZNF33A	5.70	6.05	5.27
FBXL7	5.70	6.16	5.08
RNA5SP59	5.70	6.50	5.13
BAG1	5.70	6.05	4.93
LOC100128751	5.70	6.09	5.05

OTTHUMG00000018660	5.70	6.18	5.26
FAM104A	5.70	6.03	5.19
TRMT5	5.70	6.39	5.15
CD99L2	5.70	5.95	5.11
GSTM2	5.70	6.06	5.45
COG3	5.70	6.10	5.10
PPP6R1	5.70	5.94	5.39
ST5	5.70	5.93	5.34
MSRB3	5.70	6.18	5.31
RNF26	5.70	6.07	5.20
ARHGAP28	5.70	6.36	5.01
ITPKB-IT1	5.70	5.93	5.26
NTRK2	5.70	6.17	5.18
OTTHUMG00000152764	5.70	6.39	5.12
BAMBI	5.70	6.48	5.17
TRIM23	5.70	6.10	5.07
SH3GLB2	5.70	5.85	5.53
SMCR7	5.70	5.96	5.30
ZSCAN9	5.70	6.05	5.33
ZFP90	5.70	6.32	5.30
C6ORF205	5.70	6.43	4.94
PTH1R	5.70	6.20	5.33
KIR2DL1	5.70	6.48	5.25
KCNQ1OT1	5.70	6.11	5.22
BAG3	5.70	6.13	5.03
ATF5	5.70	6.08	5.33
CEP97	5.70	6.11	5.16
FAM213A	5.69	6.30	5.02
EGFL7	5.69	5.90	5.43
CAPG	5.69	6.20	5.03
MAF1	5.69	6.06	5.18
AGPAT6	5.69	6.01	5.23
EIF2B4	5.69	5.93	5.38
KIR3DL3	5.69	6.38	5.00
NUDT9	5.69	6.17	5.36
MZB1	5.69	5.99	5.06
TRAJ39	5.69	6.35	5.28
NEU1	5.69	5.90	5.45
C5AR1	5.69	6.29	4.92
ELMO1	5.69	6.18	5.19
SFT2D1	5.69	6.34	5.20
TFDP1	5.69	6.03	5.21
SOCS3	5.69	6.47	4.99
ZNF451	5.69	5.98	5.26
PARP3	5.69	5.92	5.27
PNPLA6	5.69	6.11	4.86
EHMT2	5.69	5.98	5.48
COG4	5.69	6.02	5.34
SNUPN	5.69	6.03	5.13

PPP2R1B	5.69	6.08	5.32
ORC2	5.69	6.11	5.09
MOGS	5.69	6.07	5.40
CLEC18A	5.69	6.60	5.01
STON1-GTF2A1L	5.69	6.18	5.41
ISG20L2	5.69	6.21	4.99
ZNF460	5.69	6.34	5.17
DOPEY1	5.69	5.91	5.13
PRAMEF15	5.69	6.78	5.17
SNRNP40	5.69	6.18	5.36
DYNC1I1	5.69	6.53	4.42
CYP27A1	5.69	6.10	5.13
SETDB1	5.69	6.02	4.99
TTYH1	5.69	6.33	5.38
IFNGR1	5.69	6.02	5.15
SMG6	5.69	5.94	5.44
TMEM63B	5.69	6.29	5.13
RABL5	5.69	5.89	5.02
CTHRC1	5.69	6.59	4.76
DHRS7B	5.69	6.01	5.17
C16orf54	5.69	6.03	5.42
AGER	5.69	5.99	5.43
PLGLB1	5.69	6.32	4.53
RAPGEF6	5.69	6.06	5.19
PTOV1	5.69	6.11	5.32
OTTHUMG00000150015	5.69	6.16	4.92
GNL3L	5.69	5.94	5.42
FAM153B	5.69	6.22	5.09
NPLOC4	5.69	6.22	5.22
MIR370	5.69	6.39	4.89
ATP7A	5.69	6.18	5.23
ABRACL	5.69	6.39	4.80
GAS1	5.68	6.02	5.12
C14orf119	5.68	6.01	5.27
JMJD6	5.68	6.10	5.28
PTAR1	5.68	6.13	5.01
TNIP2	5.68	5.98	5.30
COMMD1	5.68	5.97	4.99
LMO7	5.68	6.07	5.07
SNX19	5.68	6.18	5.24
BRD4	5.68	5.98	5.15
PLEKHO2	5.68	6.18	5.31
DIS3L	5.68	6.02	5.21
OTTHUMG00000059263	5.68	5.98	5.28
WDR5B	5.68	6.10	5.13
AP3D1	5.68	5.90	5.53
SNORA21	5.68	6.14	5.14
PAPOLG	5.68	6.01	5.29
PTGS1	5.68	6.00	5.24

LOC100288160	5.68	6.10	5.38
WDR20	5.68	6.30	5.09
SPRY4	5.68	6.24	5.19
FAM25G	5.68	6.26	5.16
STX17	5.68	6.12	5.27
BAIAP2L1	5.68	6.61	5.17
PRR3	5.68	6.03	5.22
CCDC28B	5.68	6.20	5.25
RWDD3	5.68	5.83	5.37
NBPF1	5.68	6.48	5.17
LDLRAD4-AS1	5.68	5.89	5.32
MUC21	5.68	6.10	5.35
PAOX	5.68	5.98	5.37
CPNE8	5.68	5.99	5.39
CD86	5.68	6.76	4.80
PPT2-EGFL8	5.68	5.94	5.53
SPTLC3	5.68	6.23	5.10
ZNF784	5.68	6.03	5.15
FAM83E	5.68	6.11	5.38
ABHD17A	5.68	6.31	5.09
FRG1B	5.68	6.27	4.93
COL4A2	5.68	6.25	5.38
ERAP1	5.68	6.12	5.19
PHLPP1	5.68	6.09	5.39
CCT6P3	5.68	6.17	5.18
SNORD114-1	5.68	7.14	3.46
IGHV4-61	5.68	6.10	5.13
ZNRF1	5.68	5.95	5.33
RPL39	5.68	5.82	5.49
DUSP2	5.68	6.10	5.27
ASMTL	5.68	6.01	5.41
LINC00489	5.68	6.04	5.32
LOC100128818	5.68	5.99	5.19
TUB	5.68	5.96	5.00
RNA5SP194	5.68	6.35	5.22
MDFI	5.68	6.67	5.17
CASP4	5.68	6.09	5.18
TRAPPC10	5.68	6.01	5.13
LOC100505828	5.68	6.22	5.34
MIR720	5.68	6.14	5.45
TMEM105	5.68	6.20	5.32
TYW5	5.68	6.21	5.14
CNPY4	5.68	6.10	5.13
COQ10B	5.68	6.22	4.99
HAT1	5.68	6.14	5.06
OPN1SW	5.68	6.30	4.74
ZDHHC18	5.68	5.99	5.17
SEMA3A	5.68	7.26	4.57
EPC1	5.68	6.03	5.25

CD300C	5.68	5.98	5.35
DKK3	5.68	6.23	4.98
PLAU	5.68	6.82	5.06
ZMAT2	5.68	5.88	5.54
CCDC102B	5.68	6.82	4.19
KDR	5.67	7.13	4.39
CCDC92	5.67	5.94	5.39
CCNG2	5.67	5.98	5.11
LGI4	5.67	6.24	4.97
GNB3	5.67	5.92	5.38
ALDH1L2	5.67	6.79	4.82
LOC642633	5.67	6.22	5.34
MIR497HG	5.67	6.62	4.88
SLC35A4	5.67	6.16	5.07
RUNX1-IT1	5.67	7.24	4.49
NUPL2	5.67	5.98	5.28
DNM2	5.67	5.87	5.40
C5	5.67	5.97	5.25
ECI1	5.67	5.84	5.34
DCTN4	5.67	6.01	4.79
CNTLN	5.67	6.38	4.81
OTTHUMG00000154884	5.67	6.17	5.16
HHAT	5.67	6.34	4.68
SPG21	5.67	6.11	5.13
ICAM3	5.67	5.86	5.47
TANC2	5.67	6.51	4.83
ORMDL3	5.67	6.00	5.22
SEPT7P2	5.67	6.35	5.14
CNIH2	5.67	6.03	5.41
LIX1L	5.67	6.62	4.68
OTTHUMG00000073721	5.67	6.14	5.17
BLOC1S5	5.67	6.18	4.76
HMG20A	5.67	5.89	5.49
POLR1A	5.67	5.89	5.45
CACTIN-AS1	5.67	5.95	5.39
ZBTB11	5.67	6.06	5.21
CCNY	5.67	6.29	4.96
VPS41	5.67	6.07	5.30
FAM229B	5.67	6.15	5.27
C19orf55	5.67	5.83	5.57
OTTHUMG00000001770	5.67	6.20	5.20
KIAA1841	5.67	5.98	5.09
SPRYD3	5.67	5.97	5.23
FADS1	5.67	5.93	5.30
TWISTNB	5.67	6.48	4.88
IGHD2-15	5.67	6.48	5.03
PPFIBP2	5.67	6.09	5.31
SIRT1	5.67	6.01	4.70
SCO1	5.67	5.96	5.07

HLA-DOA	5.67	6.01	5.33
MST1	5.67	6.19	5.32
FBXO18	5.67	6.09	5.31
MNT	5.67	5.93	5.34
OTTHUMG00000171661	5.67	6.32	5.00
HNRNPA0	5.67	6.11	5.22
ATAD3A	5.67	6.12	5.04
ANKRD40	5.67	6.02	5.17
ZNF469	5.67	6.05	5.25
PPA2	5.67	6.01	5.17
ATP10A	5.67	6.76	4.97
PHYHD1	5.67	6.05	5.28
ERBB2	5.67	5.87	5.18
C5orf60	5.66	6.64	5.02
PORCN	5.66	5.85	5.30
C21orf128	5.66	6.27	5.34
ABHD13	5.66	6.12	5.07
CA5BP1	5.66	6.03	5.36
OTTHUMG00000171876	5.66	6.49	5.29
RPL15	5.66	5.78	5.54
SMPD1	5.66	5.95	5.31
ZDHHC20	5.66	6.17	5.10
DPF2	5.66	5.95	5.28
C19orf80	5.66	5.99	5.30
IRAK3	5.66	6.11	5.11
LOC101060680	5.66	6.09	5.07
PHF14	5.66	6.05	4.97
LOC339505	5.66	6.15	5.16
SP110	5.66	6.01	5.33
ZNF785	5.66	5.89	5.51
ANO1	5.66	6.46	4.81
SFRP1	5.66	6.53	4.85
KIAA1967	5.66	6.02	5.23
WDR52	5.66	6.01	5.39
CLTB	5.66	6.18	5.32
TMEM80	5.66	5.95	5.40
LAP3	5.66	6.02	5.22
P2RY6	5.66	5.98	5.49
CWC15	5.66	6.12	5.26
ZCCHC2	5.66	5.99	5.22
ACVRL1	5.66	5.90	5.28
MIR4297	5.66	6.54	4.88
LOC100509205	5.66	5.82	5.47
CCNG1	5.66	6.04	5.34
C11orf58	5.66	5.88	5.12
CSTF2T	5.66	6.10	5.08
NACA2	5.66	5.99	5.24
DEFT1P	5.66	6.03	5.37
SURF1	5.66	6.03	5.23

PRPF4	5.66	6.10	5.23
SELPLG	5.66	6.00	5.42
KRT18P54	5.66	6.16	5.13
ARFGAP1	5.66	6.12	5.27
PRUNE2	5.66	6.32	4.53
STON2	5.66	6.42	5.26
TET1	5.66	6.08	4.93
CLPTM1L	5.66	6.02	5.18
ZNF608	5.66	6.09	5.12
IL3RA	5.66	6.09	4.59
OTTHUMG00000167267	5.66	6.09	5.31
B3GALT4	5.66	5.87	5.40
MPHOSPH6	5.66	6.29	5.19
PIGA	5.66	5.92	5.39
EIF3B	5.66	6.13	5.22
MIEN1	5.66	6.41	5.09
GJB2	5.66	7.22	4.60
USP3-AS1	5.66	5.99	5.26
IZUMO4	5.66	6.12	5.32
EFS	5.66	5.93	5.21
AIP	5.66	5.96	5.33
DHX38	5.66	6.19	4.98
MYLK	5.66	5.98	5.25
MIR891A	5.66	6.24	5.04
PVRIG	5.66	5.94	5.10
OTTHUMG00000166785	5.65	6.21	5.00
LRRC37BP1	5.65	6.24	5.05
TMEM184C	5.65	6.11	4.92
ERF	5.65	6.05	5.24
C7orf26	5.65	5.99	5.33
TYSND1	5.65	5.87	5.28
CDH23	5.65	6.55	4.95
CCND3	5.65	6.27	5.17
LOC79999	5.65	6.03	5.12
MAD1L1	5.65	5.81	5.49
SAP30L	5.65	6.20	4.84
C1orf131	5.65	6.18	5.42
CHPT1	5.65	5.90	5.36
DBT	5.65	6.05	5.34
PALLD	5.65	6.40	5.03
MIR197	5.65	6.31	4.65
WDR76	5.65	6.21	5.16
SMYD2	5.65	5.97	5.39
TNS3	5.65	5.95	5.19
NAV2-AS1	5.65	6.49	4.24
BRAT1	5.65	5.84	5.38
AEN	5.65	5.92	5.32
MIR4254	5.65	6.07	5.24
GBA	5.65	6.03	5.29

LOC646214	5.65	6.02	5.04
PCGF1	5.65	6.14	5.38
GALNS	5.65	5.99	5.42
NCF4	5.65	5.91	5.30
TM6SF1	5.65	6.57	4.72
KCNG2	5.65	6.06	5.29
MGAT5	5.65	6.07	5.08
TMEM14B	5.65	6.55	4.89
PTPLB	5.65	6.00	5.15
MRPL17	5.65	6.15	5.18
VSTM2L	5.65	5.97	5.33
GTF3C3	5.65	6.27	5.01
TWF2	5.65	6.08	5.05
RAD54L2	5.65	5.94	5.09
ARHGAP26	5.65	6.08	5.03
RASSF3	5.65	6.07	5.37
PRPF31	5.65	6.02	5.39
MCCD1	5.65	6.44	5.23
HAS3	5.65	6.22	5.18
RUNX1	5.65	6.11	5.16
YARS	5.65	6.40	5.23
ADAM20P1	5.65	5.97	5.24
LOC100129233	5.65	6.09	5.46
DCLRE1B	5.65	6.10	4.99
C22orf26	5.65	6.03	5.22
NSUN2	5.65	6.07	5.18
SNORA84	5.65	5.97	5.37
FGD4	5.65	5.95	5.07
SP4	5.65	6.18	5.28
CACNA1C-AS4	5.65	6.49	4.96
TSPAN4	5.65	5.82	5.50
RTTN	5.65	5.93	5.28
MPRIP	5.65	6.02	5.12
CARD8	5.65	6.07	5.22
ACAD9	5.65	6.13	5.14
ZNF362	5.65	5.98	5.22
CCT8	5.65	6.02	5.38
LSM10	5.65	5.96	5.37
MCCC1-AS1	5.65	6.06	5.29
PIK3R3	5.65	6.19	4.96
TNIP1	5.65	5.85	5.41
FAM25A	5.64	6.17	5.12
PI4KA	5.64	6.12	5.16
DMPK	5.64	5.93	5.42
MLF2	5.64	5.90	5.18
ST6GAL1	5.64	5.98	4.97
UQCRFS1	5.64	6.10	4.88
ACOX1	5.64	5.93	4.93
CCDC23	5.64	6.19	5.23

PHOSPHO1	5.64	6.28	4.88
RUVBL1	5.64	6.01	5.19
SPATA5	5.64	6.32	5.01
DDX19A	5.64	5.97	5.29
ZNF593	5.64	6.03	5.22
SMCR8	5.64	5.89	4.94
LOC100996357	5.64	6.40	5.07
FAM126B	5.64	6.00	5.23
OTTHUMG00000163917	5.64	6.46	4.98
SLC15A3	5.64	5.91	5.37
COX10-AS1	5.64	6.03	5.03
FOXD4L3	5.64	6.31	5.00
MYH11	5.64	6.28	5.22
PDK4	5.64	6.40	4.69
SMAD6	5.64	6.23	4.83
CREG1	5.64	5.97	5.07
AZIN1	5.64	6.08	5.05
MKKS	5.64	6.07	4.85
METTL15	5.64	6.05	5.37
ZNF37A	5.64	5.91	5.39
INSIG1	5.64	6.07	5.31
POLD3	5.64	5.84	5.34
ATP6V1E1	5.64	5.97	5.11
OR2A1	5.64	6.27	5.13
RRP7A	5.64	6.08	5.25
RUFY1	5.64	6.03	5.20
MKX	5.64	6.37	5.11
LOC338797	5.64	6.25	5.06
THPO	5.64	6.02	5.33
GON4L	5.64	5.88	5.25
DDX26B	5.64	6.01	4.98
KPNA1	5.64	5.95	5.01
NDUFA10	5.64	6.05	5.03
CCP110	5.64	6.37	4.98
ALS2	5.64	5.91	5.21
IL17RE	5.64	5.98	5.42
ANKRD20A2	5.64	6.12	5.18
INTS5	5.64	6.32	5.01
DVL3	5.64	6.15	5.15
TPSB2	5.64	6.13	5.31
LOC100505710	5.64	6.11	5.23
SYNM	5.64	6.09	5.27
MGRN1	5.64	5.96	5.33
SMEK2	5.64	6.19	5.07
OTTHUMG00000041460	5.64	6.09	5.22
OTTHUMG00000019603	5.64	6.19	5.24
ERLIN2	5.64	5.92	5.43
SUGP2	5.64	5.87	5.10
MAGI2-AS1	5.64	6.22	4.98

SLC26A11	5.64	5.87	5.24
THOC1	5.64	6.08	5.20
PIGN	5.64	6.35	5.15
GALNT2	5.64	6.31	5.22
ZNF490	5.64	5.81	5.37
THOC6	5.64	5.89	5.35
ZXDA	5.64	6.06	4.92
MUC3A	5.64	6.58	4.92
FAM174A	5.64	6.06	4.84
OXNAD1	5.64	5.84	5.31
ZBTB40	5.64	5.99	5.09
RBM33	5.64	6.13	5.18
ARFRP1	5.64	6.13	5.41
TPTEP1	5.64	6.16	5.03
EHBP1	5.64	6.05	5.12
OTTHUMG00000045359	5.64	5.97	5.30
DHDDS	5.63	5.88	5.32
NDUFB4	5.63	6.02	5.13
LOC255654	5.63	6.23	4.85
NUBP1	5.63	6.19	4.83
PSORS1C2	5.63	6.25	5.39
TTI1	5.63	5.99	5.34
ZNF226	5.63	6.05	5.20
B3GALNT1	5.63	5.98	5.06
SEC16A	5.63	5.96	5.21
OGFOD2	5.63	5.80	5.47
OTTHUMG00000013217	5.63	5.97	5.23
SLC46A3	5.63	6.31	5.00
MAGEA9	5.63	6.07	5.35
NKX1-2	5.63	6.11	5.20
DISP1	5.63	5.88	5.11
GPRASP1	5.63	6.09	5.29
RIOK2	5.63	6.13	5.26
BOK	5.63	5.97	5.37
ATP6V0B	5.63	5.97	5.32
PLA2R1	5.63	6.50	4.64
TEKT4	5.63	6.06	5.12
STRA13	5.63	5.91	5.26
EBAG9	5.63	5.96	5.11
MXD4	5.63	5.96	5.29
ALDH3B1	5.63	5.93	5.18
MFI2	5.63	6.25	5.15
LOC100133920	5.63	5.94	5.20
B3GNT1	5.63	5.87	5.47
HSD17B1	5.63	5.98	4.94
P2RX5-TAX1BP3	5.63	5.92	5.41
HYAL2	5.63	5.88	5.14
DIP2B	5.63	6.13	5.25
LOC100128077	5.63	5.90	5.44

FLJ41733	5.63	5.91	5.21
KGFLP2	5.63	6.17	4.72
FRMD4B	5.63	6.28	4.89
MYL7	5.63	5.84	5.41
PQLC1	5.63	5.91	5.23
MIR3934	5.63	6.12	5.16
TINF2	5.63	6.03	4.97
TCF7L1	5.63	6.00	5.22
GCN1L1	5.63	6.02	5.19
DCK	5.63	6.08	4.99
MGC4294	5.63	6.23	5.10
RNF34	5.63	6.11	5.09
CNEP1R1	5.63	6.20	4.83
SNORD23	5.63	6.11	5.10
VWA5A	5.63	6.07	5.14
PARD3	5.63	6.23	5.22
PRAMEF5	5.63	6.08	5.13
RNA5SP406	5.63	6.06	5.20
RAB21	5.63	6.21	4.91
DKC1	5.63	6.06	5.12
LINC00910	5.63	5.97	5.14
SYDE1	5.63	5.98	5.24
MTF2	5.63	6.02	5.25
HEXDC	5.63	5.99	5.13
LRP12	5.63	6.66	4.85
FAN1	5.63	6.35	5.01
C19orf71	5.63	5.93	5.35
CEP68	5.62	5.88	5.42
BCKDK	5.62	5.90	5.42
ARFIP2	5.62	5.89	4.85
OTTHUMG00000180685	5.62	6.36	4.92
CRYGC	5.62	6.06	5.19
GSE1	5.62	6.03	5.12
APITD1-CORT	5.62	5.94	5.40
PNPLA2	5.62	5.90	5.35
CSF2RA	5.62	5.95	5.28
BCS1L	5.62	5.89	5.36
OTTHUMG00000018330	5.62	6.71	4.23
CEP120	5.62	6.00	5.12
NACAD	5.62	6.03	4.88
SHKBP1	5.62	5.78	5.33
ITPR3	5.62	6.14	4.78
KRTAP2-1	5.62	6.19	5.07
TTC13	5.62	5.95	4.96
LIMK2	5.62	5.90	5.34
ABCF2	5.62	6.05	5.31
ANKRD16	5.62	6.04	5.05
PSMD3	5.62	5.97	5.28
CKS2	5.62	6.68	4.38

HBE1	5.62	6.13	5.11
MIR3944	5.62	6.10	5.28
UBE2V2	5.62	6.01	5.33
S100A2	5.62	5.92	5.30
OTTHUMG00000058631	5.62	6.39	5.09
SMNDC1	5.62	5.98	5.23
BMP8A	5.62	6.50	5.23
C16orf58	5.62	5.90	5.01
HSD17B7P2	5.62	5.98	5.08
FAM210B	5.62	6.23	4.86
SCOC	5.62	5.97	5.25
OTTHUMG00000150793	5.62	6.32	4.92
MEG8	5.62	6.89	4.81
GORASP1	5.62	5.92	5.29
MIR3918	5.62	5.95	5.03
TTN-AS1	5.62	6.05	5.16
MFSD6	5.62	5.86	5.26
CSAG2	5.62	5.89	5.23
OTTHUMG00000163201	5.62	5.96	5.11
SSU72	5.62	5.98	5.16
LRRN2	5.62	5.85	5.42
OR5C1	5.62	5.95	5.41
PDE4A	5.62	5.94	5.11
KCTD20	5.62	6.07	5.08
EDC3	5.62	6.22	5.01
ARPC5L	5.62	5.95	5.17
LOC388849	5.62	5.99	5.33
MATN4	5.62	6.06	5.28
TST	5.62	5.79	5.30
OTTHUMG00000177158	5.62	5.98	5.10
HSPA2	5.62	6.45	4.93
OTTHUMG00000175726	5.62	6.23	5.22
LRRC8E	5.62	6.44	4.98
BRD8	5.62	5.91	5.39
IRF2BPL	5.62	5.99	5.24
GOLGA1	5.62	5.87	5.15
ZNF746	5.62	5.92	5.36
SPA17	5.62	5.91	4.99
CDK18	5.62	6.20	5.18
TMEM255B	5.62	5.87	5.31
LOC100505616	5.62	5.96	5.26
CD14	5.62	6.27	5.13
ING2	5.62	6.14	4.89
TCEB1	5.62	6.26	4.96
SLC25A40	5.62	6.00	5.15
INPP5K	5.62	5.79	5.46
SYNJ1	5.62	6.03	4.77
SLC35B4	5.62	6.35	4.50
PSTK	5.62	5.97	5.34

RHOT1	5.62	6.22	4.54
LOC727710	5.62	6.17	5.09
GPAA1	5.62	5.93	5.31
PGRMC2	5.62	6.07	5.33
KIAA1704	5.62	6.01	5.16
NAV2	5.62	6.06	4.96
CARS	5.61	6.15	4.82
LOC100507463	5.61	6.06	5.23
DOCK8	5.61	6.11	5.15
NDE1	5.61	5.97	5.34
ZNF436	5.61	6.41	4.52
ZFAND2A	5.61	6.05	5.24
CELA2A	5.61	6.22	5.29
AP1B1	5.61	6.13	5.07
DKFZP586I1420	5.61	6.24	5.05
RAI14	5.61	6.65	4.87
KPNA5	5.61	5.95	5.25
F13A1	5.61	6.79	4.68
NAT10	5.61	5.89	5.27
RABGGTB	5.61	6.10	5.14
HECW2	5.61	6.44	5.11
CPSF1	5.61	6.05	5.13
TMEM258	5.61	5.84	5.35
SLC1A5	5.61	6.21	4.75
RASL10B	5.61	6.12	5.25
LOC286297	5.61	6.06	5.30
SYTL4	5.61	6.13	4.90
MYCBP2-AS1	5.61	5.89	5.32
ULBP2	5.61	6.18	4.80
MDH1	5.61	5.84	5.28
NTMT1	5.61	6.11	5.27
KIAA0907	5.61	5.79	5.24
AASDH	5.61	5.81	5.13
INTS2	5.61	6.06	5.10
LOC401321	5.61	5.87	5.00
SFRP2	5.61	6.97	4.68
PACS1	5.61	6.00	5.17
FAM86HP	5.61	6.03	4.99
FAM225A	5.61	6.23	4.96
HLA-V	5.61	5.87	5.31
LOC554223	5.61	5.87	5.31
BMP2	5.61	6.25	4.87
VPS37A	5.61	6.17	5.01
C20orf166-AS1	5.61	6.18	5.11
OTTHUMG00000180722	5.61	6.02	5.34
C15orf65	5.61	6.32	4.89
C9orf50	5.61	5.80	5.41
ITM2C	5.61	6.29	5.21
C1RL	5.61	6.03	5.34

HOXB-AS4	5.61	6.04	5.34
LSR	5.61	5.99	5.10
TSEN2	5.61	5.97	5.23
TMEM130	5.61	6.14	5.29
MIR4640	5.61	5.84	5.31
P4HB	5.61	6.03	5.13
HRH1	5.61	6.20	5.13
MAML1	5.61	5.85	5.21
PML	5.61	5.81	5.44
SREK1IP1	5.61	5.93	5.18
MIR4312	5.61	6.10	5.07
HSF4	5.61	6.16	5.31
RNF167	5.61	5.84	5.22
HOXD4	5.61	6.06	5.12
PER2	5.61	6.10	4.95
MTX2	5.61	6.29	5.01
ASB1	5.61	5.97	5.34
SDK2	5.61	5.97	5.11
AZI1	5.61	5.85	5.26
LOC100132999	5.61	5.90	5.24
GSDMD	5.61	5.94	5.25
GAS6	5.61	6.19	4.81
FAM87B	5.61	5.88	5.23
CWC25	5.61	5.88	4.95
IL18BP	5.61	5.94	5.33
HPS5	5.61	5.84	5.39
SAR1B	5.61	5.98	4.96
TRNAU1AP	5.61	5.99	5.03
EIF6	5.61	6.09	5.15
NABP1	5.61	6.13	5.12
LOC100505771	5.61	6.04	5.04
FLNC	5.61	5.96	5.36
ZFYVE9	5.61	5.82	5.13
PSEN1	5.61	5.91	5.13
PLEKHG2	5.61	5.85	5.04
C3orf62	5.61	5.88	5.32
ST7-OT4	5.61	5.91	5.17
KLF9	5.61	6.41	4.71
PCNXL2	5.60	5.85	5.28
CNIH4	5.60	6.25	5.12
SPDYE5	5.60	6.16	4.27
LOC100288152	5.60	5.96	5.31
RRN3P2	5.60	6.12	5.04
SHOX2	5.60	5.87	5.12
GPD1L	5.60	5.99	5.39
UPK2	5.60	5.95	4.98
TRAF6	5.60	5.88	5.31
ZMYM3	5.60	5.99	5.13
UBE2E3	5.60	5.92	4.78

EMC1	5.60	5.85	5.15
LSM4	5.60	5.97	5.17
DUSP3	5.60	6.37	4.87
LOC100507437	5.60	6.10	5.27
TMBIM1	5.60	6.04	4.97
HHIP	5.60	6.80	4.68
ACTR8	5.60	5.93	4.85
LUZP1	5.60	6.09	5.28
KPNA4	5.60	6.17	4.94
A4GALT	5.60	5.96	4.79
TNFSF13B	5.60	6.17	5.29
NUBP2	5.60	6.02	5.20
MIR2113	5.60	6.03	5.37
OTTHUMG00000171614	5.60	6.06	5.20
FKBP8	5.60	5.89	5.00
RBM22	5.60	5.90	5.28
MFAP2	5.60	6.53	4.68
DHRS4-AS1	5.60	5.85	5.37
RCAN2	5.60	6.23	4.65
UTY	5.60	6.69	3.48
PLA2G12A	5.60	5.99	4.98
LOC100128172	5.60	6.14	5.31
HBEGF	5.60	6.18	4.55
RNA5SP444	5.60	6.31	5.05
PRRX2	5.60	6.04	5.26
ADC	5.60	5.90	5.16
MGC10955	5.60	6.20	5.06
ETNK1	5.60	5.93	4.97
SCNM1	5.60	6.01	5.22
ALKBH6	5.60	5.96	5.32
MAGI1	5.60	5.97	5.00
ZNF813	5.60	6.18	4.99
TMEM191B	5.60	6.09	5.33
GPSM1	5.60	5.79	5.34
ABHD12	5.60	6.31	4.92
EDNRA	5.60	6.70	4.93
PPP4C	5.60	6.13	4.67
SLC26A2	5.60	5.88	5.02
RPL30	5.60	5.80	5.36
HR	5.60	5.90	5.11
MIER3	5.60	5.73	5.41
PPHLN1	5.60	5.89	5.04
OTTHUMG00000170889	5.60	6.37	4.68
LPIN2	5.60	5.92	5.27
FZD5	5.60	5.89	5.37
INPP4A	5.60	5.90	5.12
U2AF2	5.60	5.94	5.10
C12orf10	5.60	6.01	5.08
GABPB1-AS1	5.60	5.86	4.92

COPE	5.60	5.92	5.00
PSMG4	5.60	6.04	5.26
CHRNE	5.60	6.01	5.26
RPS19BP1	5.60	5.81	5.36
FASTK	5.60	5.75	5.40
TOX2	5.60	6.25	5.19
H2AFV	5.60	6.06	4.76
HMGCS1	5.60	6.14	5.07
FGFR1OP	5.60	6.01	5.14
ZNF766	5.60	6.20	5.27
INTS1	5.60	5.69	5.42
CACYBP	5.60	5.88	5.02
TMEM200A	5.60	6.43	5.09
IL15RA	5.60	5.92	5.26
THADA	5.59	5.82	5.27
MIR3922	5.59	6.03	4.96
FAM109B	5.59	6.05	5.35
SSR4	5.59	5.86	5.40
WDR1	5.59	5.95	5.17
ATXN7L1	5.59	5.81	5.35
SPATA25	5.59	6.15	5.13
SYTL2	5.59	6.12	5.23
KLK5	5.59	6.10	5.10
KLHL29	5.59	5.95	4.91
NKD1	5.59	5.93	5.31
SIRT7	5.59	5.84	5.11
HAND2	5.59	6.06	4.93
OTTHUMG00000167197	5.59	5.99	5.06
CBX1	5.59	5.88	5.29
CCDC174	5.59	6.06	4.84
UBXN2A	5.59	5.84	5.21
OTTHUMG00000002202	5.59	6.04	5.08
SUPV3L1	5.59	6.01	5.28
GUK1	5.59	5.97	5.14
ZNF256	5.59	5.91	4.87
LRCH1	5.59	6.15	4.97
TRAK1	5.59	5.83	5.36
GATA1	5.59	5.95	5.15
WDR44	5.59	6.01	4.79
ZNF85	5.59	5.84	5.07
CHTF18	5.59	5.83	5.31
LOC338667	5.59	6.04	5.02
WNT1	5.59	6.00	5.19
GPATCH2L	5.59	6.22	4.93
SLC4A3	5.59	5.94	5.24
FAM134A	5.59	6.00	5.39
LGALS9C	5.59	6.09	5.12
C12orf73	5.59	6.02	4.78
GUSBP4	5.59	5.83	5.09

PID1	5.59	6.85	4.85
GNRH2	5.59	6.07	5.24
MRPS14	5.59	6.16	4.73
MYEOV2	5.59	6.05	5.06
SMN2	5.59	6.04	5.35
ZC3H4	5.59	5.78	5.25
HAUS4	5.59	5.81	5.41
SGPP1	5.59	5.96	5.23
POM121L8P	5.59	6.42	5.06
OR2M7	5.59	6.95	4.85
MNS1	5.59	6.06	5.13
DPP7	5.59	6.06	5.03
NUP37	5.59	6.26	4.80
QARS	5.59	5.83	5.33
ZNFX1	5.59	5.89	5.20
PTGDR2	5.59	6.06	4.97
TKT	5.59	5.95	5.33
DDX56	5.59	5.99	5.12
AURKB	5.59	6.09	4.81
TUBA4A	5.59	5.98	5.29
FAM63A	5.59	5.94	5.03
SNORA15	5.59	6.08	5.33
TOM1L2	5.59	5.78	5.28
TOR3A	5.59	5.78	5.25
MTAP	5.59	5.99	5.28
SPSB3	5.59	5.88	5.21
FDX1	5.59	5.93	5.13
TPRKB	5.59	6.11	5.04
ZNF582-AS1	5.59	5.94	4.98
ARRDC4	5.59	6.03	5.12
ECSIT	5.59	6.09	4.77
GUCY1B3	5.59	6.16	5.16
PTPRE	5.59	6.06	5.10
CFD	5.59	6.06	4.97
KLK2	5.59	5.98	5.23
SLC16A3	5.59	6.10	5.22
FBXO21	5.59	5.91	5.37
KLK8	5.59	5.88	5.06
MUM1	5.59	5.93	5.29
ECHDC1	5.59	5.87	5.08
PRR26	5.59	5.95	5.13
OTTHUMG00000058163	5.59	6.12	5.29
PTH2	5.59	5.97	5.30
KLHDC4	5.59	5.80	5.39
C1orf162	5.59	6.69	5.03
RPS24	5.59	5.72	5.39
PRPF18	5.59	5.82	5.01
PTGER2	5.59	5.93	5.24
MAK	5.59	5.90	5.35

MAP4K3	5.59	5.92	5.18
CTRL	5.58	5.83	5.02
CTSW	5.58	5.98	5.29
SNORD115-39	5.58	6.68	4.63
ZNF324B	5.58	6.03	5.35
RDH5	5.58	5.82	5.34
SERF2	5.58	5.93	5.22
GHRLOS	5.58	5.89	5.32
PPP5C	5.58	5.82	5.13
SNX11	5.58	6.00	5.21
GPATCH1	5.58	5.89	5.35
RHOF	5.58	6.05	5.06
PDE6D	5.58	5.88	5.12
KCNMB1	5.58	5.98	5.13
ROR2	5.58	6.48	5.15
CAMKK2	5.58	6.02	4.96
OR1S2	5.58	6.22	4.69
ZNF800	5.58	5.97	5.13
GPRC5B	5.58	6.11	4.44
IGFALS	5.58	5.96	5.16
FAM102B	5.58	6.51	4.22
CXXC1	5.58	5.91	4.78
SLC48A1	5.58	5.89	5.14
FLOT2	5.58	6.07	5.32
ZNF703	5.58	6.00	5.16
PEX6	5.58	5.76	5.45
LGALS4	5.58	6.08	5.24
MEG9	5.58	5.89	5.24
FLJ21369	5.58	5.91	5.13
ENDOD1	5.58	6.00	4.83
POM121L1P	5.58	6.07	4.77
NPRL3	5.58	5.91	5.13
CEP164	5.58	5.81	5.35
WDR90	5.58	5.75	5.39
GPS1	5.58	5.83	5.29
METTL9	5.58	6.46	4.47
MICAL2	5.58	6.57	5.06
MRPL45	5.58	6.02	4.75
EVL	5.58	6.13	5.07
PDDC1	5.58	5.86	5.40
KATNAL1	5.58	6.25	4.83
LCOR	5.58	5.99	4.80
RBBP9	5.58	6.22	4.81
CERS5	5.58	6.22	4.92
PCP4L1	5.58	6.41	5.29
VKORC1	5.58	6.14	5.07
WDFY1	5.58	6.17	4.90
CHMP2B	5.58	6.00	4.88
PIK3R4	5.58	5.90	5.13

DPY19L2	5.58	6.10	4.70
ATE1	5.58	5.87	5.16
COMTD1	5.58	6.16	5.19
POU5F1P3	5.58	6.19	4.68
ZNF251	5.58	5.78	5.34
COMMD5	5.58	6.01	5.18
SLC25A29	5.58	5.82	5.15
NEK3	5.58	6.05	4.79
KXD1	5.58	6.12	4.66
POLR3H	5.58	6.06	5.21
DGCR8	5.58	6.09	5.11
PEG3-AS1	5.58	6.71	5.09
HCG4	5.58	6.32	5.05
LOC285463	5.58	6.06	5.27
PLIN1	5.58	6.32	4.47
TBC1D13	5.58	5.91	5.18
SHISA4	5.58	5.84	5.26
ACTR10	5.58	5.98	4.99
DTWD1	5.58	6.20	4.98
LIN7B	5.58	5.84	5.16
SREBF2	5.58	5.92	5.16
LOC100506651	5.58	6.00	4.98
C15orf61	5.58	6.00	5.25
OR4F29	5.57	5.83	5.15
ZNF692	5.57	5.85	5.06
RIC8A	5.57	5.99	5.42
NKG7	5.57	5.75	5.33
MTIF3	5.57	5.89	5.03
PDXK	5.57	6.13	5.09
ADCK3	5.57	5.80	5.29
ODF3L2	5.57	6.05	5.25
ZNF624	5.57	5.83	5.36
PHF20	5.57	5.90	5.11
P2RY2	5.57	5.94	5.20
C17orf97	5.57	6.11	5.02
PTPN21	5.57	6.24	4.93
RAP2A	5.57	6.30	4.62
LOC441242	5.57	6.32	5.22
RPLP1	5.57	6.20	4.42
LYG1	5.57	6.06	4.52
ASB13	5.57	5.86	5.30
EXOC7	5.57	5.94	5.16
ICAM1	5.57	5.95	5.38
SAMD9	5.57	6.27	5.01
TIMM22	5.57	6.18	5.05
RNA5SP278	5.57	6.31	4.85
LOC284373	5.57	5.74	5.19
TBKBP1	5.57	5.93	5.30
HIGD1B	5.57	6.77	4.89

LY86	5.57	6.65	5.20
RNF224	5.57	6.18	5.08
RARB	5.57	6.31	4.70
OTTHUMG00000150945	5.57	5.95	5.07
CCDC91	5.57	5.92	5.16
LEAP2	5.57	5.88	5.23
FAM204A	5.57	5.97	5.20
PDZD11	5.57	6.01	4.92
RAB5C	5.57	5.92	4.88
CTNNAL1	5.57	6.02	4.73
ZNF575	5.57	5.75	5.13
SNRK-AS1	5.57	5.91	5.11
SLC39A11	5.57	5.94	4.71
SSBP1	5.57	5.84	5.15
ZMYND19	5.57	5.96	5.25
RNA5SP447	5.57	6.95	4.49
G0S2	5.57	6.44	4.55
CITED2	5.57	5.89	5.03
VAMP1	5.57	5.97	5.22
ZDBF2	5.57	6.41	4.79
MAP7D1	5.57	5.99	5.21
PYCR1	5.57	5.94	5.31
AP4E1	5.57	6.00	5.00
CNTNAP3	5.57	6.50	4.59
OTTHUMG00000041543	5.57	5.92	4.69
PARD3B	5.57	5.84	5.22
NME7	5.57	6.13	5.18
LINC00328	5.57	6.13	4.85
POLR2C	5.57	5.87	5.09
MIR3925	5.57	6.07	5.06
EMC10	5.57	5.81	5.37
ZNF844	5.57	5.87	4.94
HCK	5.57	6.14	4.99
C16orf98	5.57	5.82	5.38
ANAPC4	5.57	5.88	5.28
HPRT1	5.57	6.20	4.96
ASPG	5.57	5.97	5.29
C2orf82	5.57	6.11	5.03
C3orf38	5.56	6.05	4.80
MASTL	5.56	6.23	5.14
STARD3	5.56	5.88	5.30
WDR53	5.56	5.79	5.16
OTTHUMG00000170596	5.56	5.89	5.33
CXCL16	5.56	7.29	4.72
TCP11L1	5.56	6.00	5.05
ADAMTS14	5.56	6.07	5.32
LOC400548	5.56	5.92	4.98
LDOC1	5.56	5.81	5.32
GPR157	5.56	5.84	5.29

GIMAP6	5.56	5.91	4.92
RDH11	5.56	5.82	5.10
AKTIP	5.56	6.24	4.66
OTTHUMG00000152444	5.56	5.97	5.28
USP45	5.56	6.02	4.97
SMPD4	5.56	5.93	5.06
DESI2	5.56	6.61	4.73
C12orf68	5.56	5.91	5.16
ARHGAP23	5.56	5.81	5.12
LOC646762	5.56	5.91	5.18
DGCR6L	5.56	6.00	4.62
TMEM185A	5.56	5.92	5.29
DOCK5	5.56	5.94	4.97
VPS26B	5.56	5.95	5.04
TAF5L	5.56	5.89	5.08
TMA7	5.56	5.95	5.37
BBS10	5.56	5.91	4.99
TRAF3IP1	5.56	5.81	5.19
SLC9A6	5.56	6.02	4.85
SLC2A13	5.56	5.82	5.34
SYT5	5.56	5.94	4.83
C2orf27A	5.56	6.00	5.13
TOMM40	5.56	5.89	5.30
JOSD1	5.56	5.97	4.93
DBNL	5.56	5.74	5.09
PRKD1	5.56	6.03	5.16
CSPG5	5.56	6.04	5.22
CHST11	5.56	5.98	5.26
SAMM50	5.56	6.09	5.00
ZNF514	5.56	6.00	4.47
TTC39B	5.56	5.98	5.14
LENG8-AS1	5.56	5.99	5.14
HEPN1	5.56	6.04	4.92
SIGLEC16	5.56	6.35	4.95
CCDC12	5.56	5.85	5.15
PMS2P3	5.56	6.06	4.98
LOC728208	5.56	6.15	5.09
FAM95B1	5.56	6.32	4.92
SGMS2	5.56	6.24	4.85
AGPAT5	5.56	6.01	5.03
MRPL48	5.56	5.98	5.12
SEPT10	5.56	6.18	4.65
LOC100507918	5.56	5.73	5.16
CCDC86	5.56	5.86	5.31
TMED9	5.56	5.83	5.05
TSC22D1	5.56	5.86	5.24
LOC100996664	5.56	5.99	5.25
TBC1D2B	5.56	5.93	4.94
ZNF770	5.56	6.02	4.96

OTTHUMG00000132657	5.56	6.20	5.11
PHLDB1	5.56	5.98	5.08
C11orf73	5.56	5.84	5.23
ACAT1	5.56	6.01	4.82
FBXL8	5.56	6.01	5.21
EEF2K	5.56	5.93	4.86
LOC100507346	5.56	6.61	4.10
SULT2B1	5.56	5.98	5.31
TRBV20OR9-2	5.56	5.84	5.03
LSG1	5.56	5.76	5.41
PRKAB1	5.56	5.86	5.25
SH3RF1	5.56	5.96	5.13
CTAGE6	5.55	6.20	4.74
MIR33B	5.55	6.31	5.06
TNRC6C	5.55	6.10	5.05
RNF7	5.55	6.13	4.97
CNN1	5.55	5.93	5.12
EMILIN3	5.55	6.29	4.75
FCN1	5.55	5.96	5.25
DOCK10	5.55	6.30	5.09
SNORD4B	5.55	6.52	4.91
GLE1	5.55	5.79	5.27
CHMP1A	5.55	5.78	5.16
ZNF629	5.55	5.95	5.30
GABPB1	5.55	6.04	5.09
BBS4	5.55	5.89	5.33
OPN1MW	5.55	6.05	5.05
DCUN1D5	5.55	6.16	4.95
OR2W3	5.55	6.12	5.20
GLRX2	5.55	6.24	5.03
RNA5SP408	5.55	6.00	5.21
RBM23	5.55	5.95	4.96
OTTHUMG00000170768	5.55	5.89	5.27
SST	5.55	6.07	5.03
DNASE1L1	5.55	5.96	5.06
IFT81	5.55	5.80	5.27
RAD9A	5.55	5.93	5.05
MAP3K5	5.55	6.15	4.91
NAV2-AS2	5.55	5.96	5.25
ZFP69	5.55	5.90	5.13
FKBP5	5.55	6.61	4.92
NME4	5.55	5.78	5.32
MIR181C	5.55	6.13	5.14
GLIPR2	5.55	6.18	4.64
KIF18B	5.55	5.92	5.27
KLHL5	5.55	5.98	5.14
EFCAB13	5.55	6.35	4.75
CAPZA1	5.55	6.08	4.84
LINC00839	5.55	5.78	5.37

SNORD114-27	5.55	6.79	4.76
G3BP2	5.55	5.88	5.16
SLC46A1	5.55	5.86	5.25
PMS2P1	5.55	5.94	5.19
OTTHUMG00000169707	5.55	6.03	5.17
RPL7L1	5.55	5.70	5.28
CPEB4	5.55	5.88	5.15
PRPSAP2	5.55	5.82	5.33
PGS1	5.55	6.01	5.06
NSFL1C	5.55	5.95	5.16
NFKBID	5.55	6.04	4.98
MANBAL	5.55	5.75	5.35
CSPG4P5	5.55	6.07	5.07
SP3	5.55	5.89	4.94
IGLV10-54	5.55	5.83	5.26
ASNA1	5.55	6.01	5.24
NRSN2	5.55	5.82	5.02
STIM1	5.55	5.78	5.30
DOCK6	5.55	5.97	5.16
RPS20	5.55	5.75	5.30
OTTHUMG00000169058	5.55	6.40	5.16
PLEC	5.55	5.75	5.18
TRAF7	5.55	5.79	5.23
FAM200B	5.55	5.76	5.27
SRR	5.55	6.14	4.85
FBXO27	5.55	5.89	4.95
NKAP	5.55	6.15	5.14
HEMK1	5.55	5.97	5.24
LPAR4	5.55	6.02	4.81
ADIG	5.55	5.88	5.14
OTTHUMG00000175982	5.55	5.78	5.28
LOC100507487	5.55	6.02	5.09
PDLIM4	5.55	5.99	5.22
ERP29	5.54	5.69	5.38
SNORD114-17	5.54	7.10	4.62
KLF4	5.54	5.79	5.29
SMIM13	5.54	5.92	5.19
MID2	5.54	6.23	4.61
RAD21	5.54	5.83	5.29
MEAF6	5.54	5.83	5.15
LOC100286925	5.54	6.02	5.36
SNORD42A	5.54	5.90	5.26
MIR3666	5.54	5.81	5.16
MYOG	5.54	6.08	5.26
SCAF1	5.54	5.98	4.79
C1GALT1C1	5.54	5.81	5.27
TCTN1	5.54	6.01	5.25
UBALD1	5.54	5.81	4.84
ZBTB37	5.54	5.85	5.07

BARX1	5.54	5.79	5.25
C5orf45	5.54	5.77	5.31
CLPB	5.54	5.81	5.34
CCSAP	5.54	5.82	5.14
PPM1N	5.54	6.01	5.08
RBM17	5.54	5.84	5.01
SS18L1	5.54	5.76	5.37
ZMAT3	5.54	5.95	5.19
RAB28	5.54	5.91	5.05
GYPC	5.54	5.93	5.28
BLOC1S2	5.54	6.00	4.94
SPATA13	5.54	5.90	5.32
MIR3161	5.54	6.52	4.46
UBE2I	5.54	5.88	5.12
TARBP2	5.54	5.78	5.30
PFDN6	5.54	5.82	5.33
NSMCE2	5.54	5.83	5.19
BCAP31	5.54	6.15	5.03
FBXL19	5.54	5.85	5.34
SLC15A4	5.54	6.18	5.06
SNRNP35	5.54	5.83	5.20
NBN	5.54	5.93	4.87
ZNF300	5.54	6.13	4.64
FKTN	5.54	5.90	5.09
RAD17	5.54	6.04	5.09
WWC3-AS1	5.54	5.83	5.08
LOC100129361	5.54	6.03	4.77
FURIN	5.54	6.05	5.22
RNF149	5.54	6.00	4.98
LOC100506821	5.54	6.17	5.01
PKN1	5.54	6.29	4.75
NCAPD2	5.54	5.84	4.81
THYN1	5.54	5.81	5.22
PLEKHF2	5.54	6.24	5.01
TIMM50	5.54	5.97	5.02
ZNF483	5.54	6.25	4.76
NFATC1	5.54	5.82	5.30
TRIM2	5.54	5.75	5.11
ZNF419	5.54	5.72	5.33
GAR1	5.54	5.91	5.16
MOV10	5.54	5.82	5.27
ZNF764	5.54	6.20	4.97
MPG	5.54	5.79	5.06
LOC644841	5.54	5.88	5.05
GPIHBP1	5.54	5.92	4.83
KIF3A	5.54	6.09	4.86
UNC5B	5.54	6.28	4.81
MCFD2	5.54	5.77	4.99
RRP15	5.54	6.12	4.87

FKSG29	5.54	5.96	5.23
KIAA1324L	5.54	6.04	5.03
RNF166	5.54	5.85	5.31
SLC41A2	5.54	6.50	4.58
TCF20	5.54	5.91	5.15
NUP133	5.54	5.93	5.04
CPT1C	5.54	5.79	5.29
MIR4324	5.54	6.43	4.37
CPSF2	5.54	6.17	4.83
LRPAP1	5.54	5.84	5.22
OR52W1	5.54	6.03	5.03
ZFAND1	5.54	5.97	5.16
RGL2	5.54	5.86	5.12
VWA8	5.54	5.96	5.01
COX10	5.53	6.27	5.08
FOXK2	5.53	5.76	5.29
STX6	5.53	6.00	5.16
CDK5R2	5.53	6.01	5.14
LOC100133299	5.53	6.33	4.76
VSIG10L	5.53	5.75	4.87
TOMM5	5.53	6.04	4.83
RNU7-57P	5.53	5.87	4.65
GLRX5	5.53	5.90	4.99
CHD1	5.53	5.82	4.84
MEOX1	5.53	5.79	5.14
PIGS	5.53	6.03	5.23
CD37	5.53	6.13	5.15
TEP1	5.53	5.71	5.44
TOM1	5.53	5.84	5.05
SNORD114-2	5.53	7.51	4.10
SMG5	5.53	5.69	5.29
LINC00896	5.53	5.82	5.04
LOC100289511	5.53	5.81	5.19
ZNF32	5.53	5.87	5.22
ALG3	5.53	5.94	5.19
DDX11L1	5.53	6.42	4.87
MIR4502	5.53	6.07	4.73
ELK4	5.53	5.91	5.11
CXorf23	5.53	5.93	4.75
ADCK2	5.53	5.86	5.16
ZBTB20-AS1	5.53	6.11	5.19
FAM225B	5.53	6.14	4.54
LHCGR	5.53	5.97	5.20
C1QB	5.53	6.42	4.53
USP30	5.53	5.75	5.33
RABGAP1L	5.53	5.92	5.03
UPF1	5.53	5.81	5.11
AOC2	5.53	6.05	4.70
PIGL	5.53	5.99	4.72

RPRD1A	5.53	5.90	5.18
FAM134C	5.53	6.08	5.09
LOC100996419	5.53	5.88	4.77
MIR4730	5.53	6.13	5.14
SPATC1	5.53	6.24	4.71
PGM1	5.53	6.09	4.82
TSPAN17	5.53	5.80	5.11
MICU1	5.53	6.06	4.92
C7orf65	5.53	5.99	5.25
APOA5	5.53	5.83	5.15
HOXC-AS5	5.53	6.02	5.15
SLC6A18	5.53	5.69	5.18
PSORS1C1	5.53	5.73	4.97
LMBR1L	5.53	5.95	5.23
DLX4	5.53	5.76	5.11
LOC100288866	5.53	5.92	5.04
CCNC	5.53	6.12	5.03
AOC3	5.53	6.48	4.62
VSIG8	5.53	5.86	5.20
CTPS1	5.53	5.94	5.18
PPP1R13B	5.53	5.94	5.08
ZNF248	5.53	6.15	5.20
PTPN9	5.53	5.93	5.13
RCBTB1	5.53	5.95	5.19
THRB	5.53	6.07	4.92
FMO4	5.53	6.36	4.85
LY6G6F	5.53	5.80	5.18
HHIPL1	5.53	6.29	5.04
WDR37	5.53	5.77	5.33
TSPAN13	5.53	6.30	4.51
OTTHUMG00000020883	5.53	6.54	4.96
CYP7B1	5.53	6.04	4.96
GOLGA8H	5.53	6.19	5.13
PHF5A	5.53	5.84	5.16
CHRNA3	5.53	5.86	5.25
LOC100506342	5.53	5.90	4.76
CLSTN3	5.53	5.84	5.15
ZNF320	5.53	6.02	4.92
PPP3CC	5.53	6.17	4.50
TMEM241	5.53	5.79	5.16
GNG13	5.53	6.24	5.02
CCL21	5.53	8.09	4.48
CT47A7	5.53	5.86	5.32
R3HCC1L	5.53	6.06	4.80
LOC100506388	5.53	6.05	4.88
IGLJ2	5.53	6.45	4.92
STAG2	5.53	6.01	4.97
CSNK2A1	5.53	6.25	4.80
ZNF197	5.53	5.83	5.23

PTGR2	5.53	5.84	5.18
MFAP3	5.53	5.89	4.61
ANKRD63	5.53	5.90	5.13
CAHM	5.53	5.96	5.24
GSDMB	5.52	5.81	5.19
OTTHUMG00000170970	5.52	5.78	5.11
SERINC5	5.52	6.20	5.14
ZNF329	5.52	5.99	5.01
PNOC	5.52	6.10	5.13
DENND4B	5.52	5.70	5.31
OTTHUMG00000150838	5.52	6.40	4.80
ZNF780A	5.52	5.83	5.11
AQP3	5.52	5.96	5.08
CDCA4	5.52	6.01	5.13
QSER1	5.52	5.95	4.79
TRAPPC4	5.52	6.09	5.06
LY6G6C	5.52	6.15	5.23
UCA1	5.52	6.17	4.90
SUDS3	5.52	5.87	4.98
OTTHUMG00000160084	5.52	5.82	5.21
PEX16	5.52	5.83	5.26
LOC644135	5.52	6.01	4.92
OTTHUMG00000067240	5.52	6.42	4.98
SMC4	5.52	6.24	4.88
LOC375295	5.52	5.80	5.11
ECHDC2	5.52	5.97	5.18
APLN	5.52	5.94	5.19
C19orf24	5.52	5.72	5.35
NDUFAF7	5.52	5.95	5.10
TRAF5	5.52	6.17	5.01
BRSK1	5.52	5.87	5.18
FLJ46875	5.52	5.90	5.17
SEC23B	5.52	5.96	5.06
JUN	5.52	6.69	4.20
IL17RC	5.52	5.81	5.09
NR2C1	5.52	5.97	4.92
NME1-NME2	5.52	5.81	5.23
ENTHD2	5.52	5.73	5.37
ZNF614	5.52	5.80	5.09
LOC100506219	5.52	6.28	5.09
THNSL2	5.52	5.92	5.21
CYYR1	5.52	6.06	4.72
AKT2	5.52	5.76	5.07
GDAP2	5.52	5.99	5.03
ZCCHC5	5.52	5.89	5.04
ENOX1	5.52	5.85	5.06
BRWD3	5.52	6.11	5.02
CASP8AP2	5.52	6.06	4.66
CLIC6	5.52	6.10	5.02

TRIM28	5.52	5.93	4.94
DHRS1	5.52	5.82	4.88
LOC388242	5.52	5.69	5.33
LINC00632	5.52	5.81	5.14
DPY19L3	5.52	6.05	5.07
RNA5SP490	5.52	6.13	5.17
SPRR3	5.52	6.00	5.27
RIPK2	5.52	6.08	4.77
CENPA	5.52	5.67	5.26
INADL	5.52	5.74	5.12
KCTD2	5.52	6.12	5.23
GATSL1	5.52	5.79	5.20
NLGN4Y-AS1	5.52	5.87	4.98
IL10RA	5.52	6.71	4.83
ENO1-IT1	5.52	6.39	4.89
POLR3A	5.52	5.97	5.22
OTTHUMG00000163653	5.52	6.39	5.09
GCOM1	5.52	5.69	5.29
UBE2W	5.52	5.99	5.10
FAM189B	5.52	5.75	5.24
TAB3-AS1	5.52	5.84	4.86
MIB2	5.52	5.81	5.20
MRPL11	5.52	5.83	4.82
PRPF40B	5.52	5.88	5.04
PDE4D	5.52	5.89	5.05
DSC2	5.52	6.25	4.93
SLC25A1	5.52	5.85	5.16
IGSF3	5.52	5.81	5.16
GPR155	5.52	6.75	4.70
SEPP1	5.52	6.16	4.77
IFRD2	5.52	5.82	5.00
SIN3B	5.52	5.71	5.35
KDM5D	5.52	6.66	3.71
CCDC22	5.52	5.66	5.23
SP7	5.52	6.13	5.04
MIR558	5.51	5.92	4.91
ACAT2	5.51	5.87	4.78
ZNF107	5.51	6.29	5.01
SLC16A10	5.51	6.26	4.86
OTTHUMG00000173098	5.51	5.74	5.29
OTTHUMG00000164014	5.51	6.14	4.96
BTBD9-AS1	5.51	5.88	4.88
MIR345	5.51	6.38	4.87
KIAA0556	5.51	5.68	5.32
BTBD19	5.51	5.88	5.17
CCDC144A	5.51	6.71	4.43
MIR3147	5.51	6.56	4.72
MIR1587	5.51	6.18	4.78
YWHAB	5.51	5.86	4.73

UBA5	5.51	5.74	5.12
RUSC1	5.51	5.73	5.11
MTMR12	5.51	6.02	5.11
OTTHUMG00000168308	5.51	5.89	5.20
TIRAP	5.51	5.94	5.22
SNCA	5.51	6.06	4.96
OTTHUMG00000164544	5.51	5.92	5.26
C1orf95	5.51	5.93	5.04
BRD3	5.51	5.80	5.01
ANKFY1	5.51	5.94	4.69
RILPL1	5.51	5.86	5.07
GMFB	5.51	6.17	4.88
FBLN5	5.51	6.57	4.90
RBL1	5.51	5.99	4.89
GNB4	5.51	6.07	4.95
FARP2	5.51	5.77	5.18
RALGAPA2	5.51	5.96	5.18
NAA25	5.51	5.74	5.12
ABHD5	5.51	5.73	5.24
DLG3	5.51	5.95	5.26
TNRC18	5.51	5.74	5.24
PACS2	5.51	5.77	5.23
MUSTN1	5.51	5.84	5.00
SH3D21	5.51	6.03	5.02
OR1D4	5.51	6.43	4.83
SIAE	5.51	5.75	4.97
EFCC1	5.51	6.02	4.89
HIST2H2BA	5.51	6.20	4.78
LOC100507032	5.51	5.97	4.90
ZNF143	5.51	5.80	5.27
SPATA7	5.51	6.05	4.93
POLA1	5.51	5.87	4.98
SNX25	5.51	5.91	4.42
SELM	5.51	5.72	5.21
MEIS3P1	5.51	5.84	5.00
IGSF9B	5.51	5.79	4.91
SUSD2	5.51	5.89	5.21
LINC00619	5.51	6.09	5.13
TNK2	5.51	5.74	5.19
LOC100506392	5.51	6.03	4.73
MEPCE	5.51	5.77	5.13
PPP4R4	5.51	6.17	4.89
DNASE1L2	5.51	5.96	5.15
DCAF8	5.51	5.85	5.22
LINC00083	5.51	5.98	5.03
RNA5SP135	5.51	6.52	4.74
OTTHUMG00000168486	5.51	5.95	5.09
AVEN	5.51	5.98	5.04
MZT2B	5.51	5.88	4.93

FAM193A	5.51	5.85	4.78
LETMD1	5.51	5.84	5.20
RNASET2	5.51	6.15	5.06
RPL31	5.51	5.99	4.94
KBTBD2	5.51	5.90	4.82
RGS14	5.51	5.78	5.11
ZNF660	5.51	6.02	4.86
MAPK7	5.51	5.87	5.09
OTTHUMG00000171718	5.51	6.15	5.07
ATP5J	5.51	6.00	4.68
LY6G6D	5.51	5.90	5.16
SETD8	5.51	5.81	5.11
C1orf132	5.51	5.86	5.16
CELSR2	5.51	5.80	5.26
FTSJ2	5.50	5.84	4.86
PRPH	5.50	6.05	5.24
MRPL34	5.50	5.77	5.19
BPGM	5.50	6.22	5.02
LOC100132174	5.50	6.01	4.89
PEX5	5.50	5.70	5.19
KREMEN1	5.50	6.12	4.76
CMTM4	5.50	6.06	5.11
ATR	5.50	5.90	4.74
TOP2A	5.50	6.72	4.20
POMT2	5.50	5.77	5.24
TRIM68	5.50	5.78	5.20
IFT80	5.50	5.95	5.14
C15orf38	5.50	5.89	5.18
GSTM1	5.50	5.94	5.14
TRIM3	5.50	5.70	5.25
NCEH1	5.50	6.23	5.06
TAB3	5.50	5.99	4.77
ISY1-RAB43	5.50	5.89	5.17
C1QTNF1	5.50	5.98	5.15
RBM15	5.50	5.94	4.88
SPDYC	5.50	5.74	5.10
OTTHUMG00000172599	5.50	5.98	4.98
FAM122B	5.50	5.69	5.30
ATG5	5.50	6.21	4.69
LOC100129697	5.50	5.78	5.21
OR2T27	5.50	6.38	4.92
ADAM15	5.50	5.97	5.21
LOC100128563	5.50	5.90	4.73
AP5Z1	5.50	5.93	5.09
MPDU1	5.50	5.79	4.98
LNP1	5.50	5.80	5.06
MAP1LC3C	5.50	7.51	4.30
SPR	5.50	6.10	4.90
PHF19	5.50	5.79	5.16

PSAT1	5.50	5.95	4.95
ZNF680	5.50	5.98	4.87
LINC00893	5.50	5.95	4.58
CYB561A3	5.50	5.71	5.12
OR2M1P	5.50	6.06	5.07
TRPC7-AS1	5.50	5.79	5.23
MED17	5.50	6.00	4.81
LOC100287166	5.50	5.73	5.16
MIR4803	5.50	6.19	5.04
LDB2	5.50	6.25	4.58
NUPR1L	5.50	6.15	3.57
STAMBPL1	5.50	6.08	4.92
ACSS2	5.50	5.94	5.25
FAM171B	5.50	6.09	4.79
TNKS	5.50	5.83	5.10
TFIP11	5.50	6.01	4.75
UBE2H	5.50	6.02	4.94
FLJ46300	5.50	5.94	5.08
SLC35B2	5.50	6.15	4.97
SMAGP	5.50	6.02	5.22
RCBTB2	5.50	5.86	5.16
TMEM245	5.50	5.82	4.91
NR2C2AP	5.50	5.73	5.14
DACT3	5.50	5.92	5.20
MBD3L3	5.50	6.15	5.15
UNC13B	5.50	5.88	5.31
ZNF100	5.50	6.47	4.62
ATXN1L	5.50	6.06	4.72
DNTTIP2	5.50	6.05	4.78
PRAMEF16	5.50	6.82	4.78
SNHG17	5.50	6.31	5.10
AQP5	5.50	5.81	5.04
EMG1	5.50	5.87	4.90
ANP32A-IT1	5.50	6.05	4.99
INTS6	5.50	5.77	5.18
HSP90AA1	5.50	5.96	4.94
MGST2	5.50	6.14	5.08
UBFD1	5.50	5.99	5.21
MOK	5.50	5.87	5.07
DNMBP	5.50	5.82	5.12
LCAT	5.50	5.94	4.98
KRTAP12-3	5.50	5.91	5.19
ZNF311	5.50	5.87	5.18
HOOK2	5.50	5.88	5.31
NAGS	5.50	5.77	5.22
ANXA8	5.50	5.98	5.29
EPB41L4A-AS1	5.50	5.80	5.02
PNKP	5.50	5.98	5.20
LOC392196	5.50	5.93	5.13

CHFR	5.50	5.96	5.00
LRRC32	5.50	7.04	4.51
TEX261	5.50	6.11	4.49
DCP1A	5.50	5.75	4.87
ELOF1	5.50	6.19	4.74
OTTHUMG00000161870	5.50	6.10	5.26
C17orf61-PLSCR3	5.49	5.78	5.06
GAREM	5.49	6.03	5.09
FGF10	5.49	6.68	3.88
UBOX5-AS1	5.49	5.81	5.10
PPP1R12C	5.49	5.72	5.26
LPAR6	5.49	6.06	4.82
MGAT1	5.49	5.86	5.00
TADA3	5.49	5.87	4.93
SMIM10	5.49	5.87	5.06
ZGLP1	5.49	5.85	5.04
MIR1296	5.49	5.87	5.20
AP2A1	5.49	5.77	5.20
RPP14	5.49	5.88	5.13
SNX8	5.49	5.67	5.19
KCNN4	5.49	5.70	5.06
KLF11	5.49	5.90	4.81
MUTYH	5.49	5.85	5.11
GZMM	5.49	5.96	5.16
CDKAL1	5.49	5.95	4.93
OTTHUMG00000176557	5.49	5.80	5.16
ATP6V1A	5.49	6.23	4.76
NMI	5.49	6.52	4.37
LINC00921	5.49	5.80	4.99
TACO1	5.49	5.91	4.77
MAR5	5.49	5.95	4.87
OTTHUMG00000018061	5.49	5.77	5.05
PCNXL3	5.49	5.80	4.81
LCN2	5.49	6.12	5.16
SLC25A34	5.49	5.84	5.28
OTTHUMG00000016040	5.49	5.81	4.87
PRKCZ	5.49	5.88	5.10
OTTHUMG00000163517	5.49	6.09	5.03
TSKU	5.49	6.14	4.75
OVOL3	5.49	5.87	5.11
ZNF513	5.49	5.92	5.26
IFI27L2	5.49	6.03	5.20
SEMA5B	5.49	5.94	5.13
HSD3B7	5.49	5.83	5.06
RANBP3L	5.49	6.27	4.61
PSMF1	5.49	5.89	5.03
SNORD88C	5.49	5.84	5.10
LTC4S	5.49	5.71	5.12
POU5F1	5.49	5.86	5.21

PKN3	5.49	6.10	4.93
GDI1	5.49	5.91	5.21
FCN3	5.49	5.98	4.95
ATG12	5.49	5.92	5.08
ZFYVE21	5.49	5.69	5.07
NEDD1	5.49	6.01	4.63
CROCC	5.49	5.79	5.03
FLI1-AS1	5.49	5.97	5.20
CCDC74B	5.49	6.32	5.09
LRRC56	5.49	5.68	5.29
CAMTA1	5.49	5.80	5.15
SORD	5.49	5.98	4.90
DPH1	5.49	5.77	5.13
OTTHUMG00000152619	5.49	5.93	5.18
SCN4B	5.49	5.77	5.00
LRIF1	5.49	5.83	4.86
KIF4A	5.49	5.91	5.15
USP42	5.49	5.84	5.06
DCAF15	5.49	5.88	5.07
GZMH	5.49	6.42	5.15
OR5E1P	5.49	6.24	4.54
L3MBTL2	5.48	5.82	5.13
FBXO25	5.48	6.01	5.10
SRA1	5.48	5.89	4.75
BRI3BP	5.48	5.90	4.78
LOC100287628	5.48	6.16	4.79
TBC1D12	5.48	5.74	4.95
LOC100131067	5.48	5.72	5.15
ADCY2	5.48	6.25	4.78
TMLHE-AS1	5.48	6.11	5.16
FAM96A	5.48	6.08	4.75
NSFP1	5.48	6.28	4.41
FAM138B	5.48	6.15	4.81
STX5	5.48	5.83	5.17
CD5	5.48	5.97	5.03
GRIPAP1	5.48	5.79	5.14
ZDHHC17	5.48	5.88	5.11
PABPC1	5.48	5.96	5.01
LOC613038	5.48	5.68	5.17
CRTC3	5.48	5.79	5.15
GALNT10	5.48	5.85	5.09
CREB3L1	5.48	6.08	5.04
STRADA	5.48	5.95	5.07
CIR1	5.48	5.82	4.95
MYNN	5.48	6.06	4.94
SELV	5.48	5.91	4.96
C16orf45	5.48	5.66	5.26
MGC57346	5.48	5.76	5.22
STK24	5.48	5.91	5.20

PLEKHJ1	5.48	5.74	5.23
ANGPT4	5.48	5.96	5.10
SLC16A2	5.48	6.04	5.04
HIST2H2BF	5.48	5.94	5.01
HGS	5.48	5.86	5.30
FLT1	5.48	6.00	4.75
HES1	5.48	5.85	5.06
OTTHUMG00000032141	5.48	6.43	4.77
HLA-DRB6	5.48	6.08	5.20
MIR211	5.48	5.81	5.10
FAM209B	5.48	5.95	5.09
C5AR2	5.48	5.92	4.90
DFNA5	5.48	5.84	5.19
NRF1	5.48	5.85	5.15
NSUN5	5.48	5.76	5.00
MIR596	5.48	5.94	4.94
DBN1	5.48	6.07	5.06
SAYSD1	5.48	6.03	5.09
CUL9	5.48	5.65	5.23
OBFC1	5.48	5.87	4.82
ZKSCAN3	5.48	5.69	5.20
BATF3	5.48	5.77	5.19
FHOD3	5.48	6.10	5.12
PDGFD	5.48	6.28	4.59
MRGPRD	5.48	5.96	5.17
MIR1247	5.48	5.99	5.09
FAM57B	5.48	5.97	5.03
MAPK8IP3	5.48	5.99	5.05
MIR548AN	5.48	6.30	4.68
AAR2	5.48	5.71	4.92
WDR86-AS1	5.48	5.95	5.00
GATAD2A	5.48	5.67	5.19
CNOT10	5.48	6.04	5.17
GUSBP1	5.48	6.04	4.97
TMEM74B	5.48	5.76	4.80
TTC23	5.48	5.99	5.18
MIR221	5.48	6.54	4.79
TUBGCP4	5.48	6.05	4.97
PPIE	5.48	5.94	4.66
OTTHUMG00000151363	5.48	6.21	4.99
PRKCD	5.48	5.98	5.15
RAB4A	5.48	5.94	4.82
RSG1	5.48	5.91	5.15
ARHGAP11B	5.48	5.85	4.98
DTYMK	5.48	5.99	4.99
OTTHUMG00000172590	5.48	6.45	4.23
RFTN1	5.48	5.74	4.91
TMEM176A	5.47	6.01	5.17
MKNK1	5.47	6.12	5.01

ABHD8	5.47	5.73	5.20
KIAA0226L	5.47	6.56	4.57
LOC100506801	5.47	5.77	5.13
ARHGAP29	5.47	5.98	5.09
OTTHUMG00000016875	5.47	6.63	4.61
CCNT2	5.47	5.88	4.67
LOC642929	5.47	6.65	5.07
EZH2	5.47	5.92	4.82
ADM5	5.47	5.76	5.01
OTTHUMG00000169290	5.47	5.80	4.87
PPP2R5D	5.47	6.00	4.94
C17orf99	5.47	5.90	4.89
SUGP1	5.47	5.68	5.05
NLE1	5.47	5.88	5.10
DDT	5.47	5.69	5.23
ARHGEF17	5.47	5.84	5.10
TMEM11	5.47	5.91	4.85
SYNE1-AS1	5.47	5.71	5.15
WDR74	5.47	5.80	4.90
MUC12	5.47	5.77	5.05
ZNF445	5.47	5.69	5.18
C14orf159	5.47	5.86	5.03
OTTHUMG00000166666	5.47	5.78	4.81
GAST	5.47	6.05	5.17
GAS6-AS1	5.47	5.71	5.28
P2RX1	5.47	5.83	4.83
SLC7A5	5.47	6.71	4.90
OR2L8	5.47	6.79	4.26
TRMT6	5.47	5.81	4.88
ANKS3	5.47	5.66	5.21
POP4	5.47	5.80	4.86
PTGES	5.47	7.09	4.43
COL5A3	5.47	5.77	5.08
DUSP6	5.47	5.87	4.94
NUDCD2	5.47	6.03	4.85
SLC26A4	5.47	6.05	5.04
ARX	5.47	5.64	5.21
PNPLA4	5.47	5.89	5.21
LOC100132891	5.47	5.93	5.07
ARL15	5.47	5.98	4.79
OTTHUMG00000163994	5.47	5.77	5.05
INF2	5.47	5.62	5.25
GPKOW	5.47	5.75	5.03
TRAP1	5.47	5.80	5.04
TMEM55B	5.47	5.83	5.27
STX1A	5.47	5.85	5.00
IMPAD1	5.47	6.18	4.53
FABP4	5.47	7.78	2.69
VWA1	5.47	5.71	5.22

TRAPPC12-AS1	5.47	5.84	4.89
GJA5	5.47	5.87	4.78
MIR1468	5.47	6.30	4.99
CEACAM19	5.47	5.85	4.99
HSPB11	5.47	6.11	4.35
TUBGCP3	5.47	5.93	4.90
RAB26	5.47	5.72	5.17
TMEM180	5.47	5.68	5.28
MKL1	5.47	5.78	5.19
MXI1	5.47	5.92	5.22
BANP	5.47	5.81	4.94
COG8	5.47	5.85	4.85
PPM1L	5.47	5.78	5.15
SGSH	5.47	5.66	5.05
RNA5SP420	5.47	6.12	5.08
ARID3A	5.47	5.83	5.06
TDP1	5.47	5.73	5.25
ANKZF1	5.47	5.88	5.09
MRPL30	5.47	5.80	4.85
CLASP2	5.47	5.81	5.01
CRNN	5.47	6.14	4.93
ZNF317	5.47	5.82	5.12
MTRNR2L6	5.47	6.05	5.09
RPP30	5.47	5.88	4.98
CEP104	5.47	6.03	4.99
ACSM5	5.47	5.80	4.99
PTRH2	5.47	5.95	4.93
C21orf91	5.47	5.86	5.16
MGC40069	5.47	6.01	4.65
PIGQ	5.47	5.70	5.27
KIAA1456	5.47	5.87	5.05
GALNT11	5.47	5.78	4.86
LEPREL2	5.47	5.92	5.12
NCOA6	5.47	5.76	5.06
OR2M5	5.47	6.19	4.93
ZDHHC14	5.47	5.79	5.26
PAQR4	5.47	6.01	5.01
OSGIN1	5.47	5.85	4.99
ITIH5	5.47	5.75	4.77
UTP20	5.47	5.84	5.12
RNA5SP191	5.47	6.51	4.00
GATSL3	5.46	5.80	5.11
NECAP1	5.46	5.69	5.11
MRGPRX1	5.46	5.81	5.02
URM1	5.46	5.92	5.19
CCL19	5.46	6.42	4.84
BOD1	5.46	5.79	4.84
BTBD10	5.46	5.87	5.05
NDUFV1	5.46	5.71	5.11

TMEM25	5.46	5.79	5.16
OR11H1	5.46	7.02	4.38
PKP4	5.46	5.76	5.12
MEX3A	5.46	5.73	5.04
KATNBL1	5.46	5.65	5.02
HIST1H2AE	5.46	7.03	4.29
METTL25	5.46	5.91	4.86
ZNF705E	5.46	6.15	4.86
ZFHX4	5.46	6.09	5.09
NAAA	5.46	5.86	5.13
METTL8	5.46	5.71	5.10
VAC14	5.46	5.66	5.05
DNAJC25	5.46	5.86	5.04
ZBTB24	5.46	5.68	4.86
CEBPB	5.46	5.74	5.27
LGALS3BP	5.46	5.96	4.72
GUF1	5.46	5.88	4.98
MAFA	5.46	5.87	4.97
LNX1-AS1	5.46	6.02	4.99
SNORD121B	5.46	6.04	5.18
NIPSNAP1	5.46	5.63	5.19
LOC400590	5.46	6.02	4.88
OTTHUMG00000018077	5.46	5.85	5.01
BTG3	5.46	5.90	4.85
KCP	5.46	5.82	5.05
MIR106B	5.46	5.65	5.12
CDRT15	5.46	5.95	4.84
THAP7	5.46	5.76	4.98
LOC100129476	5.46	5.76	5.24
TJAP1	5.46	5.71	5.25
LOC442132	5.46	6.39	4.48
OTTHUMG00000002201	5.46	6.08	4.93
WDR18	5.46	5.76	5.07
CAPN3	5.46	6.24	4.33
SNORA14A	5.46	7.80	3.13
FLJ41200	5.46	5.86	5.11
OTTHUMG00000161608	5.46	6.11	4.47
DCTPP1	5.46	5.89	5.09
SV2B	5.46	5.98	4.82
PAK1IP1	5.46	6.15	4.34
SGK494	5.46	5.66	5.31
AGPAT4-IT1	5.46	5.82	4.99
TMEM98	5.46	5.73	4.77
OTTHUMG00000168255	5.46	5.82	5.15
KRTAP10-3	5.46	5.95	4.66
ZBTB38	5.46	6.12	4.86
NIPA2	5.46	5.74	5.26
ZC3H3	5.46	5.73	5.08
INSR	5.46	5.87	4.98

MCOLN2	5.46	6.16	4.79
OTTHUMG00000014663	5.46	5.70	5.23
IGHV4-59	5.46	6.19	4.88
CXorf61	5.46	6.23	4.88
TMEM104	5.46	6.04	4.83
CGB1	5.46	6.12	4.60
EP400NL	5.46	5.88	5.01
GRINA	5.46	6.07	5.07
DCUN1D3	5.46	5.85	5.16
FBLN7	5.46	5.98	4.74
SEC16B	5.46	5.64	5.17
MBD6	5.46	5.70	5.25
PPP2R5A	5.46	5.67	5.06
IL9R	5.46	5.99	4.97
GLCE	5.46	5.95	4.68
GGA2	5.46	5.78	4.81
CNTRL	5.46	5.74	5.03
ZC2HC1A	5.46	6.11	4.70
ZNF142	5.46	5.82	5.01
PRC1-AS1	5.46	6.24	4.92
PLOD3	5.46	5.98	5.02
LOC100505736	5.46	6.03	5.06
TRIM40	5.46	5.80	5.03
GIPC1	5.45	5.97	5.04
KIF27	5.45	5.75	5.17
CDK16	5.45	5.97	4.98
MALSU1	5.45	5.76	5.16
UNG	5.45	5.84	5.07
ARMS2	5.45	5.91	4.95
ELMO1-AS1	5.45	6.05	4.79
ZNF791	5.45	5.84	5.09
RGL4	5.45	5.81	5.02
LOC100130700	5.45	6.00	5.15
PIGC	5.45	5.90	4.63
CHCHD5	5.45	5.61	5.24
NFKBIB	5.45	5.62	5.19
KCNQ1DN	5.45	5.76	5.21
WIPF2	5.45	6.04	4.95
BACE2	5.45	6.08	4.87
LOC100996485	5.45	5.95	4.80
SORBS2	5.45	6.04	4.56
SORBS3	5.45	5.75	5.03
TSEN54	5.45	5.73	5.22
LOC100287036	5.45	5.84	4.87
LOC728763	5.45	5.77	5.10
FRK	5.45	6.17	4.91
LOC100133445	5.45	6.06	4.68
NUTM2A-AS1	5.45	5.99	5.06
NGLY1	5.45	5.85	4.59

H2AFX	5.45	6.08	4.90
USP5	5.45	5.93	4.84
GPR18	5.45	5.74	5.23
TNFRSF13B	5.45	5.77	4.69
ZNF3	5.45	5.93	4.95
VAMP4	5.45	5.86	4.90
C1QTNF5	5.45	5.67	5.24
LINC00665	5.45	5.80	5.03
POLK	5.45	6.03	4.94
SMURF1	5.45	5.82	4.96
AKT1	5.45	5.92	5.25
SNX12	5.45	5.95	4.33
MMP16	5.45	6.10	4.40
GSTO1	5.45	5.98	4.97
LOC100506544	5.45	5.75	5.26
COL26A1	5.45	5.66	5.33
OTOP2	5.45	6.01	5.10
TFRC	5.45	6.18	4.51
ARHGAP5-AS1	5.45	5.79	5.20
OTTHUMG00000158558	5.45	5.86	5.12
D2HGDH	5.45	5.78	5.02
ZNF580	5.45	5.86	5.07
FDFT1	5.45	5.70	5.19
KCTD18	5.45	5.91	4.87
NOG	5.45	5.76	4.75
CCDC142	5.45	5.64	5.17
MIRLET7C	5.45	6.10	3.89
CACNA1I	5.45	5.83	5.01
KLHL7	5.45	5.86	4.98
SHC3	5.45	5.79	4.95
SLC7A10	5.45	5.94	5.04
UBIAD1	5.45	5.80	5.08
RAPGEF1	5.45	5.81	4.53
NCDN	5.45	5.63	5.16
MPP1	5.45	6.15	5.07
FAM133CP	5.45	5.87	4.84
PHF23	5.45	5.87	4.91
CGREF1	5.45	5.80	4.98
TMUB1	5.45	5.65	5.14
WDR78	5.45	5.92	4.89
MECP2	5.45	5.74	4.73
RBMS3-AS2	5.45	5.73	4.95
VCX3A	5.45	5.72	5.07
FOXN4	5.45	5.72	5.18
SERPINA6	5.45	6.32	4.42
HIST2H2BE	5.45	6.10	4.86
CT47A10	5.45	5.78	5.13
LCP2	5.45	6.23	4.75
KLHDC3	5.45	5.87	5.12

RNA5SP322	5.45	6.15	4.81
CAMKMT	5.45	5.82	5.03
CD7	5.45	5.87	5.14
OR51B4	5.45	6.07	5.17
LY6H	5.45	5.87	5.02
DSP	5.45	5.67	4.93
C4orf27	5.45	6.05	4.87
LEMD3	5.45	5.97	4.78
XCR1	5.45	5.92	4.94
RRP1	5.44	5.74	5.08
MBNL1-AS1	5.44	6.03	4.99
EIF4E3	5.44	5.92	5.15
PEX11G	5.44	5.77	5.09
OTTHUMG00000164478	5.44	5.80	5.08
C11orf68	5.44	5.69	5.15
TRIP10	5.44	6.01	5.08
FAM66C	5.44	5.80	4.92
ZBTB46	5.44	6.00	4.92
ZNF518A	5.44	5.92	4.93
RFXANK	5.44	5.67	5.08
SEMA4G	5.44	5.81	5.14
SNRPA1	5.44	5.71	4.85
PSPH	5.44	5.59	5.27
FLJ33630	5.44	6.05	4.93
CHRM4	5.44	5.71	5.26
GPSM3	5.44	5.85	5.01
LINC00701	5.44	5.79	4.93
OTTHUMG00000012337	5.44	5.74	5.01
MROH8	5.44	5.80	4.96
DPF1	5.44	5.78	5.19
CUEDC2	5.44	5.89	5.13
SLC9A1	5.44	5.85	4.98
LOC100506191	5.44	5.73	5.07
TSC22D1-AS1	5.44	5.71	5.17
GALNT7	5.44	6.48	4.51
CABLES1	5.44	5.87	5.01
GSX2	5.44	5.65	5.07
ZNF512B	5.44	5.84	5.11
QRSL1	5.44	5.77	5.07
BOLA1	5.44	5.83	5.17
IMPDH1	5.44	5.79	4.94
GATA5	5.44	5.89	4.84
PRAMEF24P	5.44	5.79	4.82
VRK3	5.44	5.77	5.15
OTTHUMG00000172506	5.44	5.88	5.05
NAGA	5.44	5.91	4.60
DEF8	5.44	5.67	5.22
VLDLR	5.44	6.47	4.54
NPW	5.44	5.71	4.98

RPL13P5	5.44	5.77	4.89
SHANK2	5.44	5.77	4.97
C3orf58	5.44	5.67	4.59
HSBP1L1	5.44	5.98	5.03
TPMT	5.44	6.30	4.79
CT47A1	5.44	5.76	5.17
CAB39L	5.44	5.96	4.85
ARHGAP4	5.44	5.64	5.28
BUD13	5.44	5.77	5.07
GPR179	5.44	5.78	4.80
DAAM1	5.44	6.02	5.09
ESAM	5.44	6.03	4.63
GBGT1	5.44	5.68	5.23
PLCD1	5.44	5.75	4.99
PLXNA2	5.44	6.26	4.55
NDUFS8	5.44	5.79	5.10
TNNC1	5.44	6.24	5.08
SYNGR1	5.44	5.59	5.11
NDFIP2	5.44	5.98	4.54
TMEM97	5.44	5.79	5.16
MMP11	5.44	5.73	5.18
HMGB1	5.44	5.79	5.09
MYOC	5.44	6.88	4.22
NAT6	5.44	5.72	5.28
PWWP2A	5.44	5.62	5.25
STK3	5.44	5.90	5.14
C10orf12	5.44	5.89	4.99
AGAP11	5.44	6.27	4.89
GGT1	5.44	5.61	5.21
CSRNP2	5.44	5.59	5.17
XRCC1	5.44	5.78	5.09
NDST1	5.44	5.81	5.10
PROSER2	5.44	5.74	5.16
TSPYL1	5.44	5.84	5.06
NTHL1	5.44	5.82	5.08
PDCD2	5.44	5.93	4.94
CAPS	5.44	5.83	4.94
ZNF605	5.44	5.87	5.02
OTTHUMG00000041227	5.44	6.18	4.91
NPL	5.44	6.38	4.83
ZNF76	5.44	5.62	5.13
WIPI2	5.44	5.64	4.71
MCM3AP-AS1	5.44	5.68	5.02
CCDC57	5.44	5.86	4.98
HIRA	5.44	5.78	5.05
CD79A	5.44	5.87	5.18
GUCD1	5.44	5.85	5.09
LINC00337	5.44	5.59	5.25
MRTO4	5.44	6.20	5.11

DDX10	5.44	5.63	5.00
ADD3-AS1	5.43	5.76	5.18
ZNF528	5.43	5.62	4.86
CTPS2	5.43	5.68	4.91
INO80	5.43	5.74	5.14
PPM1B	5.43	5.88	4.63
SIGIRR	5.43	5.90	4.98
AVIL	5.43	5.74	5.04
PIP4K2A	5.43	5.90	5.02
RPL34	5.43	5.58	5.09
GSPT1	5.43	5.91	4.47
TBX10	5.43	5.81	5.08
TWIST1	5.43	6.18	4.50
MAN2C1	5.43	5.78	5.13
PHKA2	5.43	5.88	4.75
RNF130	5.43	5.78	5.06
LOC100129427	5.43	5.75	5.19
PRKAA2	5.43	5.76	5.12
DMBX1	5.43	5.58	5.13
ZNF90	5.43	5.76	5.03
THAP4	5.43	5.78	5.08
RBM12B-AS2	5.43	5.90	4.94
DBP	5.43	6.12	4.88
FOXA2	5.43	5.87	5.09
APOM	5.43	5.71	5.14
CCDC74B-AS1	5.43	5.89	5.19
TRIM72	5.43	5.73	5.14
CSPG4P1Y	5.43	5.64	5.22
TLX2	5.43	6.02	5.09
SNORA6	5.43	5.78	4.88
DNAJC16	5.43	5.78	4.61
LOC100507156	5.43	6.37	4.44
DND1	5.43	5.74	4.99
ZNF500	5.43	6.09	4.87
PPP1R15A	5.43	5.92	4.92
SNHG6	5.43	5.68	5.09
OTTHUMG00000169635	5.43	5.69	4.99
CD52	5.43	6.20	4.77
MYH10	5.43	6.17	4.70
SRPK1	5.43	5.94	4.88
PNMA6D	5.43	5.78	5.19
TBC1D14	5.43	5.68	5.00
ZBTB6	5.43	5.92	4.91
SHPK	5.43	5.70	5.23
MLH3	5.43	5.68	4.61
CHMP6	5.43	5.74	5.00
AGAP3	5.43	5.64	5.09
LPPR2	5.43	5.62	5.22
STAT5A	5.43	5.79	5.08

OTTHUMG00000156019	5.43	5.77	5.03
TRAPPC8	5.43	6.03	4.57
CBFA2T2	5.43	5.85	5.15
DLC1	5.43	5.96	4.55
FAM117B	5.43	5.68	5.18
GRM4	5.43	5.83	5.17
BRWD1-IT2	5.43	5.80	5.06
TSFM	5.43	6.05	5.04
HEATR8-TTC4	5.43	5.52	5.28
SSTR2	5.43	6.22	4.46
LINC00672	5.43	5.90	5.14
HACE1	5.43	6.04	4.88
MIR744	5.43	5.75	5.08
POLR3GL	5.43	5.77	5.26
MTUS1	5.43	5.87	4.45
THOC5	5.43	5.82	4.85
SPAG5-AS1	5.43	5.64	5.05
C3orf37	5.43	5.80	4.59
DERA	5.43	5.84	4.78
IKBKB	5.43	5.65	4.88
ISCA1	5.43	5.83	4.98
ZNF484	5.43	5.79	4.94
KCTD5	5.42	5.72	5.14
GAS8	5.42	5.65	5.02
TCHP	5.42	5.89	4.99
SLC35E2	5.42	5.87	4.89
EBP	5.42	5.72	4.83
PNMA3	5.42	6.03	4.76
SPRYD4	5.42	5.83	5.23
HARS2	5.42	5.65	5.13
PLA2G4A	5.42	6.05	4.46
BRCC3	5.42	6.00	4.85
NEK11	5.42	5.74	4.88
ZNF814	5.42	5.98	4.70
CD300LG	5.42	5.86	5.07
SAMD8	5.42	5.88	4.70
S100A3	5.42	5.95	4.88
NCCRP1	5.42	5.96	4.96
OTTHUMG00000176447	5.42	5.97	4.86
CCT4	5.42	6.11	4.71
PSMB8	5.42	5.92	5.16
RRP9	5.42	5.81	5.06
C2orf76	5.42	5.75	4.93
MIRLET7A3	5.42	5.74	5.20
ANKRD20A19P	5.42	5.73	4.69
FADS6	5.42	5.93	4.85
TRMU	5.42	5.72	4.91
G2E3	5.42	5.91	5.02
BEX5	5.42	6.09	4.68

SNORA68	5.42	5.97	4.62
ARNTL2	5.42	6.48	4.59
C1orf35	5.42	5.72	5.16
PRMT5	5.42	5.86	4.98
TMEM223	5.42	5.78	5.20
GIMAP5	5.42	6.05	4.73
SZT2	5.42	5.62	5.21
CASQ1	5.42	5.79	4.96
COX5B	5.42	5.68	5.05
ZNF331	5.42	5.94	5.01
SAC3D1	5.42	5.63	5.20
MRFAP1L1	5.42	5.65	5.18
RNU6ATAC5P	5.42	6.18	4.59
SPATA21	5.42	5.88	5.06
C1orf192	5.42	6.09	4.95
ZDHHC1	5.42	5.72	5.14
CLDN9	5.42	5.81	4.91
MIR3190	5.42	6.10	4.68
ZBTB20-AS2	5.42	6.10	3.43
NUP88	5.42	5.82	4.98
CDRT15P1	5.42	6.15	4.61
NKX1-1	5.42	5.99	5.17
QDPR	5.42	5.82	5.13
KCTD21	5.42	5.91	5.13
WDR46	5.42	5.80	4.73
PHRF1	5.42	5.69	5.08
PGP	5.42	5.80	4.89
TANK	5.42	5.89	4.92
GALR2	5.42	6.13	5.00
GART	5.42	5.75	4.95
ELMSAN1	5.42	5.85	5.12
SNF8	5.42	6.01	4.75
HCN4	5.42	6.09	5.03
DYRK2	5.42	5.75	5.12
OR52R1	5.42	5.89	4.71
HDAC6	5.42	5.73	5.08
NRK	5.42	6.21	4.66
RNMTL1	5.42	5.73	5.03
C14orf132	5.42	5.86	4.93
GAK	5.42	5.57	5.18
ZRSR2	5.42	5.90	4.81
OTTHUMG00000166314	5.42	5.95	4.90
CACTIN	5.42	5.69	5.06
SRGAP1	5.42	6.19	4.36
ZDHHC8	5.42	5.80	4.64
PKP3	5.42	5.75	5.01
MAR2	5.42	5.91	5.10
TCOF1	5.42	5.73	5.19
NDUFB5	5.41	5.77	5.07

SLC6A20	5.41	5.77	5.02
OARD1	5.41	5.57	5.11
PTER	5.41	5.68	5.15
DANCR	5.41	5.82	5.02
SKP2	5.41	5.82	4.98
SSX8	5.41	6.02	4.47
WDR92	5.41	5.70	5.11
LOC730101	5.41	5.93	4.88
C14orf80	5.41	5.68	5.15
BHLHA15	5.41	6.06	4.82
LOC100289061	5.41	5.83	5.06
ZNF335	5.41	5.60	5.12
C16orf95	5.41	5.82	5.14
DRD5	5.41	5.82	5.18
AR	5.41	5.90	4.87
WNT11	5.41	5.95	5.16
VPS13A-AS1	5.41	5.62	5.12
TRAJ10	5.41	6.15	4.78
GEMIN5	5.41	5.65	5.05
RNA5SP133	5.41	5.70	5.12
TMBIM6	5.41	5.85	4.84
USP32	5.41	6.15	4.51
ZMYM5	5.41	5.82	5.07
PRSS1	5.41	6.28	4.70
ZNF684	5.41	5.69	5.06
DCP1B	5.41	5.69	4.48
PRUNE	5.41	5.83	4.89
ECI2	5.41	5.90	4.83
FBXO32	5.41	5.96	4.91
PKD1	5.41	5.92	4.95
ZNF135	5.41	5.88	5.06
ZNF213	5.41	5.60	5.16
RNA5SP251	5.41	6.09	4.95
TDRP	5.41	5.92	4.95
LOC654433	5.41	5.89	4.51
NUP50	5.41	5.79	5.05
MOAP1	5.41	5.73	4.75
DYNC1LI1	5.41	5.80	4.54
PRR4	5.41	5.94	4.96
FAM102A	5.41	6.03	4.76
SFMBT1	5.41	5.69	4.96
SNORD117	5.41	6.09	4.61
CTAG1A	5.41	5.93	5.02
GAS7	5.41	6.29	4.80
DNAJC11	5.41	5.67	5.03
PRRT3	5.41	6.04	4.87
TREM2	5.41	6.46	4.48
FAM172A	5.41	5.85	4.88
URB1	5.41	5.89	5.12

PPAPDC2	5.41	5.74	4.94
GPR137	5.41	5.76	4.94
ERCC2	5.41	5.71	4.80
NUPL1	5.41	6.03	4.80
ZNF385C	5.41	5.94	4.97
HDHD3	5.41	5.74	5.14
BRF1	5.41	5.77	5.09
LINC00278	5.41	6.29	4.70
ARHGEF25	5.41	5.68	4.98
ZBTB40-IT1	5.41	5.83	4.92
FABP5	5.41	5.72	5.26
HECTD3	5.41	5.66	5.11
RAB30	5.41	5.69	5.06
DERL3	5.41	5.85	5.02
CDC23	5.41	5.85	5.04
C10orf54	5.41	5.67	5.15
PRB2	5.41	5.93	4.65
MED15	5.41	5.90	5.09
PITHD1	5.41	6.14	4.53
CD2BP2	5.41	5.82	4.97
NSUN5P1	5.40	5.92	4.80
UBE2E2	5.40	5.81	4.72
PAAF1	5.40	5.61	5.10
RAB11FIP4	5.40	5.91	5.01
FAM207A	5.40	5.85	5.13
LOC728724	5.40	5.61	5.18
FOXP1-IT1	5.40	6.35	4.89
OTUD7B	5.40	5.65	5.11
TCEA2	5.40	5.90	4.95
TNFRSF18	5.40	5.72	4.98
LGALS9	5.40	5.78	5.07
TTTY6	5.40	5.95	4.61
TMCC1	5.40	5.61	4.98
SLC6A9	5.40	5.60	5.14
MIR539	5.40	5.63	4.86
MIR4289	5.40	5.97	4.77
SLC25A35	5.40	5.89	5.04
MIR3130-1	5.40	5.82	5.03
ZNF598	5.40	5.82	5.16
C10orf32-AS3MT	5.40	5.70	4.98
FRMD4A	5.40	5.82	4.97
YY1AP1	5.40	5.90	4.85
MIR642A	5.40	5.98	4.84
MGMT	5.40	5.64	5.04
MAP3K2	5.40	5.97	4.60
CXorf40B	5.40	5.63	4.78
ZNF561	5.40	5.77	4.88
LRRC58	5.40	5.86	4.57
TMEM9	5.40	5.76	4.85

MSS51	5.40	5.79	5.14
KCNK1	5.40	5.88	5.17
ARHGEF40	5.40	5.85	4.99
ZNF581	5.40	5.59	5.03
PYROXD2	5.40	5.67	5.19
PTGES3L-AARSD1	5.40	5.58	5.16
EPS8L2	5.40	5.66	5.03
LOC283214	5.40	5.76	4.93
UBASH3B	5.40	5.76	4.85
PRSS56	5.40	5.62	4.96
ZNF48	5.40	5.69	4.82
RAB5A	5.40	6.06	4.73
FLJ42393	5.40	5.62	4.95
SLC4A2	5.40	6.00	4.54
FADS3	5.40	5.57	4.97
AKAP1	5.40	5.64	5.17
KIAA0196-AS1	5.40	5.66	5.10
AIFM1	5.40	5.86	4.57
RMND5A	5.40	5.81	4.89
OPA3	5.40	5.74	4.90
C15orf41	5.40	5.84	4.92
KIAA1217	5.40	5.85	4.95
C9orf85	5.40	5.81	4.91
RAB11FIP5	5.40	5.51	5.16
IFNL3	5.40	6.06	4.94
TSHZ3	5.40	6.38	4.48
LOC100506183	5.40	5.69	5.12
ENHO	5.40	5.89	5.18
MRPL23-AS1	5.40	5.83	4.94
JPH4	5.40	6.05	5.07
HOXD3	5.40	5.78	5.02
ILDR1	5.40	5.96	4.96
SUV420H2	5.40	5.75	5.12
METTL6	5.40	6.09	4.68
MEIS3	5.40	5.71	4.95
PPAN-P2RY11	5.40	5.76	5.07
C16orf74	5.40	5.65	5.11
FAM179B	5.40	5.60	5.11
CDA	5.40	6.05	4.52
PODXL2	5.40	5.81	4.85
AMPD2	5.40	5.66	4.94
FYN	5.40	5.99	4.98
MAP3K3	5.40	5.83	4.89
CENPL	5.40	5.74	4.96
LOC100507424	5.40	5.71	4.84
GLIS1	5.40	5.70	5.06
FAM50B	5.40	5.70	5.12
ATOX1	5.40	5.55	5.05
HIST1H2BL	5.40	5.96	5.13

CABLES2	5.40	5.81	5.06
SNORD113-9	5.40	6.90	4.23
RNASEH1	5.39	5.57	5.02
PODN	5.39	6.15	4.74
C2orf47	5.39	5.73	4.64
DGKH	5.39	5.71	4.90
GLIS2	5.39	5.67	4.79
FMNL1	5.39	5.97	4.92
TMEM251	5.39	5.82	4.63
FZD6	5.39	5.96	4.74
MIR134	5.39	6.06	4.76
LYPD5	5.39	5.68	5.05
NIM1	5.39	5.81	4.68
YBEY	5.39	5.83	4.60
CYTH1	5.39	5.69	5.09
OTTHUMG00000163609	5.39	6.00	4.91
ID3	5.39	5.91	4.80
MAGI3	5.39	5.71	4.85
SNCAIP	5.39	5.73	4.77
RAB36	5.39	5.58	5.22
ABCA6	5.39	6.26	3.95
ID2	5.39	5.72	4.61
L3HYPDH	5.39	5.80	4.79
GPR124	5.39	6.09	5.08
ZNF862	5.39	5.66	5.07
CLDN2	5.39	6.07	4.80
DDX11L10	5.39	6.17	4.79
SNORD92	5.39	5.67	4.87
LOC100129785	5.39	5.76	4.73
TEF	5.39	5.80	4.97
ADORA2A-AS1	5.39	5.69	4.99
UBL4B	5.39	5.94	4.80
IL17B	5.39	5.84	4.85
SNORD103A	5.39	5.79	4.72
METAP1	5.39	5.83	4.97
LINC00273	5.39	6.02	4.78
RFX1	5.39	5.82	5.15
TIAM1	5.39	5.83	4.96
ZNF507	5.39	5.96	5.01
FN3KRP	5.39	5.70	5.07
SLC25A11	5.39	5.92	5.06
OTTHUMG00000017162	5.39	6.13	4.98
PHLDA2	5.39	6.17	4.78
LOC100129550	5.39	5.76	4.98
HMGB2	5.39	5.93	4.77
SH2D4A	5.39	5.86	4.59
PLVAP	5.39	6.50	4.51
RPRML	5.39	5.94	4.93
TLCD2	5.39	5.84	4.95

MCM3	5.39	5.69	4.87
OTTHUMG00000169629	5.39	5.83	4.98
CACNA1C-AS2	5.39	5.58	5.18
NOXA1	5.39	5.67	5.08
WDR34	5.39	5.94	4.89
KLHL33	5.39	5.76	5.08
OXCT1	5.39	6.05	4.52
ZBTB48	5.39	5.56	5.16
IFT88	5.39	5.70	4.87
WBSCR27	5.39	5.70	5.08
NSUN4	5.39	5.68	4.93
CELF2-AS1	5.39	5.73	4.99
PPIEL	5.39	5.70	4.85
RNA5SP84	5.39	5.93	5.14
AAMP	5.39	5.75	5.07
ZIK1	5.39	5.79	4.90
ETNK2	5.39	5.81	5.00
LINC00936	5.39	5.78	4.89
NUDT13	5.39	5.85	4.76
OTTHUMG00000150318	5.39	5.65	5.12
OTTHUMG00000037425	5.39	6.29	4.75
ITFG3	5.39	5.84	4.83
THEMIS2	5.39	5.92	4.88
MINOS1	5.39	5.76	5.01
LOC100128482	5.39	5.84	5.10
HDGFRP2	5.39	5.68	5.12
APBB1IP	5.39	6.60	3.96
RNF41	5.39	5.60	5.00
MIR326	5.38	5.92	4.73
C19orf38	5.38	5.78	5.09
VGF	5.38	5.66	5.00
MTFR1	5.38	5.72	4.95
LOC100507458	5.38	5.99	4.83
KCNJ9	5.38	5.74	4.87
ZNF7	5.38	5.69	5.04
PAPPA-AS1	5.38	6.40	4.83
MTM1	5.38	5.65	4.92
TRIM66	5.38	5.77	4.70
TUBD1	5.38	5.90	4.86
OTTHUMG00000172125	5.38	5.59	5.06
OTTHUMG00000009067	5.38	6.15	5.05
CTAGE5	5.38	5.74	4.84
KIAA1671	5.38	5.77	5.00
DNAJA3	5.38	5.65	5.08
BAG2	5.38	6.49	4.88
LMNA	5.38	6.08	4.75
NKX3-2	5.38	5.67	4.98
FMN1	5.38	5.66	5.06
HPN	5.38	5.71	5.15

OTTHUMG00000162220	5.38	5.83	4.60
GMEB2	5.38	5.68	5.23
AKT1S1	5.38	5.53	5.17
ZNF586	5.38	5.67	4.74
POLR2D	5.38	5.74	4.91
DLL1	5.38	5.93	4.94
MIR3198-1	5.38	5.82	4.95
RANBP10	5.38	5.74	5.02
CD8A	5.38	5.74	4.98
EVI2B	5.38	6.46	4.72
MEIS2	5.38	5.71	4.99
C20orf181	5.38	5.69	5.03
MAP2K5	5.38	5.80	4.67
RNGTT	5.38	5.92	4.85
DTNBP1	5.38	5.99	5.04
DAG1	5.38	5.62	5.05
STIM2	5.38	5.83	5.03
FNDC5	5.38	5.78	5.01
FBXL17	5.38	5.78	4.80
OTTHUMG00000171455	5.38	6.39	4.88
MCUR1	5.38	5.86	4.68
LOC284628	5.38	5.77	5.05
YDJC	5.38	5.81	5.09
NOP56	5.38	5.70	5.03
LOC100506963	5.38	5.52	5.11
FAM41AY1	5.38	6.03	4.82
RPL22	5.38	5.70	5.09
FLJ43763	5.38	6.15	4.98
AFG3L1P	5.38	5.62	5.01
NAPEPLD	5.38	5.91	4.72
KRT8	5.38	6.32	5.03
CDK20	5.38	5.84	5.08
LOC646588	5.38	6.68	3.73
CDNF	5.38	5.78	4.68
DLX3	5.38	6.28	4.05
RANBP6	5.38	5.97	4.95
TBL3	5.38	5.65	5.15
ROGDI	5.38	5.81	5.06
CAPN5	5.38	5.55	5.18
RSAD1	5.38	5.62	4.97
CYP21A1P	5.38	6.17	4.78
DGAT2	5.38	6.08	5.08
ZNF337	5.38	5.94	4.79
ZNF468	5.38	5.63	4.98
OTTHUMG00000074741	5.38	6.58	4.20
FAM98C	5.38	5.88	5.11
OTTHUMG00000018105	5.38	6.28	4.55
ZNF266	5.38	5.86	4.74
SNORD113-8	5.38	6.56	4.41

PPAPDC1A	5.38	5.99	4.94
MAD2L2	5.38	5.64	5.04
ANHX	5.37	5.71	5.09
SELENBP1	5.37	5.77	4.85
LOC100506379	5.37	6.04	4.91
LOC100507316	5.37	5.74	4.66
COX4I2	5.37	6.08	4.54
FLJ42102	5.37	5.65	5.17
MAGEA2	5.37	5.92	4.94
TMEM164	5.37	5.78	4.93
UEVLD	5.37	5.80	4.97
FBXW11	5.37	5.79	4.75
HRAS	5.37	5.80	4.84
PDE7A	5.37	5.81	4.95
PDE6G	5.37	5.93	4.78
SLC25A28	5.37	5.73	5.07
MADD	5.37	5.71	5.05
TRIP6	5.37	5.91	4.73
CYP2U1	5.37	5.95	4.93
LINC00523	5.37	5.96	5.14
DKFZP434F142	5.37	5.71	4.91
PPP1R9B	5.37	5.89	4.55
LAT2	5.37	5.91	5.21
TAS1R3	5.37	5.65	4.87
SNAPC2	5.37	5.76	4.83
GBA2	5.37	5.67	5.08
POM121L9P	5.37	6.49	3.89
INSC	5.37	5.93	4.75
OTTHUMG00000160774	5.37	5.70	5.01
FECH	5.37	5.80	4.88
NLRX1	5.37	5.57	5.20
RNA5SP365	5.37	5.96	4.54
MIR4646	5.37	6.00	4.49
ETV3	5.37	5.80	4.69
ACSL1	5.37	5.70	4.79
OTTHUMG00000171624	5.37	5.65	5.03
ARAP1	5.37	5.69	4.77
NSUN6	5.37	6.09	4.56
ZNF431	5.37	5.80	4.71
OXR1	5.37	6.24	4.68
ZNF410	5.37	5.91	4.89
TLR2	5.37	6.50	4.90
NUBPL	5.37	5.67	5.02
GAPDH	5.37	6.05	4.60
S100A13	5.37	5.71	4.94
BCAT2	5.37	5.73	5.03
ARG2	5.37	5.86	4.93
PYY	5.37	5.89	4.92
MAGOH	5.37	6.07	4.89

FGF14-AS1	5.37	5.69	5.01
C11orf87	5.37	5.76	5.07
LOC401397	5.37	5.77	4.86
SSBP4	5.37	5.63	4.72
HTRA3	5.37	6.06	4.91
DAPK1	5.37	6.02	4.70
PLEKHA5	5.37	5.92	4.60
ARMCX5-GPRASP2	5.37	5.70	5.05
PCNA	5.37	6.18	4.69
ZNF277	5.37	5.76	5.00
GPRIN2	5.37	5.95	5.07
OTTHUMG00000162179	5.37	5.85	5.00
BBC3	5.37	5.68	5.06
OTTHUMG00000014623	5.37	5.80	4.97
C12orf29	5.37	5.69	5.03
MRPL36	5.37	5.63	4.87
TENM1	5.37	5.83	4.89
LMTK3	5.37	5.72	5.08
TBL1X	5.37	5.84	4.94
PPFIA4	5.37	5.70	5.10
TFAP2E	5.37	5.68	5.04
TMEM132B	5.37	5.85	5.12
MX1	5.37	6.23	4.93
DAB1	5.37	5.95	4.90
KIAA0195	5.37	5.63	5.07
LOC91450	5.37	5.71	5.16
ZSCAN20	5.37	5.76	4.79
POC5	5.37	5.57	5.18
LOC100129461	5.37	5.70	5.01
NMT2	5.37	5.80	4.99
PTK6	5.37	5.67	4.98
MIRLET7A1	5.37	5.87	4.73
LOC253573	5.36	5.76	4.85
TRIM22	5.36	6.21	4.84
RSPO4	5.36	5.88	4.87
PGAP3	5.36	6.05	4.89
VGLL3	5.36	5.73	4.85
DHODH	5.36	5.69	5.13
RAB38	5.36	5.64	4.97
NDOR1	5.36	5.53	5.05
HPCAL1	5.36	5.85	4.97
SMUG1	5.36	5.83	5.05
OTTHUMG00000035956	5.36	6.16	4.56
ARHGDIB	5.36	5.72	5.08
LOC100505502	5.36	5.63	5.16
LZTS1-AS1	5.36	5.98	4.96
ZNF146	5.36	5.86	4.75
PEPD	5.36	5.92	4.53
LOC100507516	5.36	6.43	4.23

SAPCD1-AS1	5.36	5.68	5.03
TTLL12	5.36	5.78	5.02
FGD6	5.36	5.76	4.78
RBM18	5.36	5.91	4.89
LINC00617	5.36	5.81	5.06
LOC100130557	5.36	5.90	4.84
OTTHUMG00000173078	5.36	5.85	4.87
P4HA3	5.36	6.85	4.53
FAM131C	5.36	5.64	4.65
OTTHUMG00000013334	5.36	6.34	4.67
SMCR9	5.36	6.03	4.73
ZCCHC10	5.36	5.84	5.07
ST3GAL4-AS1	5.36	5.56	5.10
RRP1B	5.36	5.65	5.07
FOXF1	5.36	5.55	5.01
FAM222A	5.36	5.79	4.83
MTA1	5.36	5.73	4.94
BACH1	5.36	5.90	4.89
OTTHUMG00000086844	5.36	5.68	4.99
HPS6	5.36	5.83	4.90
TYK2	5.36	5.59	5.18
USP54	5.36	5.73	5.04
NES	5.36	6.05	4.91
GLCCI1	5.36	5.90	4.81
DKFZp779M0652	5.36	5.99	4.91
TRIM58	5.36	5.81	5.13
OTTHUMG00000058207	5.36	5.84	4.90
CDC42EP1	5.36	5.84	5.00
CSF3	5.36	5.80	4.83
CACNB3	5.36	5.66	5.19
SRGAP2-AS1	5.36	5.64	5.22
PHC2	5.36	5.75	5.03
WDR6	5.36	5.79	4.86
TSSC4	5.36	5.67	5.04
CNPPD1	5.36	5.63	4.84
SBF1	5.36	5.83	4.95
AGAP1	5.36	5.63	4.77
MTERFD1	5.36	5.67	4.94
OTTHUMG00000017963	5.36	5.89	4.95
ZDHHC3	5.36	5.74	5.03
CRAMP1L	5.36	5.60	5.06
DFNB31	5.36	5.66	4.87
OTTHUMG00000018169	5.36	5.75	5.03
SPTBN5	5.36	5.64	5.03
ZNF232	5.36	5.99	4.95
PCDHB9	5.36	5.83	4.54
SCUBE3	5.36	5.55	5.05
TMPRSS4-AS1	5.36	5.83	4.76
TBPL1	5.36	6.22	4.52

TEFM	5.36	5.95	4.91
GNAI2	5.36	5.61	4.82
PPARD	5.36	5.60	4.97
SCARF1	5.36	5.66	5.07
IER2	5.36	5.56	4.84
OTTHUMG00000170842	5.36	5.71	4.99
SLC25A5	5.36	5.95	4.83
PRICKLE2-AS3	5.36	6.16	4.88
RSPO2	5.35	6.55	4.68
LDLR	5.35	5.87	4.71
SBSN	5.35	5.90	4.97
MVB12A	5.35	6.00	4.89
TIGD5	5.35	5.63	5.03
WFIKKN1	5.35	5.84	4.95
OTTHUMG00000164793	5.35	5.98	4.53
LOC100506023	5.35	5.87	4.46
USP9Y	5.35	6.41	3.65
KLK6	5.35	5.73	4.97
BRD1	5.35	5.54	5.03
RND3	5.35	6.23	4.57
INTU	5.35	5.69	4.92
VOPP1	5.35	5.59	5.16
B4GALT4	5.35	5.67	4.88
SYT15	5.35	5.67	5.02
OTTHUMG00000179876	5.35	6.04	4.77
PNMAL2	5.35	5.76	5.08
DCBLD1	5.35	5.72	4.95
C10orf105	5.35	6.20	4.75
PYROXD1	5.35	5.87	4.61
PALB2	5.35	5.70	4.93
ITGAM	5.35	6.29	4.92
PAIP1	5.35	6.01	4.81
ARID1A	5.35	5.57	4.94
PLEKHN1	5.35	5.62	5.08
SLC41A1	5.35	5.82	4.97
GPR144	5.35	5.75	4.83
ASB16-AS1	5.35	5.73	4.83
LOC100506667	5.35	5.86	4.68
RAD51B	5.35	5.56	5.14
CRYAA	5.35	5.71	5.09
PDCL3	5.35	5.82	5.10
RXFP4	5.35	5.62	4.80
CHST12	5.35	5.66	5.14
APH1B	5.35	5.57	5.15
CYP2S1	5.35	5.77	4.81
CHRNB1	5.35	5.85	4.86
HSF1	5.35	5.68	4.93
ZMYND10-AS1	5.35	5.95	4.40
CHST1	5.35	5.88	5.13

NR2F1	5.35	5.90	4.96
GAL3ST4	5.35	6.00	4.42
CYB5D1	5.35	5.59	5.11
C10orf88	5.35	5.82	4.52
PIGO	5.35	5.88	5.08
CCDC74A	5.35	5.62	4.85
SSH2	5.35	5.67	4.89
ZNF787	5.35	6.03	4.64
RNF31	5.35	5.64	5.10
ZMYND15	5.35	5.71	4.97
FOXP3	5.35	5.96	4.92
USP37	5.35	5.69	4.83
HIST1H2BO	5.35	5.76	5.14
WNT5B	5.35	5.77	4.94
EMR2	5.35	6.02	4.73
HCCAT4	5.35	5.89	4.94
TRIM39	5.35	5.59	4.91
B9D2	5.35	5.82	4.92
OTTHUMG00000152658	5.35	5.58	5.02
AKR1C1	5.35	6.17	4.74
LOC730098	5.35	5.83	4.84
STARD10	5.35	5.79	4.87
IGHV1OR21-1	5.35	5.80	4.72
LOC100129917	5.35	5.93	4.69
SYTL1	5.35	5.66	5.04
KRTAP11-1	5.35	5.96	4.56
ASUN	5.35	6.17	4.96
WFDC10A	5.35	5.68	4.83
DNAJB5	5.35	5.81	5.03
PFKFB2	5.35	5.64	5.14
CDCA3	5.35	5.74	4.95
WIBG	5.35	5.63	4.63
DGKE	5.35	5.68	5.04
OR10G9	5.35	5.88	4.98
TPBGL	5.35	5.74	4.92
ZNF518B	5.35	5.80	4.86
C1orf86	5.35	5.69	5.03
IL2RB	5.34	5.74	4.90
OTTHUMG00000153381	5.34	5.56	5.01
VPS33B	5.34	5.63	4.81
LDLRAD4	5.34	5.83	4.89
SLC5A3	5.34	5.81	4.95
NF2	5.34	5.68	4.96
NDUFB8	5.34	5.65	4.74
KCNK12	5.34	5.84	5.03
ASAH2C	5.34	6.09	4.69
AAMDC	5.34	5.73	5.02
TRIP4	5.34	5.56	5.09
SLFN11	5.34	6.00	4.62

TRAJ54	5.34	6.08	4.67
C15orf39	5.34	5.69	4.89
OTTHUMG00000151403	5.34	5.73	4.93
PCBP3	5.34	5.96	5.04
TIMD4	5.34	7.09	3.83
LINC00413	5.34	5.81	4.97
HECA	5.34	5.71	4.90
SH2D5	5.34	5.67	5.04
MME	5.34	7.15	4.56
APTX	5.34	5.56	5.16
TFPT	5.34	5.91	4.84
DUSP7	5.34	5.72	4.86
LOC100506844	5.34	5.57	4.98
RNU12	5.34	5.72	4.38
CTRC	5.34	5.61	5.10
EPSTI1	5.34	5.76	4.88
GRB10	5.34	5.92	4.84
ETV5	5.34	6.05	4.44
FBXW4	5.34	5.83	4.56
FLI1	5.34	5.83	4.72
MIR544A	5.34	6.25	3.95
OTTHUMG00000152728	5.34	5.82	4.68
MIR920	5.34	6.47	4.69
RTN4IP1	5.34	5.85	4.77
ZNF426	5.34	5.80	5.00
PTP4A1	5.34	5.94	4.83
UNC13D	5.34	5.66	5.03
RAP1GAP2	5.34	5.89	4.82
LARP6	5.34	5.69	5.02
MCCC1	5.34	5.69	5.06
TXLNG2P	5.34	6.50	3.28
UFSP1	5.34	5.68	5.00
POLR1C	5.34	5.74	4.92
FILIP1L	5.34	5.89	4.84
MTMR9	5.34	5.86	4.90
ZNF420	5.34	5.79	4.49
SLURP1	5.34	5.68	4.71
ZNF609	5.34	5.73	4.97
OCRL	5.34	6.27	4.37
SOD2	5.34	6.01	4.58
MIR298	5.34	5.60	4.90
LINC00671	5.34	6.05	4.94
UNC93B1	5.34	5.55	5.04
MEGF8	5.34	5.87	5.04
HCG26	5.34	5.87	4.88
C15orf57	5.34	5.69	4.45
ELP4	5.34	5.76	4.85
ZKSCAN7	5.34	5.57	5.13
LOC100132815	5.34	6.09	4.81

C9orf139	5.34	5.94	4.82
NCR3	5.34	5.63	5.10
LGI2	5.34	6.29	4.00
MCU	5.34	5.78	4.75
ANKRD36C	5.34	5.78	5.03
CPT1A	5.34	5.79	4.90
CASP7	5.34	5.62	4.88
MYADML2	5.34	5.66	4.72
PPAPDC3	5.34	5.67	4.87
FAM203A	5.34	5.52	5.11
INVS	5.34	5.69	4.83
SRXN1	5.34	5.70	4.78
OTTHUMG00000171735	5.34	5.88	5.03
FANCC	5.34	5.75	4.79
ZNF529	5.34	5.65	5.10
LOC100506603	5.34	5.75	4.99
HTRA2	5.34	5.94	4.77
EXOSC9	5.34	5.77	4.63
PTCH1	5.34	6.34	4.28
TEX264	5.34	5.59	5.13
DDX23	5.34	5.74	4.88
NCBP2-AS2	5.34	5.74	4.89
KNTC1	5.34	5.81	4.76
SCYL1	5.34	5.77	4.89
ZHX2	5.33	5.63	5.00
C16orf3	5.33	5.79	4.87
HRC	5.33	5.85	4.96
OTTHUMG00000042244	5.33	6.39	4.75
TCEAL1	5.33	5.57	5.10
C2CD4A	5.33	5.59	5.03
GNB5	5.33	5.58	5.07
CEP85	5.33	5.78	4.99
CBX7	5.33	5.89	4.79
CNNM3	5.33	5.74	4.95
MEGF9	5.33	5.52	4.88
PLK3	5.33	5.83	5.02
NAGLU	5.33	5.67	5.02
FBXO10	5.33	5.61	5.06
CD84	5.33	6.47	4.67
GPN2	5.33	5.72	4.99
GPR101	5.33	5.80	4.77
TBRG1	5.33	5.87	4.80
PLXNA3	5.33	5.60	4.87
OTTHUMG00000041501	5.33	5.81	5.04
MIR152	5.33	5.76	4.86
TRIM5	5.33	5.88	5.04
SLC31A2	5.33	5.98	5.00
TRMT2B-AS1	5.33	5.62	4.82
ABCC5	5.33	5.64	5.07

ALPK1	5.33	5.62	5.15
ARRDC1	5.33	5.55	4.82
SELO	5.33	5.64	4.75
FBXO17	5.33	5.49	5.05
S100PBP	5.33	5.66	4.82
SYT2	5.33	5.79	5.01
WDR62	5.33	5.56	5.07
BPHL	5.33	5.56	4.84
RNPEPL1	5.33	5.49	5.02
RNF165	5.33	5.91	4.89
ZNF385A	5.33	5.84	4.44
DNASE2	5.33	5.83	4.92
OTTHUMG00000170505	5.33	5.78	4.69
RAB7L1	5.33	5.70	5.00
LOC100294145	5.33	5.67	4.96
XAB2	5.33	5.81	4.90
MIR22HG	5.33	5.67	4.56
EXOC8	5.33	5.65	4.94
MTG2	5.33	5.65	4.92
PPM1A	5.33	5.64	4.86
LATS2	5.33	5.94	4.86
SOGA1	5.33	5.68	4.91
NSL1	5.33	5.63	4.67
LMF2	5.33	5.56	5.07
GGCT	5.33	5.76	4.49
CRYBB2	5.33	5.69	4.83
ALPL	5.33	6.13	4.50
TGIF1	5.33	5.77	4.83
SDE2	5.33	5.94	4.71
SLC25A47	5.33	5.61	4.99
ZNF219	5.33	5.66	5.07
OTTHUMG00000169968	5.33	6.05	4.82
MINA	5.33	5.60	4.88
ZNF10	5.33	5.64	4.55
MTG1	5.33	5.73	4.95
THSD7A	5.33	5.91	4.45
HMHB1	5.33	5.96	4.89
MAP2K2	5.33	5.83	4.89
GTPBP3	5.33	5.79	4.98
NDUFB10	5.33	5.63	4.76
REM1	5.33	5.67	4.84
MESP2	5.33	5.59	5.07
CSF2RB	5.33	5.68	4.84
OSCP1	5.33	5.67	5.00
SIPA1	5.33	5.98	4.77
RBP1	5.33	5.80	4.91
EIF4EBP1	5.33	6.10	4.83
KATNA1	5.33	5.81	4.64
SPNS1	5.33	5.87	4.90

FCGR2B	5.33	5.98	4.45
RPRD1B	5.33	5.60	5.00
ABAT	5.33	5.74	5.00
LOXL4	5.33	5.89	4.78
SMLR1	5.33	5.65	5.00
C3orf14	5.33	5.86	4.84
LXN	5.33	5.92	4.61
TATDN2	5.33	5.65	4.92
ASCC1	5.33	5.65	4.89
APOA1	5.33	5.71	4.84
PNPLA8	5.33	6.11	4.46
MTFR1L	5.32	6.08	4.65
LIG1	5.32	5.55	5.09
DOK7	5.32	5.80	4.98
SQLE	5.32	5.89	4.64
EMC9	5.32	5.77	4.86
SELE	5.32	6.35	4.23
LINC00684	5.32	5.76	4.97
SNRPC	5.32	5.65	4.95
TSPAN14	5.32	5.77	4.96
COL5A1-AS1	5.32	6.56	4.53
RGS5	5.32	5.97	4.64
UTP3	5.32	6.04	4.85
GNE	5.32	5.69	4.98
UBP1	5.32	5.71	4.95
PFKFB4	5.32	5.84	4.98
PLIN3	5.32	5.90	4.69
SIRT5	5.32	5.65	5.14
TRMT11	5.32	5.70	4.88
OTTHUMG00000041297	5.32	5.78	4.88
RUSC2	5.32	5.91	4.73
EXOSC2	5.32	5.65	5.04
FZD1	5.32	6.23	4.48
LOC100130000	5.32	6.02	4.92
RNA5SP338	5.32	6.45	4.44
TMEM203	5.32	5.94	4.49
MAR8	5.32	5.58	5.12
CREB3L3	5.32	5.75	5.09
FAM210A	5.32	5.67	4.79
TEX29	5.32	5.73	4.84
TRIT1	5.32	5.90	4.61
TNF	5.32	5.93	4.98
TREX1	5.32	5.61	5.14
VPS11	5.32	5.55	4.90
THAP3	5.32	5.78	4.88
RP5-905G11.4	5.32	5.82	5.05
LINC00650	5.32	5.77	4.65
MTO1	5.32	5.69	4.81
C9orf62	5.32	5.70	4.88

FAM13A-AS1	5.32	5.77	4.94
COMMD9	5.32	5.67	4.77
GNA11	5.32	5.79	4.85
TMEM125	5.32	5.84	5.00
FOXK1	5.32	5.69	5.01
COLQ	5.32	5.64	4.97
KALRN	5.32	5.63	4.94
NPRL2	5.32	5.57	5.01
UQCC	5.32	5.62	4.99
NAF1	5.32	5.52	5.09
OTTHUMG00000170275	5.32	5.84	4.95
MIPOL1	5.32	5.67	5.02
MTMR7	5.32	5.92	4.44
PIM3	5.32	5.68	5.02
BPIFB2	5.32	5.89	5.01
TMEM186	5.32	5.63	4.93
PLA2G6	5.32	5.52	5.15
RMDN2	5.32	5.65	5.01
ARL2-SNX15	5.32	5.52	5.06
KLF5	5.32	5.51	5.12
TRIM49D2P	5.32	6.21	4.86
USP28	5.32	5.69	4.92
ARSD-AS1	5.32	5.65	4.61
CCDC111	5.32	5.75	4.86
GTF3C1	5.32	5.67	4.96
PPM1K	5.32	5.75	4.83
IL17D	5.32	5.79	4.82
LOC100996870	5.32	5.87	4.72
DOK4	5.32	5.59	4.96
OTTHUMG00000168177	5.32	5.67	5.00
CLCN3	5.32	5.90	4.67
DCUN1D2-AS2	5.32	5.74	4.70
PCCB	5.32	5.51	5.03
LINC00582	5.32	6.14	4.53
PRKD2	5.32	5.77	5.05
MAP3K14-AS1	5.32	5.58	4.90
ENDOV	5.32	5.68	5.05
MED29	5.32	5.75	4.84
SRP54	5.32	5.80	4.94
ERI3	5.32	5.56	4.94
DHTKD1	5.31	5.53	4.96
RAMP1	5.31	5.57	4.88
WASH4P	5.31	6.02	4.11
ATP5G2	5.31	5.83	4.86
HARS	5.31	5.73	4.54
TOPORS-AS1	5.31	5.74	4.86
HDAC10	5.31	5.73	4.83
FDPS	5.31	5.65	4.74
LINC00634	5.31	5.88	4.63

FAM149A	5.31	5.75	5.08
OTTHUMG00000086913	5.31	5.68	5.12
PRR14L	5.31	5.65	4.88
MIR320C1	5.31	5.69	5.08
SHE	5.31	5.58	4.99
GRIK3	5.31	5.64	5.11
OSBPL10	5.31	5.88	4.94
RFX3	5.31	5.66	5.08
SNX33	5.31	6.16	4.63
CIITA	5.31	6.12	4.99
LMNB2	5.31	5.76	5.02
PEX7	5.31	5.53	5.05
BARHL1	5.31	5.66	4.93
GMIP	5.31	5.55	5.08
MIR548I3	5.31	5.65	4.64
SWSAP1	5.31	5.52	4.97
NDUFB2-AS1	5.31	5.58	5.02
C11orf91	5.31	5.70	4.99
CHN2	5.31	5.58	4.78
LOC100506978	5.31	5.73	5.02
SNTB1	5.31	6.02	4.50
SLC5A9	5.31	5.61	5.10
CPSF3	5.31	5.80	4.92
SNORA55	5.31	5.84	4.91
ANLN	5.31	6.24	4.49
PIGZ	5.31	5.61	5.03
MR1	5.31	5.84	4.64
OTTHUMG00000180211	5.31	5.83	4.94
VARS2	5.31	5.80	5.10
OTTHUMG00000164048	5.31	6.23	4.52
HSF2	5.31	5.74	4.93
CARD9	5.31	5.74	4.85
MCM4	5.31	5.66	4.83
ANXA8L2	5.31	5.65	5.00
LOC150381	5.31	5.60	4.68
SMIM8	5.31	5.84	4.55
ZNF446	5.31	5.65	5.10
LOC728989	5.31	5.73	4.77
B4GALT7	5.31	5.78	4.85
TTTY22	5.31	5.67	4.91
CES1	5.31	6.11	4.33
GNPDA2	5.31	5.74	4.43
ZNF114	5.31	5.62	4.70
LYNX1	5.31	5.63	5.08
CENPP	5.31	6.02	4.67
ADRB3	5.31	5.67	5.08
ZBTB14	5.31	5.88	4.74
LOC283501	5.31	5.95	4.81
HJURP	5.31	5.80	5.07

RNU6-64P	5.31	5.71	5.00
ZNF184	5.31	5.67	4.83
IQCC	5.31	5.65	4.86
TIE1	5.31	5.74	4.92
MBD3L4	5.31	5.94	5.01
OBP2A	5.31	5.69	4.34
ADAMTS10	5.31	5.85	5.02
LOC374443	5.31	5.87	4.50
LOC642648	5.31	5.90	4.07
MRPL28	5.31	5.67	5.02
SKA2	5.31	5.65	4.82
BAG4	5.31	5.66	4.83
SHISA7	5.31	5.85	5.03
TNNC2	5.31	5.64	4.72
TCAP	5.31	5.96	4.75
ILVBL	5.31	5.56	4.85
RPUSD3	5.31	5.53	5.02
ZNF334	5.31	5.85	4.71
OR8B3	5.31	6.03	4.44
HOXC4	5.31	5.68	4.95
C9orf141	5.31	5.72	5.02
OTTHUMG00000161856	5.31	6.08	4.76
MMAA	5.31	5.82	4.87
C11orf49	5.31	5.62	5.05
RGP1	5.31	5.68	4.85
NOTCH1	5.31	5.70	4.80
SLC17A9	5.31	5.54	4.94
ETAA1	5.31	5.84	4.59
MCPH1	5.31	5.60	4.83
SMTN	5.31	5.48	4.99
TFAMP1	5.31	5.82	5.03
DSG2	5.31	6.91	4.43
CDAN1	5.31	5.62	4.79
PEX12	5.31	5.58	5.07
MIR4698	5.31	5.72	4.84
AMTN	5.31	7.28	3.25
THTPA	5.31	5.57	5.05
COL8A2	5.31	5.93	4.86
TNFRSF10D	5.30	6.52	4.52
TSNAX-DISC1	5.30	5.52	5.07
KRTAP1-5	5.30	5.93	4.36
LRRN4CL	5.30	5.57	4.94
OTTHUMG00000020691	5.30	5.83	5.11
RFC5	5.30	5.75	4.97
FAM127C	5.30	5.85	4.74
NPDC1	5.30	5.72	4.88
STX16-NPEPL1	5.30	5.65	5.11
LEPREL1	5.30	6.51	4.68
MDFIC	5.30	5.81	4.97

PIDD	5.30	5.58	4.96
FAM107B	5.30	5.56	4.78
NFRKB	5.30	5.73	4.97
FAM184B	5.30	5.71	5.00
AGTPBP1	5.30	5.70	4.80
TMEM134	5.30	5.78	4.71
POLR3E	5.30	5.48	5.08
GZF1	5.30	5.55	5.10
PCDH12	5.30	5.78	4.99
FAM69A	5.30	5.54	4.93
PPARG	5.30	5.95	4.43
NUDT14	5.30	5.59	4.99
SNORA74B	5.30	5.93	4.72
ZNF8	5.30	5.53	4.94
MRPS18C	5.30	5.81	4.73
VEGFB	5.30	6.08	4.82
LOC100507646	5.30	5.76	4.56
PTPRCAP	5.30	5.87	4.64
BCAR3	5.30	5.48	5.09
CCR7	5.30	6.11	4.92
MRPL4	5.30	5.56	5.02
NVL	5.30	5.92	4.68
ZNF182	5.30	5.80	4.60
RIMS4	5.30	5.57	4.84
UBAP1	5.30	5.84	4.59
PCYOX1L	5.30	5.82	4.99
DMKN	5.30	5.73	4.82
GGA3	5.30	5.70	4.85
C19orf47	5.30	5.41	5.18
PLCG2	5.30	5.82	4.93
MT1IP	5.30	5.95	4.90
PGF	5.30	5.60	4.92
LYN	5.30	5.67	4.86
TSPYL2	5.30	5.64	4.80
FAM58A	5.30	5.57	4.86
SIL1	5.30	5.67	4.97
TUBA1C	5.30	5.43	5.11
OR2J3	5.30	5.76	4.79
AGTRAP	5.30	5.75	4.97
MIR4268	5.30	5.74	4.83
RAVER1	5.30	5.68	4.84
GDPD3	5.30	5.70	4.82
ATP6V0E2-AS1	5.30	5.78	4.77
MCM7	5.30	5.79	4.79
RHOC	5.30	5.64	5.01
BCO2	5.30	5.83	4.97
AMOTL2	5.30	5.73	5.01
TEX19	5.30	5.68	4.93
FKBP14	5.30	6.27	4.39

OTTHUMG00000164495	5.30	6.11	4.59
GPR88	5.30	5.96	4.79
SREBF1	5.30	5.61	4.93
OTTHUMG00000132690	5.30	5.65	5.01
FLJ44313	5.30	5.70	4.91
PPP1R3G	5.30	5.81	4.91
KCNQ4	5.30	5.57	5.07
ARHGEF18	5.30	5.57	4.88
ZNF354C	5.30	5.73	4.71
SMPD3	5.30	5.67	4.96
SLC39A4	5.30	5.83	5.04
KRT18P55	5.30	5.67	4.98
NOP14-AS1	5.30	5.61	4.99
UVRAG	5.30	5.72	4.60
ZNF280C	5.30	5.78	3.88
MIOS	5.30	5.64	4.59
PGBD1	5.30	5.50	5.07
PCNA-AS1	5.30	5.74	4.92
CMTM3	5.30	6.30	4.65
KLC4	5.30	5.78	5.02
PANX1	5.30	6.23	4.63
ZNF765	5.30	5.52	5.01
TRAF4	5.30	5.82	5.04
EXOC6B	5.30	5.93	4.60
TAPBPL	5.30	5.79	4.98
CAND2	5.30	5.66	4.93
LOC100132984	5.30	5.80	4.94
SLC25A44	5.30	5.46	5.12
BCKDHA	5.30	5.74	4.95
RGS3	5.30	5.52	4.99
CLDN25	5.30	5.74	4.92
TIGD6	5.30	5.69	4.95
CDC40	5.30	5.69	4.75
ZBTB33	5.30	5.79	4.93
BIRC8	5.30	5.64	5.01
MIR4530	5.30	6.31	4.56
LZTFL1	5.29	5.72	4.92
BANF1	5.29	5.71	4.98
PLCH2	5.29	5.63	4.98
C2orf62	5.29	5.63	5.00
KRT81	5.29	5.80	4.70
RTN4RL1	5.29	5.66	4.97
BNIP2	5.29	5.64	4.52
FUT5	5.29	6.03	4.66
COIL	5.29	5.64	4.57
CCZ1B	5.29	5.65	4.52
CYTH4	5.29	5.74	4.88
LOC284751	5.29	5.68	4.88
ACVR2A	5.29	5.59	4.90

SMAP1	5.29	5.73	5.03
ANXA8L1	5.29	5.74	5.01
PITX3	5.29	6.02	4.98
CAAP1	5.29	5.69	4.34
COL9A1	5.29	6.10	4.19
WASH3P	5.29	5.97	3.80
LOC100128386	5.29	5.62	4.92
AGPAT1	5.29	5.64	5.03
NEGR1	5.29	5.85	4.69
APOC3	5.29	5.74	5.01
TTC33	5.29	5.75	4.88
HOMEZ	5.29	5.66	5.05
RSPH3	5.29	5.75	4.44
TTC39C	5.29	5.67	4.72
PRPF19	5.29	5.64	4.88
SMAP2	5.29	5.75	5.01
ZW10	5.29	5.72	4.92
ATP1A3	5.29	5.68	4.99
RMND1	5.29	5.80	4.49
FLJ22184	5.29	5.79	4.94
MIR330	5.29	5.80	4.79
FAM174B	5.29	5.55	5.01
TPBG	5.29	5.63	4.80
TTC32	5.29	5.66	4.81
KRTDAP	5.29	5.80	4.97
TGFBR3L	5.29	5.70	4.94
SFI1	5.29	5.48	4.96
LILRB2	5.29	6.09	4.55
RAB3IL1	5.29	5.80	4.88
NCAPH2	5.29	5.97	4.82
TATDN3	5.29	5.59	4.87
LZTS1	5.29	5.62	4.98
SPATA13-AS1	5.29	5.85	4.58
A1BG	5.29	5.46	5.07
B3GNT6	5.29	5.59	4.84
LRP8	5.29	5.64	4.81
CTNNBIP1	5.29	5.55	5.04
LOC90784	5.29	5.67	4.97
FAM83C-AS1	5.29	5.96	4.68
VASH1	5.29	5.68	4.96
MCM2	5.29	5.50	4.99
GLYCTK-AS1	5.29	5.59	4.77
SATB2	5.29	6.25	4.67
SLC2A12	5.29	6.08	4.07
GSTM4	5.29	5.76	4.98
NICN1-AS1	5.29	5.79	4.83
C9orf72	5.29	6.01	4.80
PROSC	5.29	5.56	4.75
CST7	5.29	5.72	4.99

C2CD4C	5.29	5.55	4.98
CHERP	5.29	5.63	4.92
DIRAS1	5.29	5.97	4.79
KBTBD7	5.29	5.68	4.62
AMDHD2	5.29	5.53	4.92
TAPT1	5.29	5.64	5.10
MAPK8	5.29	5.82	4.55
MORC3	5.29	5.79	4.62
CPLX3	5.29	5.86	4.71
PCDHB4	5.29	5.85	4.23
LDHD	5.29	5.71	4.83
SLC5A2	5.29	5.66	4.88
KHSRP	5.29	5.66	4.77
CYB5R4	5.29	5.89	4.42
ATXN7L2	5.29	5.55	4.87
GALC	5.29	5.79	4.81
YEATS4	5.29	6.09	4.46
OR7E47P	5.29	5.72	4.76
CADPS2	5.29	5.88	4.79
ST3GAL2	5.29	5.76	5.11
PNMA2	5.29	5.72	4.86
ANKRD23	5.29	5.73	5.01
PPP2R3A	5.29	6.34	4.45
CPNE6	5.29	5.66	4.99
STX11	5.29	5.57	5.03
COL23A1	5.29	5.67	4.98
SLC25A43	5.29	5.65	4.90
ARL10	5.29	5.60	4.83
SCARNA27	5.29	5.73	4.79
SLC30A5	5.29	5.72	4.81
LOC100506235	5.29	5.63	4.81
LRRTM2	5.29	5.81	4.58
SMAD9	5.28	5.68	4.54
ATG16L2	5.28	5.56	4.89
WWTR1-AS1	5.28	5.57	4.82
KIAA0922	5.28	5.85	4.67
IFIT3	5.28	5.78	4.84
GPR6	5.28	5.50	5.03
CCBL2	5.28	5.62	4.54
ARMCX2	5.28	5.56	4.73
TMEM159	5.28	5.63	4.57
AUH	5.28	5.47	4.75
ZNF587B	5.28	5.61	4.62
LRRK1	5.28	5.68	4.90
PAXIP1	5.28	5.56	4.80
CMTM7	5.28	5.63	4.97
PTGIR	5.28	5.56	4.79
KLHL8	5.28	5.83	4.91
POMT1	5.28	5.54	4.80

ADCY9	5.28	5.59	4.66
OTTHUMG00000153230	5.28	5.76	4.70
ST7L	5.28	5.62	4.85
L3MBTL3	5.28	5.68	4.87
C11orf30	5.28	5.57	4.78
SPATS2L	5.28	5.55	4.73
PXN	5.28	5.59	4.81
RASGEF1B	5.28	6.11	4.54
FASTKD1	5.28	5.73	4.79
C9orf169	5.28	5.52	4.99
LINC00271	5.28	5.52	4.86
SDCBP2	5.28	5.71	4.97
ZNF267	5.28	6.42	4.32
OTTHUMG00000167979	5.28	5.68	4.53
RASSF4	5.28	5.74	5.00
P2RX4	5.28	5.78	4.69
KLK10	5.28	5.68	4.79
SNORD113-4	5.28	6.73	4.26
MAGI2	5.28	5.84	4.75
CD276	5.28	6.14	4.26
ACTL7B	5.28	5.79	4.85
VSIG2	5.28	5.87	4.82
SNORA71D	5.28	6.14	4.73
TOR1B	5.28	5.75	4.79
ABCB7	5.28	5.67	4.79
MAP1LC3A	5.28	5.77	4.91
KREMEN2	5.28	5.49	5.08
LOC100506314	5.28	5.75	4.66
KIAA1161	5.28	5.62	5.05
C10orf137	5.28	5.55	5.00
ZDHHC19	5.28	5.72	4.88
C2orf53	5.28	5.70	4.88
TNFRSF1B	5.28	5.59	4.69
TRNAI2	5.28	5.60	4.63
PRLH	5.28	5.71	4.80
RGPD4	5.28	5.53	4.92
NTM	5.28	5.67	5.07
LOC729177	5.28	5.62	4.73
SOST	5.28	5.78	4.62
PYGB	5.28	5.63	4.88
ZNHIT6	5.28	5.84	4.73
CHKB-CPT1B	5.28	5.58	4.91
OTTHUMG00000176185	5.28	5.65	4.87
SLC35E2B	5.28	5.56	4.89
PIK3AP1	5.28	5.72	5.00
PPAP2C	5.28	5.56	4.86
OPN4	5.28	5.77	4.94
OTTHUMG00000036214	5.28	5.54	5.05
TMED1	5.28	5.80	4.75

HVCN1	5.28	5.57	4.93
S100A16	5.28	5.86	4.94
OTTHUMG00000019287	5.28	5.61	4.79
DCDC2B	5.28	5.53	4.86
OTTHUMG00000015926	5.28	5.87	4.54
IGFLR1	5.28	5.62	5.02
NMB	5.28	6.40	4.64
GATA3	5.28	5.56	4.86
FCN2	5.28	5.84	4.47
FAM212B	5.28	5.67	4.74
MRPL24	5.28	5.85	5.06
POT1	5.28	6.09	4.71
FBXO33	5.28	5.69	4.74
FLVCR1	5.28	5.73	4.67
OTTHUMG00000168623	5.28	5.61	4.94
MXD1	5.28	5.68	4.73
KLHL24	5.28	5.66	4.79
OTTHUMG00000168666	5.28	5.96	4.62
RNF186	5.28	5.62	5.09
SEMA4B	5.28	5.41	5.12
TLE4	5.28	5.79	5.03
RPP25L	5.28	5.55	5.15
LOC100130370	5.28	5.59	4.89
ANKRD65	5.28	5.54	4.96
MAR9	5.28	5.52	4.94
CLEC18C	5.28	6.26	4.58
CRNDE	5.28	5.84	4.52
IPO4	5.28	5.50	4.98
SNHG7	5.28	5.67	4.97
CCNJ	5.27	5.76	4.85
TARBP1	5.27	5.72	4.59
OTTHUMG00000022472	5.27	5.74	4.73
KHNYN	5.27	5.61	4.90
RNF113B	5.27	5.63	4.99
SLC7A1	5.27	5.81	4.94
VAPB	5.27	6.04	4.65
SMARCD3	5.27	5.64	4.98
NXT1	5.27	5.58	4.98
SLC35D2	5.27	5.68	4.71
NUDT17	5.27	5.44	5.05
ATP5G1	5.27	5.59	4.87
TTR	5.27	5.75	4.97
RHEB	5.27	5.55	4.92
KIR2DL4	5.27	5.80	4.87
NGDN	5.27	5.52	4.91
ULK4	5.27	5.59	4.97
MED6	5.27	5.62	4.95
LOC729603	5.27	5.73	4.53
OTUB1	5.27	5.86	4.78

CREB3L4	5.27	5.92	4.90
PFKL	5.27	5.57	5.07
AHCY	5.27	5.56	4.95
COQ10A	5.27	5.57	5.09
LRRC16B	5.27	5.77	5.04
GTF2IRD2P1	5.27	5.70	4.69
SNORD115-22	5.27	6.32	4.12
C12orf45	5.27	5.60	4.94
MAMDC4	5.27	5.54	4.89
TAF1L	5.27	5.62	5.03
PLXNB1	5.27	5.78	5.06
AKR1C2	5.27	6.08	3.77
PHF8	5.27	5.57	4.81
S1PR2	5.27	5.80	4.68
CAMSAP1	5.27	5.68	4.72
MTMR6	5.27	5.78	4.95
LINC00869	5.27	5.59	4.88
OTTHUMG00000165260	5.27	6.15	4.34
AMBRA1	5.27	5.65	4.91
ARHGAP9	5.27	5.62	4.97
RNF207	5.27	5.70	4.77
EFR3B	5.27	5.60	4.81
C1QTNF7	5.27	5.56	4.88
OTTHUMG00000170972	5.27	5.49	4.94
TMEM190	5.27	5.70	4.89
PRDM2	5.27	5.77	4.91
PRKACB	5.27	5.64	4.73
ZNF687	5.27	5.83	4.48
SPON2	5.27	5.61	4.69
CROCCP2	5.27	5.88	4.81
PARP16	5.27	5.73	4.77
CCL22	5.27	5.68	4.64
LOC728175	5.27	6.13	4.84
STRN4	5.27	5.55	5.01
LOC100505564	5.27	5.85	4.61
CAP2	5.27	5.88	4.48
OTTHUMG00000170566	5.27	5.66	4.90
ZNF559	5.27	5.58	4.99
LTB4R2	5.27	5.78	4.76
CCDC8	5.27	5.47	5.02
RPL7A	5.27	5.74	4.96
ALKBH8	5.27	5.51	5.02
LCLAT1	5.27	5.83	4.88
OTTHUMG00000090402	5.27	5.54	4.90
SAMD1	5.27	5.61	4.99
ENTPD5	5.27	5.69	4.76
MIR4644	5.27	5.75	4.87
GFRA2	5.27	6.04	4.70
OR8U1	5.27	6.00	4.74

LOC100507557	5.27	5.67	4.82
LOC100129111	5.27	5.68	4.89
TRAPPC9	5.27	5.49	4.97
ZBTB45	5.27	5.62	4.89
CA4	5.27	5.59	4.83
SIRT6	5.27	5.67	4.91
GJC2	5.27	5.70	4.66
MCRS1	5.27	5.58	4.71
CSMD2	5.27	5.94	4.80
LOC100506469	5.27	5.48	4.98
MIR610	5.27	6.15	4.70
IQCG	5.27	5.76	4.76
EMCN	5.27	6.31	3.91
PLD4	5.27	5.96	4.61
UBE2L6	5.27	5.80	4.67
SZT2-AS1	5.27	5.58	4.90
GPR142	5.26	5.83	5.01
TGDS	5.26	5.75	4.49
HCLS1	5.26	5.91	4.81
FOXO4	5.26	5.44	5.05
PGBD2	5.26	5.54	5.06
ZNF202	5.26	5.65	4.99
ABHD6	5.26	5.57	4.95
CASKIN2	5.26	5.46	4.90
RPL13	5.26	5.70	4.67
FAM25E	5.26	5.93	4.68
SLC38A10	5.26	5.53	5.05
OR7C2	5.26	5.61	4.83
PFAS	5.26	5.53	4.87
FAM177A1	5.26	5.56	4.80
RTN4R	5.26	5.67	4.91
SSSCA1-AS1	5.26	5.64	4.72
HEY2	5.26	5.81	4.79
PCBP4	5.26	5.62	4.96
ZC3H18	5.26	5.68	4.66
SSR2	5.26	5.56	4.85
RASD1	5.26	5.65	4.94
SLC25A16	5.26	5.75	4.61
CCNB1	5.26	6.38	4.23
PRIM1	5.26	5.72	4.66
PRDM11	5.26	5.58	4.78
DESI1	5.26	5.64	4.73
ACO2	5.26	5.60	4.29
TERF1	5.26	5.68	4.81
SPRED2	5.26	5.56	4.76
NFAM1	5.26	5.70	4.71
PNMAL1	5.26	5.68	4.82
PLEK	5.26	5.72	4.55
LOC100507283	5.26	5.73	4.82

TADA2A	5.26	5.69	4.84
LOC646471	5.26	5.62	4.74
ABHD11	5.26	5.49	4.92
PRICKLE3	5.26	5.69	5.07
MPV17	5.26	5.65	4.80
PABPC1L2B	5.26	6.22	4.92
LOC646278	5.26	6.24	4.50
MRPL2	5.26	5.64	4.40
LOC100506948	5.26	6.14	4.38
REXO4	5.26	5.59	5.05
OTTHUMG00000155980	5.26	5.84	4.83
FUOM	5.26	5.69	4.68
FAM95A	5.26	5.79	4.42
RELA	5.26	5.58	4.81
SEMA4C	5.26	5.68	4.63
BAIAP3	5.26	5.72	4.93
RPAP1	5.26	5.74	4.94
COQ7	5.26	5.88	4.76
NQO1	5.26	5.52	4.48
CD2	5.26	5.80	4.90
MIRLET7G	5.26	5.58	4.64
RNU7-23P	5.26	5.74	4.40
CLN3	5.26	5.74	4.84
LOC115110	5.26	5.67	5.02
LOC440896	5.26	5.77	4.96
FOXD4L2	5.26	6.01	4.51
FRMD6-AS1	5.26	5.47	4.98
MIR770	5.26	5.89	4.51
OTTHUMG00000152824	5.26	5.81	4.62
ADCK5	5.26	5.66	4.93
ASPDH	5.26	5.73	4.74
OTTHUMG00000150919	5.26	5.52	4.78
EIF2B2	5.26	5.68	4.79
PRSS50	5.26	5.52	4.74
OTTHUMG00000152980	5.26	6.50	4.47
TBC1D9B	5.26	5.58	4.92
COASY	5.26	5.53	4.91
ZNF385D-AS1	5.26	6.01	4.46
SATB1	5.26	5.57	4.80
UBOX5	5.26	5.51	5.03
USB1	5.26	5.59	4.90
PALM	5.26	5.56	5.11
TF	5.26	6.46	4.48
ZNF792	5.26	5.58	5.09
INSL3	5.26	5.64	4.84
AKR1E2	5.26	5.85	4.94
RP11-309M23.1	5.26	5.65	4.84
HCG21	5.26	5.88	4.89
SH3TC1	5.26	5.57	4.94

ANXA2P1	5.26	5.86	4.57
GPATCH2	5.26	5.60	4.69
TOE1	5.26	5.52	4.88
SLAMF8	5.26	5.63	5.00
ASIC4	5.25	5.57	4.71
FEZ1	5.25	5.81	4.69
TIMM9	5.25	5.90	4.46
TTBK1	5.25	5.50	5.06
ASPRV1	5.25	5.53	4.91
MAGI1-AS1	5.25	5.90	4.73
SIKE1	5.25	5.67	4.59
MIR933	5.25	5.78	4.65
LZTS3	5.25	5.65	4.93
NUP35	5.25	5.52	4.94
KIF7	5.25	5.43	5.00
COX14	5.25	5.73	4.69
SGIP1	5.25	6.09	4.62
ABCC10	5.25	5.53	4.96
ARID5A	5.25	5.50	5.05
CDX2	5.25	5.87	4.89
FAM91A1	5.25	5.69	4.47
NUDT16P1	5.25	5.61	4.99
PCDHB14	5.25	5.81	4.34
MIR4279	5.25	6.19	4.12
MAOB	5.25	6.06	4.45
TRAFD1	5.25	5.63	4.40
RASSF8-AS1	5.25	5.85	5.03
ZNF706	5.25	5.55	4.86
LOC151009	5.25	6.20	4.27
POLM	5.25	5.44	5.07
LOC100505624	5.25	5.50	5.03
ALKBH2	5.25	5.58	4.88
LINC00637	5.25	5.53	4.97
KDELC1	5.25	6.35	4.39
OSR1	5.25	5.70	4.72
RBM38	5.25	5.73	4.91
FAM167B	5.25	5.86	4.64
AP2A2	5.25	5.62	4.70
TRIM41	5.25	5.46	5.10
MIRLET7DHG	5.25	5.61	4.73
ABHD16B	5.25	5.65	4.85
LINC00476	5.25	5.57	4.96
NUDT16	5.25	5.73	4.50
ZNF626	5.25	5.76	4.63
AATK-AS1	5.25	5.42	4.81
USH1G	5.25	5.53	4.91
ADM	5.25	6.02	4.73
ZBTB2	5.25	5.75	4.30
MVD	5.25	5.58	4.87

FAM27E3	5.25	6.01	4.67
TPPP	5.25	5.64	4.87
OTTHUMG00000170462	5.25	5.75	4.89
OTTHUMG00000162884	5.25	5.51	5.01
RPS12	5.25	5.63	4.56
HDAC5	5.25	5.49	4.82
MTERF	5.25	6.17	4.69
TRIM39-RPP21	5.25	5.59	4.97
FAM63B	5.25	5.99	4.70
C1orf159	5.25	5.49	4.91
CNRIP1	5.25	5.77	4.74
KLF13	5.25	5.50	4.80
MIR589	5.25	5.59	4.83
INE1	5.25	5.76	4.53
TOR2A	5.25	5.77	4.99
TMPO	5.25	5.77	4.87
PRRT2	5.25	5.77	4.70
RNA5SP399	5.25	5.89	4.77
CCDC149	5.25	5.54	4.94
CTBP1	5.25	5.54	4.97
GTF3C5	5.25	5.72	4.83
DMRTC1B	5.25	5.82	4.67
MMP19	5.25	6.13	4.42
FILIP1	5.25	5.84	4.75
LOC100131392	5.25	5.97	4.34
MKS1	5.25	5.54	4.84
MMAB	5.25	5.47	4.87
TARP	5.25	5.83	4.93
PHKG2	5.25	5.78	4.68
LOC100506551	5.25	5.71	4.84
OTTHUMG00000159172	5.25	5.63	4.71
GNRH1	5.24	5.71	4.94
APIP	5.24	5.73	4.69
LOC100507443	5.24	5.58	4.80
FCGR3A	5.24	6.08	4.67
SLC7A7	5.24	5.70	4.56
YIF1B	5.24	5.44	5.08
CSH2	5.24	5.85	4.56
ZNF383	5.24	6.07	4.75
CCDC124	5.24	5.53	5.00
ANKRD18A	5.24	5.60	4.83
FLJ33360	5.24	5.59	4.92
RPS17	5.24	5.48	5.06
MEMO1	5.24	5.52	5.04
TMEM234	5.24	5.47	4.79
OTTHUMG00000163069	5.24	5.60	4.80
KCNK6	5.24	5.52	5.02
IGKV3-7	5.24	5.62	4.89
MIR187	5.24	6.07	4.66

CCDC85A	5.24	5.80	4.60
LOC401037	5.24	5.56	4.96
ZNF503	5.24	5.57	4.85
SVEP1	5.24	5.90	4.47
MIR662	5.24	5.72	4.93
AVL9	5.24	5.71	4.69
ELN	5.24	5.65	4.80
RNU6-50	5.24	5.71	4.81
ALDH1A1	5.24	7.18	3.89
SERINC4	5.24	5.47	4.67
KRTAP12-4	5.24	5.64	4.58
NDUFC2-KCTD14	5.24	5.42	4.77
LYPLA1	5.24	5.80	4.83
ZNHIT2	5.24	5.56	4.98
FLJ41423	5.24	5.63	4.84
USP31	5.24	5.39	5.08
KIAA0895L	5.24	5.39	4.98
HDGF	5.24	5.58	4.81
ATP9B	5.24	5.54	4.71
CIDECP	5.24	5.79	4.92
ANKRD20A3	5.24	5.47	4.85
ARL8B	5.24	5.88	4.41
LOC392621	5.24	5.90	4.87
DGKA	5.24	5.68	4.95
SLC20A2	5.24	5.57	4.56
CYP2B7P1	5.24	5.97	4.11
SLC19A2	5.24	5.92	4.23
YPEL1	5.24	5.53	4.66
ZNHIT1	5.24	5.54	4.96
PLEKHG3	5.24	5.48	4.85
OTTHUMG00000152058	5.24	5.56	4.93
SIRT3	5.24	5.58	4.84
CDIP1	5.24	5.71	4.85
DOM3Z	5.24	5.39	4.83
FAM216A	5.24	5.68	4.44
CC2D1B	5.24	5.77	4.83
MIR4725	5.24	5.93	4.55
MYL5	5.24	5.56	4.86
ADNP2	5.24	5.64	4.76
ANKRD33B	5.24	5.59	4.75
DAZAP1	5.24	5.76	4.58
ZSCAN25	5.24	5.78	4.66
PIGM	5.24	5.62	4.53
PCTP	5.24	5.49	4.89
CSRP2BP	5.24	5.56	4.89
OTTHUMG00000164137	5.24	5.69	4.81
ZNF181	5.24	5.60	4.73
MED20	5.24	5.54	4.87
SLC38A8	5.24	5.66	4.66

DVL2	5.24	5.69	4.84
TDRD7	5.24	5.63	4.78
OTTHUMG00000168366	5.24	5.72	4.15
ZMYM4-AS1	5.24	5.80	3.98
FAM122A	5.24	5.50	4.79
LRRC40	5.24	5.83	4.44
NSDHL	5.24	5.62	4.64
CPEB2	5.24	6.07	4.17
PELP1	5.24	5.62	4.65
MRGPRE	5.24	5.72	5.05
LINC00505	5.24	6.03	4.79
KIAA1407	5.23	5.50	4.96
WHSC1	5.23	5.77	4.79
GNA12	5.23	5.49	4.90
FHL2	5.23	6.04	4.74
KISS1	5.23	5.68	4.67
RABGGTA	5.23	5.60	4.90
GUSBP5	5.23	5.57	4.72
OTTHUMG00000040701	5.23	6.03	3.53
COQ6	5.23	5.57	4.51
TRANK1	5.23	5.77	4.97
SPON1	5.23	6.24	4.40
OTTHUMG00000164913	5.23	5.68	4.68
ZNF154	5.23	5.55	4.75
MYL6B	5.23	5.61	4.94
ISOC1	5.23	5.42	4.82
USP6NL	5.23	5.84	4.57
HFE	5.23	5.61	4.67
RTKN	5.23	5.51	4.88
XPO4	5.23	5.70	4.56
ZNF525	5.23	5.71	4.84
BATF2	5.23	5.94	4.87
AFF2	5.23	5.75	4.94
CHPF	5.23	5.62	4.75
RPL32	5.23	5.75	4.68
INTS7	5.23	5.74	4.56
TRMT10C	5.23	5.76	4.45
ME3	5.23	5.61	4.96
OTTHUMG00000151900	5.23	5.67	4.92
LOC101060198	5.23	6.04	4.39
CTSE	5.23	5.73	4.89
CLDN18	5.23	5.51	4.91
TTC28	5.23	5.65	4.51
TBC1D8B	5.23	5.83	4.73
LOC100128131	5.23	5.61	4.68
LOC100996344	5.23	5.68	4.78
SORL1	5.23	6.44	4.75
OTTHUMG00000155590	5.23	5.90	4.75
THAP11	5.23	5.62	4.71

URGCP	5.23	5.39	4.91
OTTHUMG00000019174	5.23	6.11	4.68
SLC27A1	5.23	5.61	4.95
GPR108	5.23	5.69	4.86
ZNF565	5.23	5.56	4.76
TLE2	5.23	5.69	4.74
ZHX1-C8ORF76	5.23	5.54	4.74
PLCD3	5.23	5.65	4.76
OTTHUMG00000171577	5.23	5.97	4.57
MAPK12	5.23	5.65	4.93
MOSPD2	5.23	5.85	4.57
NOL12	5.23	5.39	5.07
UMPS	5.23	5.64	4.69
CASP8	5.23	5.70	4.80
MBD3L2	5.23	5.89	4.83
SF3B2	5.23	5.52	4.79
IPO13	5.23	5.54	4.87
PRICKLE1	5.23	5.81	4.89
CEBPG	5.23	5.73	4.35
DAB2IP	5.23	5.64	4.96
RPS2P32	5.23	5.74	4.65
FANCA	5.23	5.47	5.08
PCDHB16	5.23	5.90	3.75
RPS14	5.23	5.44	4.76
TBX21	5.23	5.68	4.76
PLEKHA8	5.23	5.59	4.48
LOC100507373	5.23	5.51	4.81
DIABLO	5.23	5.52	4.79
DTX2	5.23	5.71	4.67
PDZK1IP1	5.23	5.66	4.88
CORO7-PAM16	5.23	5.67	4.74
ZNF558	5.23	5.73	4.91
ERGIC1	5.23	5.74	4.72
RABEP2	5.23	5.64	4.88
RNF187	5.23	5.59	4.89
AGPAT2	5.23	5.58	5.01
HIF1AN	5.23	5.72	4.67
GSPT2	5.23	5.61	4.77
TUBB3	5.23	6.02	4.46
FRAT2	5.23	5.54	4.88
SLC16A7	5.23	5.98	4.80
PIK3R5	5.23	5.63	4.95
AGPAT3	5.23	5.49	4.66
SLC36A4	5.23	5.72	4.56
OTTHUMG00000043709	5.23	5.58	4.89
CDK5	5.23	5.70	4.78
FAM131A	5.23	5.44	4.90
PFDN4	5.23	5.61	4.88
NAA20	5.23	5.82	4.47

EPHB3	5.23	5.84	4.74
ZNF630	5.23	5.64	4.65
OTTHUMG00000012131	5.22	5.63	4.34
C1orf226	5.22	5.67	4.87
SERPINB8	5.22	5.73	4.88
SNORD115-1	5.22	6.05	4.63
HOXC-AS1	5.22	5.69	4.80
SHROOM1	5.22	5.56	5.00
WDR86	5.22	5.64	4.92
FLJ42875	5.22	5.58	4.99
CADM1	5.22	6.26	3.90
TNRC6C-AS1	5.22	5.52	4.88
SHROOM4	5.22	5.65	4.86
SIMC1	5.22	5.55	4.94
CGNL1	5.22	5.63	4.94
PFKM	5.22	5.48	5.05
LY6G6E	5.22	5.67	4.60
SCARB1	5.22	5.63	4.78
LOC100288123	5.22	5.60	4.91
GALT	5.22	5.64	4.70
SNRPE	5.22	5.66	4.81
TP53BP2	5.22	5.76	4.66
SYNE3	5.22	5.41	5.00
GLI2	5.22	5.60	4.79
UCHL5	5.22	5.75	4.47
SMAD3	5.22	5.66	4.93
CLASRP	5.22	5.73	4.76
GGPS1	5.22	5.59	4.87
ZNF683	5.22	5.51	4.85
C17orf103	5.22	5.70	4.81
FAM35A	5.22	6.13	4.50
ING3	5.22	5.45	4.70
OR1M1	5.22	6.18	4.84
SSH3	5.22	5.60	4.99
MTRR	5.22	5.43	4.96
USP12	5.22	5.71	4.16
IGANRP	5.22	6.09	4.90
MTFMT	5.22	5.59	4.62
OTTHUMG00000168229	5.22	6.03	4.89
KRI1	5.22	5.54	4.79
MAP2K7	5.22	5.42	4.75
MIR3132	5.22	5.61	4.86
ANKMY2	5.22	5.89	4.45
CDCA5	5.22	5.63	4.72
TBRG4	5.22	5.49	4.72
LRRC29	5.22	5.98	4.77
SLC16A1	5.22	6.15	4.53
TFF3	5.22	5.83	4.96
PSD	5.22	5.61	4.79

NUCB2	5.22	5.57	4.90
LAMA5	5.22	5.57	4.92
ENTPD6	5.22	5.35	5.06
MIR30C1	5.22	5.73	4.73
ZNF716	5.22	5.79	4.59
SNAI3	5.22	5.58	4.84
RAVER2	5.22	5.73	4.77
ALG1L9P	5.22	5.58	4.83
OPN1MW2	5.22	5.65	4.80
POTEJ	5.22	5.72	4.65
GBP3	5.22	6.13	4.24
LOC643837	5.22	5.55	5.01
LOC100130111	5.22	5.60	4.75
LRRC46	5.22	5.59	4.80
LRRTM4	5.22	5.63	4.91
SLFNL1-AS1	5.22	5.53	4.98
GGTLC1	5.22	5.73	4.74
RRNAD1	5.22	5.45	5.06
ZNF740	5.22	5.43	4.95
MICALCL	5.22	5.50	4.85
NFKBIE	5.22	5.80	4.77
CA2	5.22	5.77	4.63
FRMD6	5.22	5.94	4.23
OTTHUMG00000002044	5.22	5.60	4.69
RNASE7	5.22	5.60	4.65
CPPED1	5.22	5.64	4.75
STGC3	5.22	5.65	4.75
CLN6	5.22	5.67	4.82
ATP9A	5.22	5.64	4.81
LOC285577	5.22	5.53	4.77
GLA	5.22	5.72	4.49
AMIGO1	5.22	5.45	4.87
LOC283887	5.22	5.71	4.98
ORAI2	5.22	5.68	4.84
MYRIP	5.22	5.59	4.87
MIR645	5.22	5.71	4.62
MAP3K6	5.22	5.53	4.95
SDSL	5.22	5.80	4.69
PSENEN	5.22	6.16	4.70
MSH2	5.22	5.69	4.77
SGTA	5.22	5.48	4.75
ZNF438	5.22	5.59	4.76
FAIM2	5.22	5.38	5.01
CCDC101	5.22	5.50	4.72
OTTHUMG00000153023	5.22	5.52	4.89
C12orf4	5.22	5.77	4.40
TBC1D10C	5.22	5.59	4.71
PMCHL2	5.22	5.67	4.75
OTTHUMG00000159376	5.21	5.77	4.82

COX18	5.21	5.50	4.95
ATP11A	5.21	5.51	4.96
CEP72	5.21	5.50	4.66
C19orf12	5.21	5.45	4.94
PRKX	5.21	6.21	4.52
LPAR2	5.21	5.51	4.87
SYT17	5.21	5.63	4.76
HCCS	5.21	5.82	4.25
GNL1	5.21	5.44	5.02
SLC2A6	5.21	5.77	4.71
HCG15	5.21	5.57	4.91
TLCD1	5.21	5.41	4.98
LEPR	5.21	5.79	4.90
CBLL1	5.21	5.58	4.88
TSHZ2	5.21	5.68	4.75
KRTAP9-4	5.21	5.69	4.84
TRIM26	5.21	5.49	4.93
TH	5.21	5.70	4.86
C1orf138	5.21	5.55	4.81
MSTO1	5.21	5.75	4.87
SNORA38	5.21	5.53	4.77
TTC38	5.21	5.40	4.98
ORAI1	5.21	5.59	4.56
PLA2G16	5.21	5.81	4.66
TFPI	5.21	5.85	4.22
MYBBP1A	5.21	5.61	4.92
EXOSC3	5.21	5.62	4.55
KRTCAP3	5.21	5.47	4.85
SEMA3F	5.21	5.44	4.85
LOC100505794	5.21	5.55	4.83
BPNT1	5.21	5.74	4.59
OTTHUMG00000164729	5.21	5.48	4.83
CDH24	5.21	5.63	4.37
RALGDS	5.21	5.82	4.89
GTF2F1	5.21	5.69	4.89
GK5	5.21	5.64	4.36
MATK	5.21	5.59	5.02
LRRC8A	5.21	5.57	4.91
WWC3	5.21	6.08	4.60
OTTHUMG00000164266	5.21	6.04	4.26
FAM188A	5.21	5.53	4.66
TCEAL4	5.21	5.37	5.02
FOXO6	5.21	5.55	5.02
RHBDF1	5.21	5.52	4.58
KDM6B	5.21	5.52	4.99
MCM9	5.21	5.38	4.80
IGLL5	5.21	5.80	4.82
ANKRD13B	5.21	5.50	4.86
RANBP1	5.21	5.52	4.79

FBP1	5.21	6.92	4.19
PCDHGB8P	5.21	6.08	4.74
PRR13	5.21	5.44	4.77
FAM193B	5.21	5.46	4.48
MED30	5.21	5.69	4.83
ZBTB17	5.21	5.63	4.74
COX17	5.21	5.55	4.68
RNASEH2B	5.21	5.56	4.80
GALM	5.21	5.95	4.10
MLLT3	5.21	5.81	4.79
ZNF641	5.21	5.58	4.68
GPC6-AS1	5.21	6.12	4.27
SBDSP1	5.21	5.44	4.81
USP38	5.21	5.66	4.89
CBWD6	5.21	5.58	4.86
PPP1R3F	5.21	5.61	4.93
SEC22C	5.21	5.45	4.91
GPR75	5.21	5.45	4.96
CLK2P	5.21	6.36	4.32
CAD	5.21	5.43	4.92
NOC3L	5.21	5.70	4.39
OTTHUMG00000151038	5.21	5.80	4.84
IGF1R	5.21	5.53	4.85
UBE2C	5.21	5.83	4.52
ARHGAP39	5.21	5.58	4.95
AQP11	5.21	5.46	4.89
YAF2	5.21	5.71	4.49
GPHN	5.21	5.49	4.62
SCAMP5	5.21	5.73	4.91
ZNF212	5.21	5.45	4.96
ATP4A	5.21	5.58	4.80
ARRB2	5.21	6.13	4.78
LSS	5.21	5.43	4.95
EMC2	5.20	5.80	4.47
GEMIN4	5.20	5.63	4.80
NCF1C	5.20	5.86	4.26
FAM170B	5.20	5.64	4.58
DFFB	5.20	5.35	4.99
PMVK	5.20	5.51	4.75
PRAMEF13	5.20	5.81	4.64
KIF23	5.20	5.78	4.64
PPM1F	5.20	5.61	4.92
FHOD1	5.20	5.76	4.85
OMP	5.20	5.58	4.85
UCK2	5.20	5.53	5.00
CNKSR2	5.20	5.78	4.46
SCD5	5.20	5.69	5.03
ITPK1	5.20	5.46	4.91
LMX1B	5.20	5.52	4.79

STARD5	5.20	5.59	4.55
GDF7	5.20	5.58	4.76
LOC400707	5.20	5.61	4.68
FRG1	5.20	5.74	4.54
LINC00864	5.20	5.57	4.96
BRMS1L	5.20	5.57	4.89
IFIT5	5.20	5.98	4.52
NDUFA6-AS1	5.20	5.54	4.95
OTTHUMG00000160551	5.20	5.65	4.89
PHLDA3	5.20	5.52	4.83
ST3GAL4	5.20	5.82	4.97
SV2A	5.20	5.59	4.55
CRELD1	5.20	5.79	4.92
CRYL1	5.20	5.79	4.37
PBLD	5.20	5.51	4.97
OTTHUMG00000162371	5.20	5.79	4.58
POLD1	5.20	5.60	4.81
GARNL3	5.20	5.48	4.79
LRP2BP	5.20	5.60	4.75
WDR89	5.20	5.53	4.70
C7orf43	5.20	5.47	4.84
COX6C	5.20	5.38	4.87
CDKN1A	5.20	5.83	4.72
NFS1	5.20	5.46	4.90
RAB33A	5.20	6.00	4.67
ZNF205-AS1	5.20	6.10	4.32
B3GNTL1	5.20	5.48	4.82
RP9	5.20	5.52	4.80
SOGA2	5.20	5.43	4.84
GDF15	5.20	5.51	4.85
TMEM114	5.20	5.75	4.83
ARHGAP32	5.20	6.00	4.77
ZNF577	5.20	5.71	4.74
ZNF596	5.20	5.65	4.77
C4orf46	5.20	5.62	4.91
PCSK4	5.20	5.49	4.98
CD300A	5.20	5.61	4.75
TPRA1	5.20	5.39	4.88
OASL	5.20	5.80	4.91
GPT	5.20	5.47	4.75
SPNS2	5.20	5.61	4.44
CCDC85B	5.20	5.92	4.81
UPF3B	5.20	5.70	4.74
SMOX	5.20	5.68	4.84
CPOX	5.20	5.69	4.64
LOC100507066	5.20	5.76	4.72
CERS6-AS1	5.20	5.76	4.22
FAM215B	5.20	5.77	4.49
OTTHUMG00000169240	5.20	5.99	4.65

FLJ13197	5.20	5.65	4.76
MTMR1	5.20	5.41	4.85
RNA5SP92	5.20	6.05	4.42
PCDHGA6	5.20	5.63	4.79
TRBV6-4	5.20	5.93	4.61
UBE2O	5.20	5.47	4.91
C1QL3	5.20	5.69	4.16
GDF5OS	5.20	5.89	4.75
DHX35	5.20	5.47	4.80
CXCL2	5.20	5.81	4.50
HCG14	5.20	5.63	4.58
DSCAML1	5.20	5.63	4.83
ACSM2B	5.20	5.74	4.81
SNORA27	5.20	5.62	4.62
PPP6R2	5.20	5.71	4.89
RBBP5	5.20	5.58	4.45
AFF3	5.20	5.55	4.63
HAPLN3	5.20	5.80	4.34
TSGA10IP	5.20	5.51	4.86
OGG1	5.20	5.45	4.90
KIF19	5.20	5.65	4.82
RAI1-AS1	5.20	5.42	4.80
RNU7-49P	5.20	5.76	4.25
SPDL1	5.20	6.09	4.53
PPOX	5.19	5.73	4.87
OTTHUMG00000159993	5.19	5.63	4.78
SPATA20	5.19	5.43	4.89
LRFN4	5.19	5.54	4.88
RNY4P17	5.19	6.17	3.18
OTTHUMG00000169072	5.19	5.55	4.38
LRRC14	5.19	5.47	4.93
PMM1	5.19	5.64	4.53
LEUTX	5.19	5.51	4.55
BAIAP2	5.19	5.44	4.80
PRORSD1P	5.19	5.45	4.81
WDR47	5.19	5.83	4.70
LINC00658	5.19	6.75	4.41
MROH5	5.19	5.69	4.74
SPNS3	5.19	5.51	4.44
CTSF	5.19	5.49	4.79
OTTHUMG00000153914	5.19	5.95	3.89
IPPK	5.19	5.57	4.78
NIP7	5.19	6.10	4.05
PCMT1	5.19	5.75	4.45
ENO2	5.19	6.03	4.32
THAP8	5.19	5.50	4.89
FITM1	5.19	5.67	4.73
BMF	5.19	5.48	4.72
OTTHUMG00000163567	5.19	5.72	4.78

PTPRF	5.19	5.39	4.90
KIAA0232	5.19	5.67	4.66
SAMD11	5.19	5.47	4.97
TANC1	5.19	5.65	4.86
DLGAP1-AS5	5.19	6.01	4.71
DNPEP	5.19	5.55	4.84
SLC27A4	5.19	5.68	4.53
CACNA1C	5.19	5.69	4.86
IRAK2	5.19	5.38	4.96
SLC38A7	5.19	5.48	4.87
NUCB1-AS1	5.19	5.45	4.95
SYNE2	5.19	5.77	4.90
OSBPL5	5.19	5.56	4.91
PITPNC1	5.19	5.70	4.75
ANKS1A	5.19	5.67	4.83
MRPL45P2	5.19	5.44	4.97
OTTHUMG00000020086	5.19	5.79	3.69
MFSD12	5.19	5.37	4.68
FBXO46	5.19	5.50	4.67
RNA5SP229	5.19	5.93	3.71
NLRC5	5.19	5.39	4.81
PHGDH	5.19	5.39	5.00
SYNJ2	5.19	5.79	4.41
RINL	5.19	5.70	4.81
PHYKPL	5.19	5.52	4.79
SCARNA9L	5.19	6.17	4.23
TRPV2	5.19	5.56	5.02
PVRL2	5.19	5.56	4.91
B3GAT2	5.19	5.54	4.67
ZIC1	5.19	5.95	4.54
LINC00350	5.19	5.69	4.80
ZNF217	5.19	5.55	4.76
IQCA1	5.19	5.90	4.54
YRDC	5.19	5.47	4.63
LINC00847	5.19	5.40	5.00
CMIP	5.19	5.99	4.82
OTTHUMG00000153690	5.19	5.61	4.90
HMGXB3	5.19	5.93	4.42
ASNS	5.19	5.61	4.79
CRADD	5.19	5.47	4.84
KCNS2	5.19	5.64	4.72
BST1	5.19	5.43	4.86
TPSD1	5.19	5.69	4.84
CCDC153	5.19	5.66	4.42
TAS2R4	5.19	5.42	4.75
TMEM204	5.19	5.91	4.60
RRP12	5.19	5.38	4.87
OR7E91P	5.19	5.68	4.42
RXRA	5.19	5.37	4.94

SUMO4	5.19	5.56	4.53
NMRAL1	5.19	5.69	4.56
TMEM9B-AS1	5.19	5.59	4.75
TBC1D7	5.19	5.59	4.69
TOM1L1	5.19	5.38	4.96
RBM12B	5.19	5.50	4.61
PPP2R5B	5.19	5.44	4.82
MAATS1	5.19	5.65	4.68
ASB7	5.19	5.68	4.21
TMEM199	5.18	5.40	4.98
BSDC1	5.18	5.52	4.62
TAP2	5.18	5.40	4.88
ELAC1	5.18	5.47	4.84
LOC100506374	5.18	5.76	4.71
LINC00883	5.18	5.50	5.01
PLK1	5.18	5.56	4.82
DLG3-AS1	5.18	5.72	4.60
AIPL1	5.18	5.65	4.81
LOC728065	5.18	5.70	4.85
IFT52	5.18	5.52	4.82
MBIP	5.18	5.61	4.78
MTMR4	5.18	5.63	4.90
OR2T11	5.18	5.83	4.65
RP9P	5.18	5.56	4.78
CAPN1	5.18	5.63	4.82
EHMT1-IT1	5.18	5.33	5.01
LRRC1	5.18	5.48	4.65
CYP21A2	5.18	5.51	4.96
NIPSNAP3A	5.18	5.69	4.51
SLC22A18	5.18	5.34	5.01
LOC285857	5.18	5.58	4.68
NGB	5.18	5.65	4.80
HMGCR	5.18	5.78	4.79
LRRC37A3	5.18	6.19	4.53
RFWD3	5.18	5.57	4.83
PIP5K1C	5.18	5.54	4.92
CDT1	5.18	5.39	4.93
LINC00840	5.18	5.77	4.62
NRM	5.18	5.44	4.59
PCCA	5.18	5.51	4.70
ACAP3	5.18	5.57	4.62
MIR34A	5.18	5.51	4.85
MST1P2	5.18	5.42	4.90
ATL1	5.18	5.49	4.70
DENND6B	5.18	5.68	4.84
FLJ42627	5.18	5.58	4.65
ITGB4	5.18	5.60	4.52
PLAC8L1	5.18	5.49	4.80
GPR27	5.18	5.53	4.64

ZNF826P	5.18	5.64	4.56
NADK2-AS1	5.18	5.72	4.65
OTTHUMG00000164200	5.18	5.42	4.78
LOC646938	5.18	5.49	4.91
CD82	5.18	5.62	4.72
SNORD111	5.18	6.17	4.08
RBPMS2	5.18	5.45	4.63
CBR3-AS1	5.18	6.00	4.69
ARMC7	5.18	5.76	4.94
TMEM215	5.18	5.39	4.87
BCRP2	5.18	5.50	4.72
SLC32A1	5.18	5.67	4.77
SNORA28	5.18	5.84	4.10
LACRT	5.18	5.76	4.83
XRRA1	5.18	5.57	4.71
ATG2A	5.18	5.37	4.97
UBE2J2	5.18	5.47	4.91
SNORD11	5.18	5.76	4.48
HNF4A	5.18	5.50	4.86
MAN1B1	5.18	5.38	4.99
PINX1	5.18	5.52	4.80
GRIK5	5.18	5.42	4.93
MRPL22	5.18	5.77	4.73
OTTHUMG00000164125	5.18	5.60	4.63
SYNJ2-IT1	5.18	6.16	4.76
LRP4	5.18	5.67	4.55
ZNF710	5.18	5.58	4.94
EMBP1	5.18	6.09	4.52
WDTC1	5.18	5.35	4.88
GCFC2	5.18	5.47	4.53
CCDC129	5.18	6.03	4.02
LOC100289019	5.18	5.44	4.89
FAM110A	5.18	5.33	4.60
ZNF346	5.18	5.45	4.37
LCE6A	5.18	5.81	4.67
BRSK2	5.18	5.67	4.84
ZBTB16	5.18	6.15	4.42
ZNF333	5.18	5.45	4.84
SNAP47	5.18	5.45	4.86
SSX4B	5.18	5.88	4.69
PTPRU	5.18	5.46	4.99
LOC100506934	5.18	5.84	4.87
FAHD1	5.17	5.56	4.74
TAF3	5.17	5.48	4.86
SEC22A	5.17	5.50	4.74
SERGEF	5.17	5.53	4.82
NPHP1	5.17	5.51	4.70
CKMT2	5.17	5.34	4.95
LOC155060	5.17	5.40	4.96

XAGE2	5.17	5.48	4.84
LOC100506557	5.17	5.71	4.48
OLFML1	5.17	5.70	4.72
LINC00467	5.17	5.47	4.86
CPSF3L	5.17	5.46	4.71
THOP1	5.17	5.64	4.62
PNMA6C	5.17	5.39	4.90
MTSS1L	5.17	5.78	4.59
EGLN3	5.17	5.68	4.76
DDX55	5.17	5.43	4.77
IGF2-AS	5.17	5.44	4.64
ESPN	5.17	5.54	4.62
CES2	5.17	5.55	4.74
GSTO2	5.17	5.55	4.42
TSNARE1	5.17	5.43	4.77
C2orf42	5.17	5.51	4.73
ZNF239	5.17	5.46	4.69
OTTHUMG00000151393	5.17	5.55	4.62
MRGBP	5.17	5.63	4.31
PRMT7	5.17	5.40	4.87
BZW2	5.17	5.56	4.77
LOC100132813	5.17	5.63	4.42
SNORD105B	5.17	5.84	4.59
OTTHUMG00000086303	5.17	5.61	4.81
GGH	5.17	5.64	4.66
RBBP8NL	5.17	5.69	4.77
WNT16	5.17	5.73	4.90
PKNOX2	5.17	5.64	4.53
ERI1	5.17	5.51	4.63
CNP	5.17	5.60	4.86
CADM4	5.17	5.55	4.73
OR10G3	5.17	6.00	4.57
KRT37	5.17	5.58	4.72
SNORD114-7	5.17	6.82	3.99
DEFA4	5.17	5.55	4.85
MAK16	5.17	5.71	4.73
FAM122C	5.17	5.40	4.95
PSMD9	5.17	5.69	4.64
LOC100129722	5.17	5.52	4.75
C2orf44	5.17	5.45	4.93
PHLPP2	5.17	5.59	4.78
INTS12	5.17	5.55	4.81
OTTHUMG00000178502	5.17	5.57	4.62
LINC00668	5.17	5.51	4.91
T	5.17	5.56	4.92
APH1A	5.17	5.53	4.71
DCLK1	5.17	6.03	4.36
BAP1	5.17	5.56	4.51
PKNOX1	5.17	5.68	4.69

PYCR2	5.17	5.52	4.82
OR2M3	5.17	6.03	4.62
RFNG	5.17	5.57	4.66
ARL4A	5.17	5.41	4.79
LARGE	5.17	5.50	4.76
EEF1A2	5.17	5.57	4.90
AP1S1	5.17	5.79	4.78
TBC1D9	5.17	5.67	4.34
ISCU	5.17	5.50	4.72
SYT12	5.17	5.78	4.71
LOC286437	5.17	5.62	4.36
EXOC3L1	5.17	5.56	4.86
LCE4A	5.17	5.59	4.79
ROBO2	5.17	5.76	4.83
OTTHUMG00000163595	5.17	5.78	4.49
DOC2GP	5.17	5.72	4.82
BRPF3	5.17	5.51	4.53
CCDC132	5.17	5.60	4.51
OTTHUMG00000168706	5.17	5.71	4.80
RFC3	5.17	5.43	4.74
LOC100216545	5.17	5.60	4.93
HOXC8	5.16	5.46	4.88
LOC100130547	5.16	5.55	4.70
RFX2	5.16	5.48	4.72
COMMD2	5.16	5.97	3.71
FLJ40288	5.16	5.66	4.42
MCM5	5.16	5.38	4.94
LOC100130887	5.16	5.52	4.66
PCDHB5	5.16	5.63	4.56
HCRT	5.16	5.52	4.75
C5orf38	5.16	5.54	4.82
ZSCAN21	5.16	5.55	4.58
PIGP	5.16	5.78	4.59
HOXD11	5.16	5.79	4.57
FN3K	5.16	5.37	4.93
ZSCAN32	5.16	5.52	4.89
ACBD5	5.16	5.70	4.63
CRY2	5.16	5.50	4.85
NANOS1	5.16	5.52	4.27
ZNF776	5.16	5.73	4.55
JMJD4	5.16	5.47	4.47
SMYD3	5.16	6.30	4.38
CD300E	5.16	5.73	4.62
QPCTL	5.16	5.46	4.88
DGKD	5.16	5.50	4.87
PARP8	5.16	5.54	4.55
PDZK1P1	5.16	5.47	4.90
AGBL5	5.16	5.49	4.83
HIP1R	5.16	5.51	4.93

RHOG	5.16	5.56	4.66
TACC3	5.16	5.53	4.65
FOSB	5.16	5.78	4.59
ZBTB41	5.16	6.09	4.11
COPS8	5.16	5.71	4.72
SALL4	5.16	5.55	4.85
MIR541	5.16	5.59	4.83
OTTHUMG00000020443	5.16	5.54	4.84
BTBD7	5.16	5.81	4.23
ATP13A1	5.16	5.41	4.94
MIR27A	5.16	5.76	4.92
TMEM79	5.16	5.32	5.00
LINC00630	5.16	5.83	4.58
HOXA10	5.16	5.34	4.97
TERC	5.16	5.70	4.45
SERPINE3	5.16	5.40	4.91
MBD1	5.16	5.76	4.76
CLDN15	5.16	5.37	4.87
EFNB1	5.16	5.52	4.72
LOC100271832	5.16	5.90	4.70
C5orf51	5.16	5.61	4.55
TMEM161A	5.16	5.32	4.98
OTTHUMG00000151344	5.16	5.55	4.81
ZFPL1	5.16	5.42	4.80
OTTHUMG00000158828	5.16	5.62	4.43
ARPC1B	5.16	5.68	4.55
CREBRF	5.16	5.80	4.53
COL7A1	5.16	5.73	4.70
ZNF576	5.16	5.40	4.91
LPP	5.16	5.90	4.00
PTHLH	5.16	6.42	3.97
FAM49B	5.16	5.76	4.88
BARHL2	5.16	5.72	4.79
SMARCD2	5.16	5.39	4.99
PTRH1	5.16	5.53	4.87
LOC100129434	5.16	6.16	4.14
EPOR	5.16	5.51	4.88
ASTE1	5.16	5.69	4.87
GTF2IRD1	5.16	5.45	4.86
GCHFR	5.16	5.45	5.01
KRT42P	5.16	5.54	4.76
RNU6-71P	5.16	5.75	3.89
MDGA1	5.16	5.48	4.85
BCL6B	5.16	5.71	4.38
C1orf170	5.16	5.53	4.65
CDCA7L	5.16	5.47	4.70
LOC100506746	5.16	5.35	4.98
TRAPPC12	5.16	5.61	4.79
SENP2	5.16	5.52	4.95

SF3B4	5.16	5.70	4.40
RGMA	5.16	5.45	4.67
HRSP12	5.16	5.78	4.62
ZNF75A	5.15	5.62	3.88
SNORD116-5	5.15	6.01	4.48
NCBP2	5.15	5.47	4.81
TRMT1	5.15	5.42	4.73
OTTHUMG00000165543	5.15	6.60	4.13
MRGPRG	5.15	5.55	4.87
PARP1	5.15	5.39	4.86
MIR139	5.15	5.50	4.85
ARHGEF39	5.15	5.44	4.87
SLC35A3	5.15	5.46	4.79
LOC100508120	5.15	5.61	4.64
OTTHUMG00000151895	5.15	5.55	4.54
LOC728613	5.15	5.55	4.57
MS4A8	5.15	5.54	4.71
C21orf62	5.15	5.70	4.73
C12orf65	5.15	5.68	4.81
FRMD3	5.15	5.57	4.50
ZNF205	5.15	5.88	4.68
LOC100509196	5.15	5.43	4.86
NUTM2G	5.15	5.49	4.88
NOP9	5.15	5.41	4.83
SSPN	5.15	5.50	4.89
ZNF234	5.15	5.47	4.45
ENGASE	5.15	5.45	4.83
LPHN1	5.15	5.45	4.84
SEMA4A	5.15	5.40	4.95
FKBP1B	5.15	5.52	4.72
TRAJ59	5.15	7.68	3.92
PYY2	5.15	5.92	4.75
LINC00340	5.15	5.96	4.35
SH3BP5L	5.15	5.56	4.64
LOC399829	5.15	5.61	4.72
LOC339240	5.15	5.63	4.85
SMIM12	5.15	5.57	4.82
RNY5P5	5.15	5.70	4.73
RNF220	5.15	5.61	4.75
ATP2A3	5.15	5.39	4.72
CKAP2	5.15	5.88	4.31
ABHD14B	5.15	5.41	4.91
CCDC134	5.15	5.42	4.83
CD36	5.15	6.49	3.60
NOP2	5.15	5.47	4.72
CTIF	5.15	5.53	4.73
NCKAP5L	5.15	5.57	4.72
TTC26	5.15	5.49	5.01
MYO1F	5.15	5.66	4.60

ZNF510	5.15	5.63	4.90
MIR3157	5.15	5.72	4.62
TTC12	5.15	5.44	4.91
SCLT1	5.15	5.55	4.55
IPO5	5.15	5.44	4.83
DTX4	5.15	5.44	4.85
GAL3ST3	5.15	5.68	4.87
C9orf16	5.15	5.40	5.00
CYP2A6	5.15	5.69	4.46
APCDD1L	5.15	5.81	4.72
ADAMTSL4	5.15	5.41	4.92
NDUFC1	5.15	5.55	4.78
ZDHHC2	5.15	5.38	4.78
C11orf57	5.15	5.56	4.74
TEAD4	5.15	5.51	4.69
SPATA1	5.15	5.62	4.66
OTTHUMG00000018570	5.15	5.55	4.65
RPL6	5.15	5.32	5.02
ZNF44	5.15	5.46	4.65
CD1A	5.15	6.03	4.44
IL4I1	5.15	5.42	4.89
CDR2L	5.15	5.78	4.71
LOC100128105	5.15	5.59	4.85
CNST	5.15	5.56	4.16
RPL10	5.15	5.52	4.46
PMPCA	5.15	5.33	4.51
MAPK6	5.15	5.58	4.65
FPGS	5.15	5.48	4.74
ARVCF	5.15	5.49	4.81
LOC440700	5.15	5.61	4.62
PRR21	5.15	5.71	4.41
TARM1	5.15	5.40	4.95
MIR3692	5.15	5.95	4.10
AZI2	5.15	5.50	4.85
LOC154449	5.15	5.41	4.71
ATPAF2	5.15	5.53	4.84
UBALD2	5.15	5.51	4.46
HS3ST1	5.15	5.35	4.79
OTTHUMG00000162503	5.15	5.53	4.85
IGFBP3	5.15	6.29	4.38
TRADD	5.15	5.50	4.82
RAD54B	5.15	5.45	4.60
PLCB1	5.15	5.85	4.45
LGI3	5.15	5.58	4.53
HSFX2	5.14	5.68	4.77
XKR8	5.14	5.63	4.71
MIR4290	5.14	5.75	4.69
NS3BP	5.14	5.67	4.69
ZER1	5.14	5.62	4.92

SLC29A2	5.14	5.68	4.64
LOC100996619	5.14	5.62	4.51
MBTD1	5.14	5.61	4.76
PIGG	5.14	5.53	4.77
LOC100507136	5.14	5.53	4.57
AHCYL2	5.14	5.39	4.46
GM140	5.14	6.04	4.31
SCGB1A1	5.14	5.47	4.65
RBM45	5.14	5.54	4.45
CDSN	5.14	5.65	4.79
SPATA31A4	5.14	5.37	4.70
ACYP1	5.14	5.59	4.33
C17orf107	5.14	5.39	4.92
LSM5	5.14	5.48	4.89
BAK1	5.14	5.51	4.36
ACTA2-AS1	5.14	5.58	4.69
LINC00346	5.14	5.54	4.75
GPR35	5.14	5.79	4.57
ZFYVE26	5.14	5.38	4.88
AGRN	5.14	5.56	4.79
PET117	5.14	5.59	4.73
ALKBH7	5.14	5.69	4.53
JOSD2	5.14	5.69	4.60
NEURL3	5.14	5.64	4.75
RNF152	5.14	5.62	4.67
SNAI1	5.14	5.53	4.53
NCAPG	5.14	5.72	4.66
TARS2	5.14	5.63	4.64
TAF1C	5.14	5.39	4.82
AXIN2	5.14	5.45	4.81
SOCS4	5.14	5.59	4.72
C9orf24	5.14	5.77	4.69
SENP8	5.14	5.46	4.72
EXOC2	5.14	5.58	4.50
TBX20	5.14	5.42	4.92
FAM60A	5.14	5.32	4.67
CXCL17	5.14	5.69	4.50
RNA5SP321	5.14	6.45	4.18
MIR521-1	5.14	5.77	4.57
FBRSL1	5.14	5.45	4.93
SPATA2L	5.14	5.59	4.59
CHMP7	5.14	5.74	4.61
CCNA2	5.14	5.87	4.47
NCMAP	5.14	5.68	4.70
PSMA1	5.14	5.51	4.68
RPUSD2	5.14	5.57	4.85
FHIT	5.14	5.67	4.87
ZIC5	5.14	5.65	4.69
NINL	5.14	5.52	4.71

NT5DC1	5.14	5.51	4.45
HLCS	5.14	5.64	4.84
SOX10	5.14	5.36	4.92
EXTL3-AS1	5.14	5.46	4.62
C11orf35	5.14	5.55	4.66
APC	5.14	5.48	4.90
TMEM59L	5.14	5.48	4.79
EIF4E	5.14	5.35	4.71
SPATA31A5	5.14	5.74	4.68
KCNJ12	5.14	5.63	4.83
NFASC	5.14	5.89	4.62
BLOC1S4	5.14	5.47	4.76
PARP12	5.14	5.39	4.71
PCDH9	5.14	5.92	4.07
PPP2CB	5.14	5.77	4.83
MASP2	5.14	5.32	4.91
TBC1D2	5.14	5.76	4.62
NOS1AP	5.14	5.62	4.68
CAPN12	5.14	5.30	4.97
SH2D6	5.14	5.44	4.83
OMA1	5.14	5.64	4.33
AIG1	5.14	5.78	4.52
CHAMP1	5.14	5.59	4.80
IFNAR2	5.14	5.55	4.64
OTTHUMG00000151209	5.14	5.64	4.77
CYTH2	5.14	5.43	4.75
CLUHP3	5.13	5.55	4.70
OTTHUMG00000151712	5.13	5.48	4.64
FBXO42	5.13	5.69	4.69
RIPPLY2	5.13	5.43	4.85
STK35	5.13	5.55	4.60
FAM76A	5.13	5.43	4.62
ALDH1B1	5.13	5.46	4.72
MIAT	5.13	5.38	4.81
LOC100507266	5.13	5.45	4.87
OTTHUMG00000015120	5.13	5.66	4.83
ARMCX3-AS1	5.13	5.71	4.55
RBP7	5.13	6.57	3.59
LOC100507548	5.13	5.60	4.51
SLC4A5	5.13	5.47	4.69
ZSCAN22	5.13	5.34	4.57
HIC2	5.13	5.39	4.65
FDXR	5.13	5.38	4.77
E4F1	5.13	5.40	4.77
C20orf24	5.13	5.30	4.85
KITLG	5.13	6.12	4.33
MMP21	5.13	5.43	4.64
RNF123	5.13	5.31	4.86
HSDL2	5.13	5.59	4.64

RGS17	5.13	5.47	4.62
ZNF777	5.13	5.40	4.69
PLP1	5.13	5.50	4.67
RRN3	5.13	5.44	4.61
PAGR1	5.13	5.40	4.71
TMEM138	5.13	5.52	4.66
DGKZ	5.13	5.43	4.84
LPIN1	5.13	5.32	4.88
NUTM2F	5.13	5.57	4.49
SH2D3C	5.13	5.42	4.77
IGLV4-3	5.13	5.76	4.80
KDM4A-AS1	5.13	5.56	4.87
RFX8	5.13	5.62	4.63
KRT17P1	5.13	5.88	4.26
VPS37C	5.13	5.45	4.79
LOC100506737	5.13	5.48	4.74
TMEM150A	5.13	5.56	4.73
RABIF	5.13	5.54	4.74
ZNF34	5.13	5.42	4.68
TTLL11	5.13	5.60	4.53
TRAJ23	5.13	5.72	4.41
OTTHUMG00000177236	5.13	5.67	4.79
SYN1	5.13	5.36	4.77
NADK	5.13	5.64	4.79
GALNT6	5.13	5.70	4.89
STK32C	5.13	5.58	4.77
LOC400684	5.13	5.72	4.37
NUDT7	5.13	5.61	4.40
MIR622	5.13	5.60	4.46
CTU2	5.13	5.51	4.85
OTTHUMG00000067435	5.13	6.38	3.78
PPP1R14B	5.13	5.37	4.77
LOC101060300	5.13	5.44	4.61
OTTHUMG00000171371	5.13	5.42	4.53
LOC440910	5.13	5.35	4.76
GIPC3	5.13	5.56	4.83
SMIM12-AS1	5.13	5.51	4.66
OLA1	5.13	5.61	4.46
LOC100128239	5.13	5.55	4.69
PUF60	5.13	5.56	4.67
HAVCR2	5.13	5.91	4.40
PPP1R3D	5.13	5.41	4.51
ZCCHC8	5.13	5.41	4.79
UCKL1	5.13	5.44	4.74
C14orf79	5.13	5.57	4.69
ARHGAP20	5.13	5.62	4.45
DRAP1	5.13	5.80	4.56
COL9A2	5.13	5.44	4.74
ANAPC15	5.13	5.50	4.76

HS3ST4	5.13	5.54	4.72
ROCK1P1	5.13	5.75	4.63
IFT57	5.13	5.54	4.63
AKR7A2P1	5.13	5.49	4.78
THUMPD1	5.13	5.46	4.50
LSM6	5.13	5.50	4.59
TMEM39B	5.13	5.39	4.75
MRPS31P5	5.13	5.74	4.58
LINC00453	5.13	5.44	4.77
OTTHUMG00000177740	5.13	5.78	4.57
RAB3D	5.13	5.64	3.42
CRELD2	5.13	5.47	4.65
OR10AD1	5.13	5.91	4.43
SNORD124	5.13	5.76	4.70
OTTHUMG00000178442	5.13	5.86	4.13
CCNJL	5.13	5.41	4.83
MYCBP	5.13	5.46	4.65
F11R	5.13	5.74	4.55
CDK5R1	5.13	5.39	4.78
ARHGEF2	5.13	5.36	4.87
ZNF189	5.12	5.44	4.52
RNA5SP255	5.12	6.24	3.95
CALML6	5.12	5.54	4.62
LOC100507144	5.12	5.95	4.71
MIR124-2	5.12	5.52	4.74
MIR7-3HG	5.12	5.84	4.57
OTTHUMG00000048097	5.12	5.63	4.39
TSPYL5	5.12	5.47	4.35
AXIN1	5.12	5.37	4.66
PTPN2	5.12	5.55	4.54
DENND1A	5.12	5.38	4.75
RANGAP1	5.12	5.62	4.73
FLJ44790	5.12	5.66	4.86
AP4M1	5.12	5.64	4.73
CPLX2	5.12	5.80	4.64
DAZL	5.12	5.78	4.68
KDM2B	5.12	5.35	4.96
MICAL3	5.12	5.44	4.70
OTTHUMG00000017631	5.12	5.79	4.67
C17orf76-AS1	5.12	5.46	4.80
OTTHUMG00000150049	5.12	5.69	4.78
NMUR1	5.12	5.75	4.58
C9orf156	5.12	5.50	4.72
CEP70	5.12	5.68	4.64
GDPD5	5.12	5.34	4.81
ADORA1	5.12	5.54	4.81
KBTBD6	5.12	5.80	4.85
TM6SF2	5.12	5.55	4.84
MGME1	5.12	5.67	4.44

RPL29	5.12	5.66	4.70
TSSK4	5.12	5.40	4.86
PCDH17	5.12	5.42	4.82
RAB9B	5.12	5.42	4.85
HTR2B	5.12	5.39	4.83
ASB8	5.12	5.46	4.80
TRIM53AP	5.12	5.58	4.61
DET1	5.12	5.41	4.81
LOC286370	5.12	5.60	4.82
SNX24	5.12	5.70	4.47
LMTK2	5.12	5.28	4.91
RGS19	5.12	5.42	4.90
HMGCL	5.12	5.37	4.66
FAM197Y9	5.12	6.62	2.79
SETDB2	5.12	5.39	4.81
METTL13	5.12	5.47	4.78
C8orf31	5.12	5.50	4.67
ZC3H7B	5.12	5.36	4.87
TLN2	5.12	5.28	5.04
RNF185	5.12	5.54	4.76
KLK14	5.12	5.35	4.63
PDCD1	5.12	5.72	4.62
APOC4-APOC2	5.12	5.51	4.75
ASIC3	5.12	5.77	4.77
RCAN1	5.12	6.16	4.37
LOC100133612	5.12	5.47	4.74
TCEB3	5.12	5.57	4.72
ZNF658B	5.12	5.41	4.85
QRICH2	5.12	5.57	4.78
ERVK13-1	5.12	5.61	4.43
UCK1	5.12	5.33	4.70
TMEM194A	5.12	5.62	4.68
CRIP2	5.12	5.48	4.68
TPCN1	5.12	5.67	4.83
CHST10	5.12	5.43	4.84
COL20A1	5.12	5.35	4.79
ZFYVE27	5.12	5.35	4.50
DAK	5.12	5.35	4.75
DNAJB2	5.12	5.51	4.81
EXD3	5.12	5.48	4.76
DAGLB	5.12	5.63	4.61
CDKN3	5.12	6.02	4.45
PTPN6	5.12	5.69	4.73
GTF2H3	5.12	5.64	4.81
HAUS5	5.12	5.36	5.00
BAIAP2L2	5.12	5.50	4.70
HMGA1P7	5.12	5.53	4.56
DEFB124	5.12	5.85	4.68
SNORD102	5.12	5.90	4.09

NOTCH3	5.12	5.55	4.80
SCGB3A1	5.12	5.42	4.83
HOXC10	5.12	5.43	4.76
USP13	5.12	5.42	4.89
IMP3	5.12	5.52	4.43
GTPBP6	5.12	5.58	4.55
LOC338799	5.12	5.53	4.88
TUBGCP6	5.12	5.47	4.84
FIG4	5.12	5.54	4.34
MIR4252	5.12	5.72	4.51
MRAP2	5.12	6.19	4.13
LOC400756	5.12	5.61	4.70
OTTHUMG00000162780	5.12	5.46	4.67
C2orf49	5.12	5.44	4.71
FLNB-AS1	5.12	5.41	4.69
OTTHUMG00000159304	5.11	5.50	4.55
PRAF2	5.11	5.48	4.63
UCHL1-AS1	5.11	5.83	4.36
SNORD116-28	5.11	5.48	4.53
ARSG	5.11	5.76	4.76
DPM2	5.11	5.76	4.22
SLC1A1	5.11	5.64	4.25
OTTHUMG00000168451	5.11	5.40	4.77
PRKCH	5.11	5.45	4.60
FAAH	5.11	5.53	4.81
PTPN23	5.11	5.49	4.84
ARHGEF19	5.11	5.40	4.80
MEGF6	5.11	5.56	4.92
CNDP2	5.11	5.38	4.68
RNA5SP360	5.11	5.89	3.75
ANKRD13D	5.11	5.31	4.77
SNRNP25	5.11	5.65	4.49
SYNGR3	5.11	5.29	4.70
LOC729164	5.11	5.35	4.83
ABHD17C	5.11	5.51	4.64
MGST3	5.11	5.56	4.52
TPX2	5.11	6.10	4.44
WDR24	5.11	5.42	4.70
WDR43	5.11	5.79	4.32
TMPPE	5.11	5.61	4.35
RGN	5.11	5.41	4.78
MIR4509-1	5.11	5.88	3.58
NTN1	5.11	5.53	4.63
RNA5SP122	5.11	5.54	4.61
PARP11	5.11	5.56	4.75
ZNF732	5.11	5.80	4.51
KRT85	5.11	5.77	4.66
ZBTB8B	5.11	5.49	4.89
UNC5CL	5.11	5.36	4.62

SNTA1	5.11	5.53	4.57
ZNF71	5.11	5.42	4.66
TBK1	5.11	5.57	4.32
SLC2A5	5.11	6.06	4.53
LIMK1	5.11	5.29	4.72
METTL1	5.11	5.38	4.43
OR8B12	5.11	5.75	4.47
RBM41	5.11	5.30	4.71
CCDC88B	5.11	5.68	4.66
FBXO44	5.11	5.68	4.83
CEP152	5.11	5.55	4.71
LOC339988	5.11	5.52	4.81
RNA5SP268	5.11	5.85	4.05
ETV2	5.11	5.54	4.95
OR52B6	5.11	5.83	4.34
RNY1P8	5.11	6.01	3.20
LPXN	5.11	5.67	4.64
SLC51A	5.11	5.40	4.84
TLE6	5.11	5.44	4.85
LOC100507634	5.11	5.84	4.68
VPS8	5.11	5.57	4.52
OTTHUMG00000156091	5.11	5.83	4.28
SLC6A3	5.11	5.52	4.83
MAGEC2	5.11	5.52	4.75
ACSF3	5.11	5.45	4.78
HOXC9	5.11	5.67	4.56
KCNQ1	5.11	5.44	4.72
MITF	5.11	5.61	4.49
CCDC71	5.11	5.50	4.71
CEP250	5.11	5.30	4.78
CD24	5.11	5.56	4.69
BRI3	5.11	5.40	4.65
FRMPD2P1	5.11	5.76	4.70
TAS2R5	5.11	5.68	4.26
LYRM1	5.11	5.67	4.58
SMIM7	5.11	5.42	4.83
ATXN7L3	5.11	5.40	4.70
TRAJ22	5.11	5.81	4.46
CENPJ	5.11	5.48	4.38
LINC00937	5.11	5.59	4.80
USP19	5.11	5.39	4.75
MIR191	5.11	5.55	4.00
OTTHUMG00000151374	5.11	5.38	4.93
DDX60L	5.11	5.63	4.80
OTTHUMG00000020831	5.11	5.60	4.72
MCTP1	5.11	5.87	4.18
ALDH6A1	5.10	5.41	4.67
PLXNC1	5.10	5.42	4.86
RP2	5.10	6.06	4.24

ZNF322	5.10	5.55	3.99
AUNIP	5.10	5.46	4.83
RAB33B	5.10	5.71	4.56
RFFL	5.10	5.55	4.75
TMEM231	5.10	5.63	4.56
FERD3L	5.10	5.51	4.73
NOS3	5.10	5.51	4.74
HIST1H3F	5.10	6.01	4.24
CLEC2B	5.10	6.06	4.60
PASK	5.10	5.58	4.77
KIAA1984	5.10	5.39	4.74
CRYM	5.10	5.54	4.65
ARSI	5.10	5.39	4.75
HAUS1	5.10	5.62	4.28
RALGPS1	5.10	5.46	4.71
MRRFP1	5.10	5.61	4.40
LRTM1	5.10	5.64	4.58
GPAT2	5.10	5.47	4.83
HAUS3	5.10	5.41	4.72
LOC100128262	5.10	5.48	4.92
FUT6	5.10	5.57	4.38
PRKRA	5.10	5.40	4.79
TVP23B	5.10	6.22	4.56
ORC4	5.10	5.42	4.60
DGCR14	5.10	5.30	4.84
TRIOBP	5.10	5.54	4.84
SNORD59A	5.10	6.44	3.80
ITGAX	5.10	5.61	4.67
SHF	5.10	5.30	4.83
MAP1LC3B	5.10	5.47	4.46
PFN4	5.10	5.41	4.91
TET3	5.10	6.08	4.64
TMEM216	5.10	5.44	4.62
VASN	5.10	5.35	4.85
LRRC42	5.10	5.53	4.41
LOC100130157	5.10	5.48	4.59
UNC45A	5.10	5.40	4.80
PRRT3-AS1	5.10	5.35	4.77
HOXA11	5.10	5.57	4.64
DDX20	5.10	5.27	4.63
ACVR1B	5.10	5.31	4.74
ATP2A1	5.10	5.64	4.55
SLC36A1	5.10	5.65	4.78
EVC2	5.10	5.64	4.73
KLHL2	5.10	5.49	4.73
GALNT8	5.10	5.93	4.05
PPM1J	5.10	5.69	4.72
OTTHUMG00000018553	5.10	5.64	4.54
ACADSB	5.10	5.52	4.69

OSER1	5.10	5.79	4.14
HDAC9	5.10	5.56	4.14
GFER	5.10	5.29	4.96
RBFA	5.10	5.42	4.81
LINC00485	5.10	5.62	4.54
GPRC5A	5.10	6.06	4.40
LOC728716	5.10	5.55	4.63
PITX2	5.10	5.52	4.68
ZNF208	5.10	5.47	4.44
C10orf68	5.10	5.64	4.77
OTTHUMG00000149337	5.10	6.08	4.14
CACNA1H	5.10	5.61	4.66
ZNF589	5.10	5.27	4.78
PLD1	5.10	5.45	4.65
KRTAP10-11	5.10	5.29	4.46
RHOBTB1	5.10	5.57	4.65
PET112	5.10	5.31	4.86
C6orf226	5.10	5.58	4.51
ALG10B	5.10	6.01	4.60
TUBA8	5.10	5.40	4.80
ACTR5	5.10	5.37	4.66
B3GNT9	5.10	5.52	4.70
WDR55	5.10	5.40	4.74
LETM2	5.10	5.34	4.83
RCCD1	5.10	5.67	4.86
C1orf111	5.10	5.54	4.63
LOC100129516	5.10	5.71	4.67
LRCOL1	5.10	5.47	4.67
SLC25A51	5.10	5.36	4.73
TRPM4	5.09	5.45	4.70
GALNT16	5.09	5.58	4.67
LPAR5	5.09	5.63	4.65
SLC22A12	5.09	5.34	4.60
PSRC1	5.09	5.58	4.61
AIM1	5.09	5.82	4.12
OTTHUMG00000179148	5.09	5.59	4.79
PGPEP1	5.09	5.46	4.70
ZFP41	5.09	5.42	4.73
MIER2	5.09	5.56	4.16
OTTHUMG00000169585	5.09	5.98	4.59
ANAPC1	5.09	5.68	4.48
NANOS3	5.09	5.41	4.89
LOC100144597	5.09	5.34	4.75
ERMP1	5.09	5.54	4.82
RGMB	5.09	5.43	4.65
PTDSS1	5.09	5.87	3.99
WSCD2	5.09	5.52	4.65
FAM92A1	5.09	5.59	4.65
ACP2	5.09	5.50	4.73

GAS2L1	5.09	5.38	4.82
NDUFS7	5.09	5.29	4.92
CTDP1	5.09	5.29	4.87
OTTHUMG00000166117	5.09	5.69	4.13
ADAMTS4	5.09	5.48	4.81
SET	5.09	5.45	4.57
RNA5SP425	5.09	5.68	4.32
NOP16	5.09	5.77	4.70
HOXB3	5.09	5.29	4.93
FBXW5	5.09	5.62	4.69
IRAK4	5.09	5.59	4.38
YARS2	5.09	5.57	3.97
RPS6KA1	5.09	5.50	4.80
SSPO	5.09	5.55	4.83
PLLP	5.09	5.59	4.72
APOLD1	5.09	5.84	4.76
CSTF1	5.09	5.50	4.71
PRICKLE2-AS1	5.09	5.53	4.80
GHRL	5.09	5.49	4.71
POFUT2	5.09	5.38	4.76
EMILIN2	5.09	5.53	4.80
TMPO-AS1	5.09	5.45	4.76
FAHD2CP	5.09	5.40	4.44
SHISA3	5.09	5.62	4.75
RFT1	5.09	5.64	4.66
TANGO6	5.09	5.57	4.59
DLEU2L	5.09	5.76	4.47
CCDC113	5.09	5.48	4.69
SCRN3	5.09	5.51	4.57
ZNF432	5.09	5.49	4.30
CACNA2D1	5.09	5.53	4.37
PUS10	5.09	5.30	4.78
HSD11B2	5.09	5.58	4.55
PLEKHM3	5.09	5.55	4.66
HN1L	5.09	5.45	4.44
CBX2	5.09	5.40	4.62
SLC23A3	5.09	5.37	4.57
C1orf51	5.09	5.46	4.80
ROBO3	5.09	5.45	4.88
RCAN3	5.09	5.68	4.14
LGALS8-AS1	5.09	5.62	4.60
AKAP6	5.09	5.53	4.39
CX3CR1	5.09	5.48	4.68
CT45A5	5.09	5.97	4.16
GTF2H4	5.09	5.63	4.46
ZNF493	5.09	5.96	4.73
ZMIZ1-AS1	5.09	5.71	4.82
ZNF789	5.09	5.47	4.53
MC1R	5.09	5.31	4.75

C9orf47	5.09	5.45	4.70
MIR4269	5.09	5.70	4.54
ZFYVE20	5.09	5.59	4.67
NCAN	5.09	5.70	4.73
MIR1200	5.09	5.48	4.45
MRPL10	5.09	5.44	4.76
IP6K1	5.09	5.43	4.29
TUSC1	5.09	5.27	4.81
STK10	5.09	5.54	4.76
GPT2	5.08	5.51	4.75
SCLY	5.08	5.38	4.84
MPP3	5.08	5.57	4.70
LILRA4	5.08	5.40	4.89
SPHK2	5.08	5.39	4.86
GPAM	5.08	5.32	4.89
ZBTB34	5.08	5.51	4.34
HINT3	5.08	5.57	4.56
ZNF664-FAM101A	5.08	5.29	4.88
C10orf25	5.08	5.52	4.61
PTTG2	5.08	5.59	4.68
DCAF4	5.08	5.61	4.57
TRMT1L	5.08	5.61	4.54
PHF7	5.08	5.18	4.85
KCTD1	5.08	5.39	4.84
TTLL10	5.08	5.51	4.85
KIAA0226	5.08	5.48	4.82
MYRF	5.08	5.35	4.79
TMEM45B	5.08	5.49	4.57
ABHD4	5.08	5.79	4.13
TMCC3	5.08	5.51	4.79
LRRC37A	5.08	5.66	4.48
RNA5SP32	5.08	5.91	4.64
MIR143	5.08	5.63	4.65
PPIH	5.08	5.51	4.58
PAX2	5.08	5.62	4.60
CD3EAP	5.08	5.44	4.78
WARS2	5.08	5.35	4.54
OTTHUMG00000086635	5.08	5.53	4.76
LRRC27	5.08	5.33	4.90
TRMT2B	5.08	5.40	4.85
LOC100129931	5.08	5.35	4.78
LOC728228	5.08	5.78	4.60
LRCH4	5.08	5.41	4.80
TECPR2	5.08	5.35	4.75
MIR503	5.08	5.58	4.68
LIPE	5.08	5.50	4.77
MIR2278	5.08	5.46	4.80
TMEM70	5.08	5.39	4.85
JRK	5.08	5.44	4.72

GNAI1	5.08	5.34	4.62
RAB11B-AS1	5.08	5.51	4.42
TEPP	5.08	5.36	4.64
OSGEPL1-AS1	5.08	5.53	4.37
PHLDB2	5.08	5.62	4.47
SNORD114-12	5.08	7.56	3.15
VSIG10	5.08	5.40	4.77
MYEF2	5.08	5.50	4.67
PLCD4	5.08	5.52	4.80
KAZALD1	5.08	5.44	4.68
KLK1	5.08	5.45	4.84
FANCG	5.08	5.23	4.92
TCEANC2	5.08	5.43	4.84
LPHN2	5.08	5.93	4.36
DPH3P1	5.08	5.71	4.47
JMJD1C-AS1	5.08	5.42	4.57
ATP5S	5.08	5.45	4.46
TXNRD1	5.08	5.37	4.72
SLC25A18	5.08	5.54	4.74
STARD4	5.08	5.70	4.61
GYPE	5.08	5.57	4.34
IRGQ	5.08	5.41	4.80
TRIM24	5.08	5.34	4.57
SLC34A1	5.08	5.40	4.62
KIN	5.08	5.42	4.75
ITGB3BP	5.08	5.51	4.54
COL18A1	5.08	5.59	4.41
ZBTB22	5.08	5.32	4.73
PEX14	5.08	5.36	4.82
CYHR1	5.08	5.38	4.70
LOC100505501	5.08	5.68	4.38
SNORD15B	5.08	5.62	4.45
LOC100507602	5.08	5.51	4.31
ARL4D	5.08	5.63	4.45
DNALI1	5.08	5.50	4.79
ST6GALNAC4	5.08	5.57	4.56
LOC100129831	5.08	5.26	4.86
NMNAT2	5.08	5.47	4.74
PUSL1	5.08	5.30	4.90
DHDH	5.08	5.59	4.61
PTPRK	5.08	5.76	4.11
LINC00568	5.08	5.44	4.62
BMP4	5.08	5.62	4.76
GEMIN6	5.08	5.49	4.72
RNF144B	5.08	5.70	4.48
KCTD15	5.08	5.34	4.51
PRDM8	5.08	5.75	4.52
ACOX3	5.08	5.40	4.77
COMT	5.08	5.46	4.87

LAGE3	5.08	5.71	4.70
C1orf56	5.08	5.53	4.81
DTL	5.08	5.67	4.58
KIAA1147	5.08	5.51	4.79
DTD2	5.07	5.44	4.72
CPE	5.07	5.94	3.47
UTF1	5.07	5.34	4.87
GPR3	5.07	5.66	4.64
LOC283140	5.07	5.36	4.59
USP18	5.07	5.46	4.47
MRPL9	5.07	5.74	4.64
GAL3ST2	5.07	5.31	4.45
CCDC9	5.07	5.59	4.72
ZNF517	5.07	5.55	4.70
KRTAP2-3	5.07	5.89	4.75
RAP1B	5.07	5.74	4.23
NCF1B	5.07	5.83	4.38
GPR42	5.07	6.23	4.35
MIR125A	5.07	5.64	4.74
LOC100131826	5.07	5.44	4.20
IDH3A	5.07	5.57	4.67
PRADC1	5.07	5.48	4.80
THAP9-AS1	5.07	5.45	4.69
PDE1A	5.07	5.68	4.58
SLC24A1	5.07	5.27	4.79
MAST4	5.07	5.46	4.78
APOA2	5.07	5.37	4.71
OTTHUMG00000152702	5.07	5.47	4.72
PTPRC	5.07	6.59	4.00
KBTBD4	5.07	5.31	4.77
MIR1245A	5.07	6.93	3.40
OLFM1	5.07	5.42	4.68
RTBDN	5.07	5.42	4.70
IGLON5	5.07	5.53	4.76
PDE3B	5.07	5.67	4.29
FGF14-AS2	5.07	5.41	4.74
STK32A	5.07	6.21	3.88
FOXRED1	5.07	6.04	4.63
COL24A1	5.07	5.61	4.47
IDNK	5.07	5.49	4.72
RNF44	5.07	5.33	4.58
DTWD2	5.07	5.54	4.59
EFCAB7	5.07	5.76	4.41
FOXD2-AS1	5.07	5.38	4.77
FAM211B	5.07	5.50	4.62
TRAV8-7	5.07	5.60	4.41
FAM131B	5.07	5.48	4.52
CASP9	5.07	5.27	4.68
PDXP	5.07	5.57	4.77

LMF1	5.07	5.43	4.60
TRIM17	5.07	5.27	4.69
ACRBP	5.07	5.63	4.80
ADAM1A	5.07	5.35	4.72
CCNB2	5.07	5.58	4.69
GABARAPL1	5.07	5.36	4.65
PLEKHH3	5.07	5.33	4.89
FZD10-AS1	5.07	5.64	4.73
SECTM1	5.07	5.36	4.74
BYSL	5.07	5.33	4.65
OTTHUMG00000156136	5.07	5.83	4.31
DLGAP5	5.07	6.06	4.58
SLC25A45	5.07	5.28	4.79
DENND5B	5.07	5.39	4.34
ITGB1BP1	5.07	5.64	4.41
YKT6	5.07	5.58	4.66
LOC100653000	5.07	5.30	4.78
PDIA2	5.07	5.72	4.64
LRSAM1	5.07	5.36	4.85
TUSC3	5.07	5.63	4.39
SYT8	5.07	5.28	4.54
GSG1L	5.07	5.22	4.92
ITPRIP	5.07	5.44	4.46
ZNF423	5.07	5.59	4.45
C6orf1	5.07	5.34	4.67
HPCA	5.07	5.59	4.57
FLJ38668	5.07	5.48	4.12
SPPL2B	5.07	5.49	4.76
C11orf83	5.07	5.28	4.66
HMGN1	5.07	5.43	4.33
TTC28-AS1	5.07	5.28	4.87
C17orf100	5.07	5.45	4.73
LINC00640	5.07	5.38	4.66
TSTA3	5.07	5.50	4.74
DHFRL1	5.07	5.43	4.61
HKR1	5.06	5.73	4.55
KIAA1683	5.06	5.36	4.66
HCAR3	5.06	5.76	4.47
FLJ40448	5.06	5.82	4.18
TRUB2	5.06	5.32	4.75
CDK5RAP1	5.06	5.48	4.34
LHX2	5.06	5.66	4.82
LOC284379	5.06	5.61	4.63
SMIM11	5.06	5.54	4.68
C8orf58	5.06	5.34	4.69
CALY	5.06	5.46	4.61
ARID3B	5.06	5.40	4.74
OTTHUMG00000156131	5.06	5.96	4.55
CALM1	5.06	5.33	4.53

C20orf112	5.06	5.33	4.91
NECAB3	5.06	5.45	4.70
CCDC97	5.06	5.33	4.32
OR3A4P	5.06	5.50	4.73
NHSL2	5.06	5.55	4.43
SLC30A4	5.06	5.61	4.46
AGTR1	5.06	5.56	4.41
UBE2D4	5.06	5.56	4.43
FAM110D	5.06	5.62	4.27
ARSB	5.06	5.69	4.41
DUSP27	5.06	5.44	4.71
C4orf21	5.06	5.37	4.66
FEM1A	5.06	5.27	4.80
SRRT	5.06	5.50	4.48
LOC100289090	5.06	5.58	4.54
OTTHUMG00000162193	5.06	5.44	4.48
RAP1A	5.06	5.61	4.64
DOK3	5.06	5.23	4.80
NEXN	5.06	5.98	4.43
ELF4	5.06	5.49	4.67
OTTHUMG00000168346	5.06	5.53	4.57
PPP1R32	5.06	5.31	4.79
OTTHUMG00000018322	5.06	5.49	4.57
CLEC16A	5.06	5.41	4.69
NAB2	5.06	5.76	4.50
FASN	5.06	5.30	4.91
FEN1	5.06	5.33	4.68
LINC00884	5.06	5.62	4.59
BAX	5.06	5.58	4.39
SLC6A16	5.06	5.91	4.64
PELI3	5.06	5.35	4.95
OTP	5.06	5.37	4.76
TRERF1	5.06	5.25	4.85
LZTR1	5.06	5.32	4.60
ACACB	5.06	5.44	4.61
LOC100131320	5.06	5.63	4.32
LOC729041	5.06	5.34	4.51
KLHL21	5.06	5.28	4.80
RHBDL1	5.06	5.49	4.61
POLR2F	5.06	5.34	4.85
RNMT	5.06	5.58	4.54
ZNF662	5.06	5.27	4.73
CHKA	5.06	5.50	4.43
TADA2B	5.06	5.35	4.69
OTTHUMG00000169402	5.06	5.51	4.60
GTF2H2C	5.06	5.43	4.39
ZNF271	5.06	5.75	4.04
DPP9	5.06	5.40	4.61
OTTHUMG00000180449	5.06	5.59	4.78

TLR3	5.06	5.69	4.48
S100A14	5.06	5.62	4.78
ZNF696	5.06	5.43	4.87
C17orf59	5.06	5.37	4.69
C21orf67	5.06	5.36	4.63
SNHG15	5.06	5.49	4.69
ASAP1-IT1	5.06	5.34	4.59
SLC22A23	5.06	5.32	4.51
SMIM17	5.06	5.57	4.51
RNASEH2A	5.06	5.70	4.60
OTTHUMG00000163077	5.06	5.36	4.80
TTTY14	5.06	5.88	3.87
EPHX2	5.06	5.45	4.50
ERO1LB	5.06	5.76	4.16
RDH8	5.06	5.33	4.78
MYBL2	5.06	5.42	4.74
RNF223	5.06	5.26	4.66
TDRKH	5.06	5.37	4.63
SLC52A2	5.06	5.79	4.56
IGSF8	5.06	5.36	4.68
PPP2R2A	5.06	5.29	4.72
HNF1A	5.06	5.47	4.67
INTS8	5.06	5.33	4.45
EDA2R	5.06	5.60	4.71
KCTD17	5.06	5.45	4.78
LOC100131347	5.06	5.87	4.47
C1QTNF9B-AS1	5.06	5.32	4.64
SRCIN1	5.06	5.32	4.76
LOC100506115	5.06	5.48	4.62
APCDD1	5.06	5.63	4.53
PRSS3	5.06	5.61	4.50
RAB6B	5.06	5.64	4.75
DMAP1	5.06	5.49	4.47
FAM189A2	5.06	5.36	4.78
PTPN3	5.05	5.46	4.47
STARD3NL	5.05	5.47	4.68
RNF138	5.05	5.55	4.34
CHM	5.05	5.63	4.39
CPM	5.05	6.17	3.97
LOC442075	5.05	5.43	4.49
VMO1	5.05	5.97	4.75
SLC6A12	5.05	5.58	4.73
RAMP2-AS1	5.05	5.51	4.60
LRFN3	5.05	5.67	4.58
LSM1	5.05	5.85	4.33
HOXC-AS2	5.05	5.26	4.65
LOC284757	5.05	5.74	4.56
UBE2T	5.05	5.91	4.36
SHANK2-AS3	5.05	5.50	4.52

ABLIM3	5.05	5.31	4.61
EMX1	5.05	5.27	4.71
ACTA1	5.05	5.63	4.41
CEP128	5.05	5.45	4.57
BTG1	5.05	5.68	4.65
DHX30	5.05	5.33	4.87
ZBTB3	5.05	5.66	4.32
OTTHUMG00000172617	5.05	5.59	4.11
CNNM4	5.05	5.43	4.71
C1orf145	5.05	5.69	4.76
TMEM52	5.05	5.53	4.79
PTPLA	5.05	5.36	4.77
SLC4A4	5.05	5.66	4.41
OTTHUMG00000179955	5.05	5.61	4.23
SPRR1A	5.05	5.95	4.70
GOLGA6L6	5.05	5.67	4.37
PIF1	5.05	5.47	4.82
ZNRF3-IT1	5.05	5.65	4.43
DPH5	5.05	5.66	4.63
LOC285547	5.05	5.32	4.69
NOSTRIN	5.05	5.71	4.25
INPP1	5.05	5.44	4.71
SNORA36B	5.05	5.84	4.61
LOC100133669	5.05	5.71	4.47
ZSCAN18	5.05	5.25	4.71
FAM228B	5.05	5.46	4.46
FAM90A27P	5.05	5.57	4.15
MID1IP1	5.05	5.29	4.58
LINC00484	5.05	5.45	4.41
OR52E2	5.05	5.67	4.66
RNA5SP326	5.05	5.51	4.46
ZNF610	5.05	5.50	4.63
MFSD3	5.05	5.50	4.50
KCNJ13	5.05	5.33	4.69
ADAL	5.05	5.68	4.46
DEPDC5	5.05	5.22	4.80
TMEM63C	5.05	5.42	4.75
KDM1B	5.05	5.44	4.84
SRRD	5.05	5.52	4.46
ESRRB	5.05	5.28	4.86
FAM110B	5.05	5.31	4.74
LMO2	5.05	5.25	4.68
LOH12CR1	5.05	5.44	4.76
C11orf74	5.05	5.54	4.48
LINC00434	5.05	5.68	4.55
KRT83	5.05	5.28	4.40
ANO8	5.05	5.46	4.66
OTTHUMG00000173283	5.05	5.38	4.72
PLAT	5.05	5.69	4.56

TMEM37	5.05	5.36	4.58
FAM92B	5.05	5.68	4.45
FOXB2	5.05	5.76	4.53
TP53I11	5.05	5.41	4.83
FOLR4	5.05	5.73	4.43
MPL	5.05	5.33	4.66
GMPR	5.05	5.50	4.70
PTK7	5.05	5.27	4.68
MIR877	5.05	5.51	4.48
POP5	5.05	5.31	4.60
NFKB2	5.05	5.30	4.91
FKRP	5.05	5.31	4.70
IGLJ4	5.05	6.00	4.14
PEG3	5.05	5.51	4.61
GSTZ1	5.05	5.33	4.75
MPRIP-AS1	5.05	5.56	4.46
GJA9-MYCBP	5.05	5.43	4.37
TPRG1-AS1	5.05	5.46	4.66
ZNF175	5.05	5.36	4.61
EDEM1	5.05	5.61	4.48
FPGT-TNNI3K	5.05	5.28	4.84
ARL6IP6	5.05	5.36	4.59
BTBD9	5.05	5.43	4.76
CEP76	5.04	5.54	4.60
SIGLEC11	5.04	5.49	4.24
HOXA7	5.04	5.23	4.74
SH3PXD2A	5.04	5.26	4.85
LOC100506060	5.04	5.46	4.71
RPS7	5.04	5.26	4.88
RNU7-52P	5.04	5.51	4.55
SLC27A5	5.04	5.24	4.91
OTTHUMG00000168151	5.04	5.41	4.82
PLEKHG5	5.04	5.34	4.77
KCNN3	5.04	5.41	4.62
RRAGC	5.04	5.83	4.41
MIR218-1	5.04	5.83	4.35
FGR	5.04	5.41	4.68
PDZRN3	5.04	5.31	4.77
SFXN2	5.04	5.51	4.52
CAMK2N2	5.04	5.38	4.61
AP3S1	5.04	5.40	4.54
RCE1	5.04	5.29	4.55
LOC100133746	5.04	5.67	4.51
STRIP1	5.04	5.30	4.75
TTC8	5.04	5.52	4.32
SLA	5.04	5.39	4.76
RGL3	5.04	5.35	4.51
ZBTB7B	5.04	5.20	4.76
PKDCC	5.04	5.23	4.76

C4orf6	5.04	5.65	4.70
STL	5.04	5.58	4.35
TEX22	5.04	5.47	4.87
MKRN9P	5.04	5.94	4.59
HOMER1	5.04	5.90	4.23
MTUS2-AS1	5.04	5.50	4.45
BCR	5.04	5.36	4.49
UNKL	5.04	5.40	4.66
APBB3	5.04	5.39	4.79
LOC650368	5.04	5.44	4.72
PAQR7	5.04	5.36	4.79
MKI67	5.04	5.59	4.57
OTTHUMG00000165339	5.04	5.38	4.73
RPIA	5.04	5.29	4.70
SUPT3H	5.04	5.49	4.59
THOC3	5.04	5.60	4.68
HMGB3	5.04	5.32	4.84
IMPA1	5.04	5.51	4.53
CENPF	5.04	5.92	4.21
NADSYN1	5.04	5.34	4.38
GRAMD4	5.04	5.47	4.49
LOC100130698	5.04	5.29	4.84
HINFP	5.04	5.40	4.73
APOBEC2	5.04	5.44	4.70
BAHD1	5.04	5.44	4.25
MAGOH2	5.04	5.28	4.76
SLC41A3	5.04	5.41	4.75
JARID2	5.04	5.39	4.47
SOLH	5.04	5.26	4.79
IDUA	5.04	5.36	4.42
NEK7	5.04	5.57	4.44
ACTL8	5.04	5.60	4.78
GNG3	5.04	5.56	4.70
SPTY2D1	5.04	5.28	4.60
ZNF516	5.04	5.40	4.85
ZNF584	5.04	5.41	4.80
S1PR1	5.04	5.58	4.30
OTTHUMG00000032375	5.04	5.70	4.64
TEX33	5.04	5.62	4.62
IER5L	5.04	5.47	4.76
PRKCE	5.04	5.30	4.84
PRRT1	5.04	5.25	4.85
MGLL	5.04	5.35	4.46
DLG4	5.04	5.38	4.55
ZNF829	5.04	5.57	4.75
WNT10B	5.04	5.35	4.69
SLED1	5.04	6.17	3.93
LOC96610	5.04	5.69	3.83
RAPGEF3	5.04	5.43	4.65

GLIPR1L2	5.04	5.70	4.63
PRR25	5.04	5.63	4.60
FOLR1	5.04	5.50	4.45
RNU1-14P	5.04	5.83	4.11
C18orf25	5.04	5.40	4.54
KAT8	5.04	5.18	4.85
LYSMD3	5.04	5.75	4.47
ZBTB43	5.04	5.52	4.69
SEC14L6	5.04	5.56	4.43
INSRR	5.04	5.59	4.69
TLL1	5.04	5.48	4.46
CEP44	5.04	5.69	4.59
LOC100507330	5.04	5.69	4.51
CCT6P1	5.04	5.44	4.79
INPP5D	5.04	5.41	4.57
GPR56	5.04	5.65	4.14
OTTHUMG00000151833	5.04	5.44	4.35
ORAI3	5.04	5.21	4.65
HSP90AB4P	5.04	5.45	4.52
POLN	5.04	5.40	4.74
LINS	5.04	5.58	4.64
SIGLEC7	5.04	5.67	4.29
LOC100288144	5.03	5.62	4.40
OTTHUMG00000159297	5.03	5.54	4.49
RMI1	5.03	5.45	4.69
FCRL6	5.03	5.51	4.68
OTTHUMG00000015822	5.03	5.87	4.43
FZD10	5.03	5.33	4.32
BCDIN3D	5.03	5.26	4.67
C4orf3	5.03	5.29	4.64
LMBRD2	5.03	5.51	4.36
DPYS	5.03	5.35	4.46
VWA2	5.03	5.20	4.90
IL1RAP	5.03	5.36	4.76
HES4	5.03	5.65	4.56
C1orf174	5.03	5.43	4.54
DDX11-AS1	5.03	5.39	4.61
MICALL2	5.03	5.34	4.69
GALP	5.03	5.42	4.63
TIMM17B	5.03	5.28	4.58
KIAA0247	5.03	5.40	4.31
MUC5AC	5.03	5.26	4.80
EGFLAM-AS1	5.03	5.75	4.34
EIF5A	5.03	5.37	4.75
FFAR4	5.03	5.75	4.28
TFB1M	5.03	5.71	4.66
LOC100508781	5.03	6.06	3.85
LOC729305	5.03	5.39	4.62
C11orf95	5.03	5.51	4.78

ZFP14	5.03	5.58	4.46
GNAL	5.03	5.59	4.67
NOV	5.03	6.02	4.09
RNU5E-9P	5.03	5.58	3.81
OTTHUMG00000163757	5.03	5.52	4.43
PPP4R1L	5.03	5.37	4.53
BRIX1	5.03	5.44	4.35
LOC390660	5.03	5.60	4.44
LOC100505683	5.03	5.34	4.68
PRCP	5.03	5.36	4.67
CHUK	5.03	5.71	4.45
KIAA1737	5.03	5.30	4.75
KLK3	5.03	5.71	4.65
FOXE1	5.03	5.47	4.64
MAGEB16	5.03	5.82	4.52
POLR2K	5.03	5.47	4.46
OR10D3	5.03	5.51	4.45
GSDMA	5.03	5.37	4.73
MCHR1	5.03	5.37	4.87
APOBEC3C	5.03	5.76	4.33
PHACTR1	5.03	5.40	4.52
MIR660	5.03	5.49	4.52
OTTHUMG00000163302	5.03	5.93	4.43
CIAPIN1	5.03	5.46	4.78
UBE2Q2P1	5.03	5.61	3.99
CLCN4	5.03	5.23	4.81
CEP170P1	5.03	5.63	3.83
GYG2	5.03	5.57	4.34
AVPR1B	5.03	5.71	4.62
SLC47A1	5.03	5.33	4.64
SFXN4	5.03	5.32	4.66
LOC728145	5.03	5.41	4.80
FAM43B	5.03	5.47	4.60
CCDC64B	5.03	5.15	4.82
DENND3	5.03	5.21	4.70
LOC100507460	5.03	5.96	4.63
STH	5.03	5.38	4.76
MGAT3	5.03	5.61	4.79
SH3BP1	5.03	5.34	4.60
OR1A2	5.03	5.80	4.31
RILP	5.03	5.44	4.45
SPIRE2	5.03	5.72	4.38
ERI2	5.03	5.29	4.82
ZNF717	5.03	5.72	4.74
TONSL	5.03	5.46	4.59
LAIR1	5.03	5.37	4.41
LOC283731	5.03	5.39	4.71
NAPB	5.03	5.58	4.70
SULT1A1	5.03	5.61	4.51

RGR	5.03	5.64	4.56
OTTHUMG00000170191	5.03	5.52	4.44
TRPT1	5.03	5.25	4.68
ZNF343	5.03	5.55	4.45
IL6R	5.03	5.61	4.19
FLJ13224	5.03	5.23	4.79
DBNDD1	5.03	5.48	4.66
MIR4482-1	5.02	5.35	4.56
OXSM	5.02	5.21	4.80
OR7E12P	5.02	5.89	4.43
LOC100129940	5.02	5.53	4.33
SFXN5	5.02	5.43	4.66
TMEM242	5.02	5.61	4.39
SLC35E3	5.02	5.48	4.55
TPI1	5.02	5.34	4.75
ABCC11	5.02	5.39	4.68
KRTAP16-1	5.02	5.24	4.82
PPIL6	5.02	5.29	4.67
ABCC9	5.02	6.38	3.83
FLJ34521	5.02	5.25	4.79
GOT2	5.02	5.49	4.70
C11orf1	5.02	5.55	4.44
TBC1D25	5.02	5.54	4.66
ARHGAP44	5.02	5.59	4.42
CCL4L1	5.02	5.37	4.57
OCLM	5.02	5.53	4.32
MSL3	5.02	5.53	4.50
DNAH1	5.02	5.41	4.44
CSAG1	5.02	5.84	4.15
SCARNA4	5.02	5.39	4.66
TEX38	5.02	5.37	4.61
C12orf52	5.02	5.21	4.77
VIPR1	5.02	5.47	4.59
OR10Z1	5.02	5.78	4.61
IRS1	5.02	5.31	4.65
ZNF768	5.02	5.39	4.69
GLIPR1L1	5.02	5.33	4.72
ZNF398	5.02	5.48	4.70
TFB2M	5.02	5.43	4.69
CHRND	5.02	5.37	4.24
ARHGEF28	5.02	5.41	4.34
CELF3	5.02	5.47	4.78
TREML1	5.02	5.57	4.58
FLJ12825	5.02	5.47	4.60
FXR2	5.02	5.31	4.82
OTTHUMG00000170611	5.02	5.44	4.53
ZBTB26	5.02	5.34	4.78
ALDH1A3	5.02	5.41	4.83
N6AMT2	5.02	5.52	4.48

NAALADL1	5.02	5.28	4.83
OR51V1	5.02	5.68	4.58
OTTHUMG00000167126	5.02	5.64	4.52
TLE3	5.02	5.33	4.73
ADCY6	5.02	5.26	4.55
GRPEL2	5.02	5.42	4.55
ANKRD42	5.02	5.41	4.72
SNORA47	5.02	5.47	4.63
OTTHUMG00000058148	5.02	5.66	4.39
OTTHUMG00000058669	5.02	5.66	4.39
LOC100505555	5.02	5.44	4.74
UBXN10	5.02	5.37	4.60
CECR7	5.02	5.32	4.76
SPHK1	5.02	5.38	4.72
SH3BGR	5.02	5.25	4.59
PALD1	5.02	5.40	4.71
OTTHUMG00000155064	5.02	5.53	4.37
FLJ32756	5.02	5.63	4.36
DENND2C	5.02	5.46	4.59
METTL21A	5.02	5.33	4.63
PHF15	5.02	5.31	4.73
OTTHUMG00000153152	5.02	5.89	4.36
OR1L1	5.02	5.64	4.50
GS52	5.02	5.49	4.31
DNAJB1	5.02	6.00	4.28
RHBDL2	5.02	5.49	4.36
C3	5.02	5.38	4.49
ENTPD8	5.02	5.36	4.48
PAMR1	5.02	5.62	4.49
GGT3P	5.02	5.75	4.54
AGFG2	5.02	5.58	4.65
LYRM7	5.02	5.72	4.34
PGAP2	5.02	5.33	4.51
IMMP2L	5.02	5.38	4.80
ACAP1	5.02	5.64	4.54
C19orf33	5.02	5.57	4.58
OTTHUMG00000086780	5.02	5.26	4.76
TMEM41B	5.02	5.74	4.09
BCAP29	5.02	5.49	4.37
GAMT	5.02	5.41	4.66
OTTHUMG00000163611	5.02	5.37	4.65
CDADC1	5.02	5.43	4.21
ZNRF2P1	5.02	5.73	4.37
PRCC	5.01	5.27	4.58
SPANXA2	5.01	5.36	4.64
SEC14L1P1	5.01	5.41	4.72
SNORD114-11	5.01	6.76	3.15
KIAA1551	5.01	5.24	4.70
DPEP2	5.01	5.52	4.66

RBM19	5.01	5.33	4.59
OTTHUMG00000041597	5.01	5.65	4.44
SH3RF3	5.01	5.32	4.60
RNF113A	5.01	5.36	4.46
OTTHUMG00000170113	5.01	5.59	4.53
QTRT1	5.01	5.39	4.44
CETN3	5.01	5.29	4.55
LOC100996347	5.01	5.74	4.32
HIST3H2BB	5.01	5.64	4.36
ANKRD20A9P	5.01	5.56	4.51
DCPS	5.01	5.30	4.57
CCL26	5.01	5.35	4.65
ACOT7	5.01	5.28	4.72
PSMD5-AS1	5.01	5.61	4.48
ERAL1	5.01	5.31	4.73
TBCCD1	5.01	5.63	4.23
FBLL1	5.01	5.44	4.51
PANX3	5.01	5.59	4.58
RHBG	5.01	5.37	4.54
SULT1C4	5.01	5.68	3.74
CRIP3	5.01	5.41	4.73
SSNA1	5.01	5.85	4.55
CELA3B	5.01	5.72	4.48
LOC100131691	5.01	5.38	4.62
TMEM185B	5.01	5.60	4.43
OTTHUMG00000169889	5.01	5.58	4.58
GFM1	5.01	5.61	4.52
ZNF570	5.01	5.34	4.73
TXNRD3	5.01	5.42	4.47
TRAJ5	5.01	5.84	4.26
PRKAR1B	5.01	5.42	4.70
KRTAP5-AS1	5.01	5.29	4.80
OTTHUMG00000165804	5.01	5.57	4.46
ZBTB47	5.01	5.25	4.77
RABL2B	5.01	5.31	4.71
FGFRL1	5.01	5.67	4.65
DSN1	5.01	5.45	4.41
RASIP1	5.01	5.68	4.65
MYC	5.01	5.35	4.75
CLDN5	5.01	5.28	4.57
SGK223	5.01	5.27	4.67
CCDC146	5.01	5.45	4.63
CD180	5.01	6.49	4.41
MIR575	5.01	5.72	4.63
TTC16	5.01	5.32	4.57
AKIP1	5.01	5.31	4.59
FUT4	5.01	5.47	4.49
CCDC66	5.01	5.40	4.56
GTF2A2	5.01	5.44	4.52

AGK	5.01	5.34	4.74
LY9	5.01	5.34	4.62
TFE3	5.01	5.39	4.63
POTEM	5.01	6.08	4.61
ATAD3B	5.01	5.33	4.52
C12orf43	5.01	5.27	4.82
ZFHX2	5.01	5.35	4.56
RWDD2B	5.01	5.34	4.44
OR10J3	5.01	5.83	4.55
FFAR3	5.01	5.44	4.62
IL17REL	5.01	5.22	4.67
MIR583	5.01	5.83	4.17
C9orf64	5.01	5.56	4.31
ZNF404	5.01	5.68	4.39
MIR4298	5.01	5.52	4.41
INMT-FAM188B	5.01	5.27	4.84
SETD1A	5.01	5.40	4.69
ITIH4	5.01	5.43	4.84
ADAMTS9	5.01	6.08	4.30
LOC149086	5.01	5.46	4.60
CD1C	5.01	5.65	4.56
C9orf41	5.01	5.60	4.67
OTTHUMG00000013261	5.01	5.68	4.40
NARS2	5.01	5.36	4.63
MIR4529	5.01	6.21	4.52
PCDHB18	5.01	5.43	4.69
OR1N1	5.01	5.72	4.39
FRG2C	5.01	5.56	4.51
GSTA1	5.00	5.52	4.42
SPIN2A	5.00	5.60	4.53
FBXO3	5.00	5.49	4.39
OSGIN2	5.00	5.44	4.70
THAP2	5.00	5.34	4.53
IPW	5.00	5.42	4.56
TIPIN	5.00	5.39	4.52
DSE	5.00	5.25	4.58
OTTHUMG00000179435	5.00	5.34	4.53
LOC647859	5.00	6.06	3.25
PRKAG2	5.00	5.47	4.65
ZFP1	5.00	5.52	4.46
CLDN14	5.00	5.24	4.76
E2F4	5.00	5.43	4.39
METTL4	5.00	5.39	4.55
LNX1	5.00	5.86	4.62
PRTFDC1	5.00	5.70	4.36
TNIK	5.00	5.66	4.45
RPL22L1	5.00	5.38	4.62
ST8SIA5	5.00	5.34	4.67
PEX2	5.00	5.28	4.64

LOC285043	5.00	5.46	4.75
TWIST2	5.00	5.28	4.29
CENPM	5.00	5.49	4.71
TMEM135	5.00	5.40	4.52
OTTHUMG00000171137	5.00	5.86	4.38
ZFYVE19	5.00	5.29	4.69
INPP5E	5.00	5.28	4.71
OTTHUMG00000151887	5.00	5.40	4.77
PPM1D	5.00	5.13	4.75
OTTHUMG00000156122	5.00	5.48	4.45
CTBP2	5.00	5.25	4.45
ZNF701	5.00	5.70	4.29
MIR3182	5.00	5.34	4.60
HPGDS	5.00	6.56	4.06
KCNMB2	5.00	5.42	4.61
SIGLEC9	5.00	6.09	4.52
PLCB2	5.00	5.58	4.21
AADAT	5.00	5.21	4.88
OTTHUMG00000160261	5.00	5.64	4.30
CCDC51	5.00	5.26	4.59
PTK2B	5.00	5.47	4.43
GRAMD1A	5.00	5.29	4.46
ZNF646	5.00	5.39	4.30
TMPRSS9	5.00	5.27	4.74
COA5	5.00	5.49	4.53
KCNJ15	5.00	5.41	4.46
RSRC1	5.00	5.29	4.54
OTTHUMG00000177058	5.00	5.37	4.66
FCHSD1	5.00	5.33	4.65
ADRBK1	5.00	5.22	4.60
ZNF200	5.00	5.13	4.86
RNF212	5.00	5.30	4.59
CXorf36	5.00	5.54	4.56
PGAP1	5.00	5.53	4.19
C4orf47	5.00	5.68	4.31
MSMO1	5.00	5.39	4.10
LOC648570	5.00	5.94	3.74
OTTHUMG00000009742	5.00	5.60	4.35
OTTHUMG00000170896	5.00	5.45	4.53
SCN1B	5.00	5.37	4.31
C1orf106	5.00	5.29	4.73
MIR3175	5.00	5.37	4.47
FXYD4	5.00	5.39	4.65
YWHAH	5.00	5.52	4.23
LOC100131094	5.00	5.36	4.52
RNA5SP222	5.00	5.81	4.20
CCDC42B	5.00	5.69	4.54
P2RX2	5.00	5.60	4.53
TMEM200B	5.00	5.45	4.36

WNT2B	5.00	5.26	4.64
LOC100131655	5.00	5.32	4.61
LOC407835	5.00	5.26	4.80
SULT4A1	5.00	5.57	4.72
OTTHUMG00000153822	5.00	5.39	4.56
FAM136A	5.00	5.57	4.65
MYCBP2-AS2	5.00	5.27	4.73
SMYD4	5.00	5.26	4.70
HSPA1L	5.00	5.26	4.82
LOC400685	5.00	5.56	4.50
MAD2L1BP	5.00	5.56	4.63
OTTHUMG00000020901	5.00	5.33	4.31
NRDE2	5.00	5.25	4.38
DMRT1	5.00	5.39	4.64
KISS1R	5.00	5.32	4.86
SHISA2	5.00	5.13	4.83
LOC100509541	5.00	5.47	4.51
LOC613206	5.00	5.58	4.57
ASTN1	5.00	5.47	4.69
CXorf24	5.00	5.35	4.59
LETM1	5.00	5.28	4.64
E2F3-IT1	5.00	5.32	4.62
MRPL38	5.00	5.52	4.59
RAB24	5.00	5.32	4.61
ARGFX	5.00	6.05	4.58
DOPEY2	5.00	5.29	4.58
LOC100505636	5.00	5.37	4.69
CCDC78	5.00	5.51	4.71
PTGS2	5.00	6.73	4.06
SERTAD3	5.00	5.32	4.71
MIOX	5.00	5.44	4.66
CACNA1G-AS1	5.00	5.39	4.58
PRSS53	5.00	5.31	4.62
OTTHUMG00000171380	4.99	5.79	4.36
THAP6	4.99	5.28	4.76
MIR30E	4.99	6.27	3.39
NRXN2	4.99	5.52	4.58
KRT75	4.99	5.53	4.40
OTTHUMG00000035804	4.99	5.37	4.63
PIM1	4.99	5.48	4.40
MAP3K11	4.99	5.36	4.48
CCNI2	4.99	5.36	4.45
GPATCH4	4.99	5.32	4.62
TMEM82	4.99	5.52	4.56
SNORA23	4.99	5.60	4.38
C17orf50	4.99	5.35	4.72
DAPK3	4.99	5.71	4.32
IVD	4.99	5.47	4.55
MAGIX	4.99	5.25	4.66

LOC100506295	4.99	5.36	4.51
BMP7	4.99	5.16	4.52
CARD14	4.99	5.29	4.64
ANKUB1	4.99	5.49	4.54
AKNA	4.99	5.41	4.71
MIR329-1	4.99	5.89	4.37
DIAPH3-AS1	4.99	5.30	4.23
IPP	4.99	5.51	4.45
MTX1	4.99	5.50	4.66
RNF222	4.99	5.46	4.64
LRRC17	4.99	6.80	3.76
MIR3691	4.99	5.50	4.42
TMEM235	4.99	5.42	4.70
MIR365B	4.99	5.29	4.66
ZNF35	4.99	5.38	4.29
KIF12	4.99	5.26	4.74
TRAV1-1	4.99	5.65	4.44
C11orf63	4.99	5.33	4.69
EPB41	4.99	5.23	4.77
ENTPD2	4.99	5.16	4.77
RNA5-8SP4	4.99	5.49	4.61
KCNIP2	4.99	5.42	4.63
LOC153684	4.99	5.27	4.42
LINGO4	4.99	5.71	4.33
LOC401180	4.99	5.38	4.51
RNF39	4.99	5.45	4.62
SNORA5C	4.99	5.35	4.68
HTR3A	4.99	5.52	4.69
FAM46B	4.99	5.37	4.55
TNN	4.99	5.61	4.71
MYBPH	4.99	5.36	4.64
YTHDF2	4.99	5.17	4.47
TTC18	4.99	5.57	4.41
ICMT	4.99	5.60	4.23
ASMT	4.99	5.18	4.55
PLA2G15	4.99	5.31	4.30
EPO	4.99	5.24	4.74
OR2AG1	4.99	5.54	4.25
ULK3	4.99	5.22	4.52
OTTHUMG00000015138	4.99	5.41	4.59
CSNK1G1	4.99	5.26	4.58
LOC100130950	4.99	5.35	4.46
HAUS8	4.99	5.34	4.63
DNM1P35	4.99	5.58	4.71
PCDHB15	4.99	5.61	4.12
NEURL1B	4.99	5.22	4.84
TBC1D29	4.99	5.60	4.48
CALML3	4.99	5.39	4.56
FAM53C	4.99	5.29	4.57

MIR3179-1	4.99	5.53	4.61
FHL3	4.99	5.71	4.53
ZNF669	4.99	5.31	4.40
ABHD15	4.99	5.32	4.49
LONP1	4.99	5.21	4.52
MSANTD4	4.99	5.57	4.21
GCDH	4.99	5.13	4.83
DARS2	4.99	5.31	4.74
ZNF14	4.99	5.44	4.24
SLX4	4.99	5.14	4.59
ZNF597	4.99	5.22	4.67
BPIFB3	4.99	5.35	4.68
CBS	4.99	5.54	4.42
PVR	4.99	5.25	4.51
LYPD2	4.99	5.27	4.72
LOC200772	4.99	5.32	4.62
SEPT4	4.99	5.73	4.25
HIST2H2BC	4.99	5.69	4.49
KCNJ4	4.99	5.44	4.35
SLC25A14	4.99	5.33	4.58
WDSUB1	4.98	5.32	4.47
RPS6KA2	4.98	5.23	4.60
MIRLET7D	4.98	5.32	4.61
RABL6	4.98	5.22	4.62
HCCAT5	4.98	5.42	4.37
LRWD1	4.98	5.41	4.56
LOC100288594	4.98	5.25	4.62
C10orf99	4.98	5.37	4.64
OTTHUMG00000162202	4.98	5.48	4.29
IBA57	4.98	5.63	4.64
LRRC3	4.98	5.39	4.67
TRBV19	4.98	5.40	4.66
TTF2	4.98	5.36	4.51
NYAP1	4.98	5.41	4.42
MARK4	4.98	5.20	4.54
TMEM136	4.98	5.35	4.22
SEC31B	4.98	5.32	4.63
GLP1R	4.98	5.27	4.71
OR5I1	4.98	6.03	4.10
SNCB	4.98	5.23	4.66
PSMD5	4.98	5.68	4.12
GPR1	4.98	6.39	3.65
MED19	4.98	5.22	4.64
FJX1	4.98	5.60	4.29
APBA3	4.98	5.23	4.64
LOC100133315	4.98	5.26	4.40
TTC9	4.98	6.10	4.06
DNHD1	4.98	5.28	4.72
ZNF839	4.98	5.27	4.52

LOC339874	4.98	5.59	4.53
CDC20	4.98	5.51	4.50
ZNF564	4.98	5.54	4.34
ELANE	4.98	5.16	4.75
MED22	4.98	5.35	4.76
SEMA3G	4.98	5.36	4.79
SCIMP	4.98	5.89	4.63
TNFAIP8L3	4.98	5.41	4.29
HIRIP3	4.98	5.38	4.50
IL2RA	4.98	5.35	4.68
LOC401296	4.98	5.25	4.76
AZGP1P1	4.98	5.50	4.46
GHRHR	4.98	5.43	4.51
LOC729911	4.98	5.34	4.68
TLR9	4.98	5.35	4.61
OTTHUMG00000137387	4.98	5.83	4.18
MTMR9LP	4.98	5.45	4.48
SLC11A1	4.98	6.27	4.54
EEF1DP3	4.98	5.45	4.48
IFT43	4.98	5.43	4.45
EEPD1	4.98	5.39	4.60
MFSD11	4.98	5.26	4.72
MCTP2	4.98	5.52	4.28
DACT3-AS1	4.98	5.45	4.74
FAM109A	4.98	5.48	4.46
USP39	4.98	5.33	4.47
RPH3AL	4.98	5.33	4.46
MRPS2	4.98	5.48	4.78
RORC	4.98	5.88	4.11
BAALC	4.98	5.50	4.22
TRABD2A	4.98	5.35	4.70
CLCNKB	4.98	5.74	4.31
TLR7	4.98	6.47	4.06
DUSP15	4.98	5.33	4.55
EFHD1	4.98	5.41	4.42
SDCBP2-AS1	4.98	5.28	4.51
RIPPLY1	4.98	5.49	4.59
MYL2	4.98	5.66	4.34
ARHGEF33	4.98	5.25	4.64
C14orf183	4.98	5.51	4.55
FCGR2A	4.98	6.19	4.23
METTL12	4.98	5.53	4.44
ZNF174	4.98	5.29	4.50
OR5AU1	4.98	5.73	4.49
LINC00152	4.98	5.47	4.28
FAM163B	4.98	5.53	4.45
PLA2G5	4.98	5.60	4.55
PARS2	4.98	5.21	4.58
DRD4	4.98	5.39	4.67

RFX7	4.98	5.42	4.54
C10orf128	4.98	5.38	4.55
MIR3973	4.98	5.84	4.51
LYPD4	4.97	5.40	4.55
OTTHUMG00000169970	4.97	5.50	4.61
OTTHUMG00000166987	4.97	5.97	4.15
BRD9	4.97	5.20	4.55
LOC729966	4.97	5.54	4.71
MSLN	4.97	5.32	4.60
SCNN1D	4.97	5.15	4.79
MIR222	4.97	5.97	4.43
PGD	4.97	5.52	4.65
OTTHUMG00000160821	4.97	5.21	4.35
PECR	4.97	5.27	4.65
C11orf93	4.97	5.24	4.31
TPK1	4.97	5.27	4.56
CASP10	4.97	5.36	4.24
LOC650293	4.97	5.94	3.75
MEX3D	4.97	5.35	4.61
LOC100287175	4.97	5.39	4.65
CABYR	4.97	5.42	4.60
TUSC5	4.97	5.64	4.56
AGPAT9	4.97	5.24	4.60
LINC00202-1	4.97	5.51	4.46
UHRF1BP1	4.97	5.60	4.18
CUBN	4.97	5.18	4.46
ZAP70	4.97	5.56	4.67
FSCN2	4.97	5.35	4.42
ZSCAN1	4.97	5.21	4.68
DNAH11	4.97	5.76	4.14
LOC100508226	4.97	5.42	4.25
GUCY1A2	4.97	5.43	4.56
LINC00899	4.97	5.28	4.64
GPR135	4.97	5.38	4.13
FAM166B	4.97	5.17	4.66
GPC2	4.97	5.53	4.55
ZNHIT3	4.97	5.40	4.27
G6PD	4.97	5.21	4.75
KLRG2	4.97	5.25	4.77
MYD88	4.97	5.32	4.56
SAA1	4.97	9.41	3.85
GTF2H2	4.97	5.62	4.04
SRSF12	4.97	5.45	4.51
C17orf75	4.97	5.24	4.66
TICAM1	4.97	5.34	4.60
ZBTB8OS	4.97	5.33	4.51
LOC100506343	4.97	5.28	4.66
TNFRSF11A	4.97	5.66	4.49
MIR4273	4.97	5.69	4.10

TRAPPC6A	4.97	5.38	4.63
KLHL11	4.97	5.51	4.61
CYP27C1	4.97	5.86	4.12
NPY6R	4.97	5.55	4.24
CDKN1C	4.97	5.11	4.82
FAM83H-AS1	4.97	5.24	4.60
TSPYL6	4.97	5.48	4.42
IL34	4.97	5.53	4.42
B3GALT6	4.97	5.50	4.41
LOC100505722	4.97	5.33	4.42
TGM4	4.97	5.36	4.56
HOXC12	4.97	5.41	4.64
TNFSF11	4.97	5.49	4.65
ZNF891	4.97	5.29	4.56
TLR5	4.97	5.51	4.47
DLGAP3	4.97	5.43	4.60
TMEM178A	4.97	5.28	4.57
VAC14-AS1	4.97	5.47	4.73
OTTHUMG00000014933	4.97	6.31	3.41
HMGN5	4.97	5.41	4.53
CAPN6	4.97	6.59	4.13
E2F3	4.97	5.42	4.31
OTTHUMG00000170116	4.97	5.44	4.63
DDIT3	4.97	5.79	4.06
THSD1	4.97	5.57	4.50
SEPT12	4.97	5.33	4.70
CCDC122	4.97	5.22	4.54
C14orf93	4.97	5.33	4.67
RASSF7	4.97	5.47	4.35
ARHGEF3	4.97	5.55	4.66
LINC00338	4.97	5.38	4.30
FARS2	4.97	5.32	4.32
MIR551B	4.97	5.71	4.47
SMO	4.97	5.32	4.73
STK32B	4.97	5.70	4.02
SLC22A6	4.97	5.33	4.52
CFTR	4.97	5.70	4.32
RDX	4.97	5.31	4.29
C3orf52	4.97	5.41	4.55
FBF1	4.97	5.31	4.66
PACSIN3	4.97	5.23	4.66
KCNIP2-AS1	4.97	5.50	4.64
ZNF547	4.96	5.23	4.38
C9orf123	4.96	5.15	4.36
OTTHUMG00000172027	4.96	6.01	4.28
FAM133DP	4.96	5.47	4.17
TXN2	4.96	5.21	4.71
DNMBP-AS1	4.96	5.25	4.65
FGF14-IT1	4.96	5.51	4.50

SCNN1A	4.96	5.72	4.12
KIAA0513	4.96	5.30	4.55
RBM43	4.96	5.43	4.40
EPB41L5	4.96	5.15	4.76
LINC00856	4.96	5.40	4.52
TBX19	4.96	5.53	4.61
LOC100508227	4.96	5.49	4.49
SEC14L5	4.96	5.23	4.56
ZBED5-AS1	4.96	5.28	4.69
CTNNBL1	4.96	5.54	4.35
IL17C	4.96	5.39	4.54
ENOX2	4.96	5.47	4.04
THEM4	4.96	5.58	4.37
NOC4L	4.96	5.17	4.52
GOLGA8DP	4.96	5.39	4.54
RUNDC1	4.96	5.30	4.75
MPV17L2	4.96	5.16	4.64
RNASE2	4.96	5.62	4.06
LOC100128288	4.96	5.44	4.47
IRX2	4.96	5.34	4.53
SPRY1	4.96	5.58	4.06
TNFSF14	4.96	5.25	4.69
LOC100287879	4.96	5.65	4.59
PIRT	4.96	5.19	4.65
C9orf131	4.96	5.43	4.60
TEK	4.96	5.79	4.10
TMEM194B	4.96	5.32	4.75
DZIP1L	4.96	5.34	4.62
LOC338963	4.96	5.36	4.73
LOC254128	4.96	5.20	4.37
ADCK4	4.96	5.42	4.37
ARHGAP27	4.96	5.49	4.56
OR10V2P	4.96	5.50	4.40
RRM2	4.96	5.39	4.39
ZBTB8A	4.96	5.49	4.35
BAHCC1	4.96	5.51	4.59
LOC100507447	4.96	5.85	4.25
RNA5SP35	4.96	5.92	4.17
FOXD2	4.96	5.30	4.70
DOLPP1	4.96	5.29	4.46
MIR193B	4.96	5.36	4.49
CHST5	4.96	5.53	4.67
ZSWIM1	4.96	5.26	4.59
MCC	4.96	5.29	4.50
IGSF10	4.96	5.52	4.60
PRMT10	4.96	5.25	4.63
MIR504	4.96	5.27	4.53
COMMD8	4.96	5.57	4.54
MIR371A	4.96	5.54	4.45

MUC5B	4.96	5.23	4.69
SH3GL1	4.96	5.44	4.60
ABHD12B	4.96	5.44	4.46
GUSBP11	4.96	5.49	4.54
LOC100288637	4.96	6.10	4.49
CEP170B	4.96	5.19	4.77
LOC646719	4.96	5.61	4.46
ZFAND6	4.96	5.30	4.56
TMCC2	4.96	5.39	4.62
OTTHUMG00000171048	4.96	5.21	4.67
ACTR1B	4.96	5.45	4.55
MIRLET7E	4.96	5.42	4.64
ZDHHC15	4.96	5.42	4.53
NUDCD3	4.96	5.28	4.46
CXXC11	4.96	5.25	4.70
OTTHUMG00000021366	4.96	5.30	4.63
DOC2B	4.96	5.46	4.49
CT47B1	4.96	5.26	4.42
DHX57	4.96	5.22	4.51
PQLC2	4.96	5.18	4.77
FOXE3	4.95	5.51	4.58
RNU2-7P	4.95	5.70	3.81
LOC202781	4.95	5.23	4.58
CD164L2	4.95	5.34	4.50
RASGRP3	4.95	5.77	4.03
FEZ2	4.95	5.42	4.49
CEP78	4.95	5.39	4.44
OTTHUMG00000018097	4.95	5.39	4.50
DHCR7	4.95	5.26	4.46
DHX58	4.95	5.43	4.61
LOC100128059	4.95	5.59	4.48
TMEM211	4.95	5.44	4.33
ABHD14A-ACY1	4.95	5.16	4.67
UIMC1	4.95	5.29	4.47
EPHB6	4.95	5.42	4.66
MIR494	4.95	5.79	4.35
LINC00851	4.95	5.27	4.61
HELQ	4.95	5.21	4.60
CCL27	4.95	5.37	4.65
NELFB	4.95	5.42	4.73
IGDCC3	4.95	5.44	4.55
SLC16A13	4.95	5.20	4.46
OTTHUMG00000165485	4.95	5.46	4.64
HCG20	4.95	5.55	4.70
C8G	4.95	5.50	4.52
HOXD8	4.95	5.33	4.58
LOC646626	4.95	5.11	4.71
LOC642131	4.95	5.66	4.31
SNORD114-13	4.95	6.78	3.57

LOC100128531	4.95	5.34	4.63
NUDT18	4.95	5.57	4.59
LINC00926	4.95	5.12	4.73
TCAIM	4.95	5.27	4.39
HBG2	4.95	5.48	4.60
CACNA2D2	4.95	5.20	4.75
RNA5SP160	4.95	5.85	3.87
GPR21	4.95	5.35	4.38
LOC100507564	4.95	5.16	4.62
ST3GAL3	4.95	5.24	4.61
AQP10	4.95	5.45	4.62
LAYN	4.95	5.24	4.69
MINOS1P1	4.95	5.47	4.49
SLC52A1	4.95	5.61	4.39
LOC100996291	4.95	5.43	4.34
PLCB4	4.95	5.42	4.59
RLTPR	4.95	5.17	4.67
ABCB6	4.95	5.45	4.47
ZNF568	4.95	5.32	4.61
SNORD109A	4.95	5.49	4.57
C19orf82	4.95	5.43	4.36
HMCN2	4.95	5.07	4.70
TAC3	4.95	5.51	4.36
OBSL1	4.95	5.24	4.59
C7orf49	4.95	5.23	4.50
CT47A6	4.95	5.22	4.35
OTTHUMG00000160063	4.95	5.47	4.67
HDAC4	4.95	5.29	4.56
ZNF276	4.95	5.27	4.69
OTTHUMG00000165386	4.95	5.27	4.71
RNA5SP320	4.95	5.67	4.09
ZNF737	4.95	5.68	3.91
LOC644172	4.95	5.34	4.58
MIR181A1HG	4.95	5.47	4.29
CLEC4F	4.95	5.43	4.46
LOR	4.95	5.37	4.63
WNT9A	4.95	5.62	4.56
POLR3B	4.95	5.36	4.41
RASGRP4	4.95	5.18	4.65
NPAS3	4.95	5.71	4.49
CRYGD	4.95	5.43	4.67
MPHOSPH9	4.95	5.36	4.56
FLJ37644	4.95	5.47	4.48
CTSH	4.95	5.37	4.64
PPCDC	4.95	5.36	4.61
TMEM221	4.95	5.39	4.62
RDH10	4.95	5.59	4.74
ING5	4.95	5.36	4.69
PUS1	4.95	5.26	4.62

MIR543	4.95	5.69	4.17
OTTHUMG00000152620	4.95	6.04	4.39
CCDC120	4.95	5.34	4.69
CYP17A1-AS1	4.95	5.27	4.56
POU3F2	4.95	5.13	4.66
TBCE	4.95	5.23	4.63
PSKH1	4.95	5.29	4.65
LOC100996345	4.94	5.50	4.52
C11orf80	4.94	5.41	4.55
HCAR1	4.94	5.43	4.54
NUFIP1	4.94	5.45	4.06
CRB3	4.94	5.28	4.66
CD83	4.94	5.94	4.51
PSMC3IP	4.94	5.42	4.50
KDM5C	4.94	5.37	4.36
RMND5B	4.94	5.32	4.51
EPB42	4.94	5.27	4.66
RNA5SP182	4.94	5.90	4.40
PKMYT1	4.94	5.22	4.46
SIGLEC10	4.94	5.36	4.36
GRAMD1B	4.94	5.30	4.46
ETV1	4.94	5.33	4.63
EVX1	4.94	5.25	4.55
PHLDB3	4.94	5.61	4.57
PDE1B	4.94	5.11	4.72
LOC729275	4.94	5.25	4.16
ATP2B2-IT1	4.94	5.13	4.67
NCAPD3	4.94	5.13	4.80
RNA5SP385	4.94	6.26	3.18
LYRM9	4.94	5.22	4.50
ABT1	4.94	5.21	4.48
C19orf68	4.94	5.35	4.39
ZNF620	4.94	5.36	4.38
LOC100505761	4.94	5.33	4.47
PROP1	4.94	5.36	4.52
BMS1P2	4.94	5.25	4.40
NDUFAF3	4.94	5.21	4.52
C22orf46	4.94	5.20	4.72
ZKSCAN8	4.94	5.33	4.50
MOB3C	4.94	5.37	4.44
KIFC2	4.94	5.29	4.55
OTTHUMG00000168493	4.94	5.62	4.28
UBTD1	4.94	5.24	4.55
TM2D2	4.94	5.47	4.32
FCHO1	4.94	5.26	4.75
POLRMT	4.94	5.39	4.66
MALT1	4.94	5.23	4.63
SMTNL1	4.94	5.29	4.70
KCNK15	4.94	5.29	4.25

OTTHUMG00000175754	4.94	5.20	4.66
LIAS	4.94	5.41	4.09
C1QTNF8	4.94	5.48	4.59
DSCR3	4.94	5.27	4.38
TMEM44	4.94	5.40	4.46
OTTHUMG00000154637	4.94	5.86	3.98
C19orf77	4.94	5.52	4.32
TTC21A	4.94	5.16	4.64
RBP2	4.94	5.27	4.23
LONRF1	4.94	5.48	4.06
FAM13C	4.94	5.47	4.44
WWOX	4.94	5.19	4.71
LRTM2	4.94	5.39	4.38
TSPO2	4.94	5.30	4.54
ATHL1	4.94	5.31	4.45
SASS6	4.94	5.36	4.37
B4GALNT1	4.94	5.40	4.48
UBQLN3	4.94	5.37	4.48
HS3ST3A1	4.94	5.33	4.63
LOC100506965	4.94	5.93	3.65
ELMOD3	4.94	5.18	4.71
FBXW9	4.94	5.72	4.54
SPIN3	4.94	5.38	4.35
FGF13	4.94	5.22	4.75
LOC100130502	4.94	5.55	4.43
OTTHUMG00000170998	4.94	5.38	4.43
HSPA4L	4.94	5.72	4.25
FAM221B	4.94	5.20	4.67
PCGF6	4.94	5.23	4.79
TRO	4.94	5.36	4.58
MIR501	4.94	5.23	4.48
IL27	4.94	5.14	4.62
LOC100507376	4.94	5.52	4.57
GPBAR1	4.94	5.30	4.53
RMI2	4.94	5.33	4.68
ZNF816-ZNF321P	4.94	5.16	4.62
KIF11	4.94	5.67	4.46
DRG2	4.94	5.06	4.75
LNX2	4.94	5.45	4.27
IL10RB-AS1	4.94	5.28	4.65
CARS-AS1	4.94	5.20	4.66
SHISA5	4.94	5.37	4.48
NOD1	4.94	5.47	4.42
ADAMTS13	4.94	5.42	4.45
PATZ1	4.94	5.44	4.61
SFT2D3	4.94	5.36	4.44
LOC729870	4.94	5.33	4.50
TPSG1	4.94	5.47	4.49
KLF1	4.94	5.32	4.69

DNAAF3	4.94	5.23	4.77
OTTHUMG00000152739	4.94	5.33	4.52
PJA1	4.94	5.50	4.43
MIR609	4.94	5.72	4.22
TSPAN31	4.94	5.25	4.56
PPP1R16A	4.94	5.16	4.73
RNF25	4.94	5.14	4.56
TTN	4.94	5.27	4.66
OTTHUMG00000022086	4.94	5.82	3.83
BORA	4.94	5.29	4.60
BCAR1	4.93	5.27	4.54
C20orf173	4.93	5.28	4.50
CDCP1	4.93	5.55	4.46
HOXB6	4.93	5.38	4.37
ZNF32-AS1	4.93	5.42	4.03
LEPROTL1	4.93	5.47	4.14
C1RL-AS1	4.93	5.46	4.51
FTCD-AS1	4.93	5.50	4.59
LINC00319	4.93	5.39	4.59
TRABD2B	4.93	5.36	4.60
IFT140	4.93	5.26	4.29
GPR146	4.93	5.40	4.66
C10orf82	4.93	5.25	4.61
FICD	4.93	5.13	4.64
POC1B-GALNT4	4.93	5.23	4.45
MUC6	4.93	5.14	4.69
CLCN2	4.93	5.39	4.62
XIAP	4.93	5.18	4.65
KMT2B	4.93	5.21	4.55
OTTHUMG00000157240	4.93	5.21	4.60
HEATR2	4.93	5.27	4.49
KRTAP10-2	4.93	5.22	4.49
HSDL1	4.93	5.49	4.42
CLEC1A	4.93	5.51	4.41
MAFF	4.93	5.23	4.69
STXBP2	4.93	5.31	4.60
ADAMTS1	4.93	5.84	4.22
SEC1P	4.93	5.20	4.63
SAMD14	4.93	5.41	4.55
LILRB5	4.93	5.18	4.58
LOC100287177	4.93	5.35	4.28
FGD2	4.93	5.69	4.52
SAMD13	4.93	5.16	4.67
LRRC10	4.93	5.65	4.54
SNORD94	4.93	5.58	4.57
FAM198B	4.93	5.20	4.61
PRAMEF22	4.93	5.76	3.50
HMP19	4.93	5.37	4.46
LOC100505549	4.93	5.36	4.62

ZNF611	4.93	5.55	4.45
MMP23B	4.93	5.34	4.10
ZNF713	4.93	5.20	4.42
TMEM31	4.93	5.41	4.58
SGK1	4.93	5.63	4.28
TTC36	4.93	5.40	4.44
ABCA3	4.93	5.31	4.65
EHBP1L1	4.93	5.49	4.47
EEFSEC	4.93	5.21	4.50
ZMYND12	4.93	5.30	4.46
EMILIN1	4.93	5.17	4.63
PDCD1LG2	4.93	5.39	4.18
ZNF708	4.93	5.44	4.38
SNORD115-42	4.93	5.86	3.84
CLDN7	4.93	5.22	4.55
DDX31	4.93	5.31	4.45
CD27-AS1	4.93	5.08	4.73
LOC81691	4.93	5.17	4.64
DTX3	4.93	5.26	4.48
METTL16	4.93	5.14	4.63
RPTOR	4.93	5.34	4.63
TSEN15	4.93	5.42	4.66
GBP4	4.93	5.88	4.21
ZNF84	4.93	5.44	4.16
PACSIN1	4.93	5.32	4.53
GRID2IP	4.93	5.15	4.61
KRT6B	4.93	5.78	4.20
CCDC102A	4.92	5.25	4.63
ADCY7	4.92	5.55	4.15
ZNF417	4.92	5.46	4.31
ADAT2	4.92	5.25	4.55
BRMS1	4.92	5.32	4.21
TRIM31-AS1	4.92	5.41	4.51
RPGRIP1L	4.92	5.55	4.55
TTC31	4.92	5.31	4.50
C11orf34	4.92	5.32	4.55
TMED8	4.92	5.14	4.72
GCAT	4.92	5.29	4.60
NFE2L3	4.92	5.55	4.60
SH2B3	4.92	5.32	4.61
SGOL1-AS1	4.92	5.36	4.44
ZNF697	4.92	5.13	4.65
ADM2	4.92	5.31	4.51
CYP2W1	4.92	5.20	4.26
PCSK1	4.92	5.28	4.60
PKLR	4.92	5.28	4.61
KIRREL3	4.92	5.28	4.38
EXTL2	4.92	5.53	4.45
PIGV	4.92	5.43	4.63

MED9	4.92	5.32	4.45
ARAP3	4.92	5.43	4.57
KLK4	4.92	5.26	4.68
MEN1	4.92	5.30	4.68
TRIM62	4.92	5.30	4.63
BZRAP1	4.92	5.27	4.53
MIR1260A	4.92	5.79	4.49
METTL23	4.92	5.38	4.24
PPIF	4.92	5.28	4.37
FAM118B	4.92	5.27	4.33
ZNF19	4.92	5.31	4.45
GMPPB	4.92	5.21	4.72
FFAR1	4.92	5.20	4.32
PTPRB	4.92	6.27	3.44
MT1A	4.92	5.72	3.62
GRWD1	4.92	5.19	4.65
DNAJB7	4.92	5.25	4.47
PAPLN	4.92	5.19	4.66
MIR378E	4.92	5.72	3.64
SNORA72	4.92	5.20	4.53
APOBEC3F	4.92	5.76	4.41
TM4SF19	4.92	5.60	4.41
FLJ35816	4.92	5.44	4.55
NUP107	4.92	5.29	4.26
UBN1	4.92	5.38	4.33
PDGFB	4.92	5.31	4.57
POP7	4.92	5.32	4.45
DGKI	4.92	6.27	4.09
ZNF180	4.92	5.39	4.40
CARD10	4.92	5.29	4.46
OTTHUMG00000163325	4.92	5.90	4.34
PARK2	4.92	5.21	4.56
C9orf91	4.92	5.33	4.53
ASGR1	4.92	5.24	4.58
GCK	4.92	5.10	4.68
SLC39A3	4.92	5.24	4.42
LIPA	4.92	5.20	4.24
UBB	4.92	5.28	4.37
OTTHUMG00000164068	4.92	5.21	4.49
TMEM161B	4.92	5.34	4.52
MARK2	4.92	5.39	4.49
NPTX2	4.92	5.11	4.78
EPHB4	4.92	5.37	4.47
SCGB1B2P	4.92	5.41	4.38
NPHP4	4.92	5.18	4.64
OSGEP	4.92	5.48	4.39
LOC100127940	4.92	5.42	4.47
ZNF721	4.92	5.51	4.25
TFAP4	4.92	5.54	4.58

NHEJ1	4.92	5.19	4.54
SIGLEC14	4.92	5.58	4.55
SMTNL2	4.92	5.29	4.50
MB21D2	4.92	5.22	4.37
SLC18B1	4.92	5.55	4.50
PDPR	4.92	5.42	3.58
XPNPEP2	4.92	5.27	4.43
BID	4.92	5.28	4.52
OTTHUMG00000041231	4.92	5.62	4.26
LOC148696	4.92	5.40	4.30
ELMOD2	4.91	5.87	4.09
UCHL1	4.91	5.29	4.59
LOC100507291	4.91	5.16	4.65
FLRT1	4.91	5.30	4.40
PRIM2	4.91	5.32	4.51
REG3G	4.91	5.49	4.32
EFNA1	4.91	5.47	4.38
C9orf69	4.91	5.08	4.61
OTTHUMG00000162866	4.91	5.40	4.58
CNOT7	4.91	5.11	4.63
SDR39U1	4.91	5.18	4.68
GP6	4.91	5.19	4.53
OTOS	4.91	5.15	4.68
OTTHUMG00000170764	4.91	5.56	4.27
NCF2	4.91	6.18	4.21
SRGAP3	4.91	5.22	4.67
LEF1	4.91	5.49	4.26
KCNK2	4.91	5.93	4.54
SH2B1	4.91	5.24	4.52
NLRP3	4.91	5.35	4.22
BACE2-IT1	4.91	5.48	4.48
CEND1	4.91	5.27	4.64
RMDN3	4.91	5.39	4.34
HCN3	4.91	5.21	4.51
GGA1	4.91	5.30	4.66
PM20D2	4.91	5.38	4.36
INO80C	4.91	5.30	4.50
SLC22A9	4.91	5.20	4.58
GRM1	4.91	5.29	4.59
SMCR7L	4.91	5.26	4.52
NBEAL2	4.91	5.18	4.78
OR10K2	4.91	5.39	4.30
SLC16A5	4.91	5.38	4.54
TUBB4A	4.91	5.64	4.39
C4orf33	4.91	5.46	4.37
VARS	4.91	5.23	4.54
TRIM16L	4.91	5.38	4.52
MYO18B	4.91	5.34	4.72
FEV	4.91	5.29	4.68

CATSPER3	4.91	5.24	4.58
GFAP	4.91	5.46	4.33
FER1L4	4.91	5.19	4.61
KIRREL3-AS2	4.91	5.50	4.35
LINGO1	4.91	5.18	4.67
FUZ	4.91	5.27	4.73
TOX	4.91	5.37	4.39
C5orf55	4.91	5.22	4.48
KRBA2	4.91	5.48	4.59
EIF1AY	4.91	5.93	3.64
ADAM32	4.91	5.34	4.27
PPIAL4E	4.91	5.52	4.20
MBOAT2	4.91	5.18	4.29
C1orf110	4.91	5.57	4.40
MMP24	4.91	5.24	4.41
KCNK17	4.91	5.50	4.45
PRPSAP1	4.91	5.26	4.53
PAIP2B	4.91	5.54	3.98
SPPL2C	4.91	5.47	4.53
OTTHUMG00000021126	4.91	5.25	4.44
CLDN12	4.91	5.36	4.38
CTDSP1	4.91	5.20	4.34
PTCD2	4.91	5.28	4.60
MSX2	4.91	5.27	3.91
OTTHUMG00000180598	4.91	5.28	4.66
UPRT	4.91	5.38	4.40
OTTHUMG00000032468	4.91	5.46	4.44
CRYBA4	4.91	5.25	4.38
ECEL1	4.91	5.38	4.56
NR1H2	4.91	5.63	4.64
LOC101059939	4.91	5.29	4.20
ADA	4.91	5.61	4.50
IFNL1	4.91	5.60	4.44
SNORD116-14	4.90	5.34	3.95
ASB6	4.90	5.19	4.46
GPRC5C	4.90	5.43	4.36
LMAN1L	4.90	5.29	4.40
OTTHUMG00000159737	4.90	5.52	4.27
IGBP1	4.90	5.18	4.54
OTTHUMG00000015922	4.90	5.30	4.64
LOC730183	4.90	5.66	4.39
FOXA3	4.90	5.13	4.53
SCAMP4	4.90	5.33	4.42
PRR7-AS1	4.90	5.42	4.52
NUAK1	4.90	5.52	3.89
DDX3Y	4.90	6.15	2.95
PRSS36	4.90	5.33	4.53
GPR12	4.90	5.38	4.56
C22orf23	4.90	5.21	4.58

LOC340239	4.90	5.53	4.40
EAF2	4.90	5.26	4.44
STX3	4.90	5.26	4.35
LINC00702	4.90	6.37	3.71
CCDC40	4.90	5.15	4.59
LOC90834	4.90	5.18	4.72
LOC100506804	4.90	5.34	4.59
C3orf18	4.90	5.20	4.70
MCF2L-AS1	4.90	5.44	4.36
SEPT1	4.90	5.05	4.74
SLC4A11	4.90	5.44	4.55
UCKL1-AS1	4.90	5.34	4.49
KRT19	4.90	5.43	4.48
BEAN1	4.90	5.30	4.32
ASCC2	4.90	5.31	4.64
TMEM171	4.90	5.16	4.46
LOC100996549	4.90	5.40	4.57
MIPEP	4.90	5.55	4.25
FZD7	4.90	5.39	4.59
TMEM115	4.90	5.17	4.38
LHFPL5	4.90	5.00	4.68
PRY	4.90	5.46	4.50
OTTHUMG00000165882	4.90	5.55	4.16
BATF	4.90	5.13	4.54
ALOXE3	4.90	5.46	4.47
AFAP1	4.90	5.17	4.68
OTTHUMG00000166389	4.90	5.54	4.31
SLC38A5	4.90	5.80	3.75
PTX4	4.90	5.48	4.57
ZFAND4	4.90	5.54	4.34
OTTHUMG00000031808	4.90	5.35	4.38
SGSM1	4.90	5.24	4.56
SLC37A4	4.90	5.33	4.64
PI3	4.90	5.26	4.67
OTTHUMG00000013297	4.90	5.44	4.43
C15orf37	4.90	5.41	4.57
DEFB4B	4.90	5.60	3.69
NNAT	4.90	5.40	4.07
LOC100130027	4.90	5.29	4.39
LOC100128881	4.90	5.11	4.60
FAM3A	4.90	5.18	4.58
GPR123	4.90	5.16	4.76
LGR6	4.90	5.37	4.29
ZNF496	4.90	5.01	4.74
CDKN2C	4.90	5.84	4.17
CASP6	4.90	5.19	4.15
PPP1R1B	4.90	5.18	4.48
SLC2A1-AS1	4.90	5.17	4.56
ALKBH5	4.90	5.35	4.38

UGDH-AS1	4.90	5.36	4.46
NUDT2	4.90	5.16	4.47
ANO7	4.90	5.05	4.65
OTTHUMG00000019257	4.90	5.54	4.50
DMD-AS1	4.90	5.50	4.23
LOC100287728	4.90	5.54	4.30
ST3GAL6	4.90	5.11	4.71
KIFC3	4.90	5.13	4.63
PRR9	4.90	5.45	4.46
STAMBP	4.90	5.52	4.68
NAT8	4.90	5.33	4.19
ASB9	4.90	5.69	4.47
OR6K2	4.90	5.60	3.74
BRPF1	4.90	5.14	4.65
STK11	4.90	5.38	4.54
TNFRSF10B	4.90	5.12	4.53
REPIN1	4.90	5.13	4.41
FAM138A	4.90	5.63	4.28
KLF17	4.90	5.20	4.74
CD177	4.90	5.17	4.73
LCN6	4.90	5.18	4.70
BMP3	4.90	5.62	4.33
SRRM3	4.89	5.21	4.59
OTTHUMG00000168051	4.89	5.21	4.51
SLC2A11	4.89	5.25	4.41
MIR4659B	4.89	5.61	3.41
TESC	4.89	5.23	4.48
STRIP2	4.89	5.61	4.21
MCOLN3	4.89	5.69	4.14
DHCR24	4.89	5.36	4.18
TMEM119	4.89	5.31	4.30
LOC100506319	4.89	5.63	4.48
IGLV2-18	4.89	5.82	4.30
DYRK1B	4.89	5.21	4.55
LOC100128343	4.89	5.37	4.72
VWC2	4.89	5.39	4.55
GNPDA1	4.89	5.31	4.58
OTTHUMG00000086699	4.89	5.74	4.41
SNORA65	4.89	5.24	4.29
MLYCD	4.89	5.31	4.49
KLK9	4.89	5.27	4.56
TGM2	4.89	5.49	4.46
LINC00516	4.89	5.31	4.60
KCNK5	4.89	5.47	4.27
NR1H3	4.89	5.44	4.54
LOC100287042	4.89	5.32	4.29
OPRL1	4.89	5.21	4.67
LOC101060008	4.89	6.00	4.03
LACC1	4.89	5.44	4.55

PROZ	4.89	5.16	4.61
FLJ38723	4.89	5.51	4.47
SUB1	4.89	5.03	4.62
NAPSA	4.89	5.62	4.20
CPEB1	4.89	5.39	4.27
KHDRBS3	4.89	5.44	4.44
C1QL4	4.89	5.24	4.55
SPATA6L	4.89	5.66	4.44
HNRNPA1P33	4.89	6.16	4.03
SELK	4.89	5.12	4.53
SLC13A4	4.89	5.14	4.56
OR8S1	4.89	5.31	4.58
CALML4	4.89	5.34	4.20
ZFAT	4.89	5.24	4.47
CYP2G1P	4.89	5.34	4.64
GATA6	4.89	5.28	4.44
BTNL10	4.89	5.16	4.37
SLC22A11	4.89	5.36	4.48
C1orf122	4.89	5.35	4.32
FAM90A20P	4.89	5.28	4.52
TRIM16	4.89	5.44	4.39
NOA1	4.89	5.61	4.36
CAMK2A	4.89	5.27	4.45
FAR2	4.89	5.32	4.54
TSTD1	4.89	5.55	4.21
RCOR2	4.89	5.42	4.56
PRR11	4.89	5.77	3.89
NOS2P3	4.89	5.33	4.62
ARMC2	4.89	5.40	4.34
GPR180	4.89	5.45	4.49
KLHL17	4.89	5.14	4.57
KLHDC9	4.89	5.41	4.39
ADCY10P1	4.89	5.24	4.50
LOC440040	4.89	5.77	4.25
KRT16	4.89	5.24	4.46
ZBTB49	4.89	5.15	4.45
SLC35D3	4.89	5.28	4.37
OTOP1	4.89	5.22	4.68
KCNN1	4.89	5.35	4.42
CELSR3	4.89	5.24	4.40
SHC4	4.89	5.33	4.48
PDCL	4.89	5.31	4.34
ZBED4	4.89	5.26	4.53
ZNF823	4.89	5.81	3.23
FAM81A	4.89	5.24	4.40
LOC100128310	4.89	5.66	4.31
MMEL1	4.89	5.29	4.45
RPS6KL1	4.89	5.40	4.04
CDHR1	4.89	5.35	4.64

ST6GALNAC2	4.89	5.30	4.35
LINC00263	4.89	6.01	4.31
POU2F3	4.89	5.28	4.58
OTTHUMG00000161960	4.89	5.09	4.60
ZNF491	4.89	5.49	4.30
FASTKD2	4.89	5.30	4.54
ARF5	4.89	5.42	4.30
ASPM	4.89	6.01	3.61
GALNT9	4.89	5.54	4.53
GSKIP	4.89	5.18	4.46
SLC6A7	4.88	5.20	4.52
OTTHUMG00000172914	4.88	5.29	4.59
EARS2	4.88	5.17	4.67
WDR3	4.88	5.37	4.31
TMEM222	4.88	5.37	4.49
ZSCAN10	4.88	5.39	4.53
TTI2	4.88	5.35	4.63
DNM3	4.88	5.25	4.13
VTI1A	4.88	5.20	4.38
POU3F3	4.88	5.15	4.49
LOC100507024	4.88	5.27	4.54
TUSC2	4.88	5.19	4.42
MS4A14	4.88	5.87	4.36
NT5C	4.88	5.48	4.42
NPAP1	4.88	5.27	4.29
TIFA	4.88	5.31	3.94
BPIFA2	4.88	5.27	4.50
OTTHUMG00000151400	4.88	5.25	4.37
PTGES2	4.88	5.20	4.59
KCNA1	4.88	5.49	4.29
OR56A3	4.88	5.75	4.21
B3GNT5	4.88	5.07	4.54
TMEM120B	4.88	5.41	4.47
HAUS6	4.88	5.56	4.55
ABCC5-AS1	4.88	5.51	4.26
SIX4	4.88	5.35	4.34
XYLB	4.88	5.30	4.40
NFKBIL1	4.88	5.36	4.64
TMEM5	4.88	5.19	4.50
KRTAP9-1	4.88	5.35	4.45
STAG3L4	4.88	5.33	4.28
ARSA	4.88	5.55	4.15
LFNG	4.88	5.29	4.56
GPM6B	4.88	5.42	4.52
ZNF275	4.88	5.24	4.53
ERN2	4.88	5.34	4.64
CMTM6	4.88	5.47	3.78
OTTHUMG00000164708	4.88	5.37	4.27
BIN3	4.88	5.26	4.47

IQCK	4.88	5.70	4.10
HID1	4.88	5.38	4.29
LRRC20	4.88	5.25	4.54
RIIAD1	4.88	5.37	4.46
LRP5L	4.88	5.25	4.54
ZNF879	4.88	5.30	4.18
PARD6G-AS1	4.88	5.46	4.28
CARD6	4.88	5.44	4.55
LHFPL4	4.88	5.24	4.45
ADRA1D	4.88	5.41	4.61
OTTHUMG00000037527	4.88	5.26	4.54
CDCA8	4.88	5.28	4.52
BMP8B	4.88	5.47	4.10
SELP	4.88	5.98	3.38
EYA2	4.88	5.23	4.57
NXPE3	4.88	5.36	4.04
MIR4330	4.88	5.71	3.96
C22orf39	4.88	5.08	4.51
ALAD	4.88	5.15	4.56
SOX15	4.88	5.07	4.60
DNAJC27	4.88	5.08	4.51
ATP6V1B1	4.88	5.22	4.53
LOC100128198	4.88	5.11	4.61
ZNF775	4.88	5.19	4.41
OTTHUMG00000155735	4.88	5.34	4.59
ANKRD54	4.88	5.54	4.61
FAM73B	4.88	5.52	4.47
CAMK2B	4.88	5.08	4.63
COL22A1	4.88	5.28	4.56
ZNF2	4.88	5.32	4.49
LOC100130430	4.88	5.43	4.63
FAM58BP	4.88	5.50	4.48
PI4K2A	4.88	5.41	3.84
KRT36	4.88	5.17	4.56
SMAD5-AS1	4.88	5.29	4.55
FAH	4.88	5.47	4.37
ALG6	4.88	5.32	4.49
FAM177B	4.88	5.50	4.56
ZNF511	4.88	5.24	4.27
EBF2	4.87	5.67	3.96
OTTHUMG00000038015	4.87	5.06	4.48
XAGE1A	4.87	5.30	4.49
FAM171A2	4.87	5.48	4.47
PSIMCT-1	4.87	5.32	4.38
GPD2	4.87	5.17	4.39
ARHGAP33	4.87	5.44	4.41
CORO6	4.87	5.21	4.44
PAQR3	4.87	5.50	4.20
F10	4.87	5.61	4.17

GGTA1P	4.87	5.70	3.83
MEPE	4.87	6.75	3.50
LOC642846	4.87	5.21	4.39
OR2G6	4.87	5.37	4.45
GPR20	4.87	5.33	4.44
PPP2R4	4.87	5.26	4.40
CPS1	4.87	5.15	4.61
OTTHUMG00000161256	4.87	5.58	4.46
SLC25A20	4.87	5.30	4.50
LINC00607	4.87	4.98	4.68
SYCN	4.87	5.29	4.56
GSTA7P	4.87	5.45	4.26
OTTHUMG00000161522	4.87	5.23	4.47
LOC100506358	4.87	5.16	4.34
POLE	4.87	5.13	4.61
OCIAD1-AS1	4.87	5.55	4.19
SMCR5	4.87	5.25	4.20
HIST2H3D	4.87	5.12	4.39
ZFP30	4.87	5.20	4.47
LEMD2	4.87	5.22	4.47
CCDC18	4.87	5.56	4.10
ARHGEF16	4.87	5.12	4.53
SHANK3	4.87	5.57	4.23
HRH2	4.87	5.21	4.32
ATP2B2	4.87	5.45	4.12
OTTHUMG00000162405	4.87	5.40	4.42
NTPCR	4.87	5.35	4.41
STARD13	4.87	5.31	4.44
SH3BP4	4.87	5.18	4.63
ARID3C	4.87	5.38	4.18
HOXA2	4.87	5.30	4.13
HDDC3	4.87	5.25	4.46
CES3	4.87	5.09	4.68
CCDC121	4.87	5.04	4.66
LHX1	4.87	5.31	4.57
LOC146513	4.87	5.53	4.29
IL17RB	4.87	5.63	3.98
BOLA3-AS1	4.87	5.24	4.23
AGL	4.87	5.33	4.24
MANEAL	4.87	5.29	4.49
LOC652276	4.87	5.14	4.60
LOC440461	4.87	5.32	4.24
LOC728819	4.87	5.26	4.14
NAALADL2	4.87	5.29	4.32
PYCRL	4.87	5.37	4.48
CYP2R1	4.87	5.60	4.49
TRIM6-TRIM34	4.87	5.18	4.63
LOC100132147	4.87	5.39	4.49
LOC100505664	4.87	5.15	4.59

FRA10AC1	4.87	5.11	4.24
ASCL5	4.87	5.42	4.46
CPNE9	4.87	5.35	4.60
CDH22	4.87	5.44	4.42
NXNL1	4.87	5.29	4.48
STRA6	4.87	5.10	4.65
SLC30A3	4.87	4.99	4.73
CCL16	4.87	5.62	4.37
FMO5	4.87	5.02	4.56
NOXO1	4.87	5.34	4.40
UNC5C	4.86	5.48	4.25
EPN3	4.86	5.15	4.50
SFTPA2	4.86	5.16	4.45
OTTHUMG00000017366	4.86	5.31	4.42
KRT18	4.86	5.26	4.30
DNAJC17	4.86	5.02	4.64
TNFAIP3	4.86	5.12	4.60
CKM	4.86	5.17	4.70
RAPSN	4.86	5.24	4.25
P2RX3	4.86	5.14	4.62
GRIN3B	4.86	5.08	4.56
NCAM1-AS1	4.86	5.21	4.51
GPR68	4.86	5.32	4.54
SH3BP2	4.86	5.16	4.75
GAB2	4.86	5.24	4.51
IGLV3-27	4.86	5.46	4.04
FST	4.86	5.33	4.43
HAAO	4.86	5.20	4.11
ACADL	4.86	5.82	4.14
C16orf86	4.86	5.38	4.50
LOC100506100	4.86	5.18	4.56
IFIH1	4.86	5.75	4.29
FLAD1	4.86	5.16	4.63
NLN	4.86	5.22	4.48
FBXW7	4.86	5.19	4.38
CA3	4.86	5.43	4.46
CCDC116	4.86	5.16	4.61
PITX1	4.86	5.06	4.70
OTTHUMG00000183534	4.86	5.46	4.21
KRT78	4.86	5.43	4.24
AP5M1	4.86	5.18	4.49
DBH	4.86	5.30	4.47
LOC728485	4.86	5.34	4.51
PLD2	4.86	5.44	4.51
E2F1	4.86	5.09	4.40
SKAP2	4.86	5.26	4.55
NTM-IT3	4.86	5.27	4.18
RPUSD4	4.86	5.27	4.50
SRC	4.86	5.32	4.33

MLANA	4.86	5.27	4.38
EDNRB	4.86	5.32	4.36
CCM2	4.86	5.14	4.36
CCDC170	4.86	5.29	4.52
ROR1	4.86	5.89	4.13
PPP1R3B	4.86	5.20	4.25
SLC9A5	4.86	5.12	4.60
SEMA6C	4.86	5.18	4.49
C3AR1	4.86	6.01	3.50
PYGO2	4.86	5.24	3.82
GLB1L2	4.86	5.58	4.40
HYDIN	4.86	5.11	4.38
ZEB2-AS1	4.86	5.22	4.18
SYPL2	4.86	5.21	4.36
KIAA1009	4.86	5.28	4.07
KRTAP5-9	4.86	5.81	4.04
FAM98B	4.86	5.26	4.49
CBX6	4.86	5.22	4.29
ZEB1-AS1	4.86	5.27	4.34
OTTHUMG00000160309	4.86	5.29	4.34
TRMT44	4.86	5.18	4.50
GADL1	4.86	5.10	4.63
P4HTM	4.86	5.20	4.46
OTTHUMG00000067216	4.86	5.63	4.30
OTTHUMG00000164253	4.86	5.44	4.15
MYBPC2	4.86	5.24	4.49
HOXA13	4.86	5.30	4.60
PIANP	4.86	5.20	4.44
NANP	4.86	5.14	4.55
SNORD113-1	4.86	6.76	3.04
TRPV3	4.86	5.17	4.63
CACFD1	4.86	5.33	4.36
CECR5	4.86	5.22	4.37
CAMK2G	4.86	5.47	4.43
TOB1-AS1	4.86	5.19	4.56
MICU2	4.86	5.40	3.87
FABP6	4.86	5.25	4.25
POM121L10P	4.86	5.51	4.33
OTTHUMG00000162131	4.86	5.63	4.34
TSPEAR-AS1	4.86	5.09	4.67
CSNK2A3	4.85	6.06	3.91
ANKRD46	4.85	5.11	4.63
LINC00696	4.85	5.41	4.53
ENTPD3	4.85	5.09	4.55
PCSK5	4.85	5.07	4.57
UCN2	4.85	5.24	4.09
TRDV3	4.85	5.29	4.33
CPSF4	4.85	5.31	4.55
ZNF613	4.85	5.38	4.40

LINC00597	4.85	5.61	4.13
CHRNA10	4.85	5.43	4.28
MFSD2A	4.85	5.50	4.21
PLGLA	4.85	5.25	4.30
OTTHUMG00000032718	4.85	5.36	4.43
NR4A2	4.85	5.48	4.31
ANGEL1	4.85	5.30	4.49
IKZF2	4.85	5.28	4.54
INS-IGF2	4.85	5.27	4.40
OC90	4.85	5.55	4.36
OTTHUMG00000160584	4.85	5.32	4.56
SPATA12	4.85	5.27	4.47
USP51	4.85	5.31	4.46
TMEM209	4.85	5.20	4.54
ADO	4.85	5.39	4.30
MIR149	4.85	5.51	4.37
VIL1	4.85	5.15	4.48
OTTHUMG00000161724	4.85	5.29	4.38
OTTHUMG00000151360	4.85	5.32	4.30
C17orf70	4.85	5.09	4.45
OR2G3	4.85	5.40	3.84
OLFML3	4.85	5.65	4.23
SLC43A2	4.85	5.43	4.43
ASPHD2	4.85	5.19	4.64
PLK2	4.85	5.74	3.78
EPHA1	4.85	5.01	4.60
PXMP4	4.85	5.32	4.33
MTBP	4.85	5.20	4.30
C1orf220	4.85	5.13	4.63
GSAP	4.85	5.03	4.59
OTTHUMG00000168002	4.85	5.17	4.65
OTTHUMG00000031689	4.85	5.43	4.11
NR2F2	4.85	6.55	3.55
ADCY3	4.85	5.30	4.45
C20orf27	4.85	5.31	4.44
WNK2	4.85	5.36	4.26
RAPGEF4	4.85	5.13	4.51
MAR1	4.85	5.36	4.34
LHX3	4.85	5.25	4.19
ADORA2A	4.85	5.08	4.65
SMPD2	4.85	5.34	4.35
PRPS1L1	4.85	5.29	4.37
LOC100509780	4.85	5.35	4.41
RNA5SP218	4.85	5.44	4.00
PAX8	4.85	5.44	4.59
PFN2	4.85	5.37	4.37
RELB	4.85	5.30	4.43
MECOM	4.85	5.25	4.28
OTTHUMG00000161604	4.85	5.24	4.42

TTPAL	4.85	5.26	4.21
RLN3	4.85	5.60	4.42
GLTSCR1	4.85	5.19	4.28
ALPP	4.85	5.44	4.34
UBAC1	4.85	5.21	4.15
LINC00592	4.85	5.42	4.44
AP3M2	4.85	5.31	4.49
PSD2	4.85	5.30	4.49
IGSF9	4.85	5.24	4.64
TRH	4.85	5.43	4.47
STEAP3-AS1	4.85	5.51	4.47
MIR3148	4.85	6.06	4.36
OTTHUMG00000018038	4.85	5.18	4.58
DGKK	4.85	5.34	4.16
LINC00905	4.85	5.32	4.48
ALOX12P2	4.85	5.37	4.36
F2	4.85	5.22	4.55
LRRC6	4.85	5.15	4.52
MOXD1	4.85	5.45	4.35
OTTHUMG00000020419	4.85	5.44	4.40
PABPC1L	4.85	5.27	4.26
PEX26	4.85	5.23	4.47
TLX1NB	4.84	5.33	4.18
KEAP1	4.84	5.55	4.13
SLFN12	4.84	5.37	4.51
CTRB1	4.84	5.40	4.34
LOC100505923	4.84	5.06	4.55
PRRG1	4.84	5.10	4.36
LOC731424	4.84	5.05	4.63
STK17A	4.84	5.34	4.42
ITGA3	4.84	5.09	4.69
C16orf92	4.84	5.34	4.59
CDC42EP2	4.84	5.06	4.42
OTTHUMG00000162458	4.84	5.65	4.43
PLGRKT	4.84	5.19	4.51
WRB	4.84	5.04	4.42
NGFR	4.84	5.13	4.55
DDC	4.84	5.10	4.59
MAP3K13	4.84	5.19	4.52
CHAF1B	4.84	5.32	4.52
KCNC4	4.84	5.38	4.33
ZNF727	4.84	5.23	4.26
VPS51	4.84	5.32	4.34
MIR4505	4.84	5.44	4.63
CAV2	4.84	5.12	4.48
OTTHUMG00000014661	4.84	5.37	4.37
CSH1	4.84	5.50	4.16
ODF3B	4.84	5.27	4.41
TSC22D3	4.84	5.37	4.21

PPAT	4.84	5.21	4.39
TOB2P1	4.84	5.41	4.35
CRYBB2P1	4.84	5.90	4.06
TAS1R1	4.84	5.31	4.54
WDR38	4.84	5.31	4.21
PAX7	4.84	5.24	4.43
ICAM5	4.84	5.12	4.56
CHCHD4	4.84	5.27	4.59
LINC00663	4.84	5.27	4.26
RAE1	4.84	5.24	4.57
MIR371B	4.84	5.66	3.99
ATP6V0A2	4.84	5.54	3.84
FAM64A	4.84	5.44	4.18
SAG	4.84	5.51	4.57
OR2A7	4.84	5.44	4.13
POC1A	4.84	5.26	4.35
OTTHUMG00000171691	4.84	5.63	4.37
PGAM5	4.84	5.01	4.52
TCF23	4.84	5.32	4.52
KIAA1244	4.84	5.22	4.51
KIRREL2	4.84	5.13	4.41
PLEKHB1	4.84	5.59	4.52
GGT7	4.84	5.15	4.57
OTTHUMG00000163337	4.84	5.28	4.10
HAGHL	4.84	5.12	4.61
PNPO	4.84	5.10	4.38
BNIPL	4.84	5.22	4.61
PAFAH1B3	4.84	5.33	4.48
ZNF485	4.84	5.34	4.38
ANKMY1	4.84	5.26	4.51
CMSS1	4.84	5.18	4.58
ATG4C	4.84	5.36	4.58
FXYD7	4.84	5.03	4.51
CAMP	4.84	5.17	4.53
GIT1	4.84	5.16	4.47
GAS2L3	4.84	5.31	4.42
RPP25	4.84	5.27	4.37
LOC100129936	4.84	5.65	4.21
FRMD8	4.84	5.29	4.10
VWA7	4.84	5.04	4.45
LOC148413	4.84	5.08	4.50
HSD3BP4	4.84	5.43	4.31
KIAA0101	4.84	5.13	4.43
TJP2	4.84	5.22	4.51
SFTA2	4.84	5.21	4.49
OTTHUMG00000159990	4.84	5.19	4.57
SUPT20HL1	4.84	5.53	4.33
OTTHUMG00000015119	4.84	5.21	4.47
RNA5SP91	4.84	5.51	4.09

TPD52	4.84	5.25	4.45
ARSJ	4.84	5.25	4.33
VTCN1	4.84	5.42	4.40
SCUBE2	4.84	5.20	4.47
TOMM40L	4.84	5.12	4.30
MIR597	4.84	5.39	3.90
TGFA	4.84	5.03	4.34
OR6W1P	4.84	5.40	4.55
MICALL1	4.84	5.09	4.42
BARX2	4.84	5.61	4.28
NTSR1	4.84	5.10	4.57
CEACAM3	4.84	5.45	4.50
OTTHUMG00000166306	4.84	5.13	4.06
OTTHUMG00000163804	4.84	5.15	4.55
OTTHUMG00000015092	4.84	5.25	4.36
CHN1	4.84	5.60	4.40
OR5B21	4.84	5.40	4.34
MEIS1	4.84	5.16	4.61
TGM6	4.84	5.30	4.33
DUOXA1	4.84	5.15	4.61
MFSD4	4.84	5.08	4.46
GAGE10	4.84	5.62	3.61
MAR3	4.84	5.40	4.29
LSM11	4.84	5.11	4.28
GFI1B	4.83	5.49	4.31
MIR212	4.83	5.27	4.28
KRTAP20-2	4.83	5.23	4.49
ZG16B	4.83	5.34	4.51
SNORD99	4.83	5.93	2.93
LOC100289455	4.83	5.29	4.36
DIO3OS	4.83	5.14	4.49
TOLLIP	4.83	5.31	4.35
LOC729444	4.83	5.74	4.50
OTTHUMG00000153335	4.83	5.16	4.47
SCARA5	4.83	5.31	4.08
SAE1	4.83	5.22	3.90
ZNF625-ZNF20	4.83	5.13	4.47
ARHGAP30	4.83	5.25	4.36
P2RY14	4.83	5.54	4.41
ZNF486	4.83	5.46	4.14
OSBPL6	4.83	5.69	4.34
TRAF2	4.83	5.23	4.46
OTTHUMG00000018245	4.83	5.50	3.93
PLA2G12B	4.83	5.11	4.68
RNA5SP181	4.83	5.42	4.14
CD19	4.83	5.14	4.48
ALX3	4.83	5.26	4.45
CRY1	4.83	5.69	4.27
DPH6	4.83	5.32	4.33

OR5L2	4.83	5.80	3.22
PROC	4.83	5.03	4.38
BACH2	4.83	5.27	4.39
SERHL	4.83	5.63	4.26
CACNG3	4.83	5.21	4.42
LINC00673	4.83	5.11	4.56
LOC728730	4.83	5.19	4.53
POGLUT1	4.83	5.36	4.23
RAET1E	4.83	5.24	4.58
RNF151	4.83	5.23	4.27
SIX3-AS1	4.83	5.13	4.59
DOCK2	4.83	6.02	4.18
OR2T10	4.83	5.37	4.62
OTTHUMG00000155213	4.83	5.57	4.30
MYL12B	4.83	5.17	4.56
ZNF551	4.83	5.11	4.59
VWA5B2	4.83	5.41	4.52
MIR4429	4.83	5.60	4.05
CKAP4	4.83	5.24	4.37
LINC00334	4.83	5.23	4.45
MIR766	4.83	5.40	4.22
MRVI1	4.83	5.45	4.19
CYP11B1	4.83	5.21	4.29
PADI6	4.83	5.35	4.34
PLCL2	4.83	5.22	4.41
OTTHUMG00000154972	4.83	5.27	4.52
APOBEC3G	4.83	5.29	4.27
APOBR	4.83	5.35	4.36
EXOC6	4.83	5.24	3.80
OTTHUMG00000154037	4.83	5.29	4.16
ZBTB12	4.83	5.08	4.54
TBXAS1	4.83	5.16	4.38
BLCAP	4.83	5.07	4.63
C3orf35	4.83	5.36	4.49
LOC100379224	4.83	5.01	4.43
C17orf72	4.83	5.29	4.30
WFS1	4.83	5.15	4.55
RNF121	4.83	5.37	4.28
DLGAP1	4.83	5.30	4.45
LOC100129069	4.83	5.42	4.39
NKPD1	4.83	5.19	4.61
DES	4.83	5.17	4.49
C3P1	4.83	5.17	4.60
PDE6B	4.83	5.13	4.53
LOC100996457	4.83	5.26	4.52
C17orf98	4.83	5.22	4.34
GNB1L	4.83	5.12	4.64
HEYL	4.83	5.46	4.35
KRT1	4.83	5.76	4.33

LURAP1	4.83	5.12	4.57
PMEL	4.83	5.10	4.44
HNRNPA3	4.83	5.06	4.54
LOC284014	4.83	5.37	4.43
LOC728196	4.83	5.38	4.29
SUOX	4.83	5.05	4.47
E2F5	4.83	5.34	4.42
LOC100506713	4.83	5.14	4.51
SPATA5L1	4.83	4.92	4.54
ABCC3	4.83	6.02	4.28
HUNK	4.83	5.75	4.32
OTTHUMG00000168064	4.83	5.84	4.24
LOC257396	4.83	5.11	4.63
ENPEP	4.83	5.74	3.95
LY6D	4.83	5.16	4.54
SDR42E1	4.83	5.15	4.56
ZNF691	4.82	5.23	4.58
OTTHUMG00000019004	4.82	5.37	4.49
CD1D	4.82	5.31	4.56
SYTL3	4.82	5.20	4.54
CMBL	4.82	5.04	4.46
LOC100130691	4.82	5.39	4.01
HSPA14	4.82	5.30	3.96
HLA-B	4.82	5.65	3.73
ZNF821	4.82	5.14	4.59
PIK3CG	4.82	5.24	4.40
N6AMT1	4.82	5.08	4.41
MAOA	4.82	5.61	4.38
OTTHUMG00000167601	4.82	5.38	4.34
PPP1R37	4.82	5.11	4.27
EBLN2	4.82	5.30	4.49
LRRC28	4.82	5.10	4.35
ADCYAP1R1	4.82	5.32	4.57
LONRF2	4.82	5.33	4.19
OTTHUMG00000171913	4.82	5.40	4.50
NOL6	4.82	5.06	4.50
CLEC4A	4.82	5.60	3.89
CENPO	4.82	5.10	4.47
CCDC88C	4.82	5.14	4.34
PRDM1	4.82	5.12	4.57
LOC653712	4.82	5.08	4.58
KCND3	4.82	5.47	4.26
PIK3CB	4.82	5.19	4.45
CSTF2	4.82	4.99	4.61
C1QTNF3-AMACR	4.82	5.88	3.93
BTN3A1	4.82	5.10	4.46
OTTHUMG00000163395	4.82	5.61	3.61
ALOX12	4.82	5.13	4.33
OTTHUMG00000179326	4.82	5.15	4.19

NEIL1	4.82	5.10	4.49
TOP3B	4.82	5.10	4.56
COL25A1	4.82	5.26	4.04
GOT1	4.82	5.03	4.65
TNKS2-AS1	4.82	5.10	4.50
CLMN	4.82	5.12	4.43
LOC729852	4.82	5.22	4.64
CNKSR3	4.82	5.23	4.50
ZNF365	4.82	5.27	4.44
PSEN2	4.82	5.11	4.56
ST3GAL1	4.82	5.19	4.51
SFTPB	4.82	5.26	4.39
FAM195A	4.82	5.39	4.30
ACR	4.82	5.38	3.88
SRSF10	4.82	5.36	4.13
ZNRF2	4.82	5.21	4.53
BNIP1	4.82	5.13	4.42
DOC2A	4.82	5.16	4.48
DPY19L1P1	4.82	5.35	4.36
KCNE4	4.82	5.46	4.34
TTYH2	4.82	5.15	4.45
RABEPK	4.82	5.30	4.46
GPR50	4.82	5.33	4.52
OTTHUMG00000164199	4.82	5.08	4.31
ADIPOR2	4.82	5.27	4.48
SLC35C1	4.82	5.42	4.36
RNF40	4.82	5.16	4.37
KIAA1467	4.82	5.25	4.45
ANXA2R	4.82	5.30	4.60
OTTHUMG00000019202	4.82	5.32	4.20
TCN2	4.82	5.26	4.37
ZBED3	4.82	5.21	4.26
MAPKBP1	4.82	5.06	4.64
LINC00176	4.82	5.19	4.48
AKR7A2	4.82	5.40	4.04
RASSF2	4.82	5.21	4.48
USP46	4.82	5.14	4.18
TBX6	4.82	5.19	4.43
PRR22	4.82	5.11	4.50
RASGEF1C	4.82	5.16	4.34
LOC100506105	4.82	5.15	4.24
OTTHUMG00000153173	4.82	5.22	4.58
ARL6	4.82	5.05	4.24
LRRN1	4.82	5.63	4.22
LOC100289580	4.82	5.29	4.44
ARHGEF26	4.82	5.18	4.28
ARNTL	4.82	5.40	3.95
SERPIND1	4.82	5.06	4.28
RNA5SP19	4.82	5.13	4.26

COL4A2-AS2	4.82	5.34	4.44
MIR3192	4.82	5.33	4.04
SPOCD1	4.82	5.16	4.58
ZNF136	4.82	5.18	4.36
DENND1C	4.82	5.17	4.50
WIF1	4.82	6.43	3.63
SHISA9	4.82	5.13	4.20
ATG4B	4.82	5.29	4.58
MSX1	4.82	5.16	4.64
TLR6	4.82	5.46	4.31
FUT2	4.82	5.16	4.53
PFKFB1	4.82	5.43	4.29
TMEM117	4.82	5.16	4.38
LOC100132831	4.82	5.65	4.35
GMCL1	4.82	5.41	4.33
OTTHUMG00000155027	4.82	5.32	4.45
CATSPER2	4.82	5.27	4.39
LOC101059921	4.82	5.56	4.26
WHAMM	4.82	5.18	4.47
ONECUT3	4.81	5.46	4.32
NACC1	4.81	5.07	3.93
FAM132B	4.81	5.04	4.40
OTTHUMG00000041463	4.81	5.38	4.43
CCDC115	4.81	5.14	4.53
WDR81	4.81	5.06	4.45
ADAMTS12	4.81	6.00	4.14
ANAPC2	4.81	5.05	4.56
SPRY3	4.81	5.11	4.42
ARSK	4.81	5.42	4.21
TP73-AS1	4.81	5.19	4.24
PIGH	4.81	5.33	4.31
OTTHUMG00000179638	4.81	5.38	4.13
ARHGAP40	4.81	5.50	4.43
RGS9	4.81	5.48	4.36
CCZ1	4.81	5.51	4.32
LOC339803	4.81	5.08	4.47
MAP3K10	4.81	5.32	4.41
UNC119B	4.81	5.34	4.47
DIO2	4.81	5.36	4.20
SKIDA1	4.81	5.10	4.55
LOC390705	4.81	5.16	4.58
SNORD75	4.81	5.71	3.95
ZNF134	4.81	5.17	4.28
KANSL1-AS1	4.81	5.79	4.28
RBM20	4.81	5.12	4.53
SYT13	4.81	5.76	4.27
KIAA1549L	4.81	5.21	4.20
OTTHUMG00000150002	4.81	5.13	4.50
SNORD101	4.81	5.55	3.23

HARBI1	4.81	5.11	4.50
WWC1	4.81	5.23	4.37
FANK1-AS1	4.81	5.48	4.31
GINS3	4.81	5.44	4.53
PCDH1	4.81	5.46	4.07
ZNF155	4.81	5.08	4.60
PACRGL	4.81	5.25	4.33
LOC286178	4.81	5.03	4.64
ZNF534	4.81	5.31	4.26
DNMT3A	4.81	5.04	4.58
ISG20	4.81	5.29	4.55
MUL1	4.81	5.11	4.44
LYPD3	4.81	5.23	4.32
ZNF804B	4.81	5.26	4.43
TIGD7	4.81	5.21	4.40
MAR4	4.81	5.25	4.49
MED11	4.81	5.56	4.20
ZNF550	4.81	5.20	4.39
ZNF394	4.81	5.13	4.21
LOC100506321	4.81	5.15	4.21
SATL1	4.81	5.19	4.47
FRG2B	4.81	5.19	4.33
OTTHUMG00000004419	4.81	5.10	4.58
C2orf54	4.81	5.37	4.43
TMEM184A	4.81	5.07	4.51
TERT	4.81	5.17	4.35
LINC00452	4.81	5.20	4.52
RHBDD3	4.81	5.25	4.27
QTRTD1	4.81	5.09	4.50
ZNF480	4.81	5.23	4.38
LOC400752	4.81	5.36	4.39
LMLN	4.81	5.33	4.20
CACNA1F	4.81	5.18	4.37
CPEB3	4.81	5.38	4.33
PPP1R14A	4.81	5.57	3.99
RBPJL	4.81	5.07	4.33
SMARCA5-AS1	4.81	5.11	4.50
CPLX1	4.81	5.32	4.41
ZNF786	4.81	5.36	4.51
CNTN2	4.81	5.14	4.42
SPIB	4.81	5.01	4.60
FBXL6	4.81	5.13	4.44
FGF8	4.81	5.26	4.48
ALG10	4.81	5.38	4.32
LDLRAD1	4.81	5.11	4.50
LOC145474	4.81	5.43	4.05
DFFA	4.81	5.12	4.51
RPL37A	4.81	5.02	4.46
KRTAP4-1	4.81	5.42	4.33

CUEDC1	4.81	5.07	4.26
PTPN7	4.81	5.39	4.29
TMEM128	4.81	5.08	4.38
UBAP1L	4.81	5.26	4.40
RNA5SP134	4.81	5.92	3.85
PLA2G1B	4.81	5.30	4.43
GALE	4.81	5.26	4.43
PLA2G2C	4.81	5.34	4.59
OTTHUMG00000168217	4.81	5.21	4.28
LOC399715	4.81	5.20	4.36
MFNG	4.81	5.23	4.03
CCM2L	4.81	5.36	4.34
TMEM132A	4.81	5.21	4.20
SETMAR	4.81	5.18	4.36
RPS6KA4	4.81	5.18	4.44
KRT17	4.81	5.57	4.22
ST6GAL2	4.81	5.90	4.24
IGHMBP2	4.80	5.38	4.56
FLJ20464	4.80	5.07	4.45
HPS1	4.80	5.23	4.53
DRD2	4.80	5.17	4.60
N4BP1	4.80	5.43	4.05
LRRC23	4.80	5.21	4.46
OTTHUMG00000168564	4.80	5.07	4.14
ZNF137P	4.80	5.44	4.10
GID4	4.80	5.22	4.53
TBC1D10B	4.80	5.12	4.41
CDH3	4.80	5.75	4.13
C9orf173	4.80	5.17	4.38
ORC5	4.80	5.24	4.48
TTC5	4.80	5.34	4.17
LRRC3DN	4.80	5.71	4.22
SMIM3	4.80	5.66	3.92
NRL	4.80	5.20	4.30
OTTHUMG00000165613	4.80	5.12	4.44
C2orf72	4.80	5.06	4.45
MYH16	4.80	5.15	4.27
OPRK1	4.80	5.20	4.46
LIG3	4.80	5.07	4.40
CCDC77	4.80	5.26	4.42
OTTHUMG00000168893	4.80	5.14	4.33
COG2	4.80	5.17	4.40
NPM2	4.80	5.15	4.56
TSPY4	4.80	5.75	3.88
CHCHD3	4.80	5.32	4.26
IL18	4.80	5.52	3.96
BUB1B	4.80	5.14	4.49
KCNS3	4.80	5.12	4.53
SLC25A23	4.80	5.08	4.40

LINC00087	4.80	5.28	3.93
MIR205	4.80	5.19	4.47
DFNB59	4.80	5.24	4.23
INTS4	4.80	5.32	4.23
C6orf132	4.80	5.19	4.46
LOC100287387	4.80	5.32	4.45
ANKRD44	4.80	5.37	4.44
PRND	4.80	5.16	4.57
MTCP1	4.80	5.41	4.53
ALPK3	4.80	5.41	4.28
ZNF252P	4.80	5.26	4.37
C16orf71	4.80	5.15	4.55
MINPP1	4.80	5.38	4.13
LINC00894	4.80	5.19	3.94
ZBTB18	4.80	5.28	4.48
LOC100996662	4.80	5.05	4.33
PNPLA7	4.80	5.13	4.48
RUNX3	4.80	5.28	4.44
TAAR5	4.80	5.60	4.01
LOC145837	4.80	5.27	4.56
RNF157	4.80	5.42	4.51
LOC100506035	4.80	5.23	4.19
PHOX2B	4.80	5.21	4.45
DEFB103A	4.80	5.15	4.12
OTTHUMG00000170115	4.80	5.39	4.34
SLC5A1	4.80	5.05	4.50
PRRT4	4.80	5.05	4.45
GEMIN8	4.80	5.26	4.11
GDF1	4.80	5.29	4.49
EPN2-AS1	4.80	5.45	4.43
TNS4	4.80	5.21	4.42
OTTHUMG00000013379	4.80	5.27	4.33
GHDC	4.80	5.20	4.39
OTTHUMG00000160236	4.80	5.53	4.01
KCTD4	4.80	5.13	4.49
PEF1	4.80	5.26	4.31
AURKC	4.80	5.33	4.48
CCDC34	4.80	5.16	4.46
LDLRAD2	4.80	5.12	4.10
GCH1	4.80	5.74	4.43
THUMPD2	4.80	5.43	4.40
OTTHUMG00000164832	4.80	5.37	4.36
IFI27L1	4.80	5.04	4.52
PARVB	4.80	5.03	4.46
PTCH2	4.80	5.23	4.03
ZNF347	4.80	5.06	4.20
MGAT2	4.80	5.34	3.75
ZCCHC3	4.80	5.12	4.51
C14orf166B	4.80	5.18	4.57

OTTHUMG00000172684	4.80	5.31	4.21
RSPH4A	4.80	5.14	4.49
ZNF726	4.80	5.10	4.42
MIR4534	4.80	5.54	3.77
MAEA	4.80	5.03	4.60
TNFRSF4	4.80	5.06	4.37
RABL3	4.80	5.49	4.17
KCNK4	4.80	5.23	4.47
PRMT3	4.80	5.41	3.99
GYLTL1B	4.80	5.13	4.37
OTTHUMG00000014182	4.80	5.67	4.48
OTTHUMG00000132354	4.80	5.08	4.32
GH2	4.80	5.37	3.78
SDK1	4.80	5.04	4.59
SDC3	4.80	5.51	4.25
WDR83	4.80	5.14	4.53
LOC100506674	4.80	5.14	4.35
FGF12-AS2	4.80	5.28	4.28
FBXO5	4.80	5.24	4.26
TPSAB1	4.79	5.39	4.11
GDF11	4.79	5.29	4.31
GPR162	4.79	5.05	4.36
TLDC1	4.79	5.49	4.23
AASDHPPT	4.79	5.27	4.39
OTTHUMG00000156735	4.79	5.19	4.34
TMEFF1	4.79	5.79	3.75
PRDM15	4.79	5.04	4.43
SPATA31A1	4.79	5.24	4.28
CEP41	4.79	5.10	4.49
CBLN2	4.79	5.34	4.44
C18orf21	4.79	5.32	4.08
PPL	4.79	5.40	4.33
MTHFSD	4.79	5.04	4.41
ZNF350	4.79	5.70	3.83
VDR	4.79	4.98	4.41
ZSCAN29	4.79	5.10	4.15
PCSK9	4.79	5.02	4.49
UBQLNL	4.79	5.09	4.27
MIR3150B	4.79	5.22	4.45
FAM213B	4.79	4.97	4.60
MMP15	4.79	5.11	4.37
C6orf223	4.79	5.12	4.24
FGFR1OP2	4.79	5.34	3.61
CLDN23	4.79	5.12	4.40
OTTHUMG00000155986	4.79	5.38	4.47
TNFRSF10C	4.79	5.17	4.44
SLC25A10	4.79	5.38	4.44
TRPC2	4.79	5.16	4.54
BTN2A2	4.79	5.56	4.50

ARTN	4.79	5.14	4.35
STK11IP	4.79	5.07	4.54
HP07349	4.79	5.10	4.49
POLD4	4.79	5.14	4.44
TADA1	4.79	5.20	4.25
HIST1H4D	4.79	5.37	3.94
GPLD1	4.79	5.22	4.31
DTNB	4.79	5.13	4.26
PDSS2	4.79	5.51	4.38
SLC38A9	4.79	5.14	4.41
OTTHUMG00000160577	4.79	5.18	4.30
DUSP26	4.79	5.48	4.40
DDX39B-AS1	4.79	5.08	4.38
LOC100129484	4.79	5.12	4.39
PRSS48	4.79	5.18	4.10
LOC284513	4.79	5.29	4.35
DHRS11	4.79	5.00	4.29
FKBPL	4.79	5.18	4.50
TRNP1	4.79	5.30	4.51
RPL32P3	4.79	4.96	4.27
CDK6	4.79	5.61	4.32
CROCCP3	4.79	5.18	4.16
CD27	4.79	5.13	4.42
CHAT	4.79	5.22	4.26
PVT1	4.79	5.12	4.45
DGAT2L7P	4.79	5.35	4.57
BET1L	4.79	5.42	4.24
PDP2	4.79	5.54	4.35
APBB1	4.79	5.05	4.48
GRM2	4.79	5.25	4.59
CORO1B	4.79	5.19	4.37
DUS2L	4.79	5.23	4.54
HILS1	4.79	5.30	4.26
FAM161B	4.79	5.10	4.50
LOC100507507	4.79	5.28	4.11
FOXD4L5	4.79	5.33	4.01
NKX2-5	4.79	5.24	4.42
C12orf5	4.79	5.17	4.33
OTTHUMG00000019170	4.79	5.40	4.29
SNORD114-31	4.79	6.43	3.40
MANEA	4.79	5.55	3.88
TAF1B	4.79	5.24	4.32
TMEM260	4.79	5.23	4.25
DDX59	4.79	5.12	4.22
ALPI	4.79	5.56	4.35
C14orf37	4.79	5.35	4.29
GGT6	4.79	5.18	4.43
TP53TG1	4.79	5.22	4.45
HOXA1	4.79	5.20	4.35

DENND2D	4.79	5.12	4.16
LOC375196	4.79	5.06	4.58
RBBP8	4.79	5.75	3.75
ASPHD1	4.79	5.20	4.49
INHA	4.79	5.14	4.52
GATS	4.79	5.16	4.32
XAGE3	4.79	5.23	4.25
HIF3A	4.79	5.22	4.39
CASK-AS1	4.79	5.85	3.94
TELO2	4.79	5.41	4.36
LOC727993	4.79	5.14	4.37
TAS2R14	4.79	5.33	3.95
ADAM28	4.79	5.28	4.28
COX7A2L	4.79	5.16	4.41
GRIP1	4.79	5.02	4.50
ADORA3	4.79	5.26	4.25
IQSEC2	4.79	5.14	4.44
SLC39A1	4.79	5.13	4.32
NAPA-AS1	4.79	4.96	4.51
PCSK6	4.78	5.34	4.29
LOC100289473	4.78	5.50	4.37
DHRS12	4.78	5.06	4.42
OTTHUMG00000155093	4.78	5.26	4.33
GCC1	4.78	5.01	4.38
C15orf56	4.78	5.07	4.24
MRPS36	4.78	5.27	3.97
UNC13A	4.78	5.11	4.29
NAPRT1	4.78	5.15	4.53
OTTHUMG00000168249	4.78	5.06	4.25
OTTHUMG00000166415	4.78	5.13	4.27
RASA2	4.78	5.36	3.95
H1FX-AS1	4.78	5.00	4.54
SMPDL3A	4.78	5.28	4.45
TMEM38A	4.78	5.55	4.36
DKFZp667F0711	4.78	5.13	4.26
XCL2	4.78	5.39	4.28
OPTC	4.78	5.28	4.23
C2CD4D	4.78	5.27	4.41
LOC101060308	4.78	5.22	4.31
C5orf54	4.78	5.53	3.50
LOC284023	4.78	5.16	4.46
PDE5A	4.78	5.33	3.92
SAAL1	4.78	5.07	4.53
GDAP1	4.78	5.44	4.25
CNTNAP3B	4.78	5.40	4.22
POLG2	4.78	5.31	3.75
WFDC3	4.78	5.45	3.91
GJC3	4.78	5.27	4.25
C22orf15	4.78	5.08	4.26

OR8B8	4.78	5.52	3.94
LOC100049716	4.78	5.17	4.28
IFT27	4.78	5.10	4.57
PUS3	4.78	5.35	4.34
SLFNL1	4.78	5.33	4.07
FLJ31183	4.78	5.25	4.40
UCMA	4.78	5.65	3.93
MIR449C	4.78	5.28	4.08
CHAF1A	4.78	5.07	4.50
OTTHUMG00000035181	4.78	5.09	4.45
OTTHUMG00000168304	4.78	5.62	3.89
HTR2A	4.78	5.50	4.19
AQP7	4.78	5.23	4.17
REEP6	4.78	5.08	4.55
SVILP1	4.78	5.13	4.13
SLC26A6	4.78	5.00	4.23
IQCH-AS1	4.78	5.13	4.48
PDZD3	4.78	5.15	4.51
SLC22A31	4.78	5.08	4.47
ASH1L-AS1	4.78	5.16	4.34
LMAN2L	4.78	5.55	3.71
LOC100505716	4.78	4.96	4.43
TRBV6-9	4.78	5.26	4.35
OTTHUMG00000168645	4.78	5.37	4.09
CCDC155	4.78	5.42	4.21
BLID	4.78	5.49	4.00
MADCAM1	4.78	5.26	4.36
LBP	4.78	5.76	4.14
CRMP1	4.78	5.05	4.58
C16orf87	4.78	5.53	4.34
CDKN2AIPNL	4.78	5.09	4.43
KIAA0930	4.78	5.12	4.03
CHMP4C	4.78	5.31	4.29
VMAC	4.78	5.21	4.30
UVSSA	4.78	5.13	4.28
RRN3P3	4.78	5.42	4.35
ZNF700	4.78	5.22	4.48
VGLL2	4.78	5.32	4.38
NBEA	4.78	5.35	4.50
LOC283440	4.78	5.09	4.44
OTTHUMG00000163996	4.78	5.14	4.56
CHRAC1	4.78	5.00	4.53
IQCB1	4.78	5.09	4.13
MIR3179-2	4.78	5.15	4.01
CCDC135	4.78	5.21	4.35
TMEM218	4.78	5.09	4.39
LOC100507054	4.78	5.19	4.34
PIK3C2B	4.78	5.26	4.26
CPZ	4.78	5.21	4.23

HTATIP2	4.78	5.03	4.45
LOC100133207	4.78	5.34	4.44
ACP6	4.78	5.03	4.40
FGF17	4.78	5.26	4.12
HS6ST1	4.78	5.17	4.37
LRRC37B	4.78	5.53	3.92
PRAMEF8	4.78	5.30	4.34
MIR105-2	4.78	5.13	4.34
KCNK16	4.78	5.11	4.46
ADD2	4.78	5.16	4.37
SOX7	4.77	5.18	4.38
ACSS3	4.77	5.41	4.07
LYSMD1	4.77	5.07	4.38
PLEKHM1P	4.77	5.00	4.24
INCA1	4.77	5.20	4.43
GNG5	4.77	5.36	4.28
MIR28	4.77	5.17	3.91
OTTHUMG00000161598	4.77	5.43	3.88
OTTHUMG00000032826	4.77	5.03	4.29
ZNF772	4.77	5.22	4.50
CTXN1	4.77	5.11	4.31
CCDC154	4.77	5.13	4.42
SPDEF	4.77	5.25	4.39
C19orf25	4.77	5.04	4.52
INSM1	4.77	5.28	4.00
GPX6	4.77	5.48	4.20
NRARP	4.77	5.75	4.21
LOC100128002	4.77	5.33	4.48
GNAO1	4.77	5.16	4.43
SFN	4.77	5.10	4.37
EIF2S3	4.77	5.18	3.91
EVI2A	4.77	5.65	3.89
C14orf182	4.77	5.12	4.47
ZSCAN30	4.77	5.11	4.53
AKR7L	4.77	5.23	3.64
GGT8P	4.77	5.53	4.14
LOC100507654	4.77	5.17	4.26
ZNF253	4.77	5.51	4.05
TMEM100	4.77	5.07	4.02
KRTAP29-1	4.77	5.12	4.32
SBNO2	4.77	5.31	4.55
CFC1	4.77	5.15	4.39
UBE2E1	4.77	5.07	4.26
B3GALT2	4.77	5.23	4.35
NHLH1	4.77	5.31	4.28
SMAD7	4.77	5.27	4.38
L3MBTL1	4.77	5.20	4.54
NR5A1	4.77	5.09	4.43
SNORD41	4.77	5.72	4.04

PSMG3-AS1	4.77	5.46	4.47
SCARNA21	4.77	5.11	4.33
OTTHUMG00000171325	4.77	6.12	3.76
LACTBL1	4.77	5.00	4.51
MIR1286	4.77	5.15	4.33
PRMT8	4.77	5.05	4.41
MTERFD3	4.77	5.05	4.43
TMEM86B	4.77	4.98	4.55
CSRNP1	4.77	5.12	4.50
FAM129C	4.77	5.28	4.37
CARNS1	4.77	5.23	4.44
OGFR	4.77	5.06	3.95
OXTR	4.77	5.31	4.51
MGC45922	4.77	5.12	4.53
TRPM2	4.77	5.03	4.38
BMP5	4.77	5.68	3.84
ZNF138	4.77	5.30	4.30
FAM41C	4.77	5.29	4.45
CSRP2	4.77	5.50	4.14
OVGP1	4.77	4.95	4.53
TINAGL1	4.77	5.42	4.03
EFCAB2	4.77	5.14	4.59
OTTHUMG00000183930	4.77	5.21	4.40
FXN	4.77	5.15	4.30
OTTHUMG00000170749	4.77	5.03	4.50
SEZ6L2	4.77	5.13	4.44
LOC100129724	4.77	5.06	4.32
ZNF341	4.77	5.00	4.41
LOC100506405	4.77	5.14	4.28
SPAG1	4.77	5.07	4.38
LOC440149	4.77	5.42	4.32
INHBA-AS1	4.77	5.23	4.40
SARS2	4.77	5.07	4.48
POM121L12	4.77	5.16	4.50
MIR124-1	4.77	5.07	4.44
C19orf52	4.77	5.16	4.48
HEIH	4.77	5.10	4.35
MORN4	4.77	5.28	4.38
OXCT1-AS1	4.77	4.98	4.55
MAGEA1	4.77	5.14	3.92
RAB37	4.77	5.07	4.38
C6orf25	4.77	5.23	4.49
STAC2	4.77	5.10	4.50
PEMT	4.77	5.28	4.37
MFI2-AS1	4.77	5.09	4.31
LYRM2	4.77	4.95	4.57
AATK	4.77	4.92	4.54
TAF5	4.77	5.02	4.43
LOC100996658	4.77	5.31	4.26

TRBV7-3	4.77	5.40	4.33
UNC5A	4.77	5.14	4.43
NDUFA4	4.77	5.17	4.05
GUCY1A3	4.77	5.95	3.78
SMARCAL1	4.77	5.26	4.30
FAM35BP	4.77	5.21	4.19
NUDT12	4.77	5.38	4.06
EPHX3	4.77	5.09	4.42
ZNF470	4.77	5.28	4.45
ANKRD20A1	4.77	5.58	3.93
ANKRD20A4	4.77	5.58	3.93
FAM124B	4.77	5.35	4.10
PHOSPHO2-KLHL23	4.77	5.07	4.37
LOC100128398	4.77	5.14	4.39
FAM160A1	4.77	5.21	4.28
MRPS27	4.77	5.10	4.26
IL31RA	4.77	5.53	4.33
OTTHUMG00000173288	4.77	5.14	4.31
EGOT	4.77	5.45	4.04
OTTHUMG00000170730	4.77	5.36	3.98
BTBD16	4.77	5.27	4.49
DPYSL4	4.77	5.23	4.41
MLIP	4.77	5.27	4.41
RNA5SP473	4.77	5.42	4.21
HSPA12A	4.77	5.41	4.15
TMEM210	4.77	5.26	4.21
BLZF1	4.77	5.21	4.03
SLC6A19	4.77	5.26	4.40
HMGA2	4.77	5.24	4.43
TTC24	4.76	5.51	4.36
C18orf12	4.76	5.09	4.27
MAGEA8	4.76	5.40	4.38
SEC14L2	4.76	5.02	4.52
VAT1L	4.76	5.01	4.12
FGF4	4.76	4.90	4.59
WIZ	4.76	5.29	4.36
LOC100506901	4.76	5.61	4.09
FAM200A	4.76	5.17	4.51
MIR194-1	4.76	5.41	4.09
SS18L2	4.76	5.44	4.13
AMPH	4.76	6.05	3.95
RNU1-19P	4.76	5.71	4.26
RHNO1	4.76	5.21	4.28
LOC100127967	4.76	5.30	4.05
ITPKB-AS1	4.76	5.28	4.33
ZNF41	4.76	5.07	4.39
RPS6KA2-IT1	4.76	5.15	4.49
DNASE1L3	4.76	5.61	3.82
MIR4422	4.76	5.29	4.39

AMER1	4.76	5.18	4.18
CHRNB2	4.76	5.10	4.39
FAM178B	4.76	5.10	4.42
OR2T4	4.76	5.90	4.05
CEBPA	4.76	5.10	4.33
OTTHUMG00000159392	4.76	5.29	4.34
PKD1L1	4.76	5.04	4.16
C4A-AS1	4.76	5.15	4.22
GLMN	4.76	5.19	4.25
KRTAP23-1	4.76	5.05	4.45
NRN1L	4.76	5.03	4.49
ABTB2	4.76	4.94	4.56
CINP	4.76	5.07	3.91
N4BP2L1	4.76	5.07	4.43
SLC35F2	4.76	5.04	4.55
LOC100507417	4.76	5.45	4.30
HDAC2	4.76	5.19	4.20
LOC100128653	4.76	5.39	4.11
STOML1	4.76	5.03	4.44
LOC441239	4.76	5.30	4.40
OTTHUMG00000164284	4.76	5.64	4.12
SLC18A2	4.76	5.24	4.36
SUCLG1	4.76	5.27	4.32
BSX	4.76	5.27	4.23
ENC1	4.76	4.99	4.59
POLR3F	4.76	5.24	4.41
ARHGAP22-IT1	4.76	5.03	4.22
GNAT2	4.76	5.06	4.36
WDR13	4.76	5.15	4.32
IGLL3P	4.76	5.35	3.76
LOC100505478	4.76	5.32	3.98
UNCX	4.76	5.02	4.59
ZC3H12C	4.76	5.23	4.23
FAM183CP	4.76	5.10	4.36
CISH	4.76	5.02	4.41
FAM153C	4.76	5.60	4.24
RNA5SP64	4.76	5.49	3.57
RNY3P7	4.76	5.52	3.93
NOX4	4.76	5.68	3.91
RPGR	4.76	5.05	4.40
MLXIPL	4.76	5.23	4.40
PIPOX	4.76	5.78	4.21
GNA15	4.76	5.25	4.39
PLXNA4	4.76	5.19	4.49
SNAPC4	4.76	5.11	4.54
IRF1	4.76	5.40	4.27
RRP8	4.76	5.04	4.47
TMC7	4.76	5.31	4.28
KLHL35	4.76	5.15	4.14

MRGPRG-AS1	4.76	5.12	4.47
TRMT2A	4.76	5.04	4.21
RECQL5	4.76	5.00	4.33
C7orf10	4.76	5.18	4.04
TKTL1	4.76	5.11	4.35
LANCL2	4.76	5.06	4.47
NIPAL3	4.76	5.08	4.47
SNRPEP2	4.76	4.99	4.54
WAS	4.76	5.07	4.45
SEMA4F	4.76	5.24	4.40
CCDC33	4.76	5.09	4.11
INPP5J	4.76	5.30	4.28
MAPT	4.76	5.08	4.23
CES1P2	4.76	5.03	4.33
OR51I1	4.76	5.01	4.45
SLC28A2	4.76	5.06	4.47
OTTHUMG00000034820	4.76	5.28	4.41
FAM206A	4.76	5.07	4.50
CYB5R2	4.76	5.07	4.55
TMEM107	4.76	5.09	4.45
C5orf17	4.76	5.23	4.57
PPIL1	4.76	5.21	4.31
HSD17B7	4.76	5.08	4.14
DEFA1	4.76	5.25	4.15
PDK2	4.76	5.25	4.20
IPO11	4.76	5.12	4.47
CCDC126	4.76	5.03	4.40
HMHA1	4.76	5.19	4.23
CENPV	4.76	5.21	4.29
MON1B	4.76	5.07	4.50
SYNE4	4.76	5.13	4.29
ARHGAP36	4.76	5.19	4.34
FOXP2	4.76	5.17	4.26
MYLK4	4.76	5.11	4.46
PROK1	4.76	5.11	4.33
OTTHUMG00000151627	4.76	5.43	4.24
CABP4	4.75	5.02	4.26
OTTHUMG00000032097	4.75	5.49	4.43
NYNRIN	4.75	5.01	4.52
NKAIN3	4.75	5.03	4.49
TM4SF18	4.75	5.85	3.87
NXPH4	4.75	5.29	4.22
GIMAP2	4.75	5.39	3.89
MIR4799	4.75	5.17	4.33
MGC16142	4.75	5.16	4.30
TRAF3IP3	4.75	5.11	4.45
OTTHUMG00000000564	4.75	5.18	4.09
OTTHUMG00000150605	4.75	5.82	4.16
NCAPG2	4.75	5.18	4.46

OTTHUMG00000164576	4.75	5.12	4.47
OR6V1	4.75	5.43	4.07
PHKA1	4.75	5.19	4.23
RHPN1-AS1	4.75	5.30	4.27
C16orf89	4.75	5.00	4.39
EFCAB4A	4.75	4.99	4.17
BCL2L1	4.75	5.22	4.25
DIRAS2	4.75	5.06	4.50
MCAM	4.75	5.35	4.17
ATP7B	4.75	5.18	4.51
C7orf34	4.75	5.03	4.43
POFUT1	4.75	5.23	4.29
SAPCD2	4.75	4.99	4.59
DIMT1	4.75	4.88	4.42
BIN3-IT1	4.75	4.97	4.57
MIR4733	4.75	5.17	3.76
ORC6	4.75	5.18	4.28
ESPNP	4.75	5.43	4.25
GABRD	4.75	5.07	4.30
SCARNA6	4.75	5.29	4.24
TMEM183B	4.75	5.15	4.11
MIS18A	4.75	5.12	4.26
BCRP3	4.75	5.58	3.86
C17orf96	4.75	5.00	4.46
OTTHUMG00000163630	4.75	5.42	4.44
OTTHUMG00000154167	4.75	4.97	4.46
ART1	4.75	5.30	4.32
OTTHUMG00000151420	4.75	5.22	4.12
DNAL1	4.75	5.11	4.41
LACTB2	4.75	5.19	3.97
MS4A4A	4.75	6.05	4.08
KLRAP1	4.75	5.08	4.39
PTPN5	4.75	5.20	4.19
LINC00565	4.75	4.94	4.37
PRDM10	4.75	5.08	4.57
OTUD3	4.75	5.10	4.47
LOC100996490	4.75	4.98	4.55
GTPBP8	4.75	5.26	4.32
DOK1	4.75	5.03	4.42
WEE2	4.75	5.33	4.44
KRT86	4.75	5.32	4.09
AQP2	4.75	5.04	4.42
PLB1	4.75	5.09	4.21
HOMER3	4.75	5.41	4.27
C19orf57	4.75	5.10	4.50
LOC100130193	4.75	5.55	4.12
BSPRY	4.75	5.05	4.54
TMEM88	4.75	5.27	4.33
KLC2	4.75	5.17	4.28

FLJ30679	4.75	5.40	4.38
OTTHUMG00000009373	4.75	5.38	4.21
PBXIP1	4.75	5.38	4.24
ZNF81	4.75	5.12	4.34
SEMA7A	4.75	5.29	4.33
CDC45	4.75	5.21	4.45
COA6	4.75	5.16	4.10
OTTHUMG00000014331	4.75	5.15	4.33
TRMT61A	4.75	5.07	4.48
RBM48	4.75	5.10	4.29
CLCN7	4.75	5.35	4.28
ULK1	4.75	4.95	4.36
PRSS35	4.74	5.71	3.88
MED26	4.74	4.94	4.40
LOC100129083	4.74	5.48	3.92
DUS1L	4.74	5.17	4.22
SNORD116-20	4.74	5.48	3.93
CDC42EP4	4.74	5.17	4.19
USP35	4.74	4.97	4.46
BOLA3	4.74	5.29	4.18
ADAT1	4.74	5.20	4.50
PA2G4	4.74	4.96	4.51
OR2K2	4.74	5.27	4.22
AFAP1L1	4.74	5.55	4.35
FOXD4L1	4.74	5.44	4.17
HERC6	4.74	5.20	4.25
LOC730081	4.74	5.34	4.33
IGFL4	4.74	5.26	4.42
DCC	4.74	6.18	3.88
NPM1	4.74	5.11	4.33
ATG16L1	4.74	5.23	4.49
FLJ46026	4.74	5.27	4.47
USP32P1	4.74	5.31	3.91
OTTHUMG00000168374	4.74	5.51	4.26
SLC19A1	4.74	4.99	4.54
C20orf202	4.74	4.97	4.36
TOB2	4.74	5.14	4.12
TBX2	4.74	5.08	4.23
XYLT2	4.74	5.18	4.39
MIR3160-1	4.74	5.03	4.31
ZBED6CL	4.74	5.08	4.34
SLC22A8	4.74	5.34	4.16
BTN3A3	4.74	5.40	4.19
ERCC4	4.74	5.02	4.49
CCDC24	4.74	5.15	4.44
GXYLT1	4.74	5.00	4.37
TRIM67	4.74	4.96	4.40
LOC100129518	4.74	5.28	3.76
C1orf61	4.74	5.07	4.53

GPR176	4.74	5.37	4.07
MAP3K9	4.74	5.32	4.42
IQCA1P1	4.74	5.04	4.54
SHCBP1	4.74	5.51	4.35
GALNT14	4.74	5.50	4.39
BBS5	4.74	5.15	4.26
C17orf53	4.74	5.13	4.48
MIR3659	4.74	5.46	4.08
SERPINA10	4.74	5.10	4.24
ZMAT1	4.74	5.41	4.31
FAM107A	4.74	5.05	4.38
KIAA0125	4.74	5.50	3.98
RNA5SP521	4.74	5.29	4.33
SOHLH1	4.74	4.92	4.49
ZNRF4	4.74	5.15	4.19
PDE10A	4.74	5.24	4.14
OR1F2P	4.74	5.32	4.29
CCL18	4.74	5.47	4.27
LOC100505550	4.74	5.17	4.50
LOC100128908	4.74	5.28	4.10
ENKD1	4.74	5.04	4.48
SRGAP3-AS4	4.74	5.04	4.28
LINC00654	4.74	5.12	4.41
SARDH	4.74	5.01	4.38
NACC2	4.74	5.05	4.42
RNA5SP330	4.74	5.17	4.40
LOC100129534	4.74	5.19	4.44
LOC100130071	4.74	5.26	4.21
LRRC8D	4.74	5.04	4.30
SLC5A10	4.74	5.03	4.54
CYP46A1	4.74	5.02	4.37
NME3	4.74	5.00	4.42
STRBP	4.74	5.03	4.42
CNNM2	4.74	5.06	4.43
CEP57L1	4.74	5.19	3.84
LINC00950	4.74	5.18	4.38
OTTHUMG00000168057	4.74	5.09	4.34
MLPH	4.74	5.19	4.29
FAM47E	4.74	4.94	4.58
RELT	4.74	5.19	4.37
LRRCC1	4.74	5.29	3.94
RNF43	4.74	5.13	4.47
RSPO1	4.74	5.28	4.48
MIP	4.74	5.00	4.43
ZNF594	4.74	5.30	4.40
HMGN2	4.73	5.08	3.57
VPS18	4.73	5.18	4.09
RNA5SP110	4.73	6.48	4.17
DCAF17	4.73	5.04	4.18

GET4	4.73	5.03	4.33
LOC100499221	4.73	5.26	4.33
ZNF300P1	4.73	5.10	4.33
SPATA19	4.73	5.12	4.26
ALG5	4.73	5.30	4.01
GLI4	4.73	5.36	4.41
CCR9	4.73	5.08	4.31
DNAI2	4.73	4.99	4.34
CCDC13	4.73	5.10	4.32
UNC119	4.73	5.06	4.40
LOC401480	4.73	5.10	4.34
CXorf49	4.73	5.02	4.32
HIST1H2BC	4.73	5.81	3.53
ALDH3A1	4.73	5.05	4.40
OTTHUMG00000182495	4.73	5.53	4.03
TMEM92	4.73	5.41	4.40
NRTN	4.73	5.29	4.11
GALNT12	4.73	5.26	4.38
LSM14B	4.73	4.88	4.55
RAPGEF5	4.73	5.44	4.06
LOC151657	4.73	5.17	4.14
OTTHUMG00000171570	4.73	5.46	4.33
CCDC37	4.73	5.26	4.38
WFIKKN2	4.73	4.98	4.26
ZBTB11-AS1	4.73	4.98	4.38
OTTHUMG00000171639	4.73	5.05	4.26
RNU7-27P	4.73	5.43	3.95
MYT1	4.73	5.24	3.93
LOC100133077	4.73	5.11	4.36
RAB40B	4.73	5.09	4.05
HYAL1	4.73	5.06	4.53
C6orf57	4.73	5.30	3.84
OTTHUMG00000166127	4.73	5.11	4.19
TNNI1	4.73	5.23	4.35
OTTHUMG00000171299	4.73	5.03	4.31
SPAG8	4.73	4.98	4.59
RAB3A	4.73	5.24	4.32
HAP1	4.73	5.19	4.42
KIR3DX1	4.73	5.08	4.49
C1orf167	4.73	5.29	4.49
C8orf82	4.73	4.99	4.28
NR3C2	4.73	5.07	4.27
DCAF13P3	4.73	5.30	3.89
MIR1265	4.73	5.78	3.98
PPP1R13L	4.73	5.30	4.18
CLIC5	4.73	5.69	3.38
DSERG1	4.73	5.40	4.01
ZRANB2-AS2	4.73	5.03	4.31
RNA5SP362	4.73	5.07	4.42

OR1I1	4.73	5.05	4.36
MIR4697HG	4.73	5.17	4.33
LOC101060449	4.73	5.32	3.94
GPR62	4.73	5.04	4.34
MIR1294	4.73	5.52	4.37
MYH14	4.73	5.09	4.38
OTTHUMG00000171943	4.73	5.16	3.85
CXorf40A	4.73	5.35	4.10
GHR	4.73	5.37	3.43
PDLIM2	4.73	5.13	4.50
SCYL2	4.73	5.12	4.20
NTRK1	4.73	5.34	4.14
EXD2	4.73	5.15	3.92
LOC100509315	4.73	5.11	4.44
BPIFA1	4.73	5.54	4.44
FLJ45079	4.73	5.27	4.20
ANKRD29	4.73	5.39	4.25
TPCN2	4.73	5.00	4.41
LOC100507250	4.73	5.26	4.27
PRKG1	4.73	5.37	3.64
SNPH	4.73	5.08	4.36
LRFN5	4.73	5.52	4.18
PP13	4.73	5.20	4.21
CCDC162P	4.73	5.21	3.82
KIF21B	4.73	5.22	4.53
SDR9C7	4.73	5.30	4.35
LOC100506051	4.73	5.02	4.14
SEMA6A	4.73	5.17	4.28
DMRTB1	4.73	5.31	4.23
AIF1L	4.73	5.21	4.37
CECR9	4.73	5.22	4.08
OTTHUMG00000172420	4.73	5.38	4.12
TRIM45	4.73	5.03	4.30
CARS2	4.73	4.97	4.42
LGALS7B	4.73	4.94	4.57
TTLL1	4.73	5.04	4.18
MMRN2	4.72	5.02	4.34
HOGA1	4.72	5.04	4.50
LOC100505782	4.72	5.01	4.29
MAB21L2	4.72	5.04	4.25
KATNAL2	4.72	4.96	4.55
ZNF443	4.72	5.03	4.20
LOC100129999	4.72	5.13	4.36
SUV39H1	4.72	5.09	4.47
POR	4.72	4.92	4.35
TMSB4XP4	4.72	5.29	3.89
TNNT2	4.72	5.21	3.95
APOL2	4.72	5.19	4.24
OTTHUMG00000152374	4.72	4.99	4.38

GPR110	4.72	5.27	4.11
SP6	4.72	5.10	4.37
DUS3L	4.72	4.98	4.39
IGHG3	4.72	5.25	4.01
OTTHUMG00000166506	4.72	5.21	4.29
TRIM21	4.72	5.00	4.44
RPL39L	4.72	5.79	3.90
LCN12	4.72	5.06	4.25
C7orf61	4.72	5.16	4.43
LOC100131510	4.72	5.37	4.24
OTTHUMG00000017650	4.72	5.30	4.35
DNAJC30	4.72	5.45	4.45
CYP26B1	4.72	5.34	4.29
TSPAN9	4.72	5.03	4.30
IL1F10	4.72	5.20	4.16
LOC100133985	4.72	5.44	4.09
CD72	4.72	4.99	4.29
ABCC6	4.72	5.55	4.33
SLC6A17	4.72	5.31	4.28
GUCY2EP	4.72	4.93	4.42
MPP7	4.72	5.49	4.11
EFCAB11	4.72	4.96	4.36
RBFOX3	4.72	5.10	4.45
NKX6-2	4.72	5.01	4.44
TRAM1L1	4.72	5.23	4.33
PINLYP	4.72	5.13	4.40
MAGOHB	4.72	5.40	4.13
SPATA31A3	4.72	5.30	4.21
OTTHUMG00000161871	4.72	5.32	4.22
SCN4A	4.72	5.35	4.39
LINC00683	4.72	5.12	4.29
OTTHUMG00000018145	4.72	5.10	4.38
KCNG1	4.72	5.25	4.49
OSTM1-AS1	4.72	5.27	3.92
SNCG	4.72	5.03	4.32
LRR1	4.72	5.17	3.95
DKFZP434A062	4.72	5.00	4.41
GRP	4.72	5.11	3.94
FAM99A	4.72	5.09	4.20
SURF6	4.72	4.96	4.45
SCAI	4.72	5.09	4.46
ZNF280B	4.72	5.46	4.05
LINC00954	4.72	5.07	4.27
ADAM11	4.72	5.00	4.15
FAM198A	4.72	5.26	4.41
LRRC24	4.72	5.19	4.28
DKKL1	4.72	5.22	4.32
CPT2	4.72	5.24	3.89
FAM120C	4.72	4.96	4.41

PAXBP1-AS1	4.72	5.08	4.26
HAUS2	4.72	5.41	4.19
PAPPA	4.72	5.60	4.35
MBOAT7	4.72	5.08	4.23
LOC100505949	4.72	5.33	4.41
BAAT	4.72	5.12	3.87
CDK10	4.72	5.08	4.25
GRM6	4.72	5.15	4.10
KCNH3	4.72	5.05	4.44
AMICA1	4.72	5.23	4.27
SYK	4.72	5.54	4.27
MED7	4.72	4.87	4.46
WFDC2	4.72	5.24	4.32
SLC25A19	4.72	5.40	4.21
METTL2A	4.72	5.48	3.55
GAS5-AS1	4.72	5.13	4.34
POU4F1	4.71	5.06	4.31
ZKSCAN4	4.71	5.07	4.02
RARRES1	4.71	5.02	4.25
FGF11	4.71	5.51	4.09
JRKL	4.71	5.07	4.55
TRAV22	4.71	5.30	4.37
ZNF415	4.71	5.17	4.39
ALDH3B2	4.71	5.30	4.04
P2RX6	4.71	5.20	4.25
MIR1245B	4.71	6.46	2.64
AP3B2	4.71	5.13	4.35
BOK-AS1	4.71	5.17	4.30
ST8SIA1	4.71	5.06	4.36
KCNQ2	4.71	5.08	4.23
STMN2	4.71	5.19	4.35
ZNF425	4.71	4.94	4.38
OTTHUMG00000045388	4.71	5.23	4.03
GRTP1	4.71	4.98	4.39
LOC100505622	4.71	5.16	4.23
ZBTB9	4.71	5.24	4.41
OTTHUMG00000150113	4.71	5.30	4.08
COQ2	4.71	5.19	4.12
LOC255130	4.71	5.07	4.47
ZFP2	4.71	4.98	4.31
LOC100996286	4.71	5.32	3.75
DAND5	4.71	4.95	4.16
ZNF133	4.71	4.90	4.48
LOC79015	4.71	5.09	4.51
TRIM14	4.71	5.07	4.40
OTTHUMG00000014930	4.71	5.21	4.09
NPM3	4.71	4.83	4.51
RIMBP3C	4.71	5.04	4.27
ABCC8	4.71	5.00	4.28

ZNF283	4.71	5.40	3.80
LANCL1	4.71	5.05	4.26
KCNE1	4.71	5.06	4.09
TTC7B	4.71	5.02	4.26
ATP6V1E2	4.71	5.19	4.30
GRTP1-AS1	4.71	4.91	4.50
MTFP1	4.71	5.07	4.37
C14orf23	4.71	5.29	4.12
CNTNAP1	4.71	5.17	4.24
OTTHUMG00000017359	4.71	5.22	4.27
PAG1	4.71	5.17	4.20
MAN2A2	4.71	4.91	4.39
FAM74A4	4.71	5.17	4.18
AKR1B1P6	4.71	5.36	4.22
RPL28	4.71	4.95	4.30
PAX4	4.71	5.40	4.49
TMEM200C	4.71	5.20	4.26
IL1RL2	4.71	5.18	4.21
RIMS3	4.71	5.00	4.26
C19orf69	4.71	5.19	4.07
OTTHUMG00000171473	4.71	6.14	3.32
DBF4B	4.71	5.09	4.36
MIR758	4.71	5.41	4.24
H6PD	4.71	5.22	4.17
LRRC14B	4.71	5.06	4.18
CAV3	4.71	5.16	4.32
WNT7A	4.71	5.28	4.15
CA13	4.71	4.99	4.28
LOC100132215	4.71	5.25	4.34
TTLL11-IT1	4.71	5.08	4.29
LOC100133957	4.71	4.97	4.20
LOC100996672	4.71	5.22	4.14
RNF126P1	4.71	5.00	4.24
TPPP2	4.71	5.03	4.36
BET1	4.71	5.20	4.35
APC2	4.71	5.24	4.42
C9orf106	4.71	5.24	4.26
CMTM1	4.71	5.24	4.37
INGX	4.71	5.16	4.42
FSD2	4.71	4.94	4.19
TEX9	4.71	5.00	4.03
ATP8A1	4.71	5.16	4.32
MPPED2	4.70	5.74	3.94
TRIM59	4.70	5.31	4.16
ZNF668	4.70	5.13	3.87
SC5D	4.70	5.22	4.06
FBN3	4.70	5.11	4.26
EVA1C	4.70	5.03	4.41
USP43	4.70	5.05	4.41

PLAC8	4.70	5.27	4.31
NCS1	4.70	5.18	3.89
CCDC60	4.70	5.10	4.22
LINC00243	4.70	5.10	4.43
RETN	4.70	5.13	4.15
RTL1	4.70	5.01	4.28
FCGBP	4.70	5.33	4.00
CCDC58	4.70	5.13	3.95
TMC2	4.70	4.91	4.43
INTS9	4.70	5.14	4.28
ZNF774	4.70	4.98	4.42
OTTHUMG00000164174	4.70	5.47	3.76
DZANK1	4.70	5.16	4.40
TSPAN12	4.70	5.16	4.42
INPP5A	4.70	4.84	4.35
WAC-AS1	4.70	4.90	4.33
OTTHUMG00000169388	4.70	5.06	4.20
SNHG11	4.70	5.08	4.38
ST20	4.70	4.92	4.27
LINC00885	4.70	5.10	3.89
MLNR	4.70	5.02	4.22
FAM175A	4.70	5.10	4.24
TRDMT1	4.70	5.29	4.31
OTTHUMG00000165228	4.70	5.44	4.29
DPY19L1	4.70	5.09	4.46
IGHV3-33	4.70	5.32	4.11
PTGER1	4.70	5.30	4.39
ARC	4.70	4.98	4.34
OTTHUMG00000169068	4.70	5.13	4.39
CMC1	4.70	5.03	4.33
LRRC30	4.70	5.00	4.40
OTTHUMG00000037161	4.70	5.09	4.22
SLC22A18AS	4.70	5.25	4.35
HHIP-AS1	4.70	5.15	4.16
COPG2	4.70	5.18	4.48
HAVCR1P1	4.70	5.38	3.92
MAL	4.70	5.01	4.23
TEX40	4.70	5.08	4.49
SIRPB1	4.70	5.14	4.11
CCDC171	4.70	5.02	4.28
OTTHUMG00000176406	4.70	5.26	4.21
ZNF790-AS1	4.70	4.91	4.28
BCAN	4.70	5.02	4.44
BCAM	4.70	5.61	4.00
ZBTB39	4.70	5.08	4.08
SHISA8	4.70	5.03	4.36
NKD2	4.70	5.03	4.43
TRIM15	4.70	4.96	4.36
TMEM123	4.70	4.94	4.41

WIPF3	4.70	5.27	4.32
ACSL5	4.70	5.19	3.86
OTTHUMG00000171900	4.70	5.22	4.07
LINC00602	4.70	5.12	4.33
STK40	4.70	5.05	4.44
COL8A1	4.70	6.00	3.45
C10orf71	4.70	5.25	4.12
LOC100294362	4.70	5.14	4.38
C1orf112	4.70	4.93	4.47
LOC100996669	4.70	5.41	4.28
DEFB116	4.70	5.64	3.90
THEM6	4.70	4.88	4.36
JPH2	4.70	5.04	4.33
TRMT61B	4.70	5.44	4.12
CPN1	4.70	5.15	4.09
HOXB-AS3	4.70	4.98	4.42
DENND1B	4.70	5.09	4.28
CRYBA2	4.70	5.15	4.32
MAP2K3	4.70	5.04	4.51
LOC339524	4.70	5.18	4.11
C1orf116	4.70	5.19	4.19
TSPY1	4.70	5.25	4.13
TMEM161B-AS1	4.70	5.07	4.34
FGF1	4.70	5.20	4.25
SPSB2	4.70	5.01	4.34
C9orf84	4.70	5.02	4.41
GDF2	4.70	5.29	4.12
PXN-AS1	4.70	4.92	4.39
OTTHUMG00000162467	4.70	4.98	4.31
ANO10	4.70	5.06	4.23
EIF1B	4.70	5.01	4.33
SBK1	4.70	5.15	4.22
AMOT	4.70	4.99	4.39
UNQ6494	4.70	5.01	4.19
APOC1	4.70	7.27	3.24
ICOSLG	4.70	5.07	4.29
OTTHUMG00000162476	4.70	5.17	3.72
LINC00035	4.70	5.14	4.22
CD200	4.70	5.22	3.71
C6orf47	4.70	5.08	4.21
ZNF583	4.70	4.94	4.27
KLHL6	4.70	5.08	4.30
CHCHD6	4.70	4.93	4.37
NEDD4L	4.70	5.25	4.33
DOLK	4.70	5.46	4.24
OTTHUMG00000166972	4.70	5.08	4.22
LOC100653005	4.69	5.20	4.31
LAMA3	4.69	5.19	4.37
ACBD4	4.69	4.97	4.43

OTTHUMG00000161934	4.69	5.03	4.41
PDE2A	4.69	5.17	4.23
DRAM1	4.69	5.07	4.17
TG	4.69	5.07	4.45
SNORD114-3	4.69	7.07	2.74
FAM227A	4.69	5.04	4.14
MYOT	4.69	5.13	4.15
LRRC34	4.69	5.18	4.15
OTTHUMG00000165450	4.69	5.35	3.90
CCDC136	4.69	4.97	4.27
MIR1180	4.69	5.22	4.05
PGCP1	4.69	5.15	4.22
ANKRD35	4.69	4.96	4.53
OR7E14P	4.69	5.10	4.08
ITCH-IT1	4.69	4.88	4.30
RHPN2	4.69	5.08	4.45
KRTAP5-2	4.69	5.26	3.97
KRT79	4.69	5.46	4.14
FAM83H	4.69	5.17	4.28
ODF3L1	4.69	4.89	4.47
OTTHUMG00000162005	4.69	5.77	3.95
NDUFA5	4.69	5.05	4.21
TCTEX1D4	4.69	5.53	3.88
OTTHUMG00000008038	4.69	5.20	4.23
BEND6	4.69	5.98	4.05
CANT1	4.69	5.02	4.01
CD79B	4.69	5.24	4.36
TXNRD2	4.69	4.88	4.41
GRID1	4.69	4.96	4.36
ZNF688	4.69	5.06	4.39
ALDH1L1	4.69	5.21	4.42
OR5B17	4.69	5.57	4.04
HMX3	4.69	5.24	4.09
ANKLE1	4.69	5.14	4.27
ANKS6	4.69	4.99	4.38
VWA3A	4.69	5.10	4.24
USP20	4.69	5.07	4.30
MIR654	4.69	5.35	3.78
NPNT	4.69	5.12	4.07
PSMA6	4.69	5.15	4.35
TULP1	4.69	5.11	4.45
CST8	4.69	4.90	4.14
LOC400943	4.69	4.98	4.37
KIAA1324	4.69	5.30	4.11
FLJ11235	4.69	5.35	4.04
OTTHUMG00000169280	4.69	5.29	4.04
ASCL1	4.69	5.14	4.21
RANBP17	4.69	4.87	4.19
SNORD59B	4.69	5.56	3.65

CD226	4.69	5.33	4.13
CECR1	4.69	5.63	4.27
IRF6	4.69	5.13	4.39
SYP-AS1	4.69	5.15	4.38
CHRD	4.69	5.19	4.48
GPR182	4.69	5.17	4.41
RNASEL	4.69	5.35	3.84
ZNF503-AS2	4.69	5.30	4.34
OTTHUMG00000168297	4.69	5.05	4.11
LOC643441	4.69	5.20	4.21
DUSP13	4.69	5.02	4.36
MAP3K14	4.69	5.17	4.38
PITPNM1	4.69	4.97	4.32
THRSP	4.69	5.78	3.77
OR7G2	4.69	5.03	4.45
AK8	4.69	4.95	4.42
FAM83A	4.69	5.18	4.39
AGAP2	4.69	5.04	4.24
DHRS4	4.69	5.26	4.23
MIR4258	4.69	6.23	3.99
GPR161	4.69	5.05	4.43
TREML2	4.69	5.31	4.27
NCAPH	4.69	5.14	4.40
TMEM72	4.69	5.34	4.36
LOC101060264	4.69	5.22	4.30
ADAM8	4.69	5.00	4.26
LYG2	4.69	4.89	4.08
LOC100288570	4.69	5.80	4.10
KIAA1524	4.69	5.29	4.19
PRR18	4.69	4.91	4.31
LINC00304	4.68	5.03	4.33
SSX4	4.68	5.35	4.13
IGSF21	4.68	5.00	4.30
KRT82	4.68	5.26	4.19
MKNK1-AS1	4.68	5.12	4.14
RNA5SP364	4.68	5.31	4.10
ORAOV1	4.68	5.45	4.09
PPP2R3C	4.68	5.17	4.08
ADAMTSL5	4.68	5.38	4.12
NDP-AS1	4.68	4.94	4.40
ZCCHC24	4.68	5.12	4.26
LOC644093	4.68	5.00	4.21
GAB4	4.68	5.25	4.38
FER1L6	4.68	5.10	4.42
TCHHL1	4.68	4.96	4.36
GRAMD2	4.68	4.98	4.20
NXF3	4.68	5.09	4.03
MYO7A	4.68	5.06	4.21
TRIM46	4.68	4.89	4.40

LOC284865	4.68	5.49	3.95
BZRAP1-AS1	4.68	4.89	4.48
MRPS12	4.68	5.16	4.35
OTTHUMG00000160654	4.68	5.01	4.28
TPRX1	4.68	5.23	4.06
ISL2	4.68	5.30	4.27
H2AFY2	4.68	4.98	4.39
TMPRSS5	4.68	5.23	4.45
PNKD	4.68	5.18	4.24
LOC285033	4.68	4.97	4.22
C1orf147	4.68	5.13	4.22
TTF1	4.68	5.09	4.36
FGFBP3	4.68	4.93	4.28
FAM117A	4.68	5.28	3.92
MAS1L	4.68	5.51	4.09
MYO5B	4.68	5.00	4.27
AP4S1	4.68	4.94	4.29
RFPL2	4.68	5.04	4.24
KRTAP26-1	4.68	5.41	3.90
CHST14	4.68	5.38	3.77
OTTHUMG00000169985	4.68	5.16	4.28
INTS6-AS1	4.68	4.89	4.36
KBTBD11	4.68	4.96	4.40
MFSD6L	4.68	5.23	4.32
AMER3	4.68	5.02	4.49
TSTD2	4.68	5.58	4.21
TAF1	4.68	5.22	4.21
OTTHUMG00000164286	4.68	4.93	4.50
OR3A1	4.68	5.16	4.10
WSCD1	4.68	5.02	4.37
VANGL1	4.68	5.40	4.33
MAGEA3	4.68	5.61	4.08
IGSF23	4.68	5.24	4.44
ANKRD34A	4.68	5.17	4.15
ACAD8	4.68	4.99	4.53
OTTHUMG00000165937	4.68	5.15	3.95
CEACAM1	4.68	5.28	4.21
SNORA77	4.68	5.01	4.37
SLC8A2	4.68	5.02	4.12
ITGB2	4.68	4.79	4.35
PLEKHA2	4.68	5.26	4.21
CORIN	4.68	4.98	3.88
PDZD7	4.68	5.04	4.16
SHBG	4.68	5.02	4.33
GLDC	4.68	4.90	4.42
PCDHB6	4.68	5.35	3.45
NAT8B	4.68	5.03	4.42
NUDT22	4.68	5.35	4.25
CLSPN	4.68	5.03	4.08

TSC22D4	4.68	5.29	4.17
OTTHUMG00000013230	4.68	5.10	3.89
AQP12A	4.68	5.15	3.65
DPPA2P3	4.68	5.03	4.17
LOC100129869	4.68	5.29	4.37
LOC729970	4.68	5.12	3.99
PLA2G4D	4.68	5.15	4.29
EPHB1	4.68	5.27	4.04
MRPL18	4.68	5.22	4.11
DSC3	4.68	5.56	3.87
LOC100130373	4.68	5.13	3.98
MAGEB6	4.68	5.07	4.02
LOC400622	4.68	5.00	4.33
FAM181A-AS1	4.67	5.08	3.93
MGC13053	4.67	5.16	4.20
SNORD83B	4.67	4.83	4.57
C12orf76	4.67	5.02	4.16
OTTHUMG00000013747	4.67	4.95	4.26
RNF215	4.67	4.89	4.44
LOC100289424	4.67	5.33	4.05
ZNF124	4.67	5.04	3.94
ZNF555	4.67	5.05	4.38
OTTHUMG00000156274	4.67	5.08	4.15
LILRB3	4.67	4.96	4.35
NSF	4.67	5.15	3.95
LINC00488	4.67	5.35	4.23
CYP2F1	4.67	5.05	4.33
VPS72	4.67	5.31	4.24
CACNA1C-AS1	4.67	5.04	4.08
DRAXIN	4.67	4.90	4.25
LOC100287329	4.67	5.24	4.35
MPND	4.67	5.08	4.32
ERAS	4.67	5.04	4.40
XXYLT1	4.67	4.99	4.15
LOC100127983	4.67	5.16	3.95
BAI2	4.67	5.27	4.17
YJEFN3	4.67	5.11	4.13
HELZ2	4.67	4.94	4.32
TRBV7-1	4.67	5.10	4.15
NMRK1	4.67	5.33	4.29
ALDH9A1	4.67	4.93	4.09
PBX3	4.67	5.12	4.16
SLC29A4	4.67	5.06	3.95
MYO15A	4.67	4.86	4.49
OTTHUMG00000168876	4.67	5.36	4.17
C2orf78	4.67	5.10	4.13
CMYA5	4.67	5.03	4.25
MNDA	4.67	5.88	3.93
GLB1L3	4.67	5.09	4.01

RIC3	4.67	5.00	4.23
RUNDC3A	4.67	4.99	4.13
CLIC3	4.67	5.09	4.28
C2orf91	4.67	5.23	4.30
MIR155	4.67	4.91	4.33
LINC00908	4.67	5.02	4.43
NSMF	4.67	5.03	4.28
CCDC87	4.67	4.86	4.50
LOC100507547	4.67	5.02	4.50
B3GALTL	4.67	5.32	3.57
BANK1	4.67	5.35	3.49
MOCS1	4.67	4.96	4.39
POLL	4.67	4.98	4.36
ZNF527	4.67	4.96	4.06
OTTHUMG00000003685	4.67	5.61	4.34
CTNND2	4.67	5.25	4.18
OTTHUMG00000032678	4.67	5.17	3.95
ZNF845	4.67	5.51	3.72
OBSCN	4.67	4.96	4.52
DPEP1	4.67	5.09	4.27
ITGA7	4.67	5.24	4.29
LOC100507530	4.67	5.01	4.31
IGLV3-12	4.67	5.00	4.03
LEPREL1-AS1	4.67	4.99	3.99
LOC100506834	4.67	4.99	4.37
SEC61A2	4.67	5.05	4.09
PHF16	4.67	5.33	3.73
OPN1LW	4.67	5.50	3.79
TMEM62	4.67	4.98	4.32
HEXIM2	4.67	5.04	4.39
DUSP28	4.67	4.94	4.37
CPXM2	4.67	5.25	4.09
KL	4.67	5.18	4.24
PTPRH	4.67	4.97	4.42
MIR1234	4.67	5.22	3.88
OR2T5	4.67	5.13	4.02
LOC100130480	4.67	5.12	4.37
TFR2	4.67	5.13	4.48
RPSAP52	4.67	5.37	3.66
LOC100507425	4.67	5.16	4.18
APLP1	4.67	5.22	4.18
ATP8B4	4.67	5.37	4.26
TSPY3	4.67	5.31	3.91
OSR2	4.67	5.18	3.92
SNRK	4.67	5.11	4.15
OTTHUMG00000171098	4.67	5.29	3.90
SLC36A2	4.67	4.95	4.18
OR7A10	4.67	5.25	3.87
OR2A42	4.67	5.20	3.98

CELF5	4.66	5.09	4.32
FBXO24	4.66	5.07	4.23
PDZD4	4.66	4.87	4.30
CERS4	4.66	5.16	4.28
LOC100132481	4.66	5.24	4.13
OTTHUMG00000140137	4.66	5.25	4.31
C18orf54	4.66	5.38	3.90
MCF2L	4.66	4.98	4.30
WDR85	4.66	4.94	4.49
NHEG1	4.66	4.97	4.49
MRAP	4.66	4.85	4.37
OTTHUMG00000159250	4.66	5.50	4.16
NEU4	4.66	4.98	4.34
XRCC4	4.66	5.37	3.58
TRBV25OR9-2	4.66	5.04	4.46
TEC	4.66	5.36	4.24
OTUB2	4.66	4.93	4.30
ADRA1B	4.66	4.93	4.34
SRRM5	4.66	4.91	4.13
STAG3	4.66	5.02	4.18
PAK3	4.66	4.84	4.43
ASTN2	4.66	4.88	4.52
VPS33A	4.66	4.99	4.34
GIPR	4.66	4.96	4.35
TRAJ45	4.66	5.56	3.48
TAS2R30	4.66	5.08	4.22
KCNH2	4.66	4.99	4.09
OTTHUMG00000018929	4.66	5.01	4.04
LGALS2	4.66	4.96	4.39
C8orf4	4.66	5.34	3.87
GDAP1L1	4.66	4.90	4.43
BMS1P4	4.66	5.50	3.90
ITLN1	4.66	5.58	3.99
KRTAP21-1	4.66	5.38	3.56
CETP	4.66	5.00	4.25
USP50	4.66	5.22	3.90
TMEM44-AS1	4.66	4.86	4.35
SKI	4.66	5.20	4.26
MGC39372	4.66	5.61	4.09
LCORL	4.66	5.04	4.40
SETD4	4.66	4.90	4.36
OTTHUMG00000017541	4.66	5.27	4.26
MIR449A	4.66	5.99	3.52
LCE1C	4.66	5.09	4.17
ANKRD49	4.66	5.02	4.00
KNCN	4.66	4.96	4.35
B3GAT1	4.66	5.18	4.27
NUP210L	4.66	5.28	4.24
OTTHUMG00000170272	4.66	5.10	4.01

TAF6L	4.66	5.01	4.27
CNKSR1	4.66	5.21	4.34
SLC39A10	4.66	5.22	4.02
SDCCAG3	4.66	5.30	4.17
VSTM2B	4.66	5.13	4.46
MAPK10	4.66	5.14	3.54
AKNAD1	4.66	5.50	4.16
NLGN3	4.66	4.89	4.39
SGSM2	4.66	5.23	4.01
NOVA2	4.66	5.15	4.08
TMEM182	4.66	5.19	4.17
FEZF1	4.66	5.46	4.29
SH3RF2	4.66	5.11	4.39
ADAMTS18	4.66	4.97	4.37
IL32	4.66	5.00	4.07
OTTHUMG00000020301	4.66	5.00	4.13
HOXB7	4.66	5.00	4.32
LOC100130880	4.66	5.19	4.18
ATP6AP1L	4.66	5.07	4.22
CC2D1A	4.66	5.08	4.37
OR2A4	4.66	5.14	4.28
SLC22A15	4.66	4.90	4.23
PSPN	4.66	5.13	3.85
OTTHUMG00000179676	4.66	5.12	4.17
PAK4	4.66	5.16	4.28
VTRNA2-1	4.66	5.16	4.09
PLD6	4.66	5.19	4.16
CPN2	4.66	5.30	4.15
ABCG1	4.66	5.20	4.07
ADCK1	4.66	5.09	4.34
CNGB1	4.66	4.97	4.28
CSK	4.66	4.96	4.21
TRIM61	4.66	5.04	4.35
ZNF709	4.66	5.04	4.26
PSMB11	4.66	5.00	4.36
CCNF	4.66	4.92	4.36
LOC100129455	4.66	4.94	4.31
ZCCHC23	4.66	4.83	4.20
OTTHUMG00000164827	4.66	5.18	4.31
TRAJ43	4.66	5.18	4.16
LIN52	4.66	5.22	4.32
DNAJB8	4.66	5.13	4.30
CDH26	4.66	5.03	4.41
OTTHUMG00000153714	4.66	4.97	4.19
CCL25	4.66	4.76	4.50
SLC17A8	4.65	5.15	4.03
LINC00421	4.65	5.17	4.19
HNRNPCP5	4.65	5.16	4.12
LRRC2-AS1	4.65	5.06	4.19

CLN8	4.65	5.09	4.17
PRR5L	4.65	5.05	4.36
C7orf31	4.65	5.30	4.31
AP1AR	4.65	4.98	4.21
GPR173	4.65	4.99	4.45
C20orf144	4.65	4.92	4.21
MSI1	4.65	4.97	4.32
GJB6	4.65	5.18	4.11
ARNT2	4.65	4.91	4.28
LRFN1	4.65	4.97	4.44
PVRL4	4.65	5.00	4.39
ZCCHC16	4.65	4.89	4.43
YBX3P1	4.65	5.49	4.06
CRABP2	4.65	5.53	3.65
HTR3C	4.65	5.41	4.18
LAMTOR5-AS1	4.65	4.93	4.41
VEPH1	4.65	5.16	4.13
PTGES2-AS1	4.65	4.95	4.25
CCL28	4.65	5.11	4.26
SLC5A12	4.65	4.96	4.37
ZCCHC4	4.65	4.87	4.28
RAB15	4.65	5.23	4.17
LOC339760	4.65	4.96	4.30
HRASLS5	4.65	4.96	4.24
LEFTY2	4.65	5.03	3.94
FLJ25694	4.65	5.79	4.17
MARCKSL1	4.65	5.07	4.36
UBE2M	4.65	5.40	4.29
CABP7	4.65	5.12	3.85
RAD18	4.65	5.06	4.27
ATP5L2	4.65	5.08	4.32
SOX13	4.65	4.97	4.31
LINC00870	4.65	4.95	4.51
OTTHUMG00000153029	4.65	5.42	3.99
CXCR2	4.65	5.26	4.08
RBMY1E	4.65	5.81	3.55
C14orf142	4.65	5.00	4.37
LOC100652770	4.65	5.07	4.14
SLC13A3	4.65	5.01	4.27
LRTOMT	4.65	5.05	4.34
ARHGAP22	4.65	5.20	4.18
OTTHUMG00000002707	4.65	5.20	4.04
UROC1	4.65	4.93	4.44
ZNF140	4.65	5.19	3.97
PFN3	4.65	5.01	4.25
NGF	4.65	6.29	3.95
SPACA4	4.65	5.21	4.06
DMD	4.65	4.97	4.30
MIR4757	4.65	5.31	3.89

ZNF91	4.65	5.55	4.30
GPR45	4.65	5.02	4.33
OTTHUMG00000164273	4.65	5.19	3.95
SLC6A1	4.65	5.03	4.32
LOC100134391	4.65	4.96	4.35
LOC100506691	4.65	5.08	4.11
MIR4746	4.65	5.40	3.93
SLC51B	4.65	4.98	4.17
HOXD-AS1	4.65	4.86	4.37
HMG20B	4.65	5.15	4.30
MST1L	4.65	5.58	4.14
PSG2	4.65	5.49	4.24
SYCE1	4.65	5.14	4.18
EMID1	4.65	5.30	4.27
OTTHUMG00000159353	4.65	5.14	4.21
COL17A1	4.65	5.03	4.26
FOXN3-AS2	4.65	4.91	4.20
OTTHUMG00000151821	4.65	5.16	4.36
SUGT1P1	4.65	5.27	4.28
COMMD10	4.64	5.01	4.37
FAM166A	4.64	5.08	4.43
ALX4	4.64	5.21	3.97
CHP1	4.64	5.03	4.30
ACAD10	4.64	4.84	4.34
GPR158-AS1	4.64	5.10	4.16
GALR3	4.64	5.60	4.24
MORC2-AS1	4.64	5.09	4.23
ZFP82	4.64	5.09	4.18
LOC284600	4.64	5.13	4.12
C7orf63	4.64	5.24	3.80
S100Z	4.64	5.19	4.20
PIGF	4.64	5.24	3.84
BCL2L13	4.64	4.99	4.33
LOC100129046	4.64	5.31	4.06
PCA3	4.64	5.11	4.30
PMS2	4.64	5.12	3.72
GPR156	4.64	4.94	4.32
LOC100131131	4.64	5.28	4.28
OTTHUMG00000163231	4.64	5.05	4.19
RIPK3	4.64	5.18	4.19
TP53TG5	4.64	5.01	3.97
CXADRP2	4.64	5.50	3.85
THNSL1	4.64	4.97	4.37
CLEC14A	4.64	5.56	4.07
LOC100652894	4.64	4.81	4.44
FP2234	4.64	5.02	4.06
CLUH	4.64	5.05	4.04
OTTHUMG00000170339	4.64	5.50	3.88
CMTM8	4.64	5.22	4.27

EML2	4.64	4.96	4.22
ARHGAP25	4.64	5.03	4.20
BTC	4.64	5.66	3.86
NOM1	4.64	5.14	4.36
EXTL3	4.64	4.95	4.19
OTTHUMG00000020273	4.64	5.10	4.12
FBXO48	4.64	5.19	4.10
MIF4GD	4.64	5.00	4.18
LINC00237	4.64	5.22	4.38
LINC00349	4.64	5.08	3.88
ZMYND10	4.64	4.89	4.34
LINC00887	4.64	5.20	4.21
LINC00460	4.64	4.88	4.01
OTUD1	4.64	5.26	3.66
ERMAP	4.64	4.83	4.45
NPFFR1	4.64	5.07	4.06
ZNF678	4.64	5.16	3.94
BTF3L4	4.64	5.07	4.18
SHB	4.64	5.56	4.26
AAGAB	4.64	5.06	4.29
ZNF185	4.64	5.07	4.01
SLC5A5	4.64	5.01	4.12
PPP1R26-AS1	4.64	5.08	4.19
ETV3L	4.64	5.25	4.37
PRMT1	4.64	5.15	4.27
NUDCD1	4.64	4.93	4.37
OTTHUMG00000152852	4.64	5.29	4.16
FAM183A	4.64	5.27	3.87
COBL	4.64	5.08	4.35
RELL2	4.64	4.89	4.34
TMCO4	4.64	5.06	4.19
OTTHUMG00000172124	4.64	5.39	4.01
TRIM29	4.64	4.89	4.43
C1orf115	4.64	5.10	4.26
OTTHUMG00000150805	4.64	5.10	4.24
IFNA5	4.64	5.55	4.04
GPRIN1	4.64	4.91	4.08
FSD1L	4.64	5.01	4.13
PBX4	4.64	4.98	4.37
KIF17	4.64	4.87	4.52
LOC286254	4.64	4.91	4.44
LOC100506007	4.64	5.02	3.97
NT5C1B-RDH14	4.64	4.85	4.28
TMEM158	4.64	5.56	4.20
ZIC2	4.64	5.29	4.17
LOC645638	4.64	5.52	3.81
C19orf44	4.64	4.90	4.28
ACSF2	4.64	5.05	3.99
GABARAPL3	4.64	4.96	4.35

HOXA4	4.64	5.10	4.08
PSCA	4.64	4.97	4.28
MOCOS	4.64	4.91	4.35
ZNF497	4.64	5.36	3.99
LINC00316	4.64	5.03	4.19
PRKCB	4.63	5.04	4.26
OSGEPL1	4.63	5.38	4.11
EPHA4	4.63	5.17	4.25
LINC00898	4.63	5.31	4.32
OR2J2	4.63	5.11	4.12
LRRC37A6P	4.63	5.11	3.61
BICD2	4.63	5.02	4.10
OTTHUMG00000172206	4.63	5.34	3.73
C1orf200	4.63	5.12	3.99
POLA2	4.63	5.00	4.19
GPR171	4.63	5.00	4.26
POGK	4.63	4.88	4.41
OTTHUMG00000016904	4.63	4.96	4.14
CDRT15P2	4.63	5.21	4.06
RASAL2-AS1	4.63	4.95	4.24
ZFP64	4.63	5.05	4.35
PTCSC3	4.63	5.14	4.21
TPST2	4.63	4.92	4.30
OTTHUMG00000019207	4.63	5.00	4.27
ERCC6	4.63	4.90	4.50
TSEN34	4.63	4.81	4.52
SPEG	4.63	4.85	4.20
SIGLEC6	4.63	5.02	4.11
SEC14L4	4.63	4.99	4.20
ASB10	4.63	4.90	4.24
OR9I1	4.63	5.62	4.11
FLVCR2	4.63	5.14	4.35
MZF1	4.63	4.92	4.50
LLGL1	4.63	4.86	4.37
TGFA-IT1	4.63	5.28	4.15
BEGAIN	4.63	4.98	4.11
LOC100506229	4.63	5.01	4.15
HOXA-AS2	4.63	5.01	4.23
PARVG	4.63	4.94	4.43
RNF183	4.63	5.24	4.27
ZNF639	4.63	5.26	4.23
FCAMR	4.63	5.07	4.14
MAGEL2	4.63	4.98	4.38
KCNK18	4.63	4.92	4.17
ZCWPW1	4.63	5.10	4.28
RTN1	4.63	4.91	4.38
RHOBTB2	4.63	5.14	4.24
NR2F6	4.63	5.12	4.24
MIR382	4.63	5.00	4.01

R3HDM4	4.63	5.00	4.42
ABCB9	4.63	4.95	4.22
TSL	4.63	5.05	4.35
PIWIL2	4.63	5.09	4.20
IKBKE	4.63	4.95	3.95
TSTD3	4.63	5.23	3.92
OTTHUMG00000168726	4.63	5.30	4.32
FAIM	4.63	5.02	4.05
TNNI2	4.63	5.14	3.92
TRIM11	4.63	5.03	4.19
OTTHUMG00000170825	4.63	4.99	4.31
SNORA16B	4.63	5.92	3.46
EPB41L4A	4.63	5.19	3.82
PRM2	4.63	5.21	4.20
NANS	4.63	5.07	4.12
OTTHUMG00000164520	4.63	5.94	3.71
POU2F2	4.63	5.16	4.28
C15orf52	4.63	4.90	4.27
IFITM5	4.63	5.08	4.33
RGS6	4.63	5.12	4.27
KCNE1L	4.63	4.99	4.07
PHF2P1	4.63	5.10	4.15
WDR67	4.63	4.85	4.20
SRRM4	4.63	4.96	4.25
RAD54L	4.63	4.99	4.34
NXNL2	4.63	5.02	3.90
PAQR8	4.63	4.90	4.27
MIR4486	4.63	5.78	3.79
FLJ31356	4.63	5.02	4.15
OR6S1	4.63	5.75	4.01
HRH3	4.62	5.01	4.29
LOC150622	4.62	5.54	4.08
OTTHUMG00000037132	4.62	5.27	4.10
OTTHUMG00000018267	4.62	5.30	4.03
ELMO3	4.62	4.91	4.33
H2AFB1	4.62	4.93	4.41
STAC3	4.62	5.04	4.06
TNK1	4.62	5.35	3.99
TSPAN18	4.62	5.39	3.92
MIR93	4.62	5.06	4.02
PLEKHG4	4.62	4.85	4.31
MX2	4.62	5.33	4.11
IL27RA	4.62	4.92	4.34
CCHCR1	4.62	5.00	3.92
MORF4L2-AS1	4.62	5.28	3.87
CMTM5	4.62	5.26	4.31
LOC100134229	4.62	5.22	4.10
LRRC71	4.62	4.85	4.32
LOC100287837	4.62	4.98	4.29

RPL13AP6	4.62	5.16	3.94
OTTHUMG00000155477	4.62	5.23	3.86
MTNR1B	4.62	5.33	4.28
SCARNA20	4.62	5.33	3.79
OTTHUMG00000019462	4.62	6.82	3.19
GPATCH3	4.62	4.81	4.38
RNA5SP394	4.62	5.29	4.15
LOC100144602	4.62	5.05	4.32
FAM71E1	4.62	5.08	4.39
KIAA1211L	4.62	4.96	4.37
ADAMTS16	4.62	5.01	3.88
HOTAIRM1	4.62	4.96	4.31
ECT2	4.62	5.61	3.88
ELL3	4.62	5.14	4.20
LOC100190940	4.62	5.61	4.17
FGGY	4.62	4.94	4.14
PDIK1L	4.62	5.10	4.15
LOXL1-AS1	4.62	4.95	4.22
ZFP28	4.62	5.20	3.98
FRRS1	4.62	5.06	4.29
OTTHUMG00000171672	4.62	5.18	4.26
KIAA1045	4.62	5.08	4.19
LOC728012	4.62	5.19	4.08
PUS7L	4.62	5.35	3.74
GPR65	4.62	5.24	4.09
STOX2	4.62	5.11	4.09
CSF3R	4.62	5.12	4.29
MIR890	4.62	5.26	3.68
IL11	4.62	5.55	3.95
C9orf172	4.62	5.02	4.29
ADRBK2	4.62	5.54	3.92
ENTPD1-AS1	4.62	4.87	4.27
HHLA3	4.62	4.96	4.09
LOC284933	4.62	5.33	4.24
SPTBN4	4.62	4.79	4.28
RAC3	4.62	5.36	4.25
LOC100506631	4.62	5.02	4.04
OR6N2	4.62	5.17	3.79
ARVP6125	4.62	5.35	4.03
PADI4	4.62	5.02	4.20
ITGB7	4.62	4.94	4.36
OTTHUMG00000036405	4.62	5.13	4.19
SLC7A4	4.62	4.92	4.21
STEAP1	4.62	6.01	3.29
FOXP1-AS1	4.62	5.08	4.13
RPS27A	4.62	4.95	4.20
LOC283332	4.62	5.25	4.13
SIGLEC15	4.62	5.05	3.91
LOC100132781	4.62	5.27	4.01

RCC1	4.62	5.62	4.03
GJD2	4.62	5.05	4.19
CCDC176	4.62	4.98	4.08
ANO9	4.62	5.08	4.21
FAM155A	4.62	4.90	3.89
ADAP1	4.62	5.11	4.16
LOC149373	4.62	5.13	4.15
LOC100287313	4.62	5.38	4.05
SSSCA1	4.62	5.10	3.96
VGLL1	4.62	5.10	4.25
KRTAP9-8	4.62	5.14	3.83
ATG10	4.62	5.02	4.15
SFTPC	4.62	4.83	4.26
LOC731282	4.62	5.08	4.40
FAM71E2	4.62	4.86	4.37
HOXC13	4.62	5.17	4.26
SLC9A7	4.62	5.12	4.11
CGB7	4.62	5.24	4.24
ACTRT3	4.62	5.01	4.22
FSD1	4.62	5.03	4.27
OR5D16	4.62	5.58	4.13
FAM65C	4.61	5.15	4.11
PROCA1	4.61	4.85	4.46
TMEM132C	4.61	4.93	4.44
LOC402160	4.61	4.96	4.33
SDR42E2	4.61	4.99	4.06
LIM2	4.61	5.21	4.05
MYCN	4.61	4.99	4.36
PRDM12	4.61	4.81	4.27
GRM5	4.61	5.05	4.21
OTTHUMG00000151781	4.61	5.06	4.04
PCIF1	4.61	4.92	3.95
OTTHUMG00000168211	4.61	4.87	4.32
GNG12-AS1	4.61	4.86	4.36
ZNF543	4.61	5.07	4.09
VAV3	4.61	5.07	4.04
EPHA8	4.61	4.99	4.08
CGRRF1	4.61	5.28	4.25
STMN1	4.61	5.15	3.91
LOC151171	4.61	5.20	3.99
GABRA5	4.61	5.06	4.29
OR4K14	4.61	5.36	4.14
ACOT2	4.61	5.09	3.74
DNAJC24	4.61	4.88	4.14
FAM205A	4.61	5.05	4.00
COX19	4.61	5.12	4.15
MRGPRX2	4.61	5.22	3.88
OTTHUMG00000169765	4.61	4.97	4.07
ACADS	4.61	4.92	4.11

ABCB4	4.61	5.10	4.30
TNFSF9	4.61	5.00	4.06
FLJ40194	4.61	5.39	4.13
SOX6	4.61	5.00	4.22
WDHD1	4.61	5.48	3.88
OTTHUMG00000021439	4.61	5.14	4.03
OTTHUMG00000037030	4.61	5.11	4.27
ZNF442	4.61	4.85	4.15
OTTHUMG00000152890	4.61	5.26	4.24
CES1P1	4.61	5.02	4.15
GRK6	4.61	4.97	4.15
OTTHUMG00000067122	4.61	5.08	4.33
PABPC1P2	4.61	5.02	4.03
SUFU	4.61	4.94	4.32
ROM1	4.61	5.11	4.20
KLC3	4.61	4.89	4.34
KCNT1	4.61	5.26	4.24
LRRC61	4.61	5.10	4.25
OTTHUMG00000151395	4.61	5.13	4.39
PDE3A	4.61	5.16	4.28
NECAB2	4.61	4.90	4.15
CD38	4.61	5.09	4.31
UBQLN4	4.61	4.89	4.11
MIR4772	4.61	5.20	4.23
C17orf80	4.61	5.11	4.05
GCGR	4.61	5.13	4.01
LOC100130507	4.61	5.04	3.98
MPZL2	4.61	5.50	3.86
SLC47A2	4.61	5.12	4.15
OTTHUMG00000171114	4.61	5.15	4.21
FLJ31813	4.61	4.91	4.29
SLC44A5	4.61	5.34	4.27
ZKSCAN2	4.61	5.25	4.28
TAS1R2	4.61	4.93	4.09
ZNF733P	4.61	5.80	3.48
LOC100132111	4.61	4.96	4.19
CCDC65	4.61	4.97	4.33
TBC1D8	4.61	4.96	4.19
ZDHHC13	4.61	4.94	4.10
ANKRD2	4.61	4.84	4.35
RTEL1-TNFRSF6B	4.61	4.80	4.42
FSIP1	4.61	4.98	4.14
LRIT1	4.61	5.12	4.00
SNORD66	4.61	5.15	3.91
GNLY	4.61	5.22	4.05
CLUL1	4.61	5.20	4.17
HES2	4.61	4.87	4.32
SPIN2B	4.61	4.94	4.07
DNAJA1P5	4.61	5.98	3.16

SLC18A3	4.61	5.04	4.34
NOXRED1	4.61	4.88	4.16
CDRT1	4.61	5.01	4.17
KIF2B	4.61	4.81	4.30
MFHAS1	4.61	4.87	4.35
HSD17B14	4.61	5.44	4.18
CNOT6	4.61	4.96	4.39
LOC100507117	4.61	5.14	3.97
SNORD115-20	4.61	5.77	3.32
STXBP4	4.61	4.98	4.33
TROAP	4.61	4.81	4.24
PSG10P	4.61	4.88	4.25
CACNA2D4	4.61	5.18	4.18
OTTHUMG00000162555	4.61	4.86	4.36
ZNF607	4.61	5.01	4.18
ARHGAP6	4.60	5.06	4.28
MANSC1	4.60	5.05	3.97
CCDC140	4.60	4.91	4.15
OTTHUMG00000155650	4.60	4.94	4.32
FBXO34	4.60	5.01	4.16
BFSP1	4.60	5.03	3.91
C6orf211	4.60	5.39	3.73
TAF4B	4.60	4.77	4.24
AMBP	4.60	5.36	4.23
BCL7A	4.60	5.23	4.15
OTTHUMG00000161869	4.60	4.98	4.24
THSD4	4.60	4.92	4.26
VAV2	4.60	4.99	4.18
OR2AT4	4.60	5.13	4.16
MIR769	4.60	5.12	4.08
VASH2	4.60	4.86	4.30
ZNF544	4.60	4.99	4.00
SLC25A22	4.60	4.93	4.32
CNTD1	4.60	5.07	4.34
MAP2K4	4.60	5.14	3.82
HDGFL1	4.60	5.01	4.24
C17orf51	4.60	5.10	4.22
CCDC17	4.60	5.08	4.19
TMEM178B	4.60	5.09	4.25
ISYNA1	4.60	5.00	4.14
CEACAM20	4.60	5.53	3.91
TRAV4	4.60	4.90	4.09
OTTHUMG00000160896	4.60	5.03	4.36
OTTHUMG00000157143	4.60	5.77	3.82
PCBD1	4.60	5.08	3.98
EXOC3L4	4.60	5.12	4.29
ENTPD3-AS1	4.60	5.24	4.00
SPEF2	4.60	4.92	4.35
LOC100996251	4.60	5.13	4.04

SLC1A7	4.60	4.99	4.19
SNX22	4.60	4.97	4.22
TBCC	4.60	4.93	4.22
PLXNA1	4.60	5.16	4.06
ZNF16	4.60	4.82	3.91
SPATA31A7	4.60	5.19	4.15
ZNF18	4.60	4.82	4.00
F12	4.60	5.07	4.14
LINC00960	4.60	5.53	3.34
SCRIB	4.60	5.13	4.01
MAMLD1	4.60	4.86	4.26
RNA5SP410	4.60	5.18	4.14
OTTHUMG00000137385	4.60	4.94	4.19
C1orf127	4.60	5.02	4.16
OTTHUMG00000015619	4.60	5.19	4.09
UHRF1	4.60	4.77	4.30
HSD11B1	4.60	5.18	4.26
ALS2CR12	4.60	5.01	4.37
TENM3	4.60	5.05	4.09
OTTHUMG00000153433	4.60	4.85	4.22
OR56B4	4.60	5.51	3.95
MBLAC2	4.60	4.94	4.33
RAX	4.60	5.09	4.06
STARD13-IT1	4.60	5.08	3.97
ZNF439	4.60	5.23	4.01
RPS4Y1	4.60	5.59	2.85
KLK7	4.60	5.03	4.20
SCN3B	4.60	4.96	4.21
ZUFSP	4.60	5.28	4.09
ZNF345	4.60	4.93	4.47
SYNC	4.60	5.32	4.08
LOC157860	4.60	5.14	3.94
EFTUD1	4.60	5.20	4.15
CARD16	4.60	5.02	4.03
SLCO2B1	4.60	5.52	3.95
DYSF	4.60	4.91	4.27
LOC401068	4.60	5.13	4.12
OTTHUMG00000184009	4.60	5.17	4.17
OTTHUMG00000171590	4.60	5.13	3.96
RPAIN	4.60	4.98	4.14
AGXT	4.60	5.13	3.89
NCKIPSD	4.60	5.05	4.32
GIGYF1	4.60	5.15	4.22
C1orf65	4.60	5.00	4.07
ZNF618	4.60	5.12	4.32
GCNT3	4.60	5.06	4.20
ANAPC10	4.60	4.89	4.30
EHD1	4.60	4.88	4.40
SCAND1	4.60	4.93	3.92

HOXC11	4.60	4.94	4.28
FLJ27354	4.60	4.81	4.30
LOC100996378	4.60	4.93	4.23
MAFG-AS1	4.60	5.00	4.13
WDYHV1	4.60	5.01	4.23
OTTHUMG00000010575	4.60	4.96	4.18
MDM1	4.59	4.90	4.28
C1QL1	4.59	4.97	4.11
OTTHUMG00000019095	4.59	4.95	4.27
MIR1295A	4.59	5.12	4.11
GCRG224	4.59	5.09	3.65
MEIS1-AS3	4.59	5.03	3.90
FAM66D	4.59	5.48	3.59
KRTAP1-1	4.59	5.22	4.11
ELFN1	4.59	5.05	3.81
OLFM2	4.59	4.87	4.42
FLJ31662	4.59	5.02	3.90
SNORD116-21	4.59	5.37	3.45
NCR1	4.59	4.83	4.02
OTTHUMG00000163891	4.59	4.94	4.02
UBE2N	4.59	4.82	4.22
VSTM1	4.59	5.11	3.81
C10orf2	4.59	4.86	4.34
CHEK1	4.59	5.13	4.14
OTTHUMG00000019263	4.59	4.88	4.31
STXBP6	4.59	4.84	4.16
ARHGEF5	4.59	5.13	4.13
ERN1	4.59	4.85	4.20
OTTHUMG00000152992	4.59	5.07	3.93
SHH	4.59	4.95	4.31
C1orf109	4.59	4.90	4.37
EGR3	4.59	4.91	4.15
ANKS4B	4.59	5.19	3.95
KSR2	4.59	5.08	4.01
GAB3	4.59	5.22	4.17
TRPM5	4.59	4.95	4.14
MBNL3	4.59	5.23	3.73
FAM221A	4.59	4.89	4.05
PIK3CD	4.59	4.96	4.08
OR51G2	4.59	5.00	4.07
C7orf50	4.59	5.12	4.25
ZNF132	4.59	5.14	4.18
OTTHUMG00000151498	4.59	4.82	4.30
GAL	4.59	4.87	4.12
LOC100131138	4.59	4.77	4.13
SRPX	4.59	5.03	4.21
LOC100507362	4.59	4.81	4.42
TOMM34	4.59	4.87	4.19
UAP1L1	4.59	4.88	4.30

SNORA49	4.59	5.37	3.72
C9orf37	4.59	4.98	4.00
PRELID2	4.59	4.98	4.13
CAMKV	4.59	4.93	4.28
DDO	4.59	4.82	4.14
RNU12-2P	4.59	5.22	3.92
OTTHUMG00000155977	4.59	5.20	4.13
LOC729291	4.59	5.07	4.23
HEPH	4.59	4.88	4.27
PAN3-AS1	4.59	4.85	4.29
LTB4R	4.59	4.97	4.06
VPREB1	4.59	4.81	4.34
ALG9-IT1	4.59	5.01	3.81
P4HA2-AS1	4.59	5.11	4.10
PAPPA2	4.59	4.95	3.98
OTTHUMG00000178141	4.59	4.93	4.16
JMJD7-PLA2G4B	4.59	4.98	4.33
ERVMER61-1	4.59	5.22	4.01
FAM114A2	4.59	4.86	4.25
NDP	4.59	5.29	3.55
IGSF11	4.59	5.15	4.27
FBXO39	4.59	5.12	4.24
LOC100507449	4.59	4.93	4.20
UTP23	4.59	4.97	4.01
NPC1L1	4.59	5.16	3.94
PROKR1	4.59	5.11	4.03
MYBL1	4.59	5.79	3.69
NUDT4P1	4.59	5.03	3.78
TSPAN16	4.59	5.18	4.20
LGALS7	4.59	4.95	4.37
TAS2R3	4.59	5.02	4.02
LOC100506124	4.59	5.39	3.66
RNA5SP467	4.59	5.26	3.92
NPR1	4.59	5.01	4.02
LRRC45	4.58	4.92	4.02
SH2D3A	4.58	5.38	4.15
EBF4	4.58	4.90	4.15
PLCL2-AS1	4.58	5.22	3.97
RAB11FIP1	4.58	5.03	4.27
ACTL9	4.58	4.93	4.41
CHRNA2	4.58	4.90	4.32
PLA2G2D	4.58	5.06	4.03
RDH16	4.58	5.50	4.04
TMEM198	4.58	5.10	3.84
TMEM38B	4.58	4.90	3.95
ABCA2	4.58	4.76	4.19
KLHL30	4.58	5.20	4.22
CHRNG	4.58	4.84	4.43
IFFO2	4.58	4.90	4.18

ZSWIM3	4.58	5.17	4.20
TRIM48	4.58	5.02	3.89
PEBP4	4.58	5.00	4.28
MIR4681	4.58	5.15	3.07
OTTHUMG00000020685	4.58	4.94	3.95
CRYGN	4.58	5.14	4.32
LOC100506917	4.58	5.04	4.07
DSTYK	4.58	5.21	4.11
KCNK3	4.58	5.31	4.22
FLJ36000	4.58	5.22	4.24
ZNF474	4.58	5.64	4.02
OTTHUMG00000151435	4.58	4.90	4.06
DCST2	4.58	4.94	4.18
GPX5	4.58	5.16	3.82
OTTHUMG00000012112	4.58	5.39	4.21
ABCA7	4.58	5.19	4.08
OTTHUMG00000180065	4.58	4.91	4.02
LOC100507018	4.58	5.01	4.18
PP14571	4.58	4.91	4.14
KIF20B	4.58	5.18	3.91
WDR12	4.58	5.04	4.03
L1TD1	4.58	4.99	4.12
ZNF473	4.58	5.07	4.10
PTPRVP	4.58	5.09	4.20
ZNF224	4.58	4.88	3.80
OAS2	4.58	5.08	4.12
ZMAT4	4.58	5.01	4.33
ZNF192P1	4.58	4.86	4.37
OTTHUMG00000168322	4.58	4.81	4.33
TOR4A	4.58	4.80	4.16
ZNF282	4.58	4.95	4.12
LY6G5C	4.58	4.87	4.27
RNA5SP509	4.58	5.18	3.90
AMMECR1	4.58	4.93	4.09
CCDC30	4.58	5.89	3.38
CCDC70	4.58	5.05	4.10
CABP2	4.58	5.01	3.97
GAPDHS	4.58	4.79	4.16
OTTHUMG00000151749	4.58	4.94	4.26
SNORD16	4.58	5.22	3.50
MVB12B	4.58	4.81	4.18
OTTHUMG00000164015	4.58	5.21	3.99
RPA4	4.58	5.04	4.11
GPC5	4.58	5.74	3.39
ESR2	4.58	4.81	4.38
DUOX1	4.58	4.85	3.97
AQP12B	4.58	4.98	4.13
CDX1	4.58	4.90	4.17
DDX28	4.58	5.00	3.81

LINC00086	4.58	5.27	3.84
LMNB1	4.58	5.36	4.22
OTTHUMG00000153313	4.58	4.92	4.31
SLAMF9	4.58	5.17	4.07
C6	4.58	5.39	3.96
LARS2-AS1	4.58	4.92	4.18
TTC22	4.58	5.04	4.22
NPPB	4.58	4.89	4.16
TEN1-CDK3	4.57	5.04	4.11
OTTHUMG00000159262	4.57	5.01	4.12
MIR378D2	4.57	5.07	4.19
OTTHUMG00000153159	4.57	5.26	3.74
GRIA1	4.57	5.27	4.10
LOC645249	4.57	4.95	4.20
POU6F2	4.57	4.86	4.20
OTTHUMG00000020570	4.57	5.06	3.95
PGLYRP3	4.57	5.00	4.10
TPRN	4.57	4.87	4.09
CARD17	4.57	4.76	4.34
KPTN	4.57	5.04	4.21
LOC100506207	4.57	4.89	4.19
HS1BP3	4.57	4.89	4.22
JAG2	4.57	5.05	4.18
ABCG4	4.57	4.85	4.15
SUV39H2	4.57	5.04	4.10
LOC100506413	4.57	5.26	3.95
ADAD2	4.57	5.08	4.30
OTTHUMG00000168357	4.57	4.82	4.21
EGR2	4.57	4.89	4.16
GBAS	4.57	4.92	4.27
C1orf213	4.57	4.81	4.32
LOC100506947	4.57	5.00	4.14
WNT4	4.57	4.99	4.18
SIGLEC5	4.57	4.95	4.09
LOC100128076	4.57	4.86	4.17
OTTHUMG00000165321	4.57	5.01	4.09
KLHL12	4.57	4.86	4.01
C14orf169	4.57	4.90	4.24
OTTHUMG00000153867	4.57	5.00	4.03
SSX2IP	4.57	5.17	4.29
LSM12	4.57	5.39	3.59
SRPK3	4.57	4.91	4.25
LINC00251	4.57	4.98	4.03
RASGRF1	4.57	5.07	4.09
FAM170A	4.57	5.08	3.70
FBLN2	4.57	5.30	3.58
ZC3HC1	4.57	4.88	4.28
OTTHUMG00000012183	4.57	5.33	3.94
TRAM2-AS1	4.57	5.08	4.22

SNX32	4.57	4.96	4.18
CHIC1	4.57	4.90	4.21
LOC284632	4.57	5.15	4.14
OTTHUMG00000167041	4.57	5.20	4.01
RASGRF2	4.57	5.11	4.03
RNU7-47P	4.57	5.23	3.79
MIR4300	4.57	5.18	4.19
LOC100507462	4.57	5.40	4.00
DPPA5	4.57	5.09	4.05
TMEM236	4.57	5.20	4.03
DCST1	4.57	5.00	4.16
HMGB3P1	4.57	5.45	3.79
MIR548A2	4.57	5.47	3.93
PRKAG3	4.57	4.86	4.29
OTTHUMG00000152442	4.57	5.12	4.01
CCDC180	4.57	4.83	4.36
GJB3	4.57	5.09	4.25
TUFT1	4.57	4.86	4.13
ZNF101	4.57	4.86	4.32
TIFAB	4.57	5.31	4.06
LOC100131289	4.57	4.91	4.11
ANP32A	4.57	4.80	4.04
ORC1	4.57	4.87	4.13
SLC22A13	4.57	5.13	4.17
GRIN2C	4.57	4.89	3.96
ETV4	4.57	5.08	4.17
OTTHUMG00000151775	4.57	5.04	4.14
ITGB2-AS1	4.57	5.10	4.00
STMN4	4.57	4.98	4.16
LOC100128594	4.57	4.93	4.17
LRRC4	4.57	4.86	4.17
FAM211A	4.57	5.01	4.18
AKR1C3	4.57	5.28	4.02
LOC148638	4.57	5.44	4.21
KIR3DL1	4.57	5.62	3.28
SNORA46	4.57	5.50	3.96
H1FNT	4.57	4.94	3.71
CXorf48	4.57	4.91	4.13
GABRB3	4.57	5.15	4.11
MT3	4.57	5.48	3.63
OTTHUMG00000153755	4.57	4.95	4.11
C19orf35	4.56	5.12	3.72
CRHR1-IT1	4.56	4.97	4.15
SOCS2-AS1	4.56	4.95	4.06
DCUN1D2	4.56	4.90	4.06
AIM1L	4.56	5.07	4.20
LOC653160	4.56	4.88	4.26
CIDEA	4.56	5.01	4.21
SP140	4.56	5.04	4.12

INHBB	4.56	4.82	4.12
OAS3	4.56	5.00	4.19
TMC3	4.56	4.90	4.07
SPANXB1	4.56	4.85	4.30
FAM127A	4.56	5.03	4.11
COL6A6	4.56	5.01	3.83
ELFN2	4.56	4.93	4.22
OTTHUMG00000167502	4.56	5.16	4.05
FAM179A	4.56	5.39	4.04
FAM83A-AS1	4.56	4.87	4.33
NEURL4	4.56	4.81	4.23
LEFTY1	4.56	5.08	4.33
CDC7	4.56	5.18	4.05
TMEM132E	4.56	5.03	4.33
LINC00967	4.56	4.84	4.32
F8	4.56	4.69	4.09
FAM215A	4.56	5.13	3.72
KLHDC8B	4.56	5.06	4.01
FAT3	4.56	5.02	3.69
MIR425	4.56	5.05	4.01
CCDC69	4.56	4.95	4.23
LCA10	4.56	5.42	3.93
PITPNM2	4.56	4.95	4.25
TIMELESS	4.56	4.94	4.33
OTTHUMG00000017550	4.56	5.18	4.17
NKIRAS2	4.56	5.03	4.10
AQP6	4.56	5.21	3.94
MRGPRF	4.56	5.14	3.89
OTTHUMG00000018883	4.56	5.05	4.18
RFX5	4.56	4.73	4.34
NHS	4.56	5.02	4.15
FANK1	4.56	4.79	4.21
PNO1	4.56	5.14	4.00
GPR150	4.56	5.14	4.15
RPS15AP10	4.56	5.17	3.76
OTTHUMG00000015399	4.56	5.42	4.28
OTTHUMG00000035513	4.56	5.16	3.68
BTNL9	4.56	5.03	4.12
CTH	4.56	5.30	3.84
DACH1	4.56	5.16	4.12
SFXN1	4.56	5.12	4.12
TSHR	4.56	5.03	4.02
HOXB8	4.56	4.95	4.37
LINC00404	4.56	4.86	4.26
ANKRD9	4.56	4.90	4.29
NANOS2	4.56	5.47	3.95
HLCS-IT1	4.56	5.10	4.12
BARD1	4.56	4.92	4.04
CCNO	4.56	4.96	4.25

OSCAR	4.56	5.00	4.29
ADAMTS15	4.56	5.05	4.29
OR4C6	4.56	5.04	4.19
C9orf66	4.56	5.14	4.19
ART4	4.56	4.90	4.17
MID1	4.56	4.95	3.97
SLC5A6	4.56	4.84	4.27
MAST3	4.56	5.13	4.13
HSH2D	4.56	4.82	4.21
SHMT1	4.56	4.95	4.28
OTTHUMG00000017942	4.56	4.82	4.04
STK17B	4.56	5.17	3.95
MAFK	4.56	4.95	4.25
DNAAF1	4.56	4.88	4.13
RNA5SP405	4.56	5.06	3.74
SYCP2	4.56	5.11	4.10
DRC1	4.56	4.76	4.28
DDX43	4.56	4.77	4.22
C3orf83	4.56	5.05	4.22
EHMT2-AS1	4.56	4.94	4.09
CALHM3	4.56	5.27	3.90
ZNF788	4.56	5.15	3.86
CCL4L2	4.56	5.41	4.16
CACNA1C-IT3	4.56	5.23	3.99
KLHDC7B	4.56	5.27	4.15
IL22RA1	4.56	4.85	4.31
CDY2B	4.56	5.07	4.05
ZNF850	4.56	5.03	4.06
PDZD9	4.56	4.82	4.13
GYG1	4.56	4.96	4.10
PIFO	4.56	5.53	3.90
EMC6	4.56	5.13	3.68
GABBR2	4.56	4.87	4.37
PSMD4	4.56	4.98	4.12
CFLAR-AS1	4.56	4.86	4.34
TMPRSS4	4.56	5.09	4.25
LINC00111	4.56	5.11	4.20
OR10C1	4.56	4.97	3.99
CFP	4.56	4.98	4.19
KCNIP3	4.56	5.13	3.49
OTTHUMG00000154105	4.56	4.83	4.31
AP5S1	4.55	5.10	3.91
MS4A10	4.55	4.87	4.18
LOC100506113	4.55	5.14	3.79
FLJ44511	4.55	4.79	4.41
CLEC5A	4.55	5.38	3.91
KLHL26	4.55	4.84	4.02
CYP3A7-CYP3AP1	4.55	4.97	3.97
C1D	4.55	5.11	4.05

FBXO45	4.55	4.82	4.05
PRICKLE4	4.55	5.01	4.02
ENDOU	4.55	4.83	4.18
MATN1-AS1	4.55	4.83	4.21
KRT4	4.55	5.03	4.13
IGLV3-21	4.55	5.14	3.86
MYOZ1	4.55	4.96	4.35
KRTAP10-5	4.55	5.29	3.34
ADAMTS17	4.55	5.07	3.91
ITGA2B	4.55	4.94	4.31
IQCD	4.55	4.81	4.33
C11orf45	4.55	5.11	4.21
IGHD	4.55	4.87	4.12
C16orf52	4.55	4.98	4.10
LYRM4	4.55	4.87	4.26
ZNF257	4.55	5.34	3.83
KRT15	4.55	4.89	4.27
LOC100505679	4.55	4.83	4.09
LOC100506928	4.55	5.72	4.16
ZNF33B	4.55	5.66	3.64
SNORD91B	4.55	5.30	3.44
OTTHUMG00000158357	4.55	4.97	4.13
P2RX7	4.55	5.16	4.11
SCARF2	4.55	4.83	4.36
TRIM32	4.55	5.14	3.81
CEBPA-AS1	4.55	5.24	4.11
SEPT3	4.55	5.01	4.19
ZC2HC1C	4.55	4.82	4.18
BTBD18	4.55	4.91	4.14
HTT-AS1	4.55	5.08	4.07
IRAK1	4.55	5.18	4.11
FAM171A1	4.55	4.86	4.30
ATP6V1H	4.55	4.93	4.04
SLC1A3	4.55	4.78	4.31
C1QTNF2	4.55	5.09	4.05
HKDC1	4.55	5.04	4.21
MYADML	4.55	4.93	3.48
LOC100128591	4.55	4.91	4.03
RGS11	4.55	5.16	4.25
EBF3	4.55	4.97	4.02
OTTHUMG00000164820	4.55	4.99	4.23
LOC440602	4.55	4.86	4.37
RHOA-IT1	4.55	4.88	4.16
ATAD2	4.55	5.13	4.07
HINT2	4.55	4.89	4.14
IRS2	4.55	4.88	3.97
OTTHUMG00000021338	4.55	4.82	4.14
RHBDD1	4.55	4.92	4.16
CLCNKA	4.55	5.22	4.11

ADARB2-AS1	4.55	5.09	3.94
NAG20	4.55	5.25	3.88
KRTAP5-11	4.55	5.09	3.96
NRXN3	4.55	4.93	3.96
LRP4-AS1	4.55	5.00	4.09
APCDD1L-AS1	4.55	5.92	3.91
NT5DC3	4.55	4.77	4.23
CHST13	4.55	4.79	4.05
ZBTB7C	4.55	5.03	4.07
GRASP	4.55	4.85	4.01
SPATA31A2	4.55	5.09	4.08
LOC100506679	4.55	4.94	4.19
OR9G1	4.55	5.07	3.67
LINC00322	4.55	5.10	4.01
OTTHUMG00000164954	4.55	5.00	4.13
BMP15	4.55	4.92	4.17
ZNF304	4.55	4.96	4.05
LOC285819	4.55	5.02	4.26
CXorf56	4.55	4.87	4.23
IL17RD	4.55	4.94	4.03
TMEM163	4.55	5.08	4.22
CCKBR	4.55	4.87	4.32
CXorf67	4.55	4.94	4.16
TESK2	4.55	4.73	4.18
RBKS	4.55	4.96	4.17
LRRK2	4.55	5.22	3.90
LOC100996269	4.55	5.12	3.80
CCL23	4.55	5.08	3.69
GAPT	4.55	4.76	4.19
TBC1D22B	4.55	5.20	4.14
SLC25A42	4.55	4.90	4.22
RNA5SP414	4.55	4.94	4.09
OTTHUMG00000182164	4.54	4.94	4.12
TAF1A	4.54	5.11	4.13
OTTHUMG00000168496	4.54	5.23	4.15
GDPD1	4.54	5.28	3.78
CHRNA1	4.54	5.11	3.60
OTTHUMG00000040659	4.54	5.46	3.73
PCDHB2	4.54	5.09	3.77
KAT5	4.54	5.02	3.81
OR2Z1	4.54	5.20	3.91
PEAR1	4.54	5.11	3.71
OCM	4.54	5.02	3.72
BAI1	4.54	4.95	4.21
SELRC1	4.54	5.12	4.18
SGK2	4.54	4.94	4.19
KEL	4.54	5.33	4.10
WDR25	4.54	4.98	4.19
NCKAP5	4.54	5.04	3.89

MYL3	4.54	4.89	4.27
MIR328	4.54	5.62	3.80
CD28	4.54	5.15	3.68
OTTHUMG00000086723	4.54	4.89	3.87
SSTR4	4.54	5.03	3.95
NDRG4	4.54	4.91	4.23
ZNF408	4.54	4.79	4.42
TMEM61	4.54	4.88	4.24
ZNF736	4.54	5.08	4.10
SLIT2-IT1	4.54	4.98	4.14
PADI2	4.54	5.23	4.20
PAGE2	4.54	4.98	3.65
ZCCHC18	4.54	4.82	4.06
LOC100506997	4.54	4.96	4.24
DDX11L2	4.54	5.06	3.92
TMEM167A	4.54	4.94	3.94
EGFLAM	4.54	4.90	4.03
TMEM101	4.54	4.97	4.20
OTTHUMG00000032885	4.54	5.05	4.12
CCDC159	4.54	4.85	4.22
PUS7	4.54	4.99	4.02
LRRC8B	4.54	4.77	4.29
PISD	4.54	4.90	4.14
EPN2-IT1	4.54	4.78	4.08
LCN15	4.54	4.66	4.23
RUNDC3B	4.54	5.16	4.17
LOC100270804	4.54	4.72	4.27
DISP2	4.54	5.15	4.09
HHATL	4.54	4.99	4.10
TBC1D10A	4.54	4.86	4.33
PIN1P1	4.54	5.05	4.12
LOC100996637	4.54	4.72	4.23
IER5	4.54	4.79	3.97
MIR580	4.54	4.98	3.63
OTTHUMG00000002490	4.54	5.12	3.88
CIT	4.54	5.02	4.22
CLTCL1	4.54	4.93	4.27
OTTHUMG00000170815	4.54	4.87	4.28
TCF7	4.54	4.99	4.10
SLITRK5	4.54	5.18	4.04
GPC4	4.54	5.13	3.96
ACOX2	4.54	4.98	4.26
ITGAD	4.54	4.91	4.20
LOC389332	4.54	4.92	4.29
MAP6D1	4.54	5.23	4.13
ZDHHC11	4.54	5.13	4.18
FAT2	4.54	5.06	4.01
ABHD16A	4.54	4.84	4.30
ALKBH4	4.54	5.08	4.13

MYL1	4.54	4.97	3.92
OTTHUMG00000163492	4.54	4.89	4.02
HDAC11	4.54	4.99	4.20
CHCHD1	4.54	5.40	3.47
PROKR2	4.54	4.88	3.94
PCSK2	4.54	4.70	4.23
LOC100996613	4.54	5.30	3.98
ZNF837	4.54	4.85	4.09
RNF5P1	4.54	5.17	4.13
HTR7P1	4.54	5.15	4.27
SLC39A5	4.54	4.97	4.18
CCDC179	4.54	4.94	4.03
ZAN	4.54	4.86	4.19
NBPF3	4.54	4.94	4.02
MGAT4A	4.54	5.46	3.56
OTTHUMG00000169494	4.54	4.93	4.19
VRK2	4.54	4.84	3.76
SPINT1	4.54	4.91	4.16
FGFR3	4.54	5.07	3.87
DPF3	4.54	4.90	4.27
RNU6-81P	4.54	5.30	3.80
ZMAT5	4.54	5.12	4.20
CDC14A	4.53	4.80	4.25
XIRP1	4.53	4.88	4.11
FLJ34223	4.53	5.12	4.19
OCM2	4.53	5.03	3.66
MAP7	4.53	4.77	4.14
LOC654780	4.53	4.89	4.27
ADRB1	4.53	4.89	4.12
NPHS1	4.53	4.97	4.05
IRF2BP1	4.53	4.88	3.95
ITIH1	4.53	4.96	4.03
KRT84	4.53	5.18	4.18
LRGUK	4.53	4.81	4.28
GFOD1	4.53	4.89	4.26
OTTHUMG00000152758	4.53	5.14	3.96
OTTHUMG00000182863	4.53	4.97	4.24
NPHP3-AS1	4.53	5.27	4.24
ARRDC2	4.53	4.82	4.24
CYP4F8	4.53	5.11	3.85
OTTHUMG00000074550	4.53	5.07	3.97
GPR133	4.53	5.05	3.95
DHRS2	4.53	4.74	4.24
REM2	4.53	5.04	4.14
MIR3972	4.53	4.92	3.78
CYP3A5	4.53	5.13	4.12
KCNA4	4.53	5.02	4.14
KNG1	4.53	5.14	3.98
EFNA3	4.53	5.28	4.09

LIF	4.53	5.86	3.84
ATP1B4	4.53	4.93	4.23
NKX2-8	4.53	4.96	4.14
BPI	4.53	5.12	3.82
PDX1	4.53	5.04	4.10
HEPHL1	4.53	4.78	4.24
OTTHUMG00000165102	4.53	4.84	4.19
GBX2	4.53	4.87	4.27
LOC100128028	4.53	4.80	4.31
GLI1	4.53	5.02	4.00
CNIH3	4.53	4.90	4.11
PRX	4.53	4.88	4.02
DGCR2	4.53	4.98	4.26
FASTKD3	4.53	5.04	3.96
TPTE2P6	4.53	5.09	3.64
MYPOP	4.53	4.77	4.22
CD160	4.53	4.83	4.27
POTEKP	4.53	4.99	4.21
FSCN3	4.53	4.81	4.20
MIR150	4.53	4.89	4.10
NCF1	4.53	5.30	3.86
LOC100507568	4.53	4.89	4.08
LOC100506421	4.53	5.34	3.64
WDFY3-AS2	4.53	5.31	3.52
MFSD9	4.53	4.91	4.23
SETD6	4.53	4.78	4.17
FAM161A	4.53	4.92	3.85
SLC9A3	4.53	4.85	3.89
FAM89A	4.53	4.81	4.33
ARSD	4.53	4.83	4.18
AMZ1	4.53	4.96	4.06
BVES	4.53	5.31	3.79
ZRANB2-AS1	4.53	4.87	4.25
MIR4328	4.53	4.99	3.30
ZNF600	4.53	5.54	3.88
DEPTOR	4.53	4.88	4.15
RUFY4	4.53	4.98	4.09
LOC100996876	4.53	4.97	4.17
DGKQ	4.53	4.87	4.19
LMX1A	4.53	4.76	4.03
KCNS1	4.53	5.07	4.15
ESRRA	4.53	5.14	4.29
MIR4421	4.53	5.32	3.64
TESPA1	4.53	4.92	4.14
TBCEL	4.53	5.07	4.09
TBX3	4.53	4.92	4.27
MBP	4.53	4.92	4.11
HLA-F-AS1	4.53	5.08	4.08
HP09025	4.53	4.92	4.22

FAM188B2	4.53	4.95	4.30
PRIMA1	4.52	5.08	4.04
WNT3A	4.52	4.73	4.17
CAPN11	4.52	4.82	3.89
KRT3	4.52	5.07	3.91
LOC100129138	4.52	4.91	4.19
TMEM151B	4.52	4.82	4.27
MRPL44	4.52	5.08	3.87
MORN1	4.52	4.87	4.17
LOC100129961	4.52	5.02	4.05
HSF2BP	4.52	4.86	4.14
CRX	4.52	4.93	3.88
SPAG4	4.52	5.21	3.96
LOC101060305	4.52	5.07	4.00
CCIN	4.52	4.69	4.31
CHKB-AS1	4.52	4.95	4.20
LENG1	4.52	4.87	4.34
FGF18	4.52	4.94	4.06
CYB5RL	4.52	4.74	4.11
KYNU	4.52	5.96	3.67
PPIAL4G	4.52	5.29	4.07
MIR3138	4.52	4.80	4.15
RNA5SP423	4.52	5.39	4.06
MACROD1	4.52	4.95	4.06
OTTHUMG00000163226	4.52	4.88	4.04
METTL21D	4.52	4.86	4.16
FAM86C2P	4.52	4.77	4.03
CYFIP2	4.52	4.89	4.02
IL36A	4.52	5.33	4.16
GRIA3	4.52	4.88	4.04
SLC1A6	4.52	5.01	3.94
TMEM229B	4.52	5.00	4.20
C1orf189	4.52	4.97	4.03
C2CD2L	4.52	4.80	4.12
RTN4RL2	4.52	4.88	4.16
PPARGC1B	4.52	4.84	4.08
C11orf84	4.52	4.86	4.21
GPR26	4.52	4.94	3.99
MYCT1	4.52	5.73	2.80
OTTHUMG00000155983	4.52	4.94	4.12
HPX	4.52	4.88	3.84
TMEM67	4.52	4.80	3.80
RNA5SP332	4.52	5.54	3.68
SNORD114-16	4.52	6.13	3.49
OTTHUMG00000024161	4.52	4.90	4.06
TRIM54	4.52	4.70	4.19
OTTHUMG00000163345	4.52	5.26	4.11
OTTHUMG00000150069	4.52	5.18	3.97
MECR	4.52	5.00	4.15

MEF2BNB-MEF2B	4.52	4.80	4.33
CKMT1B	4.52	5.13	4.05
AADACL3	4.52	5.08	3.96
GOLGA2P5	4.52	5.32	3.68
OTTHUMG00000008375	4.52	4.75	4.25
MAP3K7CL	4.52	4.94	4.04
WRAP53	4.52	4.65	4.40
ACHE	4.52	4.92	4.13
NCLN	4.52	4.68	4.19
C12orf66	4.52	4.97	4.14
LCA5L	4.52	4.90	4.26
OR6Y1	4.52	5.28	3.49
C4orf19	4.52	5.05	4.27
MAGEA8-AS1	4.52	4.87	4.17
C2orf43	4.52	5.07	3.99
FBXL12	4.52	4.78	3.90
PNPLA1	4.52	4.69	4.28
AKAP7	4.52	5.02	4.08
MRI1	4.52	5.03	3.82
ESPNL	4.52	5.34	3.68
MEIS1-AS2	4.52	5.00	4.22
SPOCK2	4.52	5.23	4.13
PALM2-AKAP2	4.52	4.89	4.16
TTC30A	4.52	5.10	4.15
KCNA5	4.52	5.26	3.73
RASGEF1A	4.52	4.96	3.95
FNDC8	4.52	4.99	4.23
C2orf57	4.52	5.23	3.98
LIN54	4.52	4.81	4.12
GFRA1	4.52	5.11	4.02
ZNF281	4.52	5.01	4.08
FGD3	4.52	5.17	3.99
FLVCR1-AS1	4.52	5.24	3.99
PSAPL1	4.51	4.72	4.37
OTTHUMG00000165860	4.51	5.15	3.95
BEST4	4.51	4.80	4.22
GRIA2	4.51	5.11	3.73
TBC1D24	4.51	4.76	4.20
LIPG	4.51	4.91	4.22
C14orf64	4.51	4.99	4.25
DGKG	4.51	4.96	4.06
LOC100128505	4.51	4.94	4.06
NPBWR1	4.51	4.85	4.16
MED25	4.51	4.82	4.15
FGF12	4.51	5.15	3.97
ABCG2	4.51	5.06	4.19
FUNDC1	4.51	4.94	4.15
PRM3	4.51	4.75	4.00
C14orf180	4.51	4.98	4.23

OTTHUMG00000167737	4.51	4.96	3.81
LOC284798	4.51	4.85	4.06
ABRA	4.51	4.88	4.02
LOC100134361	4.51	4.89	4.21
ZC3H10	4.51	4.78	4.23
SNORA71B	4.51	5.52	3.28
LOC400748	4.51	4.97	4.06
LOC283177	4.51	4.94	3.90
AMPD3	4.51	5.18	3.99
OTTHUMG00000165780	4.51	5.09	3.93
SLC35G2	4.51	4.80	4.22
OTTHUMG00000016846	4.51	5.07	3.95
OR1J2	4.51	5.17	4.08
CTNS	4.51	4.74	4.16
LOC100506257	4.51	4.89	3.99
HECW1	4.51	4.93	4.19
IRX1	4.51	4.85	4.12
GRK4	4.51	4.73	4.20
RPS6KA5	4.51	4.96	4.09
KIAA0825	4.51	4.88	4.14
DCDC2	4.51	4.81	4.00
ZNF433	4.51	4.76	4.28
LOC100129215	4.51	4.90	4.08
PF4V1	4.51	5.06	3.97
ALOX5	4.51	5.52	4.01
OTTHUMG00000086463	4.51	4.94	4.08
OTTHUMG00000169117	4.51	4.87	4.18
FGF14	4.51	5.15	3.92
OTTHUMG00000172569	4.51	4.82	4.11
WFDC1	4.51	4.87	4.19
NPPA	4.51	4.91	4.18
GPR132	4.51	4.82	4.11
FAIM3	4.51	4.73	4.10
LOC100505639	4.51	5.18	4.30
EFCAB1	4.51	5.13	3.46
BTK	4.51	5.23	3.80
CYP4A11	4.51	5.16	3.70
MIXL1	4.51	4.86	4.14
LOC285889	4.51	5.15	4.06
PPP1R16B	4.51	4.92	4.02
ARMC5	4.51	4.87	4.24
BTN3A2	4.51	5.24	4.14
RNA5SP415	4.51	4.98	3.81
LRRC49	4.51	4.79	4.04
LINC00649	4.51	4.90	4.14
LRRN3	4.51	5.04	3.98
C1orf105	4.51	4.96	3.95
LHX8	4.51	5.05	4.02
FAM151A	4.51	4.92	4.14

ITPKC	4.51	4.90	3.97
OTTHUMG00000162106	4.51	5.00	4.05
MYOM1	4.51	4.80	3.94
ACSM4	4.51	4.98	4.25
HSF5	4.51	4.91	4.02
RNLS	4.51	4.79	4.11
RAPGEF4-AS1	4.51	5.01	4.04
CD207	4.50	5.02	3.58
B3GALT1	4.50	4.79	4.18
CBFA2T3	4.50	4.76	4.13
OTTHUMG00000164249	4.50	4.97	4.15
CKB	4.50	5.16	3.49
OTTHUMG00000018174	4.50	4.85	4.18
DCAF4L2	4.50	5.04	3.97
SNURFL	4.50	5.20	3.90
LOC100506813	4.50	5.02	3.99
LAMA1	4.50	4.69	4.31
MAS1	4.50	4.84	4.21
ZDHHC20-IT1	4.50	4.94	4.10
MIR4326	4.50	5.23	3.06
C8orf47	4.50	4.74	4.17
COX6A2	4.50	5.00	3.95
OTTHUMG00000168310	4.50	5.33	3.93
CDPF1	4.50	4.83	4.10
CENPBD1	4.50	4.71	4.19
GADD45G	4.50	4.85	4.11
WDR11-AS1	4.50	4.71	4.31
AGRP	4.50	4.77	4.12
SRBD1	4.50	4.95	4.02
SWT1	4.50	5.04	3.96
UMOD	4.50	4.96	3.88
HPN-AS1	4.50	5.02	3.95
DIEXF	4.50	4.73	4.07
LOC100270680	4.50	4.92	3.88
ASAH2	4.50	5.16	3.59
NEFM	4.50	5.30	4.01
OR6Q1	4.50	5.03	4.03
CDH2	4.50	4.92	4.01
WFDC10B	4.50	5.18	3.85
UBL4A	4.50	4.82	4.15
SP9	4.50	4.80	4.15
KLHDC8A	4.50	4.76	4.05
LINC00330	4.50	5.25	3.82
SH2D7	4.50	5.15	4.04
SLC12A9	4.50	4.81	4.17
ACSS1	4.50	4.97	4.15
ATG4A	4.50	4.79	3.69
HLA-DRB1	4.50	8.23	1.97
NME6	4.50	4.83	4.20

GLS2	4.50	4.87	4.16
PLCL1	4.50	5.13	3.71
TRMT12	4.50	4.76	3.91
NRGN	4.50	4.90	3.97
NPPC	4.50	4.92	4.09
OTTHUMG00000176979	4.50	4.76	4.29
HNRNPKP3	4.50	4.83	4.06
OTTHUMG00000004188	4.50	5.08	4.10
GP5	4.50	4.83	4.25
SEMA6B	4.50	4.85	4.20
LOC100506858	4.50	5.00	4.02
IGLV7-43	4.50	4.78	4.29
HBM	4.50	4.83	3.97
FAM159A	4.50	4.93	4.17
TMEM129	4.50	4.79	4.06
FLG	4.50	5.10	4.21
RNF112	4.50	4.75	4.26
LOC100996624	4.50	5.87	3.85
RNF216-IT1	4.50	5.04	4.18
RYR1	4.50	4.87	4.20
ZNF519	4.50	5.08	3.82
NEIL2	4.50	4.99	4.04
RFPL1S	4.50	5.13	3.76
OTTHUMG00000057525	4.50	4.94	4.19
WHAMMP2	4.50	4.92	3.88
TANGO2	4.50	4.93	4.20
RNF216P1	4.50	5.11	3.93
KCNQ5	4.50	5.21	3.69
LOC100996665	4.50	4.81	4.28
HENMT1	4.50	4.77	3.94
SFRP5	4.50	4.71	4.17
SNORD114-26	4.50	5.56	3.25
RPTN	4.50	5.14	4.21
TFEB	4.50	4.97	4.03
MIR3162	4.50	5.14	3.50
LOC286184	4.50	4.78	4.16
BTN2A3P	4.50	4.82	3.98
FES	4.50	4.94	4.03
AGAP2-AS1	4.50	5.30	4.01
DHH	4.50	5.01	4.14
SGSM3	4.50	4.85	4.13
ATP1A2	4.50	4.89	4.17
NKRF	4.50	4.83	4.19
KIRREL3-AS3	4.50	4.84	4.10
PDZD8	4.50	5.08	3.95
TRBV5-7	4.50	4.82	4.01
TIGIT	4.50	4.84	3.70
C19orf54	4.50	4.87	4.11
OTTHUMG00000154157	4.50	4.98	3.88

PNMT	4.50	4.73	4.26
SHC2	4.50	4.91	3.54
FLJ14082	4.50	5.10	4.00
BACH1-IT2	4.50	5.04	3.98
CLEC10A	4.50	4.96	4.02
TMPRSS3	4.50	4.84	4.05
KY	4.49	4.81	4.20
RHPN1	4.49	4.89	4.01
FANCI	4.49	5.07	3.80
MMP28	4.49	4.93	4.20
CYP2D7P1	4.49	5.45	3.88
OMG	4.49	5.30	3.93
MIR4532	4.49	5.48	4.05
OTTHUMG00000152531	4.49	5.64	3.79
GPR114	4.49	4.74	4.23
CCBL1	4.49	4.62	4.38
HNRNPLL	4.49	5.40	3.99
OR1B1	4.49	4.76	4.29
RTN2	4.49	4.76	4.29
CCDC108	4.49	4.87	4.20
C3orf56	4.49	5.15	3.93
LOC100505964	4.49	5.19	4.06
OTTHUMG00000172091	4.49	5.33	3.82
LOC146880	4.49	5.49	3.81
PGLYRP1	4.49	4.83	4.24
OTTHUMG00000183152	4.49	5.33	3.82
FAM189A1	4.49	4.77	4.24
METTL20	4.49	4.85	4.21
OTTHUMG00000162135	4.49	5.14	4.00
FAM118A	4.49	4.79	4.06
IFNLR1	4.49	5.01	4.03
OTTHUMG00000176973	4.49	4.86	4.24
CD5L	4.49	5.07	3.88
RPL23AP7	4.49	5.29	4.10
GUCA1B	4.49	5.11	4.07
MAPK8IP2	4.49	4.70	3.99
OTTHUMG00000037519	4.49	4.91	4.08
DQX1	4.49	4.82	4.08
IGKV2-29	4.49	5.08	4.19
PPP1R14D	4.49	5.19	4.07
PES1	4.49	4.84	3.64
OTTHUMG00000180894	4.49	4.91	4.29
OTTHUMG00000171281	4.49	4.97	4.05
UGGT2-IT1	4.49	5.22	3.90
CAPN13	4.49	4.85	3.88
LOC100505978	4.49	4.91	3.94
LOC100287497	4.49	4.96	4.19
ZNF853	4.49	5.01	4.19
OTTHUMG00000017648	4.49	4.97	4.08

IQGAP2	4.49	5.12	3.81
PRH2	4.49	4.97	3.94
OR51S1	4.49	5.24	4.21
OTTHUMG00000172196	4.49	4.86	4.21
CD300LF	4.49	4.72	4.02
RNA5SP289	4.49	5.28	3.50
B3GNT7	4.49	4.78	4.20
LOC729658	4.49	4.88	4.07
FOXM1	4.49	4.87	3.95
MIR135B	4.49	4.91	4.25
KRTAP10-1	4.49	4.78	3.83
MAML3	4.49	4.96	4.06
CTBP1-AS2	4.49	4.87	4.13
MGC39545	4.49	4.92	3.87
LIPT2	4.49	4.83	4.24
BCDIN3D-AS1	4.49	4.88	3.86
ISLR2	4.49	4.94	4.08
POLE4	4.49	5.20	4.08
RFC2	4.49	4.77	3.95
LOC729987	4.49	4.85	4.17
KIAA1522	4.49	4.90	4.27
OR2L13	4.49	5.18	4.05
PLAC4	4.49	4.90	4.15
POLR3G	4.49	4.83	4.08
C4orf50	4.49	5.05	3.94
TRIM35	4.49	4.61	4.35
N4BP3	4.49	5.41	3.84
LINC00925	4.49	4.75	4.17
PP13439	4.49	4.80	4.24
MFSD1	4.49	4.92	4.04
PPP1R2	4.49	4.79	3.91
GGNBP1	4.49	5.15	3.97
CYP11B2	4.49	5.21	3.69
MIR509-1	4.49	5.74	3.97
MOGAT3	4.49	4.70	4.30
RBMY1D	4.49	5.63	3.29
APOBEC3D	4.49	5.07	3.85
SMYD5	4.49	4.79	4.07
CDHR5	4.49	4.71	4.28
LOC283693	4.49	5.18	3.91
SNHG1	4.49	4.80	3.90
EIF5A2	4.49	5.20	3.58
OXGR1	4.49	5.13	4.10
PRAMEF17	4.49	4.96	3.95
MLC1	4.49	4.79	4.11
LOC729995	4.48	5.09	3.97
PRTG	4.48	4.84	4.01
PTX3	4.48	5.30	3.96
OR2M4	4.48	5.22	4.16

GAL3ST1	4.48	4.79	4.11
OTTHUMG00000010584	4.48	4.83	4.15
KRTAP12-2	4.48	4.76	4.15
SNORA64	4.48	4.98	3.88
NPTX1	4.48	4.94	4.21
LINC00475	4.48	5.43	3.87
LOC286059	4.48	4.84	4.09
SYN3	4.48	4.95	3.73
TSPY26P	4.48	5.00	4.17
FAM151B	4.48	5.03	3.89
CD209	4.48	5.19	3.54
OPN5	4.48	4.82	3.97
OTTHUMG00000151090	4.48	5.11	3.55
AFMID	4.48	4.79	4.12
CTSL2	4.48	4.81	4.22
SIK1	4.48	4.87	4.06
FUK	4.48	4.86	4.25
RAD51-AS1	4.48	5.08	3.80
LINC00900	4.48	5.16	3.86
LOC100240734	4.48	5.52	3.57
LOC643723	4.48	4.69	4.24
DIO3	4.48	5.15	4.08
CSNK1A1L	4.48	5.02	3.93
ZNF32-AS2	4.48	4.68	4.25
NKIRAS1	4.48	4.87	4.10
AMN	4.48	4.77	4.14
EVI5L	4.48	4.78	4.26
ADAMTS3	4.48	4.87	4.03
LOC100507166	4.48	4.83	4.14
NDNL2	4.48	4.99	3.80
LOC284395	4.48	4.90	4.07
OTTHUMG00000181998	4.48	4.85	3.99
AK9	4.48	4.79	4.16
BSND	4.48	4.80	4.05
LOC442028	4.48	5.06	4.04
STX18-IT1	4.48	4.81	4.05
POLB	4.48	4.80	4.10
FAM162B	4.48	4.96	3.96
OTTHUMG00000164822	4.48	4.88	4.18
TSGA13	4.48	4.72	4.15
ZNF883	4.48	4.76	4.01
C22orf34	4.48	4.93	4.15
S1PR4	4.48	5.01	3.87
ODF3	4.48	5.06	3.76
PHOX2A	4.48	4.72	4.26
ALMS1P	4.48	5.30	4.12
ITPR1-AS1	4.48	4.95	4.00
RPP38	4.48	4.79	4.25
PIGW	4.48	4.72	4.13

LOC100505812	4.48	5.15	3.76
OTTHUMG00000003682	4.48	4.92	3.72
APOBEC3H	4.48	4.87	3.78
SHD	4.48	4.82	4.15
EMX2OS	4.48	4.91	4.08
AVPR2	4.48	4.64	4.33
A1BG-AS1	4.48	4.98	4.02
BACH1-IT3	4.48	4.81	4.13
ELP6	4.48	4.73	4.04
LOC100506609	4.48	4.91	4.26
OR2AJ1	4.48	5.20	3.77
OTTHUMG00000161161	4.48	5.02	3.91
LOC100652739	4.48	4.78	4.17
MIR3187	4.48	5.07	4.00
APEX2	4.48	4.92	4.21
RIC8B	4.48	4.82	4.25
DAGLA	4.48	4.80	4.17
TAF6	4.48	4.80	3.87
MAMSTR	4.48	4.79	4.07
AOAH	4.48	4.88	4.04
SKP1P2	4.48	5.07	3.99
PRKX-AS1	4.48	4.77	3.91
CLSTN2	4.48	5.03	4.10
C6orf195	4.47	4.71	4.16
TRIM10	4.47	4.69	4.12
LINC00514	4.47	4.75	4.17
FOXL1	4.47	5.05	3.77
MIR648	4.47	5.00	3.96
LINC00685	4.47	5.01	3.60
LOC400499	4.47	5.08	4.25
MIR600HG	4.47	4.69	4.15
C8orf69	4.47	4.87	4.14
OTTHUMG00000169714	4.47	4.97	4.11
FTSJD1	4.47	4.69	4.04
TRIM69	4.47	4.79	4.21
B3GNT4	4.47	5.02	4.02
MUC2	4.47	4.88	4.20
USP17L23	4.47	4.87	4.12
NEU3	4.47	5.04	4.17
LTK	4.47	4.75	3.92
PRPH2	4.47	4.87	3.89
OTTHUMG00000172123	4.47	4.94	3.94
OR7G1	4.47	5.27	3.28
PWRN1	4.47	5.24	3.66
LDB3	4.47	4.83	4.13
OTTHUMG00000153107	4.47	4.64	4.10
PDE9A	4.47	5.09	3.87
PRSS45	4.47	5.01	4.23
CACNG8	4.47	4.91	3.95

OTTHUMG00000021161	4.47	4.75	4.14
SCARNA8	4.47	5.15	3.32
MIR4710	4.47	5.18	3.74
LOC100506459	4.47	5.10	4.13
GAN	4.47	4.88	3.71
CCDC114	4.47	4.79	4.01
BRCA2	4.47	4.92	3.97
SNORD91A	4.47	5.01	3.95
PALM3	4.47	5.22	4.08
ASB18	4.47	4.90	4.02
POPDC2	4.47	5.04	4.11
MBD3	4.47	4.72	3.87
SGOL2	4.47	4.99	4.12
C11orf92	4.47	5.41	3.66
FLJ30403	4.47	4.90	4.15
OTTHUMG00000151764	4.47	5.34	3.86
EFCAB6	4.47	4.73	4.12
ADPRHL2	4.47	4.88	4.10
OTTHUMG00000171100	4.47	5.13	3.71
MS4A15	4.47	4.80	4.08
B4GALNT2	4.47	4.96	4.04
KLHL25	4.47	4.97	4.22
KIF24	4.47	4.95	3.91
CREM	4.47	4.96	4.10
LOC644656	4.47	4.86	4.23
HSD11B1L	4.47	4.68	4.24
HHEX	4.47	4.90	3.92
ARHGAP11A	4.47	5.29	3.23
HAS2-AS1	4.47	4.99	4.14
OTTHUMG00000163673	4.47	4.87	4.17
BHLHE22	4.47	4.86	4.19
RAB3B	4.47	4.87	4.08
LOC729173	4.47	5.07	4.19
UTP15	4.47	4.95	4.08
FBXO31	4.47	4.77	4.16
OTTHUMG00000168693	4.47	4.84	4.00
CRHR2	4.47	5.05	4.08
GPA33	4.47	5.12	4.00
GHSR	4.47	4.88	3.95
HEXDC-IT1	4.47	5.26	3.72
SFR1	4.47	4.71	3.98
DAPK2	4.47	5.01	3.82
OTTHUMG00000170315	4.47	4.75	4.17
LOC100505504	4.47	4.85	4.04
OTTHUMG00000017549	4.47	4.84	3.85
ABCG8	4.47	4.96	3.67
CCNYL3	4.47	4.84	4.13
MIR4675	4.47	4.70	4.01
POTEF	4.47	5.19	4.21

GDF6	4.47	5.13	3.79
OTTHUMG00000017570	4.47	4.92	3.94
LPL	4.47	5.20	3.80
MSRA	4.47	4.83	4.10
FREM2-AS1	4.46	5.17	3.88
SOWAHC	4.46	4.89	4.13
LINC00544	4.46	4.77	4.19
SOX17	4.46	5.35	4.03
MTHFR	4.46	4.77	4.10
PAGE5	4.46	5.61	3.34
AKR7A3	4.46	4.87	4.10
GCLC	4.46	4.80	4.15
SACS-AS1	4.46	4.81	4.14
TFCP2L1	4.46	5.14	3.95
UBE3D	4.46	4.83	4.06
LOC100527964	4.46	4.73	4.10
GPR152	4.46	5.08	3.96
GUCY2D	4.46	4.92	4.04
LAX1	4.46	4.84	4.00
CHDH	4.46	5.10	3.60
OTTHUMG00000019201	4.46	5.21	3.88
SLC16A8	4.46	4.90	4.03
CCRL1	4.46	4.95	4.19
RHO	4.46	4.81	4.07
NUP54	4.46	4.89	3.70
ALS2CL	4.46	4.90	4.07
KCNJ6	4.46	4.91	3.97
ASIP	4.46	5.07	3.58
IGFL1	4.46	4.98	4.00
LIPT1	4.46	5.13	3.95
OTTHUMG00000156285	4.46	4.78	4.20
MYH7	4.46	4.68	4.22
C20orf195	4.46	4.77	4.12
MED14-AS1	4.46	4.95	3.85
DNAJC3-AS1	4.46	5.05	3.97
OTTHUMG00000166037	4.46	4.94	3.89
DKFZp434J0226	4.46	4.66	4.13
CASR	4.46	4.91	4.22
KRTAP5-4	4.46	5.23	3.64
RNA5SP366	4.46	5.05	3.65
PDZRN3-AS1	4.46	4.71	4.09
BCL7C	4.46	4.74	4.22
OTTHUMG00000021064	4.46	4.83	4.04
LOC100506458	4.46	5.19	3.91
F3	4.46	6.12	3.43
BTBD3	4.46	4.69	3.97
OTTHUMG00000153019	4.46	4.87	4.09
OTTHUMG00000168461	4.46	5.12	3.84
PAX5	4.46	4.64	4.22

LINC00886	4.46	5.27	3.84
BHLHB9	4.46	4.93	3.93
MIR4660	4.46	4.94	4.03
OR10G8	4.46	5.28	3.85
TMEM133	4.46	4.89	3.75
RSPH9	4.46	4.95	4.16
FIZ1	4.46	4.98	4.14
MIR4302	4.46	4.98	3.21
OTTHUMG00000015768	4.46	5.05	3.65
CIDEC	4.46	5.67	3.77
CWF19L1	4.46	4.90	3.94
KCNMB4	4.46	4.78	4.06
NAGPA	4.46	4.87	3.84
RCSD1	4.46	4.83	3.86
IGLJ5	4.46	5.28	3.86
NUF2	4.46	4.91	4.04
LOC100506641	4.46	4.97	4.12
PTGDR	4.46	5.26	3.81
DDX60	4.46	5.27	3.82
RASSF5	4.46	4.86	3.90
ADCY5	4.46	4.87	4.21
OTTHUMG00000017599	4.46	5.15	3.95
FAM83C	4.46	5.27	3.93
LRRC73	4.46	4.98	4.19
GPR83	4.46	5.02	3.85
GUCA1A	4.46	4.93	3.80
HOXA3	4.46	4.69	4.22
MVK	4.45	4.89	3.81
CDK2AP2	4.45	4.92	3.90
LOC389199	4.45	4.68	4.13
HN1	4.45	5.19	3.86
MIR377	4.45	5.38	3.25
MAPT-IT1	4.45	5.51	4.01
TOX3	4.45	4.79	4.00
ALOX15P1	4.45	5.26	3.75
CHD5	4.45	4.71	4.00
TTTY16	4.45	5.07	3.84
LOC550112	4.45	4.83	4.21
JAK3	4.45	4.86	3.62
LOC100128770	4.45	4.83	4.06
WNT7B	4.45	4.83	4.19
LINC00324	4.45	4.83	4.20
OTTHUMG00000150946	4.45	5.13	4.06
ABCA9	4.45	4.99	3.37
DKFZp451A211	4.45	5.42	3.91
IQCE	4.45	4.85	4.11
SASH3	4.45	4.97	3.99
RDH12	4.45	5.16	3.98
MCM8	4.45	4.92	3.90

TRAPPC2	4.45	5.33	3.98
GREM2	4.45	4.79	4.07
MYO1G	4.45	4.91	4.11
AGAP1-IT1	4.45	4.77	3.93
OTTHUMG00000020646	4.45	4.75	3.85
RNF32	4.45	4.75	4.13
SPC25	4.45	5.01	4.23
LOC400043	4.45	4.78	4.15
MGC12916	4.45	5.23	3.42
ARG1	4.45	4.72	4.16
LMO3	4.45	5.21	3.91
LOC90246	4.45	5.12	3.95
IL1R2	4.45	5.09	4.10
SLC10A1	4.45	4.86	4.10
LRRC39	4.45	4.80	4.11
NTN3	4.45	4.81	4.01
RBMY1B	4.45	5.59	3.32
ITPRIPL1	4.45	5.03	3.95
OTTHUMG00000020189	4.45	4.80	4.01
ELL	4.45	5.13	3.97
SCN10A	4.45	4.97	3.75
USP12-AS2	4.45	5.11	3.69
CHD7	4.45	4.69	4.24
KCNH4	4.45	4.83	3.84
NUSAP1	4.45	4.95	4.09
PTPRN2	4.45	4.82	4.03
PEX10	4.45	4.79	4.19
LOC339529	4.45	4.81	4.13
FAM218A	4.45	4.88	4.05
COQ5	4.45	4.87	4.23
PCDHB7	4.45	5.09	3.78
KIAA1614	4.45	5.02	3.70
GRID1-AS1	4.45	4.97	3.95
CHAC1	4.45	4.87	4.08
UQCRB	4.45	4.63	3.94
MAPRE3	4.45	4.73	4.20
TBC1D16	4.45	4.87	3.79
OR51G1	4.45	5.26	3.98
LOC728752	4.45	4.96	3.88
FAM167A	4.45	4.67	4.30
C20orf166	4.45	4.76	4.15
FLJ45248	4.45	4.99	4.02
TAGAP	4.45	5.33	4.00
OR2T2	4.45	5.13	3.91
MIR23A	4.45	4.77	4.10
CASKIN1	4.45	4.89	4.09
SDHAF1	4.45	4.88	4.03
DAO	4.45	4.87	4.02
MYCBPAP	4.45	5.25	3.96

LOC340073	4.45	4.77	4.25
MS4A18	4.45	5.22	4.02
SYT10	4.45	4.89	3.95
MRS2P2	4.45	5.16	3.59
LINC00282	4.45	4.80	3.87
RPL3L	4.45	4.85	4.17
CTU1	4.44	4.95	4.12
CA8	4.44	4.91	4.09
RNA5SP431	4.44	5.02	3.68
C1QTNF4	4.44	4.74	4.07
MMP1	4.44	7.47	2.94
PMS2CL	4.44	5.08	3.65
SPATA31E1	4.44	4.84	4.20
LOC285540	4.44	5.22	3.62
CCT6B	4.44	4.84	4.07
GPR37L1	4.44	4.74	4.04
GTDC1	4.44	5.06	3.51
C9orf163	4.44	4.64	4.22
CCR1	4.44	5.47	3.89
CXorf38	4.44	4.73	3.97
C15orf53	4.44	5.24	3.95
ZNF630-AS1	4.44	5.08	4.01
RALGPS2	4.44	4.82	4.00
NPR3	4.44	4.80	3.95
OTTHUMG00000032279	4.44	5.04	3.94
OTTHUMG00000059450	4.44	4.90	4.02
RASA3-IT1	4.44	4.68	4.20
RPS6KB2	4.44	4.72	4.19
PIP5KL1	4.44	4.74	4.26
OTTHUMG00000165544	4.44	4.80	4.13
SNORD115-44	4.44	5.56	3.29
OTTHUMG00000015255	4.44	5.39	3.84
ACMSD	4.44	4.91	4.13
PHF6	4.44	5.07	3.72
LOC283575	4.44	4.90	3.92
MPZ	4.44	4.86	4.25
OTTHUMG00000017561	4.44	5.00	4.02
FHAD1	4.44	4.89	4.01
LOC100652824	4.44	4.90	3.95
KCND1	4.44	4.81	3.88
MRM1	4.44	4.98	3.58
LINC00415	4.44	4.81	3.96
PTDSS2	4.44	4.74	3.96
NXPE2	4.44	5.45	3.44
OTTHUMG00000012023	4.44	5.14	3.74
HMX1	4.44	4.85	4.07
MIR563	4.44	4.82	4.07
NMU	4.44	4.90	4.11
EFNA2	4.44	4.88	3.99

SPC24	4.44	4.72	3.94
TBC1D30	4.44	4.81	3.96
ZNF615	4.44	4.87	3.79
KPRP	4.44	5.03	3.87
SPN	4.44	4.85	4.13
AMDHD1	4.44	5.18	3.84
ACPT	4.44	4.79	4.01
MXD3	4.44	4.92	3.83
CECR2	4.44	5.01	4.00
ACVR1C	4.44	4.74	4.18
LINC00593	4.44	4.71	4.15
THBD	4.44	4.64	4.20
KRT18P10	4.44	4.85	3.80
FNDC9	4.44	4.87	3.95
PLA2G4F	4.44	4.94	4.02
PYGO1	4.44	4.70	4.11
RNY4P16	4.44	5.42	3.51
INA	4.44	4.79	4.06
LOC100286922	4.44	5.28	3.93
ANK1	4.44	4.68	4.14
HBQ1	4.44	4.90	3.98
CCR2	4.44	4.84	3.95
TACR2	4.44	4.78	4.03
LOC339807	4.44	5.02	4.02
OTTHUMG00000155618	4.44	5.06	3.61
ARFGAP2	4.44	4.79	3.89
PLAC1	4.44	4.62	4.11
C11orf86	4.44	4.99	4.10
AGMAT	4.44	4.98	3.89
IL2RG	4.44	5.15	3.97
OTTHUMG00000169251	4.44	4.94	4.04
OR10A4	4.44	4.91	3.87
LINC00667	4.44	4.84	3.73
DPY19L2P2	4.44	5.23	3.95
RAX2	4.44	5.37	3.74
OTTHUMG00000172230	4.43	4.77	4.13
LINC00116	4.43	4.78	4.05
OTTHUMG00000173226	4.43	5.19	3.83
SIM2	4.43	4.66	4.17
SRGAP3-AS3	4.43	5.11	3.96
ZNF211	4.43	4.82	3.78
ZSCAN5C	4.43	5.41	3.70
ABHD3	4.43	4.85	4.09
PIK3R2	4.43	4.70	4.06
OTTHUMG00000168342	4.43	4.89	4.13
SUSD1	4.43	4.71	4.05
IGLL1	4.43	5.28	3.72
ZNF296	4.43	4.79	4.10
ZFP37	4.43	4.70	4.02

IFI44L	4.43	5.83	3.79
OTTHUMG00000037268	4.43	4.89	4.11
ATOH8	4.43	4.84	4.02
ALOX15	4.43	5.06	4.19
FRMPD3-AS1	4.43	4.92	3.89
SLC35E4	4.43	4.95	3.92
ERBB3	4.43	4.85	3.98
ANXA9	4.43	4.91	4.08
OTTHUMG00000020434	4.43	5.13	4.07
CLHC1	4.43	5.06	4.04
ADTRP	4.43	4.98	3.86
OTTHUMG00000164709	4.43	5.12	3.79
UBR7	4.43	4.96	3.93
LHX6	4.43	4.72	4.23
PARD6A	4.43	4.69	4.00
OR6K3	4.43	5.07	3.88
IQGAP3	4.43	4.87	4.05
ALG1	4.43	4.95	4.05
PPP3R2	4.43	4.79	4.00
SLIT1	4.43	4.75	4.17
FER1L6-AS1	4.43	4.78	3.93
LOC283587	4.43	4.85	3.90
HOXD-AS2	4.43	4.65	4.17
PSTPIP1	4.43	4.80	4.08
DEFB117	4.43	4.88	3.56
LOC401437	4.43	5.04	3.83
ZNF354B	4.43	4.75	3.84
RNA5SP214	4.43	5.22	3.91
ANKDD1B	4.43	4.95	4.13
FRMPD3	4.43	4.68	4.04
SLC22A14	4.43	4.85	3.92
OSBP2	4.43	4.86	4.12
IKZF4	4.43	4.77	4.16
FRY-AS1	4.43	4.78	3.94
SMPDL3B	4.43	4.75	4.11
OTTHUMG00000039470	4.43	4.79	4.14
OR5D14	4.43	5.17	3.83
LOC728095	4.43	4.77	4.01
LOC100506527	4.43	4.75	4.22
ZNF836	4.43	5.50	3.90
ZNF705D	4.43	4.97	3.92
MYO5C	4.43	5.22	3.31
GGACT	4.43	4.61	4.24
ZNF75D	4.43	4.80	4.04
SYBU	4.43	4.73	3.89
EPHB2	4.43	4.71	3.92
EXTL1	4.43	5.21	3.73
MYCL1	4.43	4.76	4.16
TAL1	4.43	4.82	3.95

EDARADD	4.43	4.84	4.04
SOX11	4.43	4.87	3.84
STRADB	4.43	4.85	3.85
PLEKHG1	4.43	5.05	3.82
OTTHUMG00000165152	4.43	4.79	4.06
OTTHUMG00000173496	4.43	4.75	4.19
OTTHUMG00000148992	4.43	4.94	3.99
C9orf170	4.43	4.83	3.98
LOC440925	4.43	4.96	4.12
C1orf228	4.43	5.05	3.73
LOC100507459	4.43	4.72	4.13
LRP3	4.43	4.70	4.07
PDILT	4.42	5.00	4.15
CLPSL2	4.42	5.08	4.11
C2orf50	4.42	4.78	4.11
TGM1	4.42	4.72	4.07
LOC692247	4.42	4.82	4.10
LOC100128682	4.42	5.07	4.01
CTCFL	4.42	5.07	3.88
STAP2	4.42	5.02	4.04
NDUFAF5	4.42	4.91	3.94
OTTHUMG00000020483	4.42	4.73	4.08
LINC00473	4.42	4.90	3.99
CPNE5	4.42	4.74	3.92
LOC399900	4.42	4.74	4.08
DHX34	4.42	4.83	3.99
ZNF324	4.42	4.97	4.01
OTTHUMG00000018618	4.42	5.22	3.48
SLC26A10	4.42	4.70	3.86
LINC00229	4.42	4.79	4.09
SEL1L3	4.42	5.13	3.99
PANK4	4.42	4.71	4.08
HAUS7	4.42	4.92	4.06
SOGA3	4.42	5.00	3.58
CCRL2	4.42	4.85	4.11
LOC286186	4.42	5.08	4.03
RASL11A	4.42	4.83	3.82
APOPT1	4.42	4.91	3.82
CPVL	4.42	5.23	3.80
MTA3	4.42	4.58	4.21
SLC30A2	4.42	4.89	4.08
SLC2A7	4.42	4.68	4.13
ADH1B	4.42	5.06	3.84
C16orf82	4.42	4.86	3.98
LOC100129195	4.42	4.87	4.13
EXOC3L2	4.42	4.77	4.06
KIAA1024	4.42	4.75	3.95
LOC100996524	4.42	4.70	4.09
OTTHUMG00000021535	4.42	4.88	4.10

ZNF418	4.42	4.80	4.07
LINC00703	4.42	4.83	4.14
DACT1	4.42	5.07	3.85
ZC4H2	4.42	4.86	3.71
C16orf59	4.42	4.77	3.97
PKD1L2	4.42	4.81	3.91
DSTNP2	4.42	5.03	3.70
RNA5SP242	4.42	4.90	4.02
SLC22A1	4.42	4.95	4.00
MIR4310	4.42	5.35	3.62
ICA1	4.42	5.27	3.85
TMEM150B	4.42	4.95	3.96
OTTHUMG00000045406	4.42	4.70	4.23
PLA2G4C	4.42	5.03	3.83
ABCB8	4.42	4.71	4.22
CLCN5	4.42	4.76	3.89
ARHGAP15	4.42	4.77	3.68
MIR31	4.42	5.02	3.91
DNA2	4.42	4.76	4.04
KRT9	4.42	5.07	3.98
C2orf71	4.42	4.80	3.81
LOC284080	4.42	5.17	3.97
E2F8	4.42	4.79	4.09
GDNF	4.42	4.88	4.05
SOX21-AS1	4.42	4.96	4.02
TRIM49B	4.42	5.04	4.06
RNA5SP145	4.42	4.79	3.88
AKAP12	4.42	4.95	3.64
PIK3R6	4.42	4.70	4.12
CATSPER4	4.42	4.96	4.05
ESRP2	4.42	4.83	3.99
SPSB4	4.42	4.81	4.17
OTTHUMG00000172651	4.42	5.14	3.83
PNLIPRP2	4.42	4.91	3.98
KANK1	4.42	4.74	3.99
IDI1	4.42	4.79	4.17
C8orf74	4.42	5.08	3.92
OTTHUMG00000172101	4.42	4.76	4.13
FLG-AS1	4.42	4.81	4.06
SOX1	4.42	4.73	3.93
LHPP	4.42	4.95	3.96
TM4SF5	4.42	4.90	4.07
LINC00028	4.42	4.98	3.88
RAB17	4.42	4.81	3.99
KIAA1875	4.42	4.81	3.90
PPTC7	4.42	4.86	3.81
FAHD2A	4.42	4.87	4.16
BLNK	4.42	5.07	3.86
ADRA2C	4.41	4.79	4.03

OTTHUMG00000152386	4.41	5.42	3.58
RFX4	4.41	4.88	3.97
OTTHUMG00000032472	4.41	5.40	3.89
OTTHUMG00000019252	4.41	4.87	4.03
OTTHUMG00000151735	4.41	4.83	4.03
DNAH10OS	4.41	4.66	4.09
TMEM160	4.41	4.70	3.92
EPPK1	4.41	4.82	3.97
OTTHUMG00000163137	4.41	4.76	4.21
MIDN	4.41	4.64	3.98
A2ML1	4.41	4.90	3.88
FAM78A	4.41	4.68	4.15
MSR1	4.41	5.28	3.56
BEST1	4.41	4.61	4.16
MEI1	4.41	4.81	4.21
UBL7-AS1	4.41	4.88	3.58
MT1JP	4.41	5.10	3.82
CYTH3	4.41	4.78	3.73
HMGB4	4.41	5.05	3.90
RGMB-AS1	4.41	4.92	4.14
POLR2E	4.41	4.77	3.77
TRIB1	4.41	4.67	4.07
UGT8	4.41	4.81	4.13
MTRNR2L10	4.41	5.05	3.76
FAM156A	4.41	4.99	3.80
RTDR1	4.41	4.66	4.16
KLHL34	4.41	4.83	3.61
BCORL1	4.41	4.81	3.93
LYAR	4.41	4.95	3.65
WASF1	4.41	4.64	4.07
CYP4F24P	4.41	5.50	3.46
PLEKHG6	4.41	4.64	4.16
BRCA1	4.41	4.79	3.93
MIR3926-1	4.41	4.91	3.82
HOXB1	4.41	4.89	4.14
CASC5	4.41	5.16	3.80
NYX	4.41	4.86	4.00
RASD2	4.41	4.71	4.00
UNC93A	4.41	4.84	4.08
IKZF1	4.41	4.77	4.19
CDK11A	4.41	4.82	3.97
TRAPPC13	4.41	4.77	3.86
SERPINA13P	4.41	5.09	3.88
DNMT3B	4.41	4.69	3.86
SCT	4.41	4.76	3.99
SEMA3B	4.41	4.73	4.14
KBTBD3	4.41	4.54	4.07
CLVS2	4.41	5.27	3.86
HYKK	4.41	4.78	4.01

GIMAP8	4.41	4.73	3.82
SHROOM3	4.41	4.66	3.95
ZP3	4.41	4.99	3.96
KCNJ2	4.41	4.96	3.80
PI16	4.41	4.94	4.07
C1orf233	4.41	4.74	3.80
SNORA9	4.41	4.99	3.34
LOC284412	4.41	5.03	3.79
MIR4311	4.41	5.18	3.39
NAT9	4.41	4.74	3.96
FAM154A	4.41	4.80	4.07
IGF2BP2	4.41	4.72	4.01
LARS2	4.41	4.97	4.00
CCDC110	4.41	5.00	3.71
FLJ32790	4.41	4.97	4.01
MIR4771-1	4.41	4.89	3.45
C5orf30	4.41	4.92	3.92
NHSL1	4.41	4.75	3.78
ENTPD1	4.41	5.44	3.86
TMEM191C	4.41	4.82	3.47
AWAT2	4.41	4.82	3.99
ADAMTS8	4.41	4.85	4.00
CORO2B	4.41	4.94	3.92
OTTHUMG00000015860	4.41	4.69	3.97
C10orf85	4.41	4.81	4.15
LOC285768	4.41	5.05	3.83
RSPH1	4.41	4.79	3.86
SHANK1	4.41	4.88	3.90
ABCA10	4.40	5.01	3.75
ZNF22	4.40	5.09	3.94
FLJ20712	4.40	4.82	3.94
RASSF1	4.40	4.92	4.15
OTTHUMG00000022687	4.40	4.73	3.87
OTTHUMG00000017546	4.40	5.05	3.97
OTTHUMG00000171820	4.40	4.75	4.17
OTTHUMG00000170067	4.40	5.32	3.59
CSNK1A1P1	4.40	4.94	3.95
OTTHUMG00000151365	4.40	4.88	3.85
LOC100240728	4.40	4.98	3.97
OOEP-AS1	4.40	4.87	3.73
OR6C3	4.40	5.33	3.94
AP1B1P1	4.40	4.94	4.16
TMEM192	4.40	4.75	3.97
APLF	4.40	4.52	4.17
LOC100128946	4.40	4.85	3.88
SLC34A3	4.40	5.14	3.36
GATA2	4.40	4.80	3.96
FGF5	4.40	4.63	3.86
CYS1	4.40	4.79	3.54

CNTD2	4.40	4.75	3.84
HES6	4.40	4.61	4.09
MIIP	4.40	4.82	4.01
MGC15705	4.40	4.84	3.72
SERTAD1	4.40	4.73	4.13
LOC374890	4.40	5.28	3.62
TMEM213	4.40	5.03	4.01
FERMT1	4.40	4.70	4.10
DUT	4.40	4.73	3.99
FLJ26245	4.40	4.75	3.96
LOC285556	4.40	4.86	4.18
LOC100129473	4.40	4.82	3.84
TLR1	4.40	5.12	3.93
WFDC12	4.40	4.78	3.95
CRYGA	4.40	4.87	3.79
HTR3E	4.40	5.17	3.72
S100A12	4.40	5.32	3.82
CYP4B1	4.40	5.16	3.98
USHBP1	4.40	4.75	3.99
EDN1	4.40	5.08	3.57
SPRNP1	4.40	5.48	3.07
MED12L	4.40	4.68	4.07
TECPR1	4.40	4.76	4.25
AHSG	4.40	4.71	3.94
LIME1	4.40	5.05	3.88
TYRO3	4.40	4.66	4.16
LOC284837	4.40	4.86	3.76
NEURL2	4.40	4.76	3.75
MEGF11	4.40	4.78	4.15
CNGB3	4.40	4.60	4.16
LOC100506022	4.40	4.70	4.12
CDHR2	4.40	4.64	3.92
SNX10	4.40	5.56	3.90
C2orf70	4.40	4.66	4.06
PTGER4	4.40	4.98	3.85
SRMS	4.40	4.58	4.01
CD40	4.40	5.29	4.09
MIR133B	4.40	4.99	3.99
TCEB3CL2	4.40	4.92	3.98
C20orf203	4.40	4.62	4.01
ZBTB32	4.40	4.79	3.80
UGT2B10	4.40	4.72	4.05
RAB41	4.40	4.74	3.79
LOC100506054	4.40	5.00	4.02
PELI2	4.40	5.30	3.49
SLC12A3	4.40	4.64	4.02
OTTHUMG00000034822	4.40	4.92	3.95
LOC388948	4.40	4.71	3.76
C3orf20	4.40	5.02	4.08

RNF219-AS1	4.40	4.66	4.21
RNASE8	4.40	5.16	3.82
MIR659	4.40	5.11	3.51
MIR765	4.40	4.95	3.84
GPM6A	4.40	4.87	3.88
IMPG2	4.40	4.68	4.23
C9orf96	4.40	4.77	4.17
LCK	4.40	4.75	3.96
OR4D11	4.40	5.23	4.00
C7orf66	4.40	5.34	3.68
CITED1	4.40	4.65	4.06
IGFL2	4.40	4.75	3.71
USF1	4.40	4.77	3.84
USP30-AS1	4.40	4.71	4.05
ZSCAN23	4.40	4.93	3.88
OTTHUMG00000179318	4.40	5.09	3.57
C20orf196	4.40	4.73	4.17
CDHR4	4.40	4.85	3.62
THEM5	4.40	5.06	4.06
LOC643201	4.39	4.92	4.04
GABRE	4.39	4.96	3.39
KNDC1	4.39	4.72	4.04
NELL1	4.39	4.88	4.11
QSOX2	4.39	4.80	3.93
MYBPC3	4.39	4.68	3.84
ATP13A2	4.39	4.92	3.74
IL6	4.39	4.77	4.00
OTTHUMG00000153448	4.39	4.91	3.98
OR12D2	4.39	5.10	3.80
DOCK3	4.39	4.75	3.98
ARHGEF10L	4.39	4.71	4.14
KCNJ8	4.39	4.93	3.67
S1PR5	4.39	4.64	3.82
ZFP69B	4.39	4.93	3.85
OTTHUMG00000180012	4.39	4.92	3.93
ADPRM	4.39	4.80	4.03
IGLV9-49	4.39	5.17	3.88
LOC730020	4.39	5.06	3.82
OTTHUMG00000164978	4.39	5.02	4.03
OTTHUMG00000150319	4.39	5.03	3.79
OTTHUMG00000152579	4.39	4.81	4.15
LINC00426	4.39	4.78	4.18
PIGX	4.39	4.69	4.04
RASL12	4.39	5.20	4.01
OTTHUMG00000172237	4.39	4.67	4.10
DUSP8	4.39	4.88	4.24
ZNF263	4.39	4.86	3.93
NEIL3	4.39	4.91	3.81
MPPED1	4.39	4.99	3.65

OTOG	4.39	4.63	4.24
ITPKA	4.39	4.81	4.01
LINC00487	4.39	4.80	3.93
NCAM1	4.39	4.83	3.92
ZNF454	4.39	5.03	4.00
KRT31	4.39	4.85	3.93
RGS12	4.39	4.68	4.02
LOC100507388	4.39	4.67	3.86
INCENP	4.39	4.46	4.21
LRRC70	4.39	4.78	4.08
UBXN8	4.39	4.78	4.11
PMCHL1	4.39	5.59	3.12
MIR2681	4.39	4.92	3.96
MIR3674	4.39	4.66	4.09
DNAJC18	4.39	4.78	3.96
SOX30	4.39	5.21	3.97
REEP2	4.39	4.72	4.04
MIR4299	4.39	5.19	3.88
LRRC10B	4.39	4.77	3.88
ITGAE	4.39	4.67	4.17
NLRC3	4.39	4.67	4.07
TTC39A	4.39	4.68	4.09
SLMO1	4.39	5.08	3.92
OTTHUMG00000182000	4.39	4.93	4.03
LOC440742	4.39	4.80	4.05
PRKAR2B	4.39	5.33	3.46
C2orf69	4.39	4.91	3.80
OTTHUMG00000177951	4.39	4.71	3.88
ZBED3-AS1	4.39	4.93	3.97
TRIB3	4.39	4.86	4.09
LOC100506325	4.39	4.81	3.98
HIST1H2AI	4.39	5.19	3.56
TOP1P2	4.39	4.89	3.91
SORCS2	4.39	4.68	3.98
LOC147093	4.39	4.98	4.04
PROX2	4.39	4.94	3.96
RGAG4	4.39	4.88	3.92
ADIPOQ	4.39	6.48	3.33
MYLK2	4.39	4.78	4.10
LOC100289230	4.39	4.78	3.88
SP140L	4.38	5.09	3.89
ITIH4-AS1	4.38	4.97	3.76
GPANK1	4.38	4.62	4.20
ANKRD61	4.38	4.85	4.15
OR2H2	4.38	5.00	3.83
LOC387720	4.38	4.67	4.15
OTTHUMG00000152794	4.38	4.61	3.82
NAPSB	4.38	4.73	4.15
LOC100130417	4.38	4.85	3.90

CIB2	4.38	4.76	3.95
OTTHUMG00000176355	4.38	5.48	3.91
BUB1	4.38	5.03	3.90
LOC643355	4.38	4.82	3.90
LOC100133106	4.38	4.85	4.06
LOC285500	4.38	4.97	3.70
LOC100128276	4.38	4.59	4.23
LOC643387	4.38	4.68	4.11
MAFG	4.38	4.81	3.85
DEFB106A	4.38	4.94	4.08
MMS22L	4.38	4.85	3.96
LOC100289361	4.38	4.80	3.53
MOB3B	4.38	5.02	3.04
MICU3	4.38	4.97	4.04
RAB25	4.38	5.19	3.49
ZSWIM5	4.38	5.18	3.97
CCDC151	4.38	4.71	3.93
TCEANC	4.38	4.76	3.63
FASLG	4.38	5.01	3.99
KLKP1	4.38	4.81	3.86
OTTHUMG00000016716	4.38	4.91	3.81
CXCR6	4.38	4.85	4.08
KCNAB2	4.38	4.75	4.02
LOC283914	4.38	4.78	4.00
SYT9	4.38	4.85	3.90
LOC100289283	4.38	5.12	3.85
DMBT1	4.38	4.90	3.85
OR7C1	4.38	5.54	3.79
LOC440173	4.38	5.20	3.78
ZNF530	4.38	4.67	3.32
EYA1	4.38	4.77	3.69
OTTHUMG00000171046	4.38	4.66	4.06
FER1L5	4.38	4.63	4.17
SNX29P1	4.38	4.98	3.97
KRT39	4.38	4.82	4.03
TMEM81	4.38	4.74	3.81
ECEL1P2	4.38	5.04	3.92
KRT8P41	4.38	5.13	3.68
SIRPB2	4.38	4.82	3.87
LOC100507191	4.38	5.18	4.09
ALCAM	4.38	4.79	4.03
SYCE2	4.38	4.59	4.11
HCG23	4.38	4.78	4.15
LOC100506907	4.38	4.82	3.99
LOC100129973	4.38	4.76	3.87
LINC00917	4.38	4.59	4.06
AP5B1	4.38	4.55	4.10
SNORA66	4.38	4.95	2.80
KLHL31	4.38	4.96	3.80

MIR4283-1	4.38	4.96	3.90
CCDC137	4.38	4.80	4.07
PRSS33	4.38	4.95	3.87
ADARB2	4.38	4.57	3.98
PPM1H	4.38	4.89	3.77
PAX1	4.38	4.62	4.12
OTTHUMG00000153205	4.38	4.88	3.97
SLC12A8	4.38	4.83	3.30
OTTHUMG00000163336	4.38	4.76	4.15
SOCS2	4.38	4.80	3.96
FANCF	4.38	4.77	3.88
LINC00200	4.38	4.65	4.16
ADAM23	4.38	4.94	3.86
RNU7-50P	4.38	5.34	3.24
SERPINB9	4.38	5.08	3.87
SSTR5-AS1	4.38	4.95	3.79
RNA5SP206	4.38	5.60	3.65
C10orf91	4.38	4.81	4.14
ADAM22	4.38	4.76	3.79
OTTHUMG00000153742	4.38	4.97	4.01
RNU1-22P	4.38	4.99	3.67
FLJ45825	4.38	4.85	3.98
TMC8	4.38	4.76	3.96
FLJ11292	4.38	4.63	3.94
RPL19P12	4.38	4.91	3.84
ATOH1	4.38	4.85	3.75
RNA5SP437	4.37	4.93	3.93
CLLU1	4.37	4.65	4.16
GJB5	4.37	5.12	3.99
ALDH16A1	4.37	4.77	4.15
RASL11B	4.37	4.75	4.24
EVX2	4.37	4.79	4.02
LOC285484	4.37	4.83	4.02
OTTHUMG00000161631	4.37	4.82	3.85
SLC22A20	4.37	4.62	4.03
BIRC3	4.37	4.90	3.83
OCSTAMP	4.37	4.78	4.02
LOC728342	4.37	4.56	4.22
FANCM	4.37	4.74	4.03
OTTHUMG00000182192	4.37	5.26	3.58
OTTHUMG00000132217	4.37	5.14	3.46
CCDC157	4.37	4.74	4.01
CRP	4.37	5.03	3.94
DTX1	4.37	4.66	3.74
MBL1P	4.37	4.80	3.98
DUS4L	4.37	4.72	4.17
CDC34	4.37	4.81	3.97
ATRIP	4.37	4.90	3.72
RASGRP1	4.37	4.61	4.02

GP2	4.37	4.74	4.08
MIR4635	4.37	4.81	3.57
PABPC5	4.37	4.77	4.03
ARL16	4.37	4.75	3.84
SLC24A3	4.37	4.80	3.66
HDHD1	4.37	4.71	3.92
NTF3	4.37	4.78	3.55
PC	4.37	4.75	3.97
DKFZP434K028	4.37	4.82	3.86
HPDL	4.37	4.76	4.19
WBSCR28	4.37	4.76	4.01
TNFSF15	4.37	4.69	3.60
KHK	4.37	4.91	3.93
COL18A1-AS1	4.37	4.83	4.08
PLCH1-AS1	4.37	4.76	3.84
OTTHUMG00000156393	4.37	4.80	3.53
OTTHUMG00000178589	4.37	4.87	4.04
OTTHUMG00000171068	4.37	4.88	3.76
TULP2	4.37	4.96	3.84
CCDC15	4.37	4.55	3.93
ADPRH	4.37	4.83	3.83
MYH3	4.37	4.88	3.87
OTOF	4.37	4.84	3.98
CACNB4	4.37	4.83	3.99
HELT	4.37	4.67	4.10
LOC400768	4.37	4.63	4.20
RNA5SP499	4.37	5.16	3.13
LOC101060142	4.37	4.91	3.94
PISRT1	4.37	4.77	4.04
KAT7	4.37	4.77	3.69
FCRL1	4.37	4.65	4.04
OTTHUMG00000155528	4.37	4.67	3.61
AHSP	4.37	4.90	3.93
CBX4	4.37	4.68	4.01
APOA4	4.37	4.78	3.76
OR1J1	4.37	4.90	3.81
FAM172BP	4.36	5.05	3.61
LOC100289187	4.36	4.91	3.64
LOC1720	4.36	5.06	3.55
LINC00951	4.36	4.75	3.98
MORC1-AS1	4.36	4.74	4.19
E2F2	4.36	4.74	3.89
LOC100505702	4.36	4.72	3.91
RAB39B	4.36	4.93	3.84
ANO2	4.36	5.15	4.08
OTTHUMG00000150683	4.36	5.36	3.61
PCYT2	4.36	4.71	4.14
XK	4.36	4.75	3.79
LMOD2	4.36	4.74	3.98

AARS2	4.36	4.59	4.18
WDFY4	4.36	4.81	4.06
OTTHUMG00000165872	4.36	4.84	3.90
B3GNT8	4.36	4.88	3.55
HEATR4	4.36	4.71	4.15
UPP1	4.36	4.89	3.69
SLC44A3	4.36	4.58	3.99
CALHM1	4.36	4.97	3.72
LOC100129203	4.36	4.73	3.80
LOC100270679	4.36	4.71	4.05
PKD2L1	4.36	4.64	4.17
GNGT2	4.36	4.92	3.77
OTTHUMG00000133715	4.36	4.83	3.76
FTCD	4.36	4.54	4.14
PAQR5	4.36	4.85	4.03
FBXO36-IT1	4.36	4.87	3.79
OTTHUMG00000035458	4.36	5.05	3.65
TXNDC2	4.36	4.78	3.96
PSG5	4.36	4.84	4.15
VAV1	4.36	4.74	3.95
CPO	4.36	4.97	3.71
LOC100129617	4.36	4.62	4.01
MATN1	4.36	4.77	3.95
OTTHUMG00000164537	4.36	4.89	4.01
CATSPER2P1	4.36	5.26	2.86
C7orf13	4.36	4.84	4.06
MIR1183	4.36	5.05	3.58
OTTHUMG00000161051	4.36	5.10	3.96
BEND3P3	4.36	4.87	3.67
CD300LD	4.36	5.23	3.72
FAM27L	4.36	4.81	3.70
ING1	4.36	4.81	3.96
SLC10A7	4.36	4.63	4.00
CCBP2	4.36	4.73	4.03
OTTHUMG00000090443	4.36	4.81	4.00
OTTHUMG00000014193	4.36	4.71	3.73
PCDH10	4.36	4.94	3.94
G6PC	4.36	4.54	4.10
MIR4655	4.36	5.11	3.41
OTTHUMG00000017896	4.36	5.00	3.98
GJB4	4.36	4.87	3.66
DNAH17-AS1	4.36	4.56	4.20
GPRIN3	4.36	4.81	3.98
RPL7	4.36	4.73	4.11
EXPH5	4.36	4.85	4.03
NEXN-AS1	4.36	4.73	4.07
PLD5	4.36	4.66	4.12
PPFIA2	4.36	4.79	3.74
COL21A1	4.36	5.30	3.64

C1QA	4.36	4.90	3.65
SCN8A	4.36	5.10	3.56
ST18	4.36	4.93	4.13
CCDC36	4.36	4.86	3.80
C16orf46	4.36	4.83	4.07
OTTHUMG00000163233	4.36	4.84	3.89
CST9L	4.36	4.87	3.99
DEFB123	4.36	4.70	3.57
ZNF658	4.36	4.65	3.97
LOC643406	4.35	4.77	3.92
NUTF2	4.35	4.76	3.88
CSRNP3	4.35	4.73	4.06
HIST1H1A	4.35	5.19	3.57
MAR10	4.35	4.91	4.12
PRF1	4.35	4.72	3.83
TMSB4Y	4.35	5.04	3.54
OR8H3	4.35	5.16	3.81
OTTHUMG00000171062	4.35	5.12	3.62
LINC00310	4.35	4.73	4.12
TENM2	4.35	4.69	3.94
PRAMEF1	4.35	5.58	2.90
TMC6	4.35	4.59	4.09
OTTHUMG00000173251	4.35	4.82	3.99
VTRNA1-3	4.35	4.95	3.67
IRF8	4.35	5.06	3.89
TUBA3E	4.35	4.60	4.17
TMOD2	4.35	4.84	4.12
TP53I13	4.35	4.94	3.81
OTTHUMG00000171690	4.35	5.02	4.10
HCG22	4.35	5.04	3.51
OTTHUMG00000170968	4.35	4.75	3.89
PRRG3	4.35	5.17	3.82
CDH20	4.35	4.59	4.01
C5orf20	4.35	4.65	4.02
OR2L5	4.35	5.89	3.51
ADAM6	4.35	4.74	3.99
ABTB1	4.35	4.54	4.20
OTTHUMG00000015218	4.35	5.10	3.94
SCRT2	4.35	4.84	3.60
ESRRG	4.35	4.82	3.80
WNT5A-AS1	4.35	4.80	3.77
SKA3	4.35	4.75	3.94
DNAI1	4.35	4.79	3.83
CGB2	4.35	4.83	3.97
PYGM	4.35	4.73	4.10
REEP4	4.35	4.66	3.76
PDCD10	4.35	4.84	3.81
PGAM1	4.35	5.13	3.53
KCNC4-AS1	4.35	4.98	3.97

OTTHUMG00000020566	4.35	5.41	3.88
TMEM179	4.35	4.64	4.10
ZNF45	4.35	4.73	3.91
SH3GL1P2	4.35	4.83	3.10
ACRC	4.35	4.71	4.02
MIR9-1	4.35	4.96	3.79
NAV2-AS4	4.35	4.83	3.93
CSNK1G2-AS1	4.35	4.74	4.05
ZNF793	4.35	4.58	4.05
MISP	4.35	4.67	3.92
OTTHUMG00000154176	4.35	4.52	4.09
PRR16	4.35	4.92	3.75
FCGR1A	4.35	5.20	3.62
KIF20A	4.35	5.01	3.72
LYSMD4	4.35	4.77	3.96
DDN	4.35	4.98	4.03
MIR3118-4	4.35	4.95	3.53
INPP4B	4.35	4.89	3.88
LILRA3	4.35	4.77	4.05
GMNN	4.35	5.40	3.64
LOC100507308	4.35	4.79	4.10
CXorf65	4.35	4.76	3.95
C5orf49	4.35	4.99	3.96
ZNF79	4.35	4.67	3.82
ZNRF3-AS1	4.35	5.04	3.89
UCN	4.35	4.55	4.10
OTTHUMG00000160385	4.35	4.69	3.85
C8orf48	4.35	4.91	3.74
LOC284926	4.35	4.85	3.82
LOC100128703	4.35	4.95	4.13
ST3GAL5	4.35	4.68	4.03
WBSCR17	4.35	4.95	3.90
ENPP7	4.34	4.69	4.03
OR7E2P	4.34	5.02	3.87
GZMA	4.34	4.87	3.73
KDM8	4.34	4.73	3.98
CYP2E1	4.34	4.56	4.02
OTTHUMG00000037758	4.34	4.76	4.04
ASIC2	4.34	4.65	3.91
OTTHUMG00000035686	4.34	5.10	3.80
DNAH10	4.34	4.64	4.12
LOC100128130	4.34	4.99	3.67
OTTHUMG00000008075	4.34	4.87	3.80
PHF21B	4.34	4.84	4.01
LOC441178	4.34	4.90	3.71
SLC13A2	4.34	4.58	3.78
CH25H	4.34	4.90	3.81
ZC2HC1B	4.34	4.83	3.72
YPEL4	4.34	4.79	3.91

BLK	4.34	4.71	4.01
GJD3	4.34	4.80	4.05
LOC150051	4.34	4.86	3.88
OTTHUMG00000177628	4.34	4.89	3.75
NPHS2	4.34	4.64	4.05
GPN3	4.34	4.77	3.49
PIK3IP1	4.34	4.66	4.21
KIAA1257	4.34	4.69	3.88
HOXA11-AS	4.34	4.77	3.98
LOC100131373	4.34	4.85	4.01
TSPAN7	4.34	4.99	3.43
CYP27B1	4.34	4.67	3.95
RSBN1L-AS1	4.34	4.62	3.98
MAP4K2	4.34	4.82	3.86
OTTHUMG00000078329	4.34	5.11	3.87
KRTAP17-1	4.34	4.50	3.86
DKFZp686F0839	4.34	4.70	4.11
TMEM238	4.34	4.58	3.85
KLF14	4.34	4.57	4.03
SPATA41	4.34	4.77	3.82
CLP1	4.34	4.75	3.97
CMA1	4.34	5.04	3.79
RIBC1	4.34	4.56	3.90
EPS8L3	4.34	4.78	3.74
LSM3	4.34	4.69	3.88
C19orf67	4.34	4.68	3.97
OTTHUMG00000086700	4.34	5.17	4.06
CXCL5	4.34	5.03	3.97
CACNG5	4.34	4.75	3.92
ERI3-IT1	4.34	4.60	3.89
OTTHUMG00000176992	4.34	5.12	3.71
TSPAN15	4.34	4.74	3.99
SRGAP3-AS2	4.34	4.84	3.90
FLJ44881	4.34	4.97	3.93
OTTHUMG00000176316	4.34	4.71	4.06
PHYHIP	4.34	4.76	4.19
CENPE	4.34	5.00	3.79
DLG2	4.34	4.65	4.06
THEGL	4.34	4.87	3.52
ZNF467	4.34	4.75	4.07
MLIP-AS1	4.34	5.00	3.90
ZNF707	4.34	4.69	3.92
RNA5SP507	4.34	4.93	3.86
ALK	4.34	4.72	4.01
FGFR4	4.33	4.75	3.87
LINGO3	4.33	4.71	3.84
OTTHUMG00000156028	4.33	5.00	3.53
MIR668	4.33	5.36	3.51
HRNR	4.33	5.05	3.30

FAM217B	4.33	4.61	3.78
RNA5-8SP2	4.33	5.06	3.54
CAPSL	4.33	4.96	3.71
B3GAT3	4.33	4.57	4.09
MIR887	4.33	5.30	3.77
CHRM1	4.33	4.93	3.80
OTTHUMG00000017020	4.33	4.92	3.83
ZNF648	4.33	4.80	3.95
ADAMTSL1	4.33	4.90	3.76
FCRLA	4.33	5.24	3.84
NR1I3	4.33	4.58	3.91
PIH1D2	4.33	4.92	3.87
HCN1	4.33	5.14	4.00
PWP2	4.33	4.60	3.92
RCVRN	4.33	4.59	3.77
OSTCP2	4.33	4.72	3.97
TNFRSF10A	4.33	4.79	3.90
FBXL18	4.33	4.80	3.98
MIR499A	4.33	4.82	3.96
KLHL22	4.33	4.62	3.96
CHORDC1	4.33	4.70	3.54
RND1	4.33	4.97	4.01
OTTHUMG00000162679	4.33	4.66	3.97
LOC100506136	4.33	4.86	4.01
MIR585	4.33	4.70	3.88
CLDN11	4.33	4.63	3.99
LRRC37A4P	4.33	4.74	3.76
SIRPG	4.33	4.86	3.70
HAND1	4.33	4.74	4.06
OTTHUMG00000013631	4.33	5.01	3.79
BCL2L11	4.33	4.67	3.81
WNT6	4.33	4.68	3.88
ERC2-IT1	4.33	4.78	3.90
RNA5SP344	4.33	5.13	3.76
DLEU1	4.33	4.53	4.14
ZNF888	4.33	4.76	3.63
ATF3	4.33	4.83	3.62
LOC100128554	4.33	4.63	4.01
NUDT6	4.33	5.02	3.99
L2HGDH	4.33	4.53	4.14
EPCAM	4.33	4.82	3.93
RAB42	4.33	4.76	3.75
GRIK4	4.33	4.90	3.88
NRADDP	4.33	4.60	3.94
RBM15B	4.33	4.79	3.77
PLA1A	4.33	4.69	4.18
MIR181B1	4.33	5.04	3.69
OTTHUMG00000172468	4.33	4.71	3.75
FAM86EP	4.33	4.90	3.64

CSNK1G2	4.33	4.65	2.91
DPY19L2P1	4.33	4.96	3.86
LOC440894	4.33	5.28	3.76
KRT13	4.33	4.68	3.94
OR10H5	4.33	4.99	3.67
FXYD3	4.33	5.20	3.66
RBMY1A1	4.33	5.41	3.22
VANGL2	4.33	4.69	3.98
SPATA32	4.33	4.86	3.89
OTTHUMG00000144164	4.33	5.31	3.73
TTTY8	4.32	4.91	3.56
DACT2	4.32	4.91	3.95
OTTHUMG00000172085	4.32	4.64	4.08
UNC45B	4.32	4.60	4.16
AACSP1	4.32	4.87	3.62
LOC100131395	4.32	4.66	3.94
LY6K	4.32	5.31	3.97
NCR3LG1	4.32	5.01	3.55
RFXAP	4.32	4.65	3.75
C3orf27	4.32	4.48	4.08
KCNB1	4.32	4.94	3.68
KCNA10	4.32	4.80	3.68
MIR626	4.32	5.02	3.40
DLX2	4.32	5.16	3.85
KIF21A	4.32	4.73	4.01
TMPRSS11D	4.32	4.66	4.09
KRT14	4.32	4.75	4.02
LOC100506881	4.32	4.63	3.80
C15orf27	4.32	4.75	3.88
ZNF444	4.32	4.57	3.91
MAT1A	4.32	4.60	3.94
MIR1270-1	4.32	4.72	4.00
PLA2G2E	4.32	4.61	3.90
LINC00651	4.32	4.64	4.14
GPR139	4.32	4.61	4.01
LINC00656	4.32	4.86	3.93
MIR512-2	4.32	4.54	4.00
MTNR1A	4.32	4.76	4.13
LOC441728	4.32	5.23	3.70
OTTHUMG00000090439	4.32	4.59	4.09
FAM26E	4.32	4.62	3.55
LOC63930	4.32	5.09	3.94
JAZF1-AS1	4.32	4.88	3.87
C9orf147	4.32	4.67	3.78
C10orf62	4.32	4.80	3.97
ADCY1	4.32	4.99	3.79
OTTHUMG00000019300	4.32	4.90	3.51
MMP17	4.32	4.56	3.94
PCP2	4.32	5.01	3.97

TRBV5-6	4.32	5.18	3.31
DNAJC6	4.32	4.75	3.83
HCRTR1	4.32	4.74	3.99
MAPK13	4.32	4.60	4.15
OTTHUMG00000166794	4.32	5.03	3.30
ZNF699	4.32	4.92	3.34
SLC25A30-AS1	4.32	5.05	3.64
NUDT15	4.32	4.79	3.71
C8orf86	4.32	4.86	3.86
FOXRED2	4.32	4.64	3.88
OTTHUMG00000019256	4.32	4.59	3.98
ATXN8OS	4.32	5.02	3.85
GP1BA	4.32	4.66	3.62
SHOX	4.32	4.56	3.99
KIF26B	4.32	4.82	3.77
NIPSNAP3B	4.32	4.67	3.81
EHF	4.32	4.65	3.78
CTRB2	4.32	5.03	3.78
EHD3	4.32	4.78	3.83
GPR97	4.32	4.91	3.78
TMEM233	4.32	4.96	3.91
GBP1	4.32	5.12	3.67
OR1J4	4.32	5.35	3.57
C11orf96	4.32	4.57	4.06
ZNF440	4.32	4.66	3.67
OTTHUMG00000015943	4.32	4.73	3.89
BIN2	4.32	5.10	3.80
KCNA3	4.31	4.74	3.93
VBP1	4.31	4.78	3.89
WNT8A	4.31	5.13	3.79
CYB561D2	4.31	4.94	3.76
OTTHUMG00000163534	4.31	4.73	4.01
B4GALNT4	4.31	4.76	4.15
MIR3681	4.31	4.98	3.60
DBX2	4.31	4.68	4.06
ZNF546	4.31	4.63	3.82
SUSD4	4.31	4.72	3.55
MIR3165	4.31	5.11	3.64
OTTHUMG00000078370	4.31	4.81	3.81
LOC100289092	4.31	4.74	3.87
MIR1539	4.31	4.76	3.69
FAM24B-CUZD1	4.31	4.71	3.89
MUM1L1	4.31	4.62	3.80
C10orf55	4.31	5.82	3.41
ZNF852	4.31	4.63	3.86
PLA2G10	4.31	5.31	3.63
CACNA1A	4.31	6.03	3.65
STAB2	4.31	4.74	4.02
SUMF1	4.31	4.64	3.65

TMEM155	4.31	4.68	4.10
HTR1B	4.31	4.73	3.91
SLC6A2	4.31	4.84	3.93
PGBD4	4.31	4.59	4.04
PCAT1	4.31	5.02	3.85
MIR4306	4.31	5.08	3.93
LOC151174	4.31	4.74	3.86
MAP9	4.31	4.85	3.93
APOF	4.31	4.55	4.03
PDZD2	4.31	4.58	3.92
FGF21	4.31	4.60	3.86
LOC100130587	4.31	4.75	4.11
KIF3C	4.31	4.78	3.80
SH3RF3-AS1	4.31	4.58	4.03
RHBDF2	4.31	4.87	3.85
RNA5SP462	4.31	4.79	3.71
CHST2	4.31	4.58	4.11
SSR4P1	4.31	4.60	3.99
GNG4	4.31	4.64	3.98
LYZL4	4.31	4.52	3.99
PAGE2B	4.31	5.10	3.86
TNFRSF8	4.31	4.63	3.88
OTTHUMG00000173391	4.31	4.86	3.57
PXDNL	4.31	4.87	3.89
PLG	4.31	5.12	3.77
DDX51	4.31	4.57	4.06
TBC1D26	4.31	4.91	3.83
GVQW1	4.31	4.68	3.66
LSAMP-AS1	4.31	4.71	3.93
CASS4	4.31	4.86	3.49
SIX6	4.31	4.81	3.92
FLJ35024	4.31	4.86	3.87
NT5C3A	4.31	4.55	3.80
BOD1L2	4.31	4.58	3.75
ATP6V1C2	4.31	4.69	4.03
BLACE	4.31	4.98	3.69
PLEKHF1	4.31	4.64	4.01
LOC285740	4.31	4.65	4.06
C21orf54	4.31	5.13	3.78
KLHL32	4.31	4.65	3.86
MOCS3	4.31	4.85	3.74
CYB561D1	4.31	4.71	3.67
TRIM31	4.31	4.69	3.91
ZNF672	4.31	4.63	3.85
OTTHUMG00000168234	4.31	4.64	4.04
FADD	4.31	4.63	3.82
TEX101	4.31	5.08	3.75
KIAA1984-AS1	4.31	4.79	4.08
XRCC2	4.31	4.66	4.01

KRTAP4-4	4.31	4.56	3.87
AFAP1-AS1	4.31	4.58	4.13
KCNE3	4.31	4.69	3.95
SNX21	4.31	4.73	3.83
OTTHUMG00000020898	4.31	4.72	3.92
SPACA5	4.31	4.92	3.93
FBXO6	4.31	4.73	3.86
CCNE2	4.31	4.68	3.87
VCY	4.31	4.56	4.06
CHST7	4.30	4.68	3.66
BTBD11	4.30	4.96	3.86
UTS2R	4.30	4.77	3.75
PDE1C	4.30	4.62	3.94
FLJ37201	4.30	4.71	3.76
RPS26	4.30	4.86	3.86
LOC100129924	4.30	4.56	4.01
IKBKG	4.30	4.85	3.97
OTTHUMG00000020899	4.30	4.88	4.07
AKAP4	4.30	4.61	4.03
LOC100287813	4.30	4.91	3.87
CYP17A1	4.30	4.96	3.89
CASP14	4.30	4.73	3.74
GFRA3	4.30	4.72	3.77
COL4A4	4.30	4.56	4.01
PRDM16	4.30	4.55	3.92
ZNF287	4.30	4.66	3.78
KIR2DS4	4.30	4.68	3.75
ZNF653	4.30	4.49	3.87
ZFR2	4.30	4.69	3.84
LOC100506241	4.30	4.95	3.97
IGSF22	4.30	4.67	3.89
NR0B2	4.30	4.78	3.94
TENM4	4.30	4.61	3.85
HNF1A-AS1	4.30	4.79	4.08
DNLZ	4.30	4.80	3.91
KCNC1	4.30	4.51	4.05
LOC100506585	4.30	4.54	4.06
RPL36A-HNRNPH2	4.30	4.71	3.64
GEN1	4.30	4.85	3.36
CPNE4	4.30	4.55	3.97
VILL	4.30	4.72	4.03
RNU7-73P	4.30	4.97	3.63
LINC00612	4.30	4.64	3.89
LOC644090	4.30	4.71	3.88
PRR15L	4.30	4.69	3.80
FLJ35282	4.30	5.36	3.36
NEUROG3	4.30	4.53	3.78
GYG2P1	4.30	5.01	3.98
LMO7-AS1	4.30	4.62	3.99

SRD5A3-AS1	4.30	4.66	3.99
OTTHUMG00000153426	4.30	4.89	3.75
RPL23P8	4.30	5.20	3.49
SPRR2C	4.30	4.97	3.40
LIN9	4.30	4.73	3.79
CCDC85C	4.30	4.67	3.88
ZNF367	4.30	4.52	4.03
LOC284889	4.30	4.82	3.64
C1orf53	4.30	5.15	3.61
FATE1	4.30	4.82	3.89
TMC5	4.30	4.74	3.86
NPY1R	4.30	4.84	3.72
CPA1	4.30	4.72	3.81
SLC13A5	4.30	4.94	3.77
TOP1MT	4.30	4.52	3.90
PRKAR2A-AS1	4.30	4.65	3.98
CACNA1C-IT1	4.30	4.80	3.76
LIPH	4.30	4.73	3.90
FMR1-AS1	4.30	4.63	3.85
OTTHUMG00000153861	4.30	4.75	3.76
OTTHUMG00000153125	4.30	4.54	4.03
AMHR2	4.30	4.66	4.12
CDH6	4.30	5.03	3.81
PATL2	4.30	4.62	3.84
CACNA1E	4.30	4.46	4.08
SRCRB4D	4.30	4.73	3.89
ARHGEF4	4.30	4.57	3.91
MSC	4.30	4.78	3.90
UPK3A	4.30	4.66	4.01
TP63	4.30	4.91	3.89
FLJ37448	4.30	4.55	3.88
EREG	4.30	4.75	3.70
ZNF540	4.29	4.55	4.06
LRP5	4.29	4.53	3.79
C1orf216	4.29	4.67	3.44
LOC339975	4.29	4.80	3.58
C6orf123	4.29	5.06	3.71
SLC4A9	4.29	4.87	3.67
AMIGO3	4.29	4.72	3.93
LOC388553	4.29	4.67	3.98
FUT1	4.29	4.80	3.78
SNAI3-AS1	4.29	4.88	3.87
LOC728339	4.29	4.78	3.91
LINC00052	4.29	4.79	3.47
TCP10L	4.29	4.89	3.91
ECHDC3	4.29	4.55	3.94
CCDC3	4.29	4.72	3.90
GINS1	4.29	4.72	3.91
FAM196B	4.29	4.59	4.02

SLITRK2	4.29	4.65	4.14
CRB1	4.29	4.68	4.00
POPDC3	4.29	4.62	3.92
AKR1CL1	4.29	4.71	3.92
RECQL4	4.29	4.76	3.96
CYMP	4.29	4.74	4.00
KRBA1	4.29	4.65	3.82
MIR4753	4.29	5.25	3.91
DCLRE1C	4.29	4.58	4.00
LOC399744	4.29	4.67	3.75
OTTHUMG00000003649	4.29	4.73	3.77
CASC2	4.29	4.98	3.98
ITGAL	4.29	4.87	3.81
HLTF-AS1	4.29	4.68	3.86
LOC100134368	4.29	4.97	3.72
OTTHUMG00000169209	4.29	5.05	3.57
TRAF1	4.29	4.61	4.03
IDO2	4.29	4.62	3.90
LOC100130987	4.29	4.65	4.05
PRAMEF3	4.29	4.88	3.43
P2RY13	4.29	4.61	3.70
DHRS13	4.29	4.81	3.87
TMEM249	4.29	4.74	3.85
PGM5	4.29	4.86	3.94
CCDC64	4.29	5.18	4.03
NAALADL2-AS1	4.29	4.60	3.92
LRRC2	4.29	4.71	3.77
RHOXF1	4.29	4.85	4.04
CLEC4G	4.29	4.72	3.99
C10orf95	4.29	4.64	3.96
ZNF628	4.29	4.86	3.57
CUX2	4.29	4.95	3.72
FLJ16126	4.29	4.72	3.78
TTC23L	4.29	4.62	3.95
VSX2	4.29	4.66	3.67
KRTAP13-1	4.29	4.72	3.87
RSAD2	4.29	5.26	3.43
ZSCAN5A	4.29	4.85	3.84
OR4F5	4.29	5.33	3.33
GPR113	4.29	4.79	3.68
LRRC37A11P	4.29	4.57	3.84
RAD21-AS1	4.29	4.61	3.64
FANCD2	4.29	4.72	4.00
KLHL10	4.29	4.85	3.95
ZNF649	4.29	4.96	3.81
TSKS	4.29	4.85	3.94
LONRF3	4.29	4.68	3.77
OTTHUMG00000155454	4.29	4.66	3.96
LOC441601	4.29	4.76	3.85

SERP2	4.29	4.75	3.72
SYNDIG1	4.29	4.94	3.16
RPL21P44	4.29	4.86	3.40
OTTHUMG00000162399	4.29	5.15	3.65
PRDM14	4.29	4.73	3.96
MIR25	4.29	4.95	3.52
C6orf164	4.29	4.70	3.77
AIFM2	4.29	4.54	3.81
LOC400553	4.29	4.70	3.94
PCDHB19P	4.29	4.73	3.78
EFNB3	4.29	4.73	4.07
LOC100129316	4.29	4.69	3.85
PADI3	4.29	4.83	3.83
ENOX1-AS2	4.29	4.89	3.76
GPR25	4.29	4.65	3.77
FKBP6	4.29	4.74	3.77
OTTHUMG00000022248	4.29	4.75	3.74
DEFB131	4.29	4.85	3.13
ST13	4.29	4.57	4.02
DIRC3	4.29	4.86	3.63
FAM196A	4.28	4.66	4.03
OTTHUMG00000176975	4.28	4.59	4.08
LY86-AS1	4.28	4.86	3.80
OR10S1	4.28	5.20	3.44
OPLAH	4.28	4.73	3.95
LOC553103	4.28	4.68	3.87
C2orf81	4.28	4.56	3.89
KRTAP1-3	4.28	4.83	3.88
OTTHUMG00000172340	4.28	4.71	3.93
PRDM6	4.28	5.09	3.74
HGFAC	4.28	4.83	4.00
LDOC1L	4.28	4.84	3.84
OTTHUMG00000180596	4.28	4.73	3.96
CMTM2	4.28	4.62	3.86
FLJ46365	4.28	4.88	3.68
ZNF585B	4.28	4.63	3.74
PKP1	4.28	4.72	4.02
ZWINT	4.28	4.67	3.65
NUGGC	4.28	4.60	3.91
MIR32	4.28	4.82	3.88
ANKRD66	4.28	5.00	3.69
SCML1	4.28	4.63	3.81
NEB	4.28	4.47	4.05
RD3L	4.28	5.03	3.78
LOC100507930	4.28	4.68	3.94
FBXO41	4.28	4.52	4.06
CECR5-AS1	4.28	4.78	3.86
ACER2	4.28	4.56	3.90
SCTR	4.28	4.88	3.69

ZNF569	4.28	4.62	3.77
MRPL52	4.28	4.83	3.86
HBCBP	4.28	5.12	3.86
AIDA	4.28	4.89	3.84
PDIA3	4.28	4.96	3.28
OR4A5	4.28	5.00	3.86
LINC00427	4.28	4.67	4.03
VENTX	4.28	4.70	3.96
LLGL2	4.28	4.66	3.84
RPS7P5	4.28	4.80	3.84
GPR84	4.28	4.80	3.88
JAZF1	4.28	4.56	3.90
KIF1A	4.28	4.60	3.93
OTTHUMG00000163170	4.28	4.84	4.01
OTTHUMG00000163385	4.28	4.47	4.07
LOC100287015	4.28	4.83	3.87
TTTY23	4.28	5.12	3.60
NDUFAF6	4.28	4.75	4.02
ABHD1	4.28	4.61	3.97
C1orf191	4.28	4.73	3.86
CHST8	4.28	4.56	4.10
OTTHUMG00000163526	4.28	4.67	3.78
KRT35	4.28	5.01	3.78
NEUROD2	4.28	4.67	3.75
LOC100505532	4.28	4.93	3.94
FLJ16779	4.28	4.54	4.07
SERPINA12	4.28	4.61	3.85
DPPA3	4.28	4.82	3.73
OTTHUMG00000171847	4.28	4.89	3.43
CD163L1	4.28	4.87	3.65
OR10H4	4.28	5.00	2.97
TNFAIP8L1	4.28	4.51	3.61
IP6K3	4.28	4.57	3.86
LCA5	4.28	4.69	3.77
LINC00615	4.28	4.74	3.91
OOSP1	4.28	4.65	3.76
MAGEC1	4.28	5.08	3.49
BTNL3	4.28	4.75	3.68
OR2L2	4.28	4.69	4.05
GFOD2	4.28	4.79	3.87
ENPP4	4.27	4.77	3.67
HBB	4.27	5.46	3.13
FTCDNL1	4.27	4.52	3.82
ST14	4.27	4.76	4.00
VPS37D	4.27	4.63	3.58
RNA5SP274	4.27	4.98	3.73
CEP85L	4.27	4.65	4.01
LOC391722	4.27	4.83	3.85
GEMIN7	4.27	4.79	3.65

OTTHUMG00000162515	4.27	4.75	3.80
OTTHUMG00000002203	4.27	5.30	3.51
C8orf87	4.27	4.84	3.96
FLJ22447	4.27	4.80	3.95
C1orf172	4.27	4.50	3.68
ESM1	4.27	4.82	3.95
TRIM36	4.27	4.52	3.99
LRRC3-AS1	4.27	4.62	3.68
CALCA	4.27	5.08	3.65
LILRA2	4.27	5.05	3.57
TREML4	4.27	4.72	3.91
SNORD113-7	4.27	4.97	3.85
SERTM1	4.27	4.70	3.61
OTTHUMG00000160313	4.27	4.63	3.78
GCSAML-AS1	4.27	4.63	3.96
LURAP1L	4.27	4.80	3.38
C15orf62	4.27	4.45	3.76
LOC100652791	4.27	4.97	3.83
MLF1IP	4.27	4.55	4.02
SNORD58C	4.27	4.88	3.59
ZNF865	4.27	4.85	3.91
MIR640	4.27	4.88	3.43
LOC100127888	4.27	4.85	3.65
MIR4274	4.27	5.10	3.90
IRF5	4.27	4.67	3.99
SSX2	4.27	4.60	3.76
ADAMTS19	4.27	4.74	3.57
LINC00449	4.27	4.69	3.97
OTTHUMG00000154291	4.27	4.54	3.78
BDH1	4.27	4.52	3.95
ARMC6	4.27	4.59	4.04
C11orf21	4.27	4.86	3.71
SLC2A8	4.27	4.51	3.93
OTTHUMG00000032044	4.27	4.98	3.54
OTTHUMG00000017792	4.27	5.02	3.51
SLC52A3	4.27	4.69	3.81
OR5R1	4.27	4.88	3.61
ADRA2A	4.27	4.56	3.73
TEAD3	4.27	4.85	3.62
PAPOLB	4.27	4.80	3.56
TFPI2	4.27	4.94	3.77
FLJ46361	4.27	4.82	3.79
DLGAP1-AS3	4.27	4.77	3.74
AJUBA	4.27	4.69	3.81
ST8SIA4	4.27	5.02	3.65
AQP8	4.27	4.59	3.50
VIPR2	4.27	4.60	3.92
LINC00280	4.27	5.22	3.33
C13orf45	4.27	4.64	3.85

PDE4B	4.27	4.59	4.00
FLJ45513	4.27	4.60	3.73
DLX1	4.27	4.94	3.71
OTTHUMG00000017959	4.27	4.83	3.42
OTTHUMG00000020610	4.27	4.93	4.01
BRF2	4.27	4.49	3.93
ECE2	4.27	4.61	4.02
MNX1	4.27	4.58	3.84
ACE	4.26	4.65	3.87
ANKRD24	4.26	4.90	3.68
C9orf152	4.26	4.78	3.92
C1orf198	4.26	4.57	3.81
OTTHUMG00000168607	4.26	4.86	3.84
NMNAT3	4.26	4.60	3.91
LOC100507108	4.26	4.71	3.79
MRAS	4.26	4.74	3.54
DMRT3	4.26	4.70	3.90
OTTHUMG00000151413	4.26	4.70	3.86
MID1IP1-AS1	4.26	4.53	3.74
GJA3	4.26	4.41	3.90
MIR4708	4.26	4.71	3.76
IGJ	4.26	5.73	3.53
TMSB15B	4.26	4.75	3.94
EXO5	4.26	4.72	3.97
COLEC11	4.26	4.59	4.07
PDE8B	4.26	4.69	3.73
RP1L1	4.26	4.73	3.58
ITGA2	4.26	5.05	3.74
KLRC2	4.26	4.72	3.54
SNORA35	4.26	4.67	3.80
SH2B2	4.26	4.66	3.99
KRT10	4.26	4.49	3.92
RNASE13	4.26	4.74	3.90
C2orf88	4.26	4.37	4.00
OTTHUMG00000163341	4.26	4.67	4.03
GUSBP10	4.26	4.77	3.50
MCAT	4.26	4.89	3.85
RIN3	4.26	4.68	3.59
FARP1-IT1	4.26	4.48	3.72
CACNG1	4.26	4.81	3.99
MUC17	4.26	4.75	3.81
SOAT2	4.26	4.65	3.84
ERCC8	4.26	4.46	3.90
DISC2	4.26	4.81	3.78
LOC648691	4.26	4.77	3.52
FAM84A	4.26	4.52	3.87
C22orf43	4.26	4.75	3.77
MAPK15	4.26	4.61	3.74
RFC4	4.26	4.57	3.92

GNG8	4.26	4.60	3.82
PLA2G2F	4.26	4.68	3.91
SLC35G1	4.26	4.49	3.65
OPN3	4.26	4.49	4.04
CACNA1B	4.26	4.70	3.88
SH2D2A	4.26	4.71	3.69
IDH1-AS1	4.26	4.55	3.80
MYB-AS1	4.26	4.72	3.92
C19orf45	4.26	4.63	4.02
ACTL10	4.26	4.75	3.93
SNORA70B	4.26	4.94	3.42
VRTN	4.26	4.64	3.52
OTTHUMG00000078325	4.26	4.45	4.03
LINC00859	4.26	4.74	3.95
ADCYAP1	4.26	4.76	3.81
LILRA6	4.26	5.58	3.09
OTTHUMG00000160312	4.25	4.67	3.73
TMEM26	4.25	4.71	3.90
NEUROD4	4.25	4.75	3.85
SLC2A4	4.25	4.71	3.70
CHRNA4	4.25	4.49	3.92
FLJ25917	4.25	4.57	3.92
CHCHD7	4.25	4.65	3.97
MIR4462	4.25	4.77	3.57
LSAMP	4.25	4.63	3.74
PMS1	4.25	4.55	3.88
KRBOX1	4.25	4.74	3.76
SLC26A8	4.25	4.86	3.73
ZFHX4-AS1	4.25	4.58	3.50
SLC17A7	4.25	4.62	3.99
LOC100270746	4.25	4.68	3.55
MCIDAS	4.25	4.75	3.83
POU2AF1	4.25	4.49	3.75
MAP6	4.25	4.59	3.89
ATG10-AS1	4.25	4.69	3.39
RAD51C	4.25	4.65	3.82
FLJ44385	4.25	4.52	3.83
WFDC5	4.25	4.62	3.83
DDX26B-AS1	4.25	4.85	3.84
FOXI2	4.25	4.51	4.05
SLC1A2	4.25	4.54	3.77
PRR24	4.25	4.71	3.80
PRR15	4.25	4.84	3.82
SRD5A3	4.25	4.77	3.73
FGF23	4.25	4.96	3.66
HIST1H3J	4.25	5.14	3.61
GPX2	4.25	4.45	3.72
SLC16A11	4.25	4.67	3.78
PLEKHG4B	4.25	4.52	3.88

LOC284950	4.25	4.55	3.77
LOC286083	4.25	4.74	3.89
LAMB3	4.25	4.60	3.98
C15orf59	4.25	4.71	3.79
GLB1L	4.25	4.59	3.82
LINC00398	4.25	4.56	3.70
LOC731779	4.25	4.86	3.88
KCNA2	4.25	4.87	3.76
ACY3	4.25	4.78	3.96
FABP5P3	4.25	4.94	3.92
OTTHUMG00000161141	4.25	4.62	3.78
OR2V2	4.25	5.46	2.87
LRRC26	4.25	4.82	3.59
C19orf59	4.25	4.50	3.94
MAGEE1	4.25	4.52	3.98
LOC100132354	4.25	4.75	3.71
LSMEM1	4.25	4.74	3.87
OTTHUMG00000020779	4.25	4.98	3.75
SEC62-AS1	4.25	5.32	3.25
MMD	4.25	4.60	3.80
GAS2L2	4.25	4.75	3.89
HOXD12	4.25	4.88	3.79
SLC45A3	4.25	4.58	3.80
RSPH6A	4.25	4.70	3.93
OTTHUMG00000170833	4.25	4.71	3.69
IL19	4.25	4.73	3.91
HPR	4.25	4.85	2.91
PON3	4.24	5.05	3.43
LOC100507162	4.24	4.53	3.88
DNAH5	4.24	4.50	3.92
SALL2	4.24	4.80	3.97
ZSWIM4	4.24	4.45	3.86
LOC646743	4.24	5.08	3.65
NMS	4.24	4.86	3.72
LOC100506385	4.24	4.80	3.68
OTTHUMG00000151743	4.24	4.69	3.95
RRS1	4.24	4.49	3.99
ZP1	4.24	4.68	3.88
KANK4	4.24	4.60	3.77
OTTHUMG00000163588	4.24	4.65	3.97
OTTHUMG00000172997	4.24	4.84	3.46
CDRT7	4.24	4.86	3.66
MUC4	4.24	4.53	3.96
MIG7	4.24	4.71	3.90
OTTHUMG00000155112	4.24	4.83	3.44
PRSS38	4.24	4.83	3.92
ABCA8	4.24	4.93	3.19
OTTHUMG00000009145	4.24	4.65	4.03
ALDH5A1	4.24	4.55	3.66

FOXJ1	4.24	4.53	3.48
LOC113230	4.24	4.66	3.69
NARFL	4.24	4.49	3.93
MRRF	4.24	5.14	3.18
LOC339192	4.24	4.76	3.84
MANSC4	4.24	4.53	3.89
CLRN1	4.24	4.76	4.03
OR52M1	4.24	4.50	3.91
SNORD104	4.24	5.19	3.29
CD8B	4.24	4.42	3.98
DNAH9	4.24	4.43	3.96
OTTHUMG00000178556	4.24	4.82	3.84
HTR4	4.24	4.66	3.66
DNASE1	4.24	4.83	3.99
HCCAT3	4.24	4.90	3.69
RDH13	4.24	4.60	3.93
TTLL10-AS1	4.24	4.71	3.90
MIR551A	4.24	4.94	3.75
KCNJ2-AS1	4.24	4.73	3.56
NACAP1	4.24	4.73	3.78
OR6C75	4.24	5.03	3.53
ACN9	4.24	4.62	4.01
ACSBG1	4.24	4.59	3.93
RELN	4.24	4.55	3.86
LOC100288846	4.24	4.60	3.82
PTCHD4	4.24	5.00	3.75
LOC283665	4.24	4.63	3.64
HELB	4.24	4.70	3.91
HSD17B2	4.24	4.88	3.93
ZNF627	4.24	4.65	3.47
NLRP10	4.24	4.76	3.78
PSG3	4.24	5.02	3.73
WDPCP	4.24	4.69	3.65
NEBL-AS1	4.24	4.57	3.93
ACER3	4.24	4.57	3.81
SUSD3	4.24	4.51	3.97
DUSP19	4.24	4.80	3.88
NLRP6	4.24	4.76	3.82
TNFSF8	4.24	5.70	3.58
LOC643797	4.24	4.91	3.52
C14orf178	4.24	4.99	3.94
SLC6A13	4.24	4.57	4.03
FAM227B	4.24	4.73	3.78
OTTHUMG00000175407	4.24	4.73	3.88
LOC100506317	4.23	4.54	3.81
ZNF461	4.23	4.96	3.62
EDDM3B	4.23	4.98	3.66
ST3GAL6-AS1	4.23	4.71	3.76
C6orf170	4.23	4.77	3.76

ATG9B	4.23	4.79	3.89
SYT3	4.23	4.59	3.78
LIMD1-AS1	4.23	4.48	3.83
FLJ46836	4.23	4.59	3.74
GNAS-AS1	4.23	4.53	4.01
PARD6G	4.23	4.70	3.78
SCGB2B3P	4.23	4.54	3.69
KLHDC7A	4.23	4.51	3.86
CXADRP3	4.23	4.89	3.55
MEST	4.23	5.20	3.42
OTTHUMG00000165008	4.23	4.87	3.96
OTTHUMG00000156244	4.23	4.72	3.68
OPRD1	4.23	4.83	3.75
ZFP92	4.23	4.42	3.91
LOC340512	4.23	4.52	3.91
LOC100996590	4.23	4.59	3.69
LEP	4.23	5.75	3.53
OTTHUMG00000017568	4.23	4.54	3.94
C7orf57	4.23	4.70	3.91
SNAP47-AS1	4.23	4.44	4.00
RXFP3	4.23	4.55	3.56
GUCY1B2	4.23	4.63	3.88
DAZ3	4.23	5.76	3.26
SERPINB2	4.23	4.58	3.71
ZNF781	4.23	4.85	3.73
OTTHUMG00000169548	4.23	4.59	3.87
LOC400620	4.23	4.71	3.68
NOBOX	4.23	4.63	3.60
DOK6	4.23	4.83	3.44
LOC153910	4.23	4.71	3.94
NDUFB2	4.23	4.52	3.90
RYR2	4.23	4.74	3.92
IL18R1	4.23	4.49	3.96
ANTXRL	4.23	4.70	3.80
LINC00472	4.23	4.80	3.30
OTTHUMG00000014380	4.23	4.80	3.60
AGBL4	4.23	4.76	3.89
PPFIA3	4.23	4.63	3.68
SNORD58A	4.23	5.51	3.34
TMEM68	4.23	4.55	3.91
PTPRN	4.23	4.72	3.86
SLC14A2	4.23	4.71	3.73
TBC1D3P1-DHX40P1	4.23	4.87	3.77
OTTHUMG00000132237	4.22	5.47	2.75
AAED1	4.22	5.06	3.37
KRTAP5-8	4.22	4.93	3.83
C9orf129	4.22	4.64	3.88
OR51T1	4.22	4.73	3.91
KRT74	4.22	4.70	3.92

VRK1	4.22	4.91	3.69
FAM159B	4.22	4.74	3.59
PLEKHH1	4.22	4.55	3.97
TVP23A	4.22	4.89	3.73
GRIP2	4.22	4.62	3.85
FAM90A12P	4.22	4.67	3.84
LOC100631378	4.22	4.43	3.92
OTTHUMG00000022540	4.22	5.11	3.66
FAM53B	4.22	4.47	3.94
HSD52	4.22	4.55	3.99
WNT10A	4.22	4.57	3.94
OTTHUMG00000154837	4.22	4.86	3.70
NR2E3	4.22	4.48	3.81
OTTHUMG00000074395	4.22	4.51	3.75
PTRHD1	4.22	4.48	3.96
KRTAP4-2	4.22	4.64	3.60
CCDC89	4.22	4.56	3.58
LOC400692	4.22	4.53	3.85
CDYL2	4.22	4.43	4.07
C12orf54	4.22	4.52	3.92
FLJ16341	4.22	4.63	3.59
C4orf17	4.22	4.76	3.78
RNU1-21P	4.22	4.67	3.30
HSD17B12	4.22	4.39	3.87
LOC100507071	4.22	4.74	3.98
OTTHUMG00000159120	4.22	4.45	3.76
GRK1	4.22	4.79	3.80
SNORD116-30	4.22	5.23	3.14
TRIM51	4.22	5.16	3.14
SHROOM2	4.22	4.66	3.61
LRRN4	4.22	4.57	3.86
HMBS	4.22	4.48	3.81
OTTHUMG00000160427	4.22	4.74	3.87
C14orf177	4.22	4.60	3.98
FARP1-AS1	4.22	4.98	3.68
TBL2	4.22	4.76	3.90
HAS1	4.22	4.53	3.60
ICA1L	4.22	4.64	3.84
PEG10	4.22	4.60	3.79
LOC338817	4.22	4.69	3.62
ZNF74	4.22	4.57	3.65
OTTHUMG00000171511	4.22	5.03	3.72
ZBTB5	4.22	4.47	3.95
ANKRD33	4.22	4.68	3.89
ADAM20	4.22	4.55	3.94
LOC100506075	4.22	4.81	3.44
MELK	4.22	5.14	3.37
PRAMEF20	4.22	4.99	3.65
ADAMTS7	4.22	4.60	3.73

ZNF846	4.22	4.78	3.70
LY96	4.22	5.07	3.36
RBMY1J	4.22	5.40	3.26
CRLF2	4.22	4.82	3.80
OTTHUMG00000171772	4.22	4.89	3.69
VPS9D1	4.22	4.53	3.82
NAT16	4.22	4.60	3.65
TMEM145	4.22	4.58	3.75
RFK	4.22	4.50	3.80
LOC100507006	4.22	5.00	3.64
ZNF382	4.21	4.77	3.55
FAM69C	4.21	4.74	3.79
KCTD21-AS1	4.21	4.79	3.62
OTTHUMG00000151357	4.21	4.67	3.71
PDP1	4.21	4.53	3.97
C5orf28	4.21	4.57	3.82
RNA5SP105	4.21	4.69	3.61
LINC00629	4.21	4.55	3.90
FHDC1	4.21	4.73	3.20
FLJ25715	4.21	4.46	3.74
SMIM11P1	4.21	4.84	3.38
OTTHUMG00000164540	4.21	4.69	3.74
C21orf119	4.21	4.58	3.88
GDA	4.21	4.44	3.81
OTTHUMG00000019964	4.21	5.18	3.58
OR11H2	4.21	5.16	3.57
BPIFA3	4.21	4.69	3.84
HMBOX1-IT1	4.21	4.67	3.71
NIPAL2	4.21	4.39	3.76
EBI3	4.21	4.51	3.88
AMELX	4.21	4.98	3.73
LOC401324	4.21	4.89	3.35
DCTN1-AS1	4.21	4.46	4.02
IL36G	4.21	4.74	3.82
OTTHUMG00000162055	4.21	4.79	3.59
DMRTA1	4.21	4.61	3.79
TMEM52B	4.21	4.82	3.87
FZD2	4.21	4.75	3.48
ZNF471	4.21	5.11	3.44
LOC100506236	4.21	4.99	3.88
GPR89A	4.21	4.94	3.62
MOBP	4.21	4.95	3.54
GTDC2	4.21	4.52	3.94
UPK1A	4.21	4.81	3.87
GPR52	4.21	4.58	3.64
RND2	4.21	4.63	3.85
GPR112	4.21	5.03	3.74
LINC00032	4.21	4.64	3.85
UNQ6975	4.21	4.47	3.83

NR4A1	4.21	4.66	3.64
PVRL1	4.21	4.39	3.97
TREH	4.21	4.55	3.93
TRBV9	4.21	4.63	3.54
OTTHUMG00000165055	4.21	4.99	3.58
ZNF69	4.21	4.64	3.84
PHKG1	4.21	4.67	3.31
ZFAT-AS1	4.21	4.86	3.86
LOC643339	4.21	4.55	3.86
OTTHUMG00000178053	4.21	4.71	3.74
ALDH8A1	4.21	4.43	3.85
OTTHUMG00000162974	4.21	5.05	3.75
RASL10A	4.21	4.70	3.86
GJD4	4.21	4.59	3.72
LOC100996570	4.21	4.69	3.85
LOC100507651	4.21	4.61	3.87
OTTHUMG00000158362	4.21	4.52	3.78
IQCJ	4.21	4.60	3.69
KIF25	4.21	4.59	3.49
HSPB7	4.21	4.52	3.64
SCARNA11	4.21	5.03	2.72
EHHADH	4.20	4.69	3.68
SP5	4.20	4.38	3.98
ERICH1-AS1	4.20	4.54	3.73
RNU4ATAC3P	4.20	5.18	3.40
IL13	4.20	5.05	3.71
OTTHUMG00000162158	4.20	5.06	3.69
BREA2	4.20	4.64	3.72
HES3	4.20	4.56	3.72
CEP55	4.20	4.94	3.79
LRRC8C	4.20	4.50	3.90
OTTHUMG00000167194	4.20	4.99	3.45
DOK5	4.20	5.50	3.30
VWA3B	4.20	4.69	4.01
C19orf73	4.20	4.56	3.77
HSPB2-C11orf52	4.20	4.44	3.91
OTTHUMG00000150840	4.20	4.46	3.85
GREB1L	4.20	4.60	3.71
DENND2A	4.20	4.66	3.57
FRMD5	4.20	4.64	3.79
PDCD6	4.20	4.62	3.66
GPD1	4.20	4.74	3.74
LOC100289495	4.20	4.59	3.95
OTTHUMG00000074332	4.20	5.17	3.48
HULC	4.20	4.51	3.93
CLCN1	4.20	4.75	3.65
LOC146481	4.20	4.97	3.24
MCTS1	4.20	4.66	3.77
OTTHUMG00000014325	4.20	4.59	3.98

OTTHUMG00000011762	4.20	4.81	3.84
PIM2	4.20	4.65	3.67
OTTHUMG00000020886	4.20	4.61	3.85
IRF7	4.20	4.53	3.90
NR4A3	4.20	4.97	3.81
FRMPD2	4.20	4.65	3.86
CLEC19A	4.20	4.59	3.78
LAMC3	4.20	4.75	3.77
OBP2B	4.20	4.71	3.35
ALAS2	4.20	4.79	3.70
ANKEF1	4.20	4.57	3.80
USP2-AS1	4.20	4.83	3.88
DEFB107A	4.20	4.54	3.62
RNA5SP165	4.20	4.85	2.92
EPHA2	4.20	4.86	3.85
OTTHUMG00000177103	4.20	4.81	3.63
MIR2276	4.20	4.81	3.43
STK24-AS1	4.20	4.49	3.94
C7orf25	4.20	4.54	3.61
NOVA1	4.20	4.58	3.68
NAV3	4.20	4.66	3.71
CD244	4.20	4.97	3.88
DLG5-AS1	4.20	5.35	3.74
SNX16	4.20	4.54	3.54
MIR602	4.20	4.61	3.41
LOC399884	4.20	4.86	3.66
ZNF204P	4.20	4.83	3.31
OTTHUMG00000152376	4.20	4.87	3.43
MTUS2	4.20	4.48	3.80
CHIT1	4.20	4.75	3.40
RNA5SP352	4.20	5.18	3.61
NUDT10	4.20	4.55	3.50
OTTHUMG00000165718	4.20	4.75	3.66
LOC100131662	4.20	4.52	3.84
ZNF735	4.20	5.19	3.35
MIR129-2	4.20	4.74	3.78
MIR2117	4.20	4.71	3.74
LOC100507091	4.20	4.62	3.31
TRAJ26	4.20	5.28	3.40
HPSE2	4.20	4.42	3.84
SPEM1	4.20	4.64	3.68
LIFR-AS1	4.20	4.53	3.47
F7	4.20	4.96	3.56
ASB14	4.20	4.47	3.34
SRRM2-AS1	4.19	4.64	3.71
OTTHUMG00000032284	4.19	4.66	3.77
OTTHUMG00000155365	4.19	4.68	3.81
OTTHUMG00000163864	4.19	4.56	3.82
LOC286442	4.19	4.45	3.97

MOB2	4.19	4.50	3.79
PKDREJ	4.19	4.62	3.18
HLA-J	4.19	4.61	3.46
NOS1	4.19	4.80	3.89
RNA5SP129	4.19	4.98	3.53
LOC100507003	4.19	4.59	3.93
DAZ2	4.19	5.70	3.19
ATP5L	4.19	4.39	3.82
FRS3	4.19	4.48	3.75
TCL6	4.19	4.72	3.90
OTTHUMG00000163518	4.19	4.64	3.64
SMIM2	4.19	4.61	3.83
ASF1B	4.19	4.48	3.76
CACNB2	4.19	4.73	3.81
WDR16	4.19	4.71	3.67
TUBAL3	4.19	4.57	3.59
CBX8	4.19	4.68	3.60
FBXO16	4.19	4.57	3.87
GPR22	4.19	4.71	3.47
OTTHUMG00000041395	4.19	4.52	3.71
SERPINF2	4.19	4.99	3.57
HTR5A	4.19	4.78	3.51
TMIE	4.19	4.67	3.85
RP11-631M21.1	4.19	5.32	3.55
TOP1P1	4.19	4.57	3.74
IL25	4.19	4.94	3.53
TRUB1	4.19	4.68	3.63
MYT1L	4.19	4.54	3.91
UTP14A	4.19	5.07	3.68
PLIN4	4.19	4.71	3.91
IFIT1	4.19	5.07	3.50
MIR320E	4.19	4.61	3.63
C20orf26	4.19	4.53	3.83
CLRN2	4.19	4.68	3.87
PWRN2	4.19	4.77	3.64
C18orf56	4.19	4.47	3.92
CPNE7	4.19	4.74	3.84
DNAJC9	4.19	4.76	2.88
MAN1C1	4.19	4.51	3.75
VMA21	4.19	4.51	3.83
SPAG5	4.19	4.59	3.69
ARMC12	4.19	4.53	3.88
TUBA3D	4.19	4.92	3.61
ZNF711	4.19	4.61	3.82
WDR65	4.19	4.83	3.81
OTTHUMG00000163338	4.19	5.21	3.67
FOXN1	4.19	4.50	3.63
SHANK2-AS1	4.19	4.46	3.80
CABP5	4.19	5.28	3.82

RNA5SP108	4.19	5.04	3.82
LIPE-AS1	4.19	4.82	3.95
OTTHUMG00000018554	4.19	4.80	3.41
DCLK2	4.19	4.60	3.81
ZNF812	4.19	4.63	3.77
CD274	4.19	4.50	3.79
IMPA2	4.19	4.43	3.92
HIST1H3A	4.19	5.39	3.06
LOC619207	4.19	4.44	3.95
LOC100506289	4.19	4.56	3.85
KRTAP10-7	4.19	4.77	3.45
OTTHUMG00000032833	4.19	4.75	3.69
SLC25A2	4.19	4.67	3.66
PDE6A	4.19	4.62	3.55
TUBBP5	4.19	4.94	3.68
NXF5	4.19	4.70	3.61
CD6	4.19	4.92	3.69
CDC42BPG	4.19	4.42	4.04
CCDC103	4.19	4.73	3.88
FAM186B	4.19	4.75	3.81
LAMB4	4.19	4.80	3.79
IKZF3	4.18	4.47	3.71
FAM76B	4.18	4.63	3.69
DLEC1	4.18	4.62	3.79
PSD4	4.18	4.58	3.94
OTTHUMG00000163594	4.18	4.54	3.82
DUOX2	4.18	4.55	3.77
ZPLD1	4.18	4.60	3.74
KLKB1	4.18	4.85	3.46
LINC00528	4.18	4.80	3.56
OTTHUMG00000179558	4.18	4.92	3.55
OACYLP	4.18	4.70	3.73
OTTHUMG00000031951	4.18	5.07	3.28
POTEC	4.18	5.06	3.30
ADRA2B	4.18	4.40	3.68
OTTHUMG00000032051	4.18	4.58	3.75
OTTHUMG00000017875	4.18	4.46	3.84
TRPC7	4.18	4.59	3.73
FGF19	4.18	4.79	3.55
SLC8A3	4.18	4.64	3.62
SPSB1	4.18	4.77	3.65
KRTAP6-2	4.18	4.48	3.79
MYOM3	4.18	4.44	4.00
DKFZP434I0714	4.18	4.67	3.62
MIR3936	4.18	5.03	3.48
OTTHUMG00000172243	4.18	4.84	3.83
OTTHUMG00000015353	4.18	4.59	3.87
OTTHUMG00000171423	4.18	4.60	3.75
ANP32AP1	4.18	4.70	3.74

KBTBD13	4.18	4.79	3.63
SH3TC2	4.18	4.71	3.57
AADACL2	4.18	4.44	4.01
LINC00184	4.18	4.96	3.72
DHX37	4.18	4.50	3.68
OTTHUMG00000160619	4.18	4.87	3.57
BTNL8	4.18	4.95	3.71
DEFB136	4.18	4.79	3.60
BDNF	4.18	4.45	3.65
GPR148	4.18	4.81	3.77
LHFPL1	4.18	4.54	3.76
ATP6V0E2	4.18	4.78	3.69
OTTHUMG00000162996	4.18	4.54	3.59
MRO	4.18	4.57	3.77
HCG18	4.18	4.35	3.88
OTTHUMG00000003643	4.18	5.03	3.04
SYNGR4	4.18	4.84	3.57
ARHGAP26-AS1	4.18	4.63	3.85
OTTHUMG00000019561	4.18	4.46	3.87
ZNF215	4.18	4.71	3.69
PROM1	4.18	4.60	3.87
WBP11P1	4.18	4.91	3.63
VAX1	4.18	4.59	3.90
DNAAF2	4.18	4.90	3.60
IGLV4-60	4.18	4.72	3.52
DRD3	4.18	4.61	3.79
LINC00705	4.18	4.75	3.67
ASB9P1	4.18	4.86	3.76
ELOVL2-AS1	4.18	4.61	3.90
ACTR6	4.18	4.79	3.35
OTTHUMG00000173079	4.18	4.64	3.84
DKFZp686K1684	4.18	4.54	3.94
WRAP73	4.18	4.73	4.03
EDA	4.18	4.53	3.94
LGR5	4.18	4.48	3.75
MIR548K	4.18	4.80	3.42
TIGD2	4.18	4.65	3.64
TEKT5	4.18	4.66	3.29
RNA5SP419	4.18	4.69	3.61
GRAP2	4.18	4.63	3.62
C5orf27	4.18	4.96	3.63
SPEF1	4.18	4.52	3.79
LOC642426	4.17	4.74	3.44
LGI1	4.17	4.87	3.83
RYR3	4.17	4.76	3.92
GHRH	4.17	4.53	3.86
OTTHUMG00000171678	4.17	4.48	3.69
UBXN2B	4.17	4.71	3.61
BTNL2	4.17	4.83	3.74

OTTHUMG00000040900	4.17	4.63	3.57
CDC42SE2	4.17	4.34	3.86
PRKACG	4.17	4.47	3.96
CENPW	4.17	5.06	2.61
HAPLN4	4.17	4.80	3.51
CIRBP-AS1	4.17	4.68	3.78
MB21D1	4.17	4.61	3.52
FLJ44006	4.17	4.90	3.82
C1orf94	4.17	4.70	3.79
OTTHUMG00000151708	4.17	4.55	3.58
DYNAP	4.17	4.62	3.78
SSH1	4.17	4.60	3.68
RNA5SP439	4.17	4.83	3.48
ATP10B	4.17	4.40	3.98
OSBPL10-AS1	4.17	4.64	3.82
YOD1	4.17	4.41	4.03
DNAJC9-AS1	4.17	4.75	3.67
SYP	4.17	4.54	3.82
LOC284898	4.17	5.11	3.59
MIR509-3	4.17	5.14	3.06
PP2D1	4.17	4.77	3.66
RHEBL1	4.17	4.63	3.67
LOC391322	4.17	4.91	3.61
LOC388282	4.17	4.66	3.73
QPCT	4.17	4.57	3.73
ITPK1-AS1	4.17	4.67	3.85
RCHY1	4.17	4.74	3.63
EME1	4.17	4.49	3.82
TNFRSF13C	4.17	4.64	3.89
ZFYVE28	4.17	5.08	3.32
HOXB4	4.17	4.62	3.48
LOC389641	4.17	4.69	3.72
IFNG	4.17	5.03	3.68
C10orf107	4.17	4.76	3.58
FLT4	4.17	4.61	3.85
PRAMEF19	4.17	4.56	3.67
OTTHUMG00000153431	4.17	4.68	3.83
OTTHUMG00000018428	4.17	4.68	3.69
OTTHUMG00000169584	4.17	4.75	3.35
EXOSC5	4.17	4.57	3.70
LOC100507489	4.17	4.83	3.85
C21orf49	4.17	4.51	3.87
TRAV9-1	4.17	4.79	3.44
RTP3	4.17	4.63	3.54
LAMB2P1	4.17	4.71	3.74
LOC100288842	4.17	4.71	3.59
OTTHUMG00000014286	4.17	4.56	3.64
OTTHUMG00000015298	4.17	4.72	3.88
EVA1A	4.16	4.78	3.87

SLAIN1	4.16	4.65	3.74
LOC100128164	4.16	4.75	3.80
SLAMF7	4.16	4.54	3.69
SALL1	4.16	4.51	3.92
WNK4	4.16	4.82	3.50
MIR1243	4.16	4.92	3.50
RBM26-AS1	4.16	4.63	3.73
PLEKHA7	4.16	4.56	3.79
TP53AIP1	4.16	4.45	3.89
LOC387810	4.16	4.71	3.56
ZNF831	4.16	4.68	3.83
TMTC2	4.16	4.78	3.44
OTTHUMG00000016631	4.16	4.53	3.91
RAB44	4.16	4.71	3.52
TTTY5	4.16	4.75	3.67
PANX2	4.16	4.54	3.73
RIMS1	4.16	4.53	3.47
EFCAB4B	4.16	4.57	3.72
LOC100507403	4.16	4.73	3.66
CKAP2L	4.16	4.70	3.58
FMO1	4.16	4.63	3.80
TRAIP	4.16	4.55	3.81
LOC100289308	4.16	4.62	3.72
LOC644936	4.16	4.92	3.20
CHRNB4	4.16	4.46	3.93
RNA5SP137	4.16	4.66	3.71
MIR146A	4.16	4.55	3.48
OTTHUMG00000164202	4.16	4.72	3.32
TCP10	4.16	4.63	3.57
SLCO5A1	4.16	4.52	3.89
OR51H1P	4.16	4.65	3.80
TMEM139	4.16	4.59	3.66
ENO4	4.16	4.36	3.74
CHRDL1	4.16	4.59	3.68
RPS6KA2-AS1	4.16	4.72	3.88
GSTT2	4.16	4.74	3.49
NSG1	4.16	4.43	3.76
OTTHUMG00000058513	4.16	4.48	3.89
UNC79	4.16	4.69	3.79
OTTHUMG00000151817	4.16	4.97	3.56
IGHV3-38	4.16	4.94	3.54
PHGR1	4.16	4.62	3.79
E2F7	4.16	4.56	3.76
NTNG2	4.16	4.41	3.93
JAKMIP2-AS1	4.16	4.56	3.70
LOC100506013	4.16	4.53	3.30
ALPPL2	4.16	5.05	3.55
NFE4	4.16	4.59	3.70
OTTHUMG00000162304	4.16	4.75	3.61

OTTHUMG00000037610	4.16	4.94	3.42
RSPO3	4.16	4.78	3.26
MRPL49	4.16	4.72	3.47
OR5F1	4.16	4.57	3.67
NRIP3	4.16	4.58	3.70
C4BPA	4.16	5.01	3.71
MYH7B	4.16	4.52	3.64
TTLL6	4.16	4.39	3.72
MIR101-2	4.16	6.13	3.18
PCK1	4.16	4.51	3.77
OCA2	4.16	5.01	3.73
LBX2	4.16	4.46	3.72
PGC	4.16	4.61	3.89
KCNK10	4.16	5.05	3.75
IRX5	4.15	4.65	3.55
GPR119	4.15	4.61	3.31
CACNG6	4.15	4.44	3.85
OTTHUMG00000058802	4.15	4.63	3.66
TSPEAR	4.15	4.44	3.80
DCAF11	4.15	4.49	3.69
HPCAL4	4.15	4.64	3.80
OTTHUMG00000169945	4.15	4.58	3.66
FERMT3	4.15	4.41	3.92
OSER1-AS1	4.15	4.40	4.01
CFL1P1	4.15	4.98	3.55
NAIF1	4.15	4.41	3.73
OTTHUMG00000164252	4.15	4.75	3.82
TMEM35	4.15	4.52	3.87
OTTHUMG00000019251	4.15	4.75	3.71
C19orf18	4.15	4.43	3.77
SYT7	4.15	4.69	3.70
OTTHUMG00000152023	4.15	5.10	3.59
DEFB118	4.15	4.92	3.34
KLHL40	4.15	4.51	3.68
LOC221122	4.15	4.51	3.87
RASGRP2	4.15	4.84	3.48
SOX21	4.15	4.67	3.86
ELF3	4.15	4.49	3.60
CEACAM22P	4.15	4.63	3.76
F2RL2	4.15	4.72	3.75
LOC100616530	4.15	4.82	3.68
OTTHUMG00000020204	4.15	4.56	3.88
OR56B1	4.15	4.98	3.00
OTTHUMG00000160102	4.15	4.49	3.74
INHBE	4.15	4.42	3.88
LOC285389	4.15	4.76	3.66
OTTHUMG00000169123	4.15	4.62	3.95
LOC100507079	4.15	4.62	3.61
HCG4B	4.15	4.47	3.87

GCNT2	4.15	4.52	3.68
LOC221272	4.15	4.35	4.00
LOC100129048	4.15	4.82	3.67
OTTHUMG00000166132	4.15	4.53	3.59
OR7D2	4.15	4.76	3.75
AGXT2	4.15	4.53	3.77
C17orf64	4.15	4.36	3.78
EXD1	4.15	4.54	3.58
CASP12	4.15	4.55	3.82
PLAG1	4.15	4.74	3.88
NKAIN4	4.15	4.68	3.52
LOC100505904	4.15	4.69	3.76
LINC00521	4.15	4.36	3.94
DLEU7	4.15	4.58	3.60
LOC400958	4.15	4.94	3.55
LRRC18	4.15	4.68	3.42
PPP2R2C	4.15	4.47	3.86
LOC100129223	4.15	4.52	3.78
MPP2	4.15	4.70	3.76
MLN	4.15	4.59	3.65
OTTHUMG00000086912	4.15	4.68	3.26
NPY2R	4.15	4.41	3.93
OTTHUMG00000020552	4.15	4.70	3.77
OTTHUMG00000017531	4.15	4.64	3.59
IDI2-AS1	4.15	4.52	3.64
ARRDC5	4.15	4.62	3.62
NETO2	4.15	4.69	3.65
AK7	4.15	4.67	3.90
SPATA31C2	4.15	4.61	3.85
GLYCAM1	4.15	4.46	3.36
ACOT6	4.15	4.60	3.78
GNRHR	4.15	4.70	3.52
SLC16A6	4.15	4.70	3.64
LOC100130705	4.15	4.61	3.61
OTTHUMG00000005710	4.15	4.64	3.73
OTTHUMG00000165727	4.15	4.58	3.52
AFAP1L2	4.15	4.70	3.75
MYBPHL	4.15	4.66	3.80
LHFPL3-AS2	4.15	4.66	3.73
TTLL13	4.14	4.46	3.86
OTTHUMG00000161606	4.14	4.77	3.74
SLC25A5-AS1	4.14	4.59	3.72
NPPA-AS1	4.14	4.64	3.50
DPEP3	4.14	4.63	3.62
FAM90A1	4.14	4.61	3.43
OTTHUMG00000171354	4.14	4.40	3.65
KIAA0319	4.14	4.53	3.62
PHACTR3	4.14	4.47	3.81
RRP7B	4.14	4.53	3.41

LOC401242	4.14	4.58	3.91
LOC100130992	4.14	4.63	3.72
CXCL1	4.14	4.79	3.77
AVPR1A	4.14	5.15	3.10
USP32P2	4.14	5.48	3.13
OTTHUMG00000024201	4.14	4.66	3.77
LOC100506127	4.14	4.38	3.58
MPO	4.14	4.77	3.69
OTTHUMG00000151248	4.14	4.39	3.75
TRIM43B	4.14	5.14	3.46
NEUROD6	4.14	4.53	3.73
GTSE1	4.14	4.71	3.76
HAR1A	4.14	4.43	3.72
MAD2L1	4.14	4.72	3.57
WNT9B	4.14	4.36	3.86
WDR93	4.14	4.43	3.62
IGHV7-81	4.14	4.72	3.50
STYK1	4.14	4.57	3.77
CCKAR	4.14	4.58	3.54
MAP4K1	4.14	4.31	3.92
PPP1R17	4.14	4.83	3.14
MIR603	4.14	4.70	3.55
MIR545	4.14	4.94	3.56
EMR4P	4.14	4.73	3.57
CYP1A2	4.14	4.58	3.74
NOD2	4.14	4.41	3.98
OTTHUMG00000015714	4.14	5.11	3.09
SLC22A7	4.14	4.38	3.89
OTTHUMG00000163333	4.14	4.56	3.65
C22orf42	4.14	4.72	3.63
ZNF665	4.14	4.59	3.89
FGF9	4.14	4.49	3.74
CIB3	4.14	4.87	3.60
SARM1	4.14	4.55	3.87
ZNF878	4.14	4.92	3.81
FTHL17	4.14	4.47	3.21
LRRC48	4.14	4.42	3.84
EPYC	4.14	4.92	3.61
OTTHUMG00000012409	4.14	5.00	3.68
STK16	4.14	4.58	3.16
TXNDC9	4.14	4.52	3.50
DEFB115	4.14	4.57	3.81
ZNF563	4.14	4.58	3.62
FOXN3-AS1	4.14	4.46	3.84
ELMOD1	4.14	4.32	3.91
OR14I1	4.14	4.56	3.81
KRBOX1-AS1	4.14	4.49	3.77
TACR1	4.14	4.82	3.75
RBMS3-AS1	4.14	4.59	3.92

KRT8P11	4.14	4.87	3.51
CDC25A	4.14	4.52	3.77
DPH6-AS1	4.14	4.44	3.71
HS3ST2	4.14	4.43	3.73
KRTAP8-1	4.14	4.42	3.47
SERAC1	4.14	4.40	3.75
ZNF26	4.14	4.42	3.62
SOCS1	4.14	4.66	3.58
IQCF5	4.14	4.37	3.85
LAMC2	4.13	4.53	3.94
TMEM154	4.13	4.63	3.63
OTTHUMG00000035264	4.13	4.55	3.53
TPRXL	4.13	4.55	3.74
PARP10	4.13	4.68	3.61
SLC45A1	4.13	4.46	3.67
GRIN2A	4.13	4.49	3.79
SNORA75	4.13	5.11	3.10
XKR6	4.13	4.57	3.63
SYNPO2L	4.13	4.57	3.51
CENPVP1	4.13	4.49	3.90
BEX2	4.13	4.93	3.37
ANKRD53	4.13	4.50	3.92
LOC440900	4.13	4.83	3.69
PIP5K1A	4.13	5.07	3.38
HTR1F	4.13	4.75	3.62
TSPAN5	4.13	4.44	3.76
SPAG11B	4.13	4.76	3.67
GLRA4	4.13	4.32	3.82
DGAT2L6	4.13	4.32	3.83
OTTHUMG00000150041	4.13	4.74	3.70
FLJ36777	4.13	4.59	3.55
TMEM143	4.13	4.42	3.55
SPRR2F	4.13	5.04	2.87
OTTHUMG00000167160	4.13	4.96	3.79
ATP1A4	4.13	4.79	3.80
OTTHUMG00000167183	4.13	4.69	3.69
PCDH19	4.13	4.33	3.83
HTR6	4.13	4.49	3.90
OTTHUMG00000018782	4.13	4.35	3.81
NME5	4.13	4.41	3.61
FLJ42220	4.13	4.39	3.59
LOC388942	4.13	4.76	3.67
OR51F1	4.13	4.54	3.30
CDH4	4.13	4.53	3.84
MTVR2	4.13	4.59	3.46
C6orf201	4.13	4.37	3.80
RPL34-AS1	4.13	4.82	3.76
IHH	4.13	4.57	3.49
ZNF319	4.13	4.42	3.80

DLX6-AS2	4.13	4.42	3.86
ZNF664	4.13	4.51	3.68
PLXNB3	4.13	4.40	3.84
RGS9BP	4.13	4.66	3.75
IL7R	4.13	4.47	3.50
ASB2	4.13	4.61	3.77
DPYD-AS1	4.13	4.73	3.89
LINC00207	4.13	4.69	3.72
PENK	4.13	4.55	3.73
IL21R	4.13	4.61	3.83
OR7E156P	4.13	4.60	3.47
ZNF30	4.13	4.45	3.60
HMGCLL1	4.13	4.51	3.78
HAO2-IT1	4.13	5.07	3.67
SLC22A5	4.13	4.49	3.76
LOC340340	4.13	5.09	3.65
CHRNA5	4.13	4.58	3.73
KRT7	4.13	4.47	3.64
RWDD2A	4.13	4.43	3.49
HSP90AB2P	4.13	4.55	3.75
SNORD78	4.13	4.86	2.39
OTTHUMG00000166562	4.13	4.41	3.87
MAGEA10	4.13	4.83	3.75
C3orf22	4.13	4.34	3.92
LOC100289098	4.13	4.55	3.62
LOC100996634	4.13	4.55	3.60
LOC349160	4.13	4.85	3.49
LCE1E	4.13	4.68	3.30
OLIG1	4.13	4.39	3.77
OTTHUMG00000162042	4.13	4.87	3.45
AGBL3	4.13	4.54	3.83
LOC100505658	4.13	4.38	3.75
FAM133A	4.13	5.17	3.20
TICRR	4.13	4.59	3.64
TNFRSF19	4.13	4.53	3.77
OTTHUMG00000163189	4.13	4.54	3.64
SCGB1C1	4.12	4.79	3.52
LOC642236	4.12	4.72	3.16
CCDC19	4.12	4.40	3.61
LOC100506688	4.12	4.48	3.53
TK1	4.12	4.64	3.54
SIDT1	4.12	4.48	3.67
CCL4	4.12	4.60	3.57
OTTHUMG00000016828	4.12	4.42	3.67
RTP1	4.12	4.46	3.83
LOC100129858	4.12	4.81	3.57
LOC728431	4.12	4.56	3.57
BCAS4	4.12	4.73	3.57
OTTHUMG00000153468	4.12	4.70	3.76

CMPK2	4.12	4.79	3.51
LOC340113	4.12	4.56	3.46
OTTHUMG00000167549	4.12	4.66	3.75
OR3A3	4.12	4.76	3.60
INHBC	4.12	4.49	3.72
MGC16275	4.12	4.49	3.64
CCDC163P	4.12	5.07	3.64
TCTE1	4.12	4.61	3.68
DEFB119	4.12	4.80	3.77
ZNF860	4.12	4.53	3.75
RNA5SP309	4.12	5.01	3.37
NBLA00301	4.12	4.43	3.61
GPRC5D	4.12	4.71	3.71
LOC100507554	4.12	4.64	3.67
QPRT	4.12	4.69	3.35
S100P	4.12	4.34	3.86
HERC5	4.12	5.10	3.49
ABCD1	4.12	4.32	3.87
ACTN3	4.12	4.85	3.74
LINC00372	4.12	4.79	3.57
S100A5	4.12	4.63	3.71
ACRV1	4.12	4.38	3.86
PRKCG	4.12	4.44	3.69
PTPRT	4.12	4.61	3.54
KDM4D	4.12	4.46	3.48
CLVS1	4.12	4.36	3.84
ERVMER34-1	4.12	4.63	3.58
ZNF578	4.12	5.31	3.38
C5orf63	4.12	4.46	3.79
CD22	4.12	4.39	3.86
SLC37A1	4.12	4.71	3.64
FAM86B3P	4.12	4.49	3.54
LOC100505853	4.12	4.86	3.42
ANKRD55	4.12	4.53	3.63
PGLYRP4	4.12	4.52	3.54
ARAP1-AS2	4.12	4.55	3.54
CYP1B1-AS1	4.12	4.65	3.55
C10orf129	4.12	4.64	3.81
LOC100507384	4.12	4.55	3.54
ANKRD6	4.12	4.34	3.73
CYB561	4.12	4.60	3.79
ASB12	4.12	4.65	3.67
LOC116437	4.12	4.53	3.77
TSPAN10	4.12	4.66	3.54
CHAC2	4.12	4.64	3.65
NAP1L6	4.12	4.98	3.49
F2RL1	4.12	4.69	3.88
DSCR10	4.12	4.46	3.84
FAHD2B	4.11	4.53	3.40

C20orf85	4.11	4.34	3.79
OTTHUMG00000164347	4.11	4.67	3.68
EPHA5	4.11	4.39	3.71
CYTIP	4.11	5.19	3.70
TINCR	4.11	4.93	3.24
CSTL1	4.11	4.51	3.87
UNC5D	4.11	4.54	3.82
C3orf49	4.11	4.53	3.77
XKR5	4.11	4.56	3.65
MIR4778	4.11	4.81	3.40
FAM66B	4.11	4.86	3.31
BIRC5	4.11	4.43	3.55
SMIM6	4.11	4.49	3.54
AOC4	4.11	4.97	3.51
OTTHUMG00000154794	4.11	4.55	3.68
WTAPP1	4.11	5.17	3.37
MAST4-AS1	4.11	4.67	3.52
CDH15	4.11	4.71	3.76
FAM65B	4.11	4.45	3.86
REPS2	4.11	5.00	3.26
OTTHUMG00000014290	4.11	4.49	3.90
FIGNL2	4.11	4.69	3.81
TAL2	4.11	4.86	3.77
TRAV2	4.11	4.62	3.65
C12orf60	4.11	4.60	3.86
OVOL1	4.11	4.53	3.43
LOC283387	4.11	4.96	3.46
LINC00633	4.11	4.97	3.27
H2BFXP	4.11	4.75	3.27
C17orf58	4.11	4.70	3.71
MDS2	4.11	4.56	3.66
UNC5B-AS1	4.11	4.83	3.67
CT62	4.11	4.51	3.73
FUT7	4.11	4.55	3.78
ZNF32-AS3	4.11	4.49	3.67
LOC284009	4.11	4.56	3.47
IL7	4.11	4.53	3.24
SLC9A7P1	4.11	4.87	3.21
RIPK4	4.11	4.41	3.75
ANKRD34C	4.11	4.55	3.53
CAPN9	4.11	4.46	3.73
MIR140	4.11	5.16	3.30
NDC80	4.11	4.97	3.47
POU3F1	4.11	4.54	3.62
CELF4	4.11	4.62	3.57
FOXQ1	4.11	4.63	3.78
TUBB1	4.11	4.68	3.46
SEZ6L	4.11	4.75	3.84
OTTHUMG00000169825	4.11	4.88	3.36

CNTN4-AS1	4.11	4.64	3.62
TRPM6	4.11	4.35	3.72
NLRP8	4.11	4.55	3.74
LOC388906	4.11	4.62	3.70
ACCSL	4.11	4.63	3.78
BEST3	4.11	4.34	3.85
OTTHUMG00000157190	4.11	4.48	3.82
SULT1C3	4.11	4.44	3.70
EYA4	4.11	4.68	3.56
RHAG	4.11	4.34	3.66
OTTHUMG00000164157	4.11	4.72	3.59
SLC25A15	4.11	4.58	3.37
MYO7B	4.11	4.52	3.79
GLT1D1	4.11	4.46	3.80
FOXC2	4.11	4.50	3.82
CYP4X1	4.11	5.03	3.27
ABCB1	4.10	4.64	3.59
PPP1R14C	4.10	5.38	3.25
LOC93622	4.10	4.54	3.54
CCDC175	4.10	4.40	3.70
TAS2R41	4.10	4.76	3.86
OTTHUMG00000150809	4.10	4.45	3.78
NRG2	4.10	4.59	3.86
OTTHUMG00000002945	4.10	5.25	2.66
OR6P1	4.10	4.86	3.29
FCRLB	4.10	4.56	3.67
DRGX	4.10	4.39	3.83
CCDC27	4.10	4.43	3.77
PPARA	4.10	4.48	3.95
CD300LB	4.10	4.65	3.58
PITPNM3	4.10	4.38	3.67
PRSS46	4.10	4.66	3.68
OTTHUMG00000163062	4.10	4.63	3.57
AWAT1	4.10	4.55	3.62
FLJ27243	4.10	4.51	3.51
LOC100506465	4.10	4.54	3.74
OTTHUMG00000167377	4.10	4.56	3.75
RPRM	4.10	4.51	3.73
OTTHUMG00000017897	4.10	4.77	3.73
CRYBB1	4.10	4.43	3.64
HDX	4.10	4.40	3.71
RUVBL1-AS1	4.10	4.80	3.56
OTTHUMG00000014230	4.10	4.51	3.58
ITGB1BP2	4.10	4.23	3.88
OTTHUMG00000156062	4.10	4.39	3.61
GRM3	4.10	4.45	3.81
RNF138P1	4.10	4.50	3.62
OTTHUMG00000170826	4.10	4.62	3.77
TMEM144	4.10	4.57	3.56

OTTHUMG00000165747	4.10	4.61	3.52
TXLNB	4.10	4.69	3.49
NLRP7	4.10	4.39	3.72
ZIC4-AS1	4.10	5.00	3.59
C20orf96	4.10	4.40	3.53
SPATS1	4.10	4.43	3.71
OTTHUMG00000154727	4.10	4.58	3.60
FIGN	4.10	4.56	3.55
PDZK1	4.10	4.73	3.33
LINC00534	4.10	4.74	3.67
SLC16A9	4.10	4.38	3.81
WDR4	4.10	4.45	3.56
LOC84931	4.10	4.41	3.87
TEX41	4.10	4.54	3.79
LOC339166	4.10	4.51	3.72
PGLYRP2	4.10	4.52	3.75
PDHA2	4.10	4.65	3.65
PLA2G4E	4.10	4.55	3.66
TRIP13	4.10	4.34	3.91
ERVV-1	4.10	4.61	3.76
XKR4	4.10	4.62	3.61
LOC341056	4.10	4.66	3.68
SEMA4D	4.10	4.38	3.71
MTHFD2L	4.10	4.70	3.67
ISPD	4.10	4.63	3.74
YPEL5	4.10	4.43	3.65
OTTHUMG00000164413	4.10	4.97	3.70
OTTHUMG00000018022	4.10	4.57	3.63
KRTAP15-1	4.10	5.02	3.32
DYRK3	4.10	4.80	3.59
STX1B	4.10	4.52	3.77
ANKRD18CP	4.10	4.50	3.68
CCDC166	4.10	4.41	3.91
LOC340178	4.10	5.22	3.46
OTTHUMG00000167947	4.10	4.40	3.82
GRHL2	4.10	4.47	3.80
LOC100134259	4.10	4.88	3.41
LOC100505774	4.10	5.01	3.38
LINC00284	4.10	4.85	2.96
EHHADH-AS1	4.10	4.52	3.66
ZNF833P	4.10	4.65	3.55
TFF1	4.10	4.53	3.57
LOC731223	4.09	4.49	3.61
LHX9	4.09	4.48	3.71
PNCK	4.09	4.47	3.65
ZDHHC23	4.09	4.57	3.78
OTTHUMG00000155311	4.09	4.50	3.26
ABLIM2	4.09	4.68	3.77
LRRC3B	4.09	4.43	3.80

LOC400128	4.09	4.33	3.84
OTTHUMG00000000412	4.09	4.87	3.68
OTTHUMG00000165950	4.09	4.80	3.57
OTTHUMG00000019258	4.09	4.71	3.64
LOC149134	4.09	4.53	3.48
LOC101060244	4.09	4.50	3.50
OTTHUMG00000032152	4.09	4.68	3.57
ASCL4	4.09	4.37	3.75
C15orf60	4.09	4.26	3.74
FAM5B	4.09	4.41	3.73
PRSS27	4.09	4.50	3.54
OTTHUMG00000156146	4.09	4.51	3.78
KDM4B	4.09	4.62	3.43
IGFBPL1	4.09	4.45	3.68
NME9	4.09	4.50	3.76
C3orf72	4.09	4.47	3.86
SIRPD	4.09	4.47	3.60
DLK1	4.09	4.46	3.70
FAM19A5	4.09	4.39	3.61
DUSP4	4.09	4.59	3.51
OTTHUMG00000037358	4.09	4.43	3.67
PRKXP1	4.09	4.65	3.33
MYPN	4.09	4.46	3.68
LINC00501	4.09	4.75	3.23
POP1	4.09	4.37	3.73
MUSK	4.09	4.26	3.87
OR13A1	4.09	4.81	3.76
FOXI3	4.09	4.39	3.66
TNFAIP8	4.09	4.56	3.76
SLC46A2	4.09	4.68	3.82
BCL2A1	4.09	4.40	3.79
CD3D	4.09	4.96	3.33
SEL1L2	4.09	4.45	3.75
OTTHUMG00000017337	4.09	4.45	3.58
ITGB5-AS1	4.09	4.85	2.82
HIF1A-AS1	4.09	4.43	3.81
FSHB	4.09	4.66	3.73
LOC255167	4.09	4.56	3.83
OTTHUMG00000000565	4.09	4.35	3.43
MIR362	4.09	4.60	3.56
OTTHUMG00000171984	4.09	4.63	3.19
MORC1	4.09	4.66	3.11
LINC00114	4.09	4.28	3.78
ARRDC3-AS1	4.09	4.31	3.86
CFL1	4.09	4.36	3.73
OTTHUMG00000172491	4.09	4.45	3.37
OTTHUMG00000164862	4.09	4.55	3.71
C19orf81	4.09	4.47	3.32
OTTHUMG00000168813	4.09	4.75	3.36

HEY1	4.09	4.75	3.00
NOX5	4.09	4.32	3.91
RNASEH2B-AS1	4.09	4.58	3.69
OTTHUMG00000171471	4.09	4.52	3.53
THAP7-AS1	4.09	4.83	3.77
FAM170B-AS1	4.09	4.55	3.72
EFCAB10	4.09	4.60	3.67
FBN2	4.09	4.58	3.81
LINC00620	4.08	4.61	3.21
DNAH7	4.08	4.34	3.78
MIR500A	4.08	4.54	3.43
OTTHUMG00000155178	4.08	4.82	3.60
CAPN8	4.08	4.33	3.66
OTTHUMG00000165902	4.08	4.93	3.23
GABRB2	4.08	4.45	3.67
GSX1	4.08	4.38	3.83
FA2H	4.08	4.35	3.69
OTTHUMG00000171829	4.08	5.20	3.23
LOC646268	4.08	4.73	3.54
OTX1	4.08	4.33	3.88
LOC100996425	4.08	4.50	3.65
OR5B12	4.08	4.44	3.80
CDC42EP3	4.08	4.79	3.73
TSPAN1	4.08	4.72	3.18
OTTHUMG00000021104	4.08	4.82	3.33
OR1A1	4.08	4.77	3.61
MIR195	4.08	4.68	3.59
SHANK2-AS2	4.08	4.54	3.72
RAPGEFL1	4.08	4.29	3.66
FAM187B	4.08	4.67	3.69
TLX3	4.08	4.52	3.76
OLR1	4.08	5.85	3.65
MRPL23	4.08	4.44	3.72
OTTHUMG00000166356	4.08	4.53	3.47
MIR4436A	4.08	4.94	3.22
OTTHUMG00000162001	4.08	5.00	3.40
PTTG1	4.08	4.68	3.71
LINC00210	4.08	4.38	3.52
SNORA74A	4.08	4.52	3.74
CCL3	4.08	4.38	3.43
C12orf39	4.08	4.48	3.70
LOC284930	4.08	4.63	3.54
DNAH6	4.08	4.43	3.71
MGC16025	4.08	4.44	3.22
LOC441204	4.08	4.48	3.77
PAGE1	4.08	4.43	3.64
OTTHUMG00000074319	4.08	4.67	3.77
ACSBG2	4.08	4.69	3.49
TMEM150C	4.08	4.33	3.61

TFAP2A	4.08	4.35	3.77
LINC00943	4.08	4.62	3.72
OTTHUMG00000165840	4.08	4.50	3.69
CCL17	4.08	4.78	3.66
MAP7D2	4.08	4.35	3.66
RBM47	4.08	4.74	3.68
AANAT	4.08	4.36	3.56
CLEC4C	4.08	4.40	3.55
LOC101060717	4.08	4.50	3.70
OTTHUMG00000161607	4.08	4.52	3.83
APOL3	4.08	4.45	3.57
NALCN-AS1	4.08	4.72	3.34
OTTHUMG00000020352	4.08	4.83	3.68
MS4A12	4.08	4.42	3.82
LOC100131060	4.07	4.42	3.71
FAM71F2	4.07	4.39	3.87
CROT	4.07	4.52	3.68
MIR4458	4.07	4.33	3.86
RAD51	4.07	4.51	3.51
OTTHUMG00000003326	4.07	4.54	3.57
PIN4P1	4.07	4.49	3.66
FOXG1	4.07	4.38	3.52
BOLL	4.07	4.26	3.80
RAB40C	4.07	4.37	3.80
LOC100506473	4.07	4.41	3.79
OTTHUMG00000179151	4.07	4.41	3.35
TUBA3FP	4.07	4.48	3.45
HIST1H2AM	4.07	5.47	2.59
LOC219690	4.07	4.27	3.74
PTPN20A	4.07	4.79	3.40
PRG3	4.07	4.33	3.72
ALOX15B	4.07	4.42	3.83
TMEM254	4.07	4.76	3.57
CDK15	4.07	4.72	3.62
SLC25A33	4.07	4.48	3.78
OTTHUMG00000046176	4.07	4.45	3.77
TTTY21	4.07	4.60	3.57
TMCO2	4.07	4.44	3.70
HLA-DOB	4.07	4.51	3.74
FBXL19-AS1	4.07	4.36	3.49
TRIM73	4.07	4.35	3.73
LOC392364	4.07	4.91	2.83
TMEM217	4.07	4.82	3.59
CEACAM16	4.07	4.81	3.01
ENOX1-AS1	4.07	4.68	3.67
RRM2B	4.07	4.78	3.21
OTTHUMG00000154787	4.07	4.42	3.51
OTTHUMG00000169332	4.07	4.67	3.26
HOXA6	4.07	4.50	3.28

KIAA1804	4.07	4.78	3.44
ABCA4	4.07	4.45	3.66
PPP1R26	4.07	4.57	3.55
DPH2	4.07	4.25	3.82
OTTHUMG00000018923	4.07	4.57	3.60
PRSS54	4.07	4.44	3.66
FOXI1	4.07	4.64	3.69
OR51E2	4.07	4.47	3.56
DLL4	4.07	4.74	3.54
PCDHB13	4.07	5.20	2.78
IL36B	4.07	4.62	3.57
S100A7A	4.07	4.25	3.82
OTTHUMG00000150003	4.07	4.51	3.75
OTTHUMG00000017626	4.07	4.68	3.64
OTTHUMG00000017334	4.07	4.72	3.87
IGFN1	4.07	4.50	3.82
TIMM44	4.07	4.43	3.28
LOC100130264	4.07	4.45	3.82
XKRX	4.07	4.66	3.72
ACVR2B-AS1	4.07	4.39	3.79
OTTHUMG00000168899	4.07	4.60	3.50
LOC101060038	4.07	4.60	3.61
OTTHUMG00000020009	4.07	4.92	3.40
LOC339666	4.07	4.33	3.68
CACNA1G	4.07	4.56	3.63
OTTHUMG00000159760	4.07	4.31	3.83
OTTHUMG00000168744	4.07	4.45	3.57
SIGLEC8	4.07	4.45	3.54
HOXA-AS3	4.07	4.55	3.64
OTTHUMG00000041137	4.07	4.36	3.84
SYDE2	4.07	4.64	3.62
ANO1-AS2	4.07	4.52	3.43
MGC15885	4.06	4.45	3.66
IYD	4.06	4.26	3.60
IGLV3-32	4.06	4.58	3.75
OR10K1	4.06	4.69	3.57
CNNM1	4.06	4.46	3.69
CNBD2	4.06	4.68	3.61
LINC00479	4.06	4.69	3.73
OTTHUMG00000037400	4.06	4.78	3.47
SOWAHD	4.06	4.57	3.75
CRHBP	4.06	4.81	3.51
PKP2	4.06	4.44	3.73
FAM183B	4.06	4.33	3.69
OTTHUMG00000169893	4.06	4.50	3.66
DPP6	4.06	4.57	3.64
LCE3C	4.06	4.50	3.59
SNORA70D	4.06	4.52	3.48
SGPP2	4.06	4.31	3.81

LRRC4C	4.06	4.61	3.56
GRIN2B	4.06	4.47	3.65
ACER1	4.06	4.44	3.66
GRK7	4.06	4.75	3.58
GPR143	4.06	4.48	3.75
LOC100506791	4.06	4.45	3.59
DCAF12L1	4.06	4.53	3.66
SDS	4.06	4.68	3.58
EFCAB8	4.06	4.66	3.81
GIPC2	4.06	4.57	3.41
CNGA3	4.06	4.34	3.80
SLC25A25	4.06	4.44	3.55
RGS7BP	4.06	4.70	3.60
HAO2	4.06	4.70	3.52
LTB	4.06	5.28	3.53
SLC18A1	4.06	4.27	3.71
L1CAM	4.06	4.47	3.64
OTUD6A	4.06	4.39	3.76
SPATA31D5P	4.06	4.75	3.34
SLC15A1	4.06	4.64	3.56
TXNDC16	4.06	4.70	3.65
FIBCD1	4.06	4.35	3.64
TSSC1	4.06	4.33	3.61
OTTHUMG00000151793	4.06	4.27	3.82
MPV17L	4.06	4.60	3.21
CBLN1	4.06	4.35	3.79
LRRC55	4.06	4.65	3.58
ANKK1	4.06	4.33	3.68
SLC5A4	4.06	4.33	3.76
GVINP1	4.06	4.53	3.47
CA5A	4.06	4.58	3.44
CEACAM18	4.06	4.48	3.88
FCER2	4.06	4.67	3.73
OTTHUMG00000163993	4.06	4.92	2.97
DNER	4.06	4.59	3.61
RNF182	4.06	4.80	3.68
OR4D9	4.06	4.71	3.53
NXT2	4.06	4.36	3.61
OTTHUMG00000171503	4.06	4.59	3.76
LOC283710	4.06	4.68	3.65
C12orf71	4.06	4.67	3.65
CYP4F30P	4.06	5.21	3.40
LINC00299	4.06	4.66	3.71
UPK3B	4.06	4.62	3.77
FMO6P	4.06	4.67	3.68
ABCG5	4.06	4.38	3.76
EID2	4.06	4.54	3.69
LOC283403	4.06	4.63	3.55
OTTHUMG00000160281	4.06	4.98	3.40

DEFB105B	4.06	4.37	3.54
MUCL1	4.06	4.37	3.69
MED18	4.06	4.72	3.68
SPATC1L	4.06	4.40	3.41
MIR4446	4.06	4.45	3.71
LOC100507150	4.06	4.47	3.80
ARHGAP26-IT1	4.06	4.79	3.25
LCT	4.06	4.43	3.68
PP13004	4.06	4.33	3.41
LCN8	4.06	4.72	3.66
TFDP3	4.06	4.66	3.68
MASP1	4.05	4.46	3.50
EN1	4.05	4.41	3.63
VSTM5	4.05	4.72	3.75
ZNF488	4.05	4.52	3.74
MIR204	4.05	5.11	3.42
KIAA1656	4.05	4.64	3.64
TTTY17A	4.05	4.83	3.28
CDIPT-AS1	4.05	4.48	3.73
C11orf16	4.05	4.49	3.38
OTTHUMG00000153300	4.05	4.57	3.35
NR6A1	4.05	4.56	3.15
TFAP2D	4.05	4.48	3.58
MAG	4.05	4.76	3.41
SLC17A2	4.05	4.44	3.53
LINC00707	4.05	4.41	3.54
DUSP9	4.05	4.76	3.57
EDN2	4.05	4.37	3.54
ESYT3	4.05	4.25	3.59
RNA5SP520	4.05	4.79	3.47
AP1S2	4.05	4.79	3.48
LOC149950	4.05	4.56	3.68
OTTHUMG00000020544	4.05	4.37	3.63
LOC100288524	4.05	4.62	3.50
OTTHUMG00000154542	4.05	4.54	3.66
YIPF7	4.05	4.29	3.73
OTTHUMG00000020885	4.05	4.58	3.36
OTTHUMG00000164480	4.05	4.47	3.43
NKX2-2-AS1	4.05	4.41	3.69
OTTHUMG00000177663	4.05	4.54	3.26
LOC100130348	4.05	4.46	3.75
LOC729815	4.05	5.10	3.24
OTTHUMG00000166836	4.05	4.89	3.27
TNR	4.05	4.34	3.69
MSTN	4.05	5.54	3.19
CPA4	4.05	4.31	3.70
LOC100506178	4.05	4.50	3.65
LINC00320	4.05	4.65	3.50
PIN4	4.05	4.49	3.59

METTL7B	4.05	4.42	3.32
RTP2	4.05	4.46	3.52
LOC100128822	4.05	4.63	3.61
ZNF815P	4.05	4.52	3.70
ENPP3	4.05	4.35	3.63
OTTHUMG00000169978	4.05	4.59	3.73
OTTHUMG00000169468	4.05	4.61	3.62
HOXB13	4.05	4.52	3.48
KLK15	4.05	4.79	3.74
OTTHUMG00000015931	4.05	4.84	3.79
DPYD-AS2	4.05	4.50	3.89
OTTHUMG00000163754	4.05	4.72	3.20
EID2B	4.05	4.50	3.74
GBP1P1	4.05	4.62	3.81
DEFB113	4.05	4.53	3.64
SNAP25	4.05	4.99	3.28
OXCT2	4.05	4.44	3.64
OR10H1	4.05	4.79	3.57
SSTR1	4.05	4.44	3.74
LOC389906	4.05	4.64	3.57
WNT8B	4.05	4.33	3.73
MIR1537	4.05	5.04	3.43
EGFEM1P	4.05	4.72	3.38
EPS8L1	4.05	4.25	3.77
FMO3	4.05	4.40	3.55
OTTHUMG00000168913	4.05	4.40	3.57
PPY2	4.05	4.40	3.57
NLRP13	4.04	4.45	3.74
VWA5B1	4.04	4.43	3.85
C5orf46	4.04	4.36	3.68
LOC100506253	4.04	4.32	3.56
EML6	4.04	4.39	3.69
PIWIL3	4.04	4.35	3.73
OTTHUMG00000168583	4.04	4.61	3.25
LMCD1-AS1	4.04	4.44	3.58
LOC100133683	4.04	4.31	3.52
PLEKHO1	4.04	4.62	3.49
OTTHUMG00000159705	4.04	4.55	3.57
LOC100506766	4.04	4.49	3.55
NDN	4.04	4.27	3.60
LOC100505735	4.04	4.51	3.69
OTTHUMG00000177876	4.04	4.83	3.28
OTTHUMG00000152762	4.04	4.55	3.39
OTTHUMG00000173224	4.04	4.53	3.34
DNAJC12	4.04	4.46	3.51
LINC00548	4.04	4.49	3.62
LEMD1-AS1	4.04	4.44	3.88
LOC100505666	4.04	4.59	3.62
ADIPOQ-AS1	4.04	4.72	3.05

DPPA4	4.04	4.69	3.56
CYSLTR1	4.04	5.10	2.93
DNAH2	4.04	4.43	3.70
LINC00359	4.04	4.62	3.24
LIPC	4.04	4.27	3.74
GCNT7	4.04	4.36	3.71
C8orf49	4.04	4.56	3.67
SPATA8-AS1	4.04	4.72	3.46
GABRP	4.04	4.35	3.57
SNRPF	4.04	4.39	3.57
XRCC6BP1	4.04	4.30	3.67
OR2S2	4.04	4.45	3.42
MC5R	4.04	4.68	3.61
TMEM75	4.04	4.30	3.69
FRAT1	4.04	4.68	3.61
SRL	4.04	4.76	3.39
OLFM3	4.04	4.63	3.58
ZSCAN12P1	4.04	4.46	3.77
LOC100996663	4.04	4.50	3.46
SDR16C5	4.04	4.54	3.62
OTTHUMG00000153105	4.04	4.69	3.41
CD247	4.04	4.34	3.74
RASAL1	4.04	4.72	3.30
OTTHUMG00000172611	4.04	4.84	3.62
LOC729867	4.04	4.58	3.64
SLFN14	4.04	4.44	3.57
UNC80	4.04	4.34	3.67
OTTHUMG00000183047	4.04	4.52	3.42
SRD5A1	4.04	4.48	3.33
GIMD1	4.04	4.45	3.67
LINC00838	4.04	4.67	3.83
MIR7-2	4.04	4.81	3.41
LOC643733	4.04	5.19	2.83
OTTHUMG00000009500	4.04	5.22	2.52
TRIM63	4.04	4.46	3.72
SLC45A4	4.04	4.45	3.66
SMG8	4.04	4.41	3.60
SLC22A2	4.04	4.37	3.76
FAM47A	4.04	4.46	3.49
NEK10	4.03	4.21	3.81
RFESD	4.03	4.74	3.26
LOC100505918	4.03	4.73	3.69
IL36RN	4.03	4.49	3.58
OSBPL7	4.03	4.36	3.66
APOE	4.03	4.80	3.37
NPAS1	4.03	4.39	3.61
OOEP	4.03	4.52	3.69
DNAJA4	4.03	4.33	3.59
FLJ32154	4.03	4.39	3.55

SAMD5	4.03	4.35	3.31
TLX1	4.03	4.40	3.50
SPTBN2	4.03	4.30	3.82
OTTHUMG00000153180	4.03	4.54	3.65
LOC100505921	4.03	4.47	3.51
OTTHUMG00000032013	4.03	4.64	3.48
MACROD2-AS1	4.03	4.62	3.56
ARHGEF38-IT1	4.03	4.61	3.50
NPB	4.03	4.45	3.69
CLDN4	4.03	4.32	3.59
AMH	4.03	4.23	3.80
LOC100132146	4.03	4.29	3.54
CST9	4.03	4.26	3.70
MIR4253	4.03	5.05	2.85
MIR4435-1	4.03	4.50	3.64
CYP4F22	4.03	4.77	3.46
FAM71A	4.03	4.43	3.54
MIR374B	4.03	4.93	2.94
RAB6C	4.03	4.73	3.26
C10orf35	4.03	4.25	3.41
GIMAP7	4.03	4.73	3.26
LOXHD1	4.03	4.60	3.59
AIM2	4.03	4.56	3.72
OTTHUMG00000019502	4.03	4.56	3.70
TEX14	4.03	4.33	3.71
OGDHL	4.03	4.52	3.68
HIVEP3	4.03	4.62	3.38
OTTHUMG00000167521	4.03	4.60	3.50
LOC100288814	4.03	4.44	3.60
LOC100291105	4.03	4.42	3.76
C3orf65	4.03	4.77	3.49
JPH3	4.03	4.33	3.71
AOX1	4.03	4.49	3.46
FLJ41130	4.03	4.51	3.69
NAP1L3	4.03	4.44	3.48
FAM72C	4.03	4.46	3.59
IL1RL1	4.03	4.37	3.80
OTTHUMG00000165649	4.03	4.46	3.65
ZNF274	4.03	4.47	3.53
ZNF225	4.03	4.64	3.18
ALS2CR11	4.03	4.51	3.41
OPALIN	4.03	4.53	3.77
C14orf164	4.03	4.34	3.63
OTTHUMG00000168443	4.03	4.85	3.43
MBLAC1	4.03	4.39	3.75
OR2B11	4.03	4.74	3.31
ARGFXP2	4.03	4.48	3.66
LINC00445	4.03	4.56	3.80
FBXO36	4.03	4.45	3.62

KCNQ3	4.03	4.42	3.71
LOC150577	4.03	4.39	3.59
C8orf12	4.03	4.41	3.81
STARD8	4.03	4.78	3.07
CACNG2	4.03	4.82	3.40
FAM83G	4.03	4.51	3.71
GPR17	4.03	4.37	3.62
OTTHUMG00000164738	4.03	4.46	3.42
OTTHUMG00000152908	4.03	4.72	3.42
LOC100128905	4.03	4.88	3.64
C8orf17	4.03	4.24	3.85
TRAV13-1	4.03	4.44	3.48
THAP10	4.03	4.32	3.77
CDKN2B	4.03	4.37	3.58
HOTTIP	4.02	4.41	3.18
PKD1L3	4.02	4.53	3.51
MSANTD1	4.02	4.45	3.30
ANO5	4.02	4.94	3.39
CBLN4	4.02	4.45	3.63
OTTHUMG00000046223	4.02	4.66	3.21
HRK	4.02	4.32	3.61
LOC339788	4.02	4.51	3.61
PDSS1	4.02	4.46	3.46
LOC100505676	4.02	4.39	3.47
GAD2	4.02	4.48	3.71
MAP2	4.02	4.72	3.49
PNLIPRP1	4.02	4.45	3.51
TBC1D21	4.02	4.48	3.58
LOC100507053	4.02	4.52	3.70
FLJ45671	4.02	4.76	3.69
TEKT2	4.02	4.32	3.82
OTTHUMG00000152930	4.02	4.51	3.66
SNORD116-27	4.02	4.94	2.99
KRT5	4.02	4.21	3.75
OTTHUMG00000159958	4.02	4.77	3.56
SORCS1	4.02	4.60	3.62
LCNL1	4.02	4.29	3.69
NOTUM	4.02	4.28	3.76
FAM155B	4.02	4.32	3.47
CAMSAP3	4.02	4.35	3.72
OLFML2A	4.02	4.82	2.84
OTTHUMG00000156193	4.02	4.87	3.59
C22orf24	4.02	4.58	3.79
OTTHUMG00000150859	4.02	4.34	3.75
LINC00536	4.02	4.52	3.64
ZNF782	4.02	4.26	3.73
LOC100507199	4.02	4.33	3.61
RNU6-82P	4.02	4.79	3.03
OTTHUMG00000161345	4.02	4.67	3.61

FAM3D	4.02	4.34	3.76
LOC284551	4.02	4.26	3.73
OTTHUMG00000019702	4.02	4.51	3.74
METTL24	4.02	4.36	3.67
HIST1H1D	4.02	4.42	3.72
PWWP2B	4.02	4.75	3.55
OAZ3	4.02	4.46	3.30
BTD	4.02	4.49	3.71
LINC00336	4.02	4.71	3.44
HOXB9	4.02	4.41	3.59
KIF9	4.02	4.40	3.59
PZP	4.02	4.58	3.71
ZNF284	4.02	4.94	3.24
SYT6	4.02	4.37	3.78
OTTHUMG00000041219	4.02	4.59	3.47
LOC646241	4.02	4.52	3.50
TRAJ36	4.02	5.06	3.52
NXPE4	4.02	4.38	3.40
LOC100129636	4.02	4.43	3.76
NPAS4	4.02	4.74	3.44
ZNF195	4.02	4.28	3.60
C17orf77	4.02	4.61	3.66
CDHR3	4.02	4.19	3.89
GPR160	4.02	4.39	3.79
PAWR	4.02	4.46	3.63
OTTHUMG00000152523	4.01	4.87	3.24
NTNG1	4.01	4.44	3.71
AMIGO2	4.01	4.55	3.57
FAS-AS1	4.01	4.55	3.54
C11orf82	4.01	4.39	3.70
GABRQ	4.01	4.27	3.71
SLC3A1	4.01	4.83	3.64
LOC51145	4.01	4.64	3.55
OTTHUMG00000161392	4.01	4.44	3.38
LOC100132055	4.01	4.25	3.70
OTTHUMG00000169088	4.01	4.71	3.54
OTTHUMG00000159367	4.01	4.71	3.23
NETO1	4.01	4.51	3.69
DCLRE1A	4.01	4.45	3.70
LOH12CR2	4.01	4.24	3.63
ABO	4.01	5.04	3.32
LOC100506923	4.01	4.30	3.28
ZNF396	4.01	4.46	3.42
PRDM9	4.01	4.79	3.29
TEX37	4.01	4.37	3.61
FGD5P1	4.01	4.25	3.44
OTTHUMG00000163748	4.01	4.52	3.71
LOC285696	4.01	4.19	3.72
LOC100130169	4.01	4.66	3.62

EPDR1	4.01	4.40	3.37
OTTHUMG00000015498	4.01	4.45	3.64
TTC25	4.01	4.36	3.71
POTEG	4.01	4.53	3.66
OTTHUMG00000150375	4.01	4.29	3.68
RNASE3	4.01	4.83	3.15
GCA	4.01	4.43	3.81
TCL1A	4.01	4.54	3.55
NEDD9	4.01	4.32	3.73
LINC00494	4.01	4.59	3.66
ZYG11A	4.01	4.41	3.47
LOC100507160	4.01	4.40	3.58
CCDC181	4.01	4.41	3.66
SGCG	4.01	4.72	3.55
CCDC147	4.01	4.29	3.83
LOC100506083	4.01	4.55	3.63
DIAPH3	4.01	4.97	3.43
OR8H2	4.01	5.55	3.02
OTTHUMG00000183949	4.01	4.28	3.65
EPX	4.01	4.39	3.61
LPP-AS1	4.01	4.56	3.57
DNASE2B	4.01	4.41	3.63
OR51E1	4.01	4.66	3.06
FOXF2	4.01	4.70	3.48
STIL	4.01	4.38	3.80
DKFZp434L192	4.01	4.53	3.68
OTTHUMG00000151740	4.01	4.32	3.38
LINC00661	4.01	4.30	3.45
THOC7-AS1	4.01	4.14	3.78
NOTO	4.01	4.38	3.62
ADAMTS9-AS1	4.01	4.47	3.65
GLT6D1	4.01	4.51	3.58
OTTHUMG00000168872	4.01	4.44	3.47
NPTXR	4.00	4.45	3.45
RGS8	4.00	4.44	3.61
ZNF57	4.00	4.45	3.50
OTTHUMG00000170258	4.00	4.53	3.52
PRINS	4.00	4.67	3.64
LINC00313	4.00	4.43	3.21
LOC100506373	4.00	4.34	3.60
CCK	4.00	4.35	3.47
LOC100507140	4.00	4.48	3.24
WFDC8	4.00	4.54	3.48
LOC100130238	4.00	4.29	3.59
C1orf222	4.00	4.42	3.69
TTTY7	4.00	4.50	3.55
GSG1	4.00	4.60	3.66
SPDYE4	4.00	4.41	3.69
GP9	4.00	4.26	3.61

FHL5	4.00	5.83	2.84
OTTHUMG00000021953	4.00	4.75	3.32
SNORD116-10	4.00	4.75	3.36
ZNF778	4.00	4.36	3.70
STC1	4.00	4.22	3.63
GNA14	4.00	4.29	3.75
CRISP3	4.00	4.49	3.49
ZFY	4.00	4.51	3.31
DGCR5	4.00	4.40	3.63
ADCY8	4.00	4.38	3.76
PRSS42	4.00	4.30	3.80
LINC00167	4.00	4.33	3.75
LOC100131496	4.00	4.45	3.59
ANKRD30BP2	4.00	5.58	3.38
DBC1	4.00	4.52	3.52
MIR487B	4.00	5.51	2.79
OTTHUMG00000163488	4.00	4.56	3.35
SLC7A3	4.00	4.92	3.50
OTTHUMG00000156402	4.00	4.81	3.62
TPO	4.00	4.51	3.81
ZNF702P	4.00	4.59	3.44
OTTHUMG00000153209	4.00	4.68	3.45
OR1G1	4.00	4.83	3.58
FAM111B	4.00	4.50	3.23
CATSPER1	4.00	4.55	3.43
MIR129-1	4.00	4.67	2.91
OTTHUMG00000164043	4.00	4.39	3.50
CNDP1	4.00	4.50	3.44
LINC00311	4.00	4.42	3.71
KCTD19	4.00	4.23	3.63
OTTHUMG00000171341	4.00	4.49	3.45
LOC147646	4.00	4.34	3.55
MYF5	4.00	4.55	3.52
NLGN4Y	4.00	4.88	3.21
SFTA3	4.00	4.36	3.53
BCAR4	4.00	4.49	3.57
BSN	4.00	4.21	3.78
ZKSCAN5	4.00	4.23	3.69
OTTHUMG00000014842	4.00	4.57	3.60
OTTHUMG00000031781	4.00	4.65	3.28
ISX	3.99	4.38	3.35
SNORD116-12	3.99	4.54	3.24
RNFT2	3.99	4.52	3.64
LOC100129520	3.99	4.44	3.48
ESPL1	3.99	4.25	3.56
RBMXL3	3.99	4.32	3.46
LOC100507431	3.99	4.37	3.60
CYP8B1	3.99	4.35	3.64
PTPN20B	3.99	4.78	3.33

SERPINA11	3.99	4.41	3.49
MLLT10P1	3.99	4.33	3.64
POLR1B	3.99	4.26	3.50
AGMO	3.99	4.20	3.48
OTTHUMG00000162519	3.99	4.51	3.24
NDUFAF1	3.99	4.41	3.59
CT60	3.99	4.48	3.63
KIAA0895	3.99	4.26	3.81
OTTHUMG00000171387	3.99	4.39	3.45
PGA3	3.99	4.75	2.86
GCKR	3.99	4.35	3.72
OTTHUMG00000020335	3.99	4.67	2.82
MARK1	3.99	4.30	3.66
ODF4	3.99	4.65	3.57
LOC100093698	3.99	4.37	3.35
LOC100129029	3.99	4.31	3.65
SLA2	3.99	4.77	3.44
CCNYL1	3.99	4.76	2.84
TIGD3	3.99	4.40	3.60
AMBN	3.99	4.47	3.64
VN1R2	3.99	4.73	3.62
BTN1A1	3.99	4.53	3.72
TEX13B	3.99	4.41	3.73
LOC100130451	3.99	4.56	3.20
MKRN7P	3.99	4.96	3.10
MIR138-2	3.99	4.39	3.46
TMPRSS7	3.99	4.23	3.73
OTTHUMG00000164075	3.99	4.79	3.18
SUN5	3.99	4.50	3.32
PRSS37	3.99	4.52	3.45
MIR3939	3.99	4.63	3.59
CHML	3.99	5.00	3.26
LOC389273	3.99	4.30	3.70
MIR1972-1	3.99	4.45	3.49
FAM86C1	3.99	4.44	3.42
LINC00440	3.99	4.65	3.15
OTTHUMG00000004135	3.99	4.62	3.13
KCNIP1	3.99	4.43	3.63
FLJ27255	3.99	4.57	3.39
TM4SF19-AS1	3.99	4.61	3.64
ZCWPW2	3.99	4.77	3.54
MYEOV	3.99	4.28	3.69
TMPRSS2	3.99	4.47	3.56
RGS22	3.99	4.38	3.57
LOC100506606	3.99	4.38	3.58
PDE12	3.99	4.51	3.06
LINC00239	3.99	4.23	3.72
TMOD4	3.99	4.54	3.46
NCBP2-AS1	3.99	4.15	3.72

OTTHUMG00000015079	3.99	4.43	3.68
OTTHUMG00000164263	3.99	4.60	3.47
PCDHB3	3.98	4.34	3.42
TBC1D4-AS1	3.98	4.75	3.35
OTTHUMG00000019939	3.98	4.65	3.26
OR11L1	3.98	4.37	3.53
LINC00525	3.98	4.51	3.62
AKAP5	3.98	4.41	3.72
ATP8B3	3.98	4.40	3.59
KRT72	3.98	4.43	2.97
LOC644050	3.98	4.40	3.61
SESN2	3.98	4.39	3.73
RNF122	3.98	4.50	3.51
OTTHUMG00000164837	3.98	4.88	3.39
TSPAN33	3.98	4.33	3.69
TRAJ17	3.98	4.33	3.02
KRTAP20-4	3.98	4.42	2.91
OTTHUMG00000150429	3.98	4.59	3.51
OTTHUMG00000159413	3.98	4.37	3.53
SLC39A2	3.98	4.62	3.48
CYP26A1	3.98	4.49	3.27
LOC389043	3.98	4.73	3.19
DHRS7C	3.98	4.65	3.33
GBA3	3.98	4.66	3.43
KCNJ6-IT1	3.98	5.08	3.38
OTTHUMG00000180232	3.98	4.68	3.37
LINC00339	3.98	4.37	3.63
OTTHUMG00000151592	3.98	4.28	3.68
COL19A1	3.98	4.29	3.70
GPC6-AS2	3.98	4.64	3.47
TMIGD1	3.98	4.58	3.40
KLRB1	3.98	4.29	3.54
TEDDM1	3.98	4.31	3.61
EGFL6	3.98	4.65	3.64
ZNF750	3.98	4.22	3.72
TBATA	3.98	4.22	3.67
OTTHUMG00000169349	3.98	4.39	3.76
ZNF268	3.98	4.53	3.49
LINC00594	3.98	4.46	3.41
CDCP2	3.98	4.35	3.46
WT1	3.98	4.35	3.63
C4orf36	3.98	4.46	3.65
OTTHUMG00000017910	3.98	5.01	3.31
RGAG1	3.98	4.46	3.77
VWDE	3.98	4.30	3.27
FLJ41941	3.98	4.48	3.56
KCTD6	3.98	4.53	3.54
OTTHUMG00000162302	3.98	4.54	3.33
B3GNT3	3.98	4.38	3.61

GCSAM	3.98	4.42	3.71
TDRD10	3.98	4.36	3.66
FLJ33581	3.98	4.52	3.42
C17orf102	3.98	4.40	3.63
OTTHUMG00000162672	3.98	4.35	3.56
OTTHUMG00000171168	3.98	4.60	3.53
OTTHUMG00000018645	3.98	4.54	3.38
MYOM2	3.98	4.24	3.77
ZC3H12B	3.98	4.66	3.35
FAM71B	3.98	4.72	3.47
MGAT5B	3.98	4.39	3.71
CACNG4	3.98	4.39	3.41
POM121L2	3.98	4.39	3.41
PHKA1-AS1	3.98	4.34	3.69
MRPL40	3.98	4.29	3.68
GREB1	3.98	4.44	3.78
CLPSL1	3.98	4.38	3.75
OTTHUMG00000020205	3.98	4.38	3.40
OTTHUMG00000167224	3.98	4.47	2.93
OTTHUMG00000182079	3.98	4.34	3.63
OTTHUMG00000177747	3.98	4.23	3.53
OTTHUMG00000163707	3.98	4.35	3.51
SIRT4	3.98	4.30	3.66
GOLGA6L7P	3.98	5.44	2.68
ZFP57	3.98	4.31	3.72
LINC00439	3.98	4.71	3.23
LINC00511	3.98	4.64	3.38
LOC440292	3.98	4.40	3.44
TMEM74	3.98	4.36	3.15
OTTHUMG00000152596	3.98	4.71	3.21
OTTHUMG00000151596	3.98	4.42	3.48
C16orf78	3.98	4.55	3.69
RPL36P20	3.98	4.73	2.66
OTTHUMG00000159111	3.98	4.38	3.59
BCAS1	3.98	4.36	3.51
NF1P2	3.98	4.45	2.87
LOC100128560	3.98	4.28	3.58
LOC100996342	3.98	4.20	3.55
DLX6-AS1	3.98	4.38	3.68
LOC100130954	3.98	4.53	3.38
FLJ37035	3.98	4.68	3.63
MIR3685	3.98	4.70	2.71
OTTHUMG00000159625	3.97	4.42	3.43
DCSTAMP	3.97	4.45	3.47
OTTHUMG00000132645	3.97	4.38	3.56
MIR1324	3.97	4.38	3.62
TCF24	3.97	4.28	3.52
RNASE11	3.97	4.51	3.42
OTTHUMG00000137386	3.97	4.25	3.79

TRIL	3.97	4.13	3.70
FAM26F	3.97	4.69	3.04
SPRED3	3.97	4.20	3.72
SLC10A2	3.97	4.42	3.54
ZFPM2	3.97	4.36	3.57
CEBPE	3.97	4.23	3.79
OTTHUMG00000170224	3.97	4.41	3.54
LOC100131132	3.97	4.13	3.80
FAM66A	3.97	4.85	3.06
LINC00092	3.97	4.52	3.63
LINC00112	3.97	4.59	3.40
FOXR1	3.97	4.47	3.30
LOC389023	3.97	4.48	3.51
LOC100506411	3.97	4.41	3.45
LGALS12	3.97	4.74	3.63
S100A7	3.97	4.47	3.39
COMMD6	3.97	4.31	3.49
TRPV1	3.97	4.26	3.63
AMZ2P1	3.97	4.51	3.46
TAS2R31	3.97	4.64	2.40
OTTHUMG00000019965	3.97	5.18	3.10
OTTHUMG00000161731	3.97	4.65	3.24
FUNDC2	3.97	4.22	3.73
CCDC42	3.97	4.50	3.52
LINC00174	3.97	4.55	3.64
PCYT1B	3.97	4.29	3.62
DUPD1	3.97	4.36	3.54
TDRD9	3.97	4.55	3.61
LOC400456	3.97	4.40	3.48
MMRN1	3.97	4.76	3.17
LOC285441	3.97	4.38	3.54
LCE2D	3.97	4.53	3.30
NKX2-2	3.97	4.52	3.54
LOC100506271	3.97	4.37	3.47
RPS6KA6	3.97	4.56	3.07
C8orf37	3.97	4.43	3.46
OR14C36	3.97	4.46	3.48
LINC00909	3.97	4.28	3.55
RNA5SP395	3.97	4.78	3.20
MIR431	3.97	4.47	3.65
MIR4295	3.97	5.13	2.96
SPATA9	3.97	4.41	3.32
TGM5	3.97	4.35	3.71
C16orf93	3.97	4.36	3.10
OR1S1	3.97	4.80	2.81
TRHDE-AS1	3.97	4.77	3.09
OTUD7A	3.97	4.32	3.32
OTTHUMG00000151942	3.97	4.34	3.47
SPACA3	3.97	4.39	3.60

LOC100652758	3.97	4.33	3.50
YPLR6490	3.97	4.41	3.53
POU4F2	3.97	4.24	3.74
TRAV40	3.97	4.69	3.34
CLEC2L	3.97	4.18	3.59
LINC00211	3.97	4.28	3.50
KCND2	3.97	4.41	3.61
GPR55	3.97	4.64	3.64
FLJ90680	3.97	4.61	3.38
NEK2	3.97	4.59	3.42
MIR511-1	3.97	4.89	3.41
CCAT1	3.97	4.31	3.63
RNU4ATAC2P	3.97	4.50	3.50
HIST1H3E	3.97	5.29	2.67
MIR188	3.97	4.57	3.28
OTTHUMG00000018238	3.97	4.39	3.44
ELOVL7	3.97	4.28	3.57
OTTHUMG00000158502	3.97	4.37	3.49
REC8	3.97	4.28	3.65
MIR4704	3.96	4.62	3.18
LOC100499183	3.96	4.57	3.66
LPPR3	3.96	4.62	3.21
RSPH10B2	3.96	4.41	3.38
GPR33	3.96	4.60	3.53
LINC00102	3.96	4.41	3.27
NKAPP1	3.96	4.44	3.66
OTTHUMG00000154733	3.96	4.40	3.48
OTTHUMG00000166136	3.96	4.57	3.67
USP49	3.96	4.48	3.62
RAD51AP1	3.96	4.51	3.73
ABCB5	3.96	4.80	3.57
ELAVL3	3.96	4.45	3.35
BPIFC	3.96	4.40	3.71
LOC643085	3.96	4.33	3.64
COLEC10	3.96	4.21	3.69
OTTHUMG00000162626	3.96	4.57	3.28
EID3	3.96	4.29	3.49
ACTN1-AS1	3.96	4.35	3.47
UPB1	3.96	4.20	3.32
HLX	3.96	4.18	3.63
CA1	3.96	4.32	3.55
ACOXL	3.96	4.26	3.53
OTTHUMG00000066368	3.96	4.54	3.46
LOC100128374	3.96	4.34	3.67
PACRG	3.96	4.33	3.40
LOC100133032	3.96	4.41	3.42
ZNF169	3.96	4.22	3.66
TSG1	3.96	4.41	3.64
GPR78	3.96	4.53	3.37

TLDC2	3.96	4.35	3.35
ZC3H12D	3.96	4.26	3.37
SKINTL	3.96	4.68	3.70
OTTHUMG00000156732	3.96	4.49	3.56
ROPN1L	3.96	4.40	3.66
OTTHUMG00000168414	3.96	4.39	3.45
PP12708	3.96	4.38	3.40
APOB	3.96	4.63	3.50
PPP2R3B	3.96	4.18	3.80
LINC00371	3.96	4.28	3.51
SPTB	3.96	4.29	3.67
LOC100506048	3.96	4.64	3.55
LOC284577	3.96	4.53	3.40
LOC150568	3.96	4.28	3.63
OTTHUMG00000152844	3.96	4.24	3.53
MIR634	3.96	4.99	2.99
KRT77	3.96	4.80	3.32
CLDN1	3.96	4.31	3.40
SPINK4	3.96	4.32	3.34
LOC100652911	3.96	4.25	3.64
OTTHUMG00000153489	3.96	4.19	3.65
RADIL	3.96	4.36	3.60
TRGV2	3.95	4.65	3.28
SLC36A3	3.95	4.32	3.67
HHATL-AS1	3.95	4.42	3.48
OTTHUMG00000153644	3.95	4.46	3.37
KRTAP25-1	3.95	4.56	3.62
NRG4	3.95	4.46	3.63
OTTHUMG00000041351	3.95	4.44	3.51
OTTHUMG00000150930	3.95	4.20	3.57
LOC100505613	3.95	4.76	2.85
IL1A	3.95	4.25	3.43
GRHL3	3.95	4.57	3.48
ALYREF	3.95	4.66	3.47
FLJ38576	3.95	4.58	3.27
SDPR	3.95	4.59	3.13
LOC730139	3.95	4.32	3.59
C14orf28	3.95	4.29	3.50
SCML4	3.95	4.29	3.53
TMEM177	3.95	4.16	3.68
RNU7-9P	3.95	5.65	3.41
NKX6-3	3.95	4.48	3.46
PRAM1	3.95	4.77	3.41
APOO	3.95	5.10	2.47
LOC100507077	3.95	4.60	3.62
TPTE2	3.95	4.24	3.58
C17orf78	3.95	4.38	3.61
ESR1	3.95	4.37	3.36
NUDT11	3.95	4.95	2.22

DCAF8L1	3.95	4.37	3.52
ZNF572	3.95	4.33	3.60
MPST	3.95	4.59	3.64
OTTHUMG00000165330	3.95	4.13	3.61
PRSS55	3.95	4.36	3.47
UG0898H09	3.95	4.15	3.61
MYLK3	3.95	4.28	3.55
OTTHUMG00000164964	3.95	4.45	3.31
AMER2	3.95	4.41	3.55
LOC100507501	3.95	4.78	3.48
NIPAL4	3.95	4.43	3.48
OTTHUMG00000151813	3.95	4.44	3.23
LRRTM3	3.95	4.39	3.40
MIR16-2	3.95	4.62	3.32
OTTHUMG00000163668	3.95	4.43	3.62
IGKV1-6	3.95	4.70	2.93
FAM173B	3.95	4.15	3.49
OTTHUMG00000171591	3.95	4.53	3.22
LOC730668	3.95	4.50	3.28
C6orf52	3.95	4.45	3.17
ACTL6B	3.95	4.39	3.43
CLDN6	3.95	4.33	3.66
GPC3	3.95	4.20	3.72
OTTHUMG00000020213	3.95	4.41	3.37
OR51M1	3.95	4.32	3.52
OTTHUMG00000170975	3.95	4.28	3.53
ABCA13	3.95	4.35	3.67
MOGAT1	3.95	4.49	3.66
OTTHUMG00000161851	3.95	4.68	3.42
IDI2	3.95	4.34	3.67
MIR885	3.95	4.24	3.58
FAM71F1	3.94	4.27	3.63
LOC286177	3.94	4.46	3.29
ZNF667	3.94	4.64	3.40
GRIN3A	3.94	4.30	3.48
SLC25A41	3.94	4.73	3.13
OTTHUMG00000008128	3.94	4.38	3.50
TTLL9	3.94	4.30	3.60
SCN2B	3.94	4.56	3.44
LOC283588	3.94	4.35	3.39
OTTHUMG00000171478	3.94	4.83	3.05
LOC100128593	3.94	4.32	3.62
SIGLECL1	3.94	4.39	3.39
LRP2	3.94	4.19	3.69
MC2R	3.94	4.44	3.05
DEFA5	3.94	4.54	3.29
INTS4L1	3.94	4.80	3.22
LOC286009	3.94	4.19	3.55
DAZ1	3.94	4.58	3.35

OTTHUMG00000018345	3.94	4.72	3.33
FCER1A	3.94	5.56	3.34
MFSD2B	3.94	4.21	3.57
FBXO40	3.94	4.66	3.37
LINC00959	3.94	4.17	3.76
LOC100506393	3.94	4.17	3.52
LOC100293612	3.94	4.23	3.74
DKK1	3.94	5.47	2.40
TMEM191A	3.94	4.95	3.45
FAM219B	3.94	4.18	3.78
TRAV30	3.94	4.39	3.43
SERPINA4	3.94	4.39	3.56
OTTHUMG00000151460	3.94	4.45	3.00
DTNA	3.94	4.32	3.50
STK33	3.94	4.35	3.69
OTTHUMG00000017736	3.94	4.59	3.25
C7	3.94	5.16	2.76
PRM1	3.94	4.99	3.26
LOC100996404	3.94	4.26	3.42
MIR1197	3.94	5.64	3.38
MSL3P1	3.94	4.46	3.34
AP4B1-AS1	3.94	4.52	3.11
LILRA5	3.94	4.35	3.36
SVOP	3.94	4.62	3.62
C20orf201	3.94	4.56	3.30
PCDH7	3.94	4.46	3.54
FLJ30375	3.94	4.47	3.43
WDR17	3.94	4.24	3.58
LOC285692	3.94	4.41	3.40
C19orf40	3.94	4.27	3.67
TAF7L	3.94	4.52	3.63
LOC100147773	3.94	4.24	3.47
OTTHUMG00000167159	3.94	4.23	3.37
RNF180	3.94	4.37	3.51
HAR1B	3.94	4.42	3.32
KCND3-AS1	3.94	4.49	3.52
LOC100240735	3.94	4.80	3.22
C11orf65	3.93	4.16	3.71
TMEM17	3.93	4.53	3.54
SAMD3	3.93	4.32	3.59
OTTHUMG00000009791	3.93	4.52	3.13
HTR1D	3.93	4.45	3.60
UBAC2-AS1	3.93	4.36	3.53
OTTHUMG00000030382	3.93	4.39	3.53
L3MBTL4	3.93	4.50	3.29
LOC727808	3.93	4.25	3.69
DMTN	3.93	4.35	3.53
AZFP	3.93	4.39	3.74
MST1R	3.93	4.22	3.50

LOC100188947	3.93	4.57	3.51
OTTHUMG00000150610	3.93	5.06	3.20
LOC100505768	3.93	4.39	3.22
SLC24A4	3.93	4.23	3.73
LYPD6B	3.93	4.15	3.53
NLRP4	3.93	4.27	3.66
C9orf53	3.93	4.29	3.57
OTTHUMG00000014196	3.93	4.72	3.57
LOC100132339	3.93	4.34	3.52
NXF2B	3.93	4.28	3.51
CASP1	3.93	4.54	3.47
MMP20	3.93	4.43	3.50
LRRC25	3.93	4.57	3.51
SRY	3.93	5.41	2.37
PPP5D1	3.93	4.45	3.41
MAGEB18	3.93	4.18	3.62
MIR1236	3.93	4.49	3.68
LOC100130642	3.93	4.44	3.28
TMEM229A	3.93	4.23	3.49
CDKL2	3.93	4.52	3.55
OTTHUMG00000178198	3.93	4.46	3.39
OTTHUMG00000171462	3.93	4.28	3.54
DIAPH3-AS2	3.93	4.77	3.47
ZNF487P	3.93	4.59	3.39
RIPPLY3	3.93	4.41	3.42
SLC25A21-AS1	3.93	4.35	3.47
KHDC3L	3.93	4.37	3.73
OTTHUMG00000166470	3.93	4.33	3.54
LOC100505989	3.93	4.63	3.29
LRRC33	3.93	4.29	3.45
C2orf61	3.93	4.48	3.34
LOC100499194	3.93	4.40	3.59
BCL2L14	3.93	4.45	3.71
GDPGP1	3.93	4.22	3.44
MT1DP	3.93	4.26	3.38
OTTHUMG00000160511	3.93	4.57	3.56
CLEC9A	3.93	4.86	3.26
GK3P	3.93	4.80	3.42
AURKA	3.93	4.38	3.45
NLRP2	3.93	4.36	3.63
FAM124A	3.93	4.38	3.63
PLA2G7	3.93	4.43	3.57
LOC100130899	3.93	4.36	3.42
CYP2J2	3.93	4.37	3.26
CHGA	3.93	4.63	3.37
KCNJ10	3.93	4.58	3.35
LOC100505625	3.93	4.39	3.56
TMPRSS13	3.93	4.61	3.53
WDR88	3.93	4.65	3.39

C4orf40	3.93	4.27	3.55
METAP1D	3.93	4.31	3.55
OR6C74	3.93	4.94	3.37
ZBP1	3.93	4.40	3.64
PLS1	3.93	4.31	3.46
PM20D1	3.93	4.44	3.30
CHRM3	3.93	4.48	3.61
CCDC147-AS1	3.93	4.50	3.34
MIR4480	3.93	4.44	3.45
KLHL13	3.93	4.48	3.25
AZU1	3.92	4.25	3.64
ZNF366	3.92	4.56	3.31
SSUH2	3.92	4.40	3.53
PROX1	3.92	4.29	3.55
TDRD12	3.92	4.27	3.73
TM7SF2	3.92	4.23	3.54
RRH	3.92	4.40	3.43
SLC35F1	3.92	4.34	3.54
KRT20	3.92	4.25	3.37
OTTHUMG00000171464	3.92	4.35	3.60
HELLS	3.92	4.44	3.29
AGBL1	3.92	4.24	3.69
PSKH2	3.92	4.48	3.52
ST13P4	3.92	4.27	3.57
SPATA31D4	3.92	4.64	3.33
NHLRC1	3.92	4.33	3.59
SIGLEC12	3.92	4.19	3.76
OTTHUMG00000177234	3.92	4.33	3.19
OTTHUMG00000169333	3.92	4.26	3.46
OTTHUMG00000059447	3.92	4.42	3.27
SCML2	3.92	4.40	3.42
IL12RB1	3.92	4.24	3.36
STARD6	3.92	4.51	3.67
FSHR	3.92	4.28	3.48
HOXD9	3.92	4.30	3.61
HNF1B	3.92	4.49	3.58
PRSS30P	3.92	4.45	3.27
OTTHUMG00000152486	3.92	4.17	3.55
DPP3	3.92	4.13	3.62
GABRB1	3.92	4.56	3.34
ZNF677	3.92	4.13	3.67
CHST4	3.92	4.49	3.57
ABCB11	3.92	4.44	3.45
C21orf58	3.92	4.48	3.17
OTTHUMG00000163284	3.92	4.17	3.60
LOC729609	3.92	4.29	3.60
C10orf53	3.92	4.31	3.57
LOC100506085	3.92	4.48	3.52
FAM90A26P	3.92	4.39	3.44

LOC100289673	3.92	4.14	3.56
IL12RB2	3.92	5.29	3.18
WDR52-AS1	3.92	4.25	3.56
LOC100128830	3.92	4.47	3.66
XDH	3.92	4.57	3.52
FREM1	3.92	4.27	3.50
CCDC144NL	3.92	4.78	3.13
ST6GALNAC5	3.92	4.55	3.62
IL20RA	3.92	4.37	3.35
SCNN1B	3.92	4.34	3.42
CCL15-CCL14	3.92	4.98	2.90
LINC00564	3.91	4.45	3.49
LOC100505786	3.91	4.75	3.35
C12orf36	3.91	4.66	3.53
DEF6	3.91	4.22	3.78
HTR1A	3.91	4.33	3.20
OTTHUMG00000168473	3.91	4.22	3.51
ANGPT1	3.91	4.87	3.03
HNF4G	3.91	4.29	3.64
STEAP1B	3.91	4.28	3.52
ZNF587	3.91	4.51	3.28
FAM226B	3.91	4.46	3.37
NT5DC4	3.91	4.60	3.50
OTTHUMG00000074200	3.91	4.28	3.58
OTTHUMG00000163675	3.91	4.38	3.50
SERPINA7	3.91	4.26	3.41
FAM181A	3.91	4.23	3.58
C16orf47	3.91	4.33	3.59
KIAA1107	3.91	4.32	3.19
LILRP2	3.91	4.45	3.26
MEX3B	3.91	4.45	3.57
PKHD1	3.91	4.46	3.67
OTTHUMG00000151591	3.91	4.54	3.34
ZNF571	3.91	4.16	3.59
AIFM3	3.91	4.33	3.55
TRBV5-5	3.91	4.27	3.35
ART5	3.91	4.20	3.70
ZNF541	3.91	4.18	3.59
TPGS1	3.91	4.51	3.40
ZBED2	3.91	4.31	3.15
PLEKHS1	3.91	4.58	3.64
FGB	3.91	4.31	3.65
OTTHUMG00000179141	3.91	4.33	3.25
NBPF18P	3.91	4.62	3.34
OTTHUMG00000153146	3.91	4.67	3.36
PPP1R9A	3.91	4.31	3.23
C11orf71	3.91	4.51	3.52
CSMD1	3.91	4.27	3.66
GTF2E1	3.91	4.44	3.50

OTTHUMG00000018491	3.91	4.23	3.62
ARHGEF26-AS1	3.91	4.68	3.52
ZIC4	3.91	4.28	3.59
LOC100126784	3.91	4.22	3.60
TMEM95	3.91	4.57	3.66
OTTHUMG00000163330	3.91	4.17	3.72
OR6B1	3.91	4.20	3.65
GJA10	3.91	4.69	3.41
KCNG4	3.91	4.33	3.41
LOC100287808	3.91	4.18	3.65
C11orf44	3.91	4.45	3.55
NUP210	3.91	4.15	3.39
TRBV7-7	3.91	4.62	2.87
CD1E	3.91	4.54	3.35
HGC6.3	3.91	4.19	3.57
XKR9	3.91	4.17	3.60
IL1RN	3.91	4.62	3.52
OTTHUMG00000170885	3.91	4.71	3.35
TECTB	3.91	4.18	3.59
MYH13	3.90	4.47	3.59
OTTHUMG00000156038	3.90	4.25	3.47
FLJ21408	3.90	4.69	3.51
ZNF214	3.90	4.46	3.62
OTTHUMG00000171832	3.90	4.28	3.53
FAM228A	3.90	4.36	3.47
LPHN3	3.90	4.63	3.53
KIAA1644	3.90	4.85	3.52
OTTHUMG00000171178	3.90	4.60	3.33
TAGLN3	3.90	4.35	3.50
NRAP	3.90	4.37	3.54
RGS20	3.90	4.34	3.40
TMEM201	3.90	4.40	3.41
LUCAT1	3.90	4.28	3.59
LOC727982	3.90	4.31	3.53
ABCC2	3.90	4.47	3.55
LOC100131047	3.90	4.29	3.56
LOC93463	3.90	4.28	3.57
KRTAP3-2	3.90	4.55	3.39
BCL11B	3.90	4.37	3.57
HSPB9	3.90	4.35	3.55
SPATA2	3.90	4.22	3.36
LOC649133	3.90	4.61	3.51
ELOVL6	3.90	4.61	3.65
ZNF720	3.90	4.19	3.62
RPLP0P2	3.90	4.28	3.70
TSNAXIP1	3.90	4.15	3.57
C1orf101	3.90	4.32	3.56
GCLM	3.90	4.75	3.09
OTTHUMG00000036265	3.90	4.17	3.59

CD101	3.90	4.21	3.61
ZNF689	3.90	4.67	3.41
FRMD7	3.90	4.34	3.55
LOC339568	3.90	4.41	3.46
ALOX12B	3.90	4.17	3.52
MIR488	3.90	4.87	3.17
ALLC	3.90	4.57	3.52
IGKV2-40	3.90	4.29	3.63
CTSG	3.90	4.52	3.52
ZNF619	3.90	4.45	3.35
NAT1	3.90	4.11	3.52
PRSS12	3.90	4.34	3.39
TTTY1B	3.90	4.32	3.50
OTTHUMG00000164945	3.90	4.66	3.48
ZDHHC8P1	3.90	4.48	3.21
FGFBP1	3.90	4.22	3.36
EDAR	3.90	4.37	3.35
LOC348817	3.90	4.19	3.45
NHLRC4	3.90	4.30	3.47
RNA5SP374	3.90	4.92	3.23
POTEH	3.90	4.40	3.28
LINC00221	3.90	4.03	3.76
LACE1	3.90	4.23	3.57
FANCE	3.90	4.24	3.61
OTTHUMG00000155481	3.90	4.41	3.30
LPAR3	3.90	4.29	3.49
ZNF556	3.90	4.13	3.38
LINC00636	3.90	4.26	3.61
ATP6V0A4	3.90	4.39	3.29
ARR3	3.90	4.11	3.55
IFNL2	3.90	4.45	3.30
OTTHUMG00000162606	3.90	4.85	3.45
SNORA37	3.90	4.37	3.49
LOC100128840	3.90	4.32	3.45
PRLHR	3.89	4.40	3.57
SCGB2B2	3.89	4.30	3.49
TMEM8C	3.89	4.29	3.28
LOC101060103	3.89	4.59	3.25
NLRP12	3.89	4.34	3.48
MARS2	3.89	4.34	3.65
LOC100130539	3.89	4.42	3.38
LOC285972	3.89	4.51	3.35
LCN1	3.89	4.40	3.31
LOC100506025	3.89	4.01	3.68
RNA5SP152	3.89	4.44	3.22
OTTHUMG00000163124	3.89	4.39	3.24
OR9Q2	3.89	4.64	3.37
PRR5-ARHGAP8	3.89	4.29	3.56
DBF4	3.89	4.59	3.58

NUAK2	3.89	4.50	3.67
OTTHUMG00000132826	3.89	4.79	3.42
MIR521-2	3.89	4.85	3.14
SBK2	3.89	4.20	3.47
ZNF616	3.89	4.35	3.53
OTTHUMG00000164860	3.89	4.34	3.26
KCNK13	3.89	4.11	3.44
LOC728805	3.89	4.57	3.36
DUXA	3.89	4.20	3.57
OTTHUMG00000035690	3.89	4.61	3.39
ARHGEF37	3.89	4.24	3.53
CALB2	3.89	4.40	3.37
DMD-AS2	3.89	4.16	3.62
DEPDC7	3.89	4.44	3.52
CCDC61	3.89	4.64	3.48
HPD	3.89	4.30	3.58
FLJ30838	3.89	4.21	3.55
ITIH2	3.89	4.67	3.46
OTTHUMG00000160485	3.89	4.65	3.61
ZNF674	3.89	4.19	3.37
GFRA4	3.89	4.42	3.53
FLJ43879	3.89	4.39	3.29
MIR4474	3.89	4.28	3.59
NIPA1	3.89	4.96	2.59
MREG	3.89	4.04	3.68
THRB-AS1	3.89	4.68	3.29
TTTY2	3.89	4.84	3.22
SKP1	3.89	4.27	3.29
C2orf73	3.89	4.51	3.53
LYPD1	3.89	4.23	3.45
PRR23A	3.89	4.53	3.42
MIR3123	3.89	4.08	3.16
LINC00675	3.89	4.65	3.51
OTTHUMG00000162485	3.89	4.38	3.55
TMEM255A	3.89	4.62	3.38
LINC00849	3.89	4.69	2.78
OTTHUMG00000155987	3.89	4.15	3.60
RHOXF2	3.89	4.36	3.45
EGFR-AS1	3.89	4.56	3.44
SCN5A	3.89	4.16	3.53
LOC100292922	3.89	4.91	2.66
HOTAIR	3.89	4.24	3.36
MB	3.89	4.37	3.14
IGKV6-21	3.89	4.45	3.38
ELAVL4	3.89	4.27	3.43
DYRK4	3.89	4.09	3.70
OTTHUMG00000167401	3.89	4.33	3.53
OTTHUMG00000152568	3.89	4.24	3.27
EXO1	3.89	4.26	3.56

LOC100507435	3.89	4.44	3.37
OVCH1-AS1	3.89	4.45	3.41
RNA5SP483	3.88	4.65	2.86
LOC729574	3.88	4.31	3.55
FGF3	3.88	4.41	3.62
HTR3B	3.88	4.35	3.48
OTTHUMG00000161885	3.88	4.64	3.05
MAP3K15	3.88	4.14	3.32
CLC	3.88	4.95	3.32
IGF2BP1	3.88	4.22	3.54
TMEM174	3.88	4.33	3.45
C11orf53	3.88	4.17	3.69
SLC12A7	3.88	4.42	3.28
OR51J1	3.88	4.61	3.29
OTTHUMG00000166090	3.88	4.20	3.30
PAEP	3.88	4.41	3.29
HLA-DRB5	3.88	7.66	2.03
MIR581	3.88	4.51	2.97
OTTHUMG00000161875	3.88	4.41	3.18
CENPK	3.88	4.81	3.19
ARAP2	3.88	4.40	3.54
GJB1	3.88	4.09	3.70
WDR63	3.88	4.23	3.49
PARP15	3.88	4.67	3.50
OTTHUMG00000015667	3.88	4.32	3.52
GSTCD	3.88	4.45	3.49
A2MP1	3.88	4.48	3.43
MROH2A	3.88	4.20	3.62
THAP9	3.88	4.18	3.61
FLJ37453	3.88	4.41	3.05
ECT2L	3.88	4.11	3.62
LOC100509445	3.88	4.19	3.53
CENPI	3.88	4.74	3.20
NTRK3	3.88	4.39	3.59
ZNF391	3.88	4.20	3.44
LOC100507351	3.88	4.35	3.47
KNSTRN	3.88	4.24	3.53
FLJ45721	3.88	4.64	3.32
NMBR	3.88	4.26	3.60
AOX2P	3.88	4.28	3.42
GNMT	3.88	4.04	3.60
KCNA7	3.88	4.23	3.59
UCP3	3.88	4.13	3.61
SGOL1	3.88	4.45	3.55
SLITRK4	3.88	4.31	3.55
OTTHUMG00000171360	3.88	4.18	3.65
CD200R1	3.88	4.49	3.30
OR2A2	3.88	4.68	2.67
EIF1B-AS1	3.88	4.35	2.96

OTTHUMG00000008359	3.88	4.48	3.35
OTTHUMG00000176976	3.88	4.21	3.60
GBP6	3.88	4.76	3.34
OTTHUMG00000163615	3.88	4.35	3.44
IPCEF1	3.88	4.54	3.56
FRMD1	3.88	4.57	3.42
OTTHUMG00000168001	3.88	4.32	3.15
LOC100506351	3.88	4.46	3.22
MLLT4-AS1	3.88	4.20	3.36
ID4	3.88	4.84	3.31
ITGA8	3.88	4.38	3.52
LOC440337	3.88	4.29	2.99
SYNPO2	3.88	5.02	2.58
MFAP3L	3.88	4.43	3.07
LOC100996348	3.88	4.41	3.18
OTTHUMG00000031895	3.88	4.41	3.62
SPINK9	3.88	4.43	3.39
OTTHUMG00000171877	3.88	4.39	3.40
C11orf89	3.87	4.56	3.42
COCH	3.87	4.09	3.65
OTTHUMG00000177262	3.87	4.37	3.15
OTTHUMG00000168261	3.87	4.12	3.53
LOC100507267	3.87	4.09	3.64
GRB7	3.87	4.30	3.45
TNNI3	3.87	4.41	3.40
PRSS16	3.87	4.53	3.54
PLCXD3	3.87	4.23	3.58
RNA5SP441	3.87	4.65	2.97
TRIM7	3.87	4.22	3.26
GDPD4	3.87	4.46	3.23
FAM47B	3.87	4.30	3.27
PBK	3.87	5.11	2.95
OTTHUMG00000172597	3.87	4.54	3.22
RNA5SP460	3.87	4.20	3.35
TUSC7	3.87	4.05	3.70
OTTHUMG00000154835	3.87	4.25	3.23
TNP1	3.87	4.53	3.25
GNAT1	3.87	4.44	3.26
MEP1B	3.87	4.40	3.38
CELSR1	3.87	4.13	3.56
DBIL5P	3.87	4.20	3.38
COL6A4P2	3.87	4.23	3.45
GSDMC	3.87	4.23	3.46
ZSCAN16	3.87	4.80	3.35
AZGP1	3.87	4.72	3.15
FUT9	3.87	4.15	3.57
EPHA10	3.87	4.12	3.55
VSNL1	3.87	4.40	3.60
TAAR3	3.87	4.78	3.39

NKAIN2	3.87	4.46	3.25
NKAPL	3.87	4.03	3.59
CHRNA9	3.87	4.28	3.10
SMIM2-AS1	3.87	4.38	3.26
OTTHUMG00000158223	3.87	4.23	3.66
DEFB132	3.87	4.20	3.45
KRT6A	3.87	4.78	3.11
OTTHUMG00000037935	3.87	4.47	3.31
LINC00906	3.87	4.33	3.38
XCL1	3.87	4.46	3.30
SLC26A4-AS1	3.87	4.21	3.52
TMTC4	3.87	4.33	3.57
TTC40	3.87	4.23	3.60
ENO3	3.87	4.28	3.38
NLGN4X	3.87	4.18	3.38
MGC42157	3.87	4.22	3.39
MTL5	3.87	4.14	3.72
LOC401463	3.87	4.47	3.48
OR4M1	3.87	4.36	3.47
OTTHUMG00000165726	3.87	4.16	3.31
OR52J3	3.87	4.27	3.30
OR6C4	3.87	4.21	3.53
CABP1	3.87	4.48	3.49
OTTHUMG00000017564	3.87	4.11	3.55
NUDT4	3.87	4.34	3.55
KRTAP5-10	3.87	4.39	3.22
KCNJ11	3.87	4.31	3.40
CCR10	3.87	4.33	3.42
RNA5SP124	3.87	5.22	3.09
CLEC17A	3.87	4.34	3.21
MGC45800	3.87	4.17	3.64
KLF15	3.87	4.39	3.35
LOC100288181	3.87	4.13	3.29
RENBP	3.87	4.20	3.34
IGLV4-69	3.87	4.37	3.37
GLYCTK	3.87	4.10	3.67
LILRA1	3.87	4.25	3.43
TMC1	3.86	4.20	3.39
TFAP2C	3.86	4.20	3.42
OTTHUMG00000014691	3.86	4.39	3.17
LOC143188	3.86	4.33	3.32
GTSF1	3.86	4.59	3.49
MIR598	3.86	4.57	2.98
LOC100506172	3.86	4.22	3.53
OTTHUMG00000164197	3.86	4.55	3.15
CENPN	3.86	4.46	3.46
SLC38A4	3.86	4.65	2.88
FOXA1	3.86	4.29	3.45
CCNYL2	3.86	4.51	2.45

LINC00928	3.86	4.17	3.15
GPR111	3.86	4.54	3.28
RPSAP9	3.86	4.25	3.24
OTTHUMG00000171195	3.86	4.44	3.17
ACAA1	3.86	4.07	3.56
RNF157-AS1	3.86	4.39	3.51
DRP2	3.86	4.32	3.47
PTCHD2	3.86	4.20	3.50
CACNA1D	3.86	4.31	3.33
DNAH17	3.86	4.36	3.61
MCM10	3.86	4.22	3.48
LCE5A	3.86	4.94	3.36
ARSH	3.86	4.07	3.54
IL37	3.86	4.30	3.39
CEP19	3.86	4.37	3.34
OR51F2	3.86	4.79	2.97
DDX25	3.86	4.35	3.48
OTTHUMG00000171355	3.86	4.49	3.54
PLK5	3.86	4.48	3.46
C9orf171	3.86	4.12	3.60
MPP4	3.86	4.20	3.52
FRAS1	3.86	4.31	3.45
OTTHUMG00000158954	3.86	4.26	3.41
OTTHUMG00000015980	3.86	4.36	3.10
TSPAN9-IT1	3.86	4.55	3.47
CSN3	3.86	4.34	3.27
OTTHUMG00000156022	3.86	4.50	3.32
MAB21L1	3.86	4.16	3.52
LOC344887	3.86	4.31	3.10
CYP4F3	3.86	4.36	3.45
NYAP2	3.86	4.37	3.34
ASRGL1	3.86	4.23	3.53
PSTPIP2	3.86	4.29	3.30
OTTHUMG00000140109	3.86	4.38	3.14
OTTHUMG00000163905	3.86	4.67	3.16
LOC149351	3.86	4.25	3.46
CDH17	3.86	4.38	3.44
OTTHUMG00000162518	3.86	4.29	3.27
STPG1	3.86	4.34	3.44
TSPY2	3.86	5.11	2.22
SYT1	3.86	4.19	3.38
RNY4P4	3.86	4.61	3.11
POTEB2	3.86	4.46	3.22
TSSK6	3.86	4.08	3.55
LOC414300	3.86	4.49	3.42
RBMXL2	3.86	4.16	3.46
OTTHUMG00000021313	3.86	4.84	3.29
LOC100506422	3.86	4.40	3.58
LOC284344	3.85	4.64	3.39

OTTHUMG00000150956	3.85	4.30	3.28
P2RY1	3.85	4.40	3.45
GK	3.85	4.68	3.03
GSG2	3.85	4.44	3.40
LINC00861	3.85	4.43	3.40
MLF1	3.85	4.68	3.18
OTTHUMG00000154969	3.85	4.47	3.28
RGS1	3.85	4.90	2.94
MORN3	3.85	4.35	3.41
LINC00162	3.85	4.32	3.25
MYH6	3.85	4.18	3.54
PMP2	3.85	4.44	3.23
BANF2	3.85	4.39	3.48
LOC100287792	3.85	4.19	3.27
PRKAG2-AS1	3.85	4.46	3.36
DUSP5	3.85	4.30	3.43
PRSS41	3.85	4.37	3.24
OTTHUMG00000016489	3.85	4.57	2.52
OR10H3	3.85	4.71	3.37
ST6GALNAC1	3.85	4.66	3.42
SPATA31B1	3.85	4.38	3.37
OTTHUMG00000020070	3.85	4.25	3.45
OTTHUMG00000018994	3.85	4.35	3.50
MIR4804	3.85	4.21	3.42
HS3ST3B1	3.85	4.50	3.27
OTTHUMG00000014768	3.85	4.24	3.35
OTTHUMG00000162516	3.85	4.51	3.09
WBP2NL	3.85	4.06	3.54
MYLK-AS2	3.85	4.23	3.44
UBASH3A	3.85	4.19	3.49
LOC283854	3.85	4.31	3.54
OTTHUMG00000169663	3.85	4.75	3.22
PIGR	3.85	4.23	3.39
GRIN1	3.85	4.07	3.67
KCNH1	3.85	4.35	3.42
CAMKK1	3.85	4.23	3.48
DCAF13	3.85	4.70	3.09
DBIL5P2	3.85	4.36	3.49
FOXR2	3.85	4.66	3.42
ZNF429	3.85	4.48	3.17
MIR181A2HG	3.85	4.37	3.04
WDR66	3.85	4.25	3.41
OTTHUMG00000171321	3.85	4.17	3.51
LOC284561	3.85	4.25	3.17
MIR193A	3.85	4.45	3.14
OTTHUMG00000153094	3.85	4.11	3.46
HIGD1C	3.85	4.10	3.62
IL21R-AS1	3.85	4.32	3.35
OTTHUMG00000032077	3.85	4.32	3.25

OTTHUMG00000151726	3.85	4.72	3.15
LEF1-AS1	3.85	4.25	3.38
TUBA3C	3.85	4.55	3.11
LHFPL3-AS1	3.85	4.15	3.54
NMRK2	3.85	4.20	3.38
UBE2QL1	3.85	4.32	3.43
RNU7-2P	3.85	4.74	2.84
LOC100506476	3.85	4.49	3.12
ZNF549	3.85	4.31	3.17
MRVI1-AS1	3.85	4.38	3.27
KCNAB3	3.85	4.30	3.38
C12orf42	3.85	4.16	3.64
CLEC4E	3.85	4.27	3.19
CYCS	3.85	4.59	3.42
LOC730811	3.85	4.21	3.54
EPM2A	3.84	4.10	3.46
FMO2	3.84	4.88	2.83
PTH2R	3.84	4.62	3.47
OSM	3.84	4.08	3.44
SNRPD2P2	3.84	4.25	3.62
NEFH	3.84	4.22	3.54
ZBTB42	3.84	4.36	3.59
TMEM121	3.84	4.64	3.29
SLAMF1	3.84	4.20	3.52
LINC00638	3.84	4.33	3.32
OIT3	3.84	4.52	3.36
RAB19	3.84	4.38	3.36
OTTHUMG00000184016	3.84	4.19	3.51
TRHDE	3.84	4.78	3.15
OTTHUMG00000020208	3.84	4.26	3.48
LCE1A	3.84	4.81	2.99
PCCA-AS1	3.84	4.26	3.52
TIPARP-AS1	3.84	4.41	3.29
CASQ2	3.84	4.75	3.25
LOC100509621	3.84	4.05	3.03
MAST1	3.84	4.16	3.39
OTTHUMG00000169024	3.84	4.29	3.22
NXPH2	3.84	4.29	3.44
MSX2P1	3.84	4.56	3.25
SAA2	3.84	4.23	3.31
KIF15	3.84	4.26	3.55
ZNF17	3.84	4.37	3.46
GIP	3.84	4.40	3.18
SMCO3	3.84	4.26	3.61
ICAM4	3.84	4.19	3.61
ZSCAN12	3.84	4.40	3.41
SLC25A31	3.84	4.36	3.38
SCGB3A2	3.84	4.53	3.24
NALCN	3.84	4.28	3.52

CHP2	3.84	4.22	3.41
GPR137C	3.84	4.48	3.42
FRRS1L	3.84	4.23	3.22
MIR424	3.84	4.13	3.74
LINC00158	3.84	4.44	3.33
OTTHUMG00000032103	3.84	4.34	3.44
FBXW4P1	3.84	4.48	3.06
OTTHUMG00000086763	3.84	4.38	3.51
TMPRSS6	3.84	4.47	3.44
LINC00515	3.84	4.63	3.06
LCE3D	3.84	4.42	3.41
OTTHUMG00000034556	3.84	4.36	3.56
AGBL2	3.83	4.43	3.32
OTTHUMG00000165790	3.83	4.43	3.39
CNTNAP5	3.83	4.31	3.12
LINC00312	3.83	4.45	3.18
LINC00606	3.83	4.36	3.39
COL28A1	3.83	4.39	3.41
LOC389602	3.83	4.14	3.58
PNLDC1	3.83	4.23	3.53
LOC402779	3.83	4.38	3.03
NAT8L	3.83	4.29	3.41
MIR302D	3.83	4.35	3.28
AKT3-IT1	3.83	4.24	3.40
MAN1B1-AS1	3.83	4.17	3.36
LOC283682	3.83	4.34	3.45
DBX1	3.83	4.19	3.10
SPTSSB	3.83	5.00	2.60
GLP2R	3.83	4.09	3.56
LOC339822	3.83	4.08	3.43
CCDC106	3.83	4.25	3.44
MIR4784	3.83	4.22	2.97
KCNIP4	3.83	4.11	3.42
GPR85	3.83	3.98	3.69
TGM7	3.83	4.39	3.33
TBX22	3.83	4.36	3.42
RPL13A	3.83	4.27	3.18
FLJ42842	3.83	4.56	3.32
SKCG-1	3.83	4.43	3.40
FAM209A	3.83	4.48	2.90
SNORA34	3.83	4.30	3.48
OTTHUMG00000170068	3.83	4.60	3.24
IQCF3	3.83	4.21	3.49
LOC150935	3.83	4.33	3.50
MIR3174	3.83	4.48	3.21
ERBB4	3.83	4.17	3.20
OTTHUMG00000001769	3.83	4.29	3.53
LOC100292073	3.83	4.20	3.53
REEP1	3.83	4.54	3.30

STEAP2-AS1	3.83	4.49	3.41
LYPD6	3.83	4.58	3.42
TBX1	3.83	4.15	3.40
GPR149	3.83	4.63	3.55
RNA5SP341	3.83	4.50	3.36
QRFPR	3.83	4.25	3.33
KCNJ14	3.83	4.19	3.48
SCG2	3.83	5.88	2.87
EMB	3.82	4.49	3.14
OTTHUMG00000008236	3.82	4.21	3.27
OTTHUMG00000169474	3.82	4.33	3.23
FCRL2	3.82	4.31	3.09
OCLN	3.82	4.30	3.44
LOC100996571	3.82	4.36	3.43
MIR1251	3.82	4.28	3.49
OTTHUMG00000152981	3.82	4.77	2.93
RNA5SP295	3.82	5.06	2.46
TRAJ11	3.82	4.88	3.38
LOC339442	3.82	4.29	3.36
MIR595	3.82	4.17	3.35
AVP	3.82	4.34	3.39
LOC730227	3.82	4.20	3.48
OTTHUMG00000159018	3.82	4.26	3.50
SCNN1G	3.82	4.13	3.29
CDC20B	3.82	4.20	3.45
MLK7-AS1	3.82	4.26	3.46
LOC100506134	3.82	4.65	3.14
EFHC2	3.82	4.22	3.22
MIR518A2	3.82	4.62	3.25
MTRNR2L1	3.82	4.72	3.29
FENDRR	3.82	4.28	3.19
SH3GL1P1	3.82	4.30	2.97
BIK	3.82	4.11	3.52
TIMM8A	3.82	4.21	3.12
PPEF1	3.82	4.43	3.28
OTTHUMG00000161365	3.82	4.26	3.46
MIR4497	3.82	4.63	3.27
CTNNA3	3.82	4.39	3.50
USH1C	3.82	4.23	3.52
LYSMD2	3.82	4.20	3.48
ANKS1B	3.82	4.21	3.46
GLUD2	3.82	4.73	3.29
ANAPC1P1	3.82	4.36	3.30
LOC401177	3.82	4.28	3.47
LOC729348	3.82	4.07	3.47
SLC26A1	3.82	4.20	3.38
OTTHUMG00000172266	3.82	4.40	3.30
RORB	3.82	4.06	3.52
HSD17B6	3.82	4.27	3.38

TMEM253	3.82	4.24	3.34
LOC728024	3.82	4.24	3.39
FSIP2	3.82	4.09	3.43
SNORA13	3.82	4.32	3.05
EGF	3.82	4.59	3.43
SNORA70E	3.82	4.52	2.36
MIR643	3.82	4.48	2.59
OTTHUMG00000017871	3.82	4.66	3.19
HMGCS2	3.82	4.17	3.48
OTTHUMG00000170239	3.82	4.41	3.45
CA10	3.82	4.27	3.21
ZNF197-AS1	3.82	4.63	3.21
OTTHUMG00000162992	3.82	4.20	3.27
C17orf47	3.82	4.26	3.51
LOC100507389	3.82	4.21	3.47
LINC00924	3.82	4.35	3.34
TNFSF4	3.82	4.88	3.20
LOC100996578	3.81	4.45	3.11
IL10	3.81	4.25	3.34
SAMD12	3.81	4.21	3.45
OTTHUMG00000017554	3.81	4.17	3.49
TFAM	3.81	4.87	3.24
OTTHUMG00000159201	3.81	4.51	3.33
CPB2-AS1	3.81	4.29	3.22
SCN11A	3.81	4.17	3.34
TRBV6-6	3.81	4.23	3.60
SLC25A21	3.81	4.26	3.34
COL18A1-AS2	3.81	4.21	3.39
MIR550A2	3.81	5.25	2.98
GATM	3.81	4.42	2.98
TMEM40	3.81	4.16	3.37
C1orf195	3.81	4.32	3.41
LRRC66	3.81	4.12	3.26
HTR3D	3.81	4.72	3.30
OTTHUMG00000002754	3.81	4.40	3.18
RBPMS-AS1	3.81	4.23	3.13
OTTHUMG00000162408	3.81	4.08	3.41
LOC729059	3.81	4.38	3.40
ELF5	3.81	4.34	3.30
RNU5F-6P	3.81	4.62	2.91
TMEM51-AS1	3.81	4.16	3.28
MIR1237	3.81	4.42	2.78
CEL	3.81	4.41	3.45
PRKCQ	3.81	4.45	3.47
OR4D10	3.81	4.77	2.80
OTTHUMG00000001764	3.81	4.18	3.40
LINC00845	3.81	4.17	3.20
POU5F2	3.81	4.25	3.41
SLC6A4	3.81	4.48	3.43

TAS2R16	3.81	4.63	3.30
OTTHUMG00000171197	3.81	4.31	3.44
LOC100507670	3.81	4.33	2.99
KCNJ5	3.81	4.21	3.53
NKX2-1	3.81	4.11	3.56
SOX2	3.81	4.08	3.36
SMCO4	3.81	4.15	3.32
OTTHUMG00000086827	3.81	4.23	3.42
BEND4	3.81	4.12	3.44
LOC100505795	3.81	4.29	3.18
CERKL	3.81	4.43	3.54
LINC00710	3.81	4.47	3.48
OTTHUMG00000164547	3.81	4.31	3.18
OTTHUMG00000037131	3.81	4.27	3.25
OTTHUMG00000032809	3.81	4.37	3.26
OTTHUMG00000018179	3.81	4.30	3.32
C5orf34	3.81	4.30	3.47
ZNF695	3.81	4.18	3.61
HIST1H4A	3.81	4.37	3.34
MIR206	3.80	4.35	2.99
SUPT20HL2	3.80	4.21	3.34
LOC100128176	3.80	4.14	3.38
STAU2-AS1	3.80	4.22	3.40
OTTHUMG00000010650	3.80	4.63	3.16
NXN	3.80	4.12	3.41
USP2	3.80	4.18	3.53
HGF	3.80	4.44	3.36
SYNDIG1L	3.80	4.69	3.27
OTTHUMG00000172029	3.80	4.08	3.33
KIRREL3-AS1	3.80	4.18	3.26
NTRK3-AS1	3.80	4.18	3.42
PDRG1	3.80	4.04	3.57
AARD	3.80	3.97	3.59
RHCG	3.80	4.10	3.61
SCGB1D2	3.80	4.27	3.30
OTTHUMG00000154666	3.80	4.44	3.25
SELL	3.80	4.60	3.38
C17orf66	3.80	4.19	3.29
OR2A5	3.80	4.21	2.99
ACSM1	3.80	4.28	3.15
VWC2L-IT1	3.80	4.29	3.04
SOWAHB	3.80	4.18	3.25
NAP1L2	3.80	4.34	3.41
C9orf116	3.80	4.11	3.51
HABP2	3.80	4.17	3.41
LOC285191	3.80	4.23	3.46
RNA5SP304	3.80	4.14	3.20
IGSF5	3.80	4.09	3.52
KRT19P2	3.80	4.10	3.40

FAM169B	3.80	4.01	3.49
FLJ20444	3.80	4.48	3.14
OTTHUMG00000154317	3.80	4.09	3.36
LOC348761	3.80	4.49	3.06
LOC101243545	3.80	4.09	3.44
MIR542	3.80	4.67	2.97
BMP10	3.80	4.24	3.17
OTTHUMG00000152505	3.80	4.26	3.21
OTTHUMG00000059903	3.80	4.65	3.45
OTTHUMG00000151367	3.80	4.45	3.35
LOC100506406	3.80	4.19	3.06
ZRANB3	3.80	4.37	3.24
OTTHUMG00000168462	3.80	4.25	3.31
CYLC2	3.80	4.26	3.29
ZNF671	3.80	4.26	3.19
REG4	3.80	4.07	3.39
OTTHUMG00000017625	3.80	4.16	3.27
OTTHUMG00000162130	3.80	4.10	3.14
OTTHUMG00000037934	3.80	4.44	3.46
KRT17P2	3.80	4.64	2.84
HGD	3.80	4.15	3.56
OTTHUMG00000015101	3.80	4.25	3.48
RAD9B	3.80	4.51	3.21
BEND3	3.80	4.23	3.50
SVIP	3.80	4.24	3.42
OTTHUMG00000168054	3.80	4.68	2.82
OTTHUMG00000153145	3.80	4.28	3.26
LOC100506384	3.80	4.60	3.06
STAT4	3.80	4.09	3.42
OTTHUMG00000032661	3.80	4.28	3.17
BPIFB4	3.80	4.19	3.48
CDH16	3.79	4.31	3.39
LINC00958	3.79	4.32	3.20
MIR202	3.79	4.16	3.38
MIR518A1	3.79	4.56	3.40
MIR125B2	3.79	4.40	3.29
MIR519D	3.79	4.09	3.30
OTTHUMG00000017341	3.79	4.30	3.46
LILRB1	3.79	4.22	3.27
PRAME	3.79	4.30	3.43
LOC100506286	3.79	4.37	3.30
IGFBP1	3.79	4.51	3.47
OTTHUMG00000153092	3.79	4.12	3.35
TTLL8	3.79	4.27	3.53
NAGPA-AS1	3.79	4.33	3.22
MIR4540	3.79	4.66	3.30
LOC100130458	3.79	4.37	3.24
ZNF557	3.79	4.78	2.67
OTTHUMG00000132241	3.79	4.38	3.31

SRP14-AS1	3.79	4.04	3.58
HOXD1	3.79	4.00	3.53
TNNT1	3.79	4.37	3.23
LGALS14	3.79	4.55	3.14
LOC285627	3.79	4.39	3.31
MIR1208	3.79	4.16	3.55
ACOT11	3.79	4.06	3.53
SIX1	3.79	4.24	3.26
GRM8	3.79	4.13	3.54
OTTHUMG00000178670	3.79	4.46	3.32
LINC00608	3.79	4.26	3.51
GINS2	3.79	4.11	3.39
MIR30A	3.79	4.24	2.94
PDE4C	3.79	4.15	3.37
MTOR-AS1	3.79	4.60	3.01
LOC100293962	3.79	4.24	3.32
OTTHUMG00000037300	3.79	4.35	3.41
FOXL2	3.79	4.17	3.31
DGUOK-AS1	3.79	4.16	3.36
GLTPD2	3.79	4.12	3.44
KIT	3.79	4.69	3.03
OTTHUMG00000015978	3.79	4.46	2.99
OTTHUMG00000170371	3.79	4.55	3.29
EIF3IP1	3.79	4.44	3.34
OR10G7	3.79	4.52	3.28
FLJ41278	3.79	4.19	3.39
RNA5SP417	3.79	4.26	3.24
OTTHUMG00000166508	3.79	4.17	3.56
MYCNOS	3.79	4.25	3.38
OTTHUMG00000164201	3.79	4.35	3.29
LOC729681	3.79	4.18	3.37
NKAIN1	3.79	4.26	3.24
DMRT2	3.79	4.06	3.32
CCDC41-AS1	3.79	4.43	3.29
RNA5SP471	3.79	4.81	3.15
GRIK1	3.79	4.11	3.37
BPIFB1	3.79	4.17	3.46
LOC100130673	3.79	4.33	3.45
UBE2NL	3.78	4.75	3.34
LOC100506885	3.78	4.24	3.50
OR8A1	3.78	4.30	3.33
DNAJC22	3.78	4.28	3.30
OTTHUMG00000166544	3.78	4.36	3.39
RHCE	3.78	4.22	2.97
GBX1	3.78	4.12	3.35
OTTHUMG00000066698	3.78	4.70	3.09
OTTHUMG00000008077	3.78	4.29	3.47
IL20RB	3.78	4.41	3.10
IGBP1-AS1	3.78	4.49	3.23

LINC00577	3.78	4.58	3.32
LOC100422737	3.78	4.04	3.56
LOC286359	3.78	4.25	3.52
C21orf88	3.78	4.28	3.54
LMOD3	3.78	4.77	3.34
IGLV3-22	3.78	4.17	3.23
LOC100288798	3.78	4.87	3.27
JAKMIP3	3.78	4.36	3.43
SLITRK1	3.78	4.50	3.19
OTTHUMG00000154822	3.78	4.28	3.30
SLC22A4	3.78	4.01	3.50
SNRPG	3.78	4.48	3.27
HIST1H2AA	3.78	4.54	2.99
NOX1	3.78	4.03	3.47
ADPGK-AS1	3.78	4.46	3.44
B4GALT4-AS1	3.78	4.07	3.38
MOG	3.78	4.52	3.08
OTTHUMG00000166178	3.78	4.35	3.39
TRGV10	3.78	4.37	3.13
KAZN	3.78	4.10	3.45
KIF9-AS1	3.78	4.05	3.42
MIR181B2	3.78	4.38	3.30
OTTHUMG00000160465	3.78	4.64	3.22
IL24	3.78	4.34	3.21
GGN	3.78	4.58	3.46
CDH7	3.78	4.28	3.47
CDC6	3.78	4.61	2.97
BEST2	3.78	4.31	3.47
LINC00442	3.78	4.24	3.31
HESX1	3.78	4.15	3.27
CNGA2	3.78	4.26	3.31
EFHB	3.78	4.08	3.50
LOC100128893	3.78	4.32	3.27
OTTHUMG00000152485	3.78	4.53	3.43
OTTHUMG00000019203	3.78	4.21	3.31
SMR3B	3.78	4.85	2.97
IL3	3.78	4.57	3.30
OTTHUMG00000157659	3.78	4.02	3.48
SPIN4-AS1	3.78	4.16	3.12
OTTHUMG00000017563	3.78	4.04	3.41
C7orf71	3.78	4.04	3.33
NMUR2	3.78	4.38	3.35
OTTHUMG00000018055	3.78	4.17	3.52
SCG5	3.78	4.52	3.28
CACNA2D3	3.78	4.14	3.39
NUTM1	3.78	4.31	3.09
MIR4327	3.78	4.43	3.18
ZNF599	3.78	4.27	3.38
OTTHUMG00000166592	3.78	4.12	3.11

ANKRD31	3.78	4.29	3.32
ZNF554	3.78	4.26	3.29
MIR1323	3.78	4.60	3.06
IGKV1D-17	3.78	4.48	2.87
OTTHUMG00000159346	3.78	4.26	3.39
LOC643401	3.78	4.78	3.28
VN2R9P	3.78	4.18	3.29
IMMP1L	3.78	4.29	3.41
PNLIP	3.78	4.07	3.33
SNORD61	3.78	4.50	3.10
LOC100507584	3.78	4.21	3.39
ASAH2B	3.78	4.44	3.43
LOC388414	3.78	4.12	3.38
SULT1C2P1	3.78	4.39	3.13
KLHL41	3.78	4.27	3.46
AP1M2	3.77	4.42	3.34
OTTHUMG00000035479	3.77	4.02	3.38
MGC34034	3.77	4.16	3.33
FIGNL1	3.77	4.24	3.02
KCNH6	3.77	4.15	3.33
MYOCD	3.77	4.11	3.34
NEUROG1	3.77	4.35	3.17
LOC402269	3.77	4.14	3.08
OTTHUMG00000164700	3.77	4.42	3.14
RIMKLA	3.77	4.10	3.23
ZNF771	3.77	4.26	3.23
POLE2	3.77	4.45	3.26
TREML5P	3.77	4.67	2.70
IQCJ-SCHIP1-AS1	3.77	4.47	3.42
DHFR	3.77	4.17	3.03
RNA5SP357	3.77	4.34	2.83
LOC388820	3.77	4.05	3.14
LINC00595	3.77	4.20	3.07
OTTHUMG00000160332	3.77	4.71	3.21
CCDC112	3.77	4.30	3.36
FAM9C	3.77	4.20	3.35
LINC00029	3.77	4.16	3.42
ZNF223	3.77	4.46	3.37
OR56A5	3.77	4.40	3.13
SPINK2	3.77	4.09	3.38
TCEB3C	3.77	4.99	3.20
KCNG3	3.77	4.42	3.31
LINC00572	3.77	4.00	3.54
NKX2-4	3.77	4.09	3.53
CXCL9	3.77	4.48	2.90
OTTHUMG00000164854	3.77	4.08	3.62
BHMT2	3.77	4.13	3.49
CCDC39	3.77	4.12	3.08
KIF25-AS1	3.77	4.07	3.42

HIPK4	3.77	4.11	3.34
OTTHUMG00000156391	3.77	4.11	3.39
MORN5	3.77	4.24	3.29
ITIH3	3.77	4.15	3.38
HOXC-AS3	3.77	4.34	3.47
FBXL15	3.77	4.03	3.44
CTNNA2	3.77	4.19	3.33
CGN	3.77	4.19	3.46
PGA5	3.77	4.30	3.04
OTTHUMG00000019649	3.77	4.04	3.54
SLC45A2	3.77	4.19	3.26
ZWILCH	3.77	4.29	3.40
PRODH2	3.77	4.28	3.48
DIP2A-IT1	3.77	4.29	3.17
GLYATL1	3.77	4.34	3.38
WFDC6	3.77	4.14	3.55
KIF6	3.76	4.19	3.36
MIR760	3.76	3.92	3.46
TCF15	3.76	4.19	3.43
ZNF252P-AS1	3.76	4.02	3.20
LOC643542	3.76	4.09	3.22
OTTHUMG00000040729	3.76	4.12	3.38
SLCO4A1	3.76	4.32	3.18
LOC256880	3.76	4.38	3.43
LOC644554	3.76	4.08	3.15
LOC401442	3.76	4.32	3.15
KALP	3.76	4.01	3.46
CDH1	3.76	4.14	3.51
DLL3	3.76	4.06	3.35
CETN1	3.76	4.04	3.45
PLEK2	3.76	4.26	3.19
MIR568	3.76	4.55	2.94
SLC25A48	3.76	3.97	3.54
KRTAP6-3	3.76	4.65	3.29
CCBE1	3.76	4.26	3.38
TDRD6	3.76	4.16	3.38
RHBDL3	3.76	4.59	3.29
FSTL4	3.76	4.20	3.30
OTTHUMG00000172094	3.76	4.17	3.39
GFOD1-AS1	3.76	4.19	3.36
LINC00410	3.76	4.32	3.16
RHD	3.76	4.73	2.19
HCN2	3.76	4.05	3.35
LOC100505685	3.76	4.23	3.45
LOC100505754	3.76	4.58	3.11
DCDC2C	3.76	4.16	3.47
NOS2	3.76	4.40	3.31
CELA1	3.76	4.06	3.42
CCDC11	3.76	4.11	3.50

OTTHUMG00000015939	3.76	4.28	3.18
GOLGA8UP	3.76	4.06	3.20
SIT1	3.76	4.38	3.26
GKAP1	3.76	4.07	3.49
OTTHUMG00000032120	3.76	4.31	3.17
TRAJ27	3.76	4.52	3.28
OTTHUMG00000166131	3.76	4.08	3.54
SNORA14B	3.76	5.01	2.78
LINC00202-2	3.76	4.22	3.17
OTTHUMG00000164086	3.76	4.15	3.29
USP29	3.76	4.37	3.46
TMEM99	3.76	4.31	3.14
ATP8A2	3.75	4.06	3.13
SH2D4B	3.75	4.10	3.53
DNAL4	3.75	3.94	3.38
NRXN1	3.75	4.15	3.53
SLC26A9	3.75	4.31	3.23
OTTHUMG00000022550	3.75	4.33	2.78
LINC00051	3.75	4.38	3.23
LINC00517	3.75	4.47	3.40
OTTHUMG00000150045	3.75	4.34	3.04
OTTHUMG00000162962	3.75	4.10	3.28
LOC100506328	3.75	4.05	3.35
NR5A2	3.75	4.37	3.34
FAM81B	3.75	4.45	3.15
MMP25	3.75	4.35	3.45
ITLN2	3.75	4.12	3.20
CYP2A13	3.75	4.23	3.38
OTTHUMG00000032679	3.75	4.06	2.86
OTTHUMG00000163965	3.75	4.31	3.26
COX6B2	3.75	4.10	3.36
RNU7-22P	3.75	4.37	3.21
TRAJ56	3.75	4.57	2.67
OTTHUMG00000161707	3.75	4.46	3.03
FGF13-AS1	3.75	4.57	3.38
LOC644838	3.75	4.09	3.45
RNF125	3.75	4.27	3.16
PRKG2	3.75	4.27	3.37
SLC7A9	3.75	4.18	3.45
CPSF4L	3.75	4.03	3.29
PICK1	3.75	4.25	3.25
DPY19L2P3	3.75	4.51	3.35
OTTHUMG00000168121	3.75	4.80	3.22
IFNA2	3.75	4.16	3.13
ALB	3.75	4.30	3.42
OTTHUMG00000045137	3.75	4.09	3.46
LOC100506011	3.75	4.10	3.18
IGHV3-74	3.75	4.47	3.17
HK3	3.75	4.16	3.11

SNTG2	3.75	4.30	3.41
POU6F2-AS1	3.75	4.11	3.37
ZNF165	3.75	4.38	3.31
OTTHUMG00000163129	3.75	4.18	3.34
LOC401164	3.75	4.38	3.04
BEX1	3.75	4.55	2.72
OTTHUMG00000018026	3.75	4.33	3.40
LRRC36	3.75	4.29	3.21
OTTHUMG00000018320	3.75	4.16	3.36
OTTHUMG00000017587	3.75	4.29	3.09
MIR548O2	3.75	4.60	2.55
FCAR	3.75	4.17	3.50
OTTHUMG00000164773	3.75	4.05	3.41
CXXC4	3.75	4.20	3.16
CATSPERG	3.75	4.29	3.43
CERS3	3.75	4.15	3.29
OTTHUMG00000157083	3.75	4.03	3.26
LENEP	3.75	4.54	3.35
GLYATL2	3.75	4.77	2.38
URB2	3.75	4.03	3.53
CARD11	3.75	4.20	3.30
TTTY4	3.75	4.14	3.42
LOC649395	3.75	4.71	2.77
OR6F1	3.74	4.13	3.17
MIR4776-2	3.74	4.32	3.07
MMACHC	3.74	4.27	3.38
LOC723805	3.74	4.46	3.27
OTTHUMG00000151745	3.74	4.18	3.27
DSC1	3.74	4.24	3.32
RHOXF2B	3.74	4.20	3.28
PIR-FIGF	3.74	4.19	3.38
LINC00520	3.74	4.29	3.40
LOC100129935	3.74	4.08	3.17
MUC7	3.74	4.45	3.08
KIF5A	3.74	4.05	3.18
DEFB108B	3.74	4.11	3.56
DEFB134	3.74	4.02	3.31
OTTHUMG00000151623	3.74	4.10	3.33
BAIAP2-AS1	3.74	4.33	3.47
ANKRD1	3.74	4.05	3.42
JPH1	3.74	4.15	3.39
ADH4	3.74	4.18	3.51
NXF2	3.74	4.11	3.27
MGST1	3.74	4.16	3.27
SNAP25-AS1	3.74	3.93	3.46
OR13J1	3.74	4.46	2.67
ANKRD36BP2	3.74	4.73	3.22
OR6B3	3.74	4.13	3.06
OTTHUMG00000015403	3.74	3.97	3.24

LINC00348	3.74	4.57	3.11
PAR1	3.74	4.16	3.26
OVCH1	3.74	4.36	3.37
RNA5SP131	3.74	4.33	2.55
TRIAP1	3.74	4.12	3.24
MIS18A-AS1	3.74	4.12	2.90
BBS12	3.74	4.41	3.31
MIR3183	3.74	4.09	3.09
H2AFB3	3.74	4.09	3.42
MSRB1	3.74	4.14	3.37
OTTHUMG00000171523	3.74	3.99	3.42
SPATA8	3.74	4.12	3.37
OTTHUMG00000169993	3.74	5.14	2.76
EPHA6	3.73	4.09	3.39
MIR518C	3.73	4.62	2.76
OTTHUMG00000015988	3.73	4.09	3.38
KRT71	3.73	4.37	2.78
OTTHUMG00000170325	3.73	4.28	3.27
C1orf68	3.73	4.09	3.37
AIRE	3.73	4.11	3.34
LOC283038	3.73	4.04	3.36
OTTHUMG00000166465	3.73	4.13	3.37
LRRC4B	3.73	4.25	3.19
LOC101060609	3.73	4.16	3.30
LRRC31	3.73	4.43	3.26
CXCR3	3.73	4.58	3.08
CA14	3.73	4.42	3.22
KLK13	3.73	4.09	3.33
TRIM71	3.73	4.31	2.83
ALKBH1	3.73	4.16	3.28
NPY4R	3.73	4.15	3.40
OTTHUMG00000170586	3.73	4.08	3.48
LOC100131497	3.73	4.88	3.18
FBXO15	3.73	4.15	3.44
SIAH3	3.73	4.32	3.00
STX19	3.73	4.07	3.27
KHDC1L	3.73	4.67	3.03
LOC100268168	3.73	4.15	3.09
DSCC1	3.73	4.46	3.19
OTTHUMG00000172115	3.73	4.29	3.29
ZNF235	3.73	4.04	3.44
RNA5SP259	3.73	4.62	3.01
SLC28A3	3.73	4.07	3.33
CYP39A1	3.73	4.20	3.17
RAP1GAP	3.73	3.96	3.36
HP	3.73	5.11	2.89
TCF7L1-IT1	3.73	4.30	3.04
OTTHUMG00000179967	3.73	4.23	2.97
CRYBA1	3.73	4.16	3.24

OTTHUMG00000170044	3.73	4.46	3.08
OTTHUMG00000170731	3.73	4.27	3.06
DKFZp451B082	3.73	4.21	3.37
LOC100506016	3.73	3.97	3.26
SLC4A8	3.73	3.92	3.27
SNORD114-24	3.73	5.51	2.82
KCTD16	3.73	3.94	3.46
LOC340357	3.73	4.18	3.24
LOC554206	3.73	4.48	3.27
MOGAT2	3.73	4.03	3.15
SPINK6	3.73	4.72	3.33
RHOV	3.73	4.25	3.40
C3orf43	3.73	4.21	3.34
TMEM239	3.73	4.36	3.44
LINC00540	3.73	4.30	2.88
CNTNAP2	3.73	3.97	3.51
NPS	3.73	4.21	3.06
C17orf67	3.73	4.03	3.22
LRMP	3.73	4.06	3.49
ACSM3	3.73	4.12	3.57
OTTHUMG00000163130	3.73	4.15	3.33
IRX4	3.73	4.28	3.21
OTTHUMG00000015771	3.73	4.47	3.11
LINC00486	3.73	4.16	3.36
C1QTNF9	3.73	4.40	2.54
ERVV-2	3.73	4.25	3.23
LOC344967	3.73	4.16	3.24
LINGO2	3.73	4.12	3.40
DHRSX-IT1	3.73	4.07	3.42
COL4A3	3.73	4.04	3.37
OTTHUMG00000032768	3.73	4.19	3.15
LOC100506369	3.73	4.15	3.29
ABHD17B	3.72	4.27	3.30
CD3G	3.72	4.14	3.23
LOC100506795	3.72	4.32	3.32
LOC100132529	3.72	4.03	3.42
NXPH1	3.72	4.14	3.54
DRD1	3.72	3.98	3.30
LOC100505695	3.72	4.31	3.23
LOC646522	3.72	4.23	3.43
FAM87A	3.72	4.20	3.19
OTTHUMG00000164156	3.72	4.27	3.22
MIR4263	3.72	4.80	2.66
OLIG3	3.72	4.15	3.19
BTBD17	3.72	4.10	3.21
GLRB	3.72	4.42	3.23
LOC339685	3.72	4.37	3.36
OTTHUMG00000032101	3.72	4.27	3.18
SLITRK3	3.72	4.10	3.22

OTTHUMG00000032491	3.72	4.29	3.19
TRAV20	3.72	4.25	3.12
DLG1-AS1	3.72	4.72	3.34
KLHL14	3.72	4.18	3.37
ZNF681	3.72	4.34	3.24
OR5K2	3.72	4.35	3.23
OTTHUMG00000010944	3.72	4.40	3.35
OTTHUMG00000020894	3.72	4.13	3.39
ERC2	3.72	4.06	3.16
PVALB	3.72	4.28	3.20
NLGN1	3.72	4.05	3.37
LPAL2	3.72	4.49	3.11
GALNTL6	3.72	4.12	3.36
MARVELD2	3.72	3.93	3.40
HBBP1	3.72	4.70	2.94
OTTHUMG00000162923	3.72	4.20	3.31
ZNF501	3.72	4.17	3.21
OTTHUMG00000159096	3.72	3.97	3.40
FBXL16	3.72	4.01	3.49
FAM163A	3.72	4.19	3.25
FBXL13	3.72	4.37	3.24
SLC38A11	3.72	4.57	3.11
HNRNPA1L2	3.72	4.48	3.28
RNA5SP387	3.72	4.57	3.01
LOC100506258	3.72	4.08	3.29
ZNF843	3.72	4.19	2.94
OTTHUMG00000170725	3.72	4.13	3.29
FUT3	3.72	3.92	3.36
OTTHUMG00000168240	3.72	4.11	3.15
SLFN13	3.72	4.16	3.15
OTTHUMG00000163339	3.72	4.14	3.18
OTTHUMG00000166909	3.72	4.06	3.36
LOC647983	3.72	4.14	3.22
KCNMB3	3.72	3.95	3.37
LOC100506895	3.72	3.99	3.23
NT5C1A	3.72	4.15	3.18
PTPRG-AS1	3.72	3.92	3.41
LOC157273	3.72	4.10	3.24
OTTHUMG00000153617	3.72	4.21	3.23
MKRN1	3.71	4.27	3.30
CDKN2A	3.71	4.25	3.42
OTTHUMG00000156460	3.71	4.29	3.21
OTTHUMG00000170259	3.71	4.67	3.11
OTTHUMG00000015665	3.71	4.36	3.27
LOC100630918	3.71	4.20	3.19
LOC254028	3.71	4.13	3.34
OTTHUMG00000019542	3.71	4.71	2.76
OTTHUMG00000156619	3.71	4.46	3.09
LOC286190	3.71	4.11	3.13

RPS26P11	3.71	4.58	3.30
CLDN22	3.71	4.30	3.15
FDXACB1	3.71	4.25	3.30
OTTHUMG00000021819	3.71	4.02	3.44
OTTHUMG00000173284	3.71	4.09	3.58
OTTHUMG00000155766	3.71	4.50	2.96
ZDHHC22	3.71	4.23	3.51
OTTHUMG00000166137	3.71	3.95	3.27
MIR4305	3.71	4.66	3.29
UMODL1	3.71	4.16	3.35
LOC100287834	3.71	5.43	2.33
USH2A	3.71	3.99	3.36
MOV10L1	3.71	4.43	3.06
IGHV3-20	3.71	4.36	3.09
OTTHUMG00000151328	3.71	3.93	3.39
OTTHUMG00000040862	3.71	4.11	3.16
XXYLT1-AS1	3.71	4.00	3.38
OTTHUMG00000166233	3.71	4.11	3.27
OTTHUMG00000020074	3.71	4.30	3.29
PIP5K1B	3.71	4.07	3.31
RLBP1	3.71	4.01	3.45
OTTHUMG00000016072	3.71	4.09	2.97
KLK12	3.71	4.13	3.19
NKX6-1	3.71	4.15	3.33
PRAMEF4	3.71	4.45	2.84
OTTHUMG00000170117	3.71	4.28	3.29
LPPR4	3.71	4.33	2.97
HOMER2	3.71	4.34	3.40
OTTHUMG00000168223	3.71	4.23	2.98
CSN1S1	3.71	5.61	2.75
LINC00551	3.71	4.17	3.25
MIR553	3.71	4.57	2.88
LOC100506714	3.71	4.11	3.39
C2CD4B	3.71	4.41	3.40
OTTHUMG00000020688	3.71	4.01	3.42
OTTHUMG00000161792	3.71	4.11	3.30
LOC100129098	3.71	4.14	3.06
OTTHUMG00000057558	3.71	4.30	2.91
DSPP	3.71	4.15	3.29
EFCAB3	3.70	4.07	3.20
PAX9	3.70	4.33	3.32
FLT3	3.70	4.09	3.28
SERPINA9	3.70	4.07	3.43
DOCK9-AS1	3.70	4.12	3.07
LINC00681	3.70	4.16	3.34
LINC00483	3.70	4.11	3.05
CALML5	3.70	4.13	3.33
OTTHUMG00000176716	3.70	4.45	3.14
OTTHUMG00000019594	3.70	4.29	3.20

POTED	3.70	4.37	2.98
LBX1-AS1	3.70	4.20	3.09
ACVR2B	3.70	4.08	3.39
OTTHUMG00000016045	3.70	4.04	3.43
OTTHUMG00000015812	3.70	4.12	3.30
CXCR1	3.70	4.50	3.23
KIAA1958	3.70	3.91	3.49
STOML3	3.70	4.15	3.43
ZNF416	3.70	4.01	3.21
ATP4B	3.70	4.04	3.35
ZNF428	3.70	3.93	3.30
SLC12A5	3.70	4.39	2.86
OTTHUMG00000164999	3.70	4.02	3.39
TAS2R19	3.70	5.19	3.00
LOC729040	3.70	4.18	3.19
LOC100507639	3.70	4.46	3.32
OTTHUMG00000017483	3.70	4.11	3.28
OTTHUMG00000014989	3.70	4.32	3.23
LOC641515	3.70	4.17	3.13
TPM3	3.70	4.21	3.13
LINC00601	3.70	4.26	3.26
TXNDC8	3.70	4.22	2.99
OR13C4	3.70	4.45	3.18
TRBV21OR9-2	3.70	4.47	2.97
C19orf26	3.70	4.23	3.44
PAX3	3.70	3.98	3.18
OTTHUMG00000172086	3.70	4.35	3.23
DKFZP434L187	3.70	4.20	3.20
BDNF-AS	3.70	3.85	3.45
MME-AS1	3.70	5.47	2.82
HMMR	3.70	4.20	3.12
C6orf100	3.70	3.98	3.41
OTTHUMG00000155764	3.70	4.46	3.18
MBL2	3.70	4.11	3.17
OTTHUMG00000170227	3.70	4.68	3.17
LOC100505588	3.70	4.01	3.35
OTTHUMG00000156060	3.70	4.16	3.22
KLHL36	3.70	4.07	3.20
GYS1	3.70	4.36	3.29
OTTHUMG00000020030	3.70	4.06	3.24
FAM104B	3.70	4.16	3.28
PATE2	3.69	4.34	3.25
LCTL	3.69	4.09	3.06
GOLT1A	3.69	4.09	3.20
NCBP2L	3.69	4.10	3.42
SLC5A11	3.69	4.22	3.37
CCR6	3.69	4.05	3.37
XXYLT1-AS2	3.69	4.30	3.08
C3orf36	3.69	4.20	3.07

FLJ46134	3.69	4.08	3.46
CCDC177	3.69	4.58	2.90
OTTHUMG00000165711	3.69	4.35	3.08
CYP4F12	3.69	4.08	3.23
SNORD114-4	3.69	5.89	2.61
TRPC6	3.69	4.37	2.89
NKX2-6	3.69	4.05	3.34
OTTHUMG00000021872	3.69	4.17	3.24
OTTHUMG00000169952	3.69	4.31	3.22
GABRR3	3.69	4.19	3.05
CAPS2	3.69	3.97	3.22
OTTHUMG00000166208	3.69	4.30	2.93
KIAA1211	3.69	4.30	3.26
MIRLET7I	3.69	4.07	2.85
TRPV5	3.69	4.30	3.00
CSTA	3.69	4.53	3.08
DAPK1-IT1	3.69	4.94	2.50
NODAL	3.69	4.09	3.37
IL9	3.69	4.19	3.38
MIR623	3.69	4.27	3.16
OTTHUMG00000021098	3.69	4.02	3.34
ACSL6	3.69	4.08	3.31
DOHH	3.69	3.99	3.47
FLJ41481	3.69	4.09	3.10
LOC100128787	3.69	3.93	3.33
OTTHUMG00000155708	3.69	4.10	3.28
OTTHUMG00000158980	3.69	4.23	3.20
LOC255187	3.69	4.79	3.24
SLC39A12	3.69	3.96	3.34
HPSE	3.69	4.30	2.93
LOC729770	3.69	4.14	3.18
OR7E37P	3.69	4.12	3.34
GRAMD1C	3.69	3.91	3.27
DEPDC1B	3.69	4.23	3.36
DKFZP434H168	3.68	3.96	3.25
OTTHUMG00000017397	3.68	4.17	2.97
OR13G1	3.68	4.32	3.13
OTTHUMG00000171836	3.68	4.76	3.16
BEND2	3.68	4.20	3.14
SNORD113-2	3.68	5.33	2.77
OR1D5	3.68	5.44	2.18
OTTHUMG00000168902	3.68	4.11	3.36
LOC100127974	3.68	4.14	2.96
LRP1B	3.68	4.13	3.20
LINC00424	3.68	4.12	3.18
OTTHUMG00000162710	3.68	4.38	3.28
LRCH2	3.68	4.21	2.82
ZNF876P	3.68	4.21	3.20
OTTHUMG00000155021	3.68	4.18	3.39

OTTHUMG00000172157	3.68	4.09	3.32
MYH1	3.68	4.51	3.33
EFNB2	3.68	4.23	2.85
LOC644189	3.68	4.13	2.87
OTTHUMG00000162156	3.68	4.07	3.01
CNR1	3.68	4.63	3.37
OTTHUMG00000035542	3.68	4.48	3.18
ST7-AS1	3.68	4.03	3.27
MUC19	3.68	4.17	3.36
OR7E24	3.68	4.09	3.35
OTTHUMG00000158939	3.68	3.93	3.33
OTTHUMG00000163857	3.68	4.40	3.06
OR7D4	3.68	4.44	3.21
MIR497	3.68	4.20	3.39
OR51A4	3.68	4.69	2.55
CLEC12A	3.68	4.43	2.72
SPTA1	3.68	3.96	3.45
ASXL3	3.68	4.22	3.26
ARHGEF35	3.68	3.91	3.34
OTTHUMG00000171183	3.68	4.12	3.27
DNAJC27-AS1	3.68	4.23	3.30
M1AP	3.68	4.07	3.20
SPINK5	3.68	4.16	3.48
ETNPPL	3.68	4.10	3.30
RNA5SP66	3.68	4.36	2.89
CCDC96	3.68	4.11	3.27
ABCD2	3.68	4.35	2.94
RIBC2	3.68	4.16	3.30
MIR3199-1	3.68	5.44	3.01
OR4X2	3.68	4.33	3.01
FLJ46284	3.68	4.01	3.34
OTTHUMG00000016041	3.68	3.80	3.47
PTGER3	3.68	4.44	3.14
PDC	3.68	4.22	3.43
ALDH1A2	3.68	4.50	3.08
EMR1	3.68	4.20	3.15
RAB40A	3.68	3.90	3.09
LOC285626	3.68	4.04	3.48
CACNA2D3-AS1	3.68	4.16	3.30
UBE2MP1	3.68	4.62	3.10
OTTHUMG00000156395	3.68	3.97	3.33
LOC285548	3.67	4.17	3.25
CXCR2P1	3.67	3.92	3.20
IGHV1-58	3.67	4.32	2.76
LOC100507630	3.67	4.12	3.22
DEFB122	3.67	4.35	3.07
HTR1E	3.67	4.23	3.29
APOL4	3.67	4.10	3.30
UBXN11	3.67	3.91	3.35

OTTHUMG00000172297	3.67	4.57	3.18
FLJ38379	3.67	4.62	2.46
RD3	3.67	4.21	3.33
ST8SIA2	3.67	4.08	3.18
OTTHUMG00000163254	3.67	4.33	3.20
AKR1B15	3.67	4.17	3.13
LINC00441	3.67	4.15	3.06
FAM19A2	3.67	4.29	3.02
CADM2-AS1	3.67	4.29	3.00
LRAT	3.67	4.23	2.99
TBR1	3.67	3.95	3.45
CTAGE1	3.67	4.11	3.12
TRBV10-1	3.67	4.03	3.43
MMP12	3.67	4.04	3.45
OTTHUMG00000161245	3.67	4.28	3.23
PCDH20	3.67	4.10	3.27
NOX3	3.67	3.93	3.31
OR2G2	3.67	4.25	2.91
MIR383	3.67	4.01	3.38
OTTHUMG00000153374	3.67	4.19	3.09
LOC440905	3.67	4.90	2.89
DLGAP1-AS2	3.67	3.98	2.97
PDE6H	3.67	3.99	3.26
LOC100505545	3.67	4.27	3.22
CXorf27	3.67	4.11	3.07
RPSAP58	3.67	4.05	3.19
DEFB130	3.67	3.90	3.46
OR4C15	3.67	4.13	3.10
OTTHUMG00000169029	3.67	4.24	2.99
C8B	3.67	4.07	3.08
GPR39	3.67	4.02	3.21
RNU7-58P	3.67	4.38	2.89
NR1I2	3.67	3.91	3.34
CASZ1	3.67	4.15	3.11
ALDH4A1	3.67	3.92	3.44
AMY2A	3.67	4.41	3.06
OTTHUMG00000155824	3.67	4.26	3.05
LOC100507540	3.67	3.87	3.23
OTTHUMG00000016042	3.67	4.05	3.24
MYB	3.67	3.95	3.17
LINC00402	3.67	4.19	3.13
LOC100506175	3.67	4.39	2.44
LINC00433	3.67	3.97	3.35
C1orf64	3.67	4.25	3.20
SNORD114-6	3.67	4.65	1.84
LOC100507664	3.67	4.11	3.17
OTTHUMG00000164880	3.67	4.26	3.43
C21orf90	3.67	4.22	2.99
OTTHUMG00000172113	3.66	4.12	3.32

SPHKAP	3.66	4.19	3.39
OTTHUMG00000011879	3.66	4.98	2.79
TEX30	3.66	4.17	3.18
CDH12	3.66	3.96	3.36
MIR196A2	3.66	4.44	3.00
PRTN3	3.66	4.20	2.88
OTTHUMG00000178331	3.66	4.64	2.97
UCP1	3.66	3.95	3.28
SEC14L3	3.66	4.27	3.39
YY2	3.66	4.08	3.25
DCX	3.66	4.10	3.36
IFIT2	3.66	4.39	2.96
PPP2R2B-IT1	3.66	4.13	3.38
OTTHUMG00000164458	3.66	4.32	2.87
RAB3IP	3.66	3.89	3.37
LRFN2	3.66	4.19	3.02
RABGAP1L-IT1	3.66	4.69	2.85
ROS1	3.66	4.26	3.20
ZP4	3.66	4.08	3.11
ABCC13	3.66	4.39	3.10
SLC16A14	3.66	3.99	3.25
SMIM5	3.66	4.07	3.23
LRRC52	3.66	3.94	3.41
MIR153-1	3.66	4.11	3.39
RDM1	3.66	4.03	3.30
TNMD	3.66	4.18	3.17
B3GALT5	3.66	4.24	3.28
LOC285084	3.66	4.21	3.10
LINC00920	3.66	4.16	3.40
LMO1	3.66	4.07	3.38
OTTHUMG00000154100	3.66	4.13	3.22
CXXC1P1	3.66	4.03	3.31
SNX20	3.66	4.25	3.21
KLHL15	3.66	4.08	2.98
LOC340094	3.66	4.05	3.22
LOC100507537	3.66	4.17	3.27
PGPEP1L	3.66	4.06	3.08
OTTHUMG00000151796	3.66	4.00	3.21
SVOPL	3.66	4.14	3.22
MIR888	3.66	4.58	2.79
OTTHUMG00000022338	3.66	4.44	2.86
OR4C16	3.66	4.37	2.90
UCN3	3.66	3.98	3.14
LOC100506446	3.66	4.07	3.27
IGKV1D-27	3.66	4.56	2.79
FLJ46010	3.66	4.47	3.29
CCER1	3.66	3.85	3.41
FAM105A	3.66	4.66	2.94
LOC100996310	3.66	4.01	3.15

OTTHUMG00000163392	3.66	4.33	3.15
RALYL	3.66	3.95	3.28
LOC100507073	3.66	4.26	3.25
LOC728769	3.66	3.87	3.29
TTTY20	3.66	4.07	3.29
YTHDC1	3.66	4.15	2.88
TSSC1-IT1	3.66	3.93	3.38
AXDND1	3.66	3.90	3.18
NUDT9P1	3.65	4.03	3.00
OTTHUMG00000161687	3.65	3.96	3.27
GINS4	3.65	3.98	3.40
TP53RK	3.65	4.24	3.11
OTTHUMG00000036746	3.65	4.19	3.43
OTTHUMG00000168169	3.65	4.27	3.21
IGHV3OR16-7	3.65	4.07	3.09
LINC00940	3.65	3.93	3.41
OTTHUMG00000017902	3.65	3.84	3.27
SNORD1A	3.65	5.10	2.85
ZNF502	3.65	4.45	2.88
CORO2A	3.65	3.95	3.33
PCED1B	3.65	4.11	3.11
NRG1	3.65	4.02	3.24
HS6ST2-AS1	3.65	4.15	3.41
IRGM	3.65	4.20	3.05
OTTHUMG00000019067	3.65	3.99	2.98
TMEM108	3.65	4.13	3.14
SLC17A4	3.65	4.11	3.22
OTOP3	3.65	4.54	3.02
CDY2A	3.65	4.44	2.98
LOC646903	3.65	4.06	3.36
ZNF682	3.65	4.39	3.08
CHEK2	3.65	4.26	3.30
OTTHUMG00000154152	3.65	3.98	3.43
ITGA4	3.65	4.09	3.28
MIR802	3.65	4.33	3.30
MAGEA4	3.65	3.95	3.35
TACSTD2	3.65	4.00	2.80
LOC100506368	3.65	4.13	3.15
SPAG11A	3.65	4.38	3.16
LOC440028	3.65	3.97	3.32
ZNF503-AS1	3.65	4.05	3.21
FAM212A	3.65	4.00	3.22
ATP8B5P	3.65	4.11	3.15
MIR1293	3.65	4.21	3.10
LOC100128714	3.65	4.06	3.10
OR9Q1	3.65	3.99	3.15
DCHS2	3.65	3.92	3.25
FANCD2OS	3.65	4.24	2.95
KCNAB1	3.64	3.84	3.24

OTTHUMG00000166770	3.64	4.13	3.20
OTTHUMG00000015925	3.64	4.37	3.05
NPBWR2	3.64	4.25	3.35
CITED4	3.64	4.04	3.12
OTTHUMG00000031861	3.64	4.00	3.21
KRT33B	3.64	4.74	3.08
OR2H1	3.64	4.28	3.21
OR10A7	3.64	4.34	2.77
IGLV2-33	3.64	4.46	2.68
OTTHUMG00000164258	3.64	4.39	3.15
C1orf100	3.64	3.93	3.25
ARHGEF9-IT1	3.64	3.99	3.11
MIR656	3.64	4.13	3.30
MIR4650-1	3.64	4.35	3.19
RNF144A-AS1	3.64	4.28	3.28
LDHAL6A	3.64	4.28	3.09
SKOR2	3.64	4.08	3.35
LOC643923	3.64	3.90	3.28
C2orf66	3.64	3.96	3.15
OTTHUMG00000171652	3.64	4.44	2.96
OTTHUMG00000172318	3.64	4.39	3.20
OTTHUMG00000155881	3.64	4.10	3.34
ST7-AS2	3.64	4.15	3.35
F2RL3	3.64	4.21	3.02
C7orf33	3.64	4.00	3.30
MIR4493	3.64	4.08	3.25
PRNT	3.64	4.41	3.03
HDAC11-AS1	3.64	3.95	3.13
TBX5-AS1	3.64	4.29	3.31
MAPK11	3.64	4.15	3.26
RNU6-63P	3.64	3.98	3.34
LOC100133306	3.64	3.89	3.20
IGHV1-3	3.64	4.51	3.09
FKBP1C	3.64	4.21	3.26
IRX6	3.64	4.27	2.77
FCGR3B	3.64	4.32	2.76
OTTHUMG00000167762	3.64	4.19	3.00
CYP2B6	3.64	4.39	2.98
CTXN2	3.64	3.99	3.00
LRRC37A5P	3.64	4.02	3.28
OTTHUMG00000015322	3.64	4.35	3.16
SLC4A1	3.64	4.11	3.20
OTTHUMG00000172461	3.64	4.07	3.37
ZCCHC12	3.64	4.16	3.27
RNA5-8SP7	3.64	4.22	2.97
PRKCQ-AS1	3.64	4.34	3.02
KLRG1	3.64	3.97	3.43
MIR192	3.63	4.38	3.03
OTTHUMG00000171626	3.63	4.12	3.13

LOC100505727	3.63	3.91	2.94
GLTPD1	3.63	4.03	3.24
OTTHUMG00000172090	3.63	4.23	3.16
LINC00895	3.63	4.08	3.25
C10orf112	3.63	4.14	3.26
C1orf194	3.63	3.80	3.38
EFCAB14-AS1	3.63	4.11	2.97
SHISA6	3.63	3.99	3.35
SCN9A	3.63	4.69	2.64
OTTHUMG00000164154	3.63	4.12	3.27
ABCA17P	3.63	4.33	3.26
ZNF492	3.63	4.19	2.75
LOC100272217	3.63	3.99	3.05
TTK	3.63	4.26	3.21
PTCHD1	3.63	4.60	3.20
FCRL3	3.63	3.90	3.39
DNAJC28	3.63	4.34	3.12
PRR23B	3.63	4.07	3.09
CCDC81	3.63	3.84	3.42
OTTHUMG00000152555	3.63	4.43	3.28
TMEM254-AS1	3.63	4.17	2.81
LOC729558	3.63	4.09	3.03
LOC100996721	3.63	4.39	2.84
OTTHUMG00000161646	3.63	4.38	3.27
ACPP	3.63	3.97	3.30
OR5A2	3.63	4.63	2.86
NLRP5	3.63	3.97	2.97
CYP4F11	3.63	4.20	3.11
FREM3	3.63	4.06	3.34
ATP13A4	3.63	4.08	3.34
OR5V1	3.63	4.11	2.97
LOC100131257	3.63	4.08	3.36
OTTHUMG00000012473	3.63	4.11	3.32
OTX2	3.63	4.05	3.22
TMEM246	3.63	3.92	3.44
TMEM132D	3.63	3.88	3.46
BST2	3.63	4.59	2.96
TLR10	3.63	4.35	2.96
GNN	3.63	3.92	3.28
C16orf55	3.63	4.25	2.80
SLC6A5	3.63	4.03	3.17
OTTHUMG00000167328	3.63	4.11	3.27
EOMES	3.63	3.98	3.41
MIR4783	3.63	4.20	3.28
OTTHUMG00000167185	3.63	3.84	3.25
OTTHUMG00000151845	3.63	4.96	3.01
BRIP1	3.63	3.99	3.28
SEZ6	3.63	4.08	3.17
RBFOX1	3.63	4.12	3.27

BPIFA4P	3.63	4.37	3.21
HIST1H4L	3.63	4.60	2.84
NOC2L	3.63	4.37	2.91
OTTHUMG00000156394	3.63	3.99	3.07
OTTHUMG00000168428	3.63	4.28	3.04
PTPRZ1	3.63	5.29	2.91
OTTHUMG00000155789	3.63	4.03	2.99
SPATA3	3.62	4.09	3.09
TMIGD2	3.62	3.95	3.31
LOC100128264	3.62	4.31	3.30
C18orf61	3.62	4.10	3.28
LOC728537	3.62	4.15	3.07
SYN2	3.62	4.29	3.01
GAS2	3.62	4.09	3.13
LCE2B	3.62	4.82	3.03
OR2V1	3.62	4.07	2.90
TMEM170B	3.62	3.89	3.30
C3orf80	3.62	4.14	3.26
NPY	3.62	4.04	3.31
ZNF714	3.62	3.94	2.71
LOC100505875	3.62	4.39	3.07
DAW1	3.62	3.89	3.11
SYT4	3.62	4.11	2.97
NTN5	3.62	3.82	3.24
OTTHUMG00000017032	3.62	4.02	3.30
OTTHUMG00000159050	3.62	4.21	3.25
LOC100507224	3.62	4.46	2.46
LOC100506810	3.62	3.82	3.35
POU5F1B	3.62	4.14	2.95
RNA5SP30	3.62	4.44	2.94
RNA5SP491	3.62	3.89	3.19
ARL5C	3.62	4.12	3.34
OTTHUMG00000017159	3.62	4.06	3.02
KRT38	3.62	4.49	3.16
MIR1185-2	3.62	4.26	2.64
LOC400891	3.62	4.07	3.31
LOC91548	3.62	4.36	3.23
OTTHUMG00000164849	3.62	4.21	3.09
LOC285000	3.62	3.89	3.31
SLFN12L	3.62	4.09	3.24
HCFC1-AS1	3.62	4.02	3.05
LOC100128253	3.62	3.84	3.39
OTTHUMG00000034985	3.62	3.97	3.26
MIR4325	3.62	4.72	2.81
IL33	3.62	4.10	3.13
OTTHUMG00000164213	3.62	4.08	3.04
KIAA1239	3.62	4.02	3.02
SPANXN4	3.62	4.03	3.04
OTTHUMG00000008374	3.62	4.52	2.51

MIR601	3.62	4.60	2.95
OR2W1	3.62	4.01	3.16
OTTHUMG00000153380	3.61	4.13	2.93
RNASE9	3.61	3.97	3.28
OTTHUMG00000172166	3.61	4.20	3.08
MIR223	3.61	3.84	3.30
TMPRSS12	3.61	3.91	3.31
CD3E	3.61	4.30	3.12
IVL	3.61	4.08	3.09
KIF14	3.61	4.27	3.17
UGT1A9	3.61	4.08	3.20
CECR3	3.61	4.30	2.87
LINC00543	3.61	4.08	3.22
C1orf87	3.61	3.99	3.23
SNORA26	3.61	4.30	3.21
GFI1	3.61	4.07	2.85
TNFSF18	3.61	3.91	3.07
HOXB-AS2	3.61	4.05	2.97
OTTHUMG00000066730	3.61	3.94	2.98
CDC25C	3.61	3.97	3.29
PRICKLE2-AS2	3.61	4.30	3.38
MYO1A	3.61	3.90	3.47
OTTHUMG00000163155	3.61	4.10	3.28
AQP7P1	3.61	4.45	2.90
MS4A4E	3.61	4.88	2.75
MIR219-2	3.61	4.29	2.73
LOC100130548	3.61	4.20	3.23
LOC100652931	3.61	4.41	3.05
LOC442497	3.61	4.22	3.15
GCM1	3.61	3.85	3.13
RGS4	3.61	4.20	3.15
EIF5AL1	3.61	4.08	2.87
OTTHUMG00000171909	3.61	4.09	3.14
OTTHUMG00000162820	3.61	4.30	2.51
TMEM212-AS1	3.61	4.16	3.15
FLJ43585	3.61	3.97	3.41
OTTHUMG00000007957	3.61	4.03	3.15
FLJ26850	3.61	4.03	3.13
OTTHUMG00000182110	3.61	4.08	3.13
ATP1B3-AS1	3.61	4.29	3.20
PRSS58	3.61	3.92	3.37
TMEM102	3.61	4.35	2.98
DKFZp564H213	3.61	3.98	3.14
TRBV6-7	3.61	4.20	3.27
MIR573	3.61	4.51	2.68
TTC3-AS1	3.61	4.06	3.15
MAGEC3	3.61	3.80	3.45
CXorf28	3.61	4.43	3.19
OTTHUMG00000171092	3.61	4.12	3.22

NCAM2	3.61	3.88	3.35
C1orf186	3.60	4.36	3.03
MARVELD3	3.60	3.85	3.19
OTTHUMG00000158832	3.60	4.15	3.29
FETUB	3.60	4.05	3.20
OTTHUMG00000169106	3.60	4.10	2.93
KRT2	3.60	4.35	3.31
CYorf17	3.60	4.15	3.22
LINC00356	3.60	4.18	3.27
TRIM64B	3.60	3.97	3.11
LINC00694	3.60	3.95	3.16
LOC730338	3.60	3.86	3.32
OTTHUMG00000155390	3.60	4.27	3.07
FLJ45256	3.60	3.86	3.37
CCR4	3.60	4.14	3.07
ASCL2	3.60	4.05	3.11
MIR3164	3.60	4.21	3.28
EML5	3.60	4.06	3.32
ANKRD20A5P	3.60	4.58	2.68
FNDC7	3.60	4.37	3.07
OTTHUMG00000014234	3.60	4.12	3.23
SPATA31D1	3.60	4.28	2.91
CACNA1S	3.60	4.05	3.25
KRTAP2-2	3.60	4.36	2.75
NCALD	3.60	3.93	3.14
SMCP	3.60	4.19	3.30
E2F6	3.60	3.97	3.32
OTTHUMG00000017551	3.60	4.04	3.14
LYZL6	3.60	4.31	3.03
LINC00929	3.60	4.37	3.19
WNK3	3.60	4.08	3.08
LOC100507221	3.60	3.98	2.92
LYRM5	3.60	4.10	3.11
APOBEC4	3.60	4.06	3.20
TRBV2	3.60	4.02	3.30
LOC151484	3.60	4.05	3.15
CACNA1C-AS3	3.60	4.36	3.18
EPB41L4B	3.60	4.05	3.30
ASTL	3.60	3.75	3.22
MIR1260B	3.60	4.37	3.12
HFE2	3.60	3.90	3.10
MIR31HG	3.60	4.79	2.57
IQCH	3.60	3.76	3.46
NLRP9	3.60	4.23	3.04
PCDHA11	3.60	3.78	3.47
OTTHUMG00000164265	3.60	4.07	2.79
OTTHUMG00000166054	3.60	4.34	3.10
ZNRF3	3.60	4.06	3.29
OTTHUMG00000022289	3.60	3.81	3.30

IQUB	3.60	3.76	3.40
OTTHUMG00000160859	3.60	4.80	2.02
NFE2	3.60	4.19	3.16
OTTHUMG00000016932	3.60	4.05	3.17
MGC27382	3.59	4.41	3.23
LOC401286	3.59	4.36	3.01
PCDH8	3.59	3.90	3.23
SPOCK3	3.59	4.24	3.06
RNA5SP175	3.59	3.89	3.12
GIN1	3.59	4.57	3.13
LNX1-AS2	3.59	4.33	3.20
SDIM1	3.59	4.18	3.21
LINC00626	3.59	4.54	3.18
LOC730441	3.59	4.22	3.45
C1orf173	3.59	4.35	3.01
KLHL1	3.59	4.20	3.16
COL4A6	3.59	4.10	2.99
LOC285419	3.59	4.10	3.01
SLAMF6	3.59	3.97	3.16
CXorf64	3.59	3.95	3.16
DNTT	3.59	3.94	3.25
RIMBP2	3.59	3.97	3.29
LOC284240	3.59	3.99	3.15
EMX2	3.59	3.85	3.19
SYT16	3.59	3.88	3.33
ZAR1L	3.59	4.00	3.12
OTTHUMG00000166335	3.59	4.00	3.09
DEC1	3.59	3.89	3.36
LOC100505633	3.59	4.06	3.05
PGBD5	3.59	4.03	3.22
CARTPT	3.59	4.08	3.01
LOC100507642	3.59	3.97	3.26
OTTHUMG00000171799	3.59	3.89	3.36
LINC00208	3.59	3.92	3.33
EPHX4	3.59	3.96	3.07
PEX5L	3.59	4.13	3.14
PABPN1L	3.59	3.88	3.06
AJAP1	3.59	3.91	3.34
KBTBD8	3.59	3.95	3.19
SLC25A52	3.59	4.48	3.08
LOC100132077	3.59	3.95	3.10
CYP11A1	3.59	4.12	3.32
OTTHUMG00000170677	3.59	4.01	2.93
MIR4661	3.59	4.10	2.94
MGC2889	3.59	4.15	3.07
WDR64	3.59	3.91	3.24
OTTHUMG00000161704	3.59	4.32	3.12
SKOR1	3.59	3.94	3.35
CBY3	3.58	4.01	3.21

LOC401312	3.58	4.22	2.98
TRIM55	3.58	4.32	3.00
ASH1L-IT1	3.58	4.04	3.23
PADI1	3.58	4.00	3.30
DAPL1	3.58	4.45	3.06
GRXCR2	3.58	4.24	3.05
PPP2R2B	3.58	4.12	3.33
LOC100289333	3.58	3.83	3.26
TRPM3	3.58	4.08	3.13
OTTHUMG00000140195	3.58	4.19	3.20
RNU5E-1	3.58	4.13	3.06
OTTHUMG00000021379	3.58	3.89	3.37
OTTHUMG00000008994	3.58	4.02	3.18
KCNJ3	3.58	3.97	3.19
OTTHUMG00000180720	3.58	4.00	3.07
CEACAM4	3.58	3.94	3.11
LOC100652736	3.58	3.91	3.06
OTTHUMG00000150950	3.58	4.04	3.21
PVRL3-AS1	3.58	3.86	3.23
C4orf26	3.58	3.92	3.26
BSPH1	3.58	4.19	3.15
OTTHUMG00000066367	3.58	3.94	2.83
GPR151	3.58	4.12	3.26
LINC00552	3.58	4.11	2.94
LINC00240	3.58	4.31	3.09
OTTHUMG00000031891	3.58	4.25	3.19
OTTHUMG00000162061	3.58	4.09	2.96
ANKRD20A11P	3.58	4.36	2.73
GPR115	3.58	4.16	3.23
CCDC141	3.58	4.08	3.06
OTTHUMG00000153631	3.58	3.83	3.27
OTTHUMG00000022458	3.58	4.10	3.10
ERMN	3.58	4.12	3.26
LOC100506647	3.58	4.15	3.06
RNA5SP422	3.58	5.24	2.75
ZNF749	3.58	4.15	3.36
OTTHUMG00000037424	3.58	4.14	3.00
TJP3	3.58	3.98	3.14
HS3ST5	3.58	4.23	2.99
MIR635	3.58	4.49	2.43
FTO-IT1	3.58	4.03	3.15
LOC100128988	3.58	4.19	3.19
LOC100506098	3.58	3.91	3.09
MOSPD1	3.58	4.36	3.05
OR6B2	3.58	4.06	2.98
CENPQ	3.58	3.95	3.21
OTTHUMG00000018050	3.58	4.07	3.10
PCED1B-AS1	3.58	4.18	3.09
MIR302B	3.57	3.90	3.26

ARL11	3.57	3.83	3.06
ADAM30	3.57	4.01	2.98
OTTHUMG00000154784	3.57	3.96	3.02
OTTHUMG00000162104	3.57	4.16	3.23
HYLS1	3.57	3.90	3.14
ATP2C2	3.57	4.00	3.38
SLC8A1-AS1	3.57	4.48	3.07
LOC100131174	3.57	4.17	2.36
NXPE1	3.57	3.91	3.12
RNU6-76P	3.57	4.28	3.12
SPAG17	3.57	4.08	2.94
KIF18A	3.57	4.04	3.11
LOC400940	3.57	3.88	3.22
SLC10A6	3.57	4.44	3.20
OTTHUMG00000009684	3.57	4.29	3.05
OTTHUMG00000155998	3.57	4.02	3.10
LOC613126	3.57	4.00	3.19
KLHL4	3.57	4.42	3.19
MS4A3	3.57	4.19	3.14
DNAH3	3.57	4.07	3.01
ADCY10	3.57	4.06	3.10
MYO16	3.57	3.92	3.29
NECAB1	3.57	3.95	3.09
PSG4	3.57	4.47	2.55
KRTAP3-1	3.57	4.21	3.22
C9orf153	3.57	4.09	3.19
UGT2A3	3.57	3.85	3.20
GLRA1	3.57	4.05	2.94
POLQ	3.57	3.84	3.21
OTTHUMG00000163261	3.57	4.09	3.18
RPGRIP1	3.57	3.95	3.10
OTTHUMG00000155116	3.57	4.25	3.15
HRASLS2	3.57	4.14	2.88
HAPLN2	3.57	3.78	3.24
FAM26D	3.57	3.82	3.20
FBLIM1	3.57	3.88	3.04
ZG16	3.57	4.28	3.21
C11orf70	3.57	3.91	3.17
OTTHUMG00000032029	3.57	4.05	3.14
LAMP3	3.57	3.95	3.38
IL12B	3.57	4.07	3.19
OTTHUMG00000169446	3.57	4.12	3.04
SPZ1	3.57	4.08	3.11
SPINT4	3.57	4.20	2.66
A1CF	3.57	3.95	3.16
IRGC	3.57	4.24	2.99
TMEM207	3.57	3.90	3.18
KCNV2	3.57	4.07	3.13
SLC6A11	3.56	4.26	2.96

FLJ45743	3.56	4.09	2.90
TMPRSS15	3.56	3.96	3.25
SLC44A4	3.56	3.93	3.06
C6orf58	3.56	4.18	3.23
ANXA2P3	3.56	4.10	2.70
OTTHUMG00000016019	3.56	4.11	3.11
NR2E1	3.56	4.24	3.26
RNU4ATAC	3.56	4.59	2.22
CHRM3-AS1	3.56	3.88	3.22
HAL	3.56	4.15	2.98
IGKV3D-11	3.56	4.00	3.14
OTTHUMG00000155653	3.56	4.02	3.16
GLYAT	3.56	4.10	2.83
STPG2	3.56	4.11	3.08
SLC35F4	3.56	3.95	3.19
CYP26C1	3.56	3.87	3.35
OTTHUMG00000163048	3.56	4.16	3.06
TNFRSF9	3.56	4.09	2.90
OR6T1	3.56	4.64	3.05
SMC1B	3.56	4.19	2.98
OTTHUMG00000163315	3.56	3.99	3.07
BFSP2	3.56	4.19	3.08
DKFZP686I15217	3.56	3.93	3.18
SPAG6	3.56	4.03	3.23
USP46-AS1	3.56	3.95	3.01
GTSF1L	3.56	3.89	3.26
ZBTB20-AS3	3.56	4.01	3.02
PDZRN4	3.56	4.14	3.17
REP15	3.56	4.30	2.73
TEX28	3.56	4.27	3.19
SNORD126	3.56	4.21	2.87
TMEM151A	3.56	3.80	3.24
WDR96	3.56	3.85	3.10
CHL1	3.56	3.84	3.19
LOC100129726	3.56	4.00	3.29
AK5	3.56	4.46	3.13
MIR550A1	3.56	4.37	2.33
OTTHUMG00000151705	3.56	3.98	2.98
RNF175	3.56	4.20	2.97
ZNF280A	3.56	4.09	3.17
LINC00535	3.56	3.95	2.85
PRAMEF2	3.56	4.32	2.87
RNU7-67P	3.56	4.11	2.73
LRRC69	3.56	4.06	3.28
OTTHUMG00000161775	3.56	3.95	3.18
ADAMDEC1	3.56	4.24	3.18
OTTHUMG00000163515	3.56	4.40	3.21
HCG24	3.56	4.15	3.12
OTTHUMG00000172472	3.56	4.76	2.72

MIR3193	3.56	4.53	2.64
OTTHUMG00000042022	3.56	4.02	3.17
TFAP2B	3.56	3.92	3.02
OTTHUMG00000132305	3.56	4.22	3.24
GABRA3	3.56	4.11	3.20
PATE1	3.56	3.87	3.29
PEX11A	3.56	4.02	3.16
OTTHUMG00000160700	3.56	4.10	3.19
MC3R	3.56	4.07	2.61
MIR4715	3.55	4.09	2.82
TRIML1	3.55	4.23	3.26
OTTHUMG00000164853	3.55	4.33	3.02
OR52E4	3.55	4.28	3.05
LOC100506255	3.55	4.28	3.14
OTTHUMG00000153649	3.55	3.95	3.25
OTTHUMG00000020177	3.55	3.94	3.12
OTTHUMG00000162491	3.55	4.12	3.08
FDPSP2	3.55	4.30	2.74
LOC389705	3.55	4.20	2.88
PCLO	3.55	4.09	2.97
RNY4P23	3.55	4.33	2.76
MIR1288	3.55	4.40	3.15
OTTHUMG00000154620	3.55	4.69	2.70
H2BFM	3.55	4.07	3.23
ABCA12	3.55	3.85	3.25
OTTHUMG00000157151	3.55	4.00	3.06
KCNIP4-IT1	3.55	4.18	3.16
MAP2K4P1	3.55	4.23	3.00
DAPP1	3.55	4.13	3.18
OTTHUMG00000164760	3.55	4.36	2.93
CYP19A1	3.55	3.96	3.27
OTTHUMG00000170089	3.55	4.12	2.87
OR13F1	3.55	5.14	2.87
SLC10A5	3.55	4.13	3.16
LOC441179	3.55	3.95	2.92
LOC400654	3.55	4.13	3.26
RNA5SP354	3.55	4.31	3.10
ISL1	3.55	4.01	3.17
FAM9B	3.55	3.98	3.26
WNT5A	3.55	4.38	3.10
LOC100289650	3.55	4.10	2.87
OTTHUMG00000163011	3.55	3.98	3.22
FOLR3	3.55	3.89	3.26
MIR3170	3.55	4.54	2.51
CREG2	3.55	4.43	3.10
FRMPD1	3.55	3.95	3.17
MYOZ2	3.55	4.15	3.10
FLJ45831	3.55	4.04	3.08
TCF21	3.55	4.34	3.16

OTTHUMG00000168839	3.55	3.95	2.70
OTTHUMG00000177332	3.55	4.16	3.15
MSGN1	3.55	3.87	3.27
LOC100506472	3.55	4.36	2.85
OR4C5	3.55	4.06	2.98
OR5M1	3.55	4.31	2.79
OTTHUMG00000163986	3.55	3.78	3.32
LOC100506514	3.54	4.07	2.93
FAM84B	3.54	3.89	3.28
MIR3665	3.54	4.74	2.40
RN7SKP9	3.54	4.41	2.93
OR8B4	3.54	4.10	3.22
LOC100507261	3.54	4.44	2.95
MIR515-1	3.54	4.59	2.66
RNF208	3.54	3.73	3.19
DMRTA2	3.54	3.81	3.23
OTTHUMG00000156392	3.54	4.10	2.98
CLDN16	3.54	3.98	3.15
OTTHUMG00000159628	3.54	4.08	3.13
LCN10	3.54	4.24	3.09
MIR4789	3.54	4.04	2.86
IGF2BP3	3.54	3.96	3.18
DGCR9	3.54	3.97	2.75
LINC00361	3.54	4.29	3.26
MIR4653	3.54	4.21	1.95
GCM2	3.54	3.98	3.10
ZNF566	3.54	4.03	3.10
OTTHUMG00000017590	3.54	3.93	3.00
LINC00331	3.54	4.48	3.09
CAMK4	3.54	3.83	3.27
PAQR9	3.54	3.94	2.97
IL20RB-AS1	3.54	4.58	3.00
PNPLA5	3.54	4.16	3.20
CC2D2B	3.54	4.09	3.28
PARPBP	3.54	3.96	2.94
LINC00700	3.54	4.00	3.29
OTTHUMG00000180543	3.54	3.94	3.20
IL18RAP	3.54	3.89	3.37
OTTHUMG00000171831	3.54	4.38	2.78
C6orf99	3.54	3.94	3.35
RBM22P1	3.54	4.22	2.93
OTTHUMG00000164531	3.54	4.99	2.55
OTTHUMG00000179655	3.54	4.32	2.80
OTTHUMG00000179489	3.54	3.99	3.18
C10orf120	3.54	3.77	3.01
MIR670	3.54	4.10	3.15
WWC2-AS2	3.54	3.80	3.25
OTTHUMG00000151750	3.54	3.85	3.19
NRON	3.54	4.25	2.99

LINC00966	3.54	4.10	2.84
GPR32	3.54	3.87	3.11
TGM3	3.53	3.79	3.20
ASB4	3.53	4.37	2.97
MIR4496	3.53	3.92	3.36
ZSCAN31	3.53	3.83	2.86
FAM83F	3.53	4.12	3.17
OTTHUMG00000041155	3.53	4.11	3.13
LINC00524	3.53	4.06	3.06
OTTHUMG00000171853	3.53	4.39	2.80
LIN28A	3.53	4.08	3.13
ITCH-AS1	3.53	3.99	3.28
TRAJ4	3.53	4.50	2.73
LINC00892	3.53	3.99	2.75
TPH1	3.53	3.75	3.34
MIR215	3.53	4.56	2.87
RASA2-IT1	3.53	4.18	2.93
FAM186A	3.53	3.89	3.04
C1orf180	3.53	4.38	3.03
OR52I1	3.53	4.04	3.11
OTTHUMG00000017646	3.53	3.88	2.73
DPRXP4	3.53	4.43	2.51
OVOS2	3.53	5.26	2.82
LOC654841	3.53	3.93	3.25
CPS1-IT1	3.53	3.97	2.97
TP73	3.53	4.06	3.30
MIR181A2	3.53	3.82	3.20
LOC254099	3.53	3.79	3.17
STK31	3.53	3.85	3.06
OTTHUMG00000165266	3.53	4.09	3.15
OTTHUMG00000037125	3.53	4.47	2.72
DKFZp686L13185	3.53	3.85	3.18
SLC15A2	3.53	3.87	3.10
OTTHUMG00000162462	3.53	4.39	2.86
LOC728463	3.53	4.31	3.18
OTTHUMG00000162825	3.53	3.95	3.07
OTTHUMG00000162631	3.53	4.31	2.98
LOC285762	3.53	3.91	3.03
LRRC43	3.53	4.29	3.07
MIR378F	3.53	4.34	2.67
LINC00941	3.53	3.95	3.10
OTTHUMG00000041032	3.53	4.43	2.37
OTTHUMG00000020689	3.53	3.94	3.15
CST13P	3.53	3.90	3.23
LOC100996447	3.53	3.89	3.18
FAAH2	3.53	4.02	2.91
CCNE1	3.53	4.10	3.16
LOC100505903	3.53	4.03	3.17
IZUMO2	3.53	3.88	3.04

SLC17A6	3.53	4.24	3.09
OTTHUMG00000163746	3.53	4.01	3.08
SOX14	3.53	3.95	3.10
STRA8	3.52	4.01	3.25
MPC1L	3.52	4.07	2.94
OR2A12	3.52	4.10	3.27
OTTHUMG00000024097	3.52	3.96	3.21
KRT76	3.52	4.09	2.77
SNORD115-32	3.52	5.01	3.02
OTTHUMG00000014802	3.52	4.21	3.10
C6orf163	3.52	4.08	2.84
DCD	3.52	3.92	3.13
OTTHUMG00000170230	3.52	4.30	2.70
CDC14B	3.52	4.05	2.90
MIR323B	3.52	4.21	2.84
LINC00461	3.52	3.78	3.27
DMGDH	3.52	3.98	3.04
OTTHUMG00000055981	3.52	3.92	2.94
OTTHUMG00000168862	3.52	4.32	3.10
C9orf135-AS1	3.52	4.21	3.09
CSTF3-AS1	3.52	3.90	3.21
RNA5SP487	3.52	3.99	3.03
PTENP1	3.52	4.18	2.35
FOLH1	3.52	4.66	2.60
SORCS3-AS1	3.52	3.75	3.21
MIR30C2	3.52	4.49	3.00
C4orf51	3.52	3.90	3.05
CA6	3.52	4.05	3.14
NWD1	3.52	3.92	2.87
OTTHUMG00000161379	3.52	4.09	2.73
ZAR1	3.52	3.91	3.13
VPREB3	3.52	3.82	3.18
CCDC138	3.52	4.13	2.96
C13orf35	3.52	3.99	3.14
MAEL	3.52	3.75	3.23
ANKFN1	3.52	4.16	2.96
LOC340581	3.52	3.98	3.05
TTLL2	3.52	4.10	3.17
SYCP3	3.52	4.03	2.86
OTTHUMG00000020194	3.52	3.93	3.26
LOC440600	3.52	4.21	2.72
LOC285593	3.52	3.83	3.25
CRCT1	3.52	3.82	3.17
MIR518B	3.52	4.38	2.43
CST5	3.52	3.84	3.34
MIR4636	3.52	4.23	2.74
GATA3-AS1	3.52	3.82	3.38
OTTHUMG00000172229	3.52	3.96	3.07
SCGN	3.52	3.84	3.18

ALDOB	3.52	3.91	3.15
NPSR1	3.52	3.83	3.20
PGM5P2	3.52	4.01	2.95
VHLL	3.52	3.97	3.07
MIR373	3.52	4.35	2.45
TEX12	3.52	4.13	3.12
FPR2	3.52	4.14	3.15
LOC100505851	3.51	4.40	3.06
OTTHUMG00000020357	3.51	3.93	3.11
OTTHUMG00000153054	3.51	3.85	3.26
OR56A1	3.51	4.10	2.85
ALPK2	3.51	3.94	3.04
IDO1	3.51	4.08	3.05
OTTHUMG00000164287	3.51	4.03	2.93
IGFBP2	3.51	3.95	3.10
OTTHUMG00000152947	3.51	3.88	3.18
LOC100996249	3.51	4.19	3.02
XRCC6P5	3.51	3.70	3.14
GJA8	3.51	3.97	3.28
SSX7	3.51	4.61	2.99
LTF	3.51	4.34	3.05
UGT2B7	3.51	3.98	2.96
OTTHUMG00000154724	3.51	3.97	3.06
OTTHUMG00000019429	3.51	3.87	3.07
OTTHUMG00000161436	3.51	4.03	3.10
OTTHUMG00000163319	3.51	4.01	3.34
OTTHUMG00000151756	3.51	4.28	2.93
MIR4755	3.51	3.91	3.13
LOC100507175	3.51	3.76	3.05
LOC440335	3.51	4.16	2.63
ELOVL2	3.51	3.87	3.02
ANKRD32	3.51	3.96	3.01
OTTHUMG00000161395	3.51	4.35	2.83
OTTHUMG00000153181	3.51	4.07	2.98
DEFB104A	3.51	4.37	2.98
PCAT4	3.51	4.15	2.91
CATSPERD	3.51	4.05	3.05
PRRG2	3.51	3.81	3.28
OTTHUMG00000166089	3.51	4.08	3.13
SNORD37	3.51	4.45	2.74
INSL4	3.51	4.21	2.89
CRYM-AS1	3.51	4.18	3.02
SPATA17	3.51	3.79	3.21
MDH1B	3.51	4.35	3.10
OTTHUMG00000160129	3.51	3.86	2.99
EVX1-AS	3.51	4.18	3.05
OR2T35	3.51	4.00	2.95
FCRL4	3.51	3.98	3.18
OTTHUMG00000042097	3.51	3.84	3.18

THSD7B	3.51	4.07	3.01
OTTHUMG00000169041	3.51	4.44	2.52
LINC00939	3.51	3.86	3.06
OTTHUMG00000007952	3.51	4.01	3.13
DUOXA2	3.51	4.17	2.86
MAL2	3.51	4.12	2.84
CALCB	3.51	4.20	3.02
PSG8	3.51	4.25	2.64
C4BPB	3.51	3.76	3.17
OTTHUMG00000165323	3.51	3.92	3.16
OTTHUMG00000171694	3.51	4.62	2.96
IMMP2L-IT1	3.51	4.02	3.09
OTTHUMG00000162318	3.51	3.93	2.92
RPE	3.50	4.01	2.27
LOC154092	3.50	3.92	3.08
LOC100288198	3.50	3.87	3.16
OTTHUMG00000164236	3.50	3.97	3.06
LOC340515	3.50	3.95	3.14
OTTHUMG00000170269	3.50	3.95	3.02
CLEC1B	3.50	4.12	3.10
TMEM71	3.50	3.93	2.90
HDC	3.50	3.99	2.84
HSD17B3	3.50	3.84	3.19
PDX1-AS1	3.50	3.88	3.15
PRR23C	3.50	4.46	2.61
DSCR9	3.50	3.74	3.17
COL6A5	3.50	3.94	3.14
OTTHUMG00000162379	3.50	3.96	3.27
EGR4	3.50	3.99	3.29
LOC100505784	3.50	4.24	3.01
CCDC63	3.50	3.96	2.90
OTTHUMG00000067508	3.50	4.03	2.90
LOC441025	3.50	3.81	2.99
OTTHUMG00000040631	3.50	3.84	3.05
PRSS3P3	3.50	4.31	2.99
FEZF2	3.50	3.84	3.18
SLC22A25	3.50	4.37	2.67
C1QL2	3.50	4.24	3.05
ST6GALNAC3	3.50	4.01	2.99
KRTAP20-3	3.50	3.86	2.63
OR1K1	3.50	4.01	3.13
OTTHUMG00000171107	3.50	3.88	3.25
TRBV5-4	3.50	4.56	2.16
MTTP	3.50	3.96	3.21
TAC1	3.50	3.97	3.21
ESRP1	3.50	3.76	3.34
MIR340	3.50	3.89	3.09
DCAF4L1	3.50	4.04	2.77
ASIC1	3.50	3.88	3.14

OTTHUMG00000163017	3.50	4.33	2.85
OTTHUMG00000181960	3.50	4.30	2.94
SETD9	3.50	4.00	2.82
NAA11	3.50	3.97	2.93
OTTHUMG00000163525	3.50	3.96	3.04
DSCAM	3.50	3.87	2.91
TBC1D3P5	3.50	4.51	2.81
OTTHUMG00000169139	3.50	3.80	3.25
OTTHUMG00000132240	3.50	3.95	2.85
OTTHUMG00000154038	3.50	4.13	3.06
BAI3	3.50	4.22	3.00
C3orf67	3.50	4.00	3.09
OTTHUMG00000163997	3.49	3.73	3.32
STAR	3.49	3.95	2.99
FLJ46906	3.49	4.27	3.08
RSPH10B	3.49	4.07	2.83
OTTHUMG00000164272	3.49	3.86	3.17
TMEM252	3.49	4.08	3.07
CIB4	3.49	4.03	2.84
ZNF77	3.49	3.96	3.06
BHMT	3.49	3.88	3.24
BRS3	3.49	4.42	3.17
ACTR3C	3.49	4.03	2.82
RP1	3.49	3.89	3.27
CES4A	3.49	4.13	2.86
LINC00882	3.49	4.05	3.24
MAPK4	3.49	4.06	3.01
NEK5	3.49	3.83	3.28
RBM44	3.49	3.96	3.19
TRMT10A	3.49	3.76	3.17
CKMT1A	3.49	4.20	2.39
UPP2	3.49	4.03	3.14
OTTHUMG00000046152	3.49	4.18	2.77
DNMT3L	3.49	3.90	3.16
LINC00971	3.49	4.18	3.14
CLCA2	3.49	3.71	3.21
OTTHUMG00000156043	3.49	4.05	3.08
ATRNL1	3.49	4.03	2.72
OTTHUMG00000018868	3.49	4.07	3.06
CEACAM21	3.49	3.79	2.75
LOC100507311	3.49	3.98	2.92
OTTHUMG00000161773	3.49	4.25	3.03
USP27X	3.49	3.98	3.17
ESP33	3.49	4.20	2.93
OTTHUMG00000169719	3.49	4.30	3.01
IFIT1B	3.49	4.22	2.57
MAGI2-IT1	3.49	4.09	3.08
CLDN24	3.49	3.91	2.91
LOC158435	3.49	3.82	3.18

LRRC38	3.49	3.91	2.85
WT1-AS	3.49	3.97	3.05
OTTHUMG00000159391	3.48	4.59	2.61
ANXA10	3.48	3.92	2.90
OTTHUMG00000002437	3.48	3.89	3.01
BLM	3.48	3.74	3.17
OTTHUMG00000151394	3.48	4.52	2.88
HCRTR2	3.48	3.88	3.13
HMX2	3.48	3.70	3.17
LOC100996416	3.48	4.37	2.87
CALML3-AS1	3.48	4.03	2.90
SPATA42	3.48	4.04	3.03
CPB1	3.48	3.96	3.06
OTTHUMG00000183861	3.48	3.95	3.18
FLJ42969	3.48	3.86	2.76
GPR141	3.48	3.72	3.24
LOC652993	3.48	3.93	3.07
OTTHUMG00000024048	3.48	3.92	3.04
OTTHUMG00000163224	3.48	3.93	3.06
C1orf177	3.48	3.93	3.17
MIR3937	3.48	4.36	2.86
LOC100507194	3.48	4.20	2.64
OTTHUMG00000169447	3.48	4.20	2.16
PRSS51	3.48	3.98	3.02
LOC100128573	3.48	3.95	3.14
OTTHUMG00000034564	3.48	3.95	2.99
LOC100128993	3.48	3.99	3.14
XIRP2	3.48	3.84	3.16
ACOT1	3.48	4.38	2.85
LINC00652	3.48	3.89	3.14
SV2C	3.48	3.87	3.03
HS6ST2	3.48	3.99	2.98
LINC00944	3.48	3.99	2.96
GLYATL3	3.48	3.93	3.12
PATE4	3.48	4.33	3.09
CD1B	3.48	4.06	3.08
OTTHUMG00000161215	3.48	4.00	2.78
OTTHUMG00000163136	3.48	4.05	2.98
TSGA10	3.48	3.74	3.07
OTTHUMG00000020097	3.48	4.27	3.17
OTTHUMG00000161670	3.48	3.78	2.94
GPRC6A	3.48	3.71	3.26
LOC100996654	3.48	3.71	3.18
OTTHUMG00000167225	3.48	3.97	3.07
TRDV1	3.48	3.79	3.16
MIR527	3.48	4.24	3.00
GPR82	3.48	4.03	3.23
HEXA-AS1	3.48	4.09	2.99
LOC440117	3.48	3.78	3.19

ARMC4	3.48	4.00	3.24
LATS2-AS1	3.48	4.46	2.69
LINC00506	3.47	4.07	2.91
OTTHUMG00000156487	3.47	4.28	2.72
LOC148709	3.47	4.30	3.00
NRG3	3.47	3.89	2.85
RNA5SP455	3.47	4.21	2.42
PROK2	3.47	3.91	3.28
LOC100507506	3.47	3.93	3.00
OTTHUMG00000151744	3.47	3.97	3.23
LELP1	3.47	4.18	3.06
LOC100130476	3.47	3.93	2.82
TMED6	3.47	3.83	3.15
VSX1	3.47	3.97	2.98
C15orf26	3.47	3.95	2.90
OTTHUMG00000164488	3.47	3.87	3.01
TMEM169	3.47	3.95	3.07
OLFM4	3.47	3.86	2.95
MIR4713	3.47	4.29	2.47
COL6A4P1	3.47	3.91	2.83
VNN1	3.47	3.77	3.13
TRIM51EP	3.47	4.06	2.78
OTTHUMG00000155482	3.47	3.98	3.11
LAMP5	3.47	3.97	3.12
STPG2-AS1	3.47	3.84	3.07
HES7	3.47	4.31	3.01
MPZL3	3.47	3.88	2.98
OTTHUMG00000020036	3.47	3.94	2.53
NEUROG2	3.47	3.81	2.95
PRKG1-AS1	3.47	4.38	2.81
MIR550B1	3.47	5.00	2.82
OTTHUMG00000165031	3.47	4.02	2.56
LINC00269	3.47	4.16	3.16
DNAJC5G	3.47	3.71	3.13
PRSS21	3.47	3.83	3.16
GCNT6	3.47	4.05	3.13
CALN1	3.47	3.82	2.98
CYP24A1	3.47	3.76	3.24
MRGPRX4	3.47	4.00	3.01
OTTHUMG00000164519	3.46	4.00	3.25
GMNC	3.46	4.07	2.90
OTTHUMG00000170253	3.46	4.12	2.99
TSHB	3.46	3.82	2.75
OTTHUMG00000160643	3.46	3.67	3.29
KRTAP4-12	3.46	4.60	2.73
OTTHUMG00000171848	3.46	3.91	3.16
PLA2G3	3.46	3.79	3.21
OTTHUMG00000020176	3.46	3.81	2.83
SNAP91	3.46	3.78	3.06

LOC100505887	3.46	4.19	3.14
GPR61	3.46	3.90	3.09
OTTHUMG00000168454	3.46	4.17	3.13
LOC100507172	3.46	4.38	2.76
OTTHUMG00000151447	3.46	3.84	3.03
LOC645188	3.46	3.90	3.01
NHLH2	3.46	3.82	3.17
SLC30A10	3.46	4.08	3.16
BDKRB1	3.46	3.78	3.28
LINC00570	3.46	3.82	2.98
MIR4277	3.46	3.98	2.94
PPP1R1C	3.46	3.94	2.76
MON1A	3.46	3.86	3.11
OTTHUMG00000066033	3.46	3.90	2.98
CAMK1G	3.46	4.04	2.86
OTTHUMG00000015537	3.46	3.99	3.12
LOC100130172	3.46	3.83	3.03
PCGEM1	3.46	3.77	3.05
WDR49	3.46	3.65	3.17
RTP4	3.46	4.19	3.08
OTTHUMG00000037823	3.46	3.84	3.24
LOC100996455	3.46	3.91	2.91
PAX6	3.46	3.99	3.09
LOC100506161	3.46	3.83	2.91
OR52A1	3.46	3.95	2.89
MAR11	3.46	4.47	2.89
IL13RA2	3.46	4.48	2.13
ZIC3	3.46	3.77	2.95
LOC100507109	3.46	4.26	2.92
OTTHUMG00000016054	3.46	3.86	3.02
TAS2R10	3.46	4.12	3.04
C20orf187	3.46	4.23	3.03
LOC641367	3.46	4.17	2.78
SAMSN1	3.46	3.83	3.22
LINC00308	3.46	3.90	2.90
ZNF285	3.46	4.05	3.04
LOC441167	3.46	3.76	3.11
OTTHUMG00000019692	3.46	3.84	3.10
LOC440518	3.46	3.87	2.96
LOC100288619	3.45	3.95	3.13
KAAG1	3.45	3.95	2.79
CDCA7	3.45	4.02	3.15
BTLA	3.45	3.98	3.05
IRF4	3.45	3.77	3.14
AKAP3	3.45	3.83	2.89
OTTHUMG00000164977	3.45	3.75	2.59
OTTHUMG00000157216	3.45	3.89	2.96
REG3A	3.45	4.24	3.05
CXCL10	3.45	4.11	2.84

CSN1S2BP	3.45	3.97	3.01
LPO	3.45	3.76	3.06
LOC100506497	3.45	3.88	3.03
C12orf74	3.45	3.98	3.02
TAS2R60	3.45	3.84	3.06
APCS	3.45	4.08	2.97
OR10A2	3.45	4.10	2.93
LOC100130301	3.45	4.12	3.06
OTTHUMG00000169048	3.45	3.89	3.06
OTTHUMG00000171950	3.45	4.10	2.93
RRAD	3.45	3.92	3.09
PSG9	3.45	3.91	2.58
OTTHUMG00000162669	3.45	4.08	2.83
C8A	3.45	3.90	2.73
LINC00659	3.45	3.70	3.14
TSSK1B	3.45	4.02	2.89
GH1	3.45	4.05	2.54
OTTHUMG00000166086	3.45	3.74	3.06
LINC00567	3.45	3.87	3.09
LOC100506944	3.45	4.10	2.83
OTTHUMG00000164397	3.45	3.82	3.00
OR5AS1	3.45	4.32	3.18
BHLHE40-AS1	3.45	3.76	3.09
LOC389033	3.45	4.54	2.47
ONECUT1	3.45	3.73	3.26
OTTHUMG00000166475	3.45	3.73	3.00
GYS2	3.45	3.64	3.06
GDPD2	3.44	3.72	3.12
KLLN	3.44	3.89	3.09
OTTHUMG00000013154	3.44	3.77	3.03
MIR615	3.44	3.81	3.11
EFTUD1P1	3.44	4.11	2.84
LOC151475	3.44	3.88	3.15
OTTHUMG00000150310	3.44	3.96	3.08
C20orf78	3.44	3.70	3.21
BVES-AS1	3.44	4.11	2.86
MIR1179	3.44	4.57	2.81
RNA5SP167	3.44	4.28	2.25
OTTHUMG00000163264	3.44	4.02	2.83
OTTHUMG00000152414	3.44	4.00	3.09
CHRM2	3.44	3.74	3.16
LINC00161	3.44	4.15	3.04
OTTHUMG00000152839	3.44	3.94	2.76
MIR4303	3.44	3.71	3.18
CLDN10	3.44	3.78	3.10
OTTHUMG00000170726	3.44	4.06	2.86
C3orf55	3.44	3.94	3.06
RLN1	3.44	4.33	2.71
HTR2A-AS1	3.44	4.05	3.19

OTTHUMG00000162997	3.44	3.74	3.12
OTTHUMG00000167434	3.44	3.92	3.05
ABP1	3.44	3.90	2.91
MIR516B1	3.44	4.18	2.96
OR1F1	3.44	4.23	2.52
LINC00691	3.44	3.82	3.07
LEKR1	3.44	3.73	3.20
LOC100506157	3.44	3.83	3.09
OTTHUMG00000171401	3.44	4.12	2.85
OTTHUMG00000014288	3.44	4.02	2.82
SNORD114-29	3.44	5.43	2.00
TPI1P2	3.44	4.14	2.87
PPP1R2P9	3.44	3.81	3.00
OTTHUMG00000172167	3.44	3.76	3.16
MCF2L2	3.44	3.64	3.28
MIR4633	3.43	3.99	2.91
OTTHUMG00000150931	3.43	3.97	3.05
OTTHUMG00000170065	3.43	4.50	2.90
P2RX6P	3.43	4.07	2.74
OTTHUMG00000034422	3.43	3.86	2.70
RPH3A	3.43	3.77	3.06
OTTHUMG00000180563	3.43	3.88	2.61
OTTHUMG00000020283	3.43	3.61	3.07
FGF20	3.43	3.99	3.05
ANKRD60	3.43	4.10	2.95
FKBP9L	3.43	4.09	3.12
MIR4773-1	3.43	4.15	2.84
CNGA4	3.43	3.72	3.23
LOC284801	3.43	3.82	2.90
CAMTA1-IT1	3.43	4.16	2.54
IGDCC4	3.43	3.77	3.17
PURG	3.43	3.80	3.02
FREM2	3.43	4.04	2.70
SALL3	3.43	3.97	3.03
C11orf85	3.43	3.77	2.96
TYR	3.43	3.78	2.93
ARIH2OS	3.43	3.83	2.93
OTTHUMG00000162703	3.43	3.94	2.98
LINC00502	3.43	3.79	3.12
KCNE2	3.43	3.82	3.19
PIP5K1P1	3.43	4.09	3.09
OTTHUMG00000159264	3.43	4.15	2.53
OTTHUMG00000156033	3.43	3.80	2.87
OTTHUMG00000166430	3.43	3.89	2.87
OTTHUMG00000166129	3.43	3.90	3.04
TRBV10-2	3.43	4.34	2.22
MIR4717	3.43	3.95	2.80
ILDR2	3.43	3.70	3.06
RNA5SP367	3.43	4.01	2.85

C5orf47	3.43	4.04	2.94
HHLA1	3.43	3.82	3.15
SLC30A8	3.43	3.64	3.21
OTTHUMG00000163601	3.43	4.03	3.03
ARHGEF7-AS1	3.43	4.13	3.02
KRT40	3.43	3.81	3.06
RNA5SP61	3.43	4.15	2.65
OTTHUMG00000021152	3.43	4.52	2.94
CXCR5	3.43	3.99	3.07
NRSN1	3.43	4.22	3.17
OPRM1	3.42	3.78	3.12
MIR3184	3.42	4.05	2.77
LINC00469	3.42	3.78	2.94
OTTHUMG00000162000	3.42	3.96	2.60
TOMM20L	3.42	3.90	3.11
TM4SF1-AS1	3.42	3.85	2.91
OTTHUMG00000165349	3.42	3.90	2.99
HMGN2P46	3.42	3.75	3.13
LINC00708	3.42	3.87	3.08
MIR592	3.42	3.91	2.90
LINC00942	3.42	3.84	2.85
MURC	3.42	3.80	3.05
OR4K13	3.42	4.74	2.88
ARL13A	3.42	3.74	3.18
KIF2C	3.42	3.88	3.07
TPI1P3	3.42	4.22	2.50
OTTHUMG00000161032	3.42	3.96	2.58
AGBL5-IT1	3.42	4.20	2.62
OTTHUMG00000152269	3.42	3.94	2.88
TAS2R40	3.42	4.18	2.79
RNA5SP416	3.42	4.33	2.82
ADAMTS20	3.42	4.10	3.13
OTTHUMG00000041461	3.42	3.93	2.88
KRT23	3.42	3.68	3.12
EPHA1-AS1	3.42	3.63	2.99
MAGEA11	3.42	3.72	3.13
RNA5SP442	3.42	4.31	1.99
OTTHUMG00000168648	3.42	3.93	3.01
LOC340508	3.42	3.94	3.02
CPXCR1	3.42	4.04	2.73
OTTHUMG00000171475	3.42	3.89	2.92
OTTHUMG00000160052	3.42	3.90	3.05
OTTHUMG00000155024	3.42	4.02	3.20
OTTHUMG00000173195	3.42	4.10	2.70
TNR-IT1	3.42	3.88	3.07
RPP40	3.42	3.82	3.12
LOC728671	3.42	3.68	3.12
CYP3A4	3.42	3.75	2.96
OTTHUMG00000156009	3.42	3.68	3.16

KCNJ16	3.42	3.81	3.19
OTTHUMG00000164235	3.42	3.65	3.07
OTTHUMG00000060108	3.42	3.82	3.07
OTTHUMG00000014150	3.42	4.31	3.04
ASCL3	3.42	3.92	3.11
TDGF1P3	3.42	3.81	3.13
GPR63	3.42	4.07	2.71
KRT80	3.42	4.18	3.20
RESP18	3.42	3.78	3.04
KMO	3.42	3.81	2.89
VCX3B	3.42	4.19	2.84
DNAH14	3.42	4.00	3.09
SLC8A1	3.42	4.25	3.06
CNTN4	3.42	3.69	3.13
OTTHUMG00000156608	3.42	3.84	3.01
WEE2-AS1	3.42	3.88	3.08
FOXB1	3.41	3.75	2.82
SLC34A2	3.41	3.67	3.02
RASEF	3.41	3.99	2.94
STK4-AS1	3.41	3.93	3.16
PAPL	3.41	3.64	3.00
OTOL1	3.41	3.75	3.04
DKK4	3.41	3.82	2.98
TC2N	3.41	4.19	3.07
LINC00303	3.41	4.05	2.90
TMEM72-AS1	3.41	3.75	2.65
MAGEE2	3.41	3.65	3.13
LOC440895	3.41	4.33	2.51
OTTHUMG00000166913	3.41	4.77	2.58
MIR3913-1	3.41	3.94	2.67
LOC100996339	3.41	3.83	2.88
IRAK1BP1	3.41	3.69	3.20
PHEX	3.41	4.05	2.72
RIMS2	3.41	3.95	2.93
LOC100505811	3.41	4.06	2.82
CXCL13	3.41	3.91	2.92
SFTPA1	3.41	3.84	3.18
DCAF12L2	3.41	3.99	2.91
CSAG4	3.41	4.02	2.45
LOC100996355	3.41	3.87	3.20
CYSLTR2	3.41	3.91	2.99
LOC388499	3.41	3.93	2.63
TMEM51	3.41	4.11	2.93
OTTHUMG00000158500	3.41	4.25	2.30
OTTHUMG00000021599	3.41	3.79	2.90
PROB1	3.41	3.74	3.06
GUCY2F	3.41	4.02	3.00
LINC00309	3.41	3.84	2.86
AKAP14	3.41	3.99	3.07

LOC644961	3.41	3.73	3.16
FAM92A1P2	3.41	3.73	3.20
OTTHUMG00000153047	3.41	3.96	2.97
OR10X1	3.41	3.80	2.88
LOC440704	3.41	4.12	2.95
SMYD1	3.41	4.07	2.88
DIO1	3.41	3.63	2.98
MESTIT1	3.41	3.95	3.13
IRG1	3.40	3.73	2.87
OTTHUMG00000032807	3.40	4.10	2.77
GRIK1-AS2	3.40	3.72	3.03
KLRC4-KLRK1	3.40	4.35	2.67
WFDC9	3.40	4.01	3.14
TCERG1L	3.40	3.72	2.76
FAM83D	3.40	4.05	2.77
ANKRD18DP	3.40	3.98	2.76
CCR5	3.40	3.72	3.17
GZMB	3.40	4.05	3.01
OTTHUMG00000020547	3.40	3.92	3.06
MIR875	3.40	4.18	2.93
ESX1	3.40	3.64	3.29
KCNJ1	3.40	4.28	3.11
LINC00589	3.40	3.93	2.97
CELF2-AS2	3.40	4.02	2.68
COQ3	3.40	4.04	2.65
GAD1	3.40	3.89	3.05
MAPK8IP1	3.40	4.15	2.79
CHRM3-AS2	3.40	3.90	2.91
OTTHUMG00000171351	3.40	4.14	2.90
IL20	3.40	3.97	2.87
FLJ45974	3.40	3.58	3.08
OTTHUMG00000163846	3.40	4.18	2.62
OTTHUMG00000016839	3.40	4.07	2.72
FAM45B	3.40	3.69	3.15
ATP13A4-AS1	3.40	4.19	2.76
OTTHUMG00000033267	3.40	3.93	2.99
USP17L10	3.40	3.94	2.65
OTTHUMG00000015954	3.40	5.22	2.34
PP2672	3.40	4.39	2.72
OTTHUMG00000035812	3.40	3.74	3.00
THEG	3.40	3.78	3.00
OTTHUMG00000173008	3.40	4.03	2.59
RNA5SP409	3.40	4.04	2.56
MGC2848	3.40	3.88	2.94
LOC730179	3.40	3.76	3.02
TPD52L3	3.40	3.76	3.07
OTTHUMG00000154833	3.40	3.57	3.07
LRRC53	3.40	3.91	3.13
FLJ42200	3.40	3.75	2.82

CPHL1P	3.40	3.79	2.79
SP8	3.40	3.99	2.98
OTTHUMG00000169138	3.40	4.13	3.14
ZNF221	3.40	4.27	2.74
REG1B	3.40	3.67	3.17
MIR4319	3.40	3.79	2.62
OTTHUMG00000018052	3.40	3.84	3.00
C1orf210	3.40	3.98	3.14
SLC35G6	3.40	3.95	2.54
LOC643623	3.40	3.84	2.81
PRR19	3.39	3.69	3.15
OR2A25	3.39	4.08	2.13
FAM27B	3.39	4.03	2.95
LINC00391	3.39	3.74	2.88
LOC284648	3.39	3.82	2.87
OTTHUMG00000032716	3.39	4.18	2.91
DSCR8	3.39	3.71	3.02
GRM7-AS1	3.39	3.99	2.94
ATP12A	3.39	3.87	3.02
RNU6-74	3.39	4.61	1.74
OTTHUMG00000019052	3.39	4.11	2.91
LINC00457	3.39	3.90	3.17
OTTHUMG00000161887	3.39	3.67	3.05
EYA3-IT1	3.39	4.08	2.95
MIR3200	3.39	3.74	2.83
OTTHUMG00000040633	3.39	4.13	2.84
WDR72	3.39	3.78	2.98
KCNH7	3.39	3.75	2.98
EFCAB12	3.39	3.73	2.80
LALBA	3.39	4.35	3.05
TRIM50	3.39	4.20	2.73
LOC100131635	3.39	3.99	2.92
C10orf90	3.39	3.75	3.17
GIF	3.39	3.71	3.02
LINC00226	3.39	3.92	3.03
KRTAP9-3	3.39	3.90	3.01
PARD3-AS1	3.39	4.26	2.87
OTTHUMG00000152416	3.39	4.15	3.04
LINC00955	3.39	3.72	3.00
LINC00642	3.39	3.79	3.06
IGSF1	3.39	3.59	3.17
CAPN14	3.39	3.88	2.83
PPP1R27	3.39	3.74	2.91
OTTHUMG00000157218	3.39	3.78	2.80
OTTHUMG00000018941	3.39	4.04	2.94
CEACAM5	3.39	4.11	2.60
ITGB6	3.39	3.75	2.89
LOC100505718	3.39	3.78	3.00
TFF2	3.39	3.93	3.00

TCP11	3.39	4.06	2.89
OR14A16	3.39	3.94	2.99
KRTAP19-5	3.38	3.76	2.94
OR2D2	3.38	3.66	3.11
OTTHUMG00000172076	3.38	4.02	3.04
FLJ22763	3.38	3.65	2.99
OTTHUMG00000161726	3.38	3.61	3.13
OR51L1	3.38	4.15	2.93
OLIG2	3.38	3.64	3.16
LOC145845	3.38	3.94	3.06
LINC00927	3.38	3.76	3.12
DEFB121	3.38	3.92	2.79
OTTHUMG00000168485	3.38	4.07	3.14
UPK1B	3.38	4.01	2.95
OTTHUMG00000170886	3.38	3.78	2.99
LOC646778	3.38	3.74	2.95
GPR15	3.38	3.61	3.16
OTTHUMG00000164139	3.38	3.69	2.88
OTTHUMG00000172034	3.38	4.15	2.86
OTTHUMG00000161635	3.38	3.87	3.04
PA2G4P4	3.38	4.03	3.04
KRTAP1-4	3.38	3.81	2.87
OTTHUMG00000151723	3.38	3.65	3.12
OTTHUMG00000153160	3.38	3.82	2.90
CDKL3	3.38	3.65	3.10
LOC400794	3.38	3.70	3.09
DPY19L2P4	3.38	3.70	3.09
OTTHUMG00000164595	3.38	3.79	2.95
CYP2C8	3.38	4.31	2.83
SSX6	3.38	3.96	2.74
SNTG1	3.38	3.83	3.03
LOC257152	3.38	3.80	2.96
AGR2	3.38	4.00	2.74
TSACC	3.38	3.83	3.04
NTSR2	3.38	3.91	3.03
LOC728114	3.38	4.01	2.82
MIR605	3.38	4.29	2.80
CASC1	3.38	3.85	3.04
OTTHUMG00000140394	3.38	4.31	2.80
LINC00616	3.38	4.35	2.82
FLG2	3.38	4.06	2.96
SGCZ	3.38	3.88	3.03
NREP-AS1	3.38	3.75	2.93
GUCY2C	3.38	3.60	2.92
FBP2	3.38	3.73	2.95
SLC25A51P1	3.38	3.77	3.00
SLC9A9-AS2	3.38	3.68	2.95
OTTHUMG00000015884	3.38	3.73	2.84
ZNF449	3.38	4.03	2.83

CDCA2	3.38	3.99	2.87
C15orf32	3.38	4.14	3.20
SNORD32B	3.38	4.02	2.75
DUSP21	3.38	3.61	3.11
MIR29B2	3.38	4.50	2.47
OTTHUMG00000165530	3.38	3.84	2.88
OTTHUMG00000067215	3.37	4.01	3.06
AOAH-IT1	3.37	4.02	2.65
OTTHUMG00000010108	3.37	3.75	2.88
NBPF4	3.37	4.12	3.02
ALDH1L1-AS2	3.37	3.74	3.02
IL1B	3.37	3.74	3.09
GEMIN8P4	3.37	3.81	2.94
OTTHUMG00000034550	3.37	4.03	3.02
C9orf40	3.37	3.68	2.97
OTTHUMG00000161193	3.37	3.85	3.04
IL15	3.37	3.94	3.02
CPA3	3.37	4.11	2.63
LINC00365	3.37	3.68	2.84
OTTHUMG00000155428	3.37	3.82	2.98
DPYSL5	3.37	3.56	3.11
LOC646498	3.37	3.84	2.76
HFM1	3.37	3.77	3.05
OTTHUMG00000171586	3.37	3.91	3.07
OR51A2	3.37	4.16	2.71
OTTHUMG00000168815	3.37	3.86	2.61
UBE2DNL	3.37	3.71	3.05
PAH	3.37	3.65	3.00
OTTHUMG00000165258	3.37	3.69	3.10
MYOD1	3.37	3.85	3.01
TXK	3.37	3.98	3.06
ETV5-AS1	3.37	3.84	2.73
VWC2L	3.37	3.68	3.02
LOC100506226	3.37	3.61	3.05
C12orf63	3.37	3.75	3.10
TDGF1	3.37	4.44	2.80
OR10J5	3.37	3.83	3.02
OTTHUMG00000066362	3.37	3.65	3.02
KRTAP4-3	3.37	3.77	2.92
OTTHUMG00000162208	3.37	3.85	3.03
OR4S2	3.37	4.14	2.49
ADAM7	3.37	3.60	3.15
ENAM	3.37	3.90	3.02
NRG3-AS1	3.37	3.88	3.03
CDK1	3.37	3.92	2.78
NT5M	3.37	3.74	3.11
OTTHUMG00000170678	3.37	4.11	2.83
LOC100506891	3.37	3.81	2.88
LOC100130156	3.36	3.89	2.84

OTTHUMG00000154962	3.36	3.77	2.77
AKR1B10	3.36	4.01	2.55
TNP2	3.36	4.04	2.98
ANP32D	3.36	4.09	2.90
OTTHUMG00000166467	3.36	3.54	3.03
LOC642366	3.36	3.71	2.97
LIN28B	3.36	3.84	2.80
ZNF80	3.36	3.78	3.07
HIST1H2AK	3.36	3.86	2.01
DKK2	3.36	3.75	2.98
CCL7	3.36	4.11	2.61
OR14K1	3.36	3.66	2.74
TMED10P1	3.36	4.01	2.85
TRPV6	3.36	3.75	3.04
BPY2	3.36	3.79	3.10
KRTAP10-8	3.36	3.79	2.98
OTTHUMG00000037985	3.36	3.99	2.87
MACROD2	3.36	3.54	3.16
OTTHUMG00000163215	3.36	3.81	2.93
SEC24B-AS1	3.36	3.71	3.02
OTTHUMG00000183865	3.36	3.61	2.99
FLJ39080	3.36	3.66	2.95
LOC100132735	3.36	3.99	2.84
IL17A	3.36	3.60	3.05
MIR9-3	3.36	3.99	2.82
OTTHUMG00000015885	3.36	3.68	3.10
OTTHUMG00000017241	3.36	3.73	3.02
OTTHUMG00000160529	3.36	3.92	2.67
GDF5	3.36	4.05	2.63
A4GNT	3.36	3.88	3.01
OTTHUMG00000032676	3.36	4.13	2.92
INSL6	3.36	4.01	3.05
RHOH	3.36	3.73	3.03
LOC440416	3.36	3.59	3.15
OTTHUMG00000155426	3.36	4.03	2.90
PCDP1	3.36	3.83	3.10
OTTHUMG00000161600	3.36	3.85	2.97
ATCAY	3.36	3.69	2.98
SPANXD	3.36	4.16	2.52
OTTHUMG00000157252	3.36	3.96	2.97
AGTR2	3.36	3.63	3.01
LOC494127	3.36	4.05	2.79
LOC253044	3.36	3.78	3.13
MIR1298	3.36	4.57	2.74
OTTHUMG00000162955	3.36	3.96	2.97
OR51B6	3.36	3.94	2.80
OTTHUMG00000151461	3.36	3.79	2.95
RNA5SP228	3.36	4.00	2.37
LPA	3.36	3.86	2.82

OTTHUMG00000166145	3.36	3.73	3.14
PANK1	3.36	3.74	2.86
OR52N4	3.36	3.83	3.00
LOC151121	3.36	4.24	2.84
PKIB	3.36	3.64	3.02
OTTHUMG00000168478	3.35	3.88	3.03
MIR103A1	3.35	3.98	2.56
OTTHUMG00000168449	3.35	3.90	2.89
CTLA4	3.35	3.64	3.15
OTTHUMG00000002525	3.35	4.44	2.97
OTTHUMG00000170887	3.35	3.61	2.92
LINC00918	3.35	4.26	2.50
LOC100130256	3.35	3.86	2.90
PRHOXNB	3.35	3.61	2.99
FAM3B	3.35	3.72	3.00
FAM150A	3.35	3.98	2.99
OTTHUMG00000037350	3.35	4.05	3.11
OTTHUMG00000161390	3.35	3.83	2.75
MIR892A	3.35	4.90	2.21
WNT2	3.35	3.57	2.93
TMPRSS11E	3.35	3.56	3.01
OTTHUMG00000078275	3.35	3.74	2.96
TSRM	3.35	3.90	3.13
TAT	3.35	3.87	2.91
ST8SIA3	3.35	3.78	2.82
ASPA	3.35	4.56	2.64
OTTHUMG00000165131	3.35	3.67	2.54
SNORA70C	3.35	4.08	2.71
OTTHUMG00000162990	3.35	3.81	2.85
LINC00837	3.35	3.89	2.72
RBFADN	3.35	3.84	3.08
BPIFB6	3.35	3.73	2.91
CCDC67	3.35	3.67	2.77
OR4P4	3.35	4.15	2.75
OR5B2	3.35	4.31	2.64
OTTHUMG00000160041	3.35	3.79	2.96
SRGAP3-AS1	3.35	3.71	2.88
MIR200B	3.35	3.79	3.07
C16orf97	3.35	3.97	2.82
OTTHUMG00000156168	3.35	3.74	3.13
CDH8	3.35	3.95	2.90
OTTHUMG00000169350	3.35	4.05	3.02
PIWIL1	3.35	3.58	3.12
C5orf52	3.35	3.88	3.00
KRT222	3.35	3.75	2.94
ZNF222	3.35	4.00	2.76
KRT34	3.35	4.37	2.95
KRTAP7-1	3.35	3.83	2.80
LCE1F	3.35	4.70	2.29

SLC22A3	3.35	3.64	2.74
SNORD114-22	3.35	4.66	2.50
MMP8	3.35	3.80	3.05
CLRN1-AS1	3.35	3.93	3.05
OTTHUMG00000157254	3.35	4.19	2.80
C9	3.35	4.14	2.90
C12orf61	3.35	3.88	2.95
LINC00366	3.35	3.80	3.13
SEPT7P9	3.35	3.96	2.83
OTTHUMG00000161457	3.35	4.06	2.98
SLC9A4	3.35	3.59	2.88
CR2	3.35	3.65	3.08
OTTHUMG00000019126	3.35	3.79	3.13
LOC100131910	3.35	3.68	2.96
CHODL	3.35	3.67	3.00
ZNF552	3.34	3.96	2.66
SAMD15	3.34	3.91	2.83
ID2B	3.34	3.82	2.77
OTTHUMG00000163796	3.34	3.94	2.90
OTTHUMG00000015698	3.34	4.25	2.95
SLC9A2	3.34	3.52	3.14
OTTHUMG00000167207	3.34	3.76	2.88
HIST1H2APS1	3.34	3.76	2.99
CCDC26	3.34	3.88	2.94
LRRTM1	3.34	3.95	2.94
CYP4A22	3.34	3.75	3.15
CD80	3.34	3.55	3.07
OTOGL	3.34	3.65	3.08
FLJ31104	3.34	3.78	2.96
OTTHUMG00000168500	3.34	3.73	2.88
CCDC105	3.34	3.75	2.88
SSTR3	3.34	3.95	2.65
ZNF273	3.34	3.83	2.85
ETV7	3.34	3.89	2.90
OTTHUMG00000163295	3.34	3.67	3.07
OTTHUMG00000151104	3.34	3.88	2.65
LOC100507336	3.34	3.66	3.14
LINC00598	3.34	3.56	3.02
CXCL11	3.34	3.84	2.82
C16orf96	3.34	4.09	2.99
RNA5SP212	3.34	3.82	2.55
CLDN20	3.34	3.86	2.92
FAM154B	3.34	3.77	3.00
PBOV1	3.34	3.92	3.05
LOC100335030	3.34	3.85	3.03
LINC00841	3.34	3.70	3.05
LOC100133286	3.34	3.72	2.84
FAM83B	3.34	3.82	2.72
CXorf57	3.34	3.64	3.06

LINC00881	3.34	3.85	2.93
ZNF536	3.33	3.67	3.13
LOC100133461	3.33	4.23	2.97
LINC00934	3.33	3.78	2.95
SERPINC1	3.33	3.61	3.01
LOC100506630	3.33	3.69	3.05
SNORD114-8	3.33	4.71	2.58
LRIT2	3.33	4.09	2.95
ZNF157	3.33	3.97	2.88
PLEKHD1	3.33	3.91	3.03
LOC286189	3.33	3.88	2.94
MAGEB17	3.33	3.81	2.73
NLRP14	3.33	3.80	2.92
LINC00964	3.33	3.94	2.86
SERPINI2	3.33	4.65	2.62
OTTHUMG00000008401	3.33	3.73	2.99
TRIM9	3.33	3.76	2.62
ZNF430	3.33	4.18	2.47
SLC22A16	3.33	3.81	3.08
MIR3662	3.33	3.81	2.93
OTTHUMG00000078274	3.33	3.77	3.02
HIGD2B	3.33	3.70	2.91
TTTY9A	3.33	3.78	3.07
PDYN	3.33	3.83	2.77
LINC00643	3.33	4.13	2.84
HORMAD1	3.33	3.63	3.01
LOC100507534	3.33	4.09	2.45
BPESC1	3.33	3.59	3.10
OTTHUMG00000163335	3.33	3.85	2.97
LINC00526	3.33	4.01	2.77
FAM184A	3.33	3.90	3.00
CCDC158	3.33	3.78	2.87
RNF150	3.33	3.89	3.08
C1orf74	3.33	3.84	2.94
SCN3A	3.33	3.87	2.95
LINC00670	3.33	3.76	2.81
FMR1NB	3.33	3.65	3.10
OTTHUMG00000153616	3.33	4.06	2.90
TRAJ47	3.33	4.23	2.77
CSN2	3.33	3.62	2.93
OTTHUMG00000041465	3.33	4.13	2.74
GPR19	3.33	3.88	3.03
SLC5A7	3.33	3.98	2.80
GK-AS1	3.33	4.16	2.75
OTTHUMG00000171211	3.33	3.63	2.95
FUT8-AS1	3.33	3.96	3.01
FBXL22	3.33	3.70	3.13
GATA4	3.33	3.92	3.00
RNA5SP79	3.33	3.80	2.74

IZUMO1	3.33	3.84	2.95
OTTHUMG00000015230	3.33	3.56	3.00
EN2	3.33	3.63	2.75
AQP4	3.33	3.76	2.80
OTTHUMG00000165451	3.33	3.93	2.94
TRPC5	3.33	3.61	2.83
FTLP10	3.32	3.81	2.94
OTTHUMG00000163701	3.32	4.12	2.43
ZNF542	3.32	3.73	2.74
IRS4	3.32	3.47	3.03
OTTHUMG00000162103	3.32	4.61	2.66
OTTHUMG00000164290	3.32	3.91	3.10
OTTHUMG00000018931	3.32	3.86	2.97
LINC00400	3.32	3.74	3.04
OTTHUMG00000154373	3.32	3.65	2.56
RFPL3S	3.32	3.85	2.67
OTTHUMG00000034834	3.32	3.72	3.02
DKFZp566F0947	3.32	3.69	2.83
ATOH7	3.32	3.61	2.95
LOC401410	3.32	3.79	2.78
SMEK3P	3.32	3.73	2.99
LOC100288310	3.32	4.24	2.88
ANO4	3.32	4.21	2.44
OR52N5	3.32	4.26	2.49
SLC16A12	3.32	3.88	2.92
ATAD3C	3.32	3.94	2.84
LINC00533	3.32	4.04	2.52
PMFBP1	3.32	3.80	3.02
LOC100506837	3.32	3.79	2.99
OTTHUMG00000152509	3.32	3.62	3.02
LOC100505540	3.32	4.04	2.63
TRPM8	3.32	3.76	2.97
LINC00689	3.32	3.78	2.74
KRTAP27-1	3.32	3.82	2.67
WDFY2-AS1	3.32	3.82	2.42
SPAM1	3.32	3.61	2.85
TRPC7-AS2	3.32	3.57	3.07
LGALS16	3.32	3.68	3.10
PMAIP1	3.32	3.87	2.75
ZNF567	3.32	4.14	2.54
OTTHUMG00000167518	3.32	3.84	3.11
RBP3	3.32	3.63	2.93
TNFRSF17	3.32	3.95	2.80
GSC2	3.32	3.98	3.05
HOPX	3.32	3.80	2.90
MIR200A	3.32	3.78	2.95
IGSF11-AS1	3.32	3.82	2.65
OTTHUMG00000015229	3.32	3.63	2.92
SCARNA14	3.32	3.84	2.75

CHODL-AS1	3.32	3.92	2.73
OTTHUMG00000150586	3.32	3.86	2.88
KIAA1210	3.32	3.90	2.94
MIR128-2	3.32	3.65	3.04
OTTHUMG00000019960	3.32	3.87	2.88
CPA5	3.32	3.69	2.99
CADM2	3.32	3.85	2.92
LOC100507254	3.32	3.68	2.99
DEPDC1	3.32	3.83	2.65
LOC93432	3.32	3.77	2.92
AKR1C6P	3.31	3.60	3.06
MIR299	3.31	4.14	2.58
OTTHUMG00000160549	3.31	3.72	2.93
OTTHUMG00000164872	3.31	3.62	3.04
DNAJB8-AS1	3.31	3.98	2.98
MAGEB1	3.31	3.49	3.21
OTTHUMG00000168140	3.31	3.71	3.00
NELL2	3.31	3.53	3.10
LINC00857	3.31	3.72	2.94
FAM19A4	3.31	3.81	2.87
PNPLA3	3.31	3.69	2.98
OTTHUMG00000172302	3.31	3.63	2.98
CHGB	3.31	3.65	2.82
OR8D1	3.31	3.55	3.06
OTTHUMG00000152562	3.31	4.17	2.37
RAB3C	3.31	3.92	2.67
FRMPD4	3.31	3.65	2.87
PRG1	3.31	3.81	2.75
LGALS17A	3.31	3.79	2.74
LIN7A	3.31	3.65	3.13
MIR532	3.31	3.84	3.04
OTTHUMG00000159500	3.31	3.79	2.79
HLA-AS1	3.31	3.59	2.76
OTTHUMG00000022481	3.31	4.47	2.29
OTTHUMG00000162517	3.31	3.68	2.97
MGARP	3.31	3.68	2.82
OTTHUMG00000011637	3.31	3.97	2.86
TMED11P	3.31	3.58	2.98
CLTC-IT1	3.31	3.78	2.84
OTTHUMG00000183862	3.31	3.85	3.03
PI15	3.31	5.29	2.56
FGA	3.31	3.73	2.93
MIR3139	3.30	4.27	2.69
OTTHUMG00000150449	3.30	3.96	2.91
OTTHUMG00000014261	3.30	4.12	2.75
OTTHUMG00000170569	3.30	4.39	2.77
OTTHUMG00000163650	3.30	3.61	3.08
OTTHUMG00000031837	3.30	3.59	2.65
OTTHUMG00000172376	3.30	3.68	2.99

TRBV24-1	3.30	3.81	2.71
FLJ10489	3.30	3.74	2.75
OTTHUMG00000170121	3.30	3.84	3.03
BEND7	3.30	3.92	2.91
TACR3	3.30	3.66	2.81
MIR627	3.30	4.38	2.51
OTTHUMG00000170556	3.30	4.42	2.57
ERP27	3.30	3.75	2.78
NANOGNB	3.30	3.72	2.92
IL12A	3.30	3.60	3.14
LIX1	3.30	3.74	3.03
MIR2114	3.30	4.12	2.49
OTTHUMG00000035577	3.30	3.97	2.83
CDY1	3.30	3.70	2.85
OTTHUMG00000165809	3.30	3.72	2.87
LOC100505878	3.30	3.89	2.98
USP6	3.30	3.88	2.83
LINC00639	3.30	3.61	2.75
HLA-DQB1	3.30	3.98	2.86
SLC7A11	3.30	3.80	2.95
OTTHUMG00000178553	3.30	3.93	2.79
MIR1827	3.30	3.75	2.94
KHDRBS2	3.30	3.80	2.84
OTTHUMG00000153018	3.30	3.88	2.66
TPRG1-AS2	3.30	4.01	2.81
MIR137HG	3.30	3.65	2.94
RXRG	3.30	3.62	2.87
NOVA1-AS1	3.30	3.57	2.99
LINC00466	3.30	3.61	2.77
CXCL3	3.30	3.79	2.89
ZIM3	3.30	3.99	2.80
OTTHUMG00000179382	3.30	3.73	2.77
EZR-AS1	3.30	3.61	2.74
CYP4Z1	3.30	3.86	2.54
OTTHUMG00000160728	3.30	3.65	2.92
LOC100169752	3.30	3.90	2.73
ELSPBP1	3.30	3.72	2.95
OTTHUMG00000177616	3.30	3.59	2.90
C1orf227	3.30	3.91	2.82
MYH15	3.30	3.64	2.95
TEKT3	3.29	3.56	2.95
OR4E2	3.29	3.85	2.89
CLPS	3.29	3.78	2.78
LOC100507173	3.29	3.92	2.94
RFX6	3.29	3.65	3.02
RNA5SP290	3.29	3.86	2.47
LHX4	3.29	3.55	3.18
LOC100507556	3.29	3.62	2.93
GPR98	3.29	3.73	2.97

MIR4798	3.29	4.02	2.52
LOC100506188	3.29	3.71	2.97
OTTHUMG00000157196	3.29	3.63	3.04
ZNF295-AS1	3.29	3.67	2.83
PGR	3.29	4.15	2.62
OTTHUMG00000152687	3.29	4.04	2.83
KPNA7	3.29	3.87	2.91
LARGE-AS1	3.29	3.89	2.76
OTTHUMG00000160300	3.29	3.80	2.90
OTTHUMG00000009144	3.29	3.61	2.68
PGK2	3.29	4.13	2.74
LINC00388	3.29	4.01	2.58
MIR1258	3.29	4.38	2.96
DYTN	3.29	4.03	2.68
OTTHUMG00000160718	3.29	3.76	2.83
PLCB2-AS1	3.29	3.78	2.98
BCMO1	3.29	3.78	2.62
OTTHUMG00000161289	3.29	3.72	2.68
OTTHUMG00000042457	3.29	3.72	2.94
FAM222A-AS1	3.29	3.66	3.09
RNA5SP164	3.29	4.09	2.65
RNU7-81P	3.29	3.97	2.65
OTTHUMG00000059376	3.29	3.82	2.47
NPVF	3.29	4.11	2.83
RNF128	3.29	4.22	2.78
PPEF2	3.29	3.80	2.94
SPATA24	3.29	3.63	2.79
LOC441233	3.29	3.86	2.66
PRLR	3.29	3.81	2.89
NKX2-3	3.29	3.66	2.73
OTTHUMG00000164903	3.29	3.92	2.45
OR52A5	3.29	3.60	2.91
ANKRD26P3	3.28	3.95	2.65
KCNQ5-AS1	3.28	3.72	2.48
SLC28A1	3.28	3.90	2.73
SERPINB7	3.28	3.96	2.84
OTTHUMG00000041127	3.28	3.49	2.97
CEACAM8	3.28	3.67	2.81
DCAF8L2	3.28	3.68	2.87
KCNV1	3.28	3.67	2.97
HTR7	3.28	4.59	2.75
MIR101-1	3.28	4.04	2.75
TRAJ46	3.28	3.67	2.68
SH3PXD2A-AS1	3.28	3.78	2.91
LINC00222	3.28	3.84	2.72
SNORD113-5	3.28	4.60	2.15
FAM169A	3.28	3.55	2.97
RAD51AP2	3.28	3.94	2.61
LOC284100	3.28	3.92	2.68

HNRNPCL1	3.28	3.88	2.78
NAV2-AS5	3.28	3.73	2.69
OTTHUMG00000086871	3.28	3.78	2.80
OTTHUMG00000168183	3.28	3.93	2.77
CST11	3.28	3.48	2.93
OTTHUMG00000151422	3.28	3.70	2.72
SLC2A2	3.28	3.96	2.80
C1orf158	3.28	4.15	2.27
MIR218-2	3.28	4.17	2.90
NAV2-AS3	3.28	3.73	2.72
LOC100288748	3.28	3.92	2.84
OTTHUMG00000169604	3.28	4.09	2.76
LOC653786	3.28	4.05	2.81
BEND5	3.28	3.64	3.03
GALNT13	3.28	3.55	2.70
LOC441402	3.28	3.87	2.55
SPRR2G	3.28	3.74	3.09
MYF6	3.28	3.74	2.99
MIR3529	3.28	4.74	2.43
ARPP21	3.28	3.62	3.01
SLC17A1	3.28	3.55	3.02
OTTHUMG00000172245	3.28	4.08	2.73
TDRG1	3.28	3.67	2.69
MIR554	3.28	3.47	2.91
BCL11A	3.28	3.86	2.99
LOC641746	3.28	3.99	2.54
OR6M1	3.28	3.88	2.90
OTTHUMG00000169115	3.28	4.18	2.60
OTTHUMG00000159881	3.27	4.00	2.72
OTTHUMG00000163858	3.27	3.68	2.51
OTTHUMG00000152138	3.27	3.94	2.75
GPR128	3.27	3.62	2.94
MACROD2-IT1	3.27	3.62	3.02
TEX26-AS1	3.27	3.64	3.00
SMIM9	3.27	3.54	2.89
TRBV5-1	3.27	3.69	2.59
OTTHUMG00000016477	3.27	3.66	2.98
OTTHUMG00000164472	3.27	4.05	2.65
MIR4487	3.27	4.51	2.85
VHL	3.27	4.03	2.68
GTSE1-AS1	3.27	3.72	2.85
RNY4P1	3.27	4.49	2.05
LANCL3	3.27	3.57	2.93
OTTHUMG00000032784	3.27	3.72	2.90
OTTHUMG00000140353	3.27	3.89	2.64
RASSF10	3.27	3.73	2.91
DCDC1	3.27	3.70	2.44
ZNF441	3.27	3.83	2.36
MYH8	3.27	4.20	2.81

PKHD1L1	3.27	3.69	3.06
LOC100505841	3.27	3.95	2.51
OTTHUMG00000172301	3.27	4.07	2.89
OTTHUMG00000018041	3.27	4.05	2.83
ZNF674-AS1	3.27	3.58	2.62
MIR4275	3.27	4.56	2.73
LOC285422	3.27	4.38	2.65
OR52E6	3.27	4.49	2.58
OTTHUMG00000152366	3.27	3.71	2.85
HOXB-AS5	3.27	3.72	2.93
OTTHUMG00000161603	3.27	4.09	2.79
PCDH15	3.27	3.86	2.99
HMGA1P4	3.27	3.73	2.89
OR4C13	3.27	3.55	2.93
ENPP6	3.27	3.49	2.96
RPUSD1	3.27	3.62	2.41
OTTHUMG00000153330	3.27	4.08	2.78
OTTHUMG00000166379	3.27	3.99	2.82
PAK7	3.27	3.75	2.99
OTTHUMG00000161218	3.27	4.72	2.68
LOC100505799	3.27	4.25	2.59
LOC100287225	3.27	3.85	2.96
LOC286114	3.26	3.73	2.95
MYO3B	3.26	3.78	2.82
OTTHUMG00000015401	3.26	4.10	2.75
TCN1	3.26	3.68	2.79
LOC339593	3.26	3.83	2.90
OTTHUMG00000160103	3.26	3.68	2.96
CCDC169-SOHLH2	3.26	3.68	2.98
SH2D1A	3.26	3.39	3.03
LOC284688	3.26	3.81	2.85
CRABP1	3.26	3.72	2.99
MYBPC1	3.26	3.62	2.91
IFNA22P	3.26	4.67	2.51
OTTHUMG00000151429	3.26	3.72	3.03
MIR764	3.26	3.52	2.87
FAM45A	3.26	3.94	2.47
SOS1-IT1	3.26	3.85	2.82
CD48	3.26	3.78	2.50
LOC101060360	3.26	3.80	2.83
CLYBL-AS2	3.26	3.71	2.92
CCDC79	3.26	3.61	3.01
ADRA1A	3.26	3.65	2.87
C17orf104	3.26	3.48	3.01
KIAA2022	3.26	3.72	2.87
LINC00423	3.26	4.09	2.64
GEMIN2	3.26	3.75	2.96
OTTHUMG00000154540	3.26	3.61	2.96
OTTHUMG00000169910	3.26	3.85	2.76

OTOA	3.26	3.69	2.82
SCARNA1	3.26	4.23	2.41
OTTHUMG00000170702	3.26	3.92	2.31
CACNG7	3.26	3.76	2.78
FAM216B	3.26	3.63	2.89
UPK1A-AS1	3.26	3.52	2.78
DNAJC5B	3.26	3.66	2.87
TMEM202	3.26	3.67	2.78
MIR3678	3.26	4.22	2.64
EPHA7	3.26	3.57	2.71
OTTHUMG00000152851	3.26	3.59	2.97
AMY2B	3.25	3.64	2.62
DLGAP2	3.25	3.83	2.81
MIR641	3.25	3.79	2.92
OTTHUMG00000170090	3.25	3.76	2.83
TCP10L2	3.25	3.79	2.71
CGA	3.25	3.60	2.96
OTTHUMG00000159723	3.25	3.75	2.70
TRPM1	3.25	4.03	2.77
LOC729950	3.25	3.54	2.90
LRRC7	3.25	4.03	2.75
MIR3942	3.25	3.76	2.64
SCRT1	3.25	3.68	2.83
OTTHUMG00000159127	3.25	3.70	2.98
LOC100130275	3.25	3.63	2.88
ALKBH3-AS1	3.25	3.61	3.02
SNX31	3.25	3.42	3.01
OTTHUMG00000060638	3.25	3.98	2.69
RNA5SP348	3.25	4.12	2.51
NUP62CL	3.25	3.55	2.92
SUCNR1	3.25	3.67	2.88
OTTHUMG00000161282	3.25	3.59	2.79
TRIML2	3.25	3.54	2.95
IL5RA	3.25	3.73	3.03
FAM35DP	3.25	4.32	2.50
NRG1-IT1	3.25	3.66	2.63
PABPC4L	3.25	3.70	2.83
C10orf113	3.25	4.17	2.13
EFCAB5	3.25	3.66	2.84
SDC4P	3.25	3.53	2.91
OTTHUMG00000166787	3.25	3.61	2.75
RNA5SP211	3.25	4.19	2.40
OTTHUMG00000160350	3.25	3.52	3.01
VNN3	3.25	3.72	2.85
RPE65	3.25	3.65	2.96
DEFB126	3.25	3.76	2.90
OTTHUMG00000018296	3.25	3.81	2.69
OTTHUMG00000156144	3.25	3.83	2.94
LOC100505518	3.25	3.84	2.87

GYPA	3.25	3.73	2.71
HSPA7	3.25	3.81	2.61
MAP10	3.25	3.51	2.88
MIR599	3.25	4.00	2.89
NR0B1	3.25	3.62	2.72
PLCXD2-AS1	3.25	3.93	2.80
LINC00574	3.25	3.87	2.88
OTTHUMG00000158944	3.25	3.67	2.67
OTTHUMG00000151815	3.25	3.65	2.75
OTTHUMG00000015993	3.25	4.15	2.67
FLJ34503	3.25	3.73	2.73
OR5A1	3.25	4.28	2.32
C10orf111	3.25	3.62	2.98
TDRD5	3.24	3.67	2.63
OTTHUMG00000163153	3.24	4.01	2.62
RPL13AP17	3.24	3.62	2.96
C9orf43	3.24	3.92	2.97
TEX15	3.24	3.73	2.76
ZNF676	3.24	4.03	2.61
SLCO1C1	3.24	3.97	2.48
OTTHUMG00000161177	3.24	3.76	2.81
LOC284788	3.24	3.83	2.87
ODF1	3.24	3.47	2.99
OTTHUMG00000163244	3.24	3.82	2.91
OR2T1	3.24	4.31	2.62
OTTHUMG00000167171	3.24	3.48	2.71
FAM135B	3.24	3.89	2.72
OR2F2	3.24	3.93	2.56
TRAV8-6	3.24	3.97	2.87
OTTHUMG00000160615	3.24	3.67	2.83
LOC389247	3.24	3.66	2.43
RACGAP1	3.24	3.66	2.61
FAM74A2	3.24	3.77	2.83
OTTHUMG00000164325	3.24	3.98	2.43
LINC00645	3.24	3.75	2.82
LOC100134040	3.24	3.72	2.93
CCT8L2	3.24	3.61	3.00
CNR2	3.24	3.65	2.90
OTTHUMG00000017960	3.24	3.83	2.80
LBX1	3.24	3.68	2.63
OTTHUMG00000155617	3.24	3.58	2.85
ATP6V0D2	3.24	4.81	2.49
ANO3	3.24	3.64	2.79
OTTHUMG00000016506	3.24	3.78	2.80
C7orf76	3.24	3.54	2.73
OTTHUMG00000156396	3.24	3.64	2.88
OTTHUMG00000163848	3.24	4.12	2.34
LOC727677	3.23	3.56	2.95
RTKN2	3.23	3.61	2.87

SPDYE3	3.23	3.62	2.50
LEMD1	3.23	3.97	2.77
GCNT4	3.23	4.06	2.83
TRPA1	3.23	3.95	2.53
OTTHUMG00000160881	3.23	3.72	2.78
LINC00277	3.23	3.60	2.87
C18orf42	3.23	3.62	2.84
KDM5C-IT1	3.23	3.65	2.72
PPBP	3.23	5.37	2.01
GUCY2GP	3.23	3.58	2.74
LINC00242	3.23	3.58	2.81
OTTHUMG00000164937	3.23	3.73	2.87
LOC286467	3.23	3.76	2.79
CSMD3	3.23	3.64	2.94
HRASLS	3.23	3.97	2.72
HP11026	3.23	3.82	2.84
OTTHUMG00000032099	3.23	4.22	2.39
PRMT6	3.23	3.66	2.96
MIR1271	3.23	3.55	2.56
MIR4535	3.23	3.82	2.40
LOC286238	3.23	3.91	2.95
PCDHB11	3.23	3.85	2.66
RNA5SP380	3.23	3.85	2.36
OTTHUMG00000056666	3.23	3.71	2.94
PYHIN1	3.23	3.77	2.74
OTTHUMG00000015821	3.23	3.68	2.82
TECTA	3.23	3.76	2.83
LOC100128437	3.23	4.29	2.45
OTTHUMG00000170368	3.23	3.78	2.92
SLC26A3	3.23	3.55	2.77
OTTHUMG00000032717	3.23	3.57	2.86
IQCF1	3.23	3.60	2.73
LOC729224	3.23	3.73	2.86
OTTHUMG00000165748	3.23	3.97	2.90
FGF16	3.23	4.09	2.52
OTTHUMG00000056662	3.23	3.60	2.73
OTTHUMG00000041347	3.23	3.57	2.77
SPG20OS	3.22	3.63	2.57
C22orf31	3.22	3.88	2.90
LOC100129995	3.22	3.65	2.92
OTTHUMG00000163982	3.22	3.71	2.72
NR2F1-AS1	3.22	3.52	2.80
SLC9A9-AS1	3.22	3.79	2.73
EIF2B5-AS1	3.22	3.66	2.61
TBC1D28	3.22	3.78	2.48
OTTHUMG00000155767	3.22	3.97	2.79
OTTHUMG00000033265	3.22	3.65	2.22
RFPL1	3.22	3.68	2.72
OTTHUMG00000150464	3.22	3.53	2.85

OTTHUMG00000152593	3.22	3.76	2.74
OTTHUMG00000160105	3.22	3.77	2.88
PPY	3.22	4.09	2.27
GRB14	3.22	3.66	2.86
CFHR5	3.22	3.68	2.75
OTTHUMG00000172296	3.22	3.83	2.68
FMN2	3.22	3.53	2.87
IGLV7-46	3.22	3.80	2.80
OTTHUMG00000010848	3.22	3.69	2.90
OTTHUMG00000172455	3.22	3.83	2.76
KRTAP4-6	3.22	3.85	2.73
BHLHE23	3.22	3.53	2.70
LOC100506389	3.22	3.53	2.96
RNA5SP398	3.22	3.72	2.66
MEFV	3.22	3.70	2.68
ASB11	3.22	3.55	2.97
OTTHUMG00000179433	3.22	3.60	2.83
ATP2B2-IT2	3.22	3.86	2.82
HOXD13	3.22	3.80	2.84
OTTHUMG00000166002	3.22	3.57	3.02
TRHR	3.21	3.90	2.35
OTTHUMG00000152016	3.21	3.48	2.93
OR2AP1	3.21	3.70	2.72
P2RY4	3.21	3.53	2.91
SLC6A14	3.21	3.45	3.01
OTTHUMG00000165125	3.21	3.72	2.75
HIC1	3.21	3.63	2.86
CYP1A1	3.21	3.66	2.72
OTTHUMG00000164326	3.21	3.66	2.66
TIGD4	3.21	3.66	2.97
CHIAP2	3.21	3.66	2.94
CCDC173	3.21	3.59	2.88
GRID2	3.21	3.84	2.69
ZNF385D	3.21	3.48	2.92
NDUFAF4	3.21	3.85	2.64
OTTHUMG00000158587	3.21	3.94	2.64
MIR4287	3.21	3.97	2.37
OR4A15	3.21	3.58	2.89
REN	3.21	3.78	2.88
OTTHUMG00000022291	3.21	3.58	2.83
LOC100506776	3.21	3.68	2.88
CAPNS2	3.21	3.78	2.83
GRHL1	3.21	3.79	2.80
LINC00307	3.21	3.63	2.96
SLC22A24	3.21	3.49	2.91
AADACL4	3.21	3.59	2.84
ARHGEF38	3.21	3.56	2.97
HSFY2	3.21	3.63	2.67
LOC152742	3.21	3.65	2.71

WASF3-AS1	3.21	3.55	2.61
ANKRD30BL	3.21	3.56	2.75
OR8D2	3.21	3.86	2.41
IQCF4	3.21	4.17	2.72
OTTHUMG00000032757	3.21	3.56	2.76
FAM27D1	3.21	3.52	3.02
LOC100506705	3.21	3.67	2.58
OR1E1	3.21	3.88	2.65
FAM205B	3.21	4.01	2.51
SPACA1	3.20	3.50	2.86
OTTHUMG00000161903	3.20	3.72	2.74
OTTHUMG00000086614	3.20	3.91	2.78
CCL20	3.20	3.62	2.67
KIAA0087	3.20	3.54	2.80
HLA-H	3.20	4.06	2.54
LINC00844	3.20	3.64	2.92
C15orf48	3.20	3.50	3.04
KRT24	3.20	3.65	2.85
FAXC	3.20	3.71	2.69
MIR4470	3.20	4.04	2.62
OTTHUMG00000163990	3.20	3.60	2.83
METTL21EP	3.20	3.69	2.72
SLC22A10	3.20	3.43	2.90
OTTHUMG00000177853	3.20	3.53	2.75
IFNB1	3.20	3.71	2.96
SLC2A9	3.20	3.60	2.84
SMCO2	3.20	3.60	2.86
OTTHUMG00000001278	3.20	3.86	2.85
OTTHUMG00000154818	3.20	4.00	2.47
OTTHUMG00000024091	3.20	3.69	2.44
VSIG1	3.20	3.82	2.74
TFEC	3.20	3.48	2.79
SNORA54	3.20	3.78	2.48
LOC100506546	3.20	3.73	2.85
JARID2-AS1	3.20	3.64	2.61
OTTHUMG00000155300	3.20	3.64	2.68
CLEC6A	3.20	3.60	2.73
MACC1	3.20	3.84	2.36
LINC00443	3.20	3.59	2.79
NSUN7	3.20	3.68	2.75
ZNF675	3.20	3.93	2.23
ABCC6P1	3.20	3.74	2.80
LOC401980	3.20	3.75	2.88
CDRT15L2	3.20	4.22	2.09
CDS1	3.20	3.57	2.90
DGCR10	3.20	3.93	2.45
GC	3.20	3.49	3.03
OR4X1	3.20	3.71	2.69
C10orf71-AS1	3.19	3.80	2.93

OSTCP1	3.19	4.14	2.71
OR6N1	3.19	3.69	2.45
CLRN3	3.19	3.78	2.68
RMST	3.19	3.48	2.75
CYP2C9	3.19	3.69	2.75
OTTHUMG00000148821	3.19	3.48	2.93
OTTHUMG00000159698	3.19	3.74	2.66
C5orf64	3.19	3.89	2.56
IGKV2-24	3.19	4.40	2.04
RNA5SP46	3.19	4.00	2.25
DHRS9	3.19	3.50	2.76
OTTHUMG00000151405	3.19	3.58	2.85
SMIM1	3.19	3.56	2.80
CCL1	3.19	3.59	2.81
OTTHUMG00000172869	3.19	3.84	2.80
IL17F	3.19	3.40	2.98
SNORD115-17	3.19	4.39	2.35
CDH10	3.19	3.66	2.59
SPATA16	3.19	3.68	2.82
RNA5SP273	3.19	4.07	1.98
FAM69B	3.19	3.62	2.73
OTTHUMG00000158757	3.19	3.98	2.77
OTTHUMG00000132776	3.19	3.71	2.74
NEFL	3.19	3.86	2.87
RGSL1	3.19	3.59	2.73
UNQ9370	3.19	3.73	2.80
MTMR8	3.19	3.63	2.74
LINC00343	3.19	3.65	2.78
EMR3	3.19	3.53	2.84
MIR4425	3.19	4.36	2.76
LOC100873065	3.19	3.44	2.91
RNA5SP361	3.19	4.17	2.46
OTTHUMG00000164340	3.19	3.65	2.81
ULBP3	3.19	3.41	2.69
CYCSP52	3.19	3.75	2.54
LOC100507600	3.18	3.86	2.53
ADAM21	3.18	3.38	3.04
OTTHUMG00000162046	3.18	3.63	2.74
FGF6	3.18	3.48	2.84
OTTHUMG00000160471	3.18	3.61	2.68
OTTHUMG00000015516	3.18	3.74	2.59
MMD2	3.18	3.69	2.88
OTTHUMG00000162146	3.18	3.80	2.75
ZNF23	3.18	3.60	2.87
KCNC2	3.18	3.87	2.89
ABCA9-AS1	3.18	3.93	2.33
ACTN2	3.18	3.50	2.64
MIR4729	3.18	3.94	2.17
CWH43	3.18	3.59	2.94

RBM46	3.18	3.39	2.82
TMEM240	3.18	3.52	2.66
CHCHD4P2	3.18	3.74	2.82
OTTHUMG00000161686	3.18	3.89	2.43
TLR8	3.18	3.85	2.34
OTTHUMG00000163758	3.18	4.04	2.63
OTTHUMG00000015002	3.18	3.64	2.83
OTTHUMG00000162107	3.18	3.81	2.70
RIMBP3B	3.18	4.18	2.26
F11	3.18	3.59	2.87
LOC100505827	3.18	4.11	2.56
OTTHUMG00000014226	3.18	3.68	2.57
RNA5SP418	3.18	4.07	2.22
DBH-AS1	3.18	3.80	2.69
LOC643486	3.18	4.35	1.76
CALCR	3.18	3.70	2.55
LOC100506675	3.18	3.42	2.80
OTTHUMG00000158734	3.18	3.71	2.57
C10orf40	3.18	3.65	2.89
OTTHUMG00000161939	3.18	3.83	2.75
MMP27	3.18	3.41	2.89
SNORD70	3.18	3.92	2.29
VN1R5	3.18	3.76	2.81
CHIA	3.18	3.59	2.84
TCHH	3.18	3.78	2.89
LOC100996583	3.18	3.66	2.56
NR1H4	3.17	3.48	2.93
OR4F13P	3.17	3.59	2.69
ACTG2	3.17	3.41	2.76
GPR31	3.17	3.41	2.84
OTTHUMG00000166791	3.17	3.90	2.69
TCAM1P	3.17	3.41	2.68
MIR1203	3.17	4.24	2.51
TRAJ31	3.17	3.96	2.24
OTTHUMG00000059745	3.17	3.54	2.85
C4orf45	3.17	3.63	2.88
OR5P3	3.17	3.62	2.88
MIR130A	3.17	3.98	2.43
FAM181B	3.17	3.90	2.64
PHYHIPL	3.17	3.42	2.93
OTTHUMG00000021757	3.17	3.42	3.01
TRAJ34	3.17	4.07	2.01
ASB5	3.17	3.63	2.80
OTTHUMG00000158714	3.17	3.89	2.74
MIR1912	3.17	3.48	2.94
FAM90A25P	3.17	3.69	2.60
GCSAML	3.17	3.54	2.89
OR6J1	3.17	3.64	2.75
AICDA	3.17	3.72	2.73

OR51D1	3.17	3.78	2.76
LINC00704	3.17	3.58	2.79
OTTHUMG00000014922	3.17	4.11	2.78
CPA2	3.17	3.77	2.61
OTTHUMG00000178131	3.17	3.69	2.75
OTTHUMG00000159058	3.17	3.61	2.70
ANKRD7	3.17	3.48	2.96
OTTHUMG00000160737	3.17	3.66	2.69
LRRC72	3.17	3.48	2.79
LOC401176	3.17	3.51	2.83
OR2W5	3.17	3.81	2.79
DEFB125	3.16	4.07	2.47
NLRC4	3.16	3.47	2.67
HYAL4	3.16	3.79	2.68
RETNLB	3.16	3.47	2.80
MGC32805	3.16	3.49	2.85
RNA5SP305	3.16	4.16	2.51
LINC00922	3.16	4.06	2.63
OR5W2	3.16	3.63	2.79
TRBV4-1	3.16	3.97	2.62
LYST-AS1	3.16	4.00	2.37
KTN1-AS1	3.16	3.59	2.88
LOC100506444	3.16	3.51	2.74
OTTHUMG00000162402	3.16	4.34	2.37
LOC100506102	3.16	3.48	2.80
OTTHUMG00000166055	3.16	3.93	2.59
OTTHUMG00000162675	3.16	3.60	2.48
LIPN	3.16	4.06	2.83
ZNF346-IT1	3.16	3.82	2.66
RMDN2-AS1	3.16	3.56	2.93
FABP9	3.16	3.99	2.74
SNORD19	3.16	3.85	2.65
MIR4255	3.16	4.08	2.37
NLRP11	3.16	3.85	2.74
MIR2682	3.16	3.44	2.93
CCL24	3.16	3.96	2.70
DIRC1	3.16	3.74	2.23
OR4Q3	3.16	3.81	2.76
R3HDML	3.16	3.50	2.75
UBE2E2-AS1	3.16	3.58	2.64
OTTHUMG00000014282	3.16	3.53	2.67
OTTHUMG00000153696	3.16	3.48	2.75
LOC100508631	3.16	3.51	2.56
CDH19	3.16	3.76	2.67
GFRAL	3.16	3.46	2.93
OTTHUMG00000152120	3.16	3.67	2.76
HAO1	3.16	3.52	2.81
GJE1	3.16	3.48	2.67
PRDM7	3.16	3.82	2.42

OTTHUMG00000031681	3.16	3.79	2.86
OTTHUMG00000153466	3.16	3.63	2.83
OTTHUMG00000172383	3.16	3.63	2.76
OTTHUMG00000154203	3.16	3.51	2.96
GNAT3	3.16	3.82	2.69
MCMDC2	3.16	3.60	2.50
ACBD7	3.16	3.60	2.69
OTTHUMG00000168371	3.16	3.85	2.57
OTTHUMG00000162584	3.16	3.78	2.61
MROH2B	3.15	3.46	2.92
OTTHUMG00000161706	3.15	3.60	2.69
OTTHUMG00000154090	3.15	3.96	2.80
TRBV3-1	3.15	3.85	2.83
C17orf74	3.15	3.63	2.71
GRAP	3.15	3.65	2.55
OR8G2	3.15	4.42	2.02
BTG4	3.15	4.09	2.79
LOC100505835	3.15	3.73	2.86
OR10AB1P	3.15	4.18	2.38
DNAH8	3.15	3.74	2.75
SNTN	3.15	3.53	2.80
OTTHUMG00000162201	3.15	3.52	2.71
FAM133B	3.15	3.63	2.78
OTTHUMG00000169926	3.15	3.65	2.31
OTTHUMG00000015708	3.15	3.36	2.71
IGBP1P1	3.15	3.90	2.55
KU-MEL-3	3.15	3.76	2.74
SYTL5	3.15	3.32	2.97
OTTHUMG00000162587	3.15	3.83	2.68
OTTHUMG00000170587	3.15	4.13	2.53
OTTHUMG00000183942	3.15	3.62	2.72
ABCC12	3.15	3.75	2.76
OTTHUMG00000171504	3.15	3.78	2.67
MOS	3.14	3.56	2.70
CLNK	3.14	3.56	2.90
OTTHUMG00000152879	3.14	3.78	2.80
CDX4	3.14	3.41	2.79
JAKMIP1	3.14	3.52	2.85
MIR548C	3.14	4.06	1.88
GCG	3.14	3.60	2.81
OTTHUMG00000015419	3.14	3.55	2.55
ST6GAL2-IT1	3.14	4.43	1.90
GBP7	3.14	3.75	2.67
OTTHUMG00000172111	3.14	3.36	2.87
RNA5SP265	3.14	4.08	2.63
AMPD1	3.14	3.75	2.66
OR13D1	3.14	3.96	2.44
RNU6-51	3.14	4.31	2.30
OTTHUMG00000168323	3.14	3.67	2.88

FER1L6-AS2	3.14	3.55	2.72
RNA5SP325	3.14	4.04	2.13
ENPP5	3.14	3.64	2.61
SLC24A5	3.14	3.48	2.70
AQP4-AS1	3.14	3.73	2.72
LOC339298	3.14	3.54	2.72
TTTY18	3.14	3.65	2.63
BCL2L15	3.14	3.53	2.72
ASB15	3.14	3.64	2.94
LOC399815	3.14	3.97	2.58
CNPY1	3.14	3.75	2.87
CASP5	3.14	4.04	2.68
LOC101060654	3.14	4.21	2.46
SBF1P1	3.14	3.52	2.70
OTTHUMG00000152029	3.13	3.61	2.66
FAM9A	3.13	3.55	2.53
PLCH1	3.13	3.41	2.74
OR4S1	3.13	3.81	2.36
IL26	3.13	3.56	2.77
UTS2B	3.13	3.59	2.89
KCNH5	3.13	3.57	2.78
EYS	3.13	3.55	2.76
POM121L4P	3.13	3.99	2.36
ADAD1	3.13	3.52	2.86
OTTHUMG00000155317	3.13	3.68	2.70
GABRG3	3.13	3.60	2.73
MIR1302-10	3.13	4.11	2.68
SNORD96B	3.13	3.75	2.39
OTTHUMG00000151631	3.13	3.51	2.70
OTTHUMG00000161340	3.13	3.74	2.52
OTTHUMG00000171379	3.13	3.69	2.67
WWC2-AS1	3.13	4.10	2.45
IL8	3.13	3.41	2.58
SOX2-OT	3.13	3.58	2.64
GALNT3	3.13	3.86	2.63
AQP9	3.13	3.49	2.73
ACTL7A	3.13	3.42	2.93
KCNU1	3.13	3.43	2.77
OTTHUMG00000015717	3.13	3.62	2.72
CR1L	3.13	3.51	2.84
GZMK	3.13	4.02	2.66
OTTHUMG00000183907	3.13	3.62	2.74
OR2B2	3.13	3.80	2.69
SLC12A1	3.13	3.58	2.74
MIR4478	3.13	3.66	2.79
GPR37	3.13	3.46	2.67
MC4R	3.13	3.54	2.63
MIR4672	3.13	3.61	2.38
OTTHUMG00000032808	3.13	3.60	2.75

RNA5SP47	3.13	3.86	2.68
BTBD8	3.13	3.81	2.78
PRAMEF14	3.13	3.93	2.54
ST8SIA6-AS1	3.13	3.49	2.77
MUC15	3.13	3.61	2.68
CES5A	3.13	3.62	2.78
ERCC6L	3.13	3.32	2.94
LOC100131796	3.12	3.55	2.59
RNU1-15P	3.12	4.12	2.37
ANXA13	3.12	3.73	2.69
OTTHUMG00000009147	3.12	3.45	2.80
TDRD1	3.12	3.77	2.69
C17orf105	3.12	3.67	2.64
OR10G2	3.12	3.75	2.50
SCN1A	3.12	3.78	2.51
FANCB	3.12	3.45	2.60
HOXB5	3.12	3.37	2.64
CTXN3	3.12	3.70	2.74
GJB7	3.12	3.54	2.79
RNA5SP76	3.12	3.56	2.80
RNA5SP221	3.12	3.71	2.18
OTTHUMG00000155853	3.12	3.39	2.82
KCNH1-IT1	3.12	4.42	2.22
OTTHUMG00000161880	3.12	3.86	2.48
OTTHUMG00000001777	3.12	3.81	2.18
TRAV13-2	3.12	3.70	2.70
RN7SKP3	3.12	4.30	2.63
LOC100509686	3.12	3.73	2.67
OTTHUMG00000171305	3.12	3.64	2.76
OTTHUMG00000161896	3.12	3.90	2.75
TEKT1	3.12	3.44	2.87
OTTHUMG00000156464	3.12	3.43	2.64
BHLHA9	3.12	3.90	2.58
SLC6A15	3.12	3.83	2.65
OTTHUMG00000160893	3.12	3.38	2.75
MIR3126	3.12	3.89	2.44
OTTHUMG00000015959	3.12	3.65	2.89
LINC00160	3.12	3.63	2.84
MIR153-2	3.12	4.06	2.37
OTTHUMG00000017651	3.12	3.73	2.55
FLJ46066	3.12	3.50	2.47
HIST1H2AH	3.12	3.38	2.67
YBX2	3.12	3.54	2.54
C21orf91-OT1	3.12	3.50	2.56
OTTHUMG00000168702	3.12	3.71	2.58
CCDC62	3.12	3.48	2.64
OTTHUMG00000152116	3.12	4.32	2.63
FABP1	3.11	3.43	2.87
ZNF730	3.11	3.41	2.79

PROSER2-AS1	3.11	3.86	2.62
IGKV1D-8	3.11	4.05	2.36
OTTHUMG00000163759	3.11	3.41	2.64
OTTHUMG00000164050	3.11	4.04	2.48
C12orf77	3.11	3.50	2.71
OTTHUMG00000035890	3.11	3.79	2.48
MIR628	3.11	3.70	2.62
KCTD8	3.11	3.51	2.76
INSL5	3.11	3.71	2.61
MTRNR2L4	3.11	4.14	2.59
OTTHUMG00000165032	3.11	3.39	2.86
FAM108A4P	3.11	3.61	2.61
LOC729121	3.11	3.53	2.84
PLEKHG7	3.11	3.49	2.63
ADH7	3.11	3.83	2.57
TRBV4-2	3.11	4.03	2.72
OTTHUMG00000015381	3.11	4.02	2.60
GRIK1-AS1	3.11	3.34	2.73
CLEC4GP1	3.11	3.87	2.04
OTTHUMG00000017600	3.11	3.44	2.86
TMEM244	3.11	3.23	2.99
OTTHUMG00000159200	3.11	3.58	2.52
TBC1D3P2	3.11	3.59	2.81
FLJ46320	3.11	3.47	2.81
FEZF1-AS1	3.11	3.52	2.85
MIR7-3	3.11	3.54	2.70
OTTHUMG00000170246	3.11	3.40	2.49
LOC100505938	3.11	3.56	2.62
ROPN1B	3.11	3.81	2.41
RNA5SP319	3.11	3.85	2.54
OTTHUMG00000009931	3.11	3.48	2.75
MS4A1	3.11	3.60	2.51
OTTHUMG00000172571	3.11	3.50	2.56
CHRNA6	3.11	3.55	2.50
RNU7-51P	3.11	3.92	2.41
GABRR1	3.11	3.60	2.62
OTTHUMG00000162826	3.11	3.52	2.78
DDI1	3.11	3.38	2.88
OTTHUMG00000169505	3.11	3.52	2.82
STXBP5-AS1	3.11	3.48	2.73
BCORP1	3.10	3.56	2.70
KRTAP13-3	3.10	3.84	2.51
OTTHUMG00000168427	3.10	3.70	2.79
OTTHUMG00000171721	3.10	3.35	2.83
DMP1	3.10	4.35	2.65
LOC100287010	3.10	3.51	2.72
LOC100034248	3.10	4.13	2.39
RNA5SP503	3.10	3.58	2.46
LOC100129297	3.10	3.52	2.81

OTTHUMG00000020846	3.10	3.71	2.76
FAM72B	3.10	4.19	2.17
RAB27B	3.10	3.36	2.82
GPC5-AS1	3.10	3.59	2.82
OTTHUMG00000162192	3.10	4.18	2.69
MIR548A1	3.10	4.23	2.32
OTTHUMG00000159002	3.10	3.46	2.77
PASD1	3.10	3.48	2.90
OTTHUMG00000153854	3.10	3.74	2.80
LINC00858	3.10	3.26	2.74
OTTHUMG00000162363	3.10	3.97	2.38
MIR16-1	3.10	3.54	2.64
TSSK2	3.10	3.63	2.78
ZP2	3.10	3.58	2.60
DIRAS3	3.10	3.52	2.61
OTTHUMG00000017086	3.10	4.05	2.52
ENTHD1	3.10	3.42	2.54
IL21-AS1	3.10	3.35	2.91
OTTHUMG00000132063	3.10	3.44	2.67
OTTHUMG00000163632	3.10	3.54	2.87
ZNF560	3.10	3.67	2.58
ADAM29	3.10	3.48	2.72
LINC00880	3.10	3.55	2.61
LOC100506394	3.10	3.65	2.38
OTTHUMG00000150625	3.10	3.90	2.63
OTTHUMG00000154836	3.10	3.55	2.59
MIR4694	3.10	4.03	2.44
SLC7A14	3.10	3.55	2.72
OTTHUMG00000163835	3.09	3.87	2.54
RAG2	3.09	3.50	2.72
LY75	3.09	3.74	2.53
CLEC2A	3.09	3.84	2.59
ANGPTL3	3.09	3.62	2.75
KIF5C	3.09	3.53	2.87
MIR148A	3.09	4.01	2.12
OTTHUMG00000165812	3.09	3.97	2.18
OTTHUMG00000171039	3.09	3.67	2.77
OTTHUMG00000178859	3.09	3.46	2.70
DPP10	3.09	3.44	2.69
OTTHUMG00000169950	3.09	3.69	2.59
OTTHUMG00000169386	3.09	3.84	2.54
OTTHUMG00000170076	3.09	3.71	2.69
MIR335	3.09	3.75	2.68
OTTHUMG00000164289	3.09	3.77	2.55
LINC00635	3.09	3.75	2.71
LOC100507363	3.09	3.37	2.63
KRT32	3.09	3.42	2.76
CYP7A1	3.09	3.58	2.62
ANKRD30A	3.09	3.47	2.79

OTTHUMG00000161302	3.09	3.50	2.80
CLGN	3.09	3.65	2.68
C10orf126	3.09	3.86	2.65
TAS2R46	3.09	3.90	2.31
MIR320C2	3.09	3.58	2.68
OTTHUMG00000158709	3.09	3.58	2.91
SLCO1A2	3.09	3.47	2.73
OTTHUMG00000154158	3.09	3.79	2.39
DGKB	3.09	3.37	2.99
OTTHUMG00000162954	3.09	3.92	2.20
OTTHUMG00000014332	3.09	3.75	2.39
SPINK7	3.09	3.52	2.57
LOC283435	3.09	4.20	2.56
OTTHUMG00000171418	3.09	4.39	2.36
NDUFAF4P1	3.09	3.54	2.38
SNORD114-25	3.09	5.46	1.75
MIR454	3.09	3.66	2.75
OTTHUMG00000164063	3.09	3.46	2.69
LDHAL6B	3.09	4.00	2.36
ZNF229	3.09	3.56	2.45
MIR4796	3.09	3.67	2.38
TMCO5A	3.09	3.52	2.74
LOC100506274	3.08	3.52	2.57
LINC00628	3.08	3.39	2.74
OTTHUMG00000168468	3.08	3.73	2.61
GABRG2	3.08	3.53	2.78
OTTHUMG00000161900	3.08	3.42	2.72
POU1F1	3.08	3.46	2.71
RNU5E-7P	3.08	3.65	2.59
LOC340074	3.08	3.68	2.50
KLB	3.08	3.26	2.87
OR9A2	3.08	3.39	2.54
SLC13A1	3.08	3.38	2.79
GPC5-AS2	3.08	3.93	2.75
PTF1A	3.08	3.43	2.63
LOC200726	3.08	3.61	2.59
ARHGEF3-AS1	3.08	3.75	2.33
TRAJ30	3.08	3.86	2.39
LINC00393	3.08	3.67	2.87
OTTHUMG00000086812	3.08	4.08	2.53
FAM110C	3.08	4.07	2.52
OR7G3	3.08	3.80	2.44
OTTHUMG00000163985	3.08	3.62	2.71
FNDC1-IT1	3.08	4.03	2.26
C6orf141	3.08	3.46	2.67
IGKV4-1	3.08	3.68	2.63
EFCAB6-AS1	3.08	3.48	2.71
GKN1	3.08	3.57	2.51
LOC100507103	3.08	3.43	2.64

SLC26A7	3.08	3.39	2.58
FZD3	3.08	3.51	2.66
OTTHUMG00000022476	3.08	3.35	2.83
C10orf114	3.08	3.50	2.58
SPANXA2-OT1	3.08	3.47	2.56
OTTHUMG00000173394	3.08	3.27	2.87
RNA5SP205	3.08	4.55	2.40
CYP4F2	3.08	3.67	2.53
GRM7	3.08	3.40	2.79
OTTHUMG00000172319	3.08	3.46	2.79
OTTHUMG00000156644	3.08	3.46	2.59
RASSF9	3.08	3.67	2.52
SLC4A10	3.08	3.45	2.68
TM4SF4	3.07	3.32	2.78
SULT2A1	3.07	3.62	2.66
KCNK9	3.07	3.37	2.75
OTTHUMG00000166216	3.07	3.40	2.56
MIR520E	3.07	4.09	2.48
TRAJ9	3.07	3.82	2.28
KRTAP4-11	3.07	3.78	2.30
OTTHUMG00000169974	3.07	4.43	2.53
MIR455	3.07	3.36	2.80
OR2B6	3.07	3.47	2.41
OTTHUMG00000020254	3.07	3.57	2.57
SLC27A2	3.07	3.62	2.66
CXorf21	3.07	4.08	2.72
WFDC11	3.07	3.29	2.59
CHL1-AS1	3.07	3.31	2.59
CCDC68	3.07	4.26	2.44
FBXW12	3.07	3.50	2.53
MIR1224	3.07	4.05	1.87
RNA5SP159	3.07	3.87	2.18
APOL1	3.07	3.63	2.64
TDH	3.07	3.49	2.68
OTTHUMG00000151746	3.07	3.34	2.78
OTTHUMG00000169704	3.07	4.00	2.70
DAOA-AS1	3.07	3.27	2.81
NEUROD1	3.07	3.43	2.83
OR4K17	3.07	3.42	2.64
OTTHUMG00000155985	3.07	3.94	2.54
SERPINB5	3.07	3.61	2.70
LINC00692	3.07	3.42	2.80
MRGPRX3	3.07	4.16	2.66
C8orf56	3.07	3.55	2.45
KRTAP19-1	3.07	3.50	2.70
MIR4788	3.07	3.46	2.90
ADAM5	3.07	3.46	2.55
MIR613	3.07	3.44	2.55
OTTHUMG00000161633	3.07	3.57	2.45

DLEU7-AS1	3.07	3.49	2.81
MAGEB3	3.07	3.56	2.63
ORM2	3.07	3.91	2.54
GBP5	3.07	3.48	2.48
ZNF705B	3.07	3.70	2.64
UBE2S	3.07	4.05	2.22
LOC730159	3.06	3.54	2.55
OTTHUMG00000162015	3.06	3.74	2.67
OTTHUMG00000162327	3.06	3.48	2.67
OTTHUMG00000167727	3.06	3.62	2.60
FDCSP	3.06	3.65	2.38
CAGE1	3.06	3.61	2.73
RNU3P3	3.06	3.72	2.40
FLJ33534	3.06	3.52	2.62
TCL1B	3.06	3.69	2.23
NKX2-1-AS1	3.06	3.28	2.82
OVCH2	3.06	3.31	2.81
OTTHUMG00000161058	3.06	3.43	2.43
RIT2	3.06	3.78	2.81
OTTHUMG00000041346	3.06	3.71	2.64
OTTHUMG00000161651	3.06	3.53	2.59
OR6C76	3.06	3.93	2.56
PIP	3.06	3.27	2.76
OTTHUMG00000168653	3.06	3.67	2.33
TSLP	3.06	3.51	2.58
BRD7P3	3.06	3.98	2.44
OTTHUMG00000017017	3.06	3.47	2.54
SORCS3	3.06	3.74	2.79
SNORA31	3.06	3.93	2.19
APOC1P1	3.06	3.72	2.44
HAVCR1	3.06	3.72	2.67
CD96	3.06	3.57	2.83
MRPL42P5	3.06	3.67	2.48
PCYT1B-AS1	3.06	3.65	2.58
LOC100505776	3.06	3.49	2.87
LOC650226	3.06	3.50	2.52
TEX13A	3.06	3.55	2.72
VSTM2A	3.06	3.46	2.76
MCF2	3.06	3.72	2.60
LINC00603	3.06	3.81	2.62
TAS2R39	3.06	3.58	2.43
OTTHUMG00000009987	3.06	3.48	2.46
OTTHUMG00000003704	3.06	3.46	2.69
ULBP1	3.05	4.14	2.13
LOC646034	3.05	3.75	2.70
OTTHUMG00000150005	3.05	3.65	2.65
OTTHUMG00000161149	3.05	3.60	2.64
TSPAN8	3.05	3.37	2.79
IGLV1-50	3.05	3.59	2.32

SNORD114-28	3.05	4.99	2.08
CLCA3P	3.05	3.50	2.73
COL4A5	3.05	3.37	2.74
PRRG4	3.05	3.49	2.62
FAM71D	3.05	3.37	2.68
NAALAD2	3.05	3.57	2.51
LOC100129175	3.05	4.14	2.64
RNA5SP307	3.05	3.81	2.70
SIM1	3.05	3.21	2.69
MIR26A2	3.05	3.51	2.68
HTN3	3.05	3.74	2.47
LOC100505534	3.05	3.44	2.61
OTTHUMG00000015005	3.05	3.80	2.72
TEX36	3.05	3.66	2.60
KRTAP2-4	3.05	4.09	2.63
OTTHUMG00000040015	3.05	3.40	2.63
LOC100505834	3.05	3.82	2.69
OTTHUMG00000015227	3.05	3.27	2.71
OTTHUMG00000015405	3.05	3.64	2.71
LIPF	3.05	3.78	2.56
OTTHUMG00000157098	3.05	3.54	2.56
OTTHUMG00000163672	3.05	3.59	2.35
MYH2	3.05	3.52	2.75
TRAV26-2	3.05	4.13	2.10
OTTHUMG00000167286	3.05	3.46	2.61
DAOA	3.05	3.42	2.68
KRT25	3.05	3.67	2.76
OTTHUMG00000170374	3.05	3.41	2.56
MYO1H	3.04	3.37	2.77
LINC00272	3.04	4.03	2.55
CNTN3	3.04	3.67	2.69
ZCCHC13	3.04	3.40	2.70
CCNB3	3.04	3.49	2.40
KCNN2	3.04	3.64	2.68
C14orf39	3.04	3.69	2.71
IGLV2-23	3.04	3.81	2.24
PYDC2	3.04	3.46	2.74
APOH	3.04	3.67	2.54
OTTHUMG00000163972	3.04	3.49	2.64
TDO2	3.04	3.38	2.42
C6orf222	3.04	3.43	2.57
MIR4301	3.04	4.03	2.31
GABRA2	3.04	3.57	2.62
OTTHUMG00000161563	3.04	3.42	2.74
EFNA4	3.04	3.40	2.51
CNTN5	3.04	3.37	2.63
MIR4420	3.04	4.00	2.37
OTTHUMG00000156041	3.04	3.37	2.66
MIR3609	3.04	3.73	2.25

FAM197Y1	3.04	3.88	2.32
TMPRSS11BNL	3.04	3.72	2.65
LOC440934	3.04	3.57	2.68
OTTHUMG00000175629	3.04	3.56	2.55
MIR3663	3.04	3.72	2.67
CNOT10-AS1	3.04	3.40	2.76
MIR4659A	3.04	4.16	2.09
RPS4Y2	3.04	3.91	2.33
MIR505	3.04	3.73	2.38
OR8K3	3.04	3.78	2.63
DNAJB13	3.04	3.41	2.59
PRL	3.04	3.40	2.63
RFPL4B	3.04	3.65	2.47
OTTHUMG00000162333	3.04	3.53	2.71
TPTE2P1	3.04	3.69	2.42
OTTHUMG00000152645	3.04	3.82	2.55
KRTAP10-4	3.04	3.63	2.52
OTTHUMG00000161728	3.03	3.78	2.42
OTTHUMG00000183254	3.03	3.74	2.32
DEFB110	3.03	3.49	2.43
A2ML1-AS1	3.03	3.54	2.34
OTTHUMG00000041229	3.03	3.40	2.66
GOT1L1	3.03	3.20	2.85
DMC1	3.03	3.56	2.69
KRTAP19-4	3.03	3.62	2.70
RNU6-56P	3.03	3.54	2.62
SLC2A14	3.03	3.67	2.59
RPS10P7	3.03	3.33	2.73
DIO2-AS1	3.03	3.48	2.62
TRAJ16	3.03	4.31	1.89
TCEAL6	3.03	4.23	2.48
CCR8	3.03	3.38	2.50
GRIK2	3.03	3.57	2.53
GRIA4	3.03	3.70	2.59
DGCR6	3.03	3.72	2.55
CDH18	3.03	3.46	2.72
OTTHUMG00000155685	3.03	3.57	2.58
MIR146B	3.03	4.03	2.20
CHDC2	3.03	3.36	2.46
ST8SIA6	3.03	3.69	2.42
CENPH	3.03	3.33	2.54
KIAA1024L	3.03	3.38	2.69
TAS2R9	3.03	3.95	2.32
SNORD115-25	3.03	4.18	2.23
OTTHUMG00000151543	3.03	3.50	2.56
HSD17B13	3.02	3.47	2.70
OTTHUMG00000162886	3.02	3.72	2.58
LOC100129055	3.02	3.53	2.59
LOC100507205	3.02	3.55	2.57

RPL10L	3.02	3.44	2.63
RNA5SP501	3.02	3.84	1.88
LOC100130331	3.02	3.27	2.78
TTC6	3.02	3.35	2.80
CTAGE11P	3.02	3.89	2.34
RNA5SP31	3.02	3.51	2.64
ZNF230	3.02	3.48	2.50
LOC283352	3.02	3.75	2.67
CELA3A	3.02	3.58	2.49
LOC284998	3.02	3.83	2.51
UTS2	3.02	3.57	2.42
OTTHUMG00000165019	3.02	3.74	2.66
TYRP1	3.02	3.53	2.55
RNA5SP388	3.02	4.92	2.03
OTTHUMG00000151362	3.02	3.36	2.72
MAP3K19	3.02	3.46	2.71
HS6ST3	3.02	3.35	2.67
AGBL5-AS1	3.02	4.13	2.65
OR8G5	3.02	3.89	2.54
OTTHUMG00000170435	3.02	3.30	2.80
TCEB3B	3.02	3.46	2.43
OTTHUMG00000168950	3.02	3.39	2.61
OTTHUMG00000150317	3.02	3.25	2.82
NXF4	3.02	3.78	2.46
OTTHUMG00000162767	3.02	3.38	2.60
MIR514A1	3.01	4.44	2.11
OTTHUMG00000161739	3.01	3.49	2.51
OTTHUMG00000160219	3.01	3.65	2.58
GDNF-AS1	3.01	3.42	2.73
RNA5SP215	3.01	3.92	2.42
FAM24A	3.01	3.35	2.77
ZNF804A	3.01	3.27	2.72
LOC729506	3.01	3.28	2.67
OTTHUMG00000163132	3.01	3.33	2.69
MIR199A1	3.01	3.89	2.39
OTTHUMG00000037789	3.01	3.77	2.45
MIR3690	3.01	3.65	2.31
LOC100130452	3.01	3.24	2.73
CNTN6	3.01	3.55	2.49
TRAJ19	3.01	4.60	2.08
OTTHUMG00000167285	3.01	3.65	2.35
APOBEC3A	3.01	3.40	2.71
OTTHUMG00000169544	3.01	3.36	2.73
OTTHUMG00000162551	3.01	3.40	2.69
C12orf56	3.01	3.99	2.59
OTTHUMG00000019009	3.01	3.40	2.66
C14orf105	3.01	3.41	2.69
FAM194B	3.01	3.32	2.78
GDF9	3.01	3.19	2.74

LOC285878	3.01	3.37	2.70
REG1P	3.01	3.41	2.50
APOBEC1	3.01	3.80	2.43
TRAV8-1	3.01	3.92	2.51
PATE3	3.01	3.34	2.33
VNN2	3.01	3.36	2.49
OTTHUMG00000154769	3.01	3.37	2.74
APBA2	3.01	3.43	2.41
OR13H1	3.01	3.37	2.53
CALB1	3.01	3.51	2.69
FIGLA	3.01	3.64	2.65
LCE1B	3.01	3.57	2.47
MKRN3	3.01	3.58	2.61
OTTHUMG00000163476	3.01	3.53	2.43
BRWD1-IT1	3.01	3.79	2.48
ELAVL2	3.00	3.93	2.15
NIPAL1	3.00	3.33	2.58
EVPLL	3.00	3.49	2.48
OTTHUMG00000162888	3.00	3.64	2.58
SMG7-AS1	3.00	3.51	2.33
RNF148	3.00	3.21	2.68
MIR3909	3.00	3.40	2.60
OTTHUMG00000163634	3.00	3.39	2.75
OTTHUMG00000164180	3.00	4.11	2.43
ADAM18	3.00	3.53	2.67
C8orf34	3.00	3.43	2.73
OTTHUMG00000169070	3.00	3.39	2.70
OTTHUMG00000170901	3.00	3.53	2.72
KDM4E	3.00	3.21	2.83
MIR331	3.00	3.70	2.56
RNA5SP223	3.00	3.40	2.20
HSD3B2	3.00	3.62	2.42
TTPA	3.00	3.33	2.62
OTTHUMG00000152676	3.00	3.74	2.67
MIR4424	3.00	4.12	2.45
OTTHUMG00000012411	3.00	3.31	2.68
SH3GL3	3.00	3.45	2.67
LINC00877	3.00	3.41	2.47
NDST3	3.00	3.44	2.67
OTTHUMG00000154465	3.00	3.46	2.56
VTRNA1-2	3.00	3.85	2.52
SLC25A3P1	3.00	3.40	2.42
OTTHUMG00000018722	3.00	3.62	2.53
OTTHUMG00000166009	3.00	3.22	2.62
PTPRR	3.00	3.71	2.34
LOC100506071	3.00	3.34	2.62
OTTHUMG00000162785	3.00	3.35	2.58
SKA1	3.00	3.49	2.65
TPTE2P5	3.00	3.50	2.16

LOC644215	3.00	3.54	2.62
CFC1B	3.00	4.25	2.29
KRTAP5-3	3.00	3.61	2.33
OR52B4	3.00	3.33	2.67
OTTHUMG00000158839	3.00	3.85	2.57
LINC00862	2.99	3.33	2.64
RN7SKP4	2.99	4.26	1.95
FCGR1C	2.99	3.62	2.36
MCHR2	2.99	3.73	2.55
ANKRD33B-AS1	2.99	3.58	2.62
LINC00624	2.99	3.49	2.61
OTTHUMG00000015418	2.99	3.70	2.47
GLOD5	2.99	3.58	2.24
LOC100500773	2.99	3.36	2.50
TLR8-AS1	2.99	3.42	2.60
AKR1C4	2.99	3.74	2.70
UNC13C	2.99	3.87	2.66
IFNG-AS1	2.99	3.78	2.56
OTTHUMG00000015478	2.99	3.60	2.38
SATB2-AS1	2.99	3.31	2.58
IFNK	2.99	3.39	2.57
LOC541472	2.99	3.45	2.57
OTTHUMG00000171322	2.99	3.46	2.25
MAB21L3	2.99	3.53	2.44
MIR617	2.99	3.37	2.58
OR10R2	2.99	3.36	2.72
OTTHUMG00000036137	2.99	3.38	2.69
BBOX1	2.99	3.59	2.62
C1orf185	2.99	3.23	2.61
SRD5A2	2.99	3.39	2.69
SLITRK6	2.99	3.50	2.57
MIR196A1	2.99	3.92	2.39
OTTHUMG00000155471	2.99	3.54	2.49
LOC645752	2.99	3.91	2.49
RGS21	2.99	3.65	2.71
IFNA8	2.99	3.48	2.61
RNA5-8SP3	2.99	3.63	2.47
TBL1Y	2.99	3.40	2.17
LOC100507377	2.99	3.27	2.75
OTTHUMG00000164076	2.99	3.42	2.66
TMPRSS11B	2.99	3.38	2.58
FAM74A3	2.98	4.15	2.29
OTTHUMG00000164459	2.98	3.39	2.72
OTTHUMG00000165053	2.98	3.47	2.47
SCAND3	2.98	3.75	2.36
MIR26A1	2.98	3.25	2.56
OTTHUMG00000171681	2.98	3.33	2.47
G6PC2	2.98	3.27	2.69
LINC00264	2.98	3.68	2.26

SMIM21	2.98	3.72	2.38
OR14J1	2.98	3.67	2.78
OTTHUMG00000020094	2.98	3.28	2.62
SLCO4C1	2.98	3.54	2.51
OTTHUMG00000170520	2.98	3.52	2.26
LOC730100	2.98	3.66	2.38
PLAC1L	2.98	3.58	2.56
OTTHUMG00000162167	2.98	3.45	2.53
LOC100287944	2.98	3.57	2.28
OTTHUMG00000180237	2.98	3.69	2.32
OTTHUMG00000177159	2.98	3.29	2.72
OTTHUMG00000169466	2.98	3.65	2.31
OTTHUMG00000154970	2.98	3.33	2.58
UGT3A1	2.98	3.50	2.61
HIST1H2AB	2.98	3.47	2.52
OR5M10	2.98	3.46	2.43
PON1	2.98	3.79	2.55
BIRC6-AS1	2.98	3.35	2.54
LOC100505947	2.98	3.67	2.15
OTTHUMG00000166246	2.98	3.41	2.64
OTTHUMG00000170123	2.98	3.61	2.60
LIPM	2.98	3.29	2.66
LINC00948	2.98	3.33	2.66
OTTHUMG00000167990	2.97	3.62	2.75
OTTHUMG00000152394	2.97	3.84	2.68
SOX3	2.97	3.48	2.59
IL23R	2.97	3.37	2.67
OTTHUMG00000161575	2.97	3.35	2.16
OTTHUMG00000031780	2.97	3.55	2.55
PRDM13	2.97	3.30	2.66
SEPT14	2.97	3.49	2.60
OR10Q1	2.97	3.64	2.37
UBE2Q1-AS1	2.97	3.27	2.77
OTTHUMG00000151742	2.97	3.44	2.39
LOC645485	2.97	3.35	2.49
OTTHUMG00000175839	2.97	3.46	2.19
YEATS2-AS1	2.97	3.73	2.51
DYNLRB2	2.97	3.54	2.52
XKR7	2.97	3.28	2.63
RNA5SP318	2.97	4.56	2.18
OTTHUMG00000171374	2.97	3.45	2.64
SYT14	2.97	3.59	2.38
PLCE1-AS1	2.97	3.34	2.55
PCDH11Y	2.97	3.29	2.58
LINC00317	2.97	3.57	2.09
IL1RAPL2	2.97	3.40	2.35
LOC100129620	2.97	3.46	2.31
OTTHUMG00000161225	2.97	3.44	2.24
KBTBD12	2.97	3.54	2.63

LOC100506478	2.97	3.53	2.32
LOC145694	2.97	3.33	2.55
TBL1XR1-AS1	2.96	3.68	2.26
CADPS	2.96	3.31	2.71
MIR4513	2.96	3.39	2.47
SPACA7	2.96	3.58	2.72
OTTHUMG00000166516	2.96	3.31	2.63
SLC9C2	2.96	3.38	2.64
OTTHUMG00000017144	2.96	3.36	2.68
OTTHUMG00000181788	2.96	3.30	2.77
NPFFR2	2.96	3.19	2.69
MDGA2	2.96	3.29	2.63
RNA5SP494	2.96	4.29	1.84
PARD6B	2.96	3.27	2.65
OTTHUMG00000158945	2.96	3.18	2.65
PEX5L-AS2	2.96	3.50	2.61
ZNF582	2.96	3.34	2.54
DNAH12	2.96	3.24	2.59
OTTHUMG00000152377	2.96	3.90	2.65
ACOT4	2.96	3.28	1.87
LOC728084	2.96	3.28	2.73
P2RY12	2.96	3.55	2.47
OTTHUMG00000160738	2.96	3.49	2.63
ADH1A	2.96	3.63	2.52
FAM53A	2.96	3.45	2.16
LOC100507546	2.96	3.20	2.68
AREG	2.96	3.21	2.77
RNA5SP230	2.96	3.77	1.98
OTTHUMG00000020075	2.96	3.52	2.61
OR8D4	2.96	3.42	2.38
OTTHUMG00000180036	2.96	3.91	2.39
SNORD12	2.96	3.61	2.12
OR10V1	2.96	3.49	2.49
CR1	2.95	3.22	2.49
SUCLA2-AS1	2.95	3.59	2.24
OTTHUMG00000150053	2.95	3.75	2.59
DCUN1D2-AS1	2.95	3.49	2.57
TNIP3	2.95	3.45	2.49
NOL4	2.95	3.45	2.61
C1QTNF1-AS1	2.95	3.16	2.60
HOOK1	2.95	3.72	2.52
TRAJ38	2.95	3.64	2.25
PRAC	2.95	3.21	2.72
OTTHUMG00000163521	2.95	3.69	2.46
LINC00279	2.95	3.46	2.58
PDE11A	2.95	3.32	2.46
GML	2.95	3.23	2.60
LINC00507	2.95	3.45	2.56
F9	2.95	3.34	2.33

RNA5SP505	2.95	3.89	1.98
KRT73	2.95	3.27	2.58
LINC00477	2.95	3.46	2.44
TAS2R13	2.95	3.18	2.55
OTTHUMG00000165905	2.95	3.56	2.47
LINC00499	2.95	3.76	2.37
LINC00596	2.95	3.45	2.37
CYP2C18	2.95	3.61	2.36
PP12613	2.95	3.48	2.47
MIR4473	2.95	3.59	2.58
OR7A5	2.95	3.44	2.49
OTTHUMG00000008929	2.95	3.37	2.50
FAM72A	2.95	3.76	2.25
BTF3P11	2.95	3.58	2.64
DDX4	2.95	3.48	2.61
MIR148B	2.94	3.69	2.30
MIR550A3	2.94	4.35	2.36
OTTHUMG00000162881	2.94	3.64	2.50
TRIM51GP	2.94	3.54	2.44
TMEM156	2.94	3.37	2.69
LPPR5	2.94	3.46	2.52
OPCML-IT2	2.94	3.34	2.63
OTTHUMG00000161634	2.94	3.45	2.42
RBMY2FP	2.94	3.57	1.99
OTTHUMG00000166007	2.94	3.28	2.58
OTTHUMG00000171367	2.94	3.28	2.13
OLAH	2.94	3.33	2.48
ZNF573	2.94	3.22	2.35
OTTHUMG00000168465	2.94	3.70	2.40
OTTHUMG00000181983	2.94	3.55	2.43
MIR4706	2.94	3.70	2.49
GRXCR1	2.94	3.39	2.63
SYCP2L	2.94	3.20	2.52
CPB2	2.94	3.23	2.62
SLC24A2	2.94	3.24	2.66
CHL1-AS2	2.94	3.58	2.31
VENTXP7	2.94	3.35	2.53
LINC00238	2.94	3.33	2.43
MIR1255B1	2.94	3.25	2.75
TRIM77	2.94	3.23	2.67
OTTHUMG00000166558	2.94	3.36	2.59
OTTHUMG00000153757	2.94	3.59	2.55
KRT33A	2.94	3.64	2.43
OTTHUMG00000032884	2.94	3.58	2.39
LOC100506474	2.94	3.22	2.64
OTTHUMG00000172151	2.94	3.45	2.70
LINC00545	2.94	3.25	2.44
FAM27E1	2.94	3.70	2.09
OTTHUMG00000014123	2.93	3.56	2.60

FLJ25758	2.93	3.33	2.39
LOC283692	2.93	3.18	2.63
OTTHUMG00000171400	2.93	3.29	2.53
TMEM247	2.93	3.20	2.48
MIR3169	2.93	3.78	2.46
OTTHUMG00000159462	2.93	3.79	2.67
VN1R1	2.93	3.53	2.69
TRAV10	2.93	3.81	2.29
FLJ45964	2.93	3.27	2.67
OTTHUMG00000162559	2.93	3.17	2.46
CARD18	2.93	3.83	2.44
DEFA6	2.93	3.57	2.55
SERPINB10	2.93	3.47	2.44
SPP2	2.93	3.31	2.57
MIR4432	2.93	3.68	2.28
LOC728606	2.93	3.22	2.67
OTOR	2.93	3.23	2.50
ELOVL3	2.93	3.27	2.47
OTTHUMG00000171352	2.93	3.54	2.50
CHEK2P2	2.93	3.66	2.27
MIR23C	2.93	3.22	2.61
TMSB15A	2.93	3.83	1.92
MIR524	2.93	3.44	2.66
SCN7A	2.93	3.18	2.64
POMC	2.92	3.31	2.18
LINC00244	2.92	3.45	2.58
TRPC5OS	2.92	3.64	2.52
GPHB5	2.92	3.38	2.63
OTTHUMG00000018058	2.92	3.19	2.58
DEFB135	2.92	3.20	2.63
OTTHUMG00000171373	2.92	3.29	2.48
CELA2B	2.92	3.45	2.26
MND1	2.92	3.63	2.35
OTTHUMG00000164966	2.92	3.40	2.25
NRCAM	2.92	3.26	2.60
HSPB8	2.92	3.13	2.56
OTTHUMG00000167824	2.92	3.38	2.20
OTTHUMG00000017769	2.92	3.39	2.57
OTTHUMG00000163008	2.92	3.19	2.60
MIR296	2.92	3.97	2.10
OTTHUMG00000166257	2.92	3.39	2.23
LOC100129213	2.92	3.39	2.38
OTTHUMG00000158586	2.92	3.41	2.56
OTTHUMG00000000481	2.92	3.39	2.52
MAGEB2	2.92	3.48	2.28
IL22RA2	2.92	3.50	2.55
SKAP1	2.92	3.26	2.43
OTTHUMG00000172163	2.92	3.31	2.39
OTTHUMG00000171798	2.92	3.61	2.35

OTTHUMG00000165261	2.92	3.42	2.61
OTTHUMG00000019540	2.92	3.59	2.12
OTTHUMG00000167330	2.92	3.62	2.49
ZPBP2	2.92	3.54	2.69
OR8K5	2.91	3.40	2.30
MIR100	2.91	4.14	2.01
OR1C1	2.91	3.49	2.45
SCARNA18	2.91	3.35	2.38
PEX5L-AS1	2.91	3.96	2.11
CHST9	2.91	3.44	2.62
IMPG1	2.91	3.32	2.50
OR4F6	2.91	3.24	2.55
WDR87	2.91	3.30	2.54
RNU7-19P	2.91	4.44	2.12
HIST1H4B	2.91	3.66	2.19
OTTHUMG00000032473	2.91	3.44	2.46
TLL2	2.91	3.71	2.48
HEMGN	2.91	3.72	2.34
OTTHUMG00000163792	2.91	3.39	2.50
FAM19A3	2.91	3.23	2.51
RNA5SP119	2.91	4.75	1.91
PIH1D3	2.91	3.27	2.53
OTTHUMG00000160894	2.91	3.41	2.52
GRPR	2.91	3.21	2.69
LOC257358	2.91	3.38	2.42
OTTHUMG00000162882	2.91	3.49	2.60
MIRLET7F1	2.91	4.77	1.60
LINC00901	2.91	3.29	2.33
PYDC1	2.91	3.50	2.24
ZFP42	2.91	3.59	2.50
C2orf83	2.91	3.44	2.49
OR6A2	2.91	3.29	2.66
SLC26A5	2.91	3.40	2.31
MIR942	2.91	3.39	2.64
LOC100506827	2.91	3.26	2.47
OTTHUMG00000161740	2.91	3.24	2.60
OTTHUMG00000174454	2.91	3.20	2.69
SSX9	2.91	3.54	2.34
OTTHUMG00000169418	2.91	3.08	2.46
OTTHUMG00000018736	2.91	3.63	2.41
RNU6-52P	2.91	4.09	2.15
OTTHUMG00000165334	2.91	3.38	2.40
ACTBL2	2.91	3.61	2.12
SDR16C6P	2.91	3.43	2.65
NPY5R	2.91	3.24	2.56
CBX3P2	2.91	3.14	2.69
OTTHUMG00000161342	2.91	3.68	2.47
OTTHUMG00000015400	2.91	3.38	2.57
SLC15A5	2.91	3.59	2.50

LOC100996263	2.90	3.13	2.52
ENKUR	2.90	3.36	2.57
LOC653653	2.90	3.63	2.25
OTTHUMG00000159221	2.90	3.36	2.47
PDE6C	2.90	3.26	2.66
MIR608	2.90	3.19	2.59
MOXD2P	2.90	3.53	2.29
OTTHUMG00000169456	2.90	3.26	2.57
CDH9	2.90	3.36	2.54
OTTHUMG00000172020	2.90	3.32	2.42
OTTHUMG00000161213	2.90	3.37	1.94
CPA6	2.90	3.42	2.61
KGFLP1	2.90	3.25	2.32
LINC00235	2.90	3.45	2.52
OTTHUMG00000158949	2.90	3.41	2.62
OTTHUMG00000150597	2.90	3.68	2.47
OR10J1	2.90	3.59	2.55
FAM19A1	2.90	3.37	2.37
OTTHUMG00000160486	2.90	3.29	2.56
P2RY10	2.90	3.29	2.61
OTTHUMG00000153650	2.90	3.28	2.52
LOC100293704	2.90	3.22	2.65
OTTHUMG00000008362	2.90	3.37	2.51
OTTHUMG00000147348	2.90	3.55	2.37
TRIM51HP	2.90	3.67	2.43
OTTHUMG00000010834	2.90	3.44	2.45
MIR490	2.90	3.24	2.61
OTTHUMG00000020140	2.90	3.39	2.17
LOC441461	2.90	3.57	2.47
LINC00930	2.90	3.69	2.58
RET	2.90	3.42	2.55
CCDC150	2.90	3.14	2.74
C7orf62	2.90	3.61	2.44
SPRR1B	2.89	3.48	2.13
SLC10A4	2.89	3.14	2.52
OTTHUMG00000171593	2.89	3.30	2.62
SPINK13	2.89	3.25	2.49
PRODH	2.89	3.23	2.68
PCDHB1	2.89	3.34	2.41
C9orf135	2.89	3.51	2.42
HPGD	2.89	3.61	2.40
CXorf22	2.89	3.13	2.38
OTTHUMG00000160645	2.89	3.54	2.42
AREGB	2.89	3.16	2.55
OTTHUMG00000170069	2.89	3.17	2.51
MIR4678	2.89	4.46	2.13
MGAM	2.89	3.46	2.27
OTTHUMG00000165532	2.89	3.62	2.50
OTTHUMG00000161848	2.89	3.40	2.11

MIR938	2.89	3.68	2.11
TMPRSS11A	2.89	3.16	2.53
SPO11	2.89	3.16	2.39
PLK4	2.89	3.31	2.49
MS4A2	2.89	3.35	2.52
MIR141	2.89	3.30	2.53
OTTHUMG00000173096	2.89	3.51	2.44
RNA5SP247	2.89	4.41	2.11
TMEM232	2.89	3.17	2.38
OTTHUMG00000164715	2.89	3.31	2.42
MIR548E	2.89	3.45	2.22
AKR1D1	2.89	3.28	2.59
RNY3P2	2.89	3.77	2.33
CLEC12B	2.89	3.45	2.67
OTTHUMG00000151379	2.89	3.32	2.48
PROX1-IT1	2.88	3.34	2.59
OTTHUMG00000170491	2.88	3.51	2.46
UGT3A2	2.88	3.09	2.72
FFAR2	2.88	3.29	2.59
OTTHUMG00000152389	2.88	3.47	2.50
THEG5	2.88	3.18	2.42
LOC154872	2.88	3.29	2.48
MIR550B2	2.88	3.42	2.25
MMP26	2.88	3.32	2.39
PXT1	2.88	3.38	2.49
FGF12-AS1	2.88	3.46	2.22
DSCAM-IT1	2.88	3.30	2.67
OTTHUMG00000163854	2.88	3.68	2.34
OTTHUMG00000160725	2.88	3.09	2.65
AP1S3	2.88	3.31	2.42
OTTHUMG00000183122	2.88	3.50	2.53
TAS2R50	2.88	3.68	2.43
OTTHUMG00000158448	2.88	3.44	2.60
OTTHUMG00000163870	2.88	3.36	2.58
LOC100506731	2.88	3.19	2.35
OTTHUMG00000175929	2.88	3.64	2.36
ARSF	2.88	3.20	2.66
LINC00664	2.88	3.16	2.64
TMPRSS11F	2.88	3.30	2.53
TTC30B	2.88	3.50	2.33
C18orf63	2.88	3.26	2.71
CHRNB3	2.88	3.36	2.17
ACTC1	2.88	3.41	2.43
GALNTL5	2.88	3.19	2.60
OTTHUMG00000161238	2.88	3.15	2.55
LINC00935	2.88	3.13	2.65
KRTAP4-8	2.88	3.66	2.30
OTTHUMG00000019968	2.88	3.34	2.32
ZNF799	2.88	3.29	2.03

LINC00578	2.88	3.15	2.59
MIR199B	2.87	3.17	2.46
OTTHUMG00000151805	2.87	3.27	2.59
OTTHUMG00000168098	2.87	3.18	2.63
OR5AK2	2.87	3.20	2.60
DSG4	2.87	3.41	2.58
OTTHUMG00000169214	2.87	3.80	2.42
LOC647323	2.87	3.39	2.57
LOC400655	2.87	3.27	2.64
ARL5B-AS1	2.87	3.45	2.56
HES5	2.87	3.29	2.63
PPM1E	2.87	3.49	2.48
SLC9B1	2.87	3.23	2.38
OTTHUMG00000019643	2.87	3.16	2.47
MIR301B	2.87	3.28	2.25
OTTHUMG00000153820	2.87	3.43	2.50
SNORD125	2.87	3.37	2.47
OTTHUMG00000153025	2.87	3.18	2.58
RBM24	2.87	3.23	2.57
OTTHUMG00000150054	2.87	3.04	2.49
HNRNPA3P1	2.87	3.22	2.63
RACGAP1P	2.87	3.05	2.70
PLSCR2	2.87	3.37	2.43
OTTHUMG00000163519	2.87	3.47	2.23
OTTHUMG00000159649	2.87	3.02	2.70
PPP1R36	2.87	3.06	2.61
VWA8-AS1	2.87	3.31	2.61
LOC392232	2.86	3.31	2.51
MAGEA6	2.86	3.27	2.30
MS4A5	2.86	3.42	2.47
RNA5SP235	2.86	3.67	2.24
OTTHUMG00000152841	2.86	3.35	2.28
FLJ32955	2.86	3.14	2.59
OTTHUMG00000009217	2.86	3.22	2.67
OTTHUMG00000153099	2.86	3.78	2.28
TRAV18	2.86	3.57	2.37
OTTHUMG00000153424	2.86	3.35	2.46
OTTHUMG00000009304	2.86	3.51	2.27
OR6C68	2.86	3.26	2.34
SNORD114-18	2.86	4.11	2.15
SNORD9	2.86	3.76	1.81
C5orf58	2.86	3.28	2.55
STAC	2.86	3.27	2.35
GABRA6	2.86	3.27	2.48
OTTHUMG00000162673	2.86	3.46	2.28
OTTHUMG00000162701	2.86	3.57	2.36
EGFLAM-AS3	2.86	3.34	2.61
HIST1H2BN	2.86	3.61	2.44
TBPL2	2.86	3.50	2.61

ARMC3	2.86	3.14	2.57
LOC285423	2.86	3.13	2.74
OTTHUMG00000159412	2.86	3.20	2.48
LRRC19	2.86	3.33	2.47
OTTHUMG00000153144	2.86	3.23	2.48
OTTHUMG00000153665	2.86	3.19	2.50
LOC340017	2.86	3.64	2.53
AMELY	2.86	3.44	2.40
PACRG-AS1	2.86	3.09	2.21
OTTHUMG00000032515	2.86	3.45	2.39
MIR4797	2.86	3.46	2.18
RNA5SP188	2.85	3.78	1.88
MIR216A	2.85	3.39	2.41
KRT27	2.85	3.55	2.44
MIR3186	2.85	3.39	2.47
ZNF728	2.85	3.48	2.46
PRAMEF11	2.85	3.86	2.23
OR1N2	2.85	3.35	2.50
MIR3122	2.85	3.23	2.38
OTTHUMG00000171308	2.85	3.38	2.37
ITK	2.85	3.37	2.39
OTTHUMG00000154935	2.85	3.20	2.55
BMPR1B	2.85	3.61	2.21
OR3A2	2.85	3.35	2.24
HSPB3	2.85	3.23	2.52
GATSL2	2.85	3.44	2.22
BSN-AS2	2.85	3.30	2.53
MIR520G	2.85	3.25	2.33
MIR17HG	2.85	3.14	2.46
OTTHUMG00000159626	2.85	3.11	2.50
OTTHUMG00000153425	2.85	3.53	2.18
ANKRD34B	2.85	3.02	2.43
SUN3	2.85	3.28	2.53
LOC644248	2.85	3.55	2.42
LPPR1	2.85	3.11	2.35
OTTHUMG00000163613	2.84	3.31	2.48
SLC9C1	2.84	3.15	2.58
IL4	2.84	3.51	2.28
TRAV34	2.84	3.38	2.37
MYO3A	2.84	3.35	2.35
OTTHUMG00000163174	2.84	3.24	2.43
LOC100507391	2.84	3.47	2.53
OTTHUMG00000163987	2.84	3.38	2.45
MIR3116-1	2.84	3.92	2.15
MIR508	2.84	3.33	2.54
CALR3	2.84	3.32	2.52
OTTHUMG00000074543	2.84	3.24	2.37
DYDC2	2.84	3.10	2.61
MIR4501	2.84	3.72	2.21

RN7SKP2	2.84	3.39	2.32
OTTHUMG00000165348	2.84	3.82	2.51
VTRNA1-1	2.84	3.56	2.37
OTTHUMG00000031862	2.84	3.34	2.49
OTTHUMG00000064344	2.84	3.39	2.51
RNA5SP270	2.84	3.42	2.44
LINC00669	2.84	3.32	2.44
MAGEB10	2.84	3.64	2.39
OTTHUMG00000162463	2.84	3.06	2.65
OTTHUMG00000163580	2.84	3.72	2.32
SEMG2	2.84	3.73	2.43
KRT19P1	2.84	3.56	2.15
SAMSN1-AS1	2.84	3.06	2.58
OR5T1	2.84	3.06	2.59
LOC339622	2.84	3.55	2.38
TMEFF2	2.84	3.18	2.51
OR6C2	2.84	3.66	2.45
OTTHUMG00000151319	2.84	3.19	2.33
RNU6-78P	2.84	3.35	2.06
TRAT1	2.84	3.56	2.43
LINC00968	2.84	3.44	2.13
LOC100287082	2.84	3.40	2.58
RNA5SP56	2.84	4.24	1.98
LOC646813	2.84	3.70	2.35
OTTHUMG00000170592	2.84	3.26	2.52
SPATA4	2.84	3.10	2.52
MIR374A	2.84	4.09	2.09
OTTHUMG00000140117	2.84	3.05	2.67
OTTHUMG00000164047	2.83	3.06	2.43
RNA5SP347	2.83	3.59	2.19
RNU6ATAC3P	2.83	3.49	1.94
LOC100132272	2.83	3.21	2.40
FAM86FP	2.83	3.09	2.58
ACE2	2.83	3.19	2.52
C8orf22	2.83	3.27	2.40
LOC729083	2.83	3.31	2.53
LINC00261	2.83	3.39	2.48
OTTHUMG00000172372	2.83	3.30	2.19
LOC643770	2.83	3.13	2.67
LOC100507387	2.83	3.15	2.26
HRG	2.83	3.47	2.28
TRAJ21	2.83	4.06	2.22
OTTHUMG00000017135	2.83	3.73	2.17
GSTTP2	2.83	3.56	2.40
PABPC5-AS1	2.83	3.22	2.44
RNA5SP496	2.83	3.60	1.91
ZPBP	2.83	3.19	2.58
NCRNA00185	2.83	3.92	1.68
OTTHUMG00000183414	2.83	3.55	2.20

TAS2R1	2.83	3.10	2.49
LINC00923	2.83	3.40	2.53
OTTHUMG00000154343	2.83	3.50	2.36
OTTHUMG00000020560	2.83	3.15	2.41
LOC100507657	2.83	3.71	2.39
OTTHUMG00000032150	2.83	3.38	2.35
SCARNA23	2.83	3.81	2.21
LOC100289533	2.83	3.25	2.38
MIR3129	2.83	3.38	2.31
LOC284661	2.82	3.19	2.46
OTTHUMG00000163836	2.82	3.09	2.34
RNA5SP510	2.82	3.49	1.99
OTTHUMG00000156569	2.82	3.40	2.30
ANKRD30BP3	2.82	3.69	2.35
CLEC4M	2.82	3.18	2.41
OR56A4	2.82	3.63	2.47
GPR50-AS1	2.82	3.21	2.52
OTTHUMG00000020459	2.82	3.27	2.51
SLCO6A1	2.82	3.04	2.61
LOC101060226	2.82	3.52	2.30
OTTHUMG00000039859	2.82	3.40	2.42
RNA5SP390	2.82	3.48	2.17
C6orf118	2.82	3.22	2.50
SMIM18	2.82	3.32	2.48
OTTHUMG00000171627	2.82	3.40	2.26
CLYBL-IT1	2.82	3.59	2.42
METTL11B	2.82	3.10	2.44
TPTE2P3	2.82	3.40	2.57
OTTHUMG00000162404	2.82	3.20	2.44
MIR4776-1	2.82	3.30	2.19
LINC00327	2.82	3.10	2.56
MIR20B	2.82	3.76	1.88
RNA5SP143	2.82	4.14	1.81
STXBP5L	2.82	3.11	2.47
FAM47C	2.82	3.60	2.37
MIR3974	2.82	4.13	2.26
OTTHUMG00000170739	2.82	3.15	2.58
VN1R10P	2.82	3.74	2.32
OTTHUMG00000170397	2.81	3.17	2.60
LINC00687	2.81	3.25	2.42
OTTHUMG00000037977	2.81	3.48	2.27
SNORD116-29	2.81	3.51	2.13
IGHV3-72	2.81	3.58	2.09
OTTHUMG00000177076	2.81	3.56	2.39
CSHL1	2.81	3.28	2.30
CDKN2B-AS1	2.81	3.21	2.36
RNA5SP96	2.81	3.81	2.50
FAM182A	2.81	3.50	2.35
HECW1-IT1	2.81	3.32	2.46

OR2Y1	2.81	3.13	2.51
TRBV5-3	2.81	3.71	2.23
OTTHUMG00000160686	2.81	3.45	2.29
KLHL38	2.81	3.39	2.38
OR5D18	2.81	3.02	2.49
OTTHUMG00000166476	2.81	3.06	2.37
OTTHUMG00000179488	2.81	3.27	2.46
CLCA4	2.81	3.16	2.29
BNC1	2.81	3.22	2.39
OR4B1	2.81	3.29	2.23
MIR4511	2.81	4.32	2.10
OTTHUMG00000017601	2.81	3.57	2.09
SNORD116-11	2.81	4.10	1.68
OTTHUMG00000166075	2.81	3.37	2.19
OR52N1	2.81	3.23	2.36
KRTAP21-2	2.81	3.14	2.54
LECT2	2.81	3.39	2.51
FGL1	2.81	3.34	2.24
OTTHUMG00000164158	2.81	3.27	2.35
OTTHUMG00000008933	2.80	3.13	2.20
MIR874	2.80	3.27	2.16
PTCHD3	2.80	3.16	2.43
BMX	2.80	3.11	2.44
OTTHUMG00000000405	2.80	3.30	2.45
OTTHUMG00000171800	2.80	3.22	2.29
OTTHUMG00000153783	2.80	3.60	2.40
NBPF10	2.80	3.70	2.28
ATP2B3	2.80	3.41	2.48
TTTY3	2.80	3.29	2.29
RNA5SP24	2.80	3.16	2.44
PCDH9-AS4	2.80	3.50	2.25
EGFLAM-AS2	2.80	3.80	2.07
OR52D1	2.80	3.88	2.37
RAG1	2.80	3.34	2.47
C1orf168	2.80	3.52	2.21
SNORD18A	2.80	3.32	2.02
OTTHUMG00000155424	2.80	3.20	2.37
MIR448	2.80	3.11	2.54
MIR1193	2.80	3.53	2.25
OTTHUMG00000133752	2.80	3.87	1.97
TCTEX1D1	2.80	3.32	2.32
LINC00588	2.80	3.25	2.28
AA06	2.80	3.10	2.15
PCP4	2.80	3.01	2.59
OTTHUMG00000183978	2.80	3.13	2.57
CCNA1	2.80	3.21	2.40
OTTHUMG00000163257	2.79	3.19	2.48
RNU6-68P	2.79	3.28	2.33
OR5AK4P	2.79	3.27	2.49

CCR3	2.79	3.44	2.52
GLRA3	2.79	3.69	2.31
TMEM212	2.79	3.17	2.48
OTTHUMG00000160614	2.79	3.40	2.27
OTTHUMG00000159743	2.79	3.32	2.41
OTTHUMG00000013089	2.79	3.60	1.51
NBEAP1	2.79	3.78	2.18
C11orf42	2.79	3.33	2.21
H2BFWT	2.79	3.47	2.44
OTTHUMG00000065060	2.79	3.36	2.07
TAAR6	2.79	3.30	2.52
MIR2116	2.79	3.60	2.14
KRT28	2.79	3.05	2.55
C3orf79	2.79	3.25	2.36
OTTHUMG00000042020	2.79	3.76	2.42
OTTHUMG00000020437	2.79	3.51	2.43
ASB17	2.79	3.26	2.25
OTTHUMG00000159132	2.79	3.27	2.27
SIGLEC17P	2.79	3.48	1.98
OTTHUMG00000172322	2.79	3.15	2.40
MTFR2	2.79	3.09	2.49
TRAJ14	2.79	3.86	2.16
LUZP2	2.79	3.05	2.56
OTTHUMG00000153530	2.79	3.12	2.56
C12orf40	2.79	3.07	2.35
OTTHUMG00000168914	2.79	3.43	2.21
OTTHUMG00000164915	2.79	4.10	2.28
MIR3156-3	2.79	3.02	2.56
OTTHUMG00000149338	2.79	2.94	2.62
LOC100128668	2.79	3.61	2.05
RNA5SP107	2.78	3.31	2.42
CRYGB	2.78	3.59	2.28
MIR3156-2	2.78	3.12	2.55
ERICH2	2.78	3.34	2.40
OTTHUMG00000162918	2.78	3.34	2.26
OTTHUMG00000003519	2.78	3.23	2.30
OTTHUMG00000155286	2.78	3.50	2.34
MIR590	2.78	3.83	2.21
KC6	2.78	3.60	2.29
MUC13	2.78	3.38	2.50
CLDN8	2.78	3.13	2.42
OTTHUMG00000154544	2.78	3.07	2.41
OTTHUMG00000058813	2.78	3.37	2.15
LOC286058	2.78	3.19	2.36
OTTHUMG00000151787	2.78	3.41	2.09
MIR506	2.78	3.46	1.74
LOC100506348	2.78	3.59	2.25
SERPINB12	2.78	3.37	2.30
LOC144017	2.78	3.05	2.29

HSP90B3P	2.78	4.26	1.99
MIR29A	2.78	3.64	2.33
OTTHUMG00000165095	2.78	3.35	2.33
LINC00293	2.78	3.21	2.39
TAS2R20	2.78	3.64	2.12
MIR520H	2.78	3.47	2.42
OR4F15	2.78	3.20	2.20
TEX26	2.78	3.06	2.46
OTTHUMG00000179714	2.78	3.07	2.37
MIR495	2.78	3.38	2.18
OTTHUMG00000154036	2.78	2.98	2.37
LINC00491	2.77	3.02	2.45
OTTHUMG00000008050	2.77	3.29	2.27
OTTHUMG00000164613	2.77	3.14	2.13
OTTHUMG00000014223	2.77	3.49	2.23
OTTHUMG00000168235	2.77	4.02	1.84
C15orf43	2.77	3.25	2.50
ART3	2.77	3.22	2.44
OTTHUMG00000169997	2.77	3.22	2.23
OTTHUMG00000165844	2.77	3.37	2.31
MIR544B	2.77	3.83	1.78
OTTHUMG00000020777	2.77	3.13	2.26
OTTHUMG00000154827	2.77	3.08	2.32
ATP6V1G3	2.77	3.16	2.30
OTTHUMG00000155483	2.77	3.11	2.54
OTTHUMG00000162359	2.77	3.11	2.56
MTRNR2L7	2.77	3.10	2.29
MIR4744	2.77	3.84	2.16
TRAV39	2.77	3.44	2.22
DPRX	2.77	3.21	2.22
ARSE	2.77	3.06	2.39
OTTHUMG00000153548	2.77	2.89	2.50
OTTHUMG00000158300	2.77	3.26	2.41
SNORD127	2.77	3.56	2.07
PRAMEF12	2.77	3.23	2.26
OTTHUMG00000021409	2.77	3.24	2.50
OR10W1	2.77	3.28	2.21
OTTHUMG00000019446	2.76	3.42	2.20
IGHV3-49	2.76	3.31	2.04
OR5H6	2.76	3.37	2.36
C3orf33	2.76	3.04	2.34
OTTHUMG00000151898	2.76	3.28	2.28
TCTE3	2.76	3.34	2.22
LOC100132501	2.76	3.27	2.45
OTTHUMG00000009148	2.76	3.08	2.35
SLC17A3	2.76	3.13	2.44
MIR3136	2.76	3.36	2.40
OTTHUMG00000162331	2.76	3.24	2.44
SPRR2D	2.76	3.13	2.16

OR1L4	2.76	3.78	2.11
C6orf10	2.76	3.37	2.15
OTTHUMG00000059209	2.76	3.34	2.33
RNA5SP104	2.76	4.21	2.19
ALX1	2.76	3.31	2.46
FAM194A	2.76	3.09	2.49
OTTHUMG00000161641	2.76	3.19	2.30
OTTHUMG00000158577	2.76	3.40	2.26
SCGB1D4	2.75	3.37	2.44
LINC00698	2.75	3.27	2.09
CDC14C	2.75	3.36	2.33
SLC5A8	2.75	3.04	2.50
PLCH1-AS2	2.75	3.04	2.39
OTTHUMG00000156017	2.75	3.19	2.43
SLC7A13	2.75	3.08	2.39
PTPRQ	2.75	3.21	2.50
LINC00865	2.75	3.17	2.41
LYZL1	2.75	3.70	2.18
KLRD1	2.75	3.00	2.48
NPSR1-AS1	2.75	3.23	2.36
OTTHUMG00000163288	2.75	3.26	1.98
KRTAP20-1	2.75	3.19	2.31
ZSWIM2	2.75	3.01	2.43
NTS	2.75	3.59	2.40
DCLK3	2.75	3.12	2.36
HIST1H2BG	2.75	3.29	2.33
OTTHUMG00000074159	2.75	3.11	2.15
OTTHUMG00000017965	2.75	3.42	2.19
LOC100129186	2.75	3.72	2.36
OTTHUMG00000153098	2.75	3.07	2.24
OTTHUMG00000164759	2.75	3.13	2.46
IFLTD1	2.75	3.16	2.29
TTTY19	2.74	3.10	2.43
HIST1H2BA	2.74	3.38	1.95
VIPR1-AS1	2.74	3.79	2.15
OTTHUMG00000154852	2.74	3.12	2.47
OTTHUMG00000158383	2.74	3.12	2.29
GABRG1	2.74	3.30	2.38
OTTHUMG00000161601	2.74	3.03	2.38
FOXD3	2.74	3.26	2.28
OTTHUMG00000172035	2.74	3.15	2.40
OTTHUMG00000158948	2.74	3.14	2.55
RNA5SP245	2.74	4.34	1.82
MIR4765	2.74	3.42	2.34
LINC00302	2.74	3.60	2.18
MIR767	2.74	3.20	2.38
KCNB2	2.74	3.11	2.39
LOC284294	2.74	3.19	2.24
PRMT5-AS1	2.74	3.02	2.38

MED27	2.74	3.76	2.10
MIR516A1	2.74	3.89	2.01
CST9LP1	2.74	3.24	2.29
SLC27A6	2.74	3.12	2.46
OR2C3	2.74	3.40	2.16
ATP5EP2	2.74	2.99	2.41
LINC00709	2.74	3.53	2.26
CLECL1	2.74	3.24	2.36
TRAV8-2	2.74	3.27	2.11
OTTHUMG00000164295	2.74	3.10	2.39
KCNH8	2.74	2.95	2.47
CNTNAP4	2.74	3.29	2.42
TRPC4	2.74	3.11	2.30
COX8C	2.74	3.24	2.46
OTTHUMG00000166512	2.74	3.18	2.30
OTTHUMG00000164525	2.74	3.26	2.41
OTTHUMG00000161216	2.74	3.88	2.13
S100A7L2	2.74	3.13	2.34
OTTHUMG00000178685	2.73	3.22	2.24
SNORD114-23	2.73	4.79	1.83
TRAJ33	2.73	3.53	2.43
SYNPR	2.73	3.10	2.50
OR52B2	2.73	3.08	2.41
OTTHUMG00000017201	2.73	3.02	2.53
OR10AG1	2.73	3.08	2.44
CYLC1	2.73	2.92	2.53
OTTHUMG00000159748	2.73	3.00	2.46
OTTHUMG00000078324	2.73	3.24	2.40
LINC00571	2.73	3.19	2.10
ROPN1	2.73	3.74	1.71
CBLC	2.73	3.20	2.38
OTTHUMG00000158360	2.73	3.05	2.35
MIR3664	2.73	3.32	2.44
C15orf54	2.73	3.40	2.24
OTTHUMG00000154623	2.73	3.39	2.36
MIR2355	2.73	3.71	2.13
TRIM42	2.73	3.45	2.24
TAAR9	2.73	3.07	2.31
SNORD97	2.73	3.26	2.52
GAGE1	2.73	3.13	2.06
CXorf66	2.73	3.60	2.39
ZFX-AS1	2.73	3.07	2.24
MIR96	2.73	3.15	2.38
LOC645949	2.73	3.49	2.01
OTTHUMG00000162459	2.73	3.03	2.32
OTTHUMG00000168552	2.73	3.27	2.22
OTTHUMG00000010019	2.73	3.01	2.30
CHRM5	2.73	3.08	2.13
SNORD83A	2.73	3.12	2.23

C11orf94	2.73	3.19	2.30
OTTHUMG00000152740	2.73	3.32	2.39
CRISP2	2.73	3.12	2.50
OTTHUMG00000015393	2.72	3.15	2.19
OTTHUMG00000160299	2.72	3.18	2.19
SNORA2B	2.72	3.31	2.24
CLEC4D	2.72	2.97	2.42
OR2D3	2.72	3.11	2.42
OTTHUMG00000074167	2.72	3.13	2.56
LOC100996552	2.72	3.29	1.95
MIR138-1	2.72	3.22	2.34
FGF22	2.72	3.17	2.15
TYRO3P	2.72	3.59	2.28
DCT	2.72	3.15	2.29
OTTHUMG00000169867	2.72	3.20	2.14
OTTHUMG00000156583	2.72	3.06	2.24
PAR4	2.72	3.01	2.42
OTTHUMG00000164463	2.72	3.30	2.40
ACOT12	2.72	3.11	2.34
HORMAD2	2.72	3.08	2.39
RNA5SP373	2.72	3.96	1.95
OTTHUMG00000161004	2.72	3.46	2.36
CER1	2.71	3.13	2.42
C2orf16	2.71	3.53	2.19
OTTHUMG00000164208	2.71	3.35	2.23
TRAJ1	2.71	3.73	1.92
C10orf131	2.71	3.77	2.27
LOC441009	2.71	3.33	2.23
CCDC38	2.71	3.53	2.32
IGHV3-43	2.71	3.68	1.70
OTTHUMG00000162581	2.71	3.06	2.37
OTTHUMG00000168375	2.71	2.89	2.30
OTTHUMG00000019020	2.71	2.91	2.41
CT64	2.71	2.90	2.47
OTTHUMG00000163316	2.71	3.34	2.14
LRIT3	2.71	3.26	2.43
ORM1	2.71	3.04	2.40
MAST4-IT1	2.71	3.40	2.20
OTTHUMG00000153725	2.71	3.12	2.44
ATG10-IT1	2.71	3.71	2.34
OTTHUMG00000162804	2.71	3.30	2.19
IL1RAPL1	2.71	2.94	2.48
OTTHUMG00000157089	2.71	3.18	2.11
LUZP4	2.71	3.17	2.22
OTTHUMG00000156217	2.71	3.23	2.32
SPERT	2.70	3.19	2.25
MIR514A2	2.70	4.12	2.05
LOC101060553	2.70	3.15	2.42
OTTHUMG00000162824	2.70	3.12	2.22

KRTAP13-2	2.70	3.12	2.27
MIR190B	2.70	3.37	2.04
OTTHUMG00000160657	2.70	2.99	2.34
SPATA31A6	2.70	3.15	2.27
LOC100507652	2.70	3.01	2.31
OTTHUMG00000166329	2.70	3.14	2.33
OTTHUMG00000169738	2.70	3.20	2.28
SAMD12-AS1	2.70	3.00	2.43
LOC100133616	2.70	3.63	2.33
OTTHUMG00000012147	2.70	3.46	2.35
OR4K2	2.70	3.77	2.15
OTTHUMG00000033074	2.70	2.99	2.27
KRTAP19-2	2.70	3.71	1.89
KRTAP6-1	2.70	3.01	2.40
LOC100133123	2.70	2.96	2.31
MIR4500HG	2.70	3.11	2.14
OTTHUMG00000177856	2.70	2.97	2.37
PKIA	2.70	3.22	2.45
MIR1471	2.70	3.45	2.19
MIR4472-1	2.70	3.65	2.01
OTTHUMG00000172228	2.70	3.09	2.32
LOC100124692	2.69	3.12	2.28
OTTHUMG00000169866	2.69	3.79	2.25
ARL9	2.69	3.77	2.11
OTTHUMG00000021932	2.69	3.50	2.10
MIR519A1	2.69	4.42	1.85
MIR125B1	2.69	3.34	1.84
CNTN1	2.69	3.12	2.37
CXCL6	2.69	3.03	2.47
OTTHUMG00000160721	2.69	3.23	2.21
RAB39A	2.69	3.07	2.24
OTTHUMG00000024194	2.69	3.24	2.21
MMP10	2.69	3.31	2.33
OTTHUMG00000018255	2.69	3.34	2.49
OTTHUMG00000169922	2.69	3.28	2.38
CCSER1	2.69	2.99	2.31
SAA3P	2.69	3.54	2.22
SNORD114-20	2.69	4.87	1.48
MIR337	2.69	3.03	2.36
HRH4	2.69	3.06	2.24
OTTHUMG00000031835	2.69	3.02	2.20
OTTHUMG00000148688	2.69	3.20	2.34
RNU6ATAC4P	2.69	3.38	1.94
OTTHUMG00000158468	2.69	3.07	2.22
OTTHUMG00000036359	2.69	3.10	2.20
MIR126	2.69	4.20	2.22
GRM7-AS2	2.69	3.10	2.26
OTTHUMG00000015769	2.68	3.40	2.11
LOC441455	2.68	3.28	2.15

XAGE5	2.68	3.28	2.30
LINC00676	2.68	3.22	2.23
C4orf22	2.68	3.28	2.31
MIR1-1	2.68	3.52	1.96
OTTHUMG00000164634	2.68	3.39	1.83
INSM2	2.68	3.29	2.39
OTTHUMG00000032035	2.68	3.48	2.22
OTTHUMG00000035042	2.68	4.23	2.15
IAPP	2.68	3.24	2.29
SNORA70F	2.68	3.49	2.12
KERA	2.68	3.05	2.13
ATP13A5-AS1	2.68	3.15	2.46
OTTHUMG00000020564	2.68	3.66	2.00
SNORA11C	2.68	3.85	1.64
OTTHUMG00000167254	2.68	3.22	2.42
STAP1	2.68	3.10	2.11
MYO16-AS2	2.68	2.95	2.04
LOC100130976	2.68	2.97	2.21
LOC644919	2.68	3.12	2.40
LIPK	2.68	3.29	2.25
OTTHUMG00000172762	2.68	3.04	2.29
OIP5	2.68	3.35	2.24
FAM217A	2.68	3.08	2.27
FSCB	2.68	2.95	2.28
MIR4711	2.68	3.09	2.21
OTTHUMG00000164044	2.67	3.27	2.35
C17orf112	2.67	3.46	1.97
CRISP1	2.67	3.06	2.39
OR13C8	2.67	3.08	2.26
GK2	2.67	3.02	2.35
OTTHUMG00000164558	2.67	3.08	2.27
BCHE	2.67	2.98	2.23
ABCC6P2	2.67	3.15	2.29
CPLX4	2.67	3.36	2.19
ADGB	2.67	3.15	2.37
RS1	2.67	3.08	2.30
OR8I2	2.67	3.31	2.40
TRGV1	2.67	3.25	2.06
NUP210P1	2.67	2.97	2.19
OTTHUMG00000172013	2.67	3.01	2.43
ACSM2A	2.67	3.31	2.13
OTTHUMG00000170736	2.67	2.99	2.36
OR5M11	2.67	3.07	2.36
SCGB2A1	2.67	3.50	2.07
IL5	2.67	3.11	2.16
OTTHUMG00000021246	2.67	2.84	2.26
SAMD7	2.67	3.02	2.42
LINC00332	2.67	2.93	2.35
CCDC83	2.67	3.12	2.36

IL22	2.67	3.26	2.03
OTTHUMG00000182965	2.67	3.02	2.30
POTEE	2.67	3.44	2.06
OTTHUMG00000154946	2.66	3.10	2.38
OTTHUMG00000035603	2.66	3.13	2.13
OTTHUMG00000164855	2.66	3.37	2.22
MIR489	2.66	3.42	2.30
MAGEB5	2.66	3.06	2.19
OTTHUMG00000173406	2.66	3.27	2.26
OTTHUMG00000153045	2.66	3.31	2.31
TRAJ25	2.66	3.42	2.16
CSRP3	2.66	3.33	2.20
OTTHUMG00000168237	2.66	3.37	2.09
OTTHUMG00000176103	2.66	2.95	2.39
OTTHUMG00000086701	2.66	3.33	1.84
OTTHUMG00000015508	2.66	3.28	2.39
OTTHUMG00000169664	2.66	2.92	2.24
EIF2B5-IT1	2.66	2.96	2.30
TRDN	2.66	2.98	2.43
TM4SF20	2.66	3.29	2.30
RNU2-3P	2.66	3.14	2.26
ZNF890P	2.66	3.09	2.07
LOC400706	2.66	3.50	2.06
OTTHUMG00000019546	2.66	3.82	2.26
LINC00919	2.66	3.10	2.15
MIR1276	2.66	3.62	2.21
RAD21L1	2.66	3.12	2.34
OTTHUMG00000169352	2.66	3.23	2.27
LSP1P3	2.66	3.26	2.03
PKD2L2	2.66	2.99	2.44
OTTHUMG00000162038	2.66	3.28	2.38
ACTRT1	2.66	3.18	2.18
OTTHUMG00000153574	2.66	3.70	1.81
SNORD1B	2.66	3.20	1.77
SAGE1	2.65	2.88	2.43
OTTHUMG00000164254	2.65	3.20	2.13
FBXL21	2.65	3.06	2.29
OTTHUMG00000015069	2.65	3.38	2.21
ZNF835	2.65	2.87	2.46
CCDC160	2.65	3.04	2.20
HTR2C	2.65	3.01	2.47
OTTHUMG00000059445	2.65	2.96	2.36
OTTHUMG00000171815	2.65	3.26	1.86
NME8	2.65	3.09	2.28
OTTHUMG00000037511	2.65	3.19	2.16
MIR3074	2.65	3.14	2.19
MIR517B	2.65	3.15	2.28
OTTHUMG00000157006	2.65	3.08	2.23
LOC643037	2.65	3.07	2.17

OTTHUMG00000172532	2.65	4.21	2.03
OTTHUMG00000178983	2.65	3.33	2.33
OTTHUMG00000162504	2.65	3.20	2.15
JAKMIP2	2.65	3.05	2.37
OTTHUMG00000171854	2.65	3.01	2.25
CRTAM	2.65	3.06	2.21
MYH4	2.65	3.58	1.63
TAS2R42	2.65	3.20	2.29
LINC00706	2.65	3.03	2.25
LINC00283	2.65	2.89	2.42
OTTHUMG00000168810	2.65	3.38	2.21
TMEM78	2.65	3.12	2.21
LOC494141	2.65	3.20	2.17
HUS1B	2.65	3.00	2.26
OTTHUMG00000170475	2.65	2.90	2.46
ANKRD22	2.65	3.03	2.22
LOC100507306	2.65	3.22	1.96
RNA5SP78	2.65	3.15	2.06
SNORD12C	2.65	3.63	1.89
PROX1-AS1	2.65	3.08	2.21
OTTHUMG00000170567	2.65	3.28	2.16
KRTAP4-9	2.64	3.06	2.18
OTTHUMG00000171356	2.64	3.05	2.31
C9orf92	2.64	3.21	2.34
OTTHUMG00000160730	2.64	3.16	2.32
OFCC1	2.64	3.09	2.29
LHX5	2.64	3.15	2.26
OTTHUMG00000162756	2.64	3.41	1.96
OTTHUMG00000165099	2.64	3.45	2.02
TMCO5B	2.64	2.94	2.27
OTTHUMG00000010107	2.64	3.01	2.27
IL31	2.64	3.08	2.27
LINC00609	2.64	3.03	2.18
LOC574538	2.64	3.08	2.22
OTTHUMG00000018730	2.64	3.26	2.28
MIR496	2.64	2.94	2.26
DSTNP4	2.64	3.60	2.05
OTTHUMG00000036254	2.63	3.25	2.25
SRD5A1P1	2.63	3.36	2.01
TRIM64C	2.63	2.97	2.20
ZNF479	2.63	3.77	1.99
ZSCAN5B	2.63	3.04	2.22
OTTHUMG00000155882	2.63	2.91	2.22
FLJ44874	2.63	2.89	2.00
RNA5SP44	2.63	2.95	2.18
HSP90AA4P	2.63	2.94	2.21
LCN9	2.63	3.08	2.30
XIRP2-AS1	2.63	3.03	2.41
CDH23-AS1	2.63	2.93	2.30

OTTHUMG00000153366	2.63	2.92	2.15
MIR518D	2.63	3.54	1.92
OTTHUMG00000156611	2.63	3.39	2.43
DPPA2	2.63	3.35	2.09
OTTHUMG00000162296	2.63	3.14	2.25
TPH2	2.63	3.21	2.12
LOC554207	2.63	2.92	2.41
SNORD116-23	2.63	3.23	1.67
OTTHUMG00000161659	2.63	3.04	2.35
SYCE1L	2.63	3.20	1.95
RNA5SP327	2.63	3.18	2.01
SPINK1	2.63	3.34	2.17
C6orf7	2.63	3.51	1.90
GALR1	2.63	2.85	2.25
MIR3682	2.63	3.32	2.24
OTTHUMG00000163214	2.63	3.32	2.19
MIR4782	2.63	3.20	2.03
GUCA1C	2.63	3.17	2.33
USP27X-AS1	2.63	3.11	2.25
OTTHUMG00000015661	2.63	2.93	2.16
SNORD114-10	2.63	4.01	1.67
LOC100506865	2.63	2.89	2.36
OTTHUMG00000153648	2.63	3.51	2.03
LGSN	2.63	3.11	2.30
SNORD90	2.63	3.38	1.91
OTTHUMG00000180046	2.63	3.25	2.07
RNU5B-3P	2.62	3.31	2.03
TMEM225	2.62	3.13	2.34
OTTHUMG00000175451	2.62	3.52	1.90
OTTHUMG00000171823	2.62	2.88	2.37
ANKRD30B	2.62	3.42	2.05
ARPP21-AS1	2.62	3.85	2.13
C2orf80	2.62	3.05	2.41
LINC00547	2.62	3.59	2.26
OR11H6	2.62	3.19	2.29
OTTHUMG00000040503	2.62	3.19	2.20
OTTHUMG00000022365	2.62	3.12	2.16
OTTHUMG00000172484	2.62	3.31	2.22
NLGN1-AS1	2.62	3.25	2.16
SULT1B1	2.62	3.68	2.07
OTTHUMG00000055852	2.62	3.24	2.20
STMND1	2.62	2.96	2.39
OTTHUMG00000158576	2.62	3.36	1.98
VAV3-AS1	2.62	2.93	2.09
MIR4670	2.62	3.31	2.15
CT49	2.62	3.19	2.09
T1560	2.62	3.12	2.21
OTTHUMG00000176917	2.62	3.36	1.60
OTTHUMG00000170437	2.62	3.27	2.13

OTTHUMG00000160044	2.62	3.01	2.33
TRBV7-4	2.62	3.37	1.86
CCL8	2.62	3.05	2.37
OTTHUMG00000000507	2.62	3.12	1.91
OTTHUMG00000165998	2.62	3.40	2.09
OTTHUMG00000158236	2.62	3.20	2.20
MIR3189	2.62	3.22	2.13
OTTHUMG00000182228	2.62	3.16	2.31
LRRIQ1	2.62	3.19	2.08
DSCAM-AS1	2.61	2.92	2.42
NAALADL2-AS2	2.61	3.32	2.12
ZNF233	2.61	2.96	2.26
OR13C3	2.61	3.15	2.16
GSTA3	2.61	3.31	2.07
OTTHUMG00000020098	2.61	3.41	2.30
BRDT	2.61	3.16	2.24
OTTHUMG00000179137	2.61	3.00	2.32
METTL21C	2.61	2.97	2.42
CCL11	2.61	3.40	1.98
OR6C65	2.61	3.46	1.86
OTTHUMG00000162674	2.61	3.00	2.15
ARL14EPL	2.61	3.10	2.23
OTTHUMG00000019514	2.61	3.25	2.08
OTTHUMG00000165130	2.61	3.28	2.23
MIR183	2.61	3.41	2.05
LCE2C	2.61	3.60	1.79
OTTHUMG00000160363	2.61	3.27	1.99
OR5T2	2.61	3.20	1.89
MLIP-IT1	2.61	2.88	2.14
ZNF679	2.61	3.06	2.22
ACTRT2	2.61	2.94	2.16
HCAR2	2.61	3.21	1.55
OR4L1	2.61	3.01	2.22
OTTHUMG00000130102	2.61	3.22	2.19
SNORD116-1	2.61	3.89	1.81
OTTHUMG00000017052	2.61	2.93	2.03
OTTHUMG00000162808	2.61	3.03	2.25
OTTHUMG00000162901	2.61	3.37	2.16
TTC9B	2.61	2.96	2.17
OTTHUMG00000065034	2.61	3.01	2.35
MIR154	2.61	3.66	1.79
SLCO1B1	2.61	2.95	2.20
PCDH9-AS1	2.60	3.57	2.12
RNA5SP234	2.60	3.29	1.65
OTTHUMG00000162122	2.60	3.17	2.19
OTTHUMG00000151713	2.60	3.19	2.28
MIR320D2	2.60	3.15	1.89
OTTHUMG00000151369	2.60	2.95	2.31
LOC100132078	2.60	2.99	2.30

LOC101060277	2.60	2.95	2.21
XKRY	2.60	3.29	2.02
KLRF1	2.60	3.13	2.04
OTTHUMG00000163168	2.60	3.16	2.24
PPEF1-AS1	2.60	3.11	2.13
OR5AP2	2.60	3.21	2.20
POU6F2-AS2	2.60	3.01	2.23
GPR158	2.60	3.17	2.31
ANXA3	2.60	3.26	2.31
OTTHUMG00000163475	2.60	3.10	2.12
OTTHUMG00000158359	2.60	3.22	2.24
USP26	2.60	2.97	2.30
RNA5SP102	2.60	3.46	2.06
HEPACAM2	2.60	3.16	2.11
TRIM64	2.60	2.74	2.40
OTTHUMG00000132738	2.60	3.43	2.17
KRT12	2.60	3.51	2.04
FSTL5	2.60	3.05	2.29
OTTHUMG00000133747	2.60	3.39	2.12
OTTHUMG00000158224	2.60	2.86	2.29
OTTHUMG00000161217	2.60	3.16	2.13
OTTHUMG00000169920	2.60	2.89	2.28
PLCZ1	2.59	2.88	2.38
OTTHUMG00000164912	2.59	3.55	2.04
TMEM27	2.59	3.28	1.79
OTTHUMG00000150106	2.59	3.17	2.34
CA7	2.59	3.01	2.26
OR4N5	2.59	3.13	1.97
OTTHUMG00000015989	2.59	3.09	2.13
OTTHUMG00000021529	2.59	3.14	1.97
LOC284263	2.59	3.04	2.30
OTTHUMG00000169288	2.59	3.25	1.87
OTTHUMG00000178568	2.59	2.85	2.21
OPA1-AS1	2.59	3.36	1.91
OTTHUMG00000175783	2.59	2.87	2.41
OTTHUMG00000160917	2.59	2.89	2.18
ATXN3L	2.59	3.13	2.09
C9orf57	2.59	2.85	2.17
RNU6-60	2.59	3.49	1.41
LINC00353	2.59	3.38	1.95
LINC00462	2.59	3.13	2.09
MIR4309	2.59	3.49	1.51
GPR87	2.59	3.42	2.24
OTTHUMG00000153708	2.59	2.98	2.29
LOC728040	2.59	3.20	2.05
MIR3188	2.59	3.34	1.83
MIR4265	2.59	3.05	2.02
OR2A14	2.59	3.25	2.17
H3F3C	2.58	3.76	2.20

OTTHUMG00000164436	2.58	2.89	2.08
MIR485	2.58	2.93	2.27
OTTHUMG00000171680	2.58	3.31	2.12
ENO1-AS1	2.58	3.31	2.31
UGT2B4	2.58	3.20	2.03
OTTHUMG00000035627	2.58	3.20	2.14
IQCF6	2.58	2.89	2.24
PPBPP2	2.58	2.82	2.22
ESCO2	2.58	3.01	2.42
CATSPERB	2.58	2.89	2.10
CLCA1	2.58	2.86	2.36
LOC100506526	2.58	2.82	2.29
OTTHUMG00000169673	2.58	3.23	2.18
OTTHUMG00000153079	2.58	3.05	2.10
OTTHUMG00000151816	2.58	3.15	2.31
OTTHUMG00000157253	2.58	3.26	2.04
KRTAP24-1	2.58	3.07	2.40
OTTHUMG00000164046	2.58	3.01	2.13
IGLV5-48	2.58	3.54	1.69
GABRA1	2.58	2.99	2.31
SPATA22	2.58	3.10	2.07
LOC100505862	2.57	3.15	2.21
TRIM49	2.57	2.87	2.18
OTTHUMG00000016533	2.57	2.91	2.39
OTTHUMG00000162603	2.57	3.36	2.20
AADAC	2.57	3.15	2.07
OR10T2	2.57	3.44	2.22
OTTHUMG00000169687	2.57	2.85	2.37
OTTHUMG00000168912	2.57	2.98	2.23
LINC00305	2.57	2.89	2.18
MIR519A2	2.57	3.23	2.17
MIR135A1	2.57	3.02	2.15
OTTHUMG00000151487	2.57	3.23	2.24
TRAJ12	2.57	3.80	2.07
LINC00470	2.57	3.17	2.00
KRTAP13-4	2.57	2.97	2.22
FLJ45872	2.57	2.88	1.90
MIR4727	2.57	2.99	2.08
OTTHUMG00000154555	2.57	2.94	2.28
CTSL3P	2.57	3.11	1.83
MIR873	2.57	3.28	2.03
OTTHUMG00000165952	2.57	2.89	2.21
OTTHUMG00000048149	2.57	3.47	2.11
MIR4438	2.57	3.32	1.66
OR4D1	2.56	2.90	2.20
MROH9	2.56	3.38	2.15
MIR3166	2.56	2.86	2.24
GCSH	2.56	3.34	1.97
SNORA58	2.56	3.64	1.71

OTTHUMG00000015930	2.56	2.84	2.01
RNA5SP421	2.56	3.04	1.87
LHFPL3	2.56	2.89	2.28
OTTHUMG00000032767	2.56	3.19	2.30
LINC00113	2.56	3.06	2.08
OR2F1	2.56	3.15	2.23
CCDC148	2.56	2.80	2.42
MIR3976	2.56	3.58	1.83
OTTHUMG00000162602	2.56	3.12	2.21
FRMD6-AS2	2.56	2.94	2.25
OTTHUMG00000163633	2.56	2.88	2.21
OTTHUMG00000170971	2.56	3.18	1.92
MIR376A2	2.56	3.76	1.52
UBE2E1-AS1	2.56	3.13	2.07
OTTHUMG00000007633	2.56	3.31	2.32
LOC100130815	2.56	3.08	2.01
ANKRD45	2.56	2.99	2.19
LOC100505474	2.56	3.06	2.15
COX7B2	2.56	3.07	2.11
OTTHUMG00000172008	2.55	3.08	2.13
IGHV3-35	2.55	3.94	1.83
SHCBP1L	2.55	2.84	2.35
FABP7	2.55	2.90	2.24
OTTHUMG00000156952	2.55	2.99	2.20
LINC00379	2.55	3.38	2.07
CEACAM6	2.55	3.24	2.17
OTTHUMG00000171560	2.55	2.94	2.19
TOPAZ1	2.55	3.01	2.24
LOC339468	2.55	3.01	2.12
OTTHUMG00000161297	2.55	2.89	2.14
MEIG1	2.55	2.91	2.36
HTR2C-IT1	2.55	3.38	2.20
LOC283922	2.55	2.96	2.08
OR11G2	2.55	3.21	2.21
OTTHUMG00000154142	2.55	2.75	2.32
OTTHUMG00000164211	2.55	3.31	2.15
LOC255177	2.55	3.08	2.29
OTTHUMG00000158064	2.55	2.88	2.32
LOC100133050	2.55	3.13	2.06
MMP7	2.55	3.18	2.30
OTX2-AS1	2.55	3.18	2.16
OTTHUMG00000169929	2.54	3.14	2.12
OTTHUMG00000164332	2.54	3.06	2.09
CTAGE7P	2.54	2.86	2.10
LINC00266-4P	2.54	3.11	2.09
OTTHUMG00000167315	2.54	3.07	2.20
KRT6C	2.54	3.25	1.99
KRTAP19-8	2.54	2.93	2.25
RGS13	2.54	3.05	2.13

LOC100996635	2.54	2.92	2.24
MIR1185-1	2.54	3.92	2.01
PPP1R3A	2.54	2.82	2.14
TRBJ2-4	2.54	3.52	1.73
MIR4718	2.54	3.22	1.61
MIR15B	2.54	3.04	2.02
OTTHUMG00000160369	2.54	3.04	2.16
TRAV16	2.54	2.92	2.10
PMCH	2.54	3.10	2.00
RERGL	2.54	2.94	2.14
CNTN4-AS2	2.54	3.14	2.25
OTTHUMG00000151939	2.54	3.10	2.10
OTTHUMG00000074551	2.54	3.02	2.25
RNA5SP95	2.54	3.29	1.50
KRTAP3-3	2.54	3.27	1.89
OTTHUMG00000169275	2.54	2.83	2.22
MIR1253	2.54	3.27	1.81
VN1R4	2.54	3.26	1.94
OTTHUMG00000017954	2.53	2.94	2.25
MIR34B	2.53	3.04	1.92
HSD3B1	2.53	3.91	1.88
TRAJ61	2.53	3.27	2.13
OTTHUMG00000015404	2.53	2.77	2.30
MIR487A	2.53	3.09	2.17
TRIM60	2.53	2.92	2.28
OTTHUMG00000169781	2.53	2.95	2.08
RNA5SP355	2.53	3.43	1.97
IFNA6	2.53	3.03	2.11
C11orf88	2.53	3.04	2.04
SULT1E1	2.53	2.82	2.34
OTTHUMG00000015084	2.53	2.95	2.06
OTTHUMG00000163717	2.53	3.08	2.20
LOC100996694	2.53	3.19	1.97
OTTHUMG00000160297	2.53	3.05	2.12
LOC100652856	2.53	2.96	2.21
DEFB114	2.53	3.13	1.99
GATM-AS1	2.53	2.96	2.09
CLYBL-AS1	2.53	2.91	2.21
CXADR	2.53	3.05	1.99
MGAT4C	2.53	3.15	2.18
DACH2	2.53	2.73	2.23
OTTHUMG00000066612	2.53	2.91	2.24
SNORA2A	2.53	3.49	2.00
OTTHUMG00000020400	2.53	2.94	2.26
OTTHUMG00000170606	2.53	3.92	2.00
CD40LG	2.53	3.18	2.06
TEX21P	2.53	2.92	2.25
LAIR2	2.53	3.35	2.07
OTTHUMG00000166062	2.53	3.00	1.98

LOC100506937	2.52	2.97	2.27
MBOAT4	2.52	3.10	2.27
CLDN10-AS1	2.52	2.94	2.17
TRPC3	2.52	2.97	2.28
SNORD116-26	2.52	2.95	1.82
MEIOB	2.52	2.75	2.23
OTTHUMG00000161925	2.52	2.87	2.22
OTTHUMG00000163029	2.52	2.85	2.28
LDHC	2.52	3.18	2.02
OTTHUMG00000163118	2.52	3.04	2.11
OR51Q1	2.52	2.94	2.11
D21S2090E	2.52	2.92	2.06
OTTHUMG00000170809	2.52	2.86	2.10
OTTHUMG00000169331	2.52	2.93	2.16
OR51C1P	2.52	3.49	2.20
PCDHB17	2.52	3.20	1.92
RNF17	2.52	2.92	2.16
C6ORF50	2.52	3.05	2.21
TRIM36-IT1	2.52	3.15	2.00
OTTHUMG00000153722	2.52	3.13	2.29
ZNF763	2.52	3.23	1.93
OTTHUMG00000183863	2.52	2.94	2.28
OTTHUMG00000154306	2.52	2.80	2.20
HERC2P10	2.52	3.26	2.18
GLRA2	2.52	3.12	2.05
OTTHUMG00000170356	2.52	2.90	2.12
OTTHUMG00000022543	2.52	3.31	1.82
C1orf146	2.51	2.84	2.26
OTTHUMG00000169347	2.51	3.12	2.05
OTTHUMG00000133689	2.51	3.02	2.21
OTTHUMG00000154379	2.51	3.00	2.24
GOLGA8T	2.51	3.04	1.91
OTTHUMG00000035544	2.51	3.02	2.22
OTTHUMG00000016461	2.51	2.90	2.17
OTTHUMG00000035527	2.51	2.83	2.07
RNU5F-2P	2.51	3.24	1.89
OTTHUMG00000168333	2.51	3.22	2.04
DSCR4-IT1	2.51	3.23	1.84
NANOG	2.51	3.04	1.44
OTTHUMG00000170436	2.51	2.77	2.14
IZUMO3	2.51	3.05	2.14
FBXO43	2.51	2.80	2.04
LOC283585	2.51	2.72	2.16
MIR3945	2.51	3.42	2.12
AKIRIN2-AS1	2.51	2.88	2.23
MIR4433	2.51	3.97	1.69
OTTHUMG00000009658	2.51	3.08	2.20
OTTHUMG00000155439	2.50	3.16	2.21
TKTL2	2.50	3.14	1.98

MIR302C	2.50	3.08	1.90
LOC728739	2.50	2.89	2.16
OTTHUMG00000015064	2.50	2.83	1.99
OTTHUMG00000153097	2.50	2.99	2.00
OTTHUMG00000161298	2.50	3.16	2.26
MTUS2-AS2	2.50	2.84	2.11
OTTHUMG00000167709	2.50	3.24	2.12
OR9G4	2.50	3.05	2.10
OTTHUMG00000164494	2.50	3.12	2.06
OTTHUMG00000169739	2.50	2.88	1.96
LRRC63	2.50	2.91	2.00
LINC00890	2.50	2.71	2.23
OTTHUMG00000161115	2.50	3.09	2.18
RPL31P11	2.50	2.80	2.26
MIR549	2.50	3.07	2.10
AFP	2.50	2.97	2.23
UBXN7-AS1	2.50	2.88	2.29
MS4A6E	2.50	2.81	2.20
MIR323A	2.50	3.60	1.77
OTTHUMG00000014347	2.50	3.22	2.07
LINC00378	2.50	2.84	2.15
RNA5SP296	2.50	3.38	1.92
KLRC1	2.50	3.04	2.08
AFF2-IT1	2.50	2.91	2.09
ASIC5	2.50	3.06	2.17
MIR1284	2.49	3.19	1.90
OTTHUMG00000172114	2.49	2.89	2.07
FLJ25363	2.49	3.30	2.15
C3orf30	2.49	2.61	2.32
USP44	2.49	2.82	2.21
LRRC9	2.49	2.69	2.26
POF1B	2.49	3.00	2.13
APOOP5	2.49	3.23	2.15
MIR4503	2.49	3.65	1.86
LINC00911	2.49	2.89	2.03
APOL5	2.49	3.06	1.96
ZNF849P	2.49	2.86	1.93
SH2D1B	2.49	2.80	1.89
OTTHUMG00000168958	2.49	2.80	2.19
EQTN	2.49	3.16	2.12
LOC100506869	2.49	2.80	2.12
HPYR1	2.49	2.94	2.17
MIR1272	2.49	2.83	2.14
OTTHUMG00000165498	2.49	2.95	1.92
RNA5SP67	2.49	3.85	1.90
RNA5SP372	2.49	3.68	1.78
OTTHUMG00000018329	2.49	2.92	2.01
LOC100289656	2.49	3.05	2.18
OTTHUMG00000153710	2.48	2.87	2.05

OTTHUMG00000169661	2.48	2.79	2.10
OTTHUMG00000154143	2.48	2.94	1.91
SETP20	2.48	3.15	2.13
LOC152586	2.48	2.80	2.07
OTTHUMG00000016051	2.48	4.18	2.04
SH3GL2	2.48	2.81	2.04
OTTHUMG00000161181	2.48	2.84	2.28
MIR520D	2.48	3.26	2.01
SPINK8	2.48	2.81	2.19
OTTHUMG00000171784	2.48	3.07	2.19
FBXW10	2.48	2.95	2.06
OTTHUMG00000018629	2.48	2.81	2.12
LOC441666	2.48	3.36	2.07
OTTHUMG00000009842	2.48	2.70	2.13
OTTHUMG00000016555	2.48	3.15	2.08
HIST1H3G	2.48	3.97	1.84
ADH6	2.48	2.95	2.14
SNORD114-9	2.48	3.54	1.82
ARL2BPP2	2.48	2.96	2.04
OTTHUMG00000152909	2.48	2.90	2.31
LINC00189	2.47	2.92	2.12
OR6X1	2.47	2.99	2.23
OTTHUMG00000171116	2.47	2.82	1.97
RNA5SP407	2.47	3.22	1.49
OTTHUMG00000169480	2.47	2.79	2.09
LOC100287632	2.47	3.07	2.24
LOC286094	2.47	2.76	2.26
OTTHUMG00000170667	2.47	3.49	1.94
OTTHUMG00000031892	2.47	2.83	2.15
SYCE3	2.47	2.89	2.25
OTTHUMG00000163543	2.47	3.14	2.00
OTTHUMG00000161247	2.47	2.82	2.17
OTTHUMG00000008126	2.47	3.14	1.82
OTTHUMG00000039910	2.47	2.70	2.12
OTTHUMG00000172356	2.47	3.00	2.09
LOC441493	2.47	3.11	2.00
OR52K1	2.47	3.13	1.77
IFNA13	2.47	3.27	1.76
DNM3-IT1	2.47	2.82	2.13
RNF133	2.47	3.28	2.02
ARID4B-IT1	2.47	3.00	1.90
NAT2	2.47	3.09	1.77
LOC100507559	2.47	2.80	2.11
LOC646168	2.46	3.08	2.15
DTHD1	2.46	2.85	2.08
MIR525	2.46	3.09	1.91
ODAM	2.46	2.84	2.17
KLHL6-AS1	2.46	3.05	1.93
OTTHUMG00000171463	2.46	2.92	1.98

ARL14	2.46	2.77	2.05
OTTHUMG00000158345	2.46	3.22	2.13
RNA5SP489	2.46	3.88	1.82
OTTHUMG00000041157	2.46	3.02	2.07
SPRR2B	2.46	3.03	1.84
OTTHUMG00000164348	2.46	2.84	2.01
OTTHUMG00000010911	2.46	2.96	1.94
ZNF724P	2.46	2.98	1.81
OTTHUMG00000154381	2.46	2.68	2.12
OTTHUMG00000154865	2.46	3.59	1.86
OTTHUMG00000166372	2.46	2.84	2.13
OTTHUMG00000180457	2.46	3.51	1.88
OTTHUMG00000162373	2.46	2.86	2.14
RNA5SP177	2.46	3.71	1.87
MIR548AG2	2.46	3.15	1.62
THEMIS	2.45	2.73	2.18
LINC00326	2.45	2.66	2.15
MIR652	2.45	2.84	1.97
OTTHUMG00000041308	2.45	3.21	1.96
MIR4527	2.45	3.20	1.84
RNU5A-5P	2.45	3.28	1.54
ADAM2	2.45	2.80	2.22
PDCL2	2.45	2.78	2.13
RNA5SP231	2.45	2.97	2.20
OTTHUMG00000171822	2.45	2.80	1.96
OTTHUMG00000163520	2.45	2.86	2.18
OTTHUMG00000169348	2.45	2.81	2.13
SPANXC	2.45	4.41	1.55
HSFY1P1	2.45	3.16	1.88
LCE3E	2.45	3.10	2.10
RNA5SP438	2.45	3.47	1.81
TINAG	2.45	2.66	2.20
RNA5SP81	2.45	3.11	1.83
CD69	2.45	2.92	2.12
OTTHUMG00000161304	2.45	2.85	2.06
OTTHUMG00000021163	2.45	2.73	2.21
NDST4	2.45	2.78	1.96
LINC00605	2.45	2.74	2.06
PIK3C2G	2.45	2.88	2.13
OTTHUMG00000161291	2.45	2.83	2.16
UBTFL1	2.45	2.85	1.72
NRG1-IT2	2.45	2.90	1.98
OTTHUMG00000161883	2.45	2.63	2.25
OTTHUMG00000163813	2.44	2.94	1.88
GMCL1P1	2.44	3.02	1.69
ZNF705G	2.44	3.02	1.97
OTTHUMG00000014342	2.44	2.99	2.02
ACTR3BP2	2.44	3.08	2.11
MIR182	2.44	2.96	2.11

PAGE3	2.44	2.97	2.23
FCRL5	2.44	2.79	1.71
FAM5C	2.44	2.70	2.16
PLS1-AS1	2.44	2.91	2.03
CRH	2.44	2.66	2.05
GRM7-AS3	2.44	2.86	2.19
OTTHUMG00000159088	2.44	2.83	2.15
CACNA1C-IT2	2.44	2.87	2.05
OTTHUMG00000153759	2.44	2.70	2.16
OTTHUMG00000171051	2.43	2.77	2.16
VIP	2.43	2.70	2.25
CCDC148-AS1	2.43	2.98	2.10
OTTHUMG00000155418	2.43	2.81	2.07
OTTHUMG00000015254	2.43	2.67	2.23
OTTHUMG00000160611	2.43	3.07	2.04
OTTHUMG00000015949	2.43	2.77	2.04
F13B	2.43	2.80	2.06
DEFB129	2.43	2.92	1.91
HBD	2.43	3.19	2.04
OTTHUMG00000168900	2.43	2.94	1.96
OTTHUMG00000171069	2.43	2.64	2.08
MIR3115	2.43	2.92	1.99
MIR526A2	2.43	3.07	2.10
HIST3H3	2.43	2.77	2.06
OTTHUMG00000170011	2.43	2.67	2.01
MIR380	2.43	3.30	1.83
MIR591	2.42	2.64	2.24
NCKAP5-IT1	2.42	2.87	1.81
SPINT3	2.42	3.10	2.14
OTTHUMG00000017237	2.42	2.77	2.03
OTTHUMG00000161691	2.42	3.04	1.95
CCDC54	2.42	2.81	2.08
SCGB2A2	2.42	3.02	1.93
LOC100144595	2.42	2.97	2.05
OTTHUMG00000151752	2.42	2.93	1.95
LINC00345	2.42	2.94	1.97
OTTHUMG00000164035	2.42	3.59	1.53
OTTHUMG00000153912	2.42	2.97	1.87
FAM66E	2.42	3.47	1.68
ZNF705A	2.42	2.83	2.08
OTTHUMG00000160075	2.42	2.71	2.19
BCL2L10	2.42	2.72	1.74
OTTHUMG00000032037	2.42	3.07	2.09
OTTHUMG00000161384	2.42	3.08	2.00
GSTA2	2.42	3.41	1.65
MIR4752	2.42	2.69	2.06
SCG3	2.42	2.97	2.04
OTTHUMG00000015055	2.42	2.80	1.73
SPATA31D3	2.42	3.14	1.95

OTTHUMG00000164665	2.42	2.71	2.20
MIR142	2.42	3.13	1.76
MIR4666A	2.42	2.99	1.93
FLJ31958	2.42	3.15	1.97
C5orf48	2.42	2.74	1.96
KRTAP4-5	2.42	2.86	2.09
LOC646999	2.41	3.05	1.60
OTTHUMG00000153414	2.41	2.82	2.23
FBXO47	2.41	3.15	2.16
OTTHUMG00000162625	2.41	3.01	2.09
OTTHUMG00000156616	2.41	2.81	2.14
LOC100288079	2.41	2.88	2.13
OR4K15	2.41	2.81	1.75
RNA5SP183	2.41	3.42	1.75
RNA5SP476	2.41	3.39	1.77
OTTHUMG00000164655	2.41	2.73	1.85
ZBBX	2.41	2.69	2.03
TTC29	2.41	2.59	1.97
OTTHUMG00000021281	2.41	2.74	2.18
OTTHUMG00000171620	2.41	2.93	1.75
MIR3649	2.41	3.51	1.62
MIR498	2.41	2.72	1.77
OTTHUMG00000171911	2.41	2.82	2.01
C12orf50	2.41	2.97	2.15
OTTHUMG00000162768	2.41	2.96	1.93
GYPB	2.41	2.72	2.13
RNU7-60P	2.40	3.60	1.76
OR5M9	2.40	3.03	2.06
OTTHUMG00000160432	2.40	2.76	1.95
PAGE4	2.40	2.61	2.24
OR52N2	2.40	2.73	2.18
OTTHUMG00000158284	2.40	2.80	2.10
OTTHUMG00000132778	2.40	2.74	2.10
LOC100130298	2.40	2.79	2.10
OTTHUMG00000164307	2.40	3.58	1.89
ZSCAN4	2.40	2.70	2.06
C21orf37	2.40	2.63	2.12
MIR520B	2.40	3.26	1.79
SNORA30	2.40	2.70	2.04
DSG1	2.40	2.88	2.00
LOC283299	2.40	2.91	2.03
OR7D1P	2.40	3.02	1.92
MT1B	2.40	2.78	2.07
OTTHUMG00000150928	2.40	2.66	2.11
MIR429	2.40	3.02	2.10
DSG3	2.40	2.72	2.06
OTTHUMG00000172112	2.40	2.98	2.08
FTMT	2.39	2.67	1.90
LOC729307	2.39	3.09	2.00

OR51A7	2.39	3.12	1.98
MIR19B2	2.39	3.20	1.92
OTTHUMG00000154028	2.39	2.98	2.22
RSU1P2	2.39	2.84	1.89
FLJ37505	2.39	2.64	2.14
OTTHUMG00000166985	2.39	3.24	1.85
MIR578	2.39	3.11	2.03
DEFB133	2.39	2.68	1.73
OTTHUMG00000160367	2.39	2.79	2.17
TRAV24	2.39	2.72	2.07
RBMY2EP	2.39	3.62	1.84
MIR4280	2.39	4.14	1.75
MIR4261	2.39	2.98	1.82
LOC100507477	2.39	2.89	2.00
OTTHUMG00000153580	2.39	2.66	2.23
CSN1S2AP	2.39	3.23	2.07
LINC00682	2.39	2.66	2.10
OTTHUMG00000035971	2.39	2.81	2.02
ANKRD62	2.39	2.89	2.13
OTTHUMG00000160879	2.39	2.88	1.89
RGS7	2.38	2.62	2.00
LOC645355	2.38	2.70	2.13
OTTHUMG00000078327	2.38	2.93	1.94
OTTHUMG00000169394	2.38	2.90	2.06
DYDC1	2.38	2.85	1.93
OR4D5	2.38	3.08	1.93
OTTHUMG00000164124	2.38	3.30	1.94
XKR3	2.38	2.84	2.04
LOC152578	2.38	2.85	2.03
TRAPPC3L	2.38	2.95	1.74
TAAR1	2.38	2.97	1.94
RNASE10	2.38	2.82	1.94
OTTHUMG00000160229	2.38	3.35	1.91
LRRD1	2.38	2.67	2.11
RNA5SP90	2.38	2.97	1.95
LOC285501	2.38	2.69	2.17
C7orf69	2.38	2.77	2.04
OTTHUMG00000019729	2.38	2.67	1.75
MIR378C	2.38	3.10	1.97
OTTHUMG00000058921	2.38	2.77	1.68
RNA5SP466	2.38	3.14	1.72
OTTHUMG00000162196	2.38	2.67	2.04
OTTHUMG00000168404	2.38	2.81	2.12
OTTHUMG00000074549	2.38	2.90	2.15
C7orf72	2.38	2.83	2.10
CCDC39-AS1	2.38	3.16	1.75
CNBD1	2.37	2.68	2.12
OR4D2	2.37	3.59	1.47
SCN2A	2.37	3.19	1.73

ZNF92	2.37	3.29	1.53
OTTHUMG00000171993	2.37	2.86	2.06
OTTHUMG00000153158	2.37	3.05	2.11
OTTHUMG00000170687	2.37	2.83	1.93
OTTHUMG00000161469	2.37	2.61	2.15
EBLN1	2.37	3.36	1.83
MIR4664	2.37	2.88	2.01
RFPL4A	2.37	3.11	1.96
MIR4764	2.37	3.13	1.88
SOSTDC1	2.37	2.69	2.01
OTTHUMG00000151001	2.37	2.69	2.13
SNORD12B	2.37	2.95	1.84
OTTHUMG00000010912	2.37	2.68	1.81
OR4K5	2.37	3.36	1.92
OTTHUMG00000154108	2.37	2.98	1.95
LINC00587	2.37	2.86	1.86
RGS18	2.37	2.80	2.02
MIR4500	2.37	3.51	1.77
OTTHUMG00000169845	2.37	2.66	2.02
LOC100507207	2.37	2.85	2.15
LOC100130964	2.36	3.03	1.95
RNU105C	2.36	2.75	2.09
OTTHUMG00000164414	2.36	3.22	1.98
OTTHUMG00000164546	2.36	3.20	1.76
LGALS13	2.36	2.78	2.03
OR51B5	2.36	2.99	1.99
OTTHUMG00000074607	2.36	2.81	2.09
TRAJ7	2.36	3.52	1.81
PSMA8	2.36	2.94	2.01
OTTHUMG00000170013	2.36	2.59	2.14
TECRL	2.36	3.03	1.96
RNU1-17P	2.36	2.73	2.06
HIST1H3H	2.36	3.39	1.54
MIR4703	2.36	2.84	1.46
MIR944	2.36	3.22	1.71
FUNDC2P2	2.36	3.63	1.85
OTTHUMG00000162711	2.35	2.67	2.05
GNGT1	2.35	2.77	2.10
CYP3A43	2.35	2.67	1.93
OTTHUMG00000161118	2.35	2.73	2.04
OTTHUMG00000153704	2.35	2.80	1.96
KRTAP19-3	2.35	3.03	2.04
OTTHUMG00000172835	2.35	3.68	1.70
LOC729020	2.35	2.75	2.03
MIR18B	2.35	2.84	1.82
LOC100505863	2.35	2.69	2.01
MEP1A	2.35	2.68	1.95
OTTHUMG00000153111	2.35	2.63	2.08
LOC100505609	2.35	2.78	1.92

OTTHUMG00000155267	2.35	2.70	2.16
EPGN	2.34	2.70	2.06
OTTHUMG00000155012	2.34	2.61	1.82
DEFB127	2.34	2.66	1.83
MIR3142	2.34	2.87	1.79
OTTHUMG00000015823	2.34	2.90	2.05
CFHR3	2.34	3.08	1.67
KRT26	2.34	3.46	2.00
OTTHUMG00000167421	2.34	2.74	1.89
SMPX	2.34	2.85	1.76
OTTHUMG00000182829	2.34	2.81	1.73
C10orf67	2.34	2.58	2.13
ANKRD20A8P	2.34	2.81	2.08
FAM72D	2.34	2.91	1.98
LINC00323	2.34	2.75	1.82
ANKRD26P1	2.34	3.06	2.05
OTTHUMG00000153703	2.34	2.83	1.95
OTTHUMG00000150951	2.34	2.49	2.11
OTTHUMG00000162593	2.34	2.65	2.01
OTTHUMG00000161916	2.34	2.81	2.04
SERHL2	2.34	3.24	1.76
ROPN1L-AS1	2.34	3.08	2.08
MIR381	2.33	2.66	1.93
HM13-IT1	2.33	2.93	1.92
OTTHUMG00000041437	2.33	3.00	1.84
OTTHUMG00000161125	2.33	2.75	2.08
RNU7-30P	2.33	3.12	1.71
AFM	2.33	2.66	2.06
OR5M8	2.33	3.04	1.93
OTTHUMG00000171940	2.33	2.71	2.13
OTTHUMG00000180387	2.33	2.57	1.99
OTTHUMG00000161226	2.33	2.94	2.02
OTTHUMG00000163015	2.33	3.16	1.95
RNA5SP239	2.33	3.32	1.76
OTTHUMG00000078249	2.33	2.92	1.87
MIRLET7A2	2.33	3.13	1.73
LINC00254	2.33	2.82	1.92
OTTHUMG00000014830	2.33	2.84	1.93
OTTHUMG00000020080	2.33	2.86	1.70
OTTHUMG00000166206	2.33	2.97	1.93
OTTHUMG00000162873	2.33	2.84	1.99
NDFIP2-AS1	2.33	2.95	1.92
OTTHUMG00000157191	2.32	2.70	2.15
SLC7A11-AS1	2.32	2.72	2.03
MIR3616	2.32	2.92	2.02
OTTHUMG00000162608	2.32	2.90	2.05
OTTHUMG00000152616	2.32	2.64	1.99
OTTHUMG00000169737	2.32	3.07	1.75
OTTHUMG00000015068	2.32	2.75	1.86

OTTHUMG00000166399	2.32	3.00	1.49
NANOGP1	2.32	2.74	2.07
OTTHUMG00000161747	2.32	2.75	1.68
TEX11	2.32	2.70	2.08
OTTHUMG00000152143	2.31	2.84	1.93
REG1A	2.31	2.73	2.03
NBPF7	2.31	2.73	1.84
OTTHUMG00000160731	2.31	2.86	1.70
OTTHUMG00000172467	2.31	3.11	1.66
LOC100506050	2.31	3.05	2.02
OTTHUMG00000008245	2.31	2.71	1.88
OTTHUMG00000153369	2.31	2.61	1.97
OTTHUMG00000152056	2.31	2.91	1.89
NBPF22P	2.31	3.09	1.58
GKN2	2.31	2.62	1.99
LINC00871	2.31	2.63	1.99
OTTHUMG00000163006	2.31	2.55	1.86
OTTHUMG00000161649	2.31	2.49	2.07
OTTHUMG00000162998	2.31	3.08	1.82
OTTHUMG00000162351	2.31	2.74	1.92
HIST1H2BJ	2.31	2.86	2.03
OTTHUMG00000074548	2.31	2.79	2.01
TRAV23DV6	2.30	2.64	1.97
RNA5SP492	2.30	2.78	1.95
OTTHUMG00000154585	2.30	2.71	2.00
RNA5SP57	2.30	2.83	1.84
OTTHUMG00000152845	2.30	2.68	1.79
MIMT1	2.30	2.67	2.05
OR1L8	2.30	2.94	1.96
OR51I2	2.30	2.73	1.95
MIR889	2.30	3.01	1.77
EIF2S3L	2.30	3.18	1.87
TRAV35	2.30	3.26	2.00
OTTHUMG00000019544	2.30	2.52	2.11
OTTHUMG00000164292	2.30	2.77	1.78
OR5J2	2.30	2.59	1.90
ZNF645	2.30	2.55	2.01
MIR4460	2.30	2.92	1.67
YY1P2	2.30	2.75	1.83
OTTHUMG00000161799	2.30	2.80	1.85
LINC00115	2.30	3.22	1.92
OTTHUMG00000172355	2.30	2.67	2.04
OTTHUMG00000158358	2.29	2.77	1.85
LOC645434	2.29	2.55	2.00
OTTHUMG00000017937	2.29	2.93	2.01
OR5L1	2.29	2.64	1.82
DEFA10P	2.29	2.53	2.05
OTTHUMG00000155686	2.29	2.70	1.76
PROL1	2.29	2.68	1.92

RNA5SP127	2.29	2.50	1.87
OR13C9	2.29	2.87	1.95
RNY3P10	2.29	3.58	1.46
OTTHUMG00000162105	2.29	2.59	2.00
OTTHUMG00000161146	2.29	2.64	2.06
SERPINB3	2.29	2.93	1.77
KRTAP19-7	2.29	3.08	1.79
OTTHUMG00000170693	2.29	2.73	2.01
OTTHUMG00000162772	2.29	2.73	1.64
OTC	2.29	2.43	1.99
OTTHUMG00000078273	2.29	2.59	2.11
LINC00690	2.29	3.06	1.98
OTTHUMG00000164594	2.28	2.79	1.74
SCP2D1	2.28	2.68	1.97
RNA5SP174	2.28	2.82	1.85
OTTHUMG00000032760	2.28	2.65	2.05
RNU6-65	2.28	3.08	1.68
MIR4307	2.28	3.83	1.75
OTTHUMG00000015096	2.28	3.11	1.95
OTTHUMG00000164155	2.28	2.83	1.96
TAAR2	2.28	2.73	2.04
OR10H2	2.28	2.71	1.92
OTTHUMG00000162807	2.28	2.78	1.92
MIR4773-2	2.28	2.72	1.90
OTTHUMG00000163874	2.28	3.01	1.82
MIR4317	2.28	3.14	1.46
LINC00347	2.28	2.82	1.96
CELP	2.28	2.67	1.99
MIR4699	2.28	2.78	2.01
MAGI2-AS2	2.28	3.09	2.02
OTTHUMG00000151905	2.28	2.54	2.00
OTTHUMG00000041126	2.28	2.50	2.00
MIR642B	2.28	3.43	1.30
IGLJ6	2.27	2.82	1.94
OTTHUMG00000152631	2.27	2.61	1.92
ANP32C	2.27	2.91	1.73
MIR3177	2.27	2.42	2.11
OTTHUMG00000017369	2.27	2.99	1.86
OTTHUMG00000039495	2.27	2.75	1.93
OTTHUMG00000017117	2.27	2.55	2.04
OTTHUMG00000170122	2.27	2.68	1.84
LOC100996267	2.27	2.56	1.99
OTTHUMG00000017173	2.27	2.56	1.97
OTTHUMG00000170010	2.27	2.62	1.90
SCEL-AS1	2.27	3.11	1.81
EMCN-IT3	2.27	2.50	2.04
PNLIPRP3	2.27	2.65	1.98
OTTHUMG00000167628	2.27	2.97	1.60
OTTHUMG00000177911	2.27	3.08	1.77

CYP4Z2P	2.27	3.02	1.81
KRTAP4-7	2.27	3.08	1.47
CNGA1	2.27	2.54	2.03
OTTHUMG00000162995	2.27	2.64	1.83
ZNF729	2.26	2.80	1.68
DEPDC4	2.26	2.43	2.06
OTTHUMG00000162778	2.26	2.89	1.81
TAS2R43	2.26	3.24	1.48
OTTHUMG00000021149	2.26	2.68	1.81
OTTHUMG00000152853	2.26	2.53	1.91
IGLV6-57	2.26	2.86	1.71
FAM86JP	2.26	2.91	1.81
MIR4684	2.26	2.89	1.78
RNA5SP125	2.26	3.06	1.79
OTTHUMG00000160185	2.26	2.76	2.00
MIR4668	2.26	3.01	1.71
OTTHUMG00000014831	2.26	2.96	1.76
OTTHUMG00000164828	2.26	2.44	1.97
LOC100128317	2.26	2.81	1.97
IFNA16	2.26	2.76	1.92
OTTHUMG00000166370	2.25	2.64	2.01
ADAT3	2.25	2.54	1.92
OTTHUMG00000154543	2.25	2.62	1.94
LINC00395	2.25	2.75	2.02
TGIF2LY	2.25	2.80	1.79
OR4F4	2.25	3.28	1.79
HIST1H1B	2.25	2.84	1.55
RNA5SP336	2.25	2.93	1.49
OTTHUMG00000153056	2.25	2.79	1.79
CRSP8P	2.25	2.98	1.73
DNAJB3	2.25	3.09	1.62
MIR128-1	2.25	2.77	1.77
KLRC3	2.25	3.03	1.80
MIR200C	2.25	3.15	1.57
USP17L3	2.25	3.14	1.64
OTTHUMG00000008095	2.25	2.78	1.81
OTTHUMG00000152139	2.25	2.77	1.96
PLSCR5-AS1	2.25	2.89	1.89
LRRIQ4	2.25	3.07	1.95
OTTHUMG00000151402	2.25	2.67	1.90
PABPC3	2.24	3.02	1.65
UBE2U	2.24	2.56	1.99
CLDN17	2.24	2.68	1.86
MIR1278	2.24	2.71	1.86
MBD3L1	2.24	2.92	1.97
LOC100507274	2.24	2.80	2.03
OR5AR1	2.24	2.79	2.02
MIR1262	2.24	2.86	1.59
OTTHUMG00000168857	2.24	2.70	1.80

DEFA3	2.24	3.26	1.75
SLCO1B3	2.24	2.61	1.84
OTTHUMG00000009114	2.24	2.69	1.87
MIR302A	2.24	2.95	1.82
MSMB	2.24	2.90	1.98
OTTHUMG00000017336	2.24	2.82	1.69
RNA5SP481	2.24	2.95	1.92
PRB4	2.24	3.82	1.78
SERPINB4	2.24	3.00	1.77
RN7SKP5	2.24	2.94	1.75
LINC00604	2.24	2.37	2.01
MIR4714	2.24	3.02	1.83
OTTHUMG00000153051	2.23	2.80	1.76
MIR676	2.23	2.88	1.77
CCDC152	2.23	2.79	1.85
SNORD72	2.23	3.83	1.35
RNU7-88P	2.23	2.94	1.80
OSTN	2.23	2.76	1.76
MSH4	2.23	2.60	1.95
SERPINB13	2.23	2.85	1.92
SNORD105	2.23	2.79	1.67
KLRF2	2.23	2.87	1.94
OTTHUMG00000172323	2.23	2.82	1.79
OR52E8	2.23	3.24	1.70
OTTHUMG00000017094	2.23	2.84	1.75
OTTHUMG00000161780	2.23	2.61	1.94
SYCP1	2.23	2.41	1.98
RXFP1	2.23	2.66	1.96
PER4	2.23	2.57	1.90
OR52K2	2.22	2.90	1.81
FOLH1B	2.22	2.91	1.68
OR6C70	2.22	2.71	1.87
LOC100288208	2.22	2.84	1.91
IGKV1D-42	2.22	2.95	1.74
OTTHUMG00000152747	2.22	2.44	2.03
MIR410	2.22	2.55	1.95
OTTHUMG00000163317	2.22	3.42	1.73
OR1L3	2.22	2.73	1.57
RNA5SP176	2.22	3.23	1.65
OTTHUMG00000017809	2.22	2.71	1.79
OTTHUMG00000021532	2.22	2.66	1.87
TRBV7-8	2.22	2.72	1.59
CD200R1L	2.22	2.53	1.97
NRG1-IT3	2.22	2.83	1.71
OR5H2	2.22	2.70	1.80
OR4C3	2.22	2.76	1.77
SCARNA15	2.22	2.67	1.59
LOC100505946	2.22	2.72	1.65
OTTHUMG00000159060	2.22	2.73	1.88

OTTHUMG00000162604	2.22	2.55	1.96
OTTHUMG00000155315	2.22	2.61	1.84
MIR520C	2.21	2.95	1.79
OTTHUMG00000067507	2.21	2.89	1.74
PIPSL	2.21	3.19	1.23
MIR1206	2.21	3.27	1.34
KRTAP19-6	2.21	2.64	1.93
LINC00889	2.21	2.78	1.80
SI	2.21	2.63	2.01
LOC340107	2.21	2.58	1.93
RNA5SP277	2.21	2.87	1.63
MIR493	2.21	3.15	1.77
SYT14L	2.21	2.64	1.58
KRTAP22-1	2.21	2.83	1.89
MIR491	2.21	2.83	1.97
IGKV2D-26	2.21	2.58	1.66
FAM223B	2.21	2.94	1.44
OR2AK2	2.21	2.58	1.99
CCDC172	2.21	2.85	1.88
OTTHUMG00000165230	2.21	2.86	1.61
IFNA17	2.21	2.89	1.85
OTTHUMG00000163914	2.21	2.41	1.89
OTTHUMG00000153044	2.21	2.81	1.78
OTTHUMG00000169476	2.21	2.59	1.98
OTTHUMG00000159128	2.21	2.70	1.79
OTTHUMG00000176580	2.21	2.63	1.90
OR2AG2	2.21	2.52	1.86
OTTHUMG00000164839	2.21	2.59	1.90
OTTHUMG00000172481	2.21	2.72	1.79
RPL13AP3	2.21	2.64	1.90
LOC729968	2.21	3.21	1.77
LINC00599	2.21	2.93	1.83
OTTHUMG00000156067	2.20	2.61	1.74
C10orf115	2.20	2.45	1.82
CFHR4	2.20	3.09	1.72
MIR208A	2.20	2.75	1.88
OTTHUMG00000035229	2.20	2.74	1.78
HS3ST6	2.20	2.66	1.74
OTTHUMG00000009218	2.20	2.68	1.90
RNA5SP369	2.20	3.15	1.59
OTTHUMG00000168501	2.20	2.87	1.75
MIR216B	2.20	2.49	1.95
TRAJ42	2.20	2.69	1.76
ADAM3A	2.20	2.42	1.94
OTTHUMG00000022220	2.20	2.48	1.94
PPP1R42	2.20	2.66	1.98
MIR567	2.20	2.84	1.92
RN7SKP10	2.20	2.59	1.80
IGLV11-55	2.20	2.64	1.89

MIR300	2.19	3.59	1.65
OTTHUMG00000015045	2.19	2.68	1.97
OR5P2	2.19	2.64	1.85
OTTHUMG00000162457	2.19	2.67	1.89
LOC100506122	2.19	2.83	1.96
OTTHUMG00000164616	2.19	2.43	1.96
OTTHUMG00000164283	2.19	2.70	1.80
SULT6B1	2.19	2.56	1.92
OTTHUMG00000162794	2.19	2.79	1.81
OTTHUMG00000172483	2.19	2.76	1.87
MIR320B2	2.19	2.76	1.85
MIR4779	2.19	2.59	1.93
OTTHUMG00000014829	2.19	2.88	1.91
OTTHUMG00000015659	2.19	2.56	1.76
OTTHUMG00000171368	2.19	2.96	1.71
OTTHUMG00000151921	2.19	2.39	2.00
OTTHUMG00000161209	2.19	3.47	1.53
LINC00879	2.18	2.60	1.86
RNA5SP376	2.18	2.64	1.64
LOC100506489	2.18	2.46	1.86
C1orf141	2.18	2.64	1.88
OTTHUMG00000171617	2.18	2.51	1.82
OTTHUMG00000158840	2.18	2.51	1.76
OTTHUMG00000170350	2.18	2.77	1.86
RNA5SP179	2.18	2.99	1.76
OTTHUMG00000171350	2.18	2.90	1.81
RNU5B-4P	2.18	2.80	1.61
EDDM3A	2.18	2.62	1.87
OTTHUMG00000163190	2.18	2.87	1.53
OTTHUMG00000164644	2.18	2.58	1.89
RBMY3AP	2.18	3.07	1.77
OTTHUMG00000161243	2.18	2.77	1.64
RNA5SP382	2.17	3.18	1.50
MIR516B2	2.17	3.18	1.48
TRAV36DV7	2.17	3.27	1.76
OR5B3	2.17	3.18	1.55
LINC00351	2.17	2.63	1.87
RNA5SP280	2.17	2.56	1.75
OTTHUMG00000078245	2.17	2.63	1.71
RNA5SP516	2.17	2.82	1.67
CXorf58	2.17	2.56	1.86
OTTHUMG00000013087	2.17	2.51	1.69
SPANXN2	2.17	2.46	1.90
KRTAP22-2	2.17	2.81	1.82
OTTHUMG00000032470	2.17	2.84	1.84
PCDH9-AS2	2.17	2.73	1.83
OR4D6	2.16	2.75	1.65
LOC100507513	2.16	2.64	1.89
CES5AP1	2.16	2.56	1.86

MIR132	2.16	2.49	1.79
LIPI	2.16	2.53	1.92
ARHGAP31-AS1	2.16	2.45	1.90
OTTHUMG00000032680	2.16	2.68	1.70
OTTHUMG00000169921	2.16	2.43	1.89
TAAR8	2.16	2.49	1.78
OTTHUMG00000152558	2.16	2.45	1.97
CYP2C19	2.16	2.38	1.83
OTTHUMG00000010769	2.16	2.61	1.71
MIR363	2.16	2.84	1.68
OTTHUMG00000158994	2.16	2.58	1.87
MIR4770	2.15	2.95	1.57
MIR4643	2.15	3.21	1.66
OTTHUMG00000165488	2.15	2.55	1.95
MIR520A	2.15	2.68	1.62
CSF2	2.15	2.33	1.94
OTTHUMG00000177334	2.15	2.66	1.76
OTTHUMG00000150865	2.15	2.40	1.61
CST1	2.15	2.86	1.68
UGT2A1	2.15	2.78	2.00
OTTHUMG00000163671	2.15	2.78	1.80
HHLA2	2.15	2.75	1.85
OTTHUMG00000166245	2.15	2.41	1.79
OR10G4	2.15	2.85	1.60
OTTHUMG00000162592	2.15	2.74	1.66
OTTHUMG00000171640	2.15	2.97	1.53
RNA5-8SP5	2.14	2.77	1.70
OTTHUMG00000074314	2.14	2.89	1.78
OR4C11	2.14	2.50	1.74
MIA2	2.14	2.57	1.75
OTTHUMG00000043433	2.14	2.84	1.46
OTTHUMG00000018047	2.14	2.51	1.46
MIR4794	2.14	2.86	1.19
OTTHUMG00000019566	2.14	2.58	1.75
RXFP2	2.14	2.31	1.88
SNORD85	2.14	2.51	1.87
OTTHUMG00000163549	2.14	2.36	1.89
MIR320B1	2.14	2.65	1.82
OTTHUMG00000161427	2.14	2.98	1.49
LOC152225	2.14	2.33	1.91
LINC00381	2.14	2.48	1.91
OTTHUMG00000163152	2.14	2.53	1.74
MIR3943	2.14	2.62	1.77
OR4K1	2.14	3.19	1.74
HPVC1	2.13	2.58	1.77
RNU7-34P	2.13	3.48	1.31
OTTHUMG00000035526	2.13	2.56	1.94
RNA5SP287	2.13	2.98	1.72
OTTHUMG00000159498	2.13	2.63	1.75

ICOS	2.13	2.42	1.78
LINC00500	2.13	2.70	1.83
OTTHUMG00000162774	2.13	2.47	1.94
RNA5SP464	2.13	2.56	1.82
OTTHUMG00000168421	2.13	2.66	1.62
RNA5SP480	2.13	2.75	1.66
FLJ16124	2.13	2.80	1.70
DMD-AS3	2.13	2.43	1.84
OTTHUMG00000160872	2.13	2.59	1.73
OTTHUMG00000153756	2.13	2.94	1.56
OTTHUMG00000067120	2.13	2.83	1.59
OTTHUMG00000162382	2.13	2.53	1.83
SNORA36C	2.13	2.57	1.87
TTTY12	2.13	2.47	1.62
OTTHUMG00000151707	2.13	2.59	1.81
CCDC178	2.13	2.37	1.80
OR6C1	2.13	2.61	1.72
OTTHUMG00000151710	2.13	2.33	1.78
OTTHUMG00000160994	2.13	2.38	1.65
DDX53	2.13	2.73	1.79
MIR409	2.13	2.40	1.82
MIR548T	2.13	2.55	1.61
MIR548AD	2.13	3.56	1.40
IGHA1	2.13	2.91	1.58
RNA5SP267	2.12	3.24	1.40
MIR4774	2.12	2.70	1.60
MIR329-2	2.12	3.16	1.65
MIR4266	2.12	3.76	1.20
OTTHUMG00000162579	2.12	2.90	1.83
MIR4696	2.12	3.20	1.80
MIR1283-2	2.12	2.50	1.59
OTTHUMG00000014906	2.12	3.20	1.69
KRTAP9-7	2.12	2.75	1.59
OTTHUMG00000154195	2.12	2.41	1.75
OTTHUMG00000171383	2.12	2.77	1.52
MIR432	2.12	2.57	1.90
OTTHUMG00000167378	2.12	2.64	1.82
RNA5SP504	2.12	2.62	1.55
ANKRD18B	2.12	2.53	1.74
FLRT3	2.12	2.34	1.84
OR8J1	2.12	3.22	1.58
KRTAP21-3	2.12	2.77	1.78
IL23A	2.12	2.75	1.66
SPANXN3	2.12	2.53	1.88
MIR655	2.11	3.07	1.77
OTTHUMG00000151358	2.11	2.52	1.59
OTTHUMG00000020021	2.11	2.30	1.72
GRM5-AS1	2.11	2.52	1.88
OR4A16	2.11	2.83	1.71

OTTHUMG00000010017	2.11	2.34	1.81
FMO9P	2.11	2.52	1.79
S100G	2.11	2.33	1.85
OTTHUMG00000017657	2.11	2.54	1.78
SSMEM1	2.11	2.52	1.62
MIR219-1	2.11	2.39	1.81
CFHR1	2.11	2.83	1.74
OR4F21	2.11	3.09	1.39
OTTHUMG00000160395	2.10	2.42	1.85
OTTHUMG00000163750	2.10	2.35	1.72
LOC100507498	2.10	2.41	1.87
OTTHUMG00000152267	2.10	2.57	1.85
OR52H1	2.10	2.47	1.86
MIR3660	2.10	3.20	1.43
OTTHUMG00000153651	2.10	2.79	1.61
OTTHUMG00000153856	2.10	2.46	1.80
MIR133A1	2.10	2.74	1.74
OR5H14	2.10	2.48	1.79
OTTHUMG00000015791	2.10	2.43	1.70
OTTHUMG00000008243	2.10	2.32	1.72
SNORD111B	2.10	3.06	1.50
OTTHUMG00000074542	2.10	2.42	1.82
MIR372	2.09	2.44	1.79
OTTHUMG00000162339	2.09	2.77	1.73
TCEAL3-AS1	2.09	2.48	1.70
OTTHUMG00000162230	2.09	2.54	1.87
MAPT-AS1	2.09	3.12	1.69
MIR514B	2.09	2.79	1.51
OTTHUMG00000014978	2.09	2.88	1.80
FGG	2.09	2.46	1.78
TMEM257	2.09	2.42	1.94
SCGB1D1	2.09	2.54	1.69
LINC00613	2.09	2.70	1.69
PCDH11X	2.09	2.50	1.60
MIR3158-2	2.09	2.75	1.53
IFNE	2.09	2.95	1.68
GDF3	2.09	2.45	1.69
OTTHUMG00000160989	2.09	2.46	1.76
OTTHUMG00000161553	2.09	2.83	1.65
RNA5SP264	2.09	2.59	1.69
MIR499B	2.08	2.50	1.73
WTH3DI	2.08	2.60	1.53
OTTHUMG00000163009	2.08	2.68	1.71
LINC00301	2.08	2.34	1.86
LOC100506926	2.08	2.80	1.78
OTTHUMG00000166487	2.08	2.34	1.86
IFNA1	2.08	2.68	1.80
OTTHUMG00000160648	2.08	2.48	1.66
MIR136	2.08	2.94	1.53

MIR1321	2.08	2.95	1.44
TRNAI6	2.08	2.53	1.63
MIR3121	2.08	2.50	1.77
OTTHUMG00000154472	2.08	2.72	1.75
MIR510	2.08	2.65	1.57
HYALP1	2.08	2.59	1.66
OTTHUMG00000152392	2.08	2.54	1.68
MIR4264	2.07	3.03	1.65
STATH	2.07	2.52	1.58
OTTHUMG00000166488	2.07	2.42	1.82
TRAV7	2.07	3.10	1.33
OTTHUMG00000152141	2.07	2.81	1.67
UOX	2.07	2.56	1.76
HMSD	2.07	2.34	1.75
OTTHUMG00000163624	2.07	2.45	1.77
IGHV3-53	2.07	2.84	1.55
LOC284825	2.07	2.35	1.82
AMMECR1-IT1	2.07	2.22	1.82
MTRNR2L5	2.07	2.68	1.67
OTTHUMG00000166293	2.07	2.62	1.73
OTTHUMG00000169639	2.07	2.59	1.66
SNORD121A	2.06	3.23	1.69
ZNF847P	2.06	2.94	1.61
OTTHUMG00000020138	2.06	2.66	1.74
OTTHUMG00000170564	2.06	2.39	1.89
MIR122	2.06	2.51	1.82
OTTHUMG00000009790	2.06	2.68	1.71
PLSCR5	2.06	2.59	1.76
CABS1	2.06	2.31	1.86
SEMG1	2.06	2.41	1.64
OTTHUMG00000014979	2.06	2.37	1.62
RNA5SP249	2.06	2.79	1.62
OTTHUMG00000161192	2.06	2.47	1.59
HBZ	2.06	2.42	1.79
MIR4645	2.06	2.57	1.68
STT3A-AS1	2.06	2.33	1.71
RBM11	2.06	2.21	1.85
OTTHUMG00000153055	2.06	2.46	1.77
LOC100507134	2.06	2.84	1.65
OTTHUMG00000166378	2.06	2.45	1.72
CAPZA3	2.06	2.50	1.85
OTTHUMG00000159435	2.06	2.34	1.84
OR2B3	2.05	2.49	1.72
ADAM21P1	2.05	2.79	1.60
OTTHUMG00000017165	2.05	2.44	1.71
MIR376B	2.05	3.30	1.49
LINC00644	2.05	2.33	1.77
OTTHUMG00000161396	2.05	2.27	1.46
MIR4498	2.05	2.47	1.67

ASZ1	2.05	2.47	1.77
OTTHUMG00000163862	2.05	2.43	1.86
OR9A4	2.05	3.31	1.68
OTTHUMG00000161695	2.05	2.26	1.76
FGF12-AS3	2.04	2.23	1.77
MS4A13	2.04	2.47	1.75
OTTHUMG00000163012	2.04	2.74	1.77
FAM71C	2.04	2.40	1.64
OTTHUMG00000155841	2.04	2.39	1.56
OTTHUMG00000162497	2.04	2.48	1.56
MIR181A1	2.04	2.42	1.70
STOX1	2.04	2.34	1.79
MIR4524A	2.04	3.09	1.35
SNORD116-24	2.04	2.99	1.36
OTTHUMG00000017562	2.04	2.46	1.70
RNA5SP252	2.04	2.60	1.72
OTTHUMG00000017016	2.04	2.22	1.58
RASSF6	2.04	2.46	1.70
TRGV11	2.04	2.34	1.87
MIR4463	2.04	2.87	1.55
IQCF2	2.04	2.47	1.63
OTTHUMG00000160708	2.04	2.71	1.77
OTTHUMG00000037783	2.04	2.86	1.67
MIR4276	2.04	2.90	1.50
RNU5B-1	2.04	3.01	1.34
OTTHUMG00000017755	2.04	2.38	1.63
MIR4282	2.04	2.80	1.19
MTRNR2L9	2.03	2.60	1.47
OTTHUMG00000163223	2.03	2.32	1.77
POU3F4	2.03	2.56	1.75
RNA5SP436	2.03	2.81	1.43
LINC00621	2.03	2.55	1.46
OTTHUMG00000171353	2.03	2.41	1.78
OTTHUMG00000035685	2.03	2.44	1.70
MIR1275	2.03	2.66	1.54
OTTHUMG00000162786	2.03	2.69	1.58
OTTHUMG00000011003	2.03	2.32	1.56
OR6K6	2.03	2.36	1.67
MIR620	2.03	2.55	1.35
OTTHUMG00000162210	2.03	2.39	1.59
ANKRD62P1-PARP4P3	2.03	2.37	1.64
OTTHUMG00000170331	2.03	2.25	1.73
MIR33A	2.03	2.85	1.65
CDKL4	2.02	2.55	1.76
RNU6-77	2.02	2.88	1.14
TRBV30	2.02	2.44	1.67
MIR144	2.02	2.84	1.67
LOC644145	2.02	2.57	1.46
FABP2	2.02	2.18	1.76

OR52L1	2.02	2.28	1.62
LIPJ	2.02	2.24	1.74
SPIC	2.02	2.90	1.58
TRBV6-8	2.02	2.91	1.74
UGT2B11	2.02	3.03	1.23
PTH	2.02	2.35	1.73
OTTHUMG00000161766	2.01	2.31	1.73
EMCN-IT1	2.01	2.38	1.67
RAET1K	2.01	2.43	1.61
OTTHUMG00000158756	2.01	2.30	1.70
OTTHUMG00000019550	2.01	2.25	1.70
GPC5-IT1	2.01	3.15	1.52
OTTHUMG00000170416	2.01	2.52	1.60
LOC728755	2.01	2.46	1.52
OR5D13	2.01	2.34	1.77
SSX5	2.01	2.79	1.51
CTAGE10P	2.01	2.35	1.53
RNA5SP458	2.01	2.46	1.74
LINC00446	2.01	2.43	1.60
LRRIQ3	2.01	2.50	1.80
SPANXN5	2.01	2.78	1.38
EFCAB9	2.01	2.71	1.54
MIR3612	2.01	2.97	1.53
OTTHUMG00000160923	2.01	2.43	1.67
RNA5SP324	2.01	2.45	1.45
OTTHUMG00000018257	2.01	2.66	1.74
RNU4-6P	2.01	2.75	1.54
LOC201651	2.01	2.42	1.81
IFNA7	2.00	3.22	1.43
PRB3	2.00	2.64	1.54
CXorf30	2.00	2.21	1.70
OR1L6	2.00	2.47	1.64
OTTHUMG00000015558	2.00	2.58	1.59
OTTHUMG00000161221	2.00	2.45	1.55
SNORD115-31	2.00	2.99	1.49
OTTHUMG00000078122	2.00	2.42	1.48
SNORD115-41	2.00	2.57	1.36
OTTHUMG00000154344	2.00	2.26	1.49
OTTHUMG00000078330	2.00	2.24	1.67
OTTHUMG00000154820	2.00	2.49	1.66
LINC00492	2.00	2.38	1.62
MIR624	2.00	2.44	1.64
OTTHUMG00000167182	2.00	2.94	1.54
OR4A47	2.00	2.26	1.61
OTTHUMG00000017899	2.00	2.55	1.63
RNA5SP77	2.00	2.88	1.58
OTTHUMG00000161717	2.00	2.23	1.81
LOC643802	1.99	2.97	1.47
LOC100505817	1.99	2.59	1.63

OTTHUMG00000160705	1.99	2.39	1.73
MIR548J	1.99	2.56	1.59
RNA5SP470	1.99	2.58	1.58
IFNA4	1.99	2.48	1.72
RSF1-IT1	1.99	2.19	1.78
OTTHUMG00000159213	1.99	2.40	1.59
HIST1H4G	1.99	2.52	1.60
MIR3152	1.99	2.62	1.74
MACC1-AS1	1.99	2.23	1.62
PCNAP1	1.98	2.31	1.55
CETN4P	1.98	2.28	1.61
RAB9BP1	1.98	2.36	1.54
OTTHUMG00000018126	1.98	2.31	1.58
RNA5SP112	1.98	2.25	1.73
OTTHUMG00000157235	1.98	2.46	1.69
MIR516A2	1.98	2.72	1.47
OR5AC2	1.98	2.69	1.54
TRAJ40	1.98	2.82	1.54
OTTHUMG00000156951	1.98	2.53	1.58
HTN1	1.98	2.88	1.61
UGT2B28	1.98	3.40	1.47
TMEM212-IT1	1.97	2.38	1.77
RNA5SP42	1.97	2.47	1.56
OTTHUMG00000150320	1.97	2.41	1.66
OTTHUMG00000162361	1.97	2.54	1.70
IGHV2-26	1.97	2.99	1.51
CEACAM7	1.97	2.30	1.64
MIR934	1.97	2.19	1.70
RNA5SP301	1.97	2.51	1.55
MIR3202-2	1.97	2.37	1.71
SCEL	1.97	2.42	1.55
SNORD115-45	1.97	2.66	1.44
PPIAL4C	1.97	2.67	1.45
VENTXP1	1.97	2.52	1.55
MIR301A	1.97	2.52	1.46
PCDH9-AS3	1.97	2.64	1.66
OTTHUMG00000164605	1.97	2.25	1.68
MIR517A	1.97	2.63	1.63
LOC729176	1.97	2.25	1.69
MIR217	1.97	2.41	1.73
OTTHUMG00000152871	1.96	2.49	1.70
PSG11	1.96	2.20	1.57
OTTHUMG00000039651	1.96	2.47	1.68
LOC100128139	1.96	2.78	1.50
OTTHUMG00000163234	1.96	2.40	1.58
LINC00333	1.96	2.53	1.50
SNORD114-5	1.96	2.95	1.11
RNA5SP400	1.96	2.43	1.61
OTTHUMG00000161742	1.96	2.74	1.56

OTTHUMG00000017137	1.96	2.28	1.61
OTTHUMG00000159007	1.96	2.22	1.71
EIF1AX-AS1	1.96	2.21	1.77
MIR4251	1.96	2.90	1.48
OTTHUMG00000171031	1.96	2.44	1.60
OTTHUMG00000155405	1.95	2.45	1.67
LINC00583	1.95	2.19	1.70
CCDC73	1.95	2.18	1.78
OTTHUMG00000160741	1.95	2.21	1.72
RPS4XP21	1.95	2.16	1.66
MIR3919	1.95	2.57	1.64
MIR3124	1.95	2.24	1.56
OTTHUMG00000164447	1.95	2.52	1.57
OTTHUMG00000033149	1.95	2.17	1.72
SNORD115-24	1.95	3.26	1.47
IFNW1	1.95	2.29	1.67
OR5T3	1.95	2.23	1.60
OTTHUMG00000018301	1.95	2.49	1.67
OTTHUMG00000153762	1.95	2.38	1.58
MIR4795	1.95	2.42	1.57
MIR586	1.95	2.26	1.66
OTTHUMG00000152624	1.94	2.14	1.70
OR2T29	1.94	2.49	1.62
C2orf27B	1.94	2.42	1.71
MIR3908	1.94	2.93	1.29
VCX	1.94	2.28	1.67
IL21	1.94	2.13	1.74
MIR517C	1.94	2.51	1.51
MIR3158-1	1.94	2.57	1.56
SNORD93	1.94	2.31	1.62
RNA5SP142	1.94	2.51	1.68
CFHR2	1.94	2.73	1.45
KRTAP9-6	1.94	2.48	1.59
TAS2R8	1.94	2.34	1.69
MIR106A	1.94	2.43	1.38
RNA5SP404	1.94	2.35	1.53
MIR411	1.93	2.49	1.31
OR8J3	1.93	2.34	1.61
OR5AN1	1.93	2.46	1.49
OR5H15	1.93	2.51	1.49
RNA5SP38	1.93	2.70	1.58
CTSL1P8	1.93	2.59	1.38
OTTHUMG00000171064	1.93	2.28	1.61
OTTHUMG00000177363	1.93	2.41	1.54
C1QTNF9-AS1	1.93	2.55	1.38
HIST1H3B	1.93	3.10	1.34
OTTHUMG00000011035	1.93	2.21	1.72
SNORD18B	1.93	2.97	1.22
PPP1R2P3	1.93	2.50	1.56

RNA5SP389	1.93	2.71	1.47
MIR876	1.93	2.25	1.68
OTTHUMG00000161660	1.93	2.19	1.59
RNA5SP454	1.93	2.83	1.62
OTTHUMG00000166133	1.93	2.24	1.77
SPINK14	1.93	2.45	1.62
OTTHUMG00000021315	1.93	2.05	1.70
OR5M3	1.92	2.51	1.53
OTTHUMG00000165512	1.92	2.32	1.59
ZNF385D-AS2	1.92	2.20	1.64
MIR1207	1.92	2.54	1.76
OTTHUMG00000046310	1.92	2.18	1.58
MIR3684	1.92	2.33	1.64
OTTHUMG00000003394	1.92	2.26	1.72
DEFB112	1.92	2.42	1.60
OTTHUMG00000164038	1.92	2.45	1.59
OTTHUMG00000022736	1.92	2.31	1.68
OR2J1	1.91	3.02	1.41
MIR3910-1	1.91	2.55	1.58
OTTHUMG00000168228	1.91	2.18	1.63
MIR4286	1.91	2.43	1.47
LINC00387	1.91	2.31	1.47
UGT2B17	1.91	2.48	1.34
MIR4768	1.91	2.36	1.65
OTTHUMG00000160100	1.91	2.46	1.44
OTTHUMG00000166110	1.91	2.27	1.59
OTTHUMG00000153377	1.91	2.14	1.65
OTTHUMG00000168930	1.91	2.17	1.67
TAS2R38	1.91	2.40	1.59
MIR4278	1.91	2.45	1.57
OTTHUMG00000039564	1.91	2.34	1.59
RNA5SP155	1.90	2.61	1.59
IL2	1.90	2.18	1.47
RNA5SP384	1.90	2.28	1.65
MIR379	1.90	2.86	1.50
OTTHUMG00000170385	1.90	2.18	1.69
PCDHB8	1.90	2.48	1.64
MIR519E	1.90	2.71	1.39
OTTHUMG00000160709	1.90	2.49	1.47
GPR174	1.90	2.63	1.63
IGHV1-18	1.90	2.81	1.43
OTTHUMG00000169533	1.89	2.30	1.47
MIR3118-1	1.89	2.22	1.64
MIR548S	1.89	2.93	1.33
RNA5SP368	1.89	2.46	1.50
HIST1H2AJ	1.89	2.61	1.51
MIR625	1.89	2.87	1.35
RNA5SP225	1.89	2.26	1.54
MIR576	1.89	2.36	1.52

OTTHUMG00000164905	1.89	2.15	1.43
SUMO1P1	1.89	2.36	1.39
RNA5SP219	1.88	2.19	1.49
SKA2P1	1.88	2.52	1.44
MIR4308	1.88	2.33	1.58
RAET1L	1.88	2.53	1.60
SNORD116-25	1.88	2.65	1.49
MIR4491	1.88	2.70	1.52
OTTHUMG00000156184	1.88	2.42	1.60
OTTHUMG00000134296	1.88	2.15	1.68
ZNF99	1.88	2.80	1.37
MIR3201	1.87	2.33	1.43
HIST1H2AD	1.87	2.38	1.48
MIR4762	1.87	2.12	1.63
MIR3675	1.87	2.10	1.64
IGKV1D-43	1.87	2.64	1.44
OR2C1	1.87	2.33	1.34
OTTHUMG00000015852	1.87	2.37	1.53
OTTHUMG00000170042	1.87	2.20	1.62
OTTHUMG00000015256	1.87	2.51	1.47
OR2AE1	1.87	2.25	1.52
OTTHUMG00000160622	1.87	2.52	1.52
OTTHUMG00000166896	1.87	2.25	1.58
OTTHUMG00000009840	1.87	2.39	1.56
SSX1	1.86	2.08	1.36
C1orf137	1.86	2.16	1.59
OTTHUMG00000169688	1.86	2.17	1.57
HELLPAR	1.86	2.74	1.41
OR8K1	1.86	2.55	1.63
MIR4499	1.86	2.31	1.54
OTTHUMG00000164897	1.86	2.01	1.63
HIST1H1T	1.86	2.30	1.63
MIR4679-2	1.85	2.51	1.35
MIR3144	1.85	2.59	1.35
LINC00290	1.85	2.07	1.59
RNA5SP227	1.85	2.86	1.55
TRAV9-2	1.85	2.13	1.54
MIR588	1.85	2.17	1.65
FAM46D	1.85	2.05	1.54
POTEA	1.85	2.12	1.58
OTTHUMG00000019260	1.85	2.31	1.58
RNA5SP198	1.85	2.48	1.60
OR10A6	1.85	2.91	1.55
OTTHUMG00000164267	1.85	2.17	1.62
MIR523	1.85	2.20	1.38
MIR3912	1.84	2.25	1.57
OTTHUMG00000017940	1.84	2.26	1.40
MIR4510	1.84	2.14	1.61
UPP2-IT1	1.84	2.16	1.67

MIR3145	1.84	2.35	1.22
TMEM30C	1.84	2.02	1.63
OTTHUMG00000019022	1.84	2.17	1.50
OTTHUMG00000017053	1.84	2.34	1.52
OR13C5	1.84	2.41	1.44
MIR562	1.84	2.21	1.44
OTTHUMG00000169689	1.84	2.75	1.27
MIR522	1.84	2.18	1.45
MIR4318	1.83	2.30	1.60
CTD-2542C24.4	1.83	2.36	1.27
SERPINB11	1.83	2.11	1.61
OTTHUMG00000169099	1.83	2.06	1.55
OTTHUMG00000144165	1.83	2.03	1.63
RNA5SP89	1.83	2.46	1.38
MIR451A	1.83	2.04	1.53
OTTHUMG00000163604	1.83	2.33	1.48
MIR4285	1.83	2.25	1.52
OTTHUMG00000162184	1.82	2.31	1.37
OTTHUMG00000017187	1.82	2.13	1.55
OTTHUMG00000015981	1.82	2.39	1.52
RNU6-54	1.82	2.11	1.35
OR7A17	1.82	2.41	1.50
RNA5SP98	1.82	2.65	1.54
MIR190A	1.82	2.53	1.54
SNORD60	1.82	2.27	1.37
LSAMP-AS2	1.82	2.25	1.50
SNORD115-3	1.82	2.58	1.44
SPRR2E	1.82	1.97	1.51
OTTHUMG00000162575	1.82	2.00	1.54
RNVU1-13	1.82	2.34	1.41
RNA5SP216	1.81	2.39	1.41
OTTHUMG00000086915	1.81	2.22	1.44
MIR891B	1.81	2.14	1.46
RLN2	1.81	2.32	1.49
OTTHUMG00000021486	1.81	2.06	1.62
OTTHUMG00000163114	1.81	2.14	1.49
AGR3	1.81	2.00	1.61
MIR4759	1.81	2.94	1.25
OR8H1	1.81	2.38	1.48
C1orf143	1.81	2.10	1.44
OTTHUMG00000166087	1.81	1.95	1.58
OTTHUMG00000162993	1.81	2.19	1.58
MIR4423	1.81	2.18	1.50
TRDJ4	1.81	2.57	1.36
MIR3938	1.81	2.32	1.43
MIR3140	1.81	2.20	1.46
MIR653	1.80	2.03	1.60
OR6C6	1.80	2.26	1.26
MIR618	1.80	1.97	1.57

MIR526B	1.80	2.68	1.35
PSG7	1.80	2.65	1.43
OTTHUMG00000172358	1.80	2.13	1.60
MIR30D	1.80	2.28	1.50
MIR4693	1.79	2.58	1.44
OTTHUMG00000158042	1.79	2.09	1.53
OTTHUMG00000152955	1.79	2.25	1.50
MIR548AM	1.79	2.54	1.27
MIR4477A	1.79	2.39	1.29
IGLV5-37	1.79	2.09	1.44
SNORD11B	1.79	2.21	1.49
SNORD116-16	1.78	2.33	1.43
OTTHUMG00000154464	1.78	1.96	1.60
FABP12	1.78	2.05	1.56
PSG1	1.78	2.93	1.46
RNA5SP54	1.78	2.21	1.57
SNORD113-6	1.78	3.11	1.31
MIR3914-1	1.78	2.26	1.39
OR10A3	1.78	2.23	1.47
OTTHUMG00000178158	1.78	2.15	1.34
OTTHUMG00000162806	1.78	2.11	1.52
LINC00314	1.78	2.06	1.48
MIR3646	1.77	2.30	1.27
C1QTNF9B	1.77	2.45	1.48
OTTHUMG00000153849	1.77	2.08	1.63
MIR367	1.77	2.22	1.46
MIR378D1	1.77	2.29	1.35
MIR4427	1.77	2.31	1.21
MIR1283-1	1.77	2.46	1.46
OR5H1	1.77	1.93	1.57
TTTY11	1.77	2.09	1.60
OTTHUMG00000159502	1.77	2.16	1.50
OTTHUMG00000074546	1.77	2.21	1.56
FAM74A1	1.77	2.35	1.37
SNORD115-33	1.76	2.24	1.53
MIR302E	1.76	2.05	1.47
OTTHUMG00000020126	1.76	2.11	1.38
FAM223A	1.76	2.02	1.46
OTTHUMG00000015760	1.76	2.18	1.53
OTTHUMG00000162017	1.76	2.23	1.51
IGKV1-5	1.76	2.05	1.50
MIR3117	1.76	2.38	1.25
RNA5SP468	1.76	2.35	1.34
OTTHUMG00000164654	1.76	2.11	1.37
OR11H4	1.76	2.10	1.56
MIR4483	1.76	2.30	1.41
OTTHUMG00000163145	1.75	2.04	1.53
OTTHUMG00000015958	1.75	2.12	1.41
KRTAP12-1	1.75	2.18	1.51

TRAC	1.75	2.13	1.38
TAS2R7	1.75	1.94	1.57
SNORA76	1.75	2.17	1.22
MIR4520A	1.74	2.00	1.61
OTTHUMG00000160975	1.74	2.13	1.46
OTTHUMG00000162781	1.74	1.90	1.51
OR2T8	1.74	2.10	1.30
IGKV6D-41	1.74	2.37	1.29
MIR4320	1.74	2.04	1.41
MIR3921	1.74	2.07	1.48
OTTHUMG00000163741	1.74	1.96	1.60
OTTHUMG00000016811	1.74	2.04	1.44
IFNA21	1.74	2.81	1.43
OTTHUMG00000078807	1.74	2.13	1.40
MIR3672	1.74	1.92	1.46
RNA5SP275	1.74	2.13	1.51
CCDC168	1.74	2.15	1.41
RNA5SP445	1.74	2.60	1.45
MIR2053	1.74	2.11	1.31
RNA5SP173	1.74	2.16	1.25
MIR4439	1.73	2.44	1.41
SLCO1B7	1.73	2.10	1.40
MIR4791	1.73	2.26	1.43
OR51B2	1.73	2.17	1.36
MIR4802	1.73	2.06	1.37
SNORD116-13	1.73	2.20	1.42
SNORD115-27	1.73	1.99	1.23
SNORD116-18	1.73	2.07	1.50
RNU7-56P	1.73	2.00	1.36
IGHD2-8	1.72	2.60	1.29
RNA5SP397	1.72	2.16	1.41
OR10A5	1.72	2.53	1.44
UBD	1.72	2.26	1.31
LOC729080	1.72	2.16	1.55
RNU5F-8P	1.72	2.17	1.37
OTTHUMG00000164603	1.71	2.03	1.48
MIR376C	1.71	2.08	1.46
RNU4-10P	1.71	2.24	1.31
OR5K4	1.71	2.35	1.39
RNA5SP271	1.71	2.12	1.34
RNA5SP178	1.71	2.67	1.26
MIR1305	1.71	2.05	1.40
MIR633	1.71	1.95	1.45
OTTHUMG00000162874	1.71	2.06	1.28
OTTHUMG00000014284	1.71	2.06	1.52
OTTHUMG00000152880	1.71	2.25	1.30
OTTHUMG00000167602	1.70	2.13	1.46
MIR507	1.70	2.07	1.35
RFPL3	1.70	2.21	1.41

RNA5SP169	1.70	2.19	1.23
MIR135A2	1.70	2.18	1.29
OTTHUMG00000163548	1.70	2.00	1.36
RNU7-20P	1.70	2.40	1.29
HIST3H2A	1.70	2.32	1.39
MIR4738	1.70	2.39	1.38
ZNF322P1	1.70	2.10	1.31
OTTHUMG00000153807	1.70	1.96	1.43
MIR1-2	1.69	1.98	1.44
OR13C2	1.69	2.16	1.39
MIR548W	1.69	2.23	1.22
OR1Q1	1.69	1.98	1.45
OR52I2	1.69	1.93	1.37
RNU5A-1	1.69	2.13	1.50
MIR3591	1.69	2.22	1.30
SNORD115-38	1.69	2.04	1.37
OTTHUMG00000158209	1.69	2.00	1.30
MIR4720	1.69	2.11	1.41
MIR561	1.69	2.13	1.40
RNA5SP269	1.69	1.97	1.37
OTTHUMG00000164593	1.69	2.13	1.36
SNORD115-2	1.68	2.68	1.42
MIR30B	1.68	2.25	1.43
OTTHUMG00000164806	1.68	1.94	1.52
MIR1252	1.68	2.03	1.50
OTTHUMG00000132249	1.68	2.06	1.28
MIR302F	1.68	2.27	1.38
RNA5SP281	1.68	2.03	1.38
MIR4475	1.68	2.20	1.27
MIR518E	1.68	2.29	1.30
MIR892B	1.68	2.15	1.33
TSPAN19	1.67	2.03	1.37
OTTHUMG00000151293	1.67	2.22	1.42
FMR1-IT1	1.67	1.98	1.28
MIR3688-1	1.67	2.18	1.24
OTTHUMG00000035609	1.67	1.97	1.30
OTTHUMG00000162941	1.67	2.06	1.35
MIR1322	1.66	2.03	1.42
MIR203	1.66	1.84	1.47
FRMPD4-AS1	1.66	1.85	1.39
MIR4471	1.66	2.23	1.19
OR9K2	1.66	2.08	1.46
MIR4705	1.66	1.96	1.34
MIR378A	1.66	2.12	1.22
OTTHUMG00000036749	1.65	2.00	1.43
MIR3920	1.65	1.97	1.46
OR5K3	1.65	2.02	1.45
MIR3146	1.65	2.12	1.35
MIR759	1.65	2.17	1.30

OTTHUMG00000020999	1.65	2.48	1.34
OR2M2	1.65	2.12	1.21
OTTHUMG00000164188	1.64	1.90	1.36
RNA5SP94	1.64	2.02	1.37
OTTHUMG00000165818	1.64	2.26	1.28
IFNA10	1.64	2.78	1.22
RAB40AL	1.64	1.98	1.37
OR12D3	1.64	2.43	1.29
MIR4490	1.64	2.19	1.36
HIST1H3C	1.64	1.97	1.35
MIR29B1	1.63	2.20	1.25
LINC00352	1.63	2.23	1.15
SNORD115-15	1.63	2.01	1.35
MIR384	1.63	1.99	1.30
MIR4676	1.63	1.96	1.27
MIR559	1.63	2.28	1.26
SNORD119	1.62	1.87	1.41
MIR4464	1.62	1.87	1.37
VTRNA3-1P	1.62	1.83	1.42
MIR4671	1.62	1.96	1.34
MIR208B	1.62	1.84	1.43
MIRLET7F2	1.61	1.84	1.35
MIR556	1.61	2.25	1.41
SNORD115-30	1.61	2.11	1.22
OTTHUMG00000017840	1.61	2.02	1.28
MIR450B	1.61	2.12	1.42
MIR519B	1.60	2.23	1.36
RNU5E-5P	1.60	2.39	1.17
OTTHUMG00000170546	1.60	2.16	1.32
MIR3119-1	1.60	1.97	1.20
MIR4272	1.60	2.25	1.33
RNA5SP109	1.59	1.83	1.33
SNORD115-8	1.59	1.91	1.31
MIR92A2	1.59	1.99	1.26
SPANXN1	1.59	1.98	1.27
TRAJ2	1.59	2.13	1.16
RNA5SP48	1.58	1.85	1.32
LOC100287612	1.58	2.09	1.27
MIR4445	1.58	2.20	1.21
UGT2B15	1.58	2.60	1.24
MIR3173	1.58	2.04	1.31
SSX3	1.58	1.95	1.26
MIR450A2	1.57	2.08	1.24
MIR1279	1.57	2.22	1.38
MIR4662A	1.57	2.21	1.26
OTTHUMG00000182183	1.57	1.93	1.30
SNORD115-14	1.57	2.15	1.30
MIR4777	1.57	1.91	1.24
RNA5SP63	1.57	2.02	1.39

OR5K1	1.57	2.32	1.21
MIR4712	1.57	1.93	1.29
OTTHUMG00000162791	1.57	1.81	1.25
OTTHUMG00000162833	1.56	1.91	1.37
SLC9B1P1	1.56	2.16	1.28
SNORD115-10	1.56	1.94	1.09
MIR4790	1.56	2.16	1.36
SNORD115-28	1.56	1.79	1.29
MIR569	1.56	1.99	1.36
SNORD115-40	1.56	1.99	1.28
MIR4735	1.55	1.82	1.36
MIR3910-2	1.55	1.79	1.35
MIR4465	1.55	1.77	1.39
RNA5SP502	1.55	1.90	1.14
MIR518F	1.55	1.85	1.19
OR2T33	1.55	1.84	1.35
RNY3P5	1.54	1.77	1.27
TUBB8P7	1.54	1.86	1.24
SNORD115-48	1.54	1.97	1.27
RNA5SP371	1.54	1.86	1.10
MIR651	1.53	1.72	1.34
GYG2-AS1	1.53	1.86	1.23
FAM74A6	1.53	2.04	1.16
RNA5SP192	1.53	1.85	1.25
KRTAP9-9	1.52	1.99	1.28
MIR4536-1	1.52	2.08	1.30
MIR3202-1	1.52	1.81	1.30
MIR3167	1.52	2.12	1.25
SNORD115-47	1.51	1.92	1.28
MIR1297	1.51	1.87	1.34
OTTHUMG00000169038	1.51	1.81	1.31
TGIF2LX	1.51	1.80	1.10
RNU5A-3P	1.51	2.31	1.10
SNORD115-35	1.51	1.82	1.24
SNORD115-21	1.50	2.08	1.19
MIR3545	1.50	1.76	1.36
MIR450A1	1.50	1.98	1.17
MIR519C	1.50	1.86	1.19
RNA5SP23	1.50	1.87	1.26
MIR4521	1.50	2.18	1.05
MIR124-3	1.49	1.85	1.31
MIR3156-1	1.49	1.73	1.22
TRGC2	1.48	2.24	1.24
SNORD115-37	1.46	1.68	1.24
MIR526A1	1.46	1.81	1.15
MIR3923	1.46	1.74	1.19
MIR4766	1.45	1.79	1.17
MIR369	1.44	1.72	1.25
SNORD115-4	1.43	1.72	1.09

NCOR1P1	1.43	1.65	1.21
MIR3668	1.43	1.79	1.14
RNU5A-2P	1.41	1.69	1.18
RNA5SP147	1.40	1.67	1.13
MIR376A1	1.39	1.75	1.08
SNORD115-34	1.38	1.85	1.11
RNA5SP329	1.38	1.70	1.14
